# Intraplate deformation of Gondwana terranes and implications for the Wilson Cycle

**DOI:** 10.1038/s41598-025-27764-6

**Published:** 2025-11-21

**Authors:** Ana Fonseca, Johan De Grave

**Affiliations:** 1 Institut Für Geowissenschaften, Universität Potsdam, Potsdam, Germany; 2 Laboratory for Mineralogy and Petrology, Department of Geology, Ghent University, Ghent, Belgium

**Keywords:** Geology, Structural geology

## Abstract

The Wilson Cycle, a cornerstone of plate tectonic theory, describes the cyclical evolution of ocean basins from rifting and spreading to closure via subduction and continental collision and assembly. While pivotal for understanding plate boundary processes, it remains limited in addressing intraplate deformation. This study revisits the Wilson Cycle by investigating intraplate deformation and mechanisms of tectonic stress partitioning in (Pre)Cambrian cratons and different orogen types in Southeast Brazil, Southeast Colombia, and Peninsular India. We focus on cratonic areas (Amazon, São Francisco and Dharwar Cratons) that are traditionally considered stable since their incorporation in Gondwana and its subsequent disintegration. We also consider some of the fringing orogens or mobile belts (Brasília and Araçuaí Orogens). Using apatite fission-track thermochronology, we analyze the timing, magnitude, and drivers of deformation and show significant variability in tectonic responses. Some cratons (Amazon and Dharwar) demonstrate unexpected exhumation driven by extensional tectonics, while others (São Francisco Craton) exhibit only localized reactivations and remain largely stable. Similarly, orogens follow distinct evolutionary paths: e.g. the Brasília Orogen became resistant to post-Gondwana deformation, effectively stagnating the Wilson Cycle, while the Araçuaí Orogen experienced intense reactivation and renewed tectonic activity. These examples give new insights into the traditional Wilson Cycle, demonstrating that intraplate deformation plays a critical role in sustaining or altering the cycle, implying the traditional concept is in need of significant revision.

**Supplementary Information:**

The online version contains supplementary material available at 10.1038/s41598-025-27764-6.

## Introduction

**Fig. 1:**
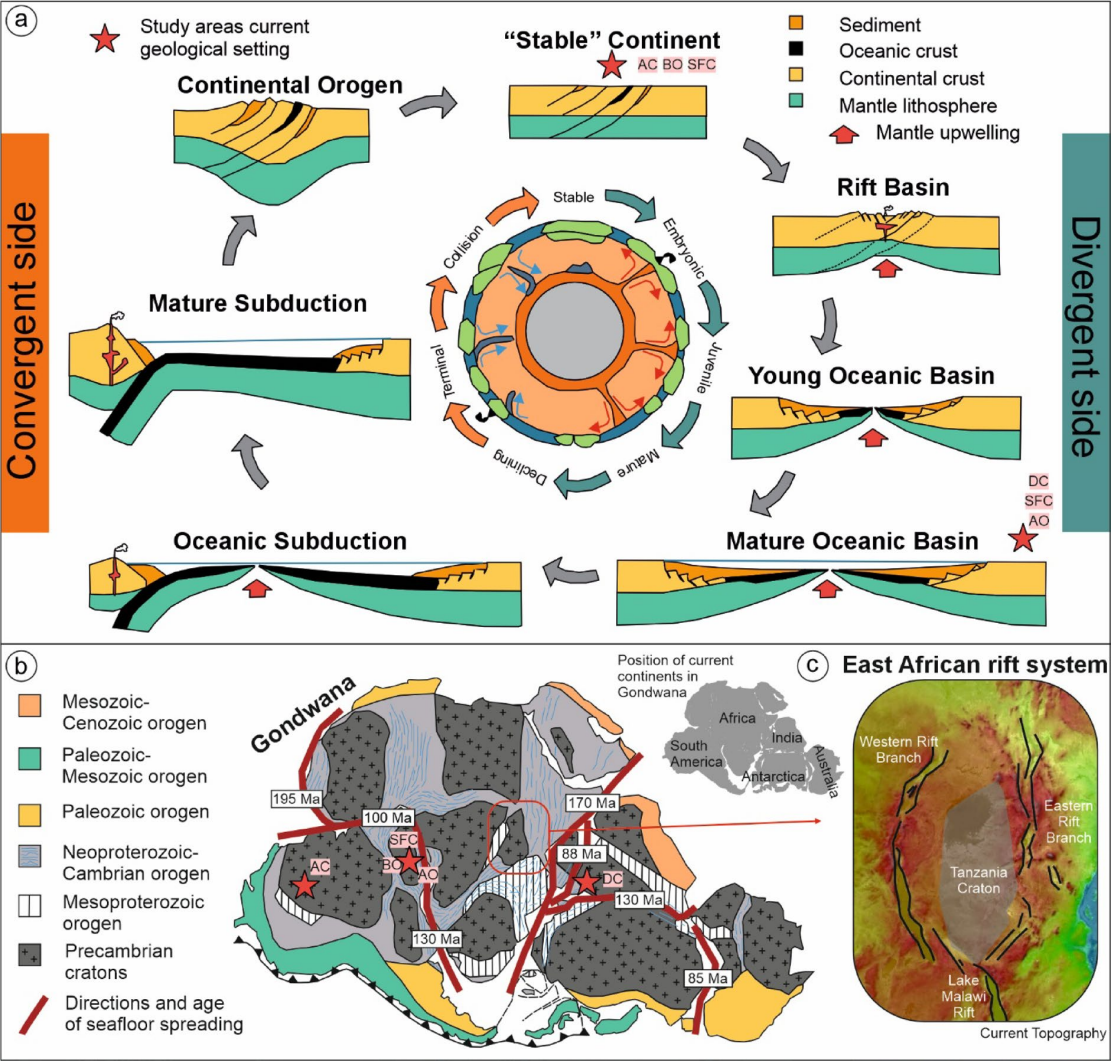
The Wilson Cycle and its implications for basin evolution and continental deformation. (**a**) Schematic representation of the Wilson Cycle (modified from^34^), illustrating the cyclic evolution of ocean basins through successive stages of rifting, oceanic spreading, subduction, and continental collision. The divergent phase is characterized by lithospheric extension, crustal thinning, and subsidence, leading to the formation of rift basins and oceanic basins at spreading centers. The convergent phase involves subduction, island arc development, and eventual continental orogeny, culminating in a stabilized continental configuration marked by minimal tectonic activity. Study areas examined here are indicated by red stars. (**b**) Reconstruction of Gondwana breakup, demonstrating that rift propagation preferentially followed pre-existing orogenic belts, consistent with Wilson Cycle predictions. (**c**) The East African Rift System exemplifies an active rift, where extensional deformation predominantly occurs along former orogenic belts while cratonic regions, such as the Tanzania Craton, remain largely unaffected. This pattern aligns with Wilson Cycle predictions, reinforcing the role of inherited lithospheric structures in continental deformation.

The theory of plate tectonics, firmly established by the late 1960s, revolutionized our understanding of Earth’s dynamic structure^1^. Previously perceived as static, the solid Earth was unveiled as a complex, dynamic and mobile system. In plate tectonic theory, tectonic activity is primarily concentrated along narrow zones at plate margins, with significantly less deformation occurring within the plates themselves. The Wilson Cycle further refined this theory by describing the lifecycle of ocean basins, from their formation through rifting and oceanic spreading to their eventual destruction via subduction and continental collision^2^ (Fig. 1).

When J. Tuzo Wilson first introduced the Wilson Cycle in his seminal paper, "Did the Atlantic close and then re-open?"^2^, he proposed that oceans close and reopen along former sutures or plate collision zones. These collision zones thus often develop orogenic belts that undergo multiple deformation events and repeatedly become part of plate boundaries. In contrast, highly stable regions are generally not expected to be affected by the Wilson Cycle and instead remain within the interior of continents over geological time. These areas are anchored by the rigid and buoyant lithosphere of cratons.

The Wilson Cycle provides a compelling framework for understanding global geodynamics. For instance, it explains why the Atlantic Ocean opened along the main structures of Neoproterozoic-Cambrian orogenic belts^3^ (Fig. 1) or why the Eastern African Rift System edges along the Tanzania Craton^4^, with rifting preferentially occurring in weaker orogenic lithospheres rather than within cratonic interiors (Fig. 1).

However, while the Wilson Cycle successfully accounts for some of these large-scale geological processes at plate boundaries, it struggles to explain intraplate deformation observed within continental interiors, often hundreds to thousands of kilometers away from any active plate boundary. For example, intracontinental orogens, such as e.g. the Tian Shan orogen in Central Asia, represent a significant deviation from the traditional Wilson Cycle model of orogeny, which is typically associated with convergent plate boundaries. These intracontinental orogens form when compressive stresses generated at distant plate boundaries are transmitted through the lithosphere^5,6^. In addition, intracratonic basins serve as another critical example of intracontinental deformation not incorporated in the Wilson Cycle model. These basins represent large depressions with prolonged and episodic subsidence that coincides with intermittent periods of apparent tectonic activity and stability within the continental interiors^7^. The mechanisms driving such intraplate deformation remain debated, but these observations suggest that continental lithosphere evolves in ways not fully captured by the classical Wilson Cycle^8^.

Another limitation of the Wilson Cycle is the assumption that tectonic evolution follows a predictable sequence, such as an orogen inevitably transitioning into a rift basin. However, the tectonic fate of a given region is not predetermined; alternative pathways exist, shaped by variations in lithospheric architecture, inherited structures, and evolving geodynamic conditions.

**Fig. 2:**
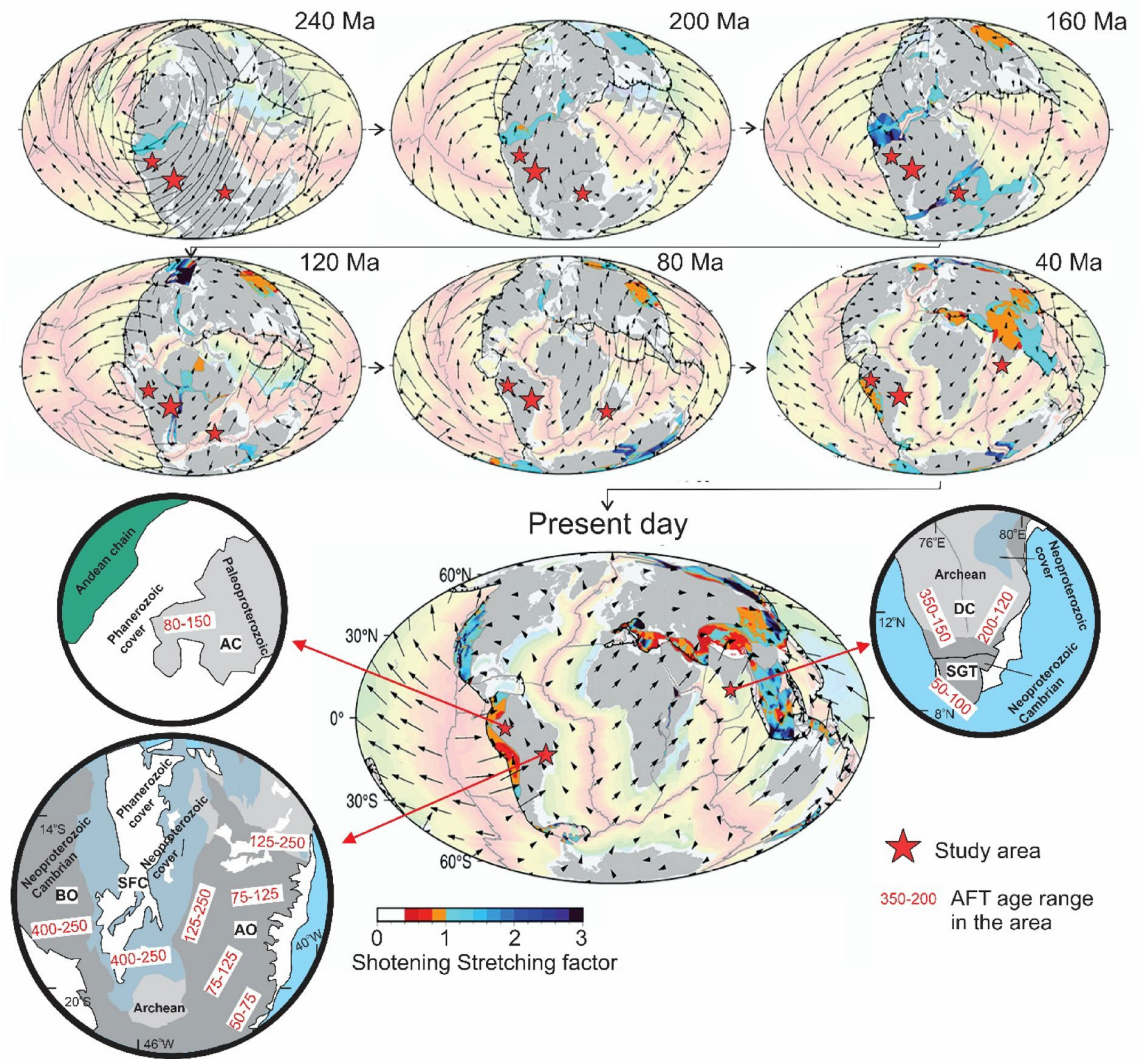
Plate reconstructions for the Triassic to present. Deforming regions are outlined in light to dark blue colors if in extension, denoted by a stretching factor > 1, while plate shortening is outlined in orange/red colors denoted by a shortening factor < 1 (modified from^54^). Light colors in the oceanic domains refer to the relative age of the ocean crust, with reddish colors indicating more recent crust and greenish colors indicating older crust. The study areas and the AFT age ranges are highlighted. BO—Brasília Orogen; SFC—São Francisco Craton; AO—Araçuaí Orogen; AC—Amazon Craton; DC—Dharwar Craton; SGT—Southern Granulite Terrane.

Here, we bridge these gaps by critically reevaluating the Wilson Cycle in light of intraplate deformation. Specifically, we examine three key limitations of the traditional model: (1) its failure to explicitly account for intraplate deformation as a distinct and significant phase of the tectonic cycle, (2) its implicit assumption that geological inheritance primarily governs plate boundary processes, overlooking its crucial role in intraplate settings, and (3) its deterministic view that a tectonic environment will inevitably transition into a predetermined state, neglecting alternative evolutionary pathways. To address these gaps, we investigate the Phanerozoic exhumation history of basement rocks in Gondwana terrains of Southeast (SE) Brazil^9–12^, SE Colombia^13^, and Peninsular India^14^. These regions, traditionally regarded as tectonically stable continental interiors and passive margins (Fig. 2), exhibit evidence of long-term intraplate deformation. Using our published low-temperature thermochronology, particularly apatite fission-track (AFT) data, we assess where the Wilson Cycle framework applies, where it does not, and how it can be refined. Our goal is to provide a more comprehensive understanding of the long-term geodynamics of the continental lithosphere.

### Reevaluating intraplate stability: Phanerozoic exhumation of Gondwana terranes

Central to AFT data interpretation is the principle that rock exhumation from deeper levels of the Earth’s crust to the surface is accompanied by a significant rock cooling^15^. Exhumation and consequential cooling are often driven by faulting and/or uplift, followed by the erosion of the overlying rock cover, progressively exposing deeper rock units to cooler upper crustal and surface conditions. By determining the timing of cooling, AFT analysis provides an indirect record of exhumation and vertical rock movements, offering key insights into past tectonic and erosional events.

**Fig. 3:**
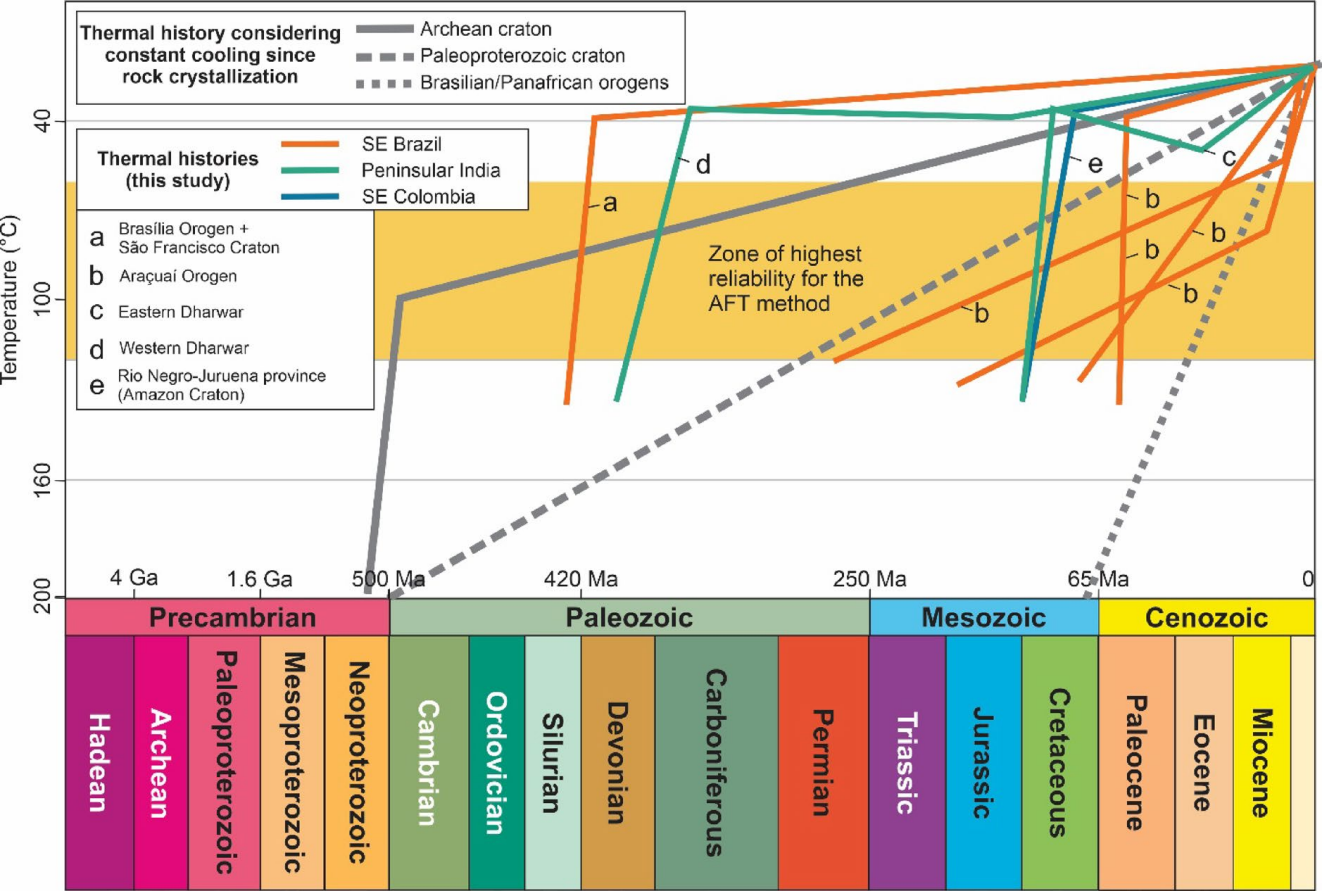
Comparison between thermal histories. In gray, the thermal histories are shown based on the assumption of continuous cooling at a constant rate of the crystalline rocks since formation. Orange, green, and blue lines illustrate simplified versions of the thermal histories constrained by thermochronology, highlighting the main trends. The different thermal histories derived from our data demonstrate the non-uniqueness of exhumation histories of the Gondwana basement. The timescale is compressed for the Precambrian relative to the Phanerozoic to accommodate different temporal resolutions. This figure is a representative schematic summarizing first-order patterns from hundreds of individual thermal history models and is not meant to display individual model uncertainties. Full model outputs, including uncertainty envelopes and representative examples, are provided in the Supplementary data.

Based on the concept of intraplate stability, one might expect that the stable intracontinental regions of Gondwana, as the ones studied here, to have undergone only a gradual and slow erosion and exhumation from their consolidation to the present (Fig. 3). However, the AFT data (Supplementary data) provide clear evidence that the “stable” basement within Gondwana experienced spatial and temporal heterogeneous exhumation throughout the Phanerozoic (Fig. 3).

In SE Brazil, our investigation covers the São Francisco Craton (SFC), an Archean-Paleoproterozoic craton, and two of its bounding Neoproterozoic-Cambrian mobile belts or orogens: the Brasília and Araçuaí orogens^16^. The Brasília Orogen (BO) is a “dead” orogen^17^, having remained within the continental interior of Gondwana and later South America since its early Phanerozoic formation. In contrast, the Araçuaí Orogen (AO) evolved into a passive margin following the disintegration of West Gondwana and the ensuing opening of the South Atlantic Ocean. This resulted in the rifting and separation of the AO into an African (West Congo orogen) and a South American (AO) segment^18^. The AFT results indicate rapid cooling in the AO during the Mesozoic and Cenozoic, in stark contrast to the SFC and BO, which underwent earlier exhumation during the Paleozoic and remained thermally stable (< 60 °C) from the Mesozoic onward (Fig. 3).

In SE Colombia, we analyzed samples from the Rio Negro–Juruena basement^19^, part of the Amazonian Craton (AC). The Rio Negro–Juruena basement consists of Paleoproterozoic granitoids and Mesoproterozoic anorogenic plutons. Throughout the Phanerozoic, this area remained within the continental interior of Gondwana and later South America. During the Jurassic to early Cretaceous, the region bordered Mesozoic Andean basins, which developed as a back-arc rift system coeval with the opening of the Equatorial Atlantic Ocean. The inverse thermal history derived from AFT data indicates a rapid basement cooling of the AC in the early Cretaceous (Fig. 3).

Southern Peninsular India encompasses the Archean Dharwar Craton (DC) and the Southern Granulite Terrane (SGT), a complex mosaic of several crustal blocks and intervening collisional sutures and shear zones that developed during the Mesoarchean to late Neoproterozoic-Cambrian^20^. Thermal history models indicate significant Jurassic basement cooling along the eastern margin of the DC, whereas the western side of the craton appears to have been more resilient to deformation during the Mesozoic and Cenozoic. Limited AFT data from the SGT suggest that exhumation was more extensive during the Cenozoic (Fig. 3).

### Phanerozoic intraplate tectonism of the Gondwana basement: timing, controls, and geological context

The earliest basement cooling event identified in our AFT dataset spans the Silurian to Carboniferous (c. 430 and 350 Ma) and occurred shortly after the final assembly of Gondwana. This broad timeframe is based on results obtained through thermal history modelling of samples from the BO, the SFC, and the western DC (Fig. 3). Similar Paleozoic cooling signals have also been widely documented in thermochronological studies across ancient West Gondwana terranes in South America and Africa^21–27^, as well as in Australian cratonic terranes^28,29^. These findings indicate that post-assembly processes exerted a widespread influence on the exhumation and thermal evolution of the Gondwana basement during the Paleozoic.

A growing body of evidence, including our findings, supports the hypothesis that far-field tectonic forces played a key role in driving uplift and exhumation within the interior of Gondwana during the Paleozoic. At this time, the supercontinent experienced long-lived orogenies along its southern margin while vast sedimentary basins developed within its interior. It was proposed^30^ that a genetic link existed between the intracratonic basins and the Famatinian and Gondwanan orogenic cycles (490–450 Ma and 320–250 Ma, respectively). These orogenic events likely triggered flexural lithospheric buckling and folding, simultaneously leading to the subsidence of some areas while others experienced uplift^31^. This dynamic interplay may have promoted basement denudation in uplifted regions adjacent to intraplate basins, ultimately resulting in the exhumation patterns recorded in our thermochronological dataset.

In regions where AFT data primarily record younger exhumation events (Mesozoic and Cenozoic), it is plausible that earlier Paleozoic cooling episodes also affected the basement but are no longer preserved. This might be the result of thermal overprinting by subsequent tectono-thermal events or the erosion of upper crustal layers that initially retained the Paleozoic cooling signature. These scenarios are likely in regions such as the AO and the Eastern DC, where Paleozoic cooling signatures are absent and the AFT records (much) younger basement denudation.

To better test whether a Paleozoic cooling signal is indeed missing, the integration of additional thermochronometers (e.g., zircon fission-track and zircon (U–Th)/He) would be highly beneficial. However, such data are currently scarce for most of our study areas, in part due to the analytical challenges of working with cratonic lithologies (e.g., high U content, prolonged accumulation of radiation damage, and age overdispersion in zircons). Addressing these limitations through future multi-method thermochronology would provide a more complete resolution of early Paleozoic cooling signals in thermochronometry datasets and help to assess its spatial extent.

In Brazil, a Jurassic-Hauterivian (200–130 Ma) phase of slow basement cooling is constrained by some samples from the AO. This cooling period aligns with the subsidence of the intracontinental Afro-Brazilian basin. Detrital zircon U–Pb data from deposits within this basin points to the AO as the primary sediment source, suggesting significant erosion during that time, and that the AO represented a paleohigh relative to the subsiding Afro-Brazilian basin^32^. Contrary to the Paleozoic cooling, the Jurassic to Hauterivian phase seems to be much more localized but similarly, it was trigger by subsiding nearby depocenters.

The rifting and breakup of Gondwana in the Mesozoic and Cenozoic produced a spatially heterogeneous exhumation response across different terranes. While the AO (Brazil), AC (Colombia), and Eastern DC (India) experienced significant exhumation under extensional stresses, exhumation in the SFC (Brazil) and Western DC (India) was more localized, primarily near major fault zones. Similar to the East African Rift System, rift shoulder uplift and subsequent erosion played a key role, but our data indicate that this uplift was neither uniform nor laterally continuous. In the AO, proximity to the rigid SFC resulted in slower exhumation rates, highlighting the influence of rheological inheritance on uplift dynamics. Here, at the boundary with the craton, the orogen is sustained by the deformed São Francisco paleocontinental lithosphere, where the preserved lithospheric thickness and rigidity are key aspects of its rheological inheritance that limited strain accommodation within the AO^12^.

During the post-rift passive margin phase, our data reveal discrete episodes of basement exhumation within the AO and Eastern DC. In the AO, a late Cretaceous to Paleocene exhumation phase led to rapid basement cooling in the southeastern orogen, primarily controlled by major shear zones that run parallel to the coastline and extend northward. Similarly, in the Eastern DC, Cenozoic cooling is concentrated along principal shear zones, indicating a structurally controlled exhumation response. While the precise driving mechanisms of these post-rift exhumation events remain unresolved, they likely involved mantle dynamics, isostatic adjustment, and reactivation of inherited structures under far-field stresses facilitated by a thinned lithosphere at the margin^12,25^. Ultimately, the evidence underscores the fundamental role of structural inheritance in localizing and partitioning deformation during passive margin evolution.

### Reevaluating the Wilson Cycle in light of intraplate tectonics

The Wilson Cycle has remained a fundamental framework in plate tectonics theory since its formalization^33^, with minimal revision in the first four decades after its formulation. However, recently^34^ need for refinement has been underscored, particularly in light of new and emerging insights, including those presented here. These revisions do not diminish the significance of the Wilson Cycle as a conceptual model but rather seek to enhance its applicability by incorporating additional tectonic paths and processes.

**Fig. 4:**
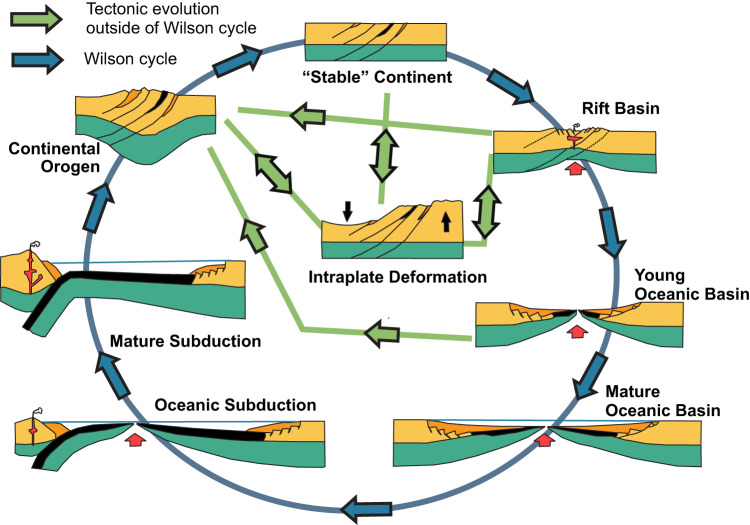
Adapted Wilson Cycle incorporating intraplate deformation and alternative tectonic evolution pathways (modified from^34^).

A first key consideration in refining the Wilson Cycle is the incorporation of intraplate tectonics as a distinct stage (Fig. 4). The existence of intraplate tectonics has long been recognized, and low-temperature thermochronology over the past decade has provided a robust method for dating and constraining deformation across diverse intraplate settings. This methodology has significantly deepened our understanding of the mechanisms governing intraplate deformation. It is now well established that stresses transmitted from plate boundaries exert a fundamental control on lithospheric vertical movements, driving processes such as foreland bulging^35^, basin inversion^36^, intracontinental orogeny^5^, and lithospheric folding^37,38^. These mechanisms, along with mantle dynamics, shape intraplate continental interiors^39^, highlighting that epeirogenic processes and tectonic reactivation are more widespread and complex than recognized in the Wilson Cycle model.

It was also suggested^8^ to integrate cratonic basins into the Wilson Cycle model. Building on this, we propose that intraplate deformation—whether through subsidence leading to basin formation or uplift causing erosion—should be recognized as an integral stage (Fig. 4). Our analysis of Gondwana terranes, for instance, demonstrates that while some intraplate areas were subsiding with active basin formation, others experienced uplift and subsequent erosion within the continental interior of Gondwana during the Paleozoic.

In addition, our results emphasize that intraplate deformation cannot be fully integrated into the Wilson Cycle without considering its controlling factors. These include the orientation of inherited structures relative to the stress field, the boundary conditions imposed by surrounding cratons, and the rheological/strength contrasts within the lithosphere. Together, these parameters dictate the efficiency, style, and localization of intraplate deformation, and help explain the contrasting exhumation histories observed across Gondwana terranes.

Another critical reconsideration of the Wilson Cycle is the underappreciated role of tectonic inheritance in intraplate deformation. While the Wilson Cycle traditionally assumes that renewed deformation occurs near pre-existing tectonic structures, this principle also applies to intraplate settings. The spatial distribution of AFT data presented here highlights the influence of inherited shear zones and fault systems in localizing deformation and driving differential exhumation in aforementioned contexts. These structures often facilitate more rapid and recent uplift, whereas proximity to rigid cratonic domains can, in some cases, suppress vertical motion, leading to more subdued exhumation magnitudes.

The assumption of cratons as uniformly stable, rigid blocks also requires further reevaluation. AFT data from the Eastern DC and AC illustrate their active role in intraplate settings, revealing significant tectonic reactivation and deep exhumation of Archean crystalline basement during the Mesozoic and Cenozoic. Rather than representing quiescent tectonic domains, their thermal histories closely parallel those of traditionally weaker regions, such as the AO. Notably, both the AO and Eastern DC experienced reactivation during syn- and post-rift phases, underscoring a shared susceptibility to deformation.

**Fig. 5:**
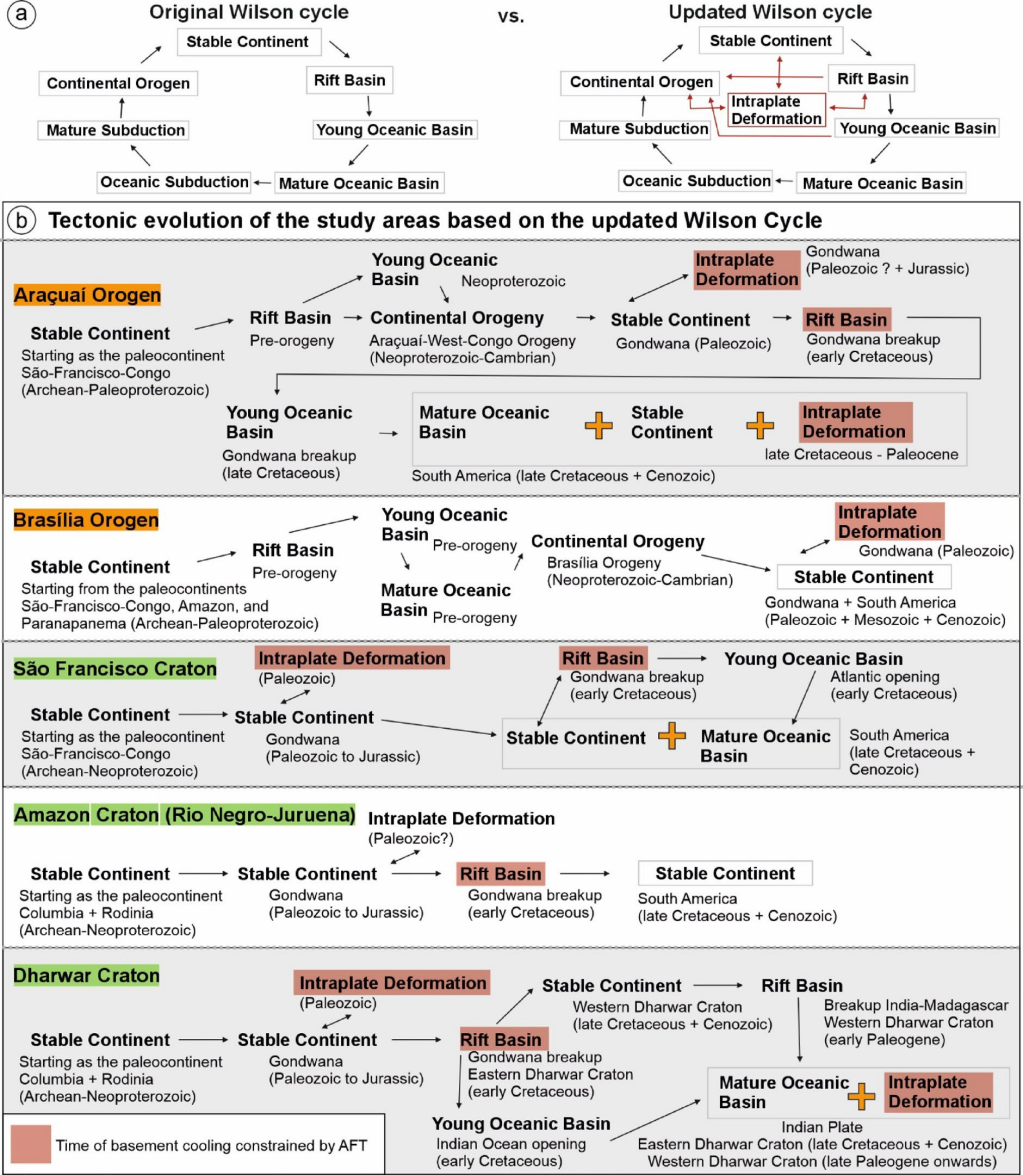
(**a**) Original versus updated Wilson Cycle including intraplate deformation. (**b**) Tectonic evolution of Gondwana terranes and orogens based on apatite fission-track constraints and the updated Wilson Cycle.

Phanerozoic tectonic records also indicate that cratons have not only accommodated intraplate deformation but have also actively participated in plate boundary processes. The evolution of the DC, AC, and SFC during Gondwana fragmentation exemplify this dynamic behavior, demonstrating that cratons can serve as loci of extension and breakup rather than merely acting as passive backstops (Fig. 5). These findings challenge the conventional view of cratons and other seemingly stable regions as tectonically inert. Even cratons that did not participate in major Phanerozoic orogenies remain potential loci for reactivation, extension, and, in some cases, breakup and orogenesis.

Finally, we propose a revision to the deterministic nature of the Wilson Cycle, which assumes a fixed sequence of ocean closure, orogeny, rifting, and renewed ocean opening at the same location (Fig. 5). A comparison of the evolution of the AO and BO demonstrates that alternative tectonic pathways must be incorporated into the model (Fig. 5). During the Mesozoic opening of the Atlantic, the AO did not rift along its ancient suture zones but instead split from its African counterpart within its crystalline core^18^. This deviation was likely influenced by factors beyond prior closure, including variations in lithospheric thickness, elevated thermal gradients in the magmatic arc, and topographic loading. Thus, the location of rifting was not solely determined by the site of the previous closure but also by additional lithospheric and thermomechanical factors.

The BO further complicates the deterministic paradigm, exhibiting resilience to Mesozoic rifting and oceanization despite its proximity to major magmatic zones, such as the Paraná LIP. Its Phanerozoic stability and craton-like exhumation patterns imply progressive lithospheric strengthening, suggestive of a “cratonization” process within mature orogenic belts. Although resistant to Mesozoic and Cenozoic tectonic stresses, Paleozoic records reveal a history of intraplate deformation, indicating that reactivation potential persists even in more resilient orogenic lithospheres. Comparative analysis of the AO and BO underscores divergent post-orogenic trajectories, calling for expanded tectonic models that accommodate orogenic stabilization pathways beyond the classical Wilson Cycle path (Fig. 4 and 5).

Several lithospheric inheritance factors may account for the diverging trajectories of the AO and BO^10^. The relative stiffness of these belts was likely influenced by (i) the size of the oceanic domain being closed, with the narrower closure of the AO favoring a hotter and weaker lithosphere, in contrast to the wider ocean involved in the BO; (ii) the presence of long-lived subduction and accretion of mafic material in the BO, producing a thicker and more refractory lithosphere; (iii) dehydration metamorphism during subduction, which further strengthened the BO lower crust; and (iv) contrasting mechanisms of post-orogenic relaxation. With respect to the latter, the AO underwent significant extensional collapse and magmatism that thermally softened its lithosphere, while the BO stabilized more rapidly. These factors help explain why the AO remained prone to Meso–Cenozoic reactivation and cooling, whereas the BO evolved toward craton-like rigidity.

Comparable patterns of intraplate reactivation have also been documented outside Gondwana, reinforcing the global relevance of our model. For instance, apatite fission-track data from the Petermann and Alice Springs orogens in central Australia record major Paleozoic cooling related to intraplate uplift along inherited shear zones^52^. Similar thermochronological evidence from the Laramide orogeny in North America demonstrates far-field stress transmission and basement-involved uplift during the Late Cretaceous–Paleogene^53^. These examples highlight that the contrasting trajectories of the AO and BO are not isolated cases but part of a broader class of non-deterministic intraplate orogenic responses.

In conclusion, the Wilson cycle is a helpful tool and essential for its concise depiction of lithospheric evolution. Its simplicity in conveying the fundamentals of plate tectonics makes it an invaluable resource in geology research and teaching. Revisiting and updating this concept helps bridge the gap between classical theories and modern geological knowledge. Recent revisions^34,40^ demonstrate its ongoing relevance. We propose adopting the modified scheme inspired by these revisions and additional insights obtained from our thermochronological data (Fig. 4). This adaptation more accurately reflects the diversity of tectonic processes occurring both at and far from plate margins, enhancing our understanding of continental lithosphere dynamics.

## Method

### Samples and study area

This study presents and discusses apatite fission-track (AFT) data from continental interiors and passive margins across ancient Gondwana terranes of Southeast (SE) Brazil, SE Colombia, and Peninsular India. A total of 135 samples were analyzed in the MinPet Laboratory at Ghent University to obtain AFT ages and reconstruct thermal histories (Supplementary data). These new results were integrated with previously published data from 270 samples (Supplementary data), resulting in a comprehensive dataset of 405 samples covering approximately 915,000 km^2^, of which ~ 700,000 km^2^ are in Brazil, 100,000 km^2^ in Colombia, and 115,000 km^2^ in India. This large-scale integration provides a regional framework for interpreting low-temperature thermochronological patterns across terrains with contrasting tectonic settings and lithospheric characteristics.

The sampling area includes the present-day passive margin of SE Brazil, from Rio de Janeiro to Salvador, and the western and eastern passive margins of southern India (south of 12°N), from Chennai to Mangalore. Inland coverage extends up to 1200 km into the Brazilian continental interior. In Colombia, samples were collected in the Guainía department near the tri-border region with Venezuela and Brazil, within a densely forested, remote area.

The AFT data were published^9–14^, and interpretations were presented in specific geological and local tectonic contexts. Here, we step back from localized interpretations to examine broader geodynamic implications and by integrating all individual data sets. Rather than focusing on individual exhumation events, this study emphasizes first-order patterns in basement cooling and intraplate deformation, with a focus on what these results reveal about the evolution of the continental lithosphere throughout the Phanerozoic and how such patterns suggest revisions to the Wilson Cycle framework.

### Low temperature thermochronology

Identifying and dating (in absolute/numerical terms) tectonism and exhumation of rocks in intraplate settings presents significant challenges. Deformation far from active plate margins, especially through processes involving rock uplift, may leave no clear material record such as contemporaneous volcanism, metamorphism, or mineralization. This is often the case in passive margin and continental interior settings, where most of the recent deformation transpired in a brittle regime by reactivation of pre-existing structural heterogeneities. Such reactivations frequently occur without or with limited crystallization of new minerals or formation of rocks that can be directly dated. As a result, traditional geochronological methods, are most of the time unable to determine the timing of intraplate tectonic processes.

In this context, low-temperature (low-T) thermochronology emerges as a crucial field of study capable of placing tectonism and rock exhumation within a temporal framework. Several such methods dedicated to deciphering the thermal history of the upper crust at temperatures below 350 °C over geological time exist^41,42^. These methods track the thermal evolution of minerals as they cool through specific temperature intervals, often driven by rock uplift and erosion.

Among the low-T thermochronology methods, fission-track, and in particular AFT analysis stands out as one of the most widely used and robust techniques. AFT offers insights into the time–temperature (t-T) path of a given rock since it has cooled below c. 120 °C^41^. Indeed, the temperature range in which the AFT method is sensitive aligns with temperatures prevalent in the upper crust (2–4 km), making it particularly suitable for studying the thermal history related to exhumation and near-surface processes.

### Apatite fission-track (AFT) analysis

A substantial portion of the AFT dataset presented in this study was generated over the past decade by the fission-track laboratory at Ghent University, using a consistent External Detector Method (EDM) procedure^41^ and standardized optical equipment.

Apatite grains were concentrated from the rocks by conventional crushing and magnetic and heavy liquid separation. Apatite grains were then hand-picked and mounted in Struers CaldoFix-2 epoxy resin, after which they were grinded on SiC paper and polished using diamond suspension (6 μm, 3 μm, and 1 μm). Apatite mounts from Brazil and Colombia were etched using protocol, 5.5 mol/l HNO_3_ for 20 s at 21 °C^43^, to reveal the spontaneous fission tracks. For the apatite samples from India, the etching was done several years ago, with a 2.5% HNO_3_ solution for 70 s at 25 °C ^44,45^ which was then the standard at the UGent FT lab (different zeta calibration factors were hence used to analyze the respective sample sets). The D_par_ values measured using the slow etching (India samples) needed to be converted in order to readily use the values in the inverse modelling software and annealing model^46^. For this conversion a standard approach was used^47^.

The etched samples were covered with a Goodfellow Clear Ruby muscovite sheet (ED) for irradiation alongside U-doped glass dosimeter mounts and apatite age standards (Durango and Fish Canyon Tuff) for calibration. The irradiation occurred in the X26 irradiation channel of reactor BR1, at the Belgian Nuclear Research Centre (SCK Mol, Belgium). Channel X26 is highly thermalized, with a thermal/epithermal fluence ratio of 98 ± 3 and a thermal fluence rate of 6.12 × 1010 n/cm^2^s. The integrated irradiation flux can reach up to 2.42 × 10^15^ n/cm^2^ for a 1.5-day period, with minimal axial and radial flux gradients, indicating effective and consistent thermal neutron distribution^48^. After irradiation, the ED’s were removed from their respective sample, standard or glass dosimeter mounts and etched in a 40% HF solution for 40 min at 21 °C in order to reveal the induced fission tracks. Apatite mounts were then affixed alongside their mica ED on a glass microscope slide for analysis.

We performed all length, angles to the crystallographic c-axis and D_par_ measurements using a Nikon Eclipse Ni-E upright motorized microscope, with a mounted Nikon DS-Ri2 camera using the Nikon–TRACKFlow analysis protocol^49^. Apatite grains were selected for analysis, taking care to exclude grains that either were tilted (i.e. c-axis not parallel to microscope observation plane), exhibited significant cracks, or visible inclusions. Track counting aimed for at least 1000 spontaneous tracks per sample. Tracks were counted within manually defined polygonal regions of interest (ROIs), avoiding, as mentioned, any dark, cracked areas. For AFT counting, a Nikon Eclipse Ni-E and Leica DFC 295 microscope was used (each optical set with its specific zeta calibration value for the analyst).

For samples analyzed with the Nikon Eclipse Ni-E microscope, coordinate transformation aligned sample mounts with ED images using homologous points from selected apatite grains and track clouds. Nikon–TRACKFlow^49^ handled image flipping and rotation for accurate alignment, and automatically measured ROIs to ensure consistent counting areas. For the Leica DFC 295 microscope, the ‘sandwich’ repositioning method^50^ was used, aligning mica and apatite as during irradiation. Images were captured by shifting focus between spontaneous and induced tracks. ROI selection, counting, and area measurement were done using Leica LAS software (version 3.0).

### Thermal history modeling

Thermal history modeling relies on mathematical formulations of thermochronometric data, here in particular of fission-track annealing over geological time to reconstruct the time–temperature (t–T) evolution of apatite-bearing samples. Several software tools have been developed for this purpose, allowing users to derive potential thermal histories from observed AFT datasets.

In this study, we employed QTQt^51^, a widely used freeware package based on Bayesian statistics. QTQt explores the probability space of thermal histories by generating a wide array of t–T paths and evaluating how well each aligns with the observed data. The program supports various annealing models and allows for the incorporation of kinetic parameters; we used D_par_^46^ as a kinetic proxy.

Inverse modeling in QTQt was carried out using a Markov Chain Monte Carlo (MCMC) search algorithm, with 10^5^ post-burn iterations per model run. The t–T prior space was conservatively defined for each sample as ± one central age for time and 70 ± 70 °C for temperature. Only present-day surface temperature was applied as an external constraint in the models. No additional geological constraints were imposed, due to the absence of reliable stratigraphic or structural benchmarks in several of the study regions. This approach ensures that model results are data-driven and minimizes the introduction of interpretative bias.

QTQt produces a suite of plausible models, each accompanied by 95% credible intervals. For each model, predicted AFT ages and mean track lengths are compared directly with the observed values to evaluate their fit. Among the model outputs, three are commonly assessed: the maximum likelihood (MaxLike) model, which best explains the data based on likelihood alone; the maximum posterior (MaxPost) model, which incorporates both data and prior information; and the expected thermal history (ExTH), which represents a weighted average of all accepted models. Most samples in this study yielded well-constrained thermal histories, with strong agreement among the MaxLike, MaxPost, and ExTH models (for modelling results, see Supplementary data). Representative ExTH models are presented in Fig. 3 to illustrate the dominant thermal patterns across the study regions.

## Supplementary Information

Below is the link to the electronic supplementary material.

Supplementary Material 1

## Data Availability

All data generated or analyzed during this study are included in this article and its supplementary information files. Additional files, including microscope images, track measurements, and calibration data, are available from the corresponding author upon reasonable request.

## References

[1] Cox, A. & Hart, R. B. *Plate Tectonics: How It Works* (Blackwell Scientific Publications, 1986).

[2] Wilson, J. T. Did the Atlantic close and then re-open?. *Nature* **211**, 676–681. 10.1038/211676a0 (1966).

[3] Audet, P. & Bürgmann, R. Dominant role of tectonic inheritance in supercontinent cycles. *Nat. Geosci.* **4**, 184–187. 10.1038/ngeo1080 (2011).

[4] Corti, G., van Wijk, J., Cloetingh, S. & Morley, C. K. Tectonic inheritance and continental rift architecture: numerical and analogue models of the East African Rift system. *Tectonics* **26**, TC6016. 10.1029/2006TC002086 (2007).

[5] Raimondo, T., Hand, M. & Collins, W. J. Compressional intracontinental orogens: ancient and modern perspectives. *Earth-Sci. Rev.* **130**, 128–153 (2014).

[6] De Grave, J., Buslov, M. M. & Van den Haute, P. Distant effects of India-Eurasia convergence and Mesozoic intracontinental deformation in Central Asia: constraints from apatite fission-track thermochronology. *J. Asian Earth Sci.* **29**, 188–204. 10.1016/j.jseaes.2006.03.001 (2007).

[7] Allen, P. A. & Armitage, J. J. Cratonic basins. In *Tectonics of Sedimentary Basins: Recent Advances* (eds Busby, C. & Azor, A.) 602–620 (Blackwell Publishing Ltd, 2012).

[8] Daly, M.C., Tozer, B. & Watts, A.B. Cratonic basins and the Wilson cycle: A perspective from the Parnaíba Basin, Brazil. Geol. Soc. Lond., Spec. Publ. vol. 470, pp. 463–477 (2019).

[9] Fonseca, A. C. et al. Devonian to Permian post-orogenic denudation of the Brasília Belt of West Gondwana: Insights from apatite fission track thermochronology. *J. Geodyn.*10.1016/j.jog.2020.101733 (2020).

[10] Fonseca, A. C. L. et al. Differential phanerozoic evolution of cratonic and non-cratonic lithosphere from a thermochronological perspective: São Francisco Craton and marginal orogens (Brazil). *Gondwana Res.* **93**, 106–126. 10.1016/j.gr.2021.01.006 (2021).

[11] Fonseca, A., Cruz, S., Novo, T., He, Z. & De Grave, J. Differential exhumation of cratonic and non-cratonic lithosphere revealed by apatite fission-track thermochronology along the edge of the São Francisco Craton, eastern Brazil. *Sci. Rep.* **12**, 1–9. 10.1038/s41598-022-06419-w (2022). 35177732 10.1038/s41598-022-06419-w PMC8854403

[12] Fonseca, A. et al. Control of inherited structural fabric on the development and exhumation of passive margins—insights from the Araçuaí Orogen (Brazil). *Geosci. Front.* **14**, 101628. 10.1016/j.gsf.2023.101628 (2023).

[13] Fonseca, A., Nachtergaele, S., Bonilla, A., Dewaele, S. & De Grave, J. Extensional exhumation of cratons: insights from the Early Cretaceous Rio Negro-Juruena belt (Amazonian Craton, Colombia). *Solid Earth* **15**, 329–352. 10.5194/se-15-329-2024 (2024).

[14] Fonseca, A., Glorie, S., He, Z., Singh, T. & De Grave, J. Contrasting thermal histories for the Indian passive margins during syn- and post-Gondwana break-up: Insights from apatite fission-track thermochronology. *Terra Nova* **34**, 543–553. 10.1111/ter.12621 (2022).

[15] Malusà, M. G. & Fitzgerald, P. G. (eds) *Fission-track thermochronology and its application to geology* (Springer, 2019).

[16] Alkmim, F. F., Kuchenbecker, M., Reis, H. L. S. & Pedrosa-Soares, A. C. The Araçuaí Belt. In (eds Heilbron, M., Cordani, U. G. & Alkmim, F. F.) São Francisco Craton, Eastern Brazil, 255–275 (Springer, 2017). 10.1007/978-3-319-01715-0

[17] Pimentel, M. M. The tectonic evolution of the Neoproterozoic Brasília Belt, central Brazil: A geochronological and isotopic approach. *Braz. J. Geol.* **46**, 67–82. 10.1590/2317-4889201620150004 (2016).

[18] Pedrosa-Soares, A.C. et al. Similarities and differences between the Brazilian and African counterparts of the Neoproterozoic Araçuaí–West Congo orogen. Geol. Soc. Lond., Spec. Publ., vol. 294, pp. 153–172 (2008). 10.1144/SP294.9

[19] Santos, J. O. S. et al. Age and autochthonous evolution of the Sunsás Orogen in West Amazon Craton based on mapping and U-Pb geochronology. *Precambrian Res.* **165**, 120–152. 10.1016/j.precamres.2008.06.009 (2008).

[20] Jayananda, M., Santosh, M. & Aadhiseshan, K. R. Formation of Archean (3600–2500 Ma) continental crust in the Dharwar Craton, southern India. *Earth-Sci. Rev.* **181**, 12–42. 10.1016/j.earscirev.2018.03.013 (2018).

[21] Bicca, M. M. et al. Tectonic evolution and provenance of the Santa Bárbara Group, Camaquã Mines region, Rio Grande do Sul, Brazil. *J. S. Am. Earth Sci.* **48**, 173–192. 10.1016/j.jsames.2013.09.006 (2013).

[22] Borba, A. W. et al. Significance of Late Paleozoic fission-track ages in volcanic rocks from the Lavras do Sul region, southernmost Brazil. *Gondwana Res.* **6**, 79–88. 10.1016/S1342-937X(05)70645-6 (2003).

[23] Doranti-Tiritan, C., Hackspacher, P. C., Ribeiro, M. C. S., Glasmacher, U. A. & Souza, D. H. Relief evolution of Poços de Caldas (SP/MG) region based on thermochronology data and 3D thermokinematic modeling. *Rev. Bras. Geomorfologia* **15**, 291–310. 10.20502/rbg.v15i2.491 (2014).

[24] Oliveira, C. H. E., Jelinek, A. R., Chemale, F. & Bernet, M. Evidence of post-Gondwana breakup in the southern Brazilian Shield: Insights from apatite and zircon fission-track thermochronology. *Tectonophysics* **666**, 173–187. 10.1016/j.tecto.2015.11.005 (2016).

[25] Jelinek, A. R. et al. Denudation history and landscape evolution of the northern East-Brazilian continental margin from apatite fission-track thermochronology. *J. S. Am. Earth Sci.* **54**, 158–181. 10.1016/j.jsames.2014.06.001 (2014).

[26] Kasanzu, C. H. et al. From source to sink in central Gondwana: Exhumation of the Precambrian basement rocks of Tanzania and sediment accumulation in the adjacent Congo Basin. *Tectonics* **35**, 2034–2051. 10.1002/2016TC004147 (2016).

[27] Krob, F. C. et al. Multi-chronometer thermochronological modelling of the late Neoproterozoic to recent t–T evolution of the SE coastal region of Brazil. *J. S. Am. Earth Sci.* **92**, 77–94. 10.1016/j.jsames.2019.02.012 (2019).

[28] Morón, S. et al. Denuding a craton: Thermochronology record of phanerozoic unroofing from the Pilbara Craton, Australia. *Tectonics* **39**, 1–30. 10.1029/2019TC005988 (2020).

[29] Nixon, A. L. et al. Intracontinental fault reactivation in high heat production areas of central Australia: Insights from apatite fission-track thermochronology. *Geochem. Geophys. Geosyst.*10.1029/2022GC010559 (2022).

[30] Milani, E.J. & De Wit, M.J. Correlations between the classic Paraná and Cape–Karoo sequences of South America and southern Africa and their basin infills flanking the Gondwanides: du Toit revisited. Geol. Soc. Lond., Spec. Publ., vol. 294, pp. 319–342 (2008). 10.1144/SP294.17

[31] Cloetingh, S. et al. Tectonic models for the evolution of sedimentary basins. Treatise Geophys. 2nd edn, 513–592 (2015).

[32] Kuchle, J. et al. A contribution to regional stratigraphic correlations of the Afro-Brazilian depression – the Dom João Stage (Brotas Group and equivalent units – Late Jurassic) in northeastern Brazilian sedimentary basins. *J. S. Am. Earth Sci.* **31**, 358–371. 10.1016/j.jsames.2011.02.007 (2011).

[33] Burke, K. & Dewey, J. F. The Wilson cycle. *Geol. Soc. Am. Abstr. Programs* **10**, 48 (1975).

[34] Wilson, R. W. et al. Fifty years of the Wilson Cycle concept in plate tectonics: an overview. Geol. Soc. Lond., Spec. Publ. 470, 1–17 (2019).

[35] Ziegler, P. A., Bertotti, G. & Cloetingh, S. Dynamic processes controlling foreland development: the role of mechanical (de)coupling of orogenic wedges and foreland. *EGU Stephan Mueller Spec. Publ. Ser.* **1**, 17–56 (2002).

[36] Ziegler, P. A., Van Wees, J. D. & Cloetingh, S. Mechanical controls on collision-related compressional intraplate deformation. *Tectonophysics* **300**, 103–129 (1998).

[37] Cloetingh, S. A. P. L., Burov, E. & Poliakov, A. Lithosphere folding: primary response to compression (from central Asia to Paris Basin). *Tectonics* **18**, 1064–1083 (1999).

[38] Cloetingh, S. Intraplate stresses: a new element in basin analysis. In New Perspectives in Basin Analysis, 205–230. Springer, New York (1988).

[39] Bertotti, G., Schulmann, K. & Cloetingh, S. A. P. L. (eds) Continental collision and the tectono-sedimentary evolution of forelands. EGU Stephan Mueller Spec. Publ. Ser. 1, 236 pp (2002).

[40] Heron, P. J., Pysklywec, R. N. & Stephenson, R. Identifying mantle lithosphere inheritance in controlling intraplate orogenesis. *J. Geophys. Res. Solid Earth* **121**, 6966–6987 (2016).

[41] Wagner, G. & Van den Haute, P. *Fission Track Dating* (Kluwer Academic Publishers, 1992). 10.1016/B0-44-452747-8/00052-1.

[42] Reiners, P. W. et al. *Geochronology and Thermochronology* (John Wiley & Sons, 2018).

[43] Donelick, R. A. Apatite etching characteristics versus chemical composition. *Nucl. Tracks Radiat. Meas.* **21**, 604 (1993).

[44] Glorie, S. et al. Tectonic history of the Irtysh shear zone (NE Kazakhstan): New constraints from zircon U/Pb dating, apatite fission-track dating and palaeostress analysis. *J. Asian Earth Sci.* **45**, 138–149. 10.1016/j.jseaes.2011.09.024 (2012).

[45] Jonckheere, R. & Ratschbacher, L. Standardless fission-track dating of the Durango apatite age standard. *Chem. Geol.* **417**, 44–57 (2015).

[46] Ketcham, R. A. et al. Improved measurement of fission-track annealing in apatite using c-axis projection. *Am. Min.* **92**, 789–798. 10.2138/am.2007.2280 (2007).

[47] Sobel, E. R. & Seward, D. Influence of etching conditions on apatite fission-track etch pit diameter. *Chem. Geol.* **271**, 59–69. 10.1016/j.chemgeo.2009.12.012 (2010).

[48] De Grave, J. et al. A new irradiation facility for FT applications at the Belgian Nuclear Research Centre: The BR1 reactor. In *International Conference on Thermochronology* (2010).

[49] Van Ranst, G., Baert, P., Fernandes, A. C. & De Grave, J. Technical note: Nikon–TRACKFlow; a new versatile microscope system for fission track analysis. *Geochronology* **2**, 93–99. 10.5194/gchron-2-93-2020 (2020).

[50] Jonckheere, R. On methodical problems in estimating geological temperature and time from measurements of fission tracks in apatite. *Radiat. Meas.* **36**, 43–55. 10.1016/S1350-4487(03)00096-9 (2003).

[51] Gallagher, K. Transdimensional inverse thermal history modelling for quantitative thermochronology. *J. Geophys. Res.* **117**, 1–16 (2012).

[52] Nixon, A. L. et al. Footprints of the Alice Springs Orogeny preserved in far northern Australia: An application of multi-kinetic thermochronology in the Pine Creek Orogen and Arnhem Province. *J. Geol. Soc.* **178**, 2020–2173. 10.1144/jgs2020-173 (2021).

[53] Stevens, A. L., Balgord, E. A. & Carrapa, B. Revised exhumation history of the Wind River Range, WY, and implications for Laramide tectonics. *Tectonics* **35**, 179–201. 10.1002/2016TC004126 (2016).

[54] Müller, R. D. et al. A global plate model including lithospheric deformation along major rifts and orogens since the Triassic. *Tectonics* **38**, 1884–1907. 10.1029/2018TC005462 (2019).

