# Supplementary material for: Intraplate deformation of Gondwana terranes and implications for the Wilson Cycle
Source: Sci Rep. 2025 Nov 21;15:41450. doi: 10.1038/s41598-025-27764-6 (PMC12639100; doi:10.1038/s41598-025-27764-6)
Supplement: Supplementary file 1 — Supplementary Material 1 [file 41598_2025_27764_MOESM1_ESM.pdf]

Supplementary data: Apatite Fission-Track data

SAMPLES ANALYZED AT UGENT (DATA AVAILABLE AT AUSGEOCHEM.AUSCOPE.ORG.AU)

Table A.1: Sample localities (in World Geodetic System – WGS84), Z: elevation above sea level, and lithology. Analysts: Ana Fonseca (AF); TN (Tiago Novo); Gabriella Piffer (GP); Johan De Grave (JDG); and Simon Nachtergaele (SN).

	Sample	X (°)	Y (°)	Z (m)	Lithology	Analyst	Reference
Samples from Brazil	S1	-42.2768	-20.7010	718	Banded granulite gneiss	AF	Fonseca et al. 2021
	S2	-42.4713	-20.7285	1192	Medium grained pyroxene granite	AF	Fonseca et al. 2021
	S3	-42.6509	-20.6900	651	Medium grained pyroxene granite	AF	Fonseca et al. 2021
	S4	-42.6844	-20.6869	614	Biotite gneiss	AF	Fonseca et al. 2021
	S5	-42.8959	-20.7306	619	Medium grained biotite gneiss	AF	Fonseca et al. 2021
	S6	-43.1099	-20.6665	615	Fine grained biotite gneiss	AF	Fonseca et al. 2021
	S7	-43.3320	-20.6757	663	Medium grained biotite gneiss	AF	Fonseca et al. 2021
	S8	-43.5802	-20.6839	722	Fine grained biotite gneiss	AF	Fonseca et al. 2021
	S9	-43.6389	-20.6725	738	Fine grained biotite gneiss	AF	Fonseca et al. 2021
	S10	-43.8328	-20.5669	926	Coarse grained biotite granite	AF	Fonseca et al. 2021
	S12	-44.1654	-20.9957	1084	Medium grained biotite granite	AF	Fonseca et al. 2021
	C2	-43.3416	-19.7112	720	Coarse grained biotite gneiss	AF	Fonseca et al. 2021
	C3	-43.2028	-19.6612	735	Biotite granite	AF	Fonseca et al. 2021
	C4	-43.0367	-19.7237	558	Coarse grained biotite gneiss	AF	Fonseca et al. 2021
	C6	-42.7839	-19.6174	262	Folded and sheared biotite gneiss	AF	Fonseca et al. 2021
	C7	-42.6943	-19.2180	242	Biotite gneiss	AF	Fonseca et al. 2021
	C8	-42.3276	-19.2300	197	Garnet bearing amphibolite	AF	Fonseca et al. 2021
	C9	-42.2509	-19.1723	208	Fine grained biotite gneiss	AF	Fonseca et al. 2021
	C11	-42.0818	-18.9656	177	Fine grained biotite gneiss	AF	Fonseca et al. 2021
	C12	-42.0225	-18.9231	188	Fine grained biotite gneiss	AF	Fonseca et al. 2021
	N3	-42.2801	-16.7072	557	Tourmaline bearing pegmatite	AF	Fonseca et al. 2021
	N4	-42.1826	-16.6299	310	Biotite granite	AF	Fonseca et al. 2021
	N5	-42.2846	-16.2219	445	Metagreywacke	AF	Fonseca et al. 2021
	N7	-42.9033	-16.2274	832	Gneiss	AF	Fonseca et al. 2021
	S11	-44.0412	-20.6574	878	Fine grained biotite gneiss	AF	Fonseca et al. 2021
	S13	-44.4227	-20.8681	992	Medium grained biotite granite	AF	Fonseca et al. 2021
	S14	-44.7343	-21.0270	997	Porphyritic basalt	AF	Fonseca et al. 2021
	1	-45.1248	-21.1736	786	Migmatitic hornblende gneiss	AF	Fonseca et al. 2021
	C1	-43.7991	-19.8133	855	Migmatitic biotite gneiss	AF	Fonseca et al. 2021
	C22	-43.9794	-19.8901	859	Fine grained biotite gneiss	AF	Fonseca et al. 2021
	C19	-44.1580	-19.9635	851	Biotite gneiss	AF	Fonseca et al. 2021
	C20	-44.6217	-19.8897	850	Biotite granite	AF	Fonseca et al. 2021

Sample	X (°)	Y (°)	Z (m)	Lithology	Analyst	Reference
C21	-44.8803	-19.8934	682	Fine grained biotite gneiss	AF	Fonseca et al. 2021
C18	-45.3290	-19.7837	680	Biotite bearing migmatite	AF	Fonseca et al. 2021
N11	-44.7121	-15.3076	620	Granite	AF	Fonseca et al. 2021
N10	-44.6414	-15.3559	602	Granite	AF	Fonseca et al. 2021
4	-45.5288	-21.1933	805	Medium grained hornblende granite	AF	Fonseca et al. 2021
5	-45.7551	-21.2543	893	Biotite gneiss	AF	Fonseca et al. 2021
6	-46.0035	-21.4468	793	Coarse grained pyroxene gneiss	AF	Fonseca et al. 2021
7	-46.3717	-21.3339	883	Coarse grained pyroxene gneiss	AF	Fonseca et al. 2021
8	-46.5302	-21.3558	972	Coarse grained pyroxene gneiss	TN	Fonseca et al. 2020
9	-46.8217	-21.2976	777	Coarse grained pyroxene gneiss	GP	Fonseca et al. 2020
10	-46.9280	-21.3034	791	Granite	GP	Fonseca et al. 2020
C17	-46.8887	-18.8789	1061	Metaconglomerate	GP	Fonseca et al. 2020
C16	-47.2202	-18.9419	884	Biotite granite	GP	Fonseca et al. 2020
C15	-47.7057	-18.7589	856	Biotite granite	GP	Fonseca et al. 2020
C14	-48.0631	-18.4268	577	Biotite bearing migmatite	GP	Fonseca et al. 2020
N12	-48.2356	-16.9667	898	Garnet bearing gneiss	GP	Fonseca et al. 2020
N13	-48.5663	-17.2695	758	Mylonitic granite	GP	Fonseca et al. 2020
N16	-49.3007	-17.2581	631	Mylonitic granite	GP	Fonseca et al. 2020
TJ 02	-15.7176	-43.0938	583	Gneiss	AF	Fonseca et al., 2022a
TJ 03	-15.7293	-43.0192	598	Granite	AF	Fonseca et al., 2022a
TJ 04	-15.6480	-42.9518	524	Granite	AF	Fonseca et al., 2022a
TJ 05	-15.5306	-42.9195	589	Gneiss	AF	Fonseca et al., 2022a
TJ 06	-15.4044	-42.8690	563	Gneiss	AF	Fonseca et al., 2022a
TJ 07	-15.2799	-42.8929	536	Gneiss	AF	Fonseca et al., 2022a
TJ 08	-15.2058	-42.8859	578	Gneiss	AF	Fonseca et al., 2022a
TJ 09	-15.1282	-42.8782	645	Gneiss	AF	Fonseca et al., 2022a
TJ 10	-15.0288	-42.9024	612	Gneiss	AF	Fonseca et al., 2022a
TJ 11	-14.9223	-42.8181	570	Gneiss	AF	Fonseca et al., 2022a
TJ 12	-14.8557	-42.7556	570	Granite	AF	Fonseca et al., 2022a
TJ 13	-14.7631	-42.6609	684	Filonite	AF	Fonseca et al., 2022a
TJ 14	-14.7226	-42.6589	651	Granite	AF	Fonseca et al., 2022a
TJ 15	-14.5580	-42.7015	612	Granite	AF	Fonseca et al., 2022a
TJ 16	-14.4564	-42.7112	583	Gneiss	AF	Fonseca et al., 2022a
TJ 17	-14.3723	-42.7756	562	Migmatite	AF	Fonseca et al., 2022a
TJ 18	-14.2777	-42.7839	520	Granite	AF	Fonseca et al., 2022a
TJ 19	-14.1933	-42.7989	515	Granite	AF	Fonseca et al., 2022a
TJ 20	-14.1316	-42.8511	521	Granite	AF	Fonseca et al., 2022a
TJ 21	-14.0331	-42.7132	546	Granite	AF	Fonseca et al., 2022a
TJ 22	-13.8732	-42.8608	538	Gneiss	AF	Fonseca et al., 2022a

Sample	X (°)	Y (°)	Z (m)	Lithology	Analyst	Reference
TJ 23	-13.8162	-42.9137	543	Gneiss	AF	Fonseca et al., 2022a
TJ 24	-13.7204	-42.9133	572	Gneiss	AF	Fonseca et al., 2022a
TJ 25	-13.6337	-42.9129	634	Granite	AF	Fonseca et al., 2022a
TJ 26	-13.7463	-42.7732	816	Gneiss	AF	Fonseca et al., 2022a
TJ 28	-14.2947	-42.2507	620	Mylonite	AF	Fonseca et al., 2022a
TJ 29	-14.4735	-42.2246	586	Granite	AF	Fonseca et al., 2022a
TJ 30	-14.9048	-41.9593	651	Gneiss	AF	Fonseca et al., 2022a
TJ 31	-14.9409	-41.6027	540	Mylonite	AF	Fonseca et al., 2022a
TJ 32	-14.9389	-41.5109	536	Mylonite	AF	Fonseca et al., 2022a
TJ 33	-14.9665	-41.4084	515	Gneiss	AF	Fonseca et al., 2022a
TJ 34	-15.0034	-41.2983	635	Mylonite	AF	Fonseca et al., 2022a
TJ 37	-15.8828	-41.4217	769	Granite	AF	Fonseca et al., 2023
TJ 39	-16.0485	-41.7754	720	Granite	AF	Fonseca et al., 2023
TJ 41	-16.2505	-41.4712	606	Granite	AF	Fonseca et al., 2023
TJ 42	-16.3589	-41.4707	555	Granite	AF	Fonseca et al., 2023
TJ 43	-16.4389	-41.4985	347	Granite	AF	Fonseca et al., 2023
TJ 44	-16.5755	-41.5045	262	Granite	AF	Fonseca et al., 2023
TJ 45	-16.6662	-41.5023	325	Granite	AF	Fonseca et al., 2023
TJ 46	-16.8172	-41.4706	500	Granite	AF	Fonseca et al., 2023
TJ 47	-16.9107	-41.4706	555	Granite	AF	Fonseca et al., 2023
TJ 48	-17.0002	-41.4469	614	Granite	AF	Fonseca et al., 2023
TJ 50	-17.2452	-41.5017	693	Granite	AF	Fonseca et al., 2023
TJ 53	-17.4293	-41.8089	830	Granite	AF	Fonseca et al., 2023
TJ 54	-17.5111	-41.8899	830	Granite	AF	Fonseca et al., 2023
TJ 55	-17.8936	-42.3801	660	Gneiss	AF	Fonseca et al., 2023
TJ 56	-17.9806	-42.3902	475	Gneiss	AF	Fonseca et al., 2023
TJ 57	-18.1048	-42.4122	567	Gneiss	AF	Fonseca et al., 2023
TA 05	-16.7253	-41.8992	281	Schist	AF	Fonseca et al., 2023
TA 07	-16.6107	-41.7562	299	Granite	AF	Fonseca et al., 2023
TA 08	-16.5795	-41.6126	284	Granite	AF	Fonseca et al., 2023
TA 13	-16.4232	-40.9552	220	Granite	AF	Fonseca et al., 2023
AL 03	-16.4262	-41.0116	212	Migmatite	AF	Fonseca et al., 2023
AL 04	-16.5842	-41.7380	270	Granite	AF	Fonseca et al., 2023
AL 05	-16.8616	-41.9465	570	Granite	AF	Fonseca et al., 2023
AL 06	-16.8751	-41.9409	780	Ignimbrite	AF	Fonseca et al., 2023
AL 07	-17.9771	-43.1204	754	Granite	AF	Fonseca et al., 2023
AL 08	-18.6652	-43.0731	730	Gneiss	AF	Fonseca et al., 2023
AL 11	-16.0804	-42.2952	617	Metaconglomerate	AF	Fonseca et al., 2023
AL 12	-15.8382	-43.0630	650	Granite	AF	Fonseca et al., 2023

Group	Sample	X (°)	Y (°)	Z (m)	Lithology	Analyst	Reference
	AL 13	-15.0631	-42.8019	703	Gneiss	AF	Fonseca et al., 2023
Samples from India	TN-04	12.9474	79.2075	203	Charnockite	JDG	Fonseca et al., 2022b
	TN-06	12.8969	78.9687	299	Granulite	JDG	Fonseca et al., 2022b
	TN-07	12.8970	78.9687	290	Granite (dyke)	JDG	Fonseca et al., 2022b
	TN-10	12.5986	78.6259	1030	Syenite	JDG	Fonseca et al., 2022b
	TN-11	12.6015	78.6208	825	Syenogranite	JDG	Fonseca et al., 2022b
	TN-12	12.5953	78.6092	623	Syenogranite	JDG	Fonseca et al., 2022b
	TN-13	12.5926	78.6060	454	Syenogranite	JDG	Fonseca et al., 2022b
	TN-14	11.9582	78.4587	445	Granite-gneiss	JDG	Fonseca et al., 2022b
	TN-16	11.9182	78.3903	445	Charnockite	JDG	Fonseca et al., 2022b
	TN-17	11.6187	78.1500	384	Gneiss	JDG	Fonseca et al., 2022b
	TN-18	11.6034	78.1419	299	Charnockite	JDG	Fonseca et al., 2022b
	TN-19	11.7750	78.1915	1345	Charnockite	JDG	Fonseca et al., 2022b
	TN-20	11.7717	78.1888	1039	Garnet migmatite	JDG	Fonseca et al., 2022b
	TN-21	11.7701	78.1869	856	Garnet gneiss	JDG	Fonseca et al., 2022b
	TN-22	11.7396	78.1767	550	Granite-gneiss	JDG	Fonseca et al., 2022b
	TN-23	11.7203	78.1747	383	Granite-gneiss	JDG	Fonseca et al., 2022b
	TN-24	11.5006	77.8913	332	Granite	JDG	Fonseca et al., 2022b
	TN-25	11.5046	77.8919	386	Granite	JDG	Fonseca et al., 2022b
	TN-26	11.4015	77.8837	300	Granite	JDG	Fonseca et al., 2022b
	TN-27	11.2198	78.1668	223	Granite-gneiss	JDG	Fonseca et al., 2022b
	TN-28	10.8230	78.6923	108	Granite	JDG	Fonseca et al., 2022b
	TN-29	11.1380	78.8231	150	(Leuco)granite	JDG	Fonseca et al., 2022b
	TN-30	11.2735	78.8897	153	(Grano)diorite	JDG	Fonseca et al., 2022b
	TN-31	12.2527	79.4429	110	Charnockite	JDG	Fonseca et al., 2022b
	TN-32	12.2524	79.4033	111	Granite	JDG	Fonseca et al., 2022b
	TN-33	12.2525	79.3876	98	Gneiss-migmatite	JDG	Fonseca et al., 2022b
	TN-34	12.2234	79.0508	210	Granite	JDG	Fonseca et al., 2022b
	TN-35	12.4858	79.1200	179	Granite-gneiss	JDG	Fonseca et al., 2022b
	TN-36	12.4678	79.3833	175	Charnockite	JDG	Fonseca et al., 2022b
	TN-37	12.5255	79.5859	109	Granite	JDG	Fonseca et al., 2022b
	TN-38	12.6079	80.0543	45	Charnockite	JDG	Fonseca et al., 2022b
Samples from Colombia	124	1.778028	68.91757	154	Porphyritic syenogranite	SN	Fonseca et al., 2024
	134	1.959167	68.36461	135	Monzogranite	SN	Fonseca et al., 2024
	135	1.834278	68.70745	147	Quartz monzodiorite	SN	Fonseca et al., 2024
	164	2.718608	67.948862	98	Monzogranite	SN	Fonseca et al., 2024
	168	2.79812	67.834094	97	Monzogranite	SN	Fonseca et al., 2024
	170	2.827199	67.799111	111	Monzogranite	SN	Fonseca et al., 2024
	171	2.728289	67.567755	88	Migmatite	SN	Fonseca et al., 2024

Sample	X (°)	Y (°)	Z (m)	Lithology	Analyst	Reference
175	2.589041	67.420448	84	Leucosome	SN	Fonseca et al., 2024
176	2.529057	67.365048	90	Migmatite	SN	Fonseca et al., 2024
180	2.166866	67.181351	86	Migmatite	SN	Fonseca et al., 2024
182	1.914885	67.066977	81	Monzogranite	SN	Fonseca et al., 2024
501	2.792967	69.281958	133	Syenogranite	SN	Fonseca et al., 2024
502	2.893865	69.061838	128	Monzogranite	SN	Fonseca et al., 2024
505	2.967441	68.714423	112	Quartzsyenite	SN	Fonseca et al., 2024
506	2.970117	68.667985	107	Quartzsyenite	SN	Fonseca et al., 2024
510	3.056191	68.506132	107	Monzogranite	SN	Fonseca et al., 2024
512	3.208222	68.21803	95	Monzogranite	SN	Fonseca et al., 2024
515	3.407566	67.973478	86	Monzogranite	SN	Fonseca et al., 2024
516	3.458146	67.981013	93	Monzogranite	SN	Fonseca et al., 2024

6

Table A.2: AFT age and length data: n is the number of counted grains; ρ_s, ρ_i, and ρ_d represent the density of spontaneous, induced tracks and induced tracks in an external detector (ED) irradiated against dosimeter glass respectively (tracks/cm^2). The ρd values are interpolated from data from regularly spaced glass dosimeters in a single irradiation container. N_s, N_i, and N_d are the number of counted spontaneous, induced tracks and induced tracks in the ED irradiated against the dosimeter glass. N_d is an interpolated value (as for ρ_d). $P(\chi^2)$ is the chi-squared probability that the dated grains have a constant ρ_s/ρ_i ratio (in %). D_{par} (in µm) was determined as the mean of 100 measurements of the track etch pit diameter parallel to the crystallographic c-axis. ζ-values (a/cm^2) were used for the calculation of the apatite FT central age (in Ma) based on Durango and Fish Canyon Tuff age standards and the IRMM-540 dosimeter glass. AFT length data are reported as raw mean track length (MTL in µm) with standard deviation σ obtained from the measurement of a number (nc) of natural, horizontal confined tracks.

	Sample	n	ρ_d	N_d	ρ_s	N_s	ρ_i	N_i	D_{par}	$P(\chi^2)$	FT Central Age	1 s.e. (AFT age)	MTL [µm]	nc	σ (MTL)	ζ	2 s.e. (ζ)
Samples from Brazil	S1	20	577400	5999	8051000	1200	5774000	893	1.79	25	98	6	13.5	81	1.5	253.6	5.5
	S2	11	482600	2375	3718000	350	4826000	327	1.69	61	81	7				313.9	5.8
	S3	25	579534	6054	8512000	1789	5716000	1354	1.99	43	97	5	13.5	87	1.7	253.6	5.5
	S4	9	448400	2255	5691000	249	4484000	261		4	61	6				286.2	4.7
	S5	25	475048	2375	5464000	1339	4750000	1105	1.37	18	90	5				313.9	5.8
	S6	22	473576	2368	2903000	524	4736000	471		98	82	6				313.9	5.8
	S7	20	472200	2361	27520000	2963	4722000	1606	1.95	44	135	6				313.9	5.8
	S8	18	470800	2354	3541000	281	4708000	144		97	143	8				313.9	5.8
	S9	18	469500	2347	22570000	1052	4695000	470	1.79	72	163	10				313.9	5.8
	S10	20	468000	2340	34750000	2434	4680000	836	1.85	37	210	10				326.5	5.7
	S12	20	465000	2325	7527000	616	4650000	280		12	158	14				313.9	5.8
	C2	22	485500	2427	8757000	1238	4855000	831	1.74	57	113	6	12.2	54	1.6	313.9	5.8
	C3	16	447500	2241	4164000	243	4475000	189		0	82	14				286.2	4.7
	C4	20	473200	2366	5980000	1196	4732000	1020	1.74	29	86	4				313.9	5.8
	C6	21	472300	2365	14370000	2976	4723000	2736	2.01	6	81	4	12.8	88	1.6	313.9	5.8
	C7	21	384100	1920	8967000	1888	3841000	2154		17	53	2				313.9	5.8
	C8	8	472700	2363	6841000	440	4727000	165	1.71	44	195	19	12.2	29	1.5	313.9	5.8
	C9	20	472400	2362	3508000	661	4724000	709	1.68	71	69	4				313.9	5.8
	C11	20	472200	2361	3872000	730	4722000	905	1.83	23	59	4	13.1	42	1.3	313.9	5.8

Samples from Brazil

Sample	n	ρ_d	N_d	ρ_s	N_s	ρ_i	N_i	D_{par}	$P(\chi^2)$	FT Central Age	1 s.e. (AFT age)	MTL [µm]	nc	σ (MTL)	ζ	2 s.e. (ζ)
C12	20	471900	2360	3592000	689	4719000	762	2.04	36	67	4				313.9	5.8
N3	7	468400	2342	31480000	1192	4684000	642	1.95	25	135	8	11.5	69	1.8	313.9	5.8
N4	23	468100	2341	3906000	687	4681000	469	1.84	9	107	8				313.9	5.8
N5	23	467900	2340	12700000	2214	4679000	1093	1.78	21	147	7	11.5	50	1.9	313.9	5.8
N7	2	466400	2332	7016000	52	4664000	24		32	157	40				313.9	5.8
S11	25	579529	6126	12780000	2231	5800000	696	1.66	43	233	14	12.6	56	1.9	253.6	5.5
S13	21	579527	6148	18890000	2500	5795000	728	1.75	12	249	19	12.7	91	1.3	253.6	5.5
S14	20	451400	2301	7123000	2018	4514000	291		77	160	12				286.2	4.7
1	23	495400	2477	26050000	2908	4954000	860	2.04	22	257	13				313.9	5.8
C1	25	487000	2435	10170000	1615	4870000	719	1.36	53	169	9				313.9	5.8
C22	20	468700	2343	30200000	3191	4687000	999	1.90	26	231	11				313.9	5.8
C19	22	469400	2347	21490000	3881	4694000	969	1.86	34	289	13	11.4	100	1.8	313.9	5.8
C20	20	469100	2346	25760000	3467	4691000	785	1.98	9	316	17				313.9	5.8
C21	22	468900	2344	17470000	3310	4689000	904	1.89	29	262	13				313.9	5.8
C18	22	469600	2348	28590000	6220	4696000	1527	1.98	7	296	13	11.2	100	1.4	313.9	5.8
N11	21	465600	2328	35810000	4083	4656000	844	2.02	59	344	16	11.9	87	1.6	313.9	5.8
N10	20	465900	2329	20380000	2455	4659000	614	1.71	14	284	17	11.7	100	1.3	313.9	5.8
4	21	494100	2470	25330000	2291	4941000	726	2.05	86	240	12				313.9	5.8
5	20	446500	2225	12150000	959	4465000	293		62	206	15				286.2	4.7
6	20	492600	2463	9860000	2167	4926000	702	1.67	9	234	14				313.9	5.8
7	21	491100	2456	12700000	1282	4911000	291	1.69	81	331	23				313.9	5.8
8	25	581800	5957	15480000	2524	5818000	542	1.50	20	337	21	12.2	80	1.3	254.6	8.8
9	20	580700	5967	5780000	1156	5807000	214	1.70	91	386	31	13.5	100	1.2	253.6	5.5
10	20	453300	2331	35990000	2241	4533000	543		59	261	19				313.9	5.8
C17	7	576900	2955	14700000	340	5769000	76		24	314	41				249.1	5.5
C16	20	576600	2954	28350000	3302	5766000	729	1.91	36	318	17	12.0	105	1.2	249.1	5.5
C15	3	576600	2954	28290000	299	5766000	78		25	269	37				249.1	5.5
C14	20	576400	2953	23620000	2491	5764000	579	1.95	48	302	16	12.1	105	1.2	249.1	5.5
N12	9	465400	2327	4486000	138	4654000	95	1.79	48	106	15				313.9	5.8

Samples from Brazil

Sample	n	ρ_d	N_d	ρ_s	N_s	ρ_i	N_i	D_{par}	$P(\chi^2)$	FT Central Age	1 s.e. (AFT age)	MTL [μm]	nc	σ (MTL)	ζ	2 s.e. (ζ)
N13	20	582700	2985	30050000	2024	5827000	592	2.07	50	243	13	11.4	100	1.8	313.9	5.8
N16	23	464600	2323	11600000	1423	4646000	387	1.73	23	263	17	10.8	24	1.9	313.9	5.8
TJ 02	20	405900	2048	10961000	1213	3740000	427	1.45	76	188	24	11.3	99	1.5	330.6	3.9
TJ 03	20	405500	2045	14283000	1358	5110000	479	1.78	87	187	11	11.0	100	1.8	330.6	3.9
TJ 04	20	405000	2043	12044000	1260	5420000	566	1.85	97	147	8	10.9	100	1.8	330.6	3.9
TJ 05	20	404400	2040	11267000	1072	3963000	369	2.61	97	191	13	11.7	100	1.6	330.6	3.9
TJ 06	20	404000	2037	13567000	1294	5212000	502	2.93	97	170	10	11.9	100	1.9	330.6	3.9
TJ 07	20	403500	2034	11717000	1335	3207000	338	2.30	94	258	17	11.3	65	1.6	330.6	3.9
TJ 08	20	403000	2032	11952000	960	4549000	370	1.73	88	171	11				330.6	3.9
TJ 09	20	400600	2018	13463000	1540	3402000	378	2.29	47	264	17	10.3	50	1.8	330.6	3.9
TJ 10	20	400100	2016	3529000	442	1288000	159	1.94	67	182	18				330.6	3.9
TJ 11	20	400000	2013	7152000	1027	4066000	587	1.65	32	115	7	9.9	24	1.3	330.6	3.9
TJ 12	20	399200	2010	18428000	2180	8167000	971	1.88	56	146	14	10.9	100	2.1	330.6	3.9
TJ 13	20	398700	2008	8082000	967	3076000	368	2.25	42	170	12	10.7	80	2.2	330.6	3.9
TJ 14	20	398200	2005	6919000	987	4469000	643	1.83	12	102	14	11.5	65	1.4	330.6	3.9
TJ 15	20	397700	2002	17514000	1662	9395000	886	1.82	85	122	6	9.7	98	2.4	330.6	3.9
TJ 16	20	397200	2000	31780000	1920	17523000	1056	1.80	83	118	5	11.9	100	1.7	330.6	3.9
TJ 17	20	395300	1989	33574000	936	13544000	2379	1.81	67	164	8	11.1	100	1.5	330.6	3.9
TJ 18	20	394900	1986	24118000	1687	9297000	638	2.25	32	170	20	11.7	100	1.6	330.6	3.9
TJ 19	20	394400	1984	8228000	1028	3334000	421	1.59	77	157	10				330.6	3.9
TJ 20	20	393900	1981	25679000	1445	9475000	533	1.72	16	175	23	11.2	100	1.6	330.6	3.9
TJ 21	20	393400	1978	11472000	962	3540000	296	1.68	86	208	15	12.1	100	1.7	330.6	3.9
TJ 22	20	393000	1976	11461000	1170	2543000	262	1.85	95	284	21	12.1	56	1.6	330.6	3.9
TJ 23	20	392500	1973	27629000	1619	7644000	455	2.29	88	227	13	12.8	100	1.2	330.6	3.9
TJ 24	20	392000	1970	6864000	828	1897000	238	1.79	76	222	17	11.7	53	1.6	330.6	3.9
TJ 25	20	504400	2503	25076000	2714	5181000	559	2.46	65	392	21	12.3	100	1.4	330.6	3.9
TJ 26	21	503100	2496	8387000	878	2092000	213	1.94	83	334	27	11.5	100	1.5	330.6	3.9
TJ 28	26	500400	2483	34889000	2218	16799000	1068	1.57	16	170	8	11.0	28	1.2	330.6	3.9
TJ 29	20	499100	2477	28712000	2553	15686000	1395	1.62	26	149	6	12.0	100	1.7	330.6	3.9

Samples from Brazil

Sample	n	ρ_d	N_d	ρ_s	N_s	ρ_i	N_i	D_{par}	$P(\chi^2)$	FT Central Age	1 s.e. (AFT age)	MTL [μm]	nc	σ (MTL)	ζ	2 s.e. (ζ)
TJ 30	27	497800	2471	2657000	435	1559000	251	1.60	100	141	12				330.6	3.9
TJ 31	23	496400	2464	35044000	2942	24592000	2068	1.32	24	116	9	10.8	100	1.2	330.6	3.9
TJ 32	20	495100	2457	6625000	771	3380000	395	1.52	64	158	11				330.6	3.9
TJ 33	12	488800	2427	1167000	74	628000	42		100	141	27				330.6	3.9
TJ 34	20	487600	2421	3981000	502	2670000	346	1.69	90	116	9				330.6	3.9
TJ 37	20	486280	2415	3149747	3221	486280	1986	1.96	36	128	5	12.2	100	1.7	327.8	3.4
TJ 39	20	484920	2408	750958	825	484920	576	1.56	44	113	7	12.7	100	1.8	327.8	3.4
TJ 41	20	483640	2402	895804	1024	483640	845	1.50	34	95	5				327.8	3.4
TJ 42	20	482280	2395	677625	823	482280	667	1.28	76	97	6	13.0	100	1.7	327.8	3.4
TJ 43	20	480920	2389	497015	480	480920	488	1.27	36	78	6	12.2	52	1.5	327.8	3.4
TJ 44	20	479640	2382	852906	794	479640	953	1.98	23	82	5				327.8	3.4
TJ 45	20	492200	2475	834926	955	492200	700	1.70	55	109	6	12.6	100	2.0	327.8	3.4
TJ 46	20	491080	2470	236462	393	491080	346	1.52	98	91	7	12.9	100	1.7	327.8	3.4
TJ 47	20	489960	2464	1661041	2026	489960	1177	1.30	12	137	7	12.3	100	1.9	327.8	3.4
TJ 48	20	418160	2102	827613	831	418160	562	1.26	46	101	7				327.8	3.4
TJ 50	20	418820	2104	1833405	2134	418820	1298	1.50	20	112	5	12.6	100	1.8	327.8	3.4
TJ 53	20	419140	2105	1987246	2259	419140	1091	1.53	12	141	7	11.4	100	1.7	327.8	3.4
TJ 54	20	419460	2106	1337620	1441	419460	934	1.84	62	105	5	11.4	100	2.0	327.8	3.4
TJ 55	20	419780	2107	752505	802	419780	491	1.86	66	111	7	12.4	100	1.8	327.8	3.4
TJ 56	20	420120	2109	1107268	1597	420120	1106	1.78	38	99	5	12.0	100	1.8	327.8	3.4
TJ 57	20	420460	2110	576369	961	420460	704	1.47	14	96	6	12.4	100	1.8	327.8	3.4
TA 05	20	420800	2111	866184	1121	420800	637	1.33	19	120	7				327.8	3.4
TA 07	20	421120	2113	2558161	2161	421120	1415	1.37	22	105	5	12.1	100	1.8	327.8	3.4
TA 08	20	421440	2114	2275756	1575	421440	886	1.36	68	122	6	12.5	100	1.9	327.8	3.4
TA 13	20	421760	2115	2949476	2053	421760	1628	1.29	22	87	4	11.6	85	1.8	327.8	3.4
AL 03	20	422080	2117	3141973	2288	422080	1109	1.35	93	141	6	12.5	100	1.8	327.8	3.4
AL 04	20	423340	2122	3014678	2490	423340	1327	1.44	70	129	5	12.3	100	1.7	327.8	3.4
AL 05	20	423660	2123	2707515	2370	423660	1449	1.47	32	113	5	12.1	100	1.5	327.8	3.4
AL 06	20	423980	2124	2724574	2136	423980	1156	1.30	32	127	6	12.1	100	1.6	327.8	3.4

	Sample	n	ρ_d	N_d	ρ_s	N_s	ρ_i	N_i	D_{par}	$P(\chi^2)$	FT Central Age	1 s.e. (AFT age)	MTL [μm]	nc	σ (MTL)	ζ	2 s.e. (ζ)
Samples from Brazil	AL 07	20	424300	2125	1120627	1233	424300	536	1.37	7	167	13	12.2	50	2.0	327.8	3.4
	AL 08	20	424620	2127	992806	1489	424620	935	1.24	71	110	5	12.8	100	1.7	327.8	3.4
	AL 11	20	424920	2128	1021632	1062	424920	480	1.35	8	153	11	12.2	100	1.7	327.8	3.4
	AL 12	20	425220	2129	1887469	1342	425220	647	1.40	1	146	11	12.2	100	1.9	327.8	3.4
	AL 13	20	425540	2130	2116821	1464	425540	531	1.29	20	191	12	11.6	85	1.6	327.8	3.4
Samples from India	TN-04	20	348200	2228	273500	1441	417200	534	1.64	99	119	7	12.7	100	1.6	253.1	2.4
	TN-06	30	349300	2235	331800	1033	160900	309	1.52	100	145	10	12.7	100	1.9	253.1	2.4
	TN-07	20	350500	2243	333500	1449	349200	447	1.38	99	146	9	11.8	100	1.7	253.1	2.4
	TN-10	20	351800	2251	315500	1393	970100	447	1.58	100	139	8	12.0	100	1.4	253.1	2.4
	TN-11	20	367200	2350	335100	1471	745700	462	1.60	99	154	9	12.4	100	1.7	253.1	2.4
	TN-12	20	368100	2356	315800	1652	414800	531	1.51	100	146	8	12.0	33	1.6	253.1	2.4
	TN-13	20	368700	2360	336900	1899	609400	577	1.50	75	155	8	11.8	100	1.5	253.1	2.4
	TN-14	20	369700	2366	326100	1089	303400	345	1.52	99	151	10	12.4	56	1.6	253.1	2.4
	TN-16	20	372000	2381	295500	345	92200	118		100	138	15				253.1	2.4
	TN-17	20	373000	2387	256600	629	196900	252	1.49	99	120	9	11.7	100	1.4	253.1	2.4
	TN-18	23	374100	2394	253200	192	51600	76	1.55	100	119	16	11.1	24	0.9	253.1	2.4
	TN-19	20	374900	2399	406600	1678	499600	415	1.60	86	190	11	12.4	100	1.4	253.1	2.4
	TN-20	16	375600	2404	371700	783	248500	214	1.65	99	174	14	11.8	32	1.3	253.1	2.4
	TN-21	11	376400	2409	358100	1344	640900	381	1.76	88	168	11	11.4	100	1.2	253.1	2.4
	TN-22	20	377200	2414	282400	1558	541500	569	1.43	95	133	7	11.6	100	1.5	253.1	2.4
	TN-23	20	378000	2419	301900	975	260900	334	1.42	99	143	10	11.2	100	1.4	253.1	2.4
	TN-25	20	382500	2448	273100	1226	357800	458	1.57	99	131	8	12.7	100	1.7	253.1	2.4
	TN-26	25	383200	2452	291500	1173	401100	408	1.50	100	140	9	13.0	63	1.9	253.1	2.4
	TN-27	20	384400	2460	284500	196	53100	68		100	137	20				253.1	2.4
	TN-28	20	385300	2466	287900	1522	511900	555	1.54	97	139	8	11.9	92	1.6	253.1	2.4
	TN-29	20	386200	2472	370400	100	421900	27		100	179	39				253.1	2.4
	TN-30	16	387100	2477	387700	1123	431700	286		96	187	13	11.5	30	1.1	253.1	2.4
	TN-31	20	387800	2482	382700	1478	509700	385	1.54	100	185	11	12.8	100	1.4	253.1	2.4
	TN-32	20	391800	2507	372700	2424	1057000	665	2	74	182	9	12.5	100	1.3	253.1	2.4

	Sample	n	ρ_d	N_d	ρ_s	N_s	ρ_i	N_i	D_{par}	$P(\chi^2)$	FT Central Age	1 s.e. (AFT age)	MTL [µm]	nc	σ (MTL)	ζ	2 s.e. (ζ)
	TN-33	20	392700	2514	343000	2348	1487000	685	2	99	168	8	13.0	100	1.2	253.1	2.4
	TN-34	20	393700	2520	352600	2311	1430000	659	2	99	173	9	12.7	100	1.3	253.1	2.4
	TN-35	20	394600	2525	252100	1387	443800	568	1	95	125	7	12.1	100	1.6	253.1	2.4
	TN-36	16	395600	2532	327300	374	118200	121	1	99	162	17	11.4	98	1.6	253.1	2.4
	TN-37	12	396500	2538	370000	1116	787800	298	1	99	183	13	12.0	100	1.2	253.1	2.4
	TN-38	20	397500	2544	387600	1434	289800	371	1	99	192	12	12.0	100	1.5	253.1	2.4
Samples from Colombia	124	25	581000	5290	1050000	984	733000	702	1	22	116	7	12.0	67	1.6	286.3	3.9
	134	20	581000	5623	762000	653	342000	300	1	19	177	15				286.3	3.9
	135	20	581000	5159	729000	909	430000	551	1	10	136	9				286.3	3.9
	164	20	553000	5624	394000	453	283000	320	1	96	111	8				286.3	3.9
	168	28	557000	5688	296000	329	242000	238	1	2	103	12				286.3	3.9
	170	25	558000	5714	554000	1147	324000	673	1	22	136	8	12.0	65	1.5	286.3	3.9
	171	25	560000	5746	1040000	1101	725000	778	1	20	112	6	11.6	100	2.0	286.3	3.9
	175	25	562000	5771	754000	1060	575000	803	1	73	105	5	11.9	100	1.7	286.3	3.9
	176	20	564000	5789	3050000	1570	3110000	1591	1	85	79	0	11.9	100	1.7	286.3	3.9
	180	25	572000	5830	639000	1101	581000	1023	2	10	87	1	11.7	100	1.7	286.3	3.9
	182	30	573000	5824	883000	1462	746000	1256	1	7	96	1	12.5	100	1.6	286.3	3.9
	501	25	574000	5811	359000	405	197000	225	1	78	146	13				286.3	3.9
	502	25	575000	5798	1630000	1397	1140000	985	1	14	117	6	11.8	100	1.6	286.3	3.9
	505	20	576000	5783	1720000	1692	1270000	1221	1	56	113	5	11.7	100	1.4	286.3	3.9
	506	25	577000	5763	2180000	2193	1670000	1634	1	0	111	6	12.2	100	1.6	286.3	3.9
	510	20	578000	5735	1490000	1092	1090000	804	1	24	112	6	12.3	100	1.5	286.3	3.9
	512	25	580000	5509	307000	380	235000	288	1	60	109	9				286.3	3.9
	515	25	581000	5462	858000	1184	677000	922	1	22	106	5	12.4	85	1.8	286.3	3.9
	516	18	581000	5404	348000	254	250000	183	1	44	113	12				286.3	3.9

Table A.3: AFT data from previous studies. Sample localities (in World Geodetic System – WGS84), Z: elevation above sea level (m), AFT age (Ma), and references.

Samples from Brazil

sample	long	lat	Z	AFT central_age	1 s.e. (AFT age)	reference
TFSEB-01B	-41.457	-21.8182	120	39	5	Carmo 2005
TFSEB-02	-41.7236	-21.6535	20	56	6	Carmo 2005
TFSEB-04	-42.1512	-21.6005	100	58	5	Carmo 2005
TFSEB-07A	-42.595	-21.5003	220	74	9	Carmo 2005
TFSEB-08	-42.7842	-21.3398	230	96	6	Carmo 2005
TFSEB-11	-43.4569	-21.241	580	96	11	Carmo 2005
TFSEB-12	-43.6089	-21.2676	930	118	8	Carmo 2005
TFSEB-16	-43.7461	-21.2831	1030	131	18	Carmo 2005
TFSEB-18	-44.3167	-21.0826	980	178	22	Carmo 2005
TFSEB-20	-44.0421	-20.3246	800	153	18	Carmo 2005
TFSEB-21	-43.9748	-20.2949	1030	142	18	Carmo 2005
TFSEB-22	-43.9594	-20.2873	1160	167	22	Carmo 2005
TFSEB-24	-43.6922	-20.3244	960	175	19	Carmo 2005
TFSEB-29	-43.2988	-20.1489	680	162	16	Carmo 2005
TFSEB-30	-43.2676	-20.1361	680	114	11	Carmo 2005
TFSEB-31	-43.0905	-20.1253	590	109	12	Carmo 2005
TFSEB-32	-42.9651	-20.1093	700	87	9	Carmo 2005
TFSEB-34	-42.7803	-20.0521	240	67	8	Carmo 2005
TFSEB-47	-40.3953	-20.3	60	81	9	Carmo 2005
TFSEB-49	-41.0223	-20.3821	1100	69	3	Carmo 2005
TFSEB-50	-41.2308	-20.2989	980	59	7	Carmo 2005
TFSEB-51	-41.7818	-20.255	570	77	8	Carmo 2005
TFSEB-52	-42.0679	-20.2704	650	94	11	Carmo 2005
ES-07	-41.6	-20.256	710	97	6	Jelinek et al. 2014
ES-08	-41.406	-20.2373	1170	80	6	Jelinek et al. 2014
ES-09	-41.0873	-20.35	918	64	5	Jelinek et al. 2014
ES-10	-40.7687	-20.4187	774	69	7	Jelinek et al. 2014
ES-13	-40.3187	-20.106	38	75	7	Jelinek et al. 2014
ES-14	-40.3687	-19.8	115	72	7	Jelinek et al. 2014
ES-15	-40.5687	-19.5373	62	97	10	Jelinek et al. 2014
ES-16	-40.5873	-19.1873	192	87	8	Jelinek et al. 2014
ES-18	-40.55	-18.5873	144	88	8	Jelinek et al. 2014
ES-19	-40.356	-18.3247	146	78	4	Jelinek et al. 2014
ES-20	-40.3687	-18.1187	190	95	7	Jelinek et al. 2014
ES-22	-40.4873	-17.7747	148	39	4	Jelinek et al. 2014
ES-23	-40.8	-17.7873	210	68	7	Jelinek et al. 2014
ES-24	-41.056	-17.8687	345	71	6	Jelinek et al. 2014
ES-25	-41.4187	-17.8373	399	112	8	Jelinek et al. 2014
ES-26	-41.656	-17.806	559	117	15	Jelinek et al. 2014
ES-27	-41.6687	-18.0247	333	98	8	Jelinek et al. 2014
ES-28	-41.8187	-18.4	303	93	7	Jelinek et al. 2014
ES-29	-41.9687	-18.8373	199	71	6	Jelinek et al. 2014

	ES-31	-41.2873	-18.7747	276	56	4	Jelinek et al. 2014
	ES-33	-42.1373	-19.8	583	77	6	Jelinek et al. 2014
	ES-34	-42.256	-20.656	808	77	6	Jelinek et al. 2014
	MG-71	-40.7422	-20.0337	705	118	6	Jelinek et al. 2014
	MG-72	-40.8845	-20.143	1020	126	12	Jelinek et al. 2014
	B1	-43.6953	-20.3234	958	159	10	Amaral-Santos et al. 2019
	Cb1	-43.5513	-19.871	1050	137	8	Amaral-Santos et al. 2019
	Co1	-43.5016	-18.8802	667	119	18	Amaral-Santos et al. 2019
	G1	-43.749	-18.4543	1044	129	8	Amaral-Santos et al. 2019
	G2	-43.733	-18.4605	1023	159	13	Amaral-Santos et al. 2019
	G3	-43.7631	-18.612	967	103	14	Amaral-Santos et al. 2019
	Ic1	-43.2446	-17.0678	777	187	18	Amaral-Santos et al. 2019
	Sm1	-43.2272	-18.0319	716	92	7	Amaral-Santos et al. 2019
	Sm2	-43.2281	-17.9506	411	137	10	Amaral-Santos et al. 2019
	BR-01	-41.8366	-20.4106	1985	145	33	Van Ranst et al. 2020
	BR-03	-41.8546	-20.4226	1215	84	5	Van Ranst et al. 2020
	BR-05	-41.9078	-20.4437	1014	64	2	Van Ranst et al. 2020
	BR-07	-41.777	-20.2614	520	63	3	Van Ranst et al. 2020
	BR-08	-41.4419	-20.2183	955	77	3	Van Ranst et al. 2020
	BR-09	-41.2583	-20.284	1086	71	3	Van Ranst et al. 2020
	BR-10	-41.0911	-20.3521	949	62	2	Van Ranst et al. 2020
	BR-11	-41.0174	-20.3873	1112	68	3	Van Ranst et al. 2020
	BR-12	-40.8597	-20.4123	818	62	3	Van Ranst et al. 2020
	BR-13	-40.273	-20.3339	1	81	5	Van Ranst et al. 2020
	BR-14	-40.3215	-20.1138	37	81	4	Van Ranst et al. 2020
	BR-16	-40.4096	-19.9324	59	72	2	Van Ranst et al. 2020
	BR-17	-40.4989	-19.9352	415	79	4	Van Ranst et al. 2020
	BR-19	-40.6786	-19.8146	141	73	3	Van Ranst et al. 2020
	BR-20	-40.6223	-19.5311	49	64	6	Van Ranst et al. 2020
	BR-22	-40.6809	-19.4084	62	78	3	Van Ranst et al. 2020
	BR-23	-40.808	-19.2362	115	78	3	Van Ranst et al. 2020
	BR-24	-40.8664	-19.2295	736	90	5	Van Ranst et al. 2020
	BR-25	-40.9089	-19.2184	437	81	4	Van Ranst et al. 2020
	BR-26	-40.9669	-19.0807	485	81	3	Van Ranst et al. 2020
	BR-27	-41.1081	-18.8674	604	67	2	Van Ranst et al. 2020
	BR-28	-41.2654	-18.7963	329	88	4	Van Ranst et al. 2020
	BR-29	-41.4108	-18.7506	351	84	4	Van Ranst et al. 2020
	BR-30	-41.9624	-18.8355	188	70	4	Van Ranst et al. 2020
Samples from India	IND 1835	12.375	75.5417	950	223	10	Gunnell et al. (2003)
	IND 1836	12.3833	75.5167	1110	238	16	Gunnell et al. (2003)
	IND 1837	12.3833	75.4931	1200	205	9	Gunnell et al. (2003)
	IND 1838	12.4333	75.4722	760	240	12	Gunnell et al. (2003)
	IND 1839	13.15	74.8444	50	192	10	Gunnell et al. (2003)
	IND 1840	13.6486	74.8292	50	216	9	Gunnell et al. (2003)
	IND 1841	13.7917	74.8	60	254	13	Gunnell et al. (2003)
	IND 1844	13.1833	75.0819	80	170	6	Gunnell et al. (2003)
	IND 1845	12.9417	76.0944	980	248	14	Gunnell et al. (2003)
	IND 1846	12.4458	75.4639	540	269	16	Gunnell et al. (2003)

IND 1847	12.45	75.4333	350	145	6	Gunnell et al. (2003)
IND 1849	12.8333	75.5	100	161	7	Gunnell et al. (2003)
IND 1850	12.8417	75.6556	340	183	5	Gunnell et al. (2003)
IND 1851	12.8833	74.9667	100	232	9	Gunnell et al. (2003)
IND 1852	12.9167	74.9167	80	211	10	Gunnell et al. (2003)
IND 1853	12.2792	76.6792	1000	164	9	Gunnell et al. (2003)
IND 1854	12.2833	76.6833	780	162	8	Gunnell et al. (2003)
IND 1856	14.2486	74.6167	40	235	7	Gunnell et al. (2003)
IND 1857	13.375	74.8986	30	198	8	Gunnell et al. (2003)
IND 1859	12.8944	75.7306	810	312	17	Gunnell et al. (2003)
IND 1860	12.7333	74.9667	60	241	27	Gunnell et al. (2003)
IND 1862	12.8472	75.7	540	233	9	Gunnell et al. (2003)
IND 1863	12.8556	75.85	930	315	17	Gunnell et al. (2003)
IND 1865	12.925	75.6972	980	360	19	Gunnell et al. (2003)
IND 1933	15.5915	73.733	0	188	11	Gunnell et al. (2003)
IND 1936	16.0535	73.7072	36	225	14	Gunnell et al. (2003)
IND 1937	16.056	73.541	35	166	6	Gunnell et al. (2003)
IND 1940	16.2538	73.7147	59	147	6	Gunnell et al. (2003)
IND 1941	16.164	73.4244	59	67	6	Gunnell et al. (2003)
IND 1943	15.938	73.9707	512	321	23	Gunnell et al. (2003)
IND 1948	16.0143	73.9572	180	227	14	Gunnell et al. (2003)
IND 1950	15.9778	73.927	137	206	8	Gunnell et al. (2003)
IND 1952	15.9227	73.8552	120	188	11	Gunnell et al. (2003)
IND 1960	14.2237	74.8102	272	243	19	Gunnell et al. (2003)
IND 1965	14.2739	74.7106	249	169	10	Gunnell et al. (2003)
IND 1967	14.2773	74.7203	425	263	15	Gunnell et al. (2003)
IND 1972	14.3592	74.7355	470	249	13	Gunnell et al. (2003)
IND 1974	14.3932	74.66	330	238	17	Gunnell et al. (2003)
IND 1977	14.431	74.6098	30	190	8	Gunnell et al. (2003)
IND 1981	14.7635	74.2143	18	108	5	Gunnell et al. (2003)
IND 1984	14.6953	74.3	85	154	7	Gunnell et al. (2003)
IND 1985	14.6865	74.358	121	136	8	Gunnell et al. (2003)
IND 1990	15.0633	74.738	183	276	23	Gunnell et al. (2003)
IND 1991	14.8463	74.1312	12	62	5	Gunnell et al. (2003)
IND 1997	14.9467	74.3597	352	222	14	Gunnell et al. (2003)
IND 1999	14.9775	74.3705	524	218	20	Gunnell et al. (2003)
IND 2000	15.0102	74.3563	508	245	11	Gunnell et al. (2003)
IND 2004	15.4155	74.495	660	214	12	Gunnell et al. (2003)
IND 2010	15.5262	74.1185	40	190	12	Gunnell et al. (2003)
IND 2011	15.6087	74.1083	268	286	18	Gunnell et al. (2003)
IND 2018	15.6843	75.5062	690	197	11	Gunnell et al. (2003)
IND 2023	15.4382	74.487	614	234	12	Gunnell et al. (2003)
IND 2029	15.4132	74.2548	335	194	13	Gunnell et al. (2003)
IND 2030	14.9922	74.1495	43	168	10	Gunnell et al. (2003)
IND 2033	15.02	74.0348	51	69	5	Gunnell et al. (2003)
IND 2037	15.1928	73.9958	15	160	5	Gunnell et al. (2003)
IND 2039	15.213	74.0375	345	54	4	Gunnell et al. (2003)
IND 2046	15.4943	73.7682	0	100	8	Gunnell et al. (2003)

IND 2052	13.675	75.25	640	232	14	Gunnell et al. (2003)
IND 2055	13.3653	75.5333	750	206	8	Gunnell et al. (2003)
IND 2057	14.2333	74.8444	540	281	15	Gunnell et al. (2003)
IND 2062	14.6944	74.3583	130	153	9	Gunnell et al. (2003)
IND 2064	15.4	74.4972	700	270	16	Gunnell et al. (2003)
IND 2065	15.1583	74.6083	580	225	18	Gunnell et al. (2003)
IND 2066	14.3833	76.0833	660	282	14	Gunnell et al. (2003)
IND 2067	14.2667	76.35	680	226	14	Gunnell et al. (2003)
IND 2069	13.5333	76	780	287	15	Gunnell et al. (2003)
IND 2070	13.3333	77.1667	830	285	2	Gunnell et al. (2003)
IND 2071	12.9667	77.6167	900	226	8	Gunnell et al. (2003)
IND 2297	13.1917	75.4	840	230	15	Gunnell et al. (2003)
IND 2298	13.25	77.25	920	232	13	Gunnell et al. (2003)
IND 2300	12.7917	75.7917	900	314	16	Gunnell et al. (2003)
IND 2301	13.5556	74.8	40	213	10	Gunnell et al. (2003)
IND 2302	13.7167	74.9917	500	323	23	Gunnell et al. (2003)
IND 2305	11.4167	76.6667	2320	411	24	Gunnell et al. (2003)
IND 2307	13.0319	75.3	480	255	13	Gunnell et al. (2003)
IND 2308	11.6667	76.5	1460	351	22	Gunnell et al. (2003)
IND 2309	13.0333	75.3083	130	223	10	Gunnell et al. (2003)
IND 2310	13.8917	74.6167	20	255	12	Gunnell et al. (2003)
IND 2312	13.6417	74.8167	40	254	15	Gunnell et al. (2003)
IND 2313	13.7333	75.0139	600	215	10	Gunnell et al. (2003)
IND 2314	14.8403	74.5097	440	276	24	Gunnell et al. (2003)
IND 2316	14.1333	74.5056	30	75	5	Gunnell et al. (2003)
IND 2317	15.8	74.2292	620	291	27	Gunnell et al. (2003)
IND 2318	14.5472	74.7	540	227	14	Gunnell et al. (2003)
IND 2319	14.5306	74.6083	400	275	16	Gunnell et al. (2003)
IND 2321	15.4236	75.4417	630	249	22	Gunnell et al. (2003)
IND 2323	13.2819	75.8625	1000	317	14	Gunnell et al. (2003)
IND 2324	15.7333	74.4917	720	355	58	Gunnell et al. (2003)
IND 2325	15.5639	75.35	620	258	15	Gunnell et al. (2003)
IND 2326	13.5333	75.3458	760	261	16	Gunnell et al. (2003)
X	8.127861	77.517167	20	49.4	6.6	Herman et al. (2013)
X	8.443806	77.206583	90	63.8	3.1	Herman et al. (2013)
X	8.607028	76.906639	20	53.2	3.7	Herman et al. (2013)
X	8.699583	76.906639	60	57.1	8.4	Herman et al. (2013)
X	8.70525	77.43225	80	77.4	3.8	Herman et al. (2013)
X	9.010333	76.878417	163	65.7	3.6	Herman et al. (2013)
X	10.210933	77.028764	1963	380	19	Herman et al. (2013)
X	11.276028	76.958639	390	208	13	Herman et al. (2013)
X	11.332019	76.840483	1197	333	16	Herman et al. (2013)
X	11.343819	76.876133	457	271	20	Herman et al. (2013)
X	11.346644	76.788797	1760	317	15	Herman et al. (2013)
X	11.391453	76.717347	2195	380	19	Herman et al. (2013)
X	11.402403	76.738592	2407	390	26	Herman et al. (2013)
X	11.495069	76.471972	880	274	22	Herman et al. (2013)
X	11.57	76.559094	888	383	27	Herman et al. (2013)

	x	11.581425	76.575169	873	241	9	Herman et al. (2013)
	x	11.749392	76.647333	863	220	14	Herman et al. (2013)
	x	12.019803	76.680167	925	172	7	Herman et al. (2013)
	x	12.264011	76.673203	817	133	15	Herman et al. (2013)

REFERENCES

Amaral-Santos, E., Jelinek, A. R., Almeida-Abreu, P. A., & Genezine, F. A. (2019). Phanerozoic cooling history of Archean/Paleoproterozoic basement in the southern Espinhaço Range, southeastern Brazil, through apatite fission-track analysis. Journal of South American Earth Sciences, 96, 102352. https://doi.org/10.1016/j.jsames.2019.102352

Carmo, I. D. O. (2005). Geocronologia do Intemperismo Cenozóico no Sudeste do Brasil.

Fonseca, A. C., Piffer, G. V., Nachtergaele, S., Van Ranst, G., De Grave, J., & Novo, T. A. (2020). Devonian to Permian post-orogenic denudation of the Brasília Belt of West Gondwana: Insights from apatite fission track thermochronology. Journal of Geodynamics, 137. https://doi.org/10.1016/j.jog.2020.101733

Fonseca, A. C. L., Novo, T. A., Nachtergaele, S., Fonte-Boa, T. M. R., Van Ranst, G., & De Grave, J. (2021). Differential Phanerozoic evolution of cratonic and non-cratonic lithosphere from a thermochronological perspective: São Francisco Craton and marginal orogens (Brazil). Gondwana Research, 93, 106–126. https://doi.org/10.1016/j.gr.2021.01.006

Fonseca, A., Cruz, S., Novo, T., He, Z., & Grave, J. De. (2022a). Differential exhumation of cratonic and non-cratonic lithosphere revealed by apatite fission-track thermochronology along the edge of the São Francisco craton, eastern Brazil. Scientific Reports, 1–9. https://doi.org/10.1038/s41598-022-06419-w

Fonseca, A., Novo, T., Fonte-Boa, T., Kuchenbecker, M., Fragoso, D. G. C., Peifer, D., Pedrosa-Soares, A. C., & de Grave, J. (2023). Control of inherited structural fabric on the development and exhumation of passive margins – Insights from the Araçuaí Orogen (Brazil). Geoscience Frontiers, 14(6), 101628. https://doi.org/10.1016/j.gsf.2023.101628

Fonseca, A., Nachtergaele, S., Bonilla, A., Dewaele, S., & de Grave, J. (2024). Extensional exhumation of cratons: insights from the Early Cretaceous Rio Negro–Juruena belt (Amazonian Craton, Colombia). Solid Earth, 15(2), 329–352. https://doi.org/10.5194/se-15-329-2024

Fonseca, A., Glorie, S., He, Z., Singh, T., & de Grave, J. (2022b). Contrasting thermal histories for the Indian passive margins during syn- and post-Gondwana break-up: Insights from apatite fission-track thermochronology. Terra Nova, 34(6), 543–553. https://doi.org/10.1111/ter.12621

Jelinek, A. R., Chemale, F., van der Beek, P. A., Guadagnin, F., Cupertino, J. A., & Viana, A. (2014). Denudation history and landscape evolution of the northern East-Brazilian continental margin from apatite fission-track thermochronology. Journal of South American Earth Sciences, 54, 158–181. https://doi.org/10.1016/j.jsames.2014.06.001

Gunnell, Y., Gallagher, K., Carter, A., Widdowson, M., & Hurford, A. J. (2003). Denudation history of the continental margin of western peninsular India since the early Mesozoic - reconciling apatite fission-track data with geomorphology. Earth and Planetary Science Letters, 215(1–2), 187–201. https://doi.org/10.1016/S0012-821X(03)00380-7

Herman, F., Seward, D., Valla, P. G., Carter, A., Kohn, B., Willett, S. D., & Ehlers, T. A. (2013). Worldwide acceleration of mountain erosion under a cooling climate. Nature, 504(7480), 423–426. https://doi.org/10.1038/nature12877

van Ranst, G., Pedrosa-Soares, A. C., Novo, T., Vermeesch, P., & de Grave, J. (2020). New insights from low-temperature thermochronology into the tectonic and geomorphologic evolution of the south-eastern Brazilian highlands and passive margin. Geoscience Frontiers, 11(1), 303–324. https://doi.org/10.1016/j.gsf.2019.05.011

MODELING RESULTS

(next pages)

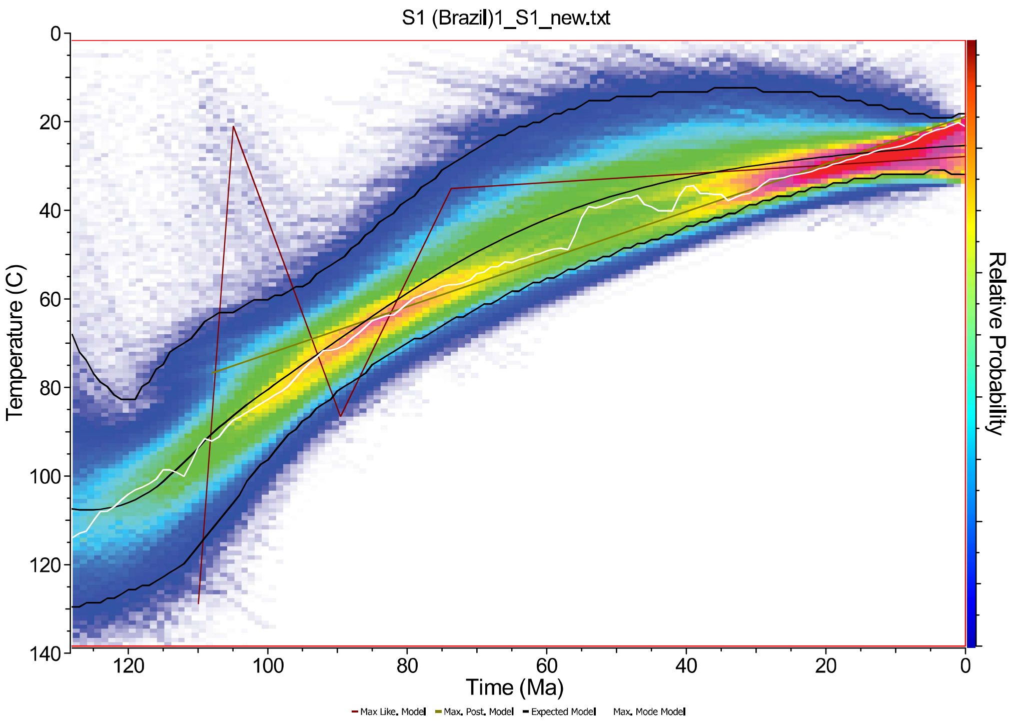
S1 (Brazil)1_S1_new.txt
Temperature (C)
Time (Ma)
Relative Probability
0
20
40
60
80
100
120
140
120
100
80
60
40
20
0
Max Like. Model
Max. Post. Model
Expected Model
Max. Mode Model

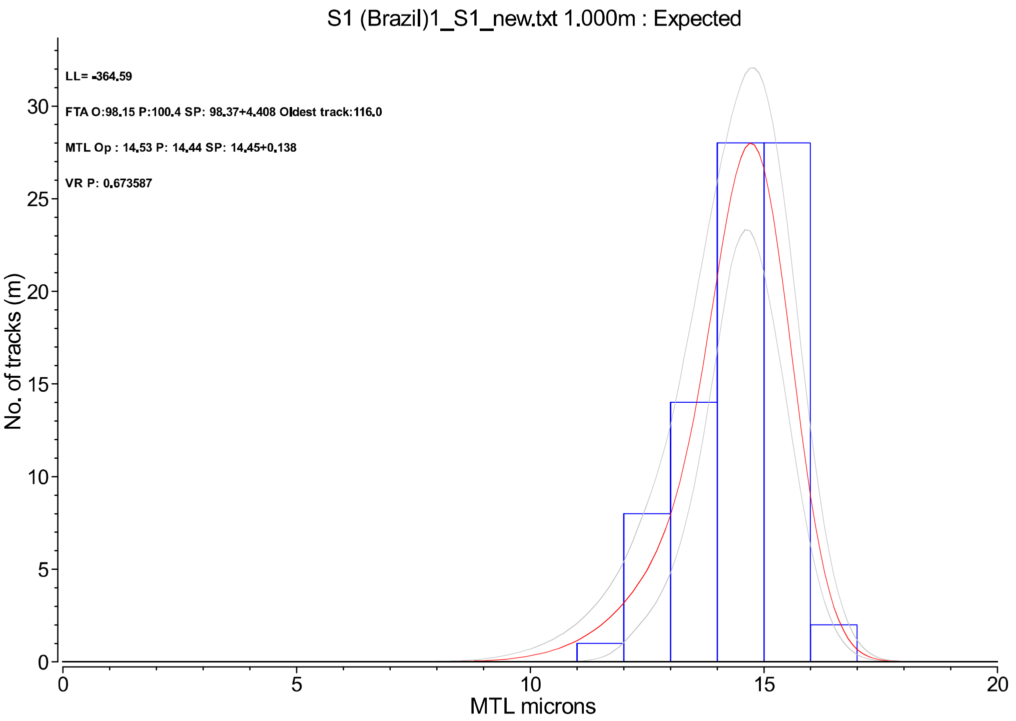
S1 (Brazil)1_S1_new.txt 1.000m : Expected
LL= -364.59
FTA O:98.15 P:100.4 SP: 98.37+4.408 Oldest track:116.0
MTL Op : 14.53 P: 14.44 SP: 14.45+0.138
VR P: 0.673587
No. of tracks (m)
MTL microns
0
5
10
15
20
25
30
0
5
10
15
20

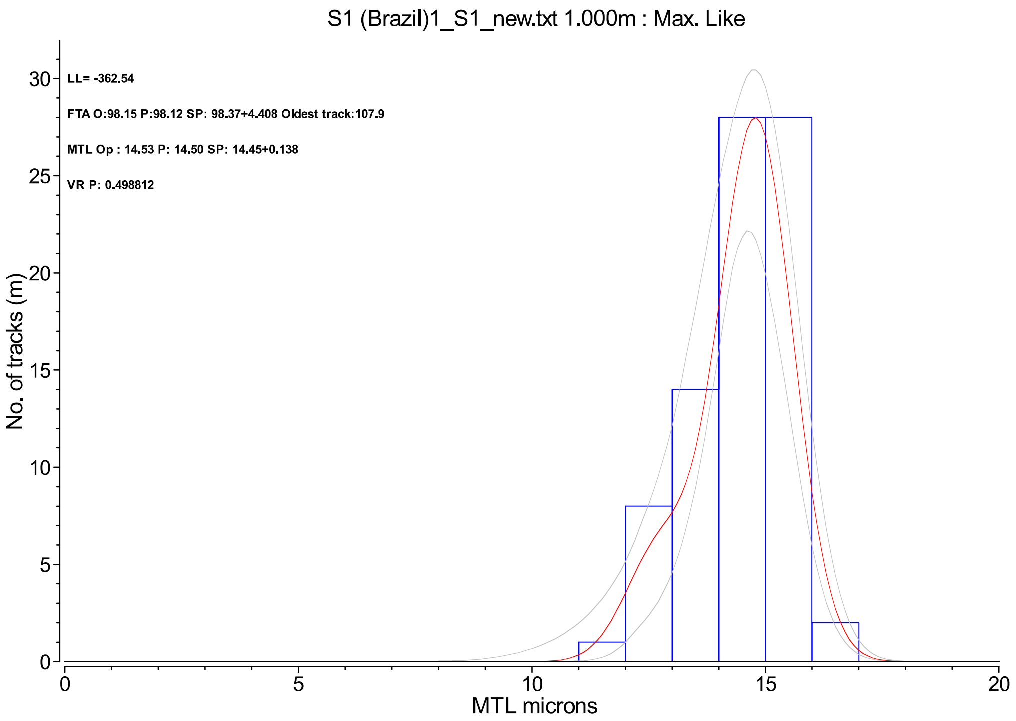
S1 (Brazil)1_S1_new.txt 1.000m : Max. Like
LL= -362.54
FTA O:98.15 P:98.12 SP: 98.37+4.408 Oldest track:107.9
MTL Op : 14.53 P: 14.50 SP: 14.45+0.138
VR P: 0.498812
No. of tracks (m)
MTL microns
0
5
10
15
20
25
30
0
5
10
15
20

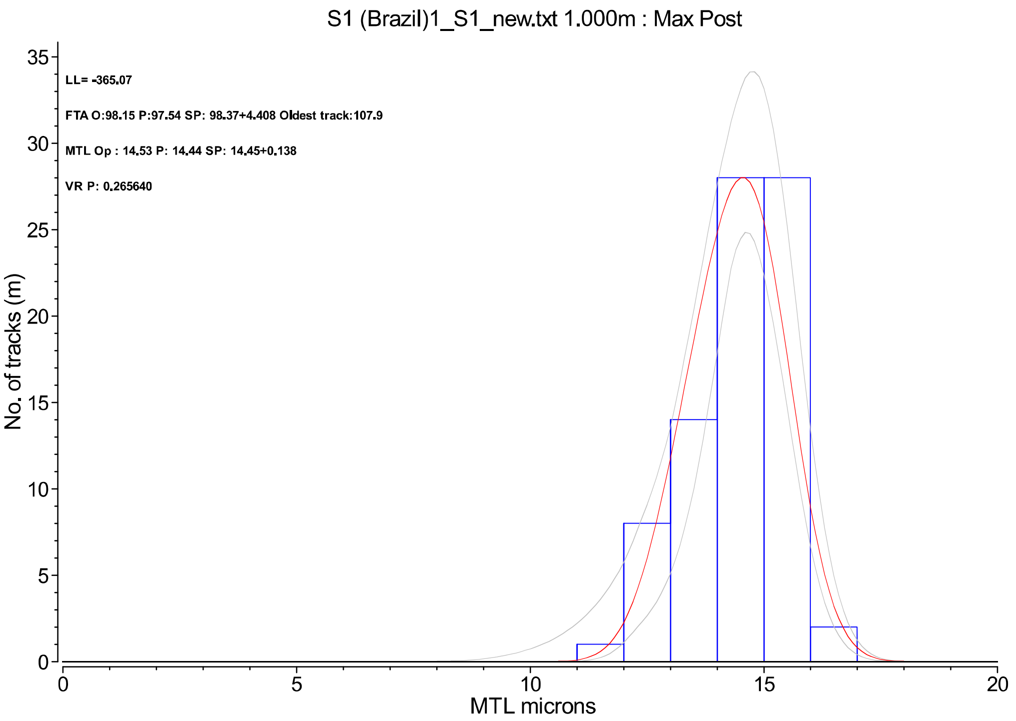
S1 (Brazil)1_S1_new.txt 1.000m : Max Post
LL= -365.07
FTA O:98.15 P:97.54 SP: 98.37+4.408 Oldest track:107.9
MTL Op : 14.53 P: 14.44 SP: 14.45+0.138
VR P: 0.265640
No. of tracks (m)
MTL microns
0
5
10
15
20
25
30
35
0
5
10
15
20

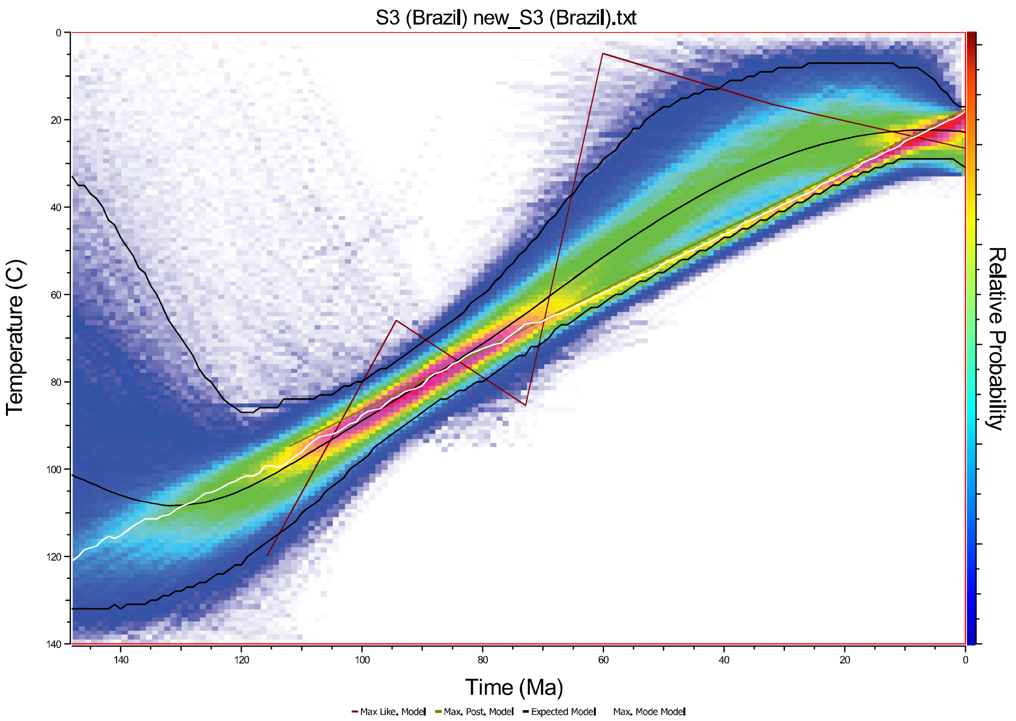
S3 (Brazil) new_S3 (Brazil).txt
Temperature (C)
Time (Ma)
Relative Probability
Max Like. Model
Max. Post. Model
Expected Model
Max. Mode Model

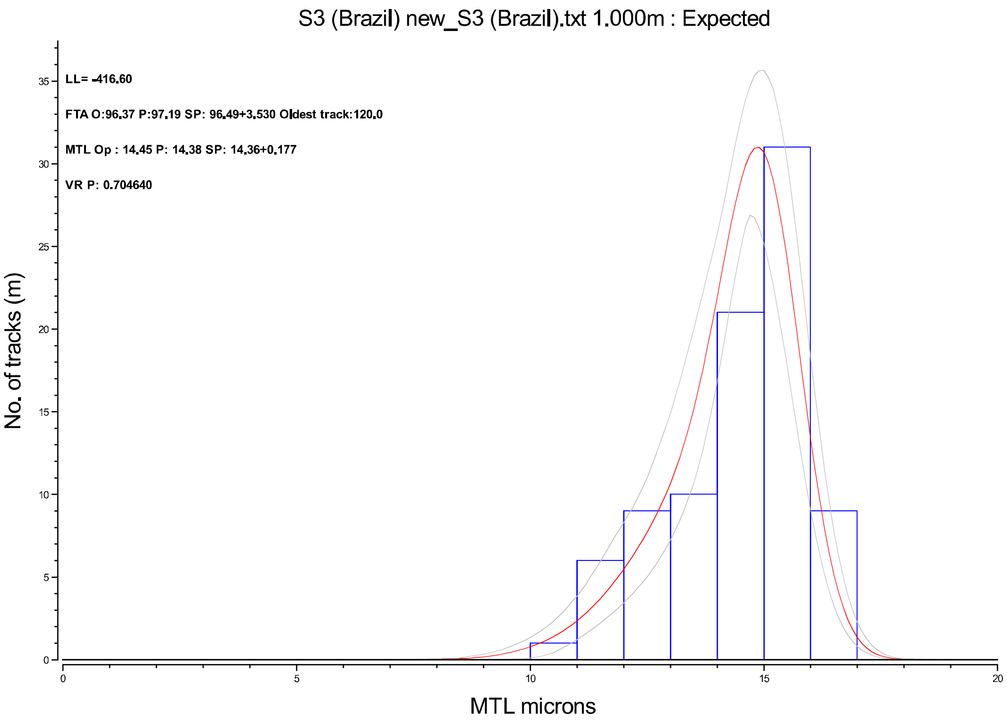
S3 (Brazil) new_S3 (Brazil).txt 1.000m : Expected
LL= -416.60
FTA O:96.37 P:97.19 SP: 96.49+3.530 Oldest track:120.0
MTL Op : 14.45 P: 14.38 SP: 14.36+0.177
VR P: 0.704640
No. of tracks (m)
MTL microns

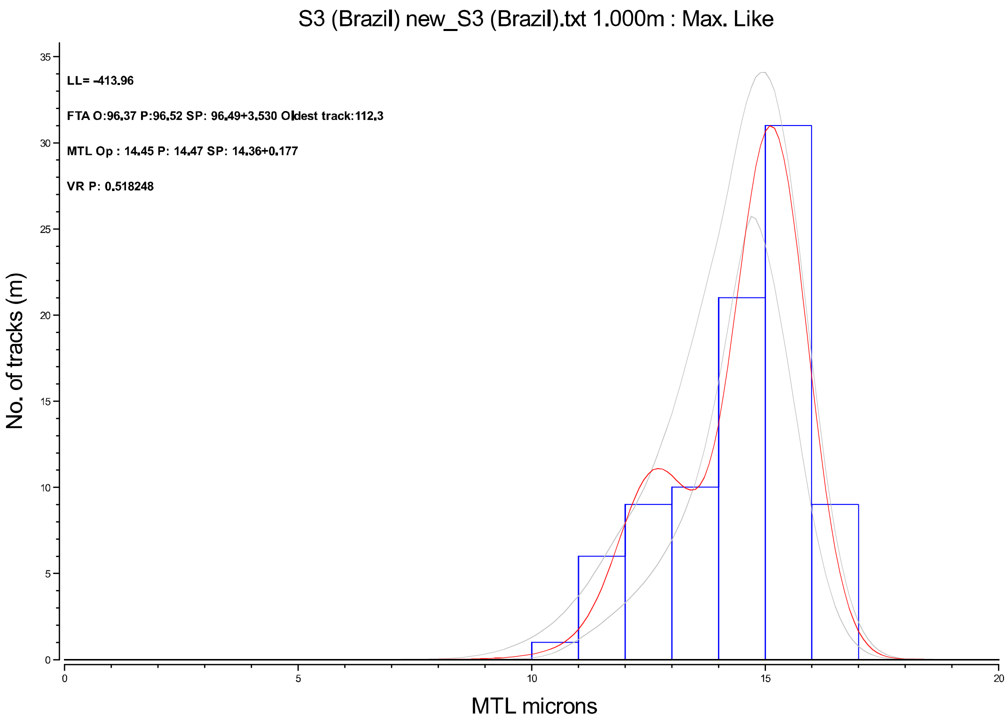
S3 (Brazil) new_S3 (Brazil).txt 1.000m : Max. Like
LL= -413.96
FTA O:96.37 P:96.52 SP: 96.49+3.530 Oldest track:112.3
MTL Op : 14.45 P: 14.47 SP: 14.36+0.177
VR P: 0.518248
No. of tracks (m)
MTL microns

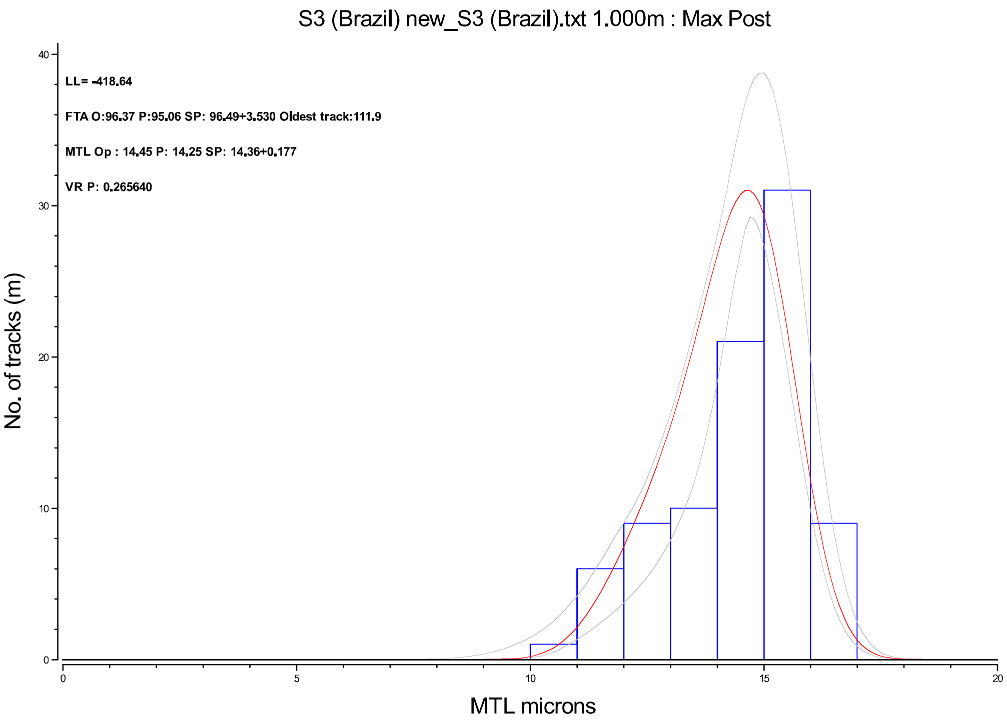
S3 (Brazil) new_S3 (Brazil).txt 1.000m : Max Post
LL= -418.64
FTA O:96.37 P:95.06 SP: 96.49+3.530 Oldest track:111.9
MTL Op : 14.45 P: 14.25 SP: 14.36+0.177
VR P: 0.265640
No. of tracks (m)
MTL microns

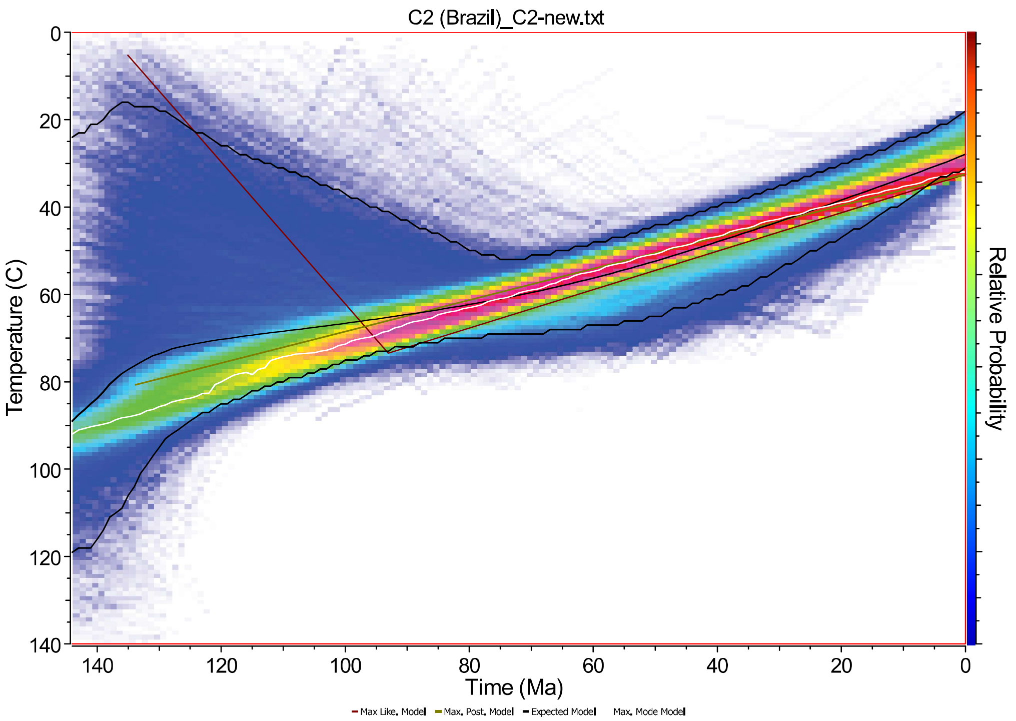

C2 (Brazil)_C2-new.txt
Temperature (C)
Time (Ma)
Relative Probability
0
20
40
60
80
100
120
140
140
120
100
80
60
40
20
0
Max Like. Model
Max. Post. Model
Expected Model
Max. Mode Model

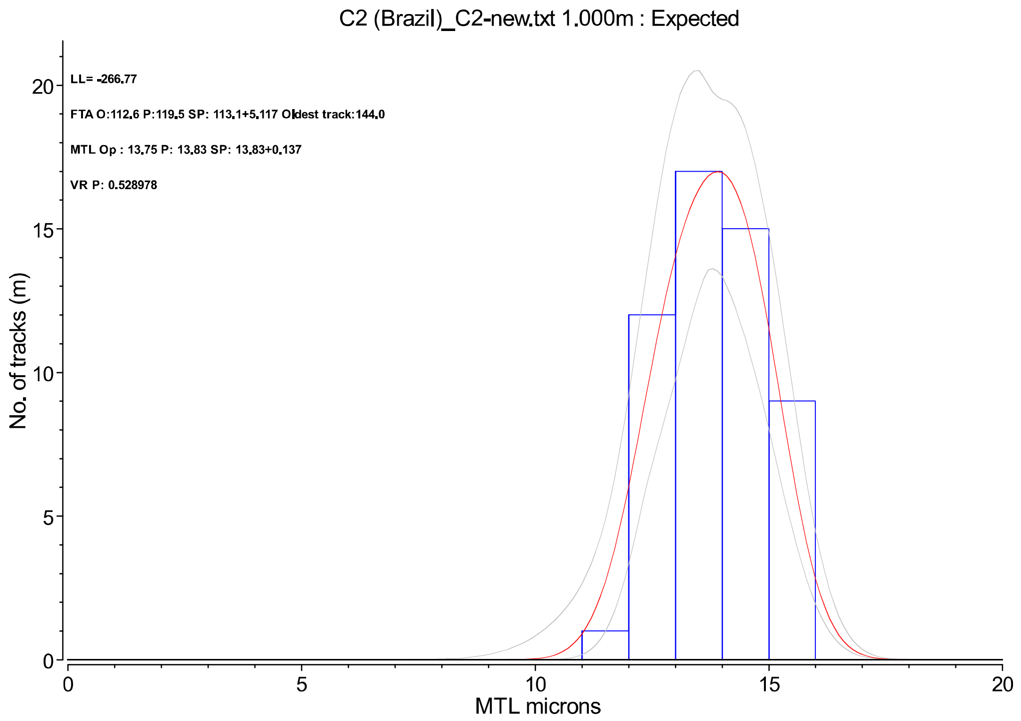

C2 (Brazil)_C2-new.txt 1.000m : Expected
LL= -266.77
FTA O:112.6 P:119.5 SP: 113.1+5.117 Oldest track:144.0
MTL Op : 13.75 P: 13.83 SP: 13.83+0.137
VR P: 0.528978
No. of tracks (m)
MTL microns
0
5
10
15
20
0
5
10
15
20

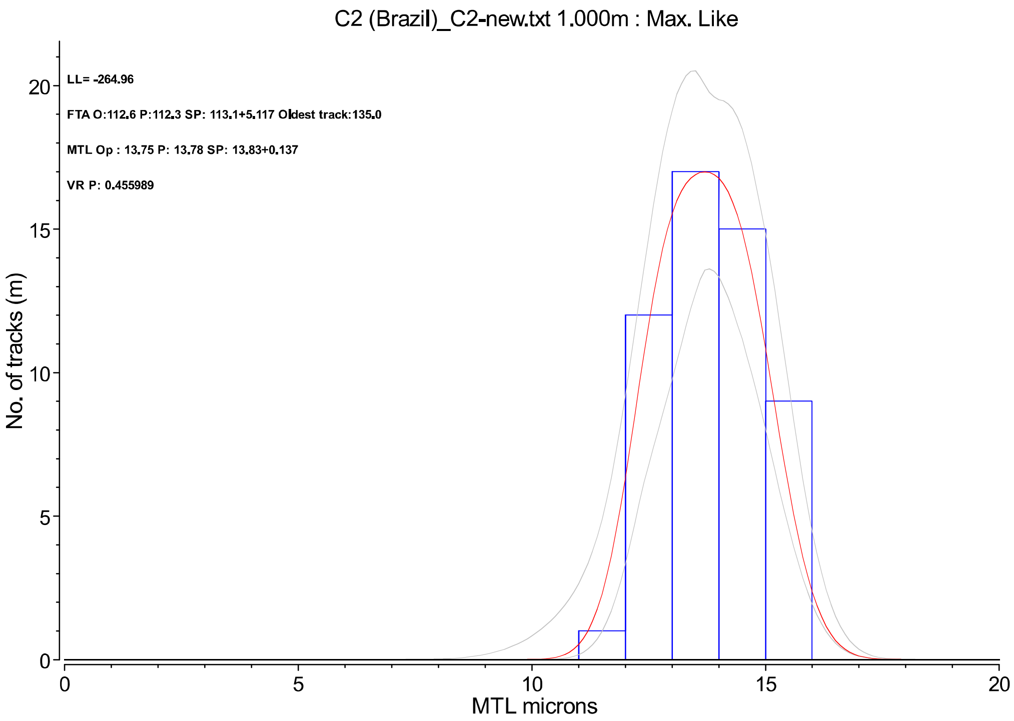

C2 (Brazil)_C2-new.txt 1.000m : Max. Like
LL= -264.96
FTA O:112.6 P:112.3 SP: 113.1+5.117 Oldest track:135.0
MTL Op : 13.75 P: 13.78 SP: 13.83+0.137
VR P: 0.455989
No. of tracks (m)
MTL microns
0
5
10
15
20
0
5
10
15
20

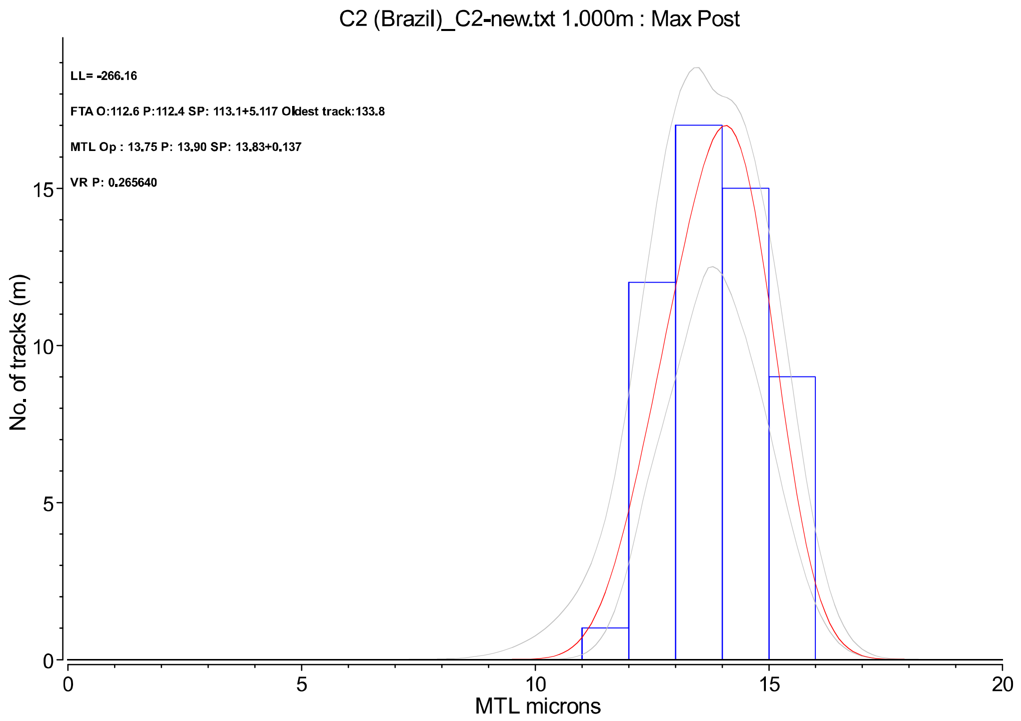

C2 (Brazil)_C2-new.txt 1.000m : Max Post
LL= -266.16
FTA O:112.6 P:112.4 SP: 113.1+5.117 Oldest track:133.8
MTL Op : 13.75 P: 13.90 SP: 13.83+0.137
VR P: 0.265640
No. of tracks (m)
MTL microns
0
5
10
15
0
5
10
15
20

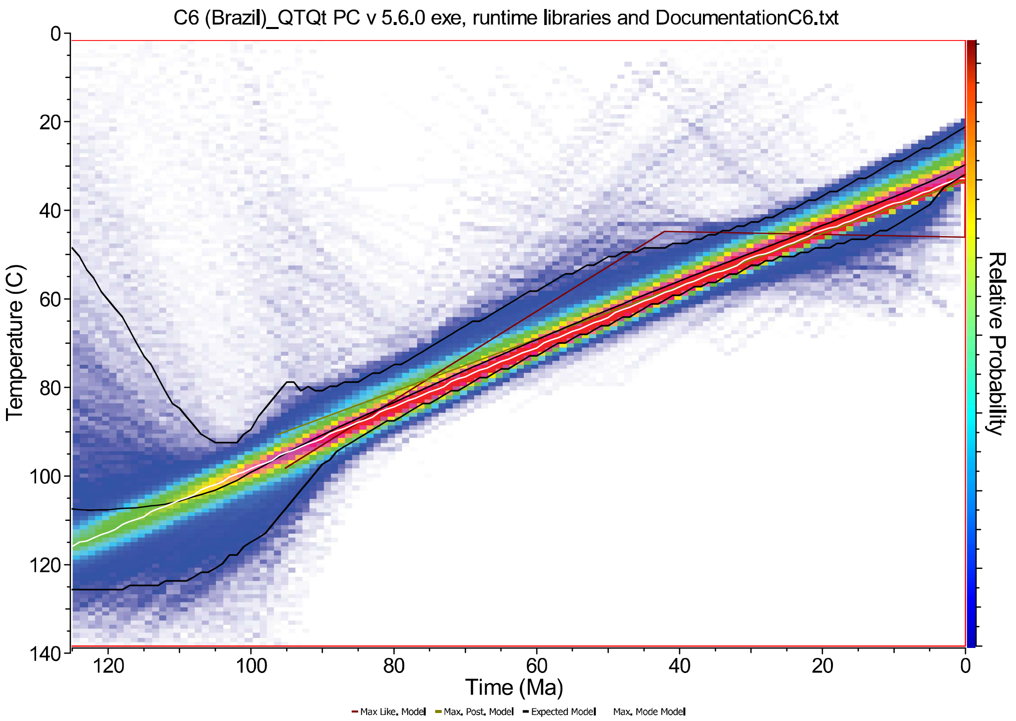
C6 (Brazil)_QTQt PC v 5.6.0 exe, runtime libraries and DocumentationC6.txt
Temperature (C)
Time (Ma)
Relative Probability
Max Like. Model
Max. Post. Model
Expected Model
Max. Mode Model

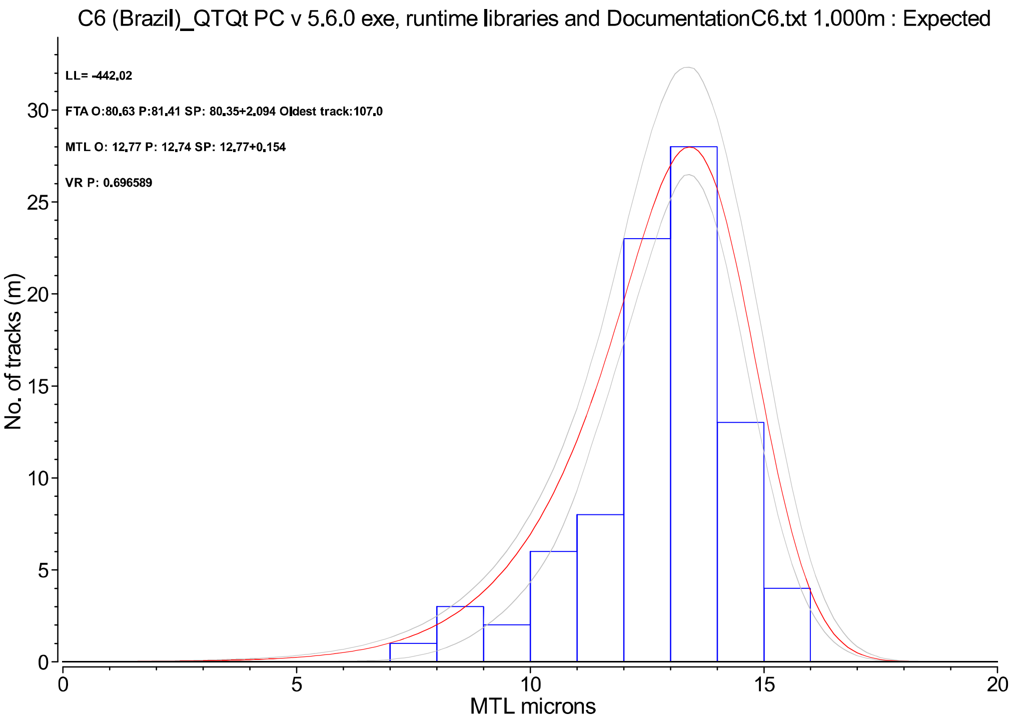
C6 (Brazil)_QTQt PC v 5.6.0 exe, runtime libraries and DocumentationC6.txt 1.000m : Expected
LL= -442.02
FTA O:80.63 P:81.41 SP: 80.35+2.094 Oldest track:107.0
MTL O: 12.77 P: 12.74 SP: 12.77+0.154
VR P: 0.696589
No. of tracks (m)
MTL microns

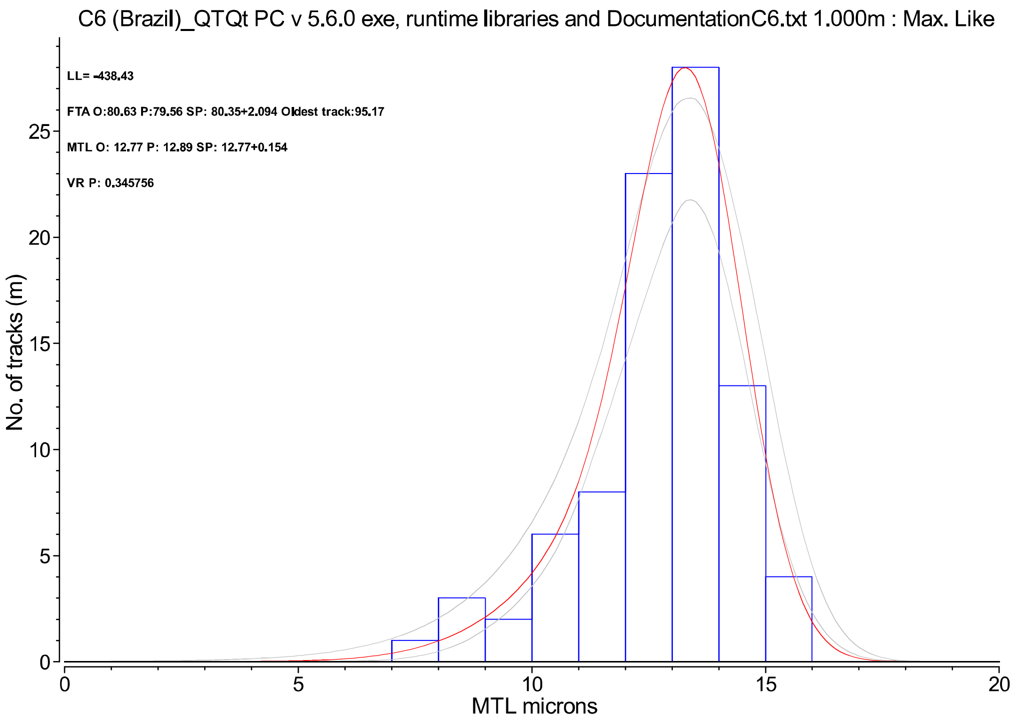
C6 (Brazil)_QTQt PC v 5.6.0 exe, runtime libraries and DocumentationC6.txt 1.000m : Max. Like
LL= -438.43
FTA O:80.63 P:79.56 SP: 80.35+2.094 Oldest track:95.17
MTL O: 12.77 P: 12.89 SP: 12.77+0.154
VR P: 0.345756
No. of tracks (m)
MTL microns

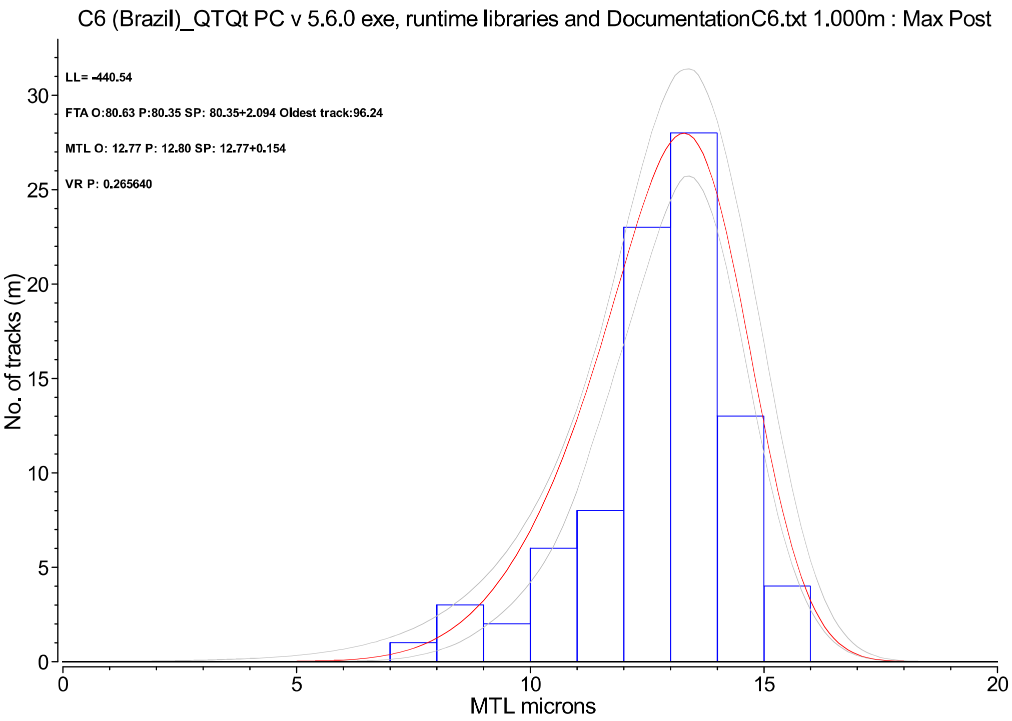
C6 (Brazil)_QTQt PC v 5.6.0 exe, runtime libraries and DocumentationC6.txt 1.000m : Max Post
LL= -440.54
FTA O:80.63 P:80.35 SP: 80.35+2.094 Oldest track:96.24
MTL O: 12.77 P: 12.80 SP: 12.77+0.154
VR P: 0.265640
No. of tracks (m)
MTL microns

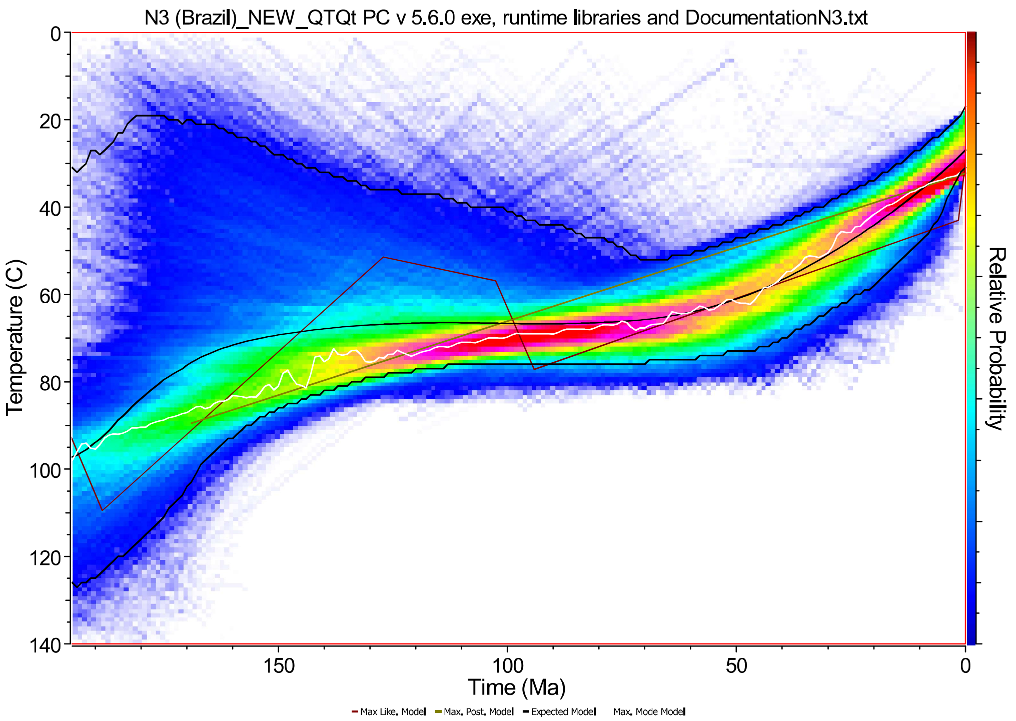
N3 (Brazil)_NEW_QTQt PC v 5.6.0 exe, runtime libraries and DocumentationN3.txt
Temperature (C)
Time (Ma)
Relative Probability
Max Like. Model
Max. Post. Model
Expected Model
Max. Mode Model

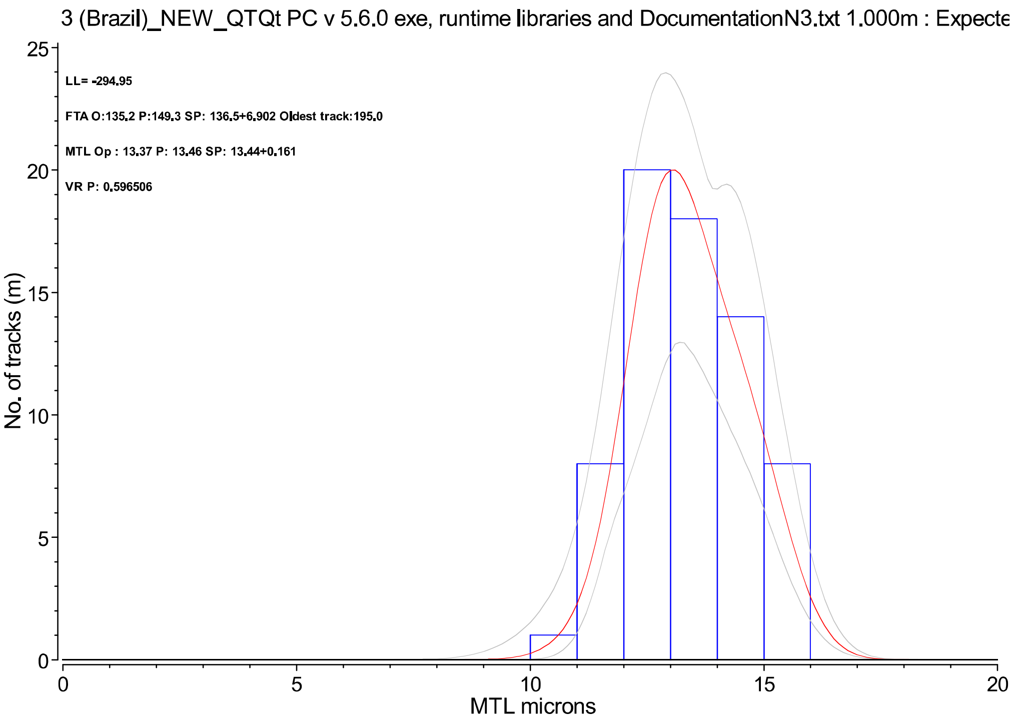
3 (Brazil)_NEW_QTQt PC v 5.6.0 exe, runtime libraries and DocumentationN3.txt 1.000m : Expecte
LL= -294.95
FTA O:135.2 P:149.3 SP: 136.5+6.902 Oldest track:195.0
MTL Op : 13.37 P: 13.46 SP: 13.44+0.161
VR P: 0.596506
No. of tracks (m)
MTL microns

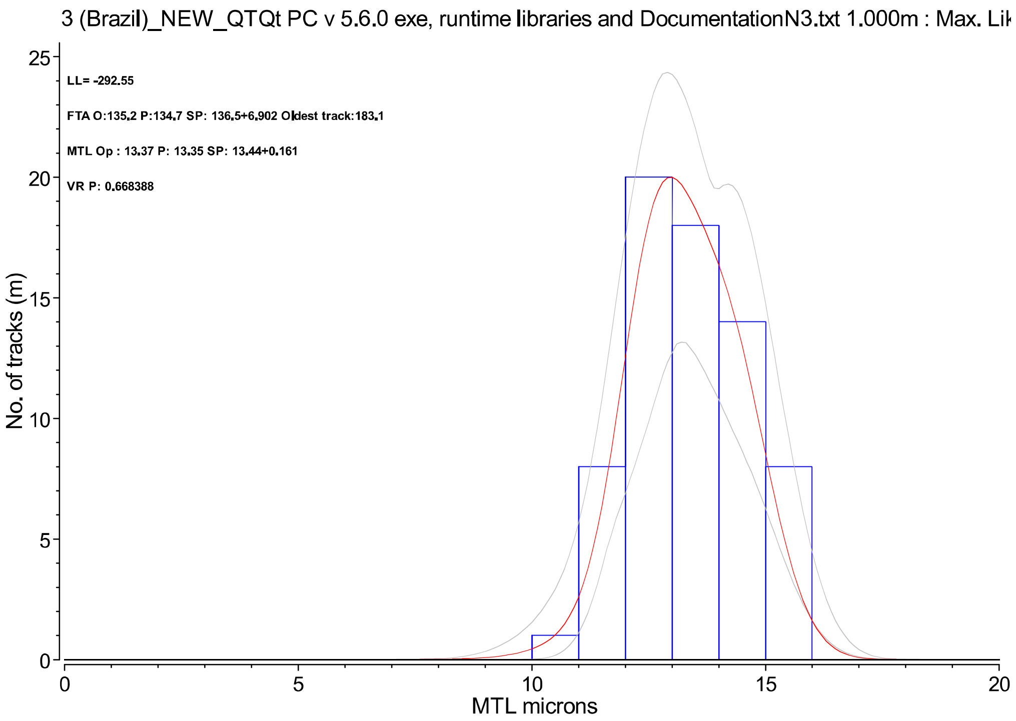
3 (Brazil)_NEW_QTQt PC v 5.6.0 exe, runtime libraries and DocumentationN3.txt 1.000m : Max. Lik
LL= -292.55
FTA O:135.2 P:134.7 SP: 136.5+6.902 Oldest track:183.1
MTL Op : 13.37 P: 13.35 SP: 13.44+0.161
VR P: 0.668388
No. of tracks (m)
MTL microns

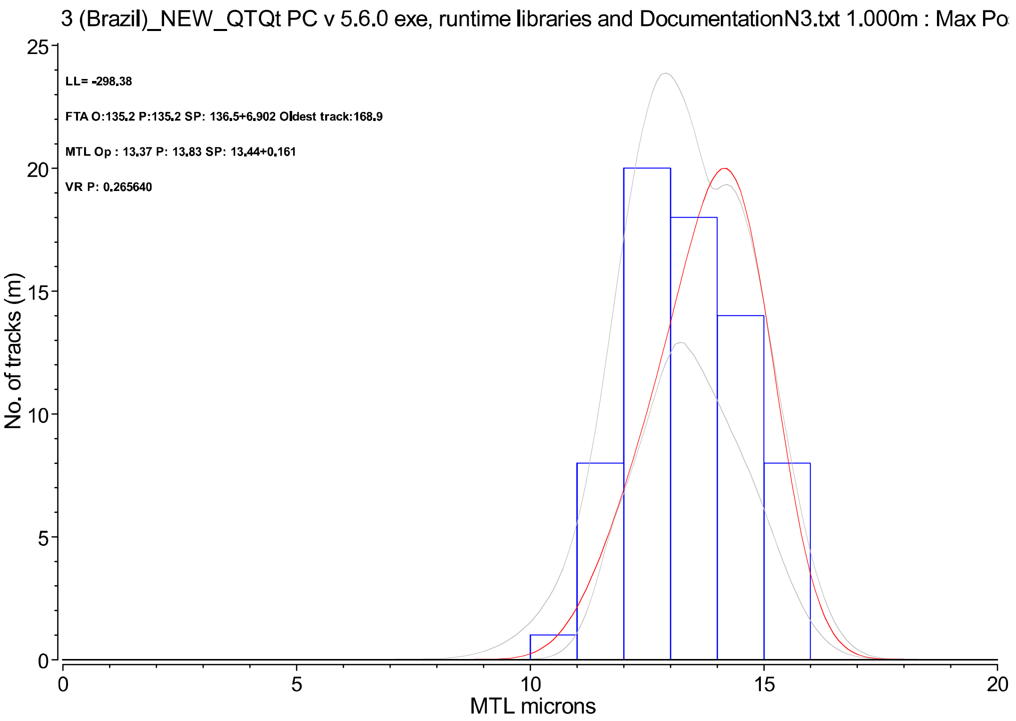
3 (Brazil)_NEW_QTQt PC v 5.6.0 exe, runtime libraries and DocumentationN3.txt 1.000m : Max Po
LL= -298.38
FTA O:135.2 P:135.2 SP: 136.5+6.902 Oldest track:168.9
MTL Op : 13.37 P: 13.83 SP: 13.44+0.161
VR P: 0.265640
No. of tracks (m)
MTL microns

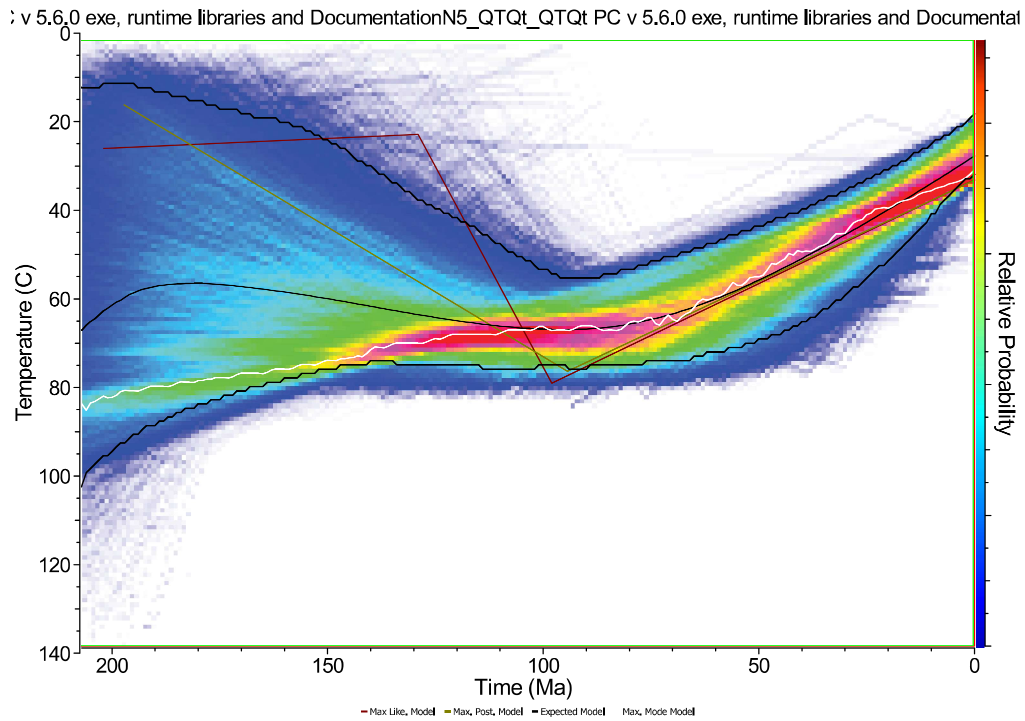

; v 5.6.0 exe, runtime libraries and DocumentationN5_QTQt_QTQt PC v 5.6.0 exe, runtime libraries and Documentat
Temperature (C)
Time (Ma)
Relative Probability
0
20
40
60
80
100
120
140
200
150
100
50
0
Max Like. Model
Max. Post. Model
Expected Model
Max. Mode Model

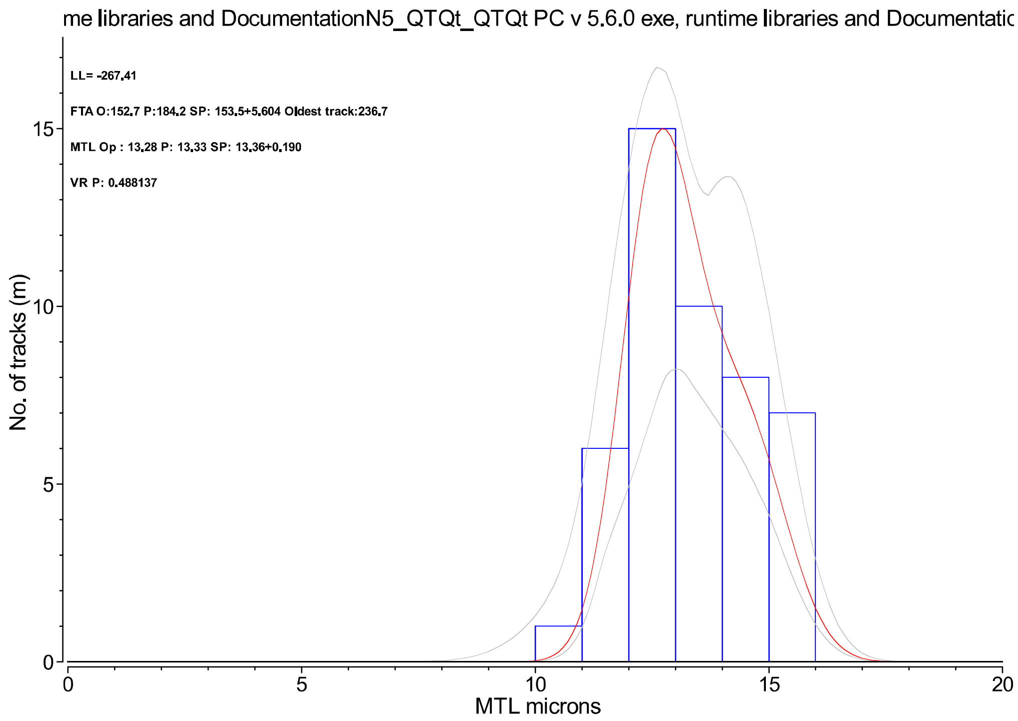

me libraries and DocumentationN5_QTQt_QTQt PC v 5.6.0 exe, runtime libraries and Documentatic
LL= -267.41
FTA O:152.7 P:184.2 SP: 153.5+5.604 Oldest track:236.7
MTL Op : 13.28 P: 13.33 SP: 13.36+0.190
VR P: 0.488137
No. of tracks (m)
MTL microns
0
5
10
15
20

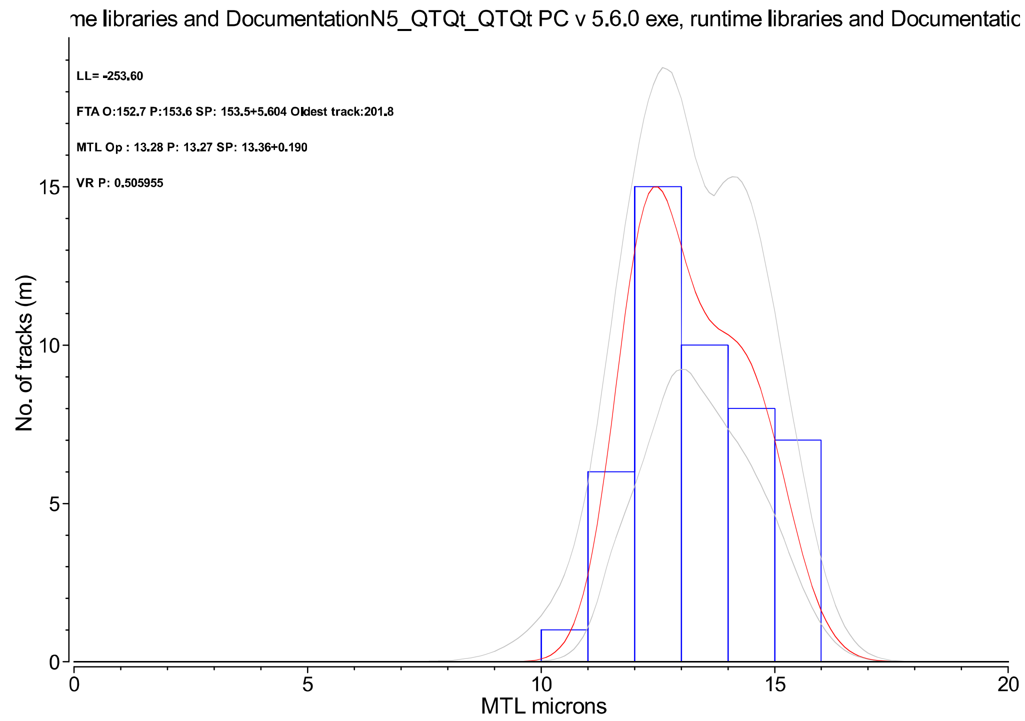

me libraries and DocumentationN5_QTQt_QTQt PC v 5.6.0 exe, runtime libraries and Documentatic
LL= -253.60
FTA O:152.7 P:153.6 SP: 153.5+5.604 Oldest track:201.8
MTL Op : 13.28 P: 13.27 SP: 13.36+0.190
VR P: 0.505955
No. of tracks (m)
MTL microns
0
5
10
15
20

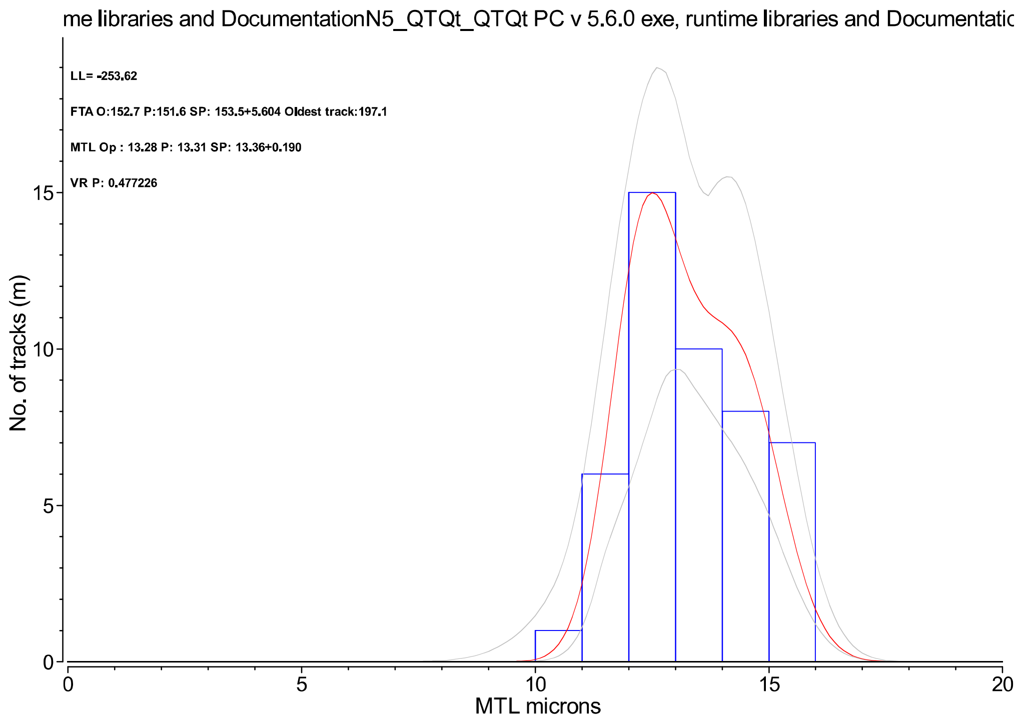

me libraries and DocumentationN5_QTQt_QTQt PC v 5.6.0 exe, runtime libraries and Documentatic
LL= -253.62
FTA O:152.7 P:151.6 SP: 153.5+5.604 Oldest track:197.1
MTL Op : 13.28 P: 13.31 SP: 13.36+0.190
VR P: 0.477226
No. of tracks (m)
MTL microns
0
5
10
15
20

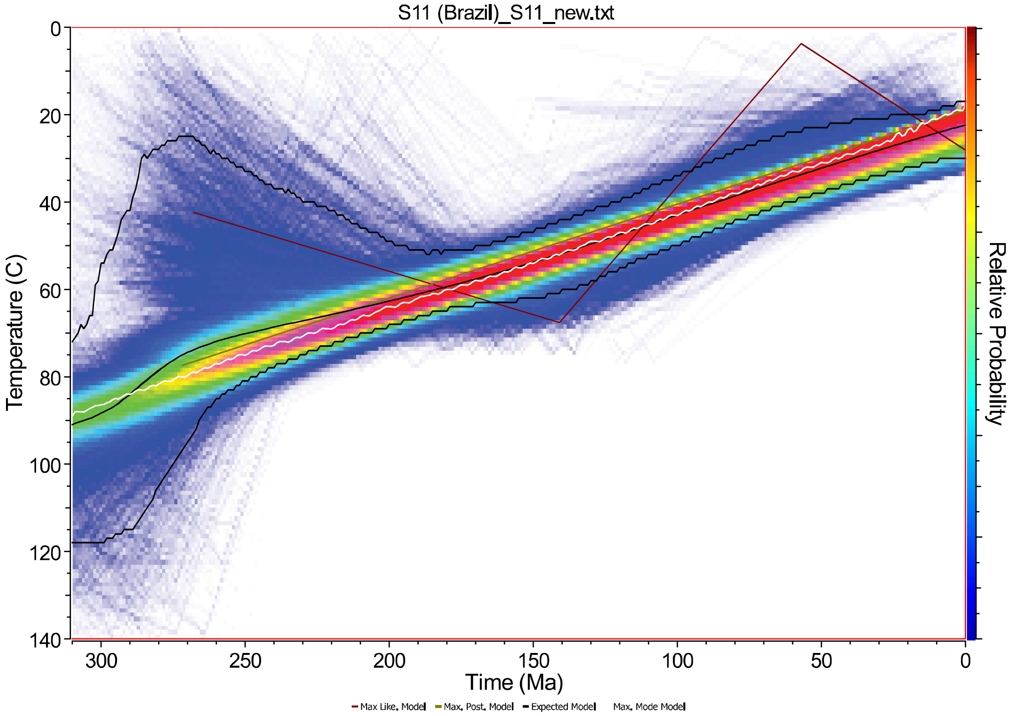
S11 (Brazil)_S11_new.txt
Temperature (C)
Time (Ma)
Relative Probability
Max Like. Model
Max. Post. Model
Expected Model
Max. Mode Model

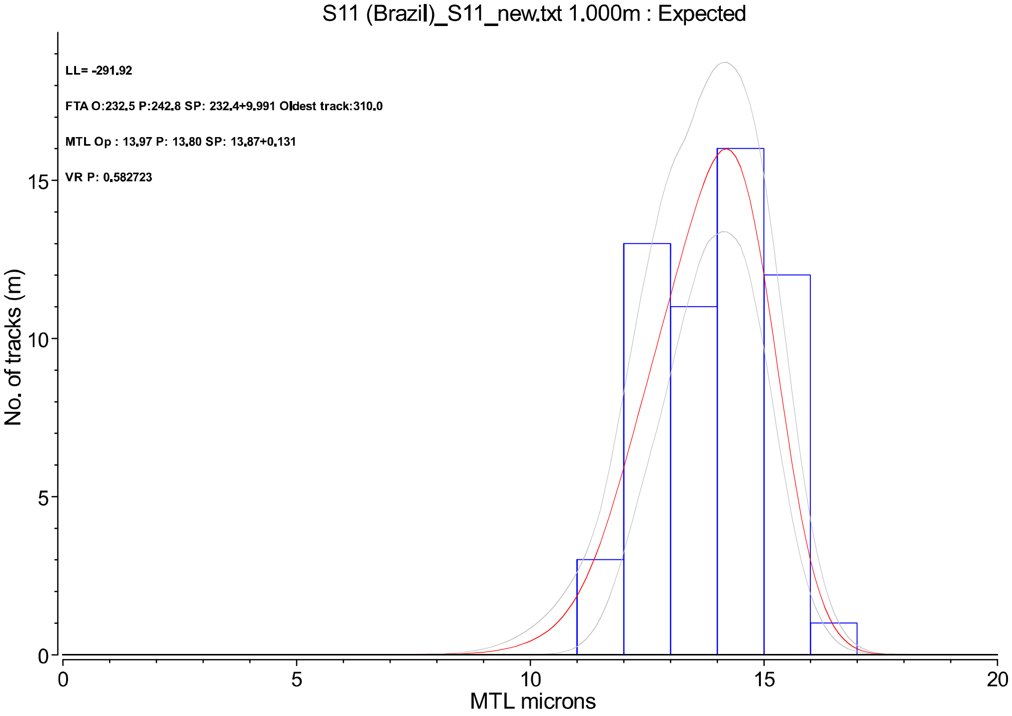
S11 (Brazil)_S11_new.txt 1.000m : Expected
LL= -291.92
FTA O:232.5 P:242.8 SP: 232.4+9.991 Oldest track:310.0
MTL Op : 13.97 P: 13.80 SP: 13.87+0.131
VR P: 0.582723
No. of tracks (m)
MTL microns

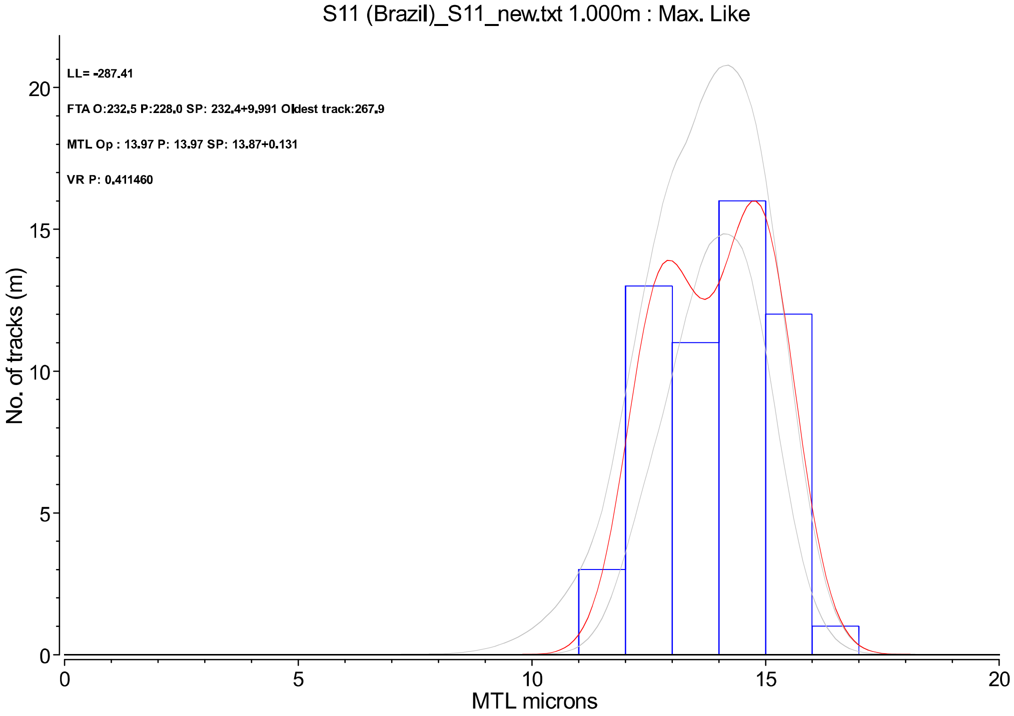
S11 (Brazil)_S11_new.txt 1.000m : Max. Like
LL= -287.41
FTA O:232.5 P:228.0 SP: 232.4+9.991 Oldest track:267.9
MTL Op : 13.97 P: 13.97 SP: 13.87+0.131
VR P: 0.411460
No. of tracks (m)
MTL microns

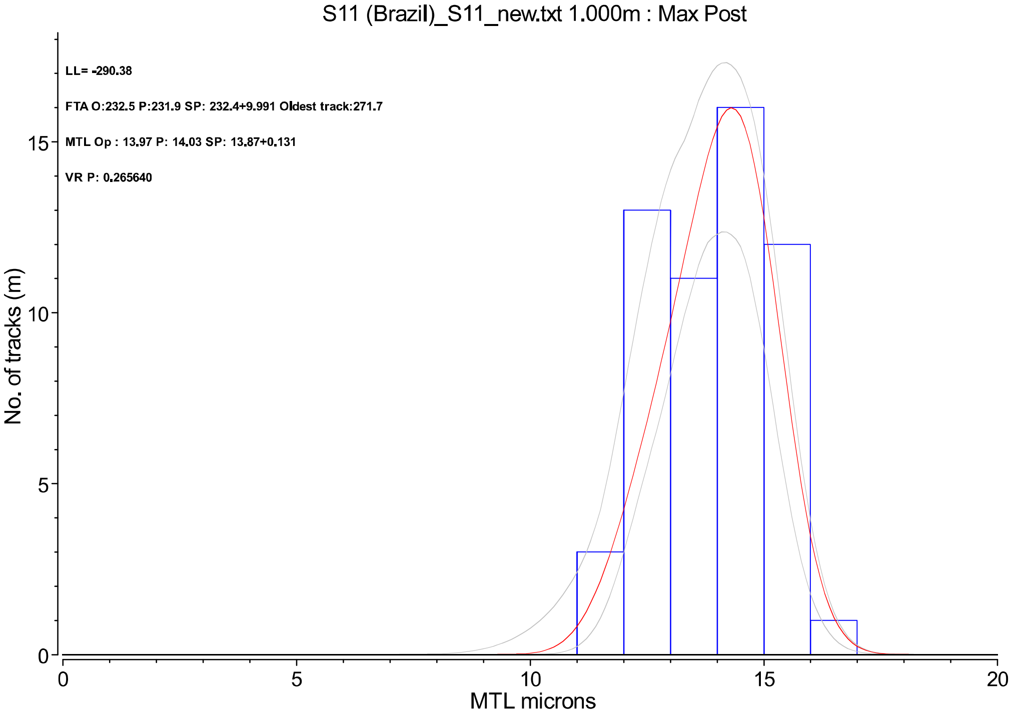
S11 (Brazil)_S11_new.txt 1.000m : Max Post
LL= -290.38
FTA O:232.5 P:231.9 SP: 232.4+9.991 Oldest track:271.7
MTL Op : 13.97 P: 14.03 SP: 13.87+0.131
VR P: 0.265640
No. of tracks (m)
MTL microns

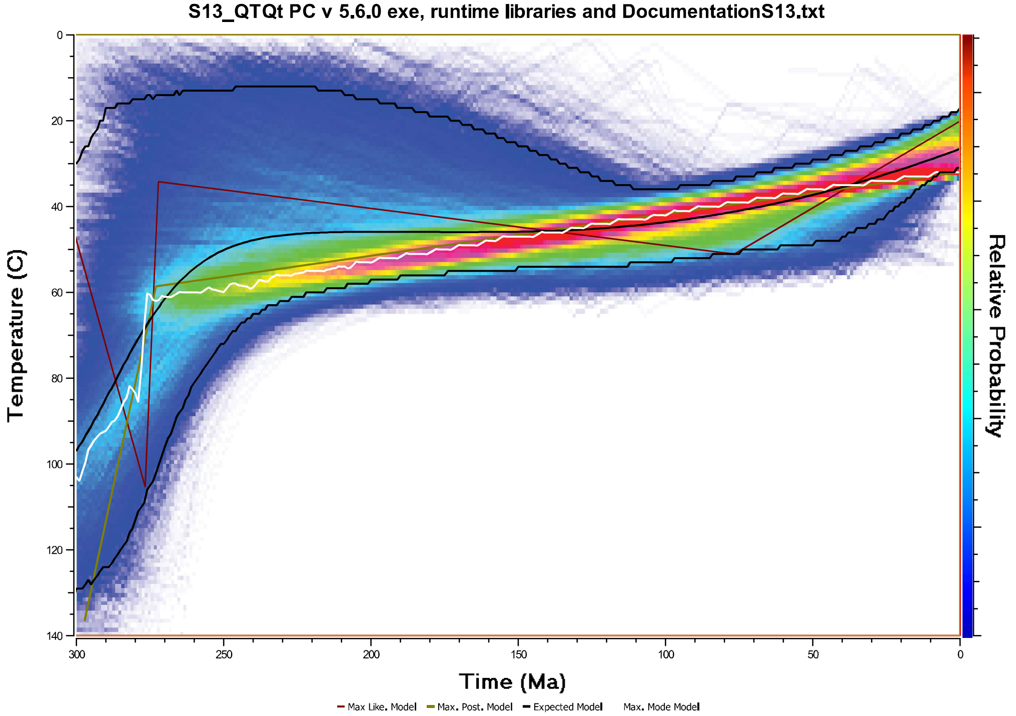
S13_QTQt PC v 5.6.0 exe, runtime libraries and DocumentationS13.txt
Temperature (C)
Time (Ma)
Relative Probability
Max Like. Model
Max. Post. Model
Expected Model
Max. Mode Model

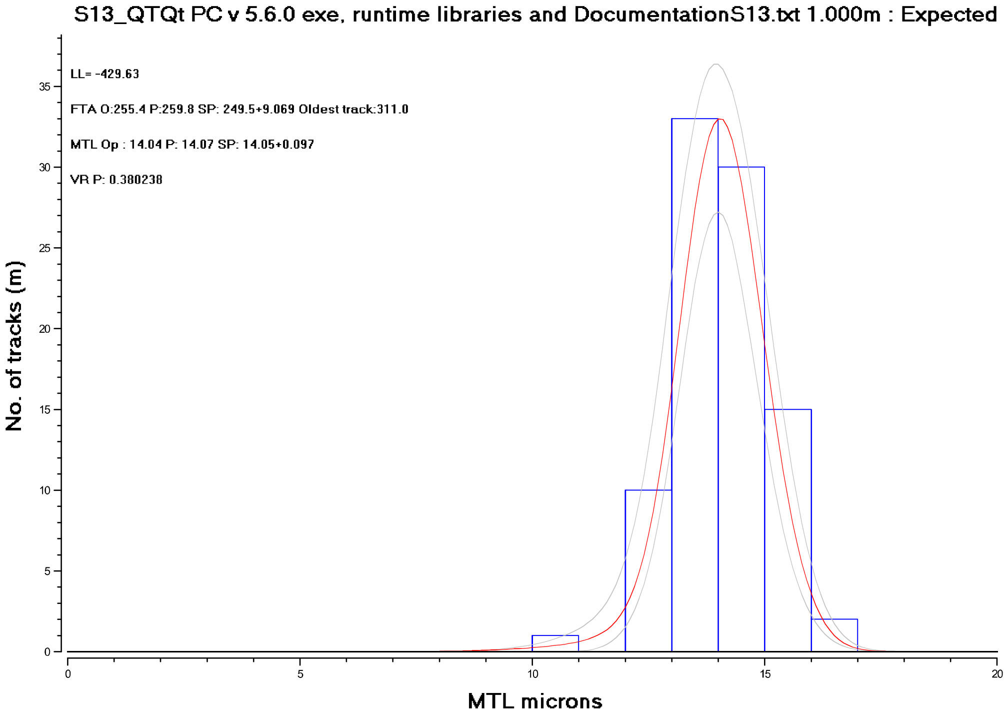
S13_QTQt PC v 5.6.0 exe, runtime libraries and DocumentationS13.txt 1.000m : Expected
LL= -429.63
FTA O:255.4 P:259.8 SP: 249.5+9.069 Oldest track:311.0
MTL Op : 14.04 P: 14.07 SP: 14.05+0.097
VR P: 0.380238
No. of tracks (m)
MTL microns

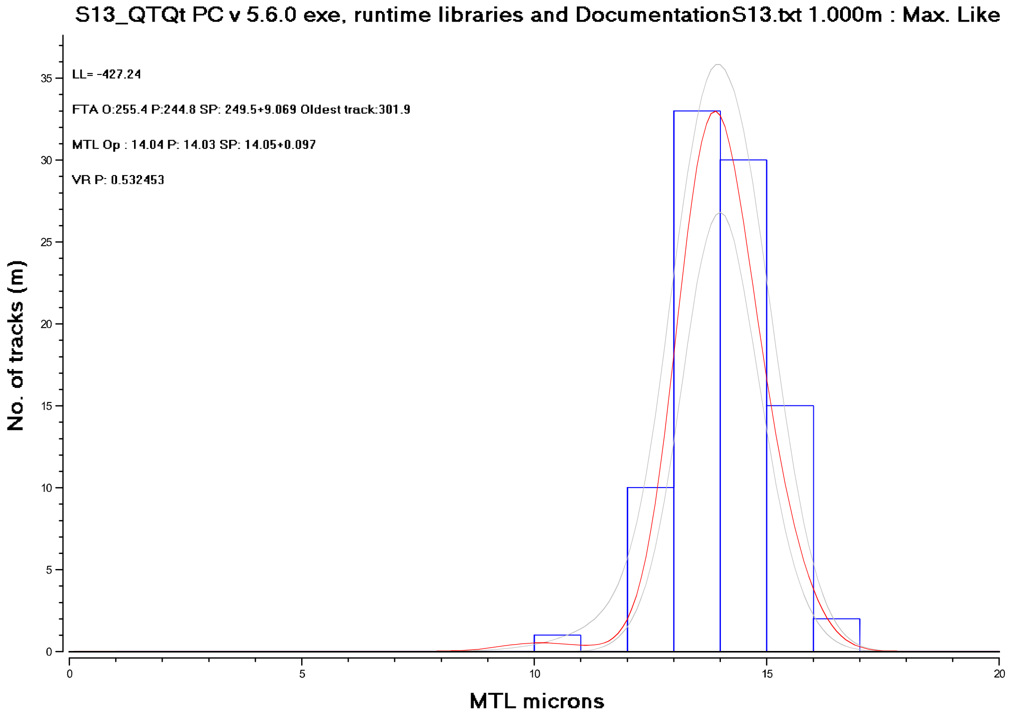
S13_QTQt PC v 5.6.0 exe, runtime libraries and DocumentationS13.txt 1.000m : Max. Like
LL= -427.24
FTA O:255.4 P:244.8 SP: 249.5+9.069 Oldest track:301.9
MTL Op : 14.04 P: 14.03 SP: 14.05+0.097
VR P: 0.532453
No. of tracks (m)
MTL microns

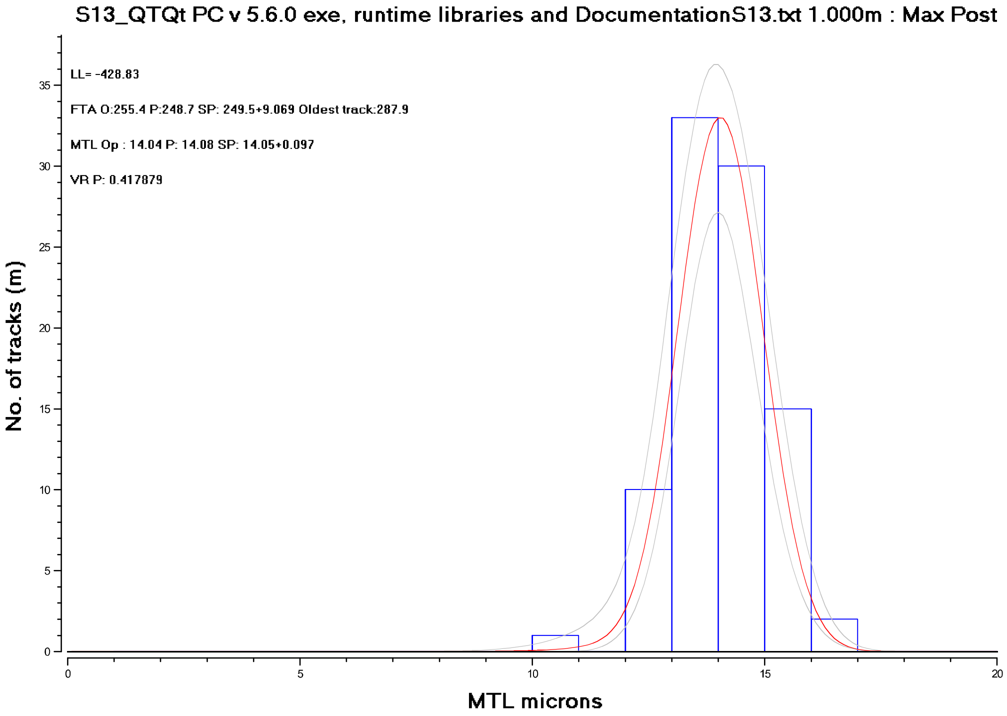
S13_QTQt PC v 5.6.0 exe, runtime libraries and DocumentationS13.txt 1.000m : Max Post
LL= -428.83
FTA O:255.4 P:248.7 SP: 249.5+9.069 Oldest track:287.9
MTL Op : 14.04 P: 14.08 SP: 14.05+0.097
VR P: 0.417879
No. of tracks (m)
MTL microns

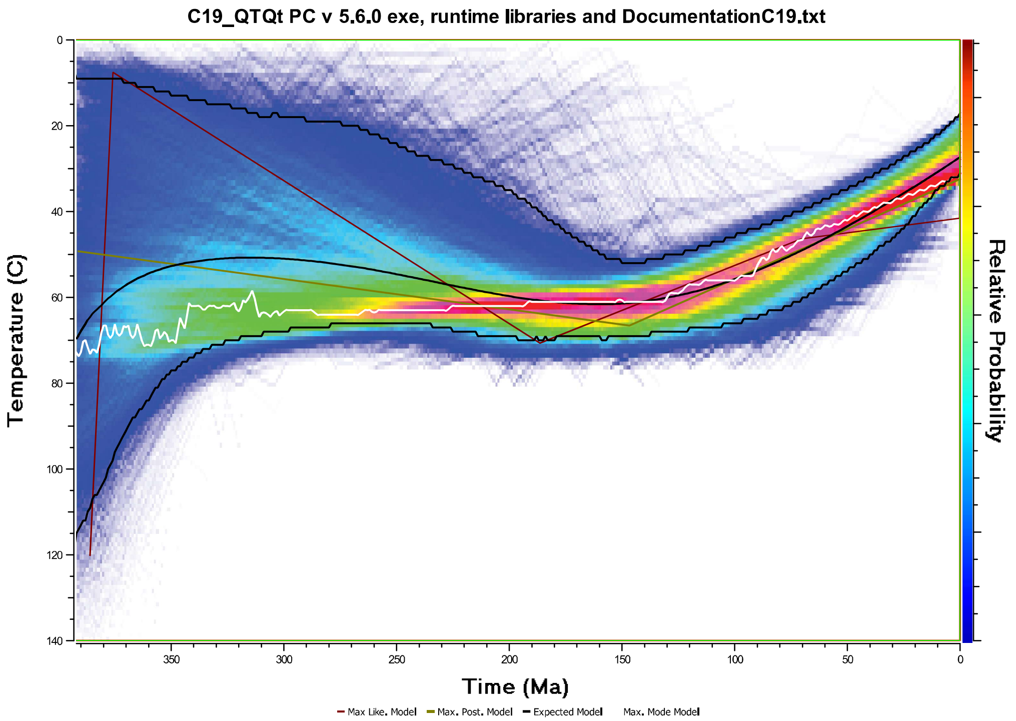
C19_QTQt PC v 5.6.0 exe, runtime libraries and DocumentationC19.txt
Temperature (C)
Time (Ma)
Relative Probability
Max Like. Model
Max. Post. Model
Expected Model
Max. Mode Model

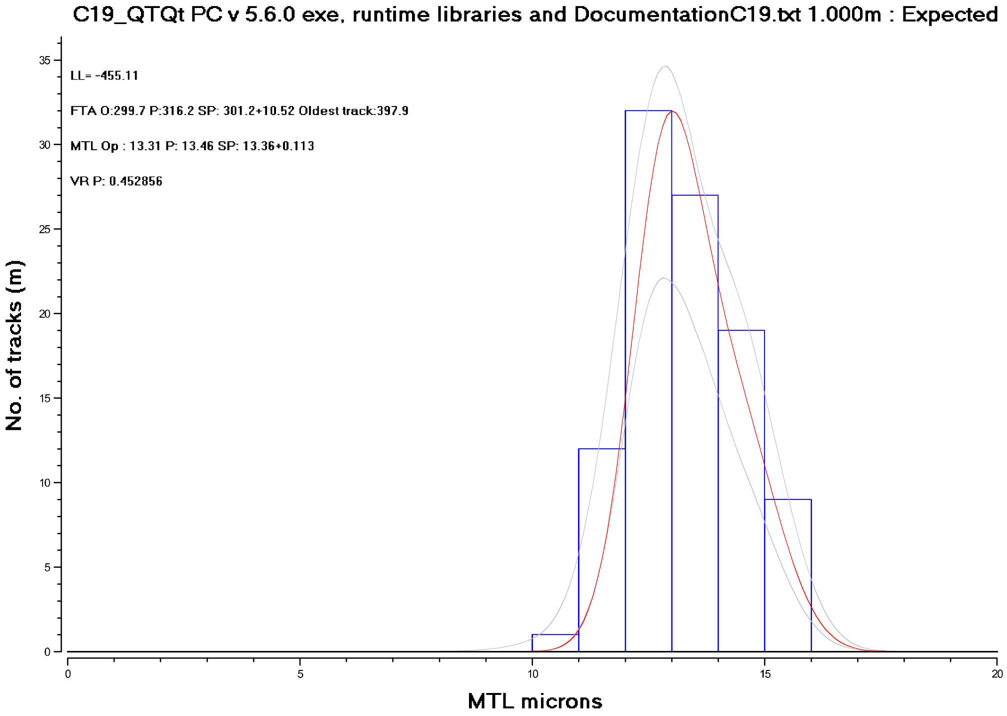
C19_QTQt PC v 5.6.0 exe, runtime libraries and DocumentationC19.txt 1.000m : Expected
LL= -455.11
FTA O:299.7 P:316.2 SP: 301.2+10.52 Oldest track:397.9
MTL Op : 13.31 P: 13.46 SP: 13.36+0.113
VR P: 0.452856
No. of tracks (m)
MTL microns

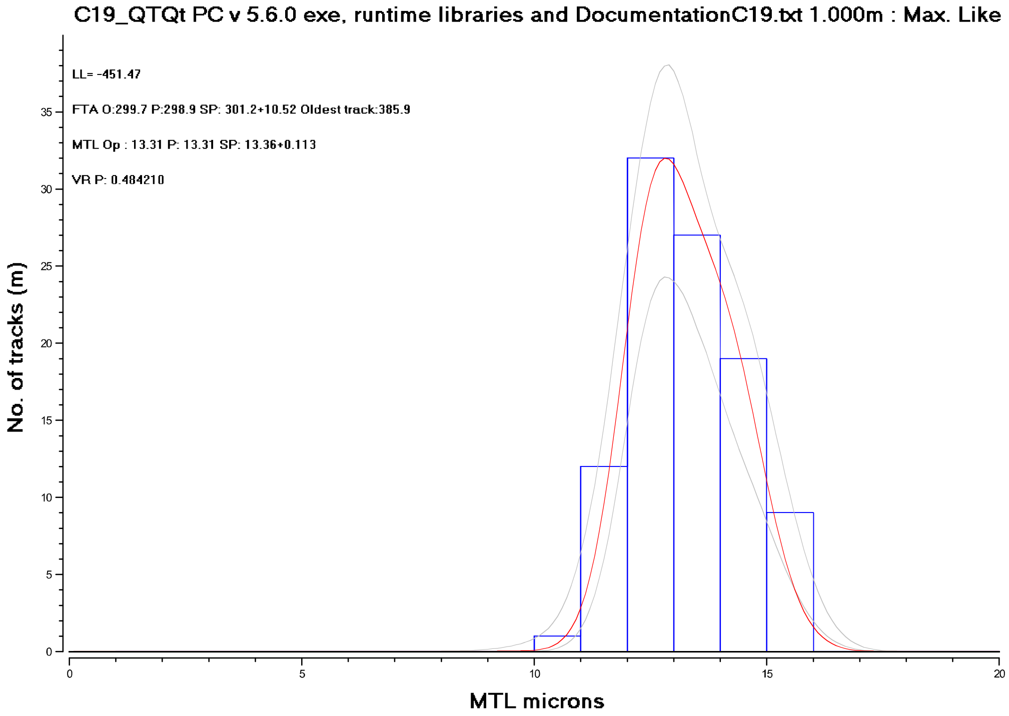
C19_QTQt PC v 5.6.0 exe, runtime libraries and DocumentationC19.txt 1.000m : Max. Like
LL= -451.47
FTA O:299.7 P:298.9 SP: 301.2+10.52 Oldest track:385.9
MTL Op : 13.31 P: 13.31 SP: 13.36+0.113
VR P: 0.484210
No. of tracks (m)
MTL microns

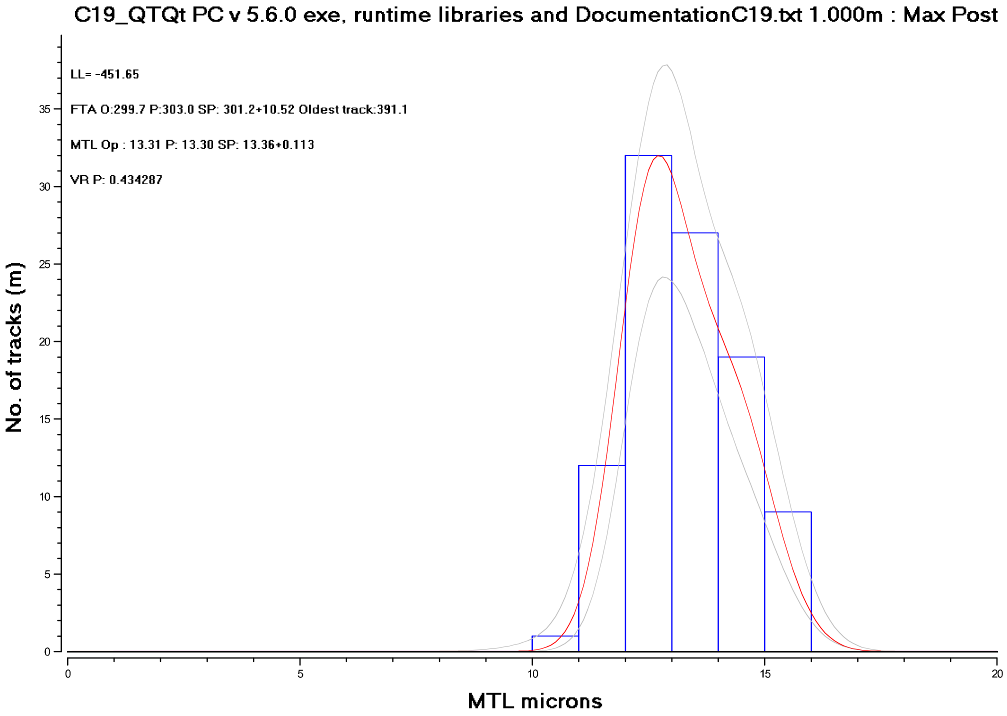
C19_QTQt PC v 5.6.0 exe, runtime libraries and DocumentationC19.txt 1.000m : Max Post
LL= -451.65
FTA O:299.7 P:303.0 SP: 301.2+10.52 Oldest track:391.1
MTL Op : 13.31 P: 13.30 SP: 13.36+0.113
VR P: 0.434287
No. of tracks (m)
MTL microns

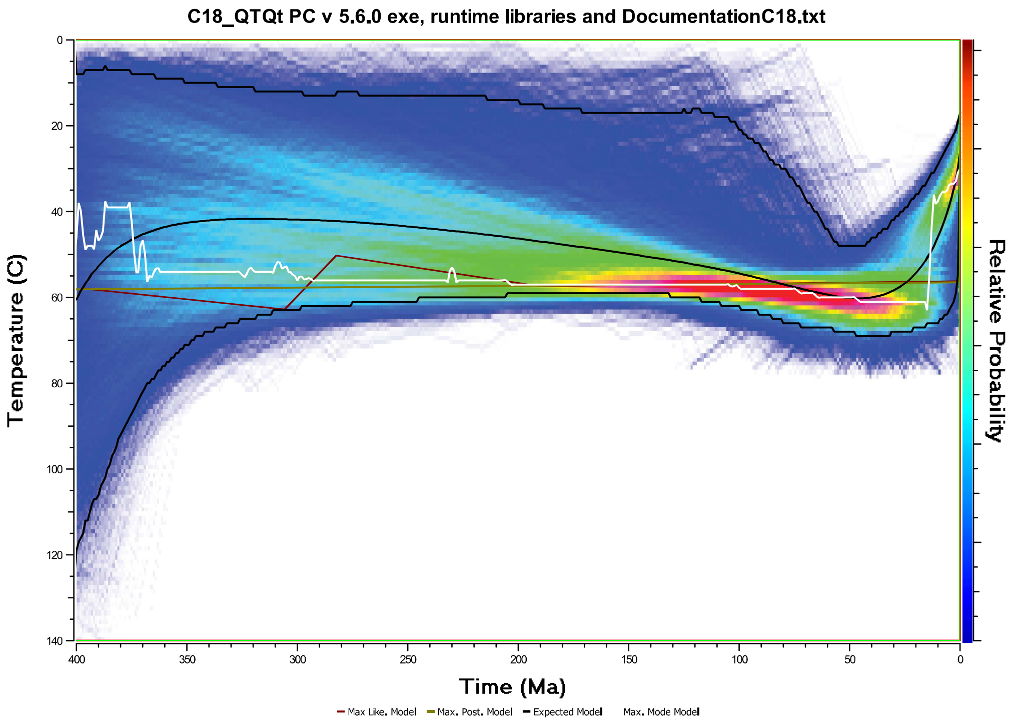
C18_QTQt PC v 5.6.0 exe, runtime libraries and DocumentationC18.txt
Temperature (C)
Time (Ma)
Relative Probability
Max Like. Model
Max. Post. Model
Expected Model
Max. Mode Model

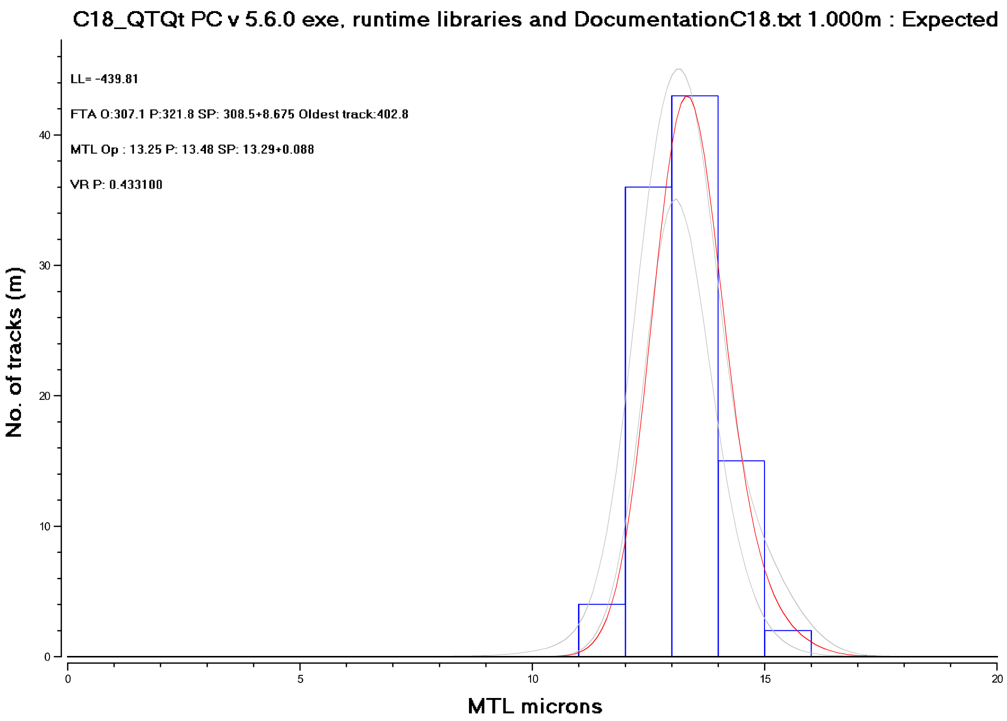
C18_QTQt PC v 5.6.0 exe, runtime libraries and DocumentationC18.txt 1.000m : Expected
LL= -439.81
FTA O:307.1 P:321.8 SP: 308.5+8.675 Oldest track:402.8
MTL Op : 13.25 P: 13.48 SP: 13.29+0.088
VR P: 0.433100
No. of tracks (m)
MTL microns

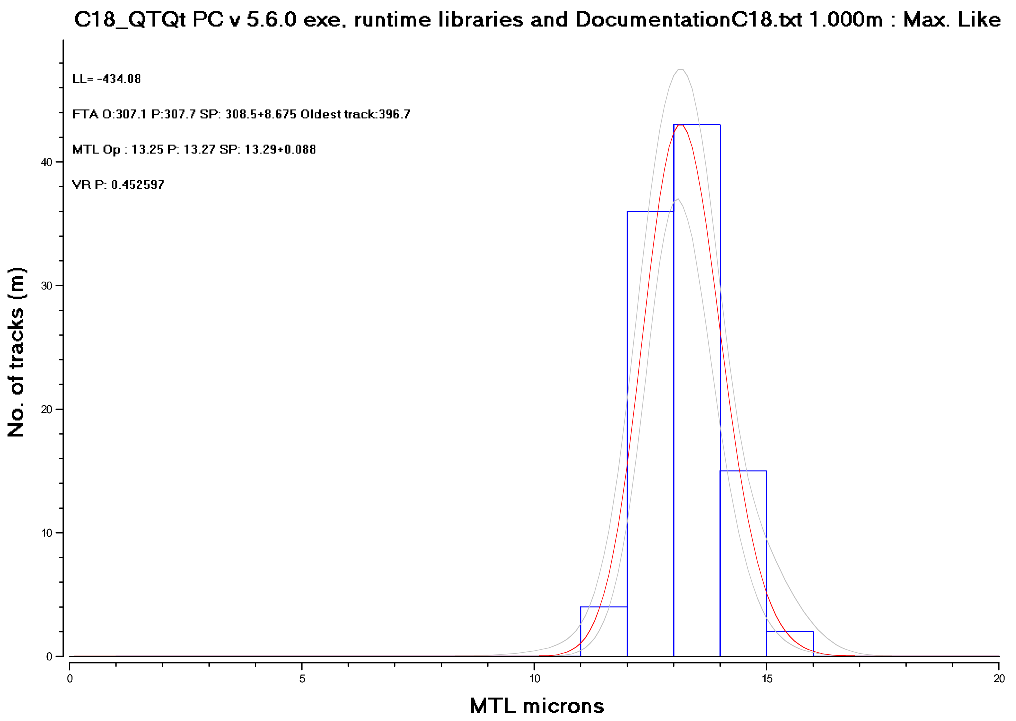
C18_QTQt PC v 5.6.0 exe, runtime libraries and DocumentationC18.txt 1.000m : Max. Like
LL= -434.08
FTA O:307.1 P:307.7 SP: 308.5+8.675 Oldest track:396.7
MTL Op : 13.25 P: 13.27 SP: 13.29+0.088
VR P: 0.452597
No. of tracks (m)
MTL microns

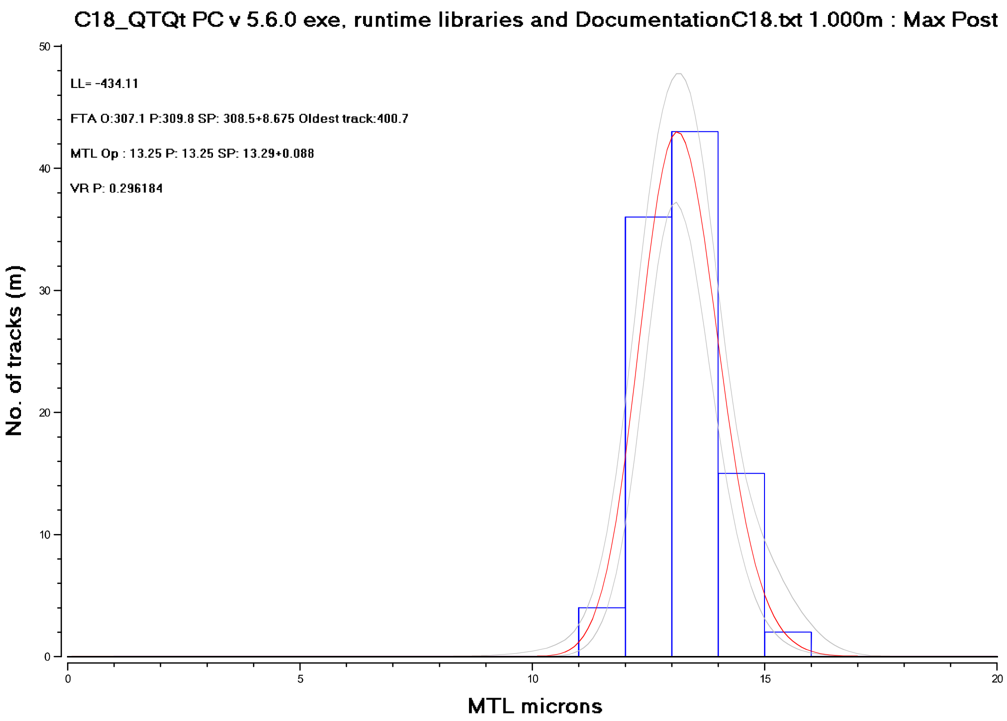
C18_QTQt PC v 5.6.0 exe, runtime libraries and DocumentationC18.txt 1.000m : Max Post
LL= -434.11
FTA O:307.1 P:309.8 SP: 308.5+8.675 Oldest track:400.7
MTL Op : 13.25 P: 13.25 SP: 13.29+0.088
VR P: 0.296184
No. of tracks (m)
MTL microns

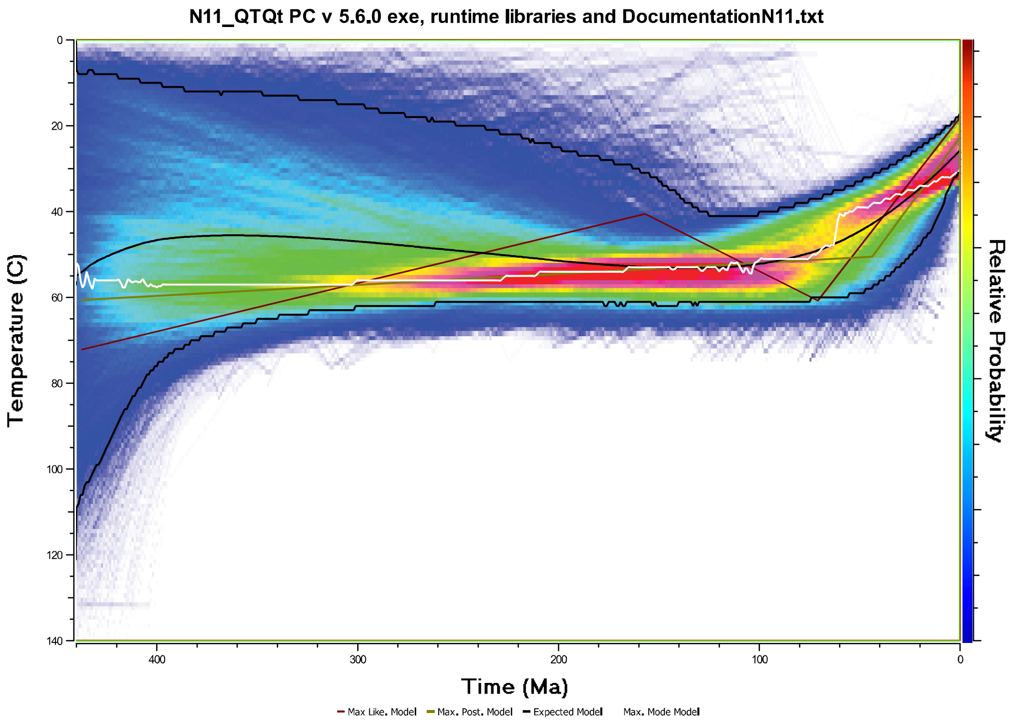

N11_QTQt PC v 5.6.0 exe, runtime libraries and DocumentationN11.txt
Temperature (C)
Time (Ma)
Relative Probability
Max Like. Model
Max. Post. Model
Expected Model
Max. Mode Model

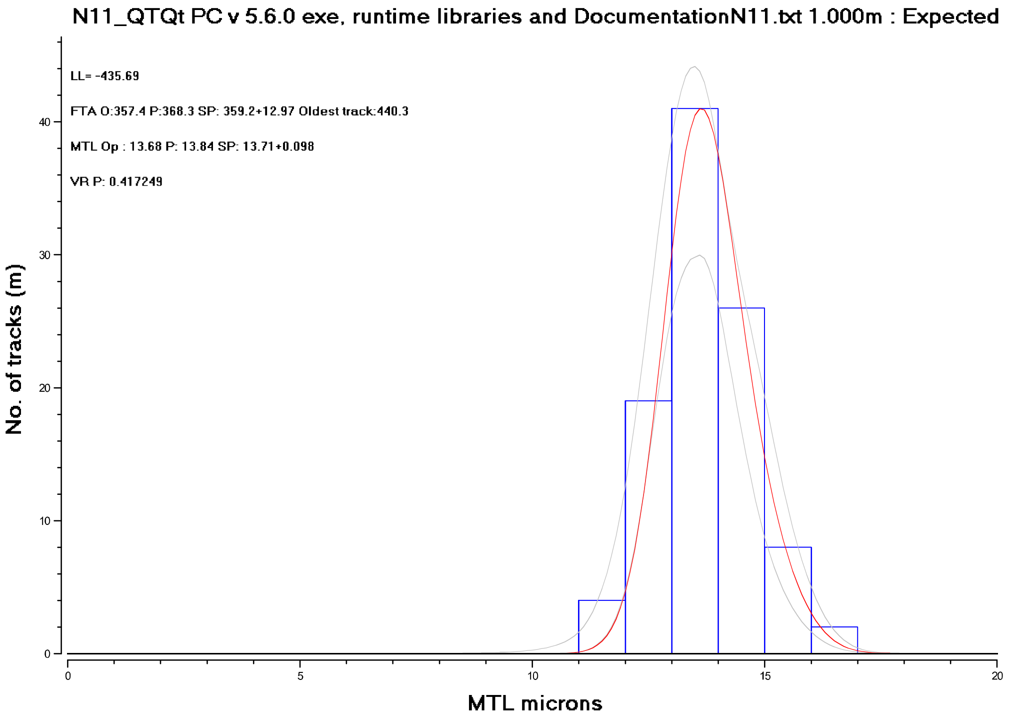

N11_QTQt PC v 5.6.0 exe, runtime libraries and DocumentationN11.txt 1.000m : Expected
LL= -435.69
FTA O:357.4 P:368.3 SP: 359.2+12.97 Oldest track:440.3
MTL Op : 13.68 P: 13.84 SP: 13.71+0.098
VR P: 0.417249
No. of tracks (m)
MTL microns

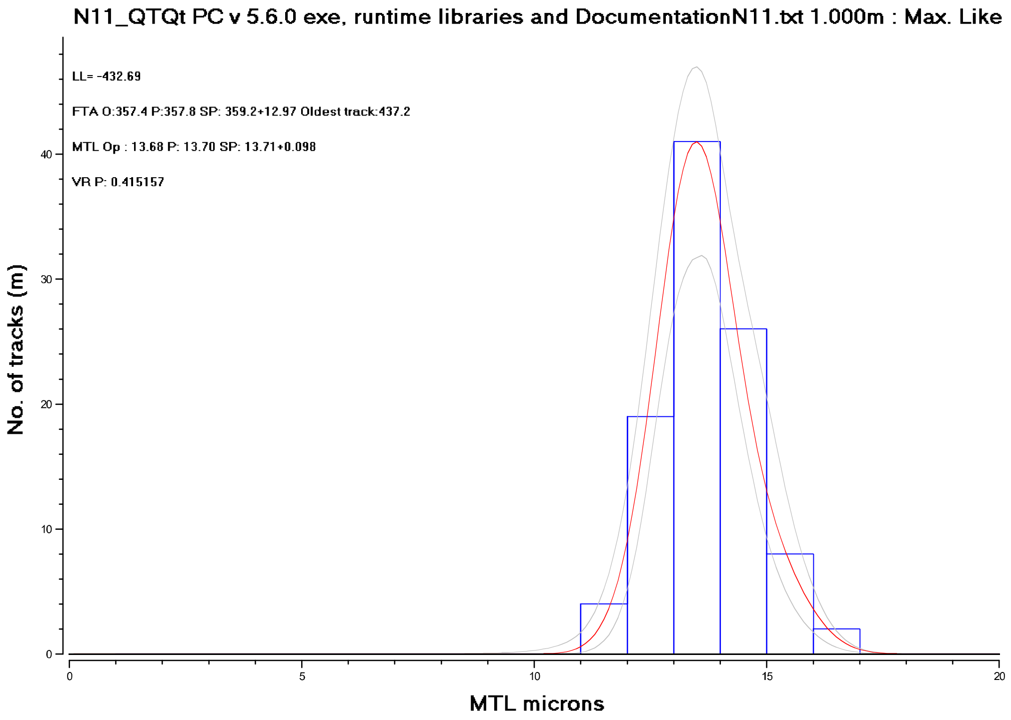

N11_QTQt PC v 5.6.0 exe, runtime libraries and DocumentationN11.txt 1.000m : Max. Like
LL= -432.69
FTA O:357.4 P:357.8 SP: 359.2+12.97 Oldest track:437.2
MTL Op : 13.68 P: 13.70 SP: 13.71+0.098
VR P: 0.415157
No. of tracks (m)
MTL microns

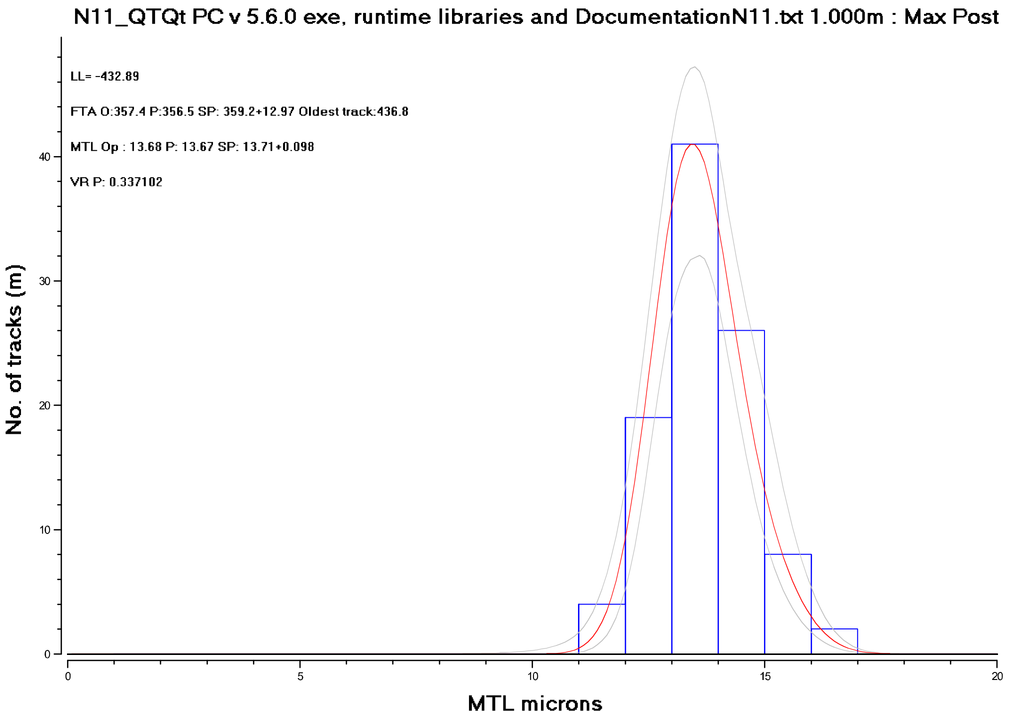

N11_QTQt PC v 5.6.0 exe, runtime libraries and DocumentationN11.txt 1.000m : Max Post
LL= -432.89
FTA O:357.4 P:356.5 SP: 359.2+12.97 Oldest track:436.8
MTL Op : 13.68 P: 13.67 SP: 13.71+0.098
VR P: 0.337102
No. of tracks (m)
MTL microns

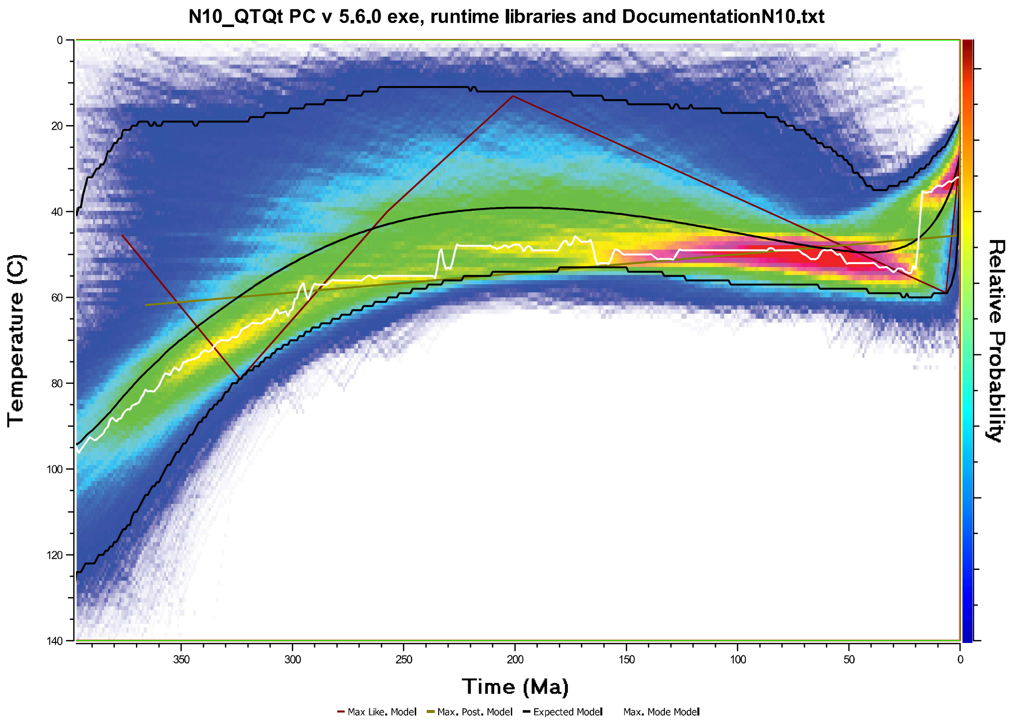
N10_QTQt PC v 5.6.0 exe, runtime libraries and DocumentationN10.txt
Temperature (C)
Time (Ma)
Relative Probability
Max Like. Model
Max. Post. Model
Expected Model
Max. Mode Model

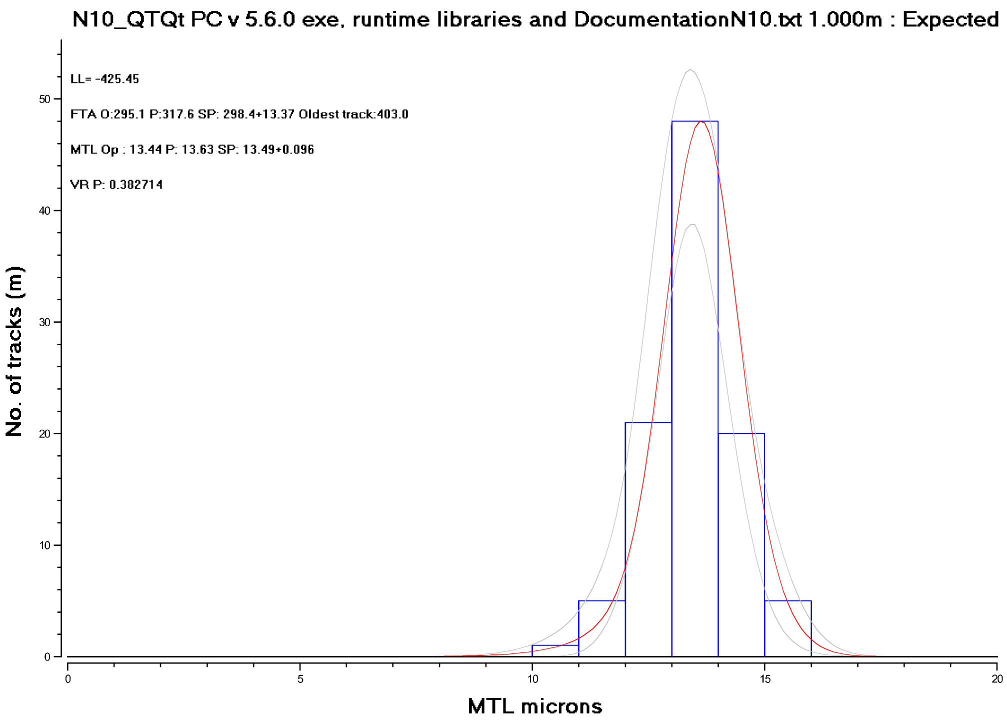
N10_QTQt PC v 5.6.0 exe, runtime libraries and DocumentationN10.txt 1.000m : Expected
LL= -425.45
FTA O:295.1 P:317.6 SP: 298.4+13.37 Oldest track:403.0
MTL Op : 13.44 P: 13.63 SP: 13.49+0.096
VR P: 0.382714
No. of tracks (m)
MTL microns

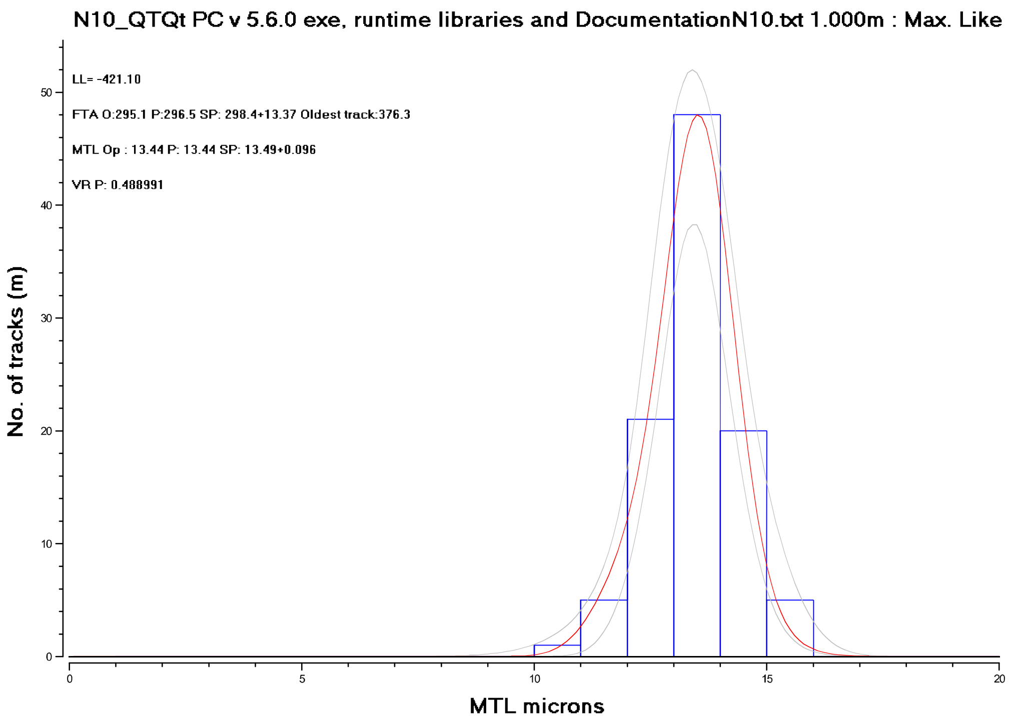
N10_QTQt PC v 5.6.0 exe, runtime libraries and DocumentationN10.txt 1.000m : Max. Like
LL= -421.10
FTA O:295.1 P:296.5 SP: 298.4+13.37 Oldest track:376.3
MTL Op : 13.44 P: 13.44 SP: 13.49+0.096
VR P: 0.488991
No. of tracks (m)
MTL microns

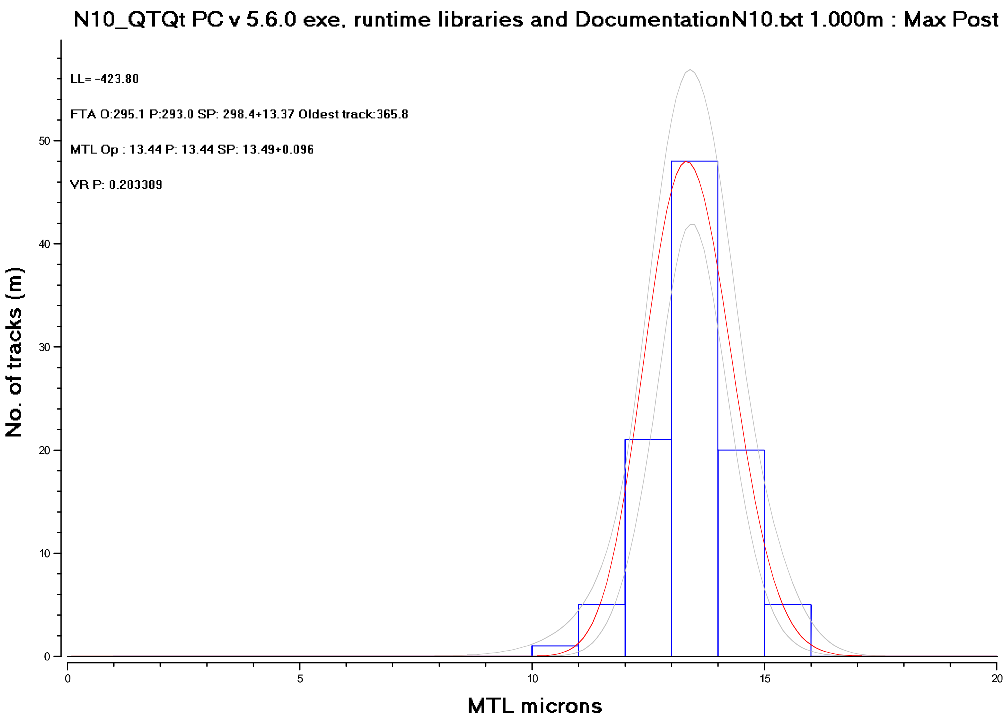
N10_QTQt PC v 5.6.0 exe, runtime libraries and DocumentationN10.txt 1.000m : Max Post
LL= -423.80
FTA O:295.1 P:293.0 SP: 298.4+13.37 Oldest track:365.8
MTL Op : 13.44 P: 13.44 SP: 13.49+0.096
VR P: 0.283389
No. of tracks (m)
MTL microns

8_QTQt PC v 5.6.0 exe, runtime libraries and Documentation8.txt

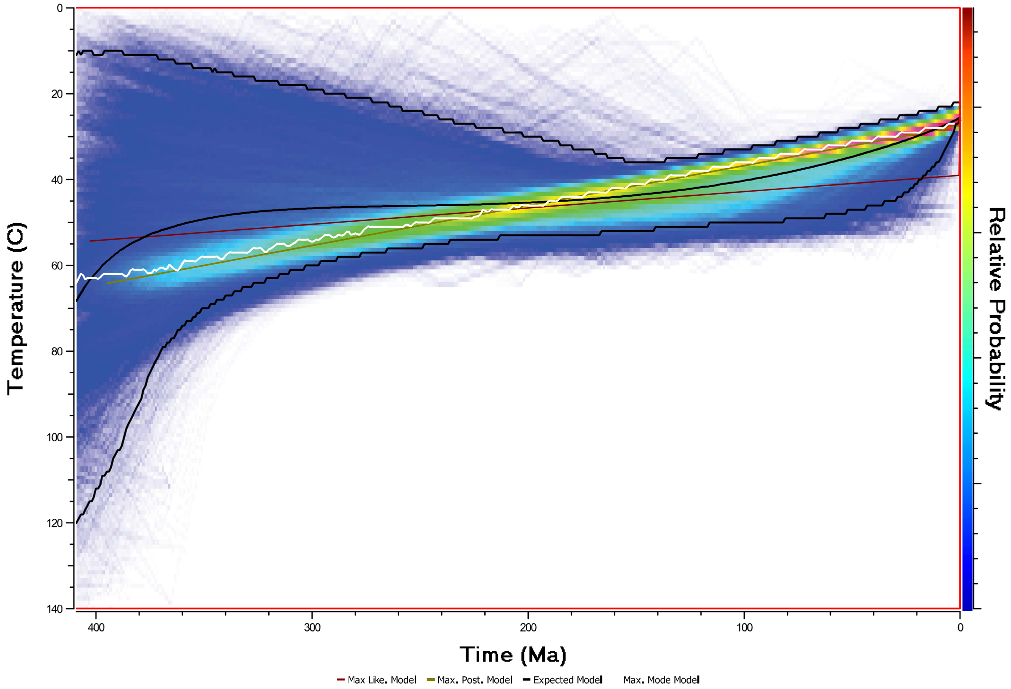

8_QTQt PC v 5.6.0 exe, runtime libraries and Documentation8.txt 3.000m : Expected

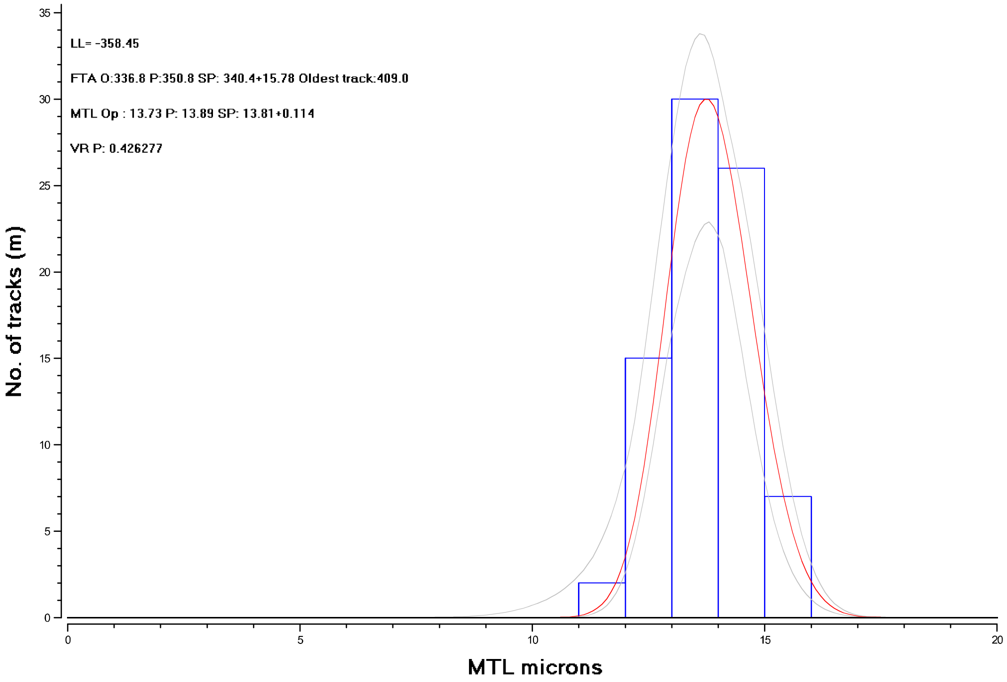

8_QTQt PC v 5.6.0 exe, runtime libraries and Documentation8.txt 3.000m : Max. Like

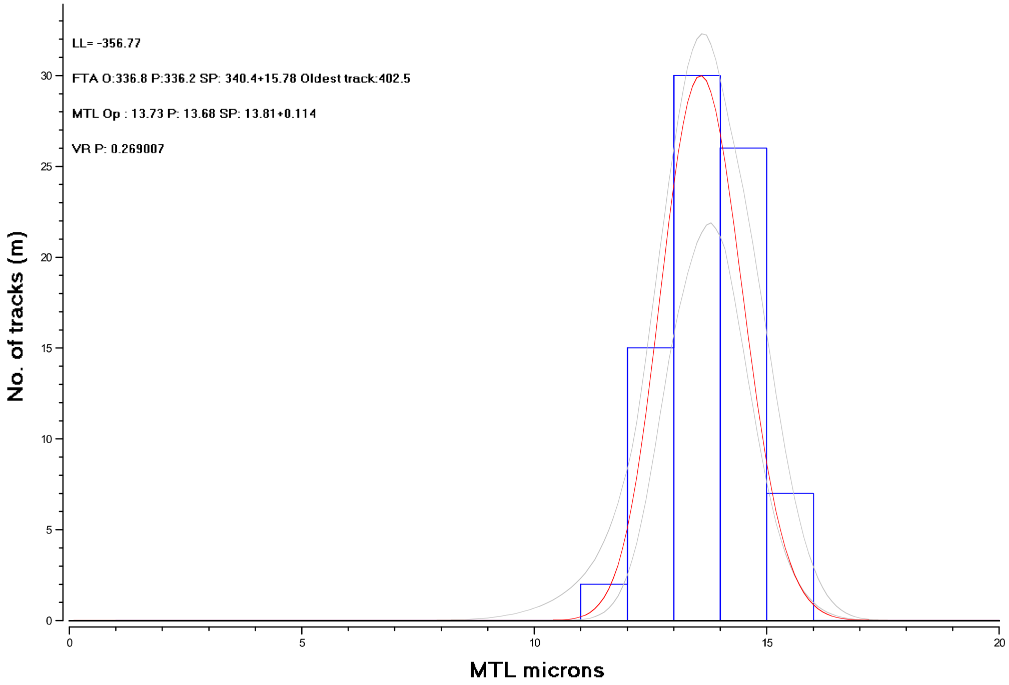

8_QTQt PC v 5.6.0 exe, runtime libraries and Documentation8.txt 3.000m : Max Post

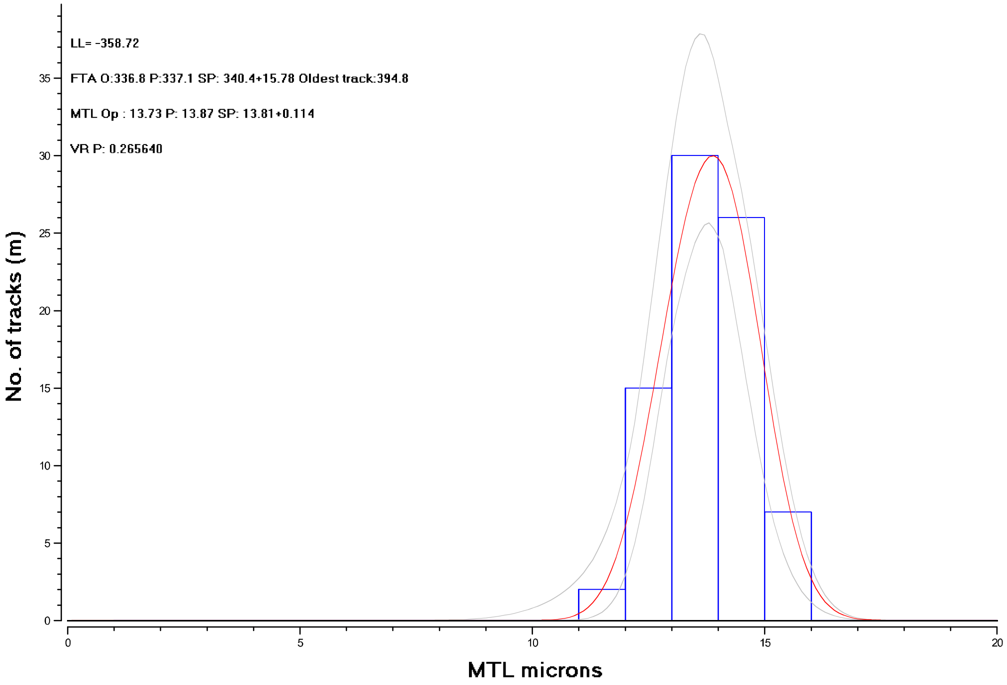

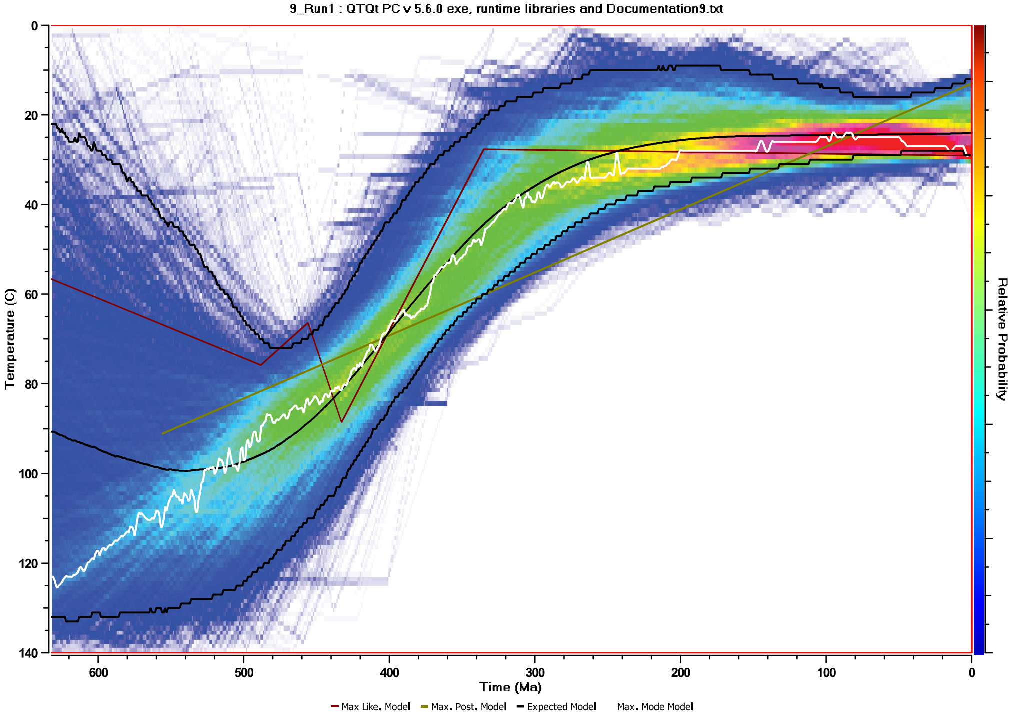
9_Run1 : QTQt PC v 5.6.0 exe, runtime libraries and Documentation9.txt
Temperature (C)
Time (Ma)
Relative Probability
Max Like. Model
Max. Post. Model
Expected Model
Max. Mode Model

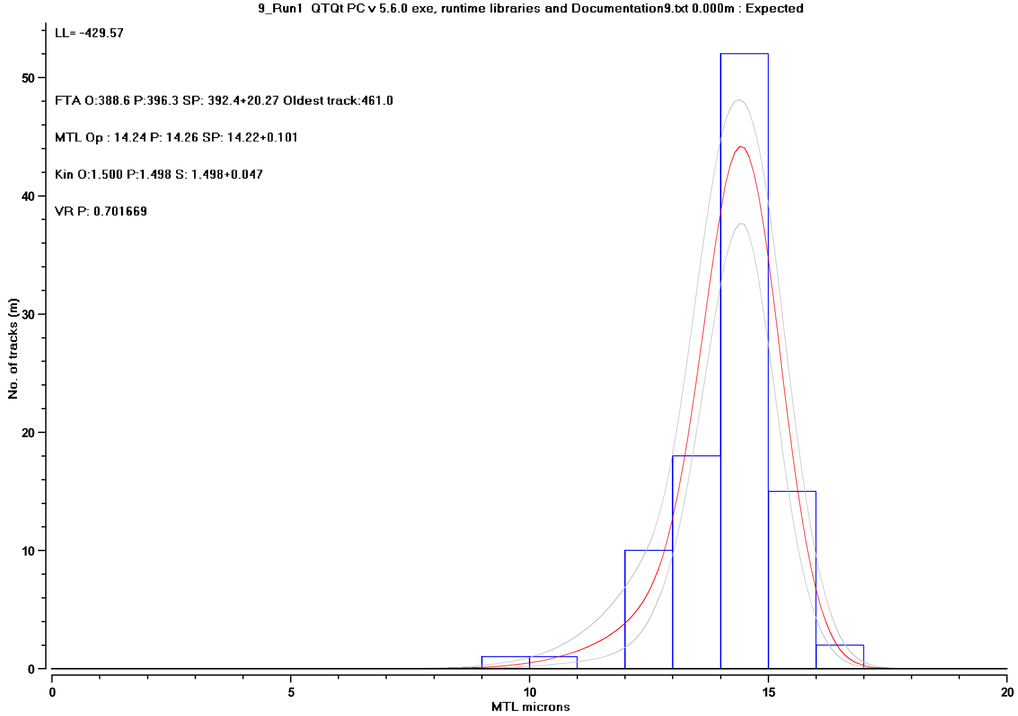
9_Run1 QTQt PC v 5.6.0 exe, runtime libraries and Documentation9.txt 0.000m : Expected
LL= -429.57
FTA O:388.6 P:396.3 SP: 392.4+20.27 Oldest track:461.0
MTL Op : 14.24 P: 14.26 SP: 14.22+0.101
Kin O:1.500 P:1.498 S: 1.498+0.047
VR P: 0.701669
No. of tracks (m)
MTL microns

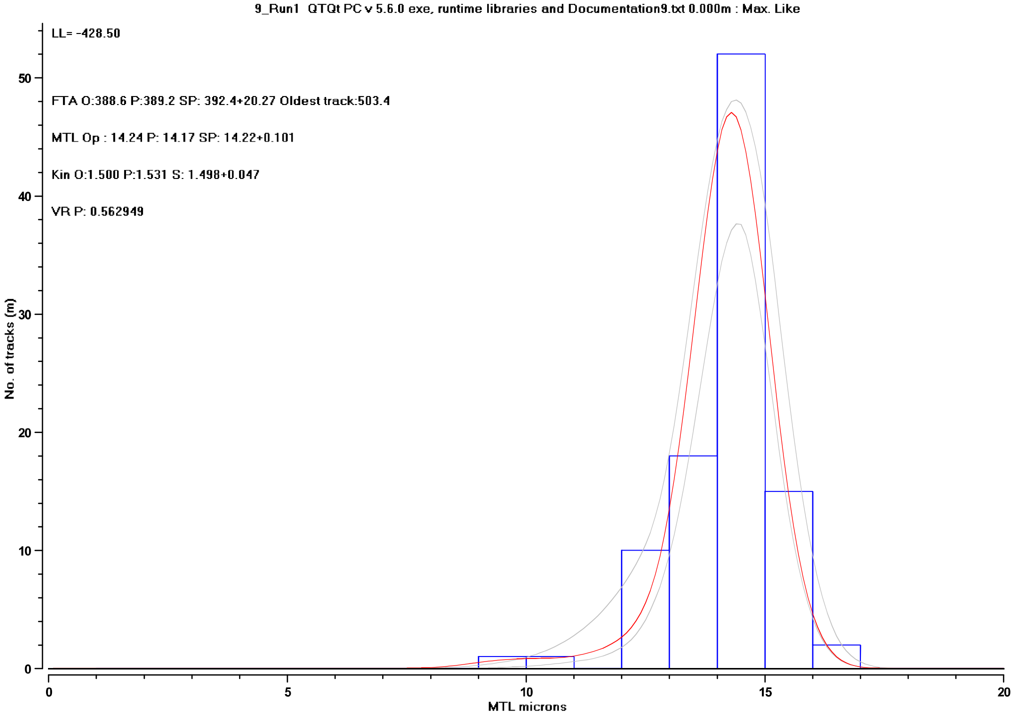
9_Run1 QTQt PC v 5.6.0 exe, runtime libraries and Documentation9.txt 0.000m : Max. Like
LL= -428.50
FTA O:388.6 P:389.2 SP: 392.4+20.27 Oldest track:503.4
MTL Op : 14.24 P: 14.17 SP: 14.22+0.101
Kin O:1.500 P:1.531 S: 1.498+0.047
VR P: 0.562949
No. of tracks (m)
MTL microns

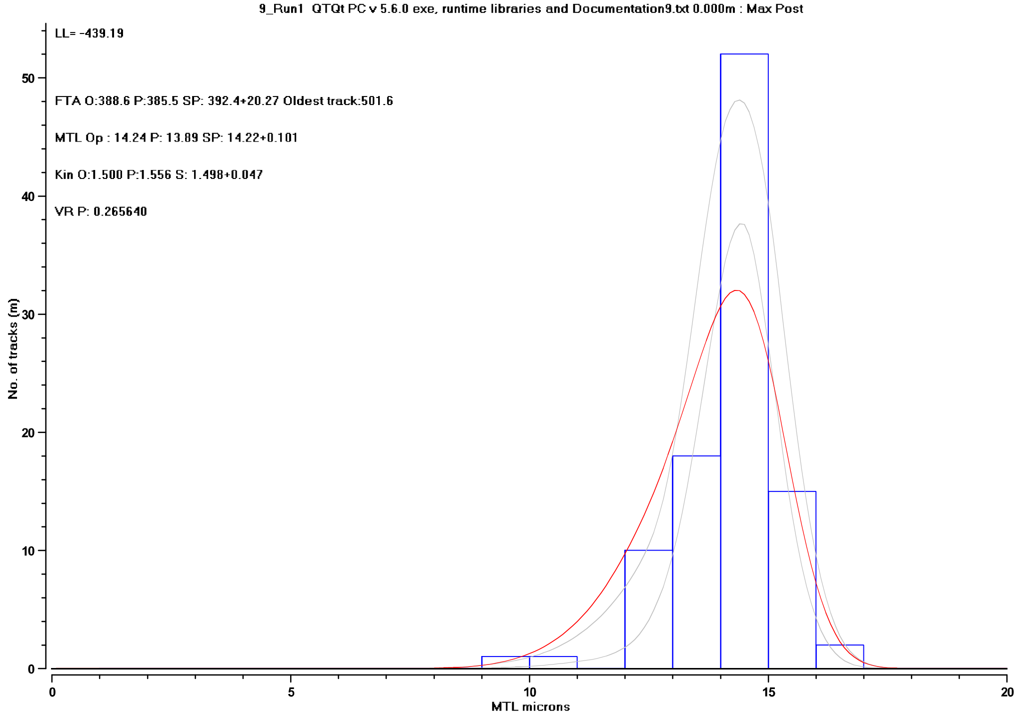
9_Run1 QTQt PC v 5.6.0 exe, runtime libraries and Documentation9.txt 0.000m : Max Post
LL= -439.19
FTA O:388.6 P:385.5 SP: 392.4+20.27 Oldest track:501.6
MTL Op : 14.24 P: 13.89 SP: 14.22+0.101
Kin O:1.500 P:1.556 S: 1.498+0.047
VR P: 0.265640
No. of tracks (m)
MTL microns

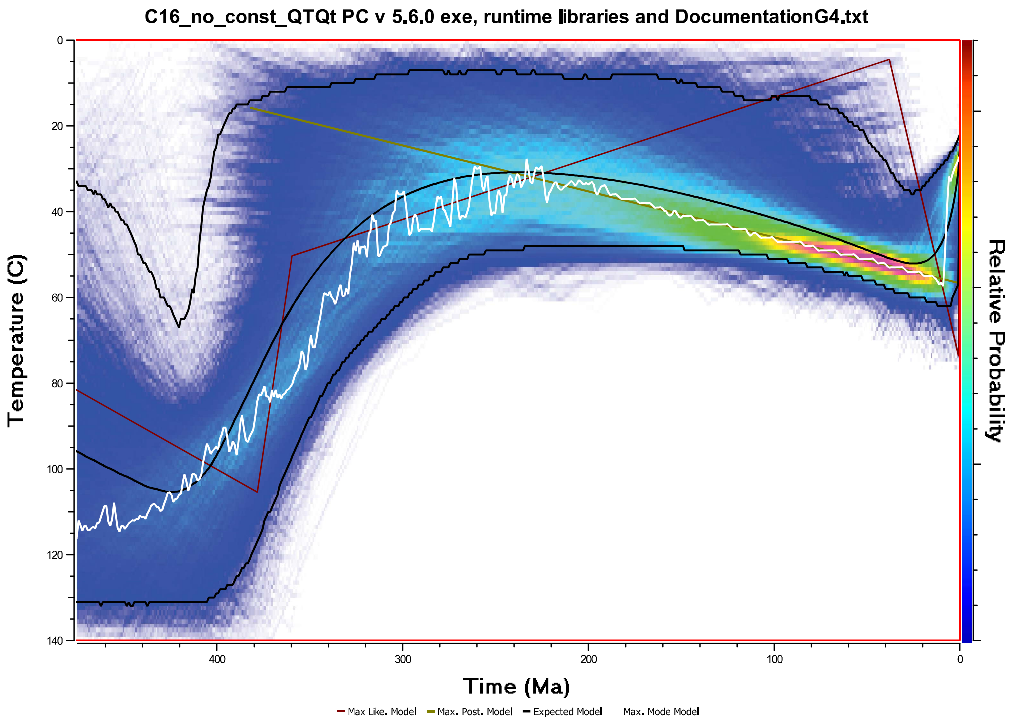
C16_no_const_QTQt PC v 5.6.0 exe, runtime libraries and DocumentationG4.txt
Temperature (C)
Time (Ma)
Relative Probability
Max Like. Model
Max. Post. Model
Expected Model
Max. Mode Model

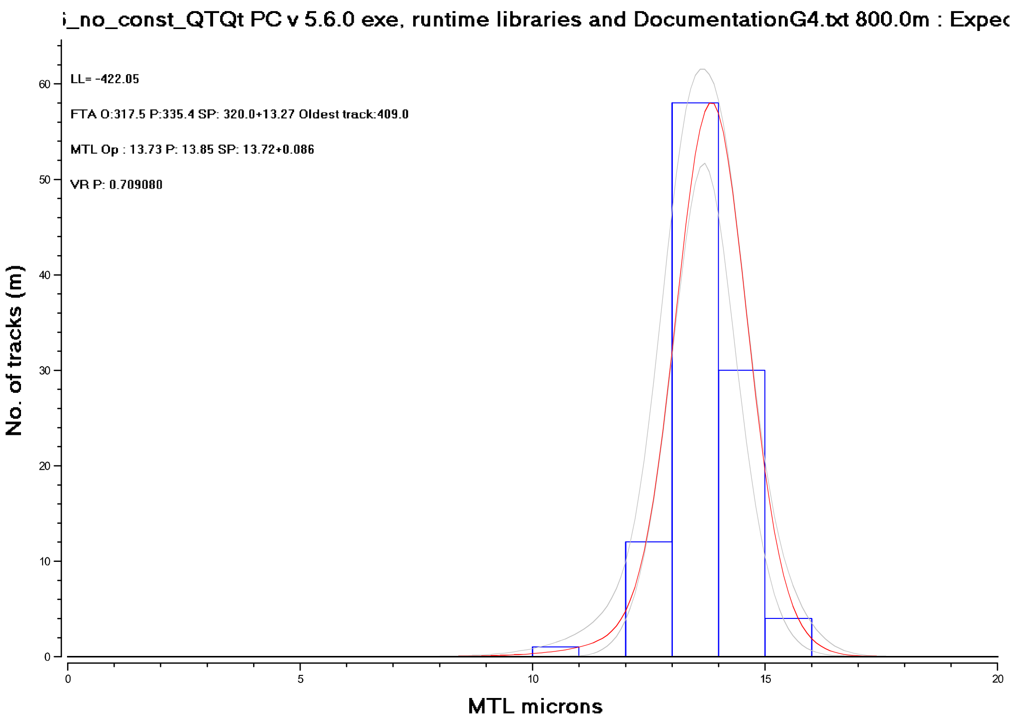
_no_const_QTQt PC v 5.6.0 exe, runtime libraries and DocumentationG4.txt 800.0m : Expec
LL= -422.05
FTA O:317.5 P:335.4 SP: 320.0+13.27 Oldest track:409.0
MTL Op : 13.73 P: 13.85 SP: 13.72+0.086
VR P: 0.709080
No. of tracks (m)
MTL microns

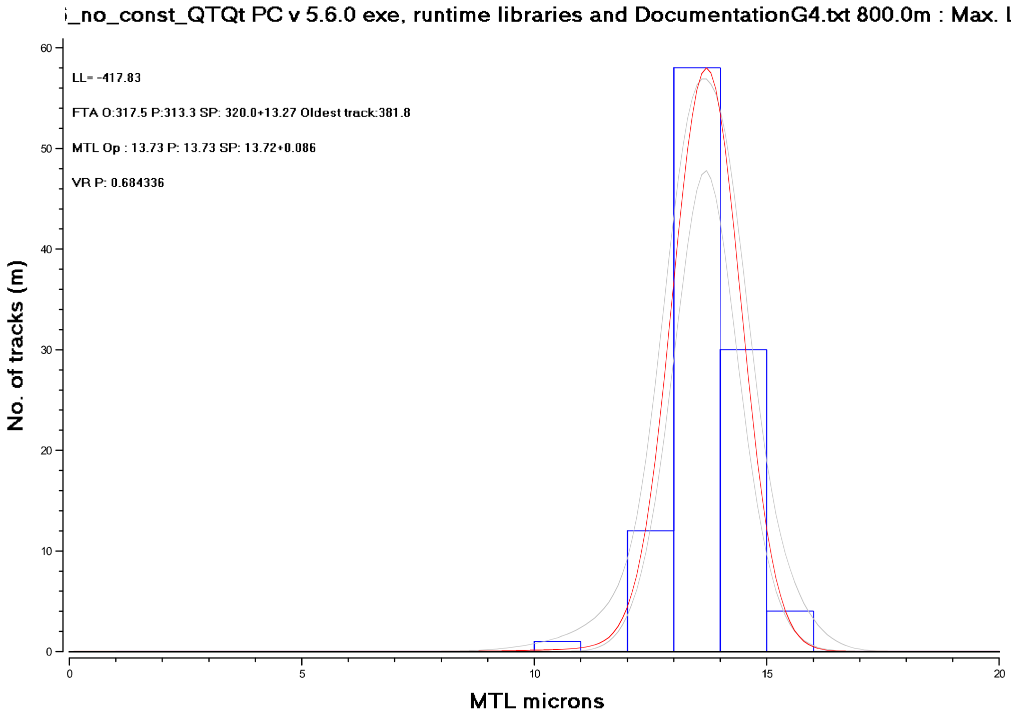
_no_const_QTQt PC v 5.6.0 exe, runtime libraries and DocumentationG4.txt 800.0m : Max. L
LL= -417.83
FTA O:317.5 P:313.3 SP: 320.0+13.27 Oldest track:381.8
MTL Op : 13.73 P: 13.73 SP: 13.72+0.086
VR P: 0.684336
No. of tracks (m)
MTL microns

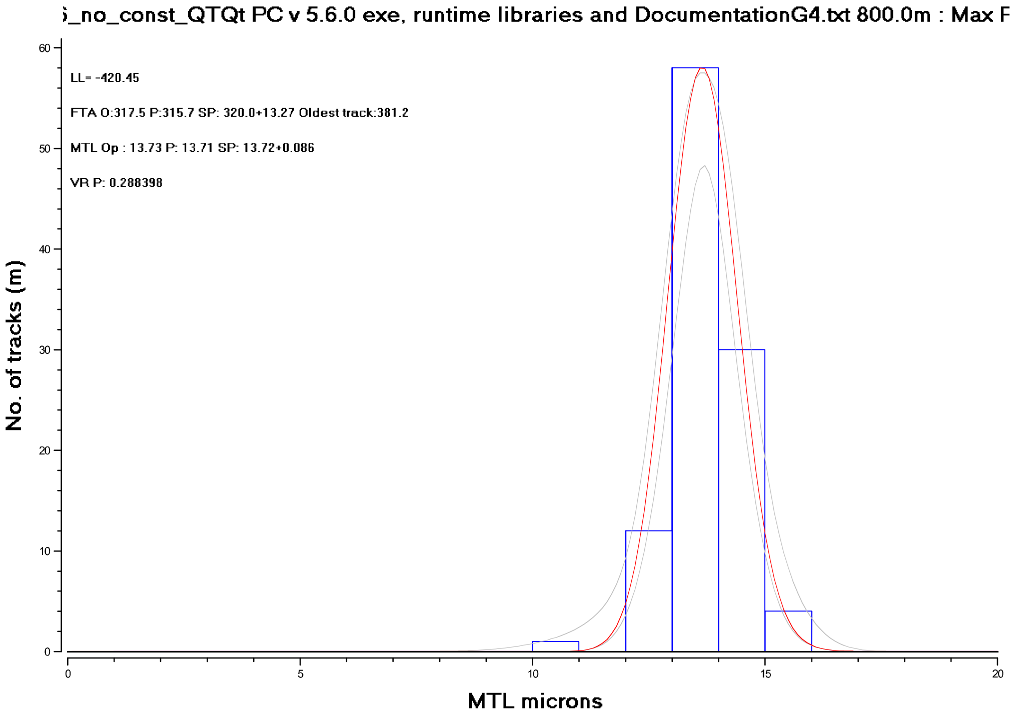
_no_const_QTQt PC v 5.6.0 exe, runtime libraries and DocumentationG4.txt 800.0m : Max F
LL= -420.45
FTA O:317.5 P:315.7 SP: 320.0+13.27 Oldest track:381.2
MTL Op : 13.73 P: 13.71 SP: 13.72+0.086
VR P: 0.288398
No. of tracks (m)
MTL microns

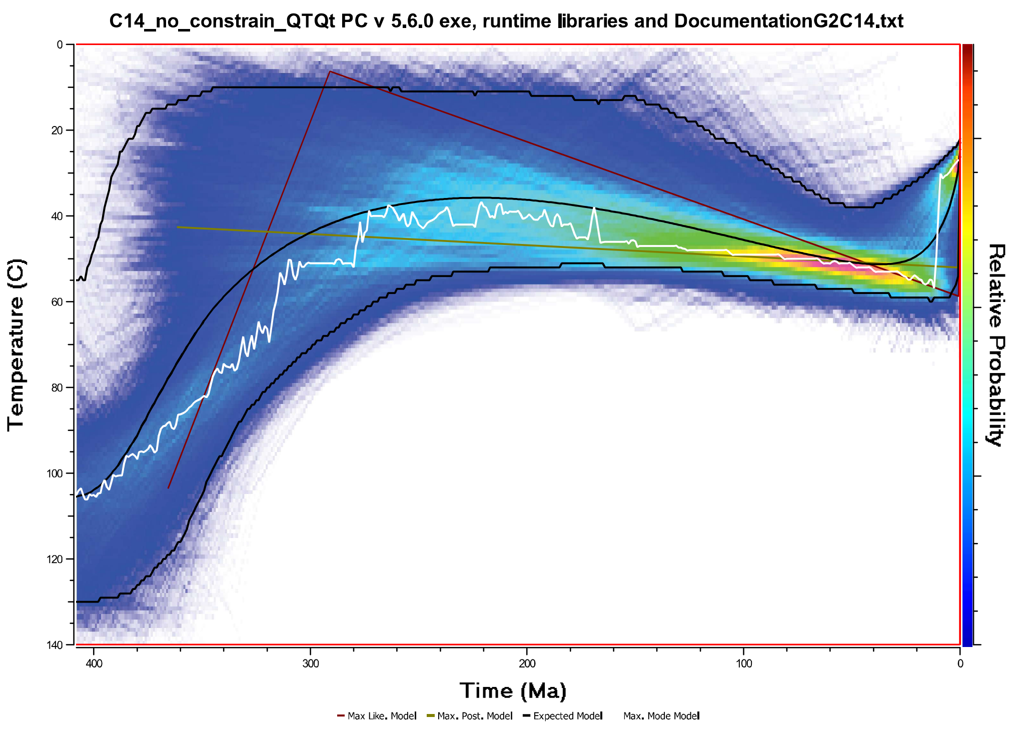
C14_no_constrain_QTQt PC v 5.6.0 exe, runtime libraries and DocumentationG2C14.txt
Temperature (C)
Time (Ma)
Relative Probability
Max Like. Model
Max. Post. Model
Expected Model
Max. Mode Model

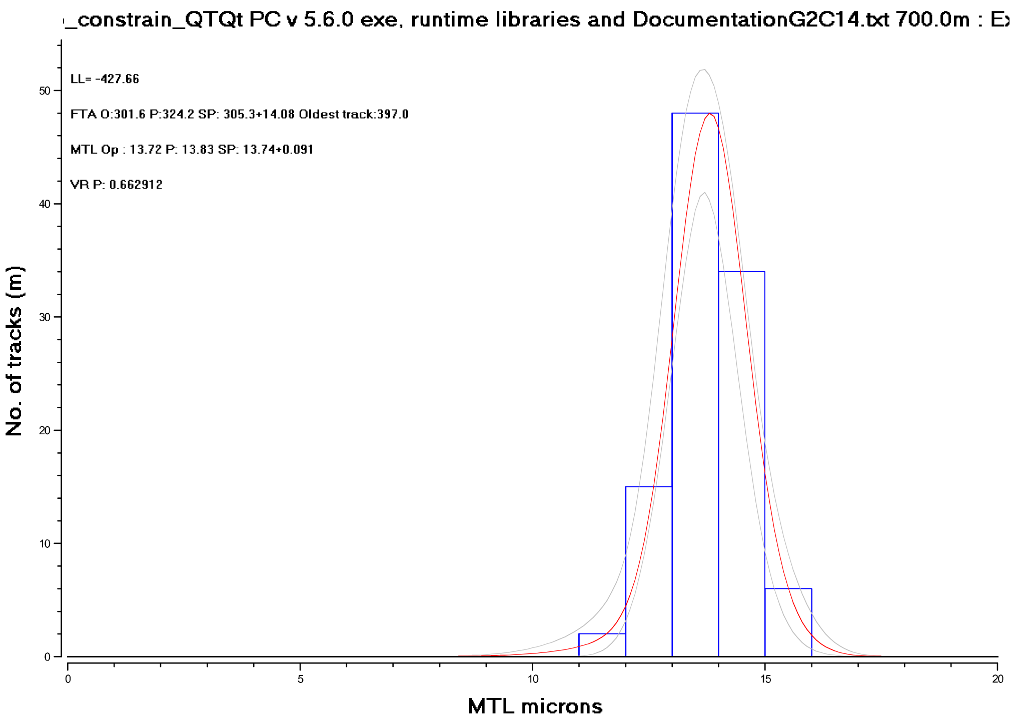
_constrain_QTQt PC v 5.6.0 exe, runtime libraries and DocumentationG2C14.txt 700.0m : E
LL= -427.66
FTA O:301.6 P:324.2 SP: 305.3+14.08 Oldest track:397.0
MTL Op : 13.72 P: 13.83 SP: 13.74+0.091
VR P: 0.662912
No. of tracks (m)
MTL microns

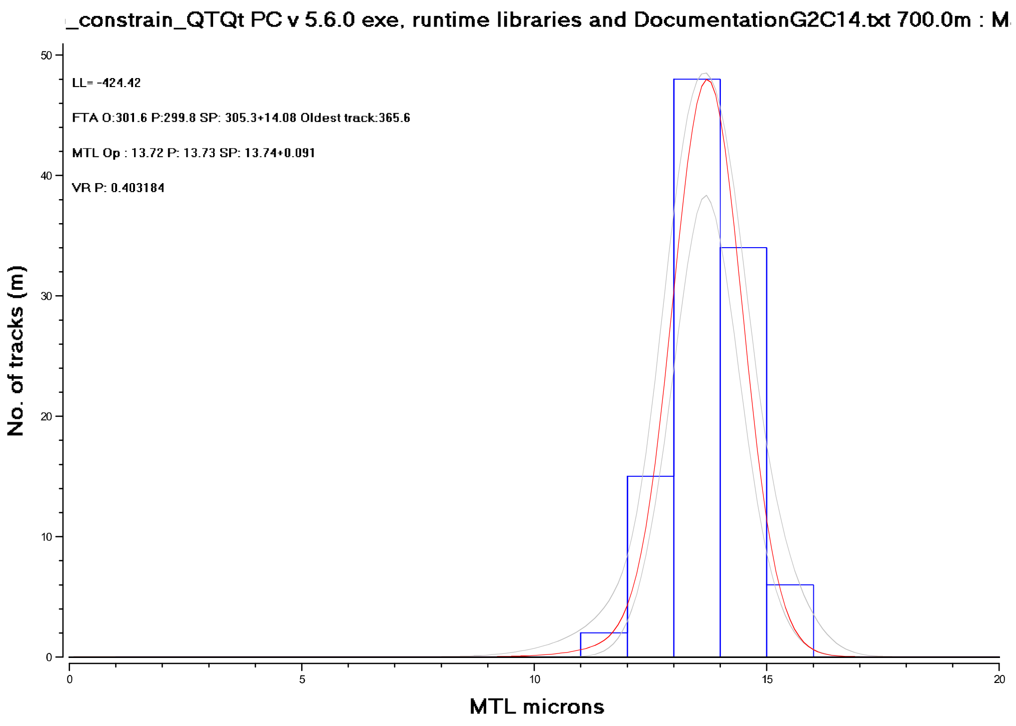
_constrain_QTQt PC v 5.6.0 exe, runtime libraries and DocumentationG2C14.txt 700.0m : M
LL= -424.42
FTA O:301.6 P:299.8 SP: 305.3+14.08 Oldest track:365.6
MTL Op : 13.72 P: 13.73 SP: 13.74+0.091
VR P: 0.403184
No. of tracks (m)
MTL microns

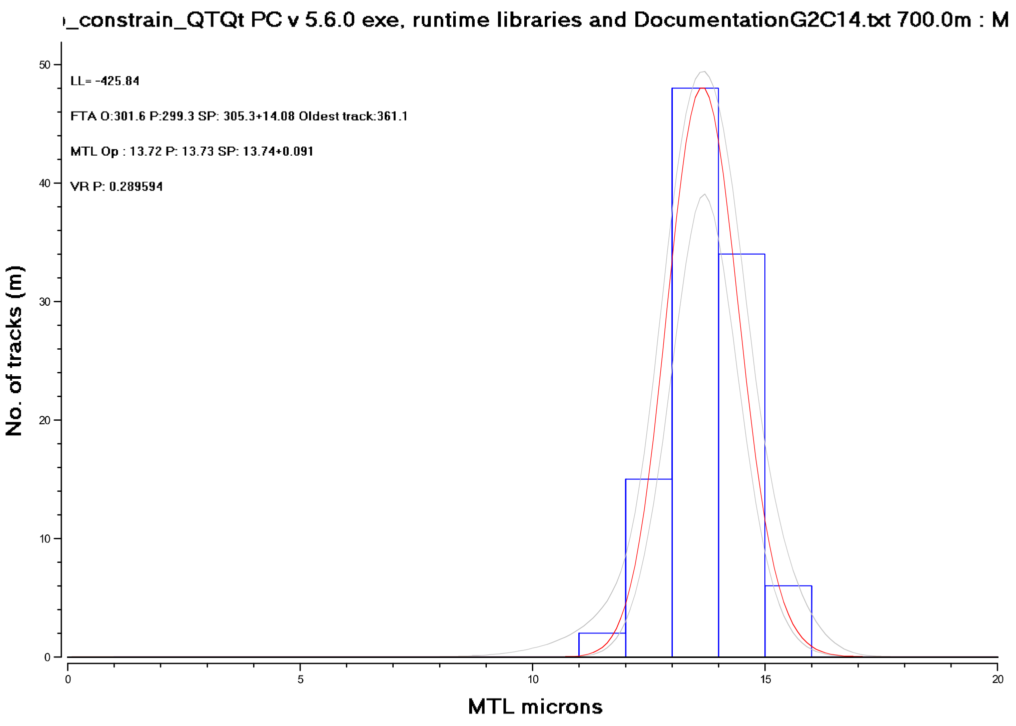
_constrain_QTQt PC v 5.6.0 exe, runtime libraries and DocumentationG2C14.txt 700.0m : M
LL= -425.84
FTA O:301.6 P:299.3 SP: 305.3+14.08 Oldest track:361.1
MTL Op : 13.72 P: 13.73 SP: 13.74+0.091
VR P: 0.289594
No. of tracks (m)
MTL microns

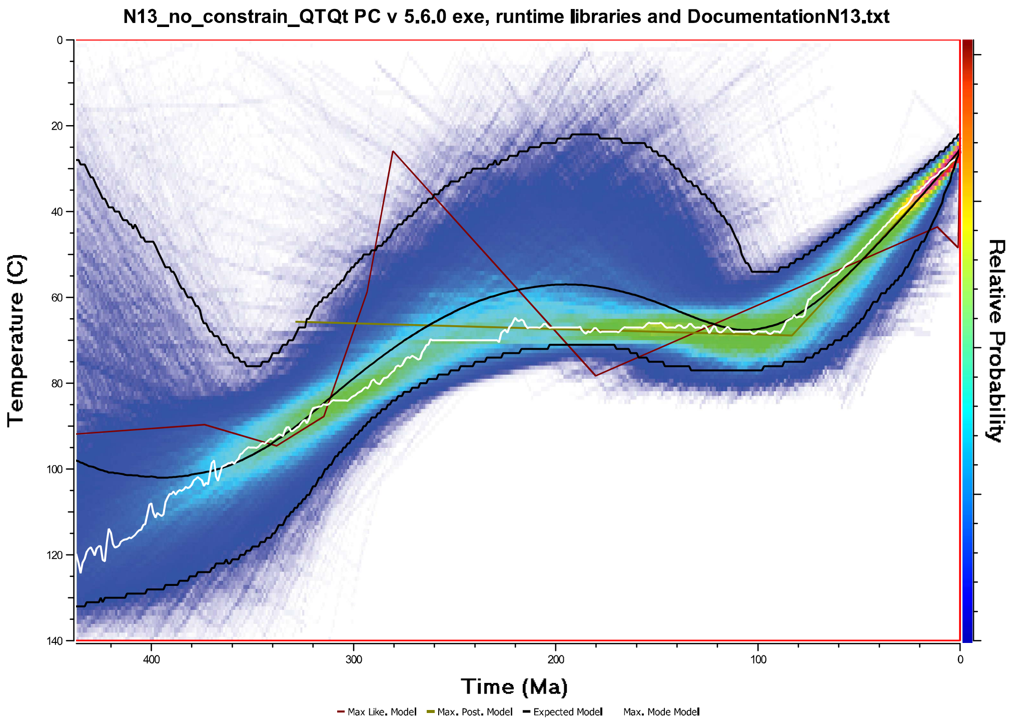
N13_no_constrain_QTQt PC v 5.6.0 exe, runtime libraries and DocumentationN13.txt
Temperature (C)
Time (Ma)
Relative Probability
Max Like. Model
Max. Post. Model
Expected Model
Max. Mode Model

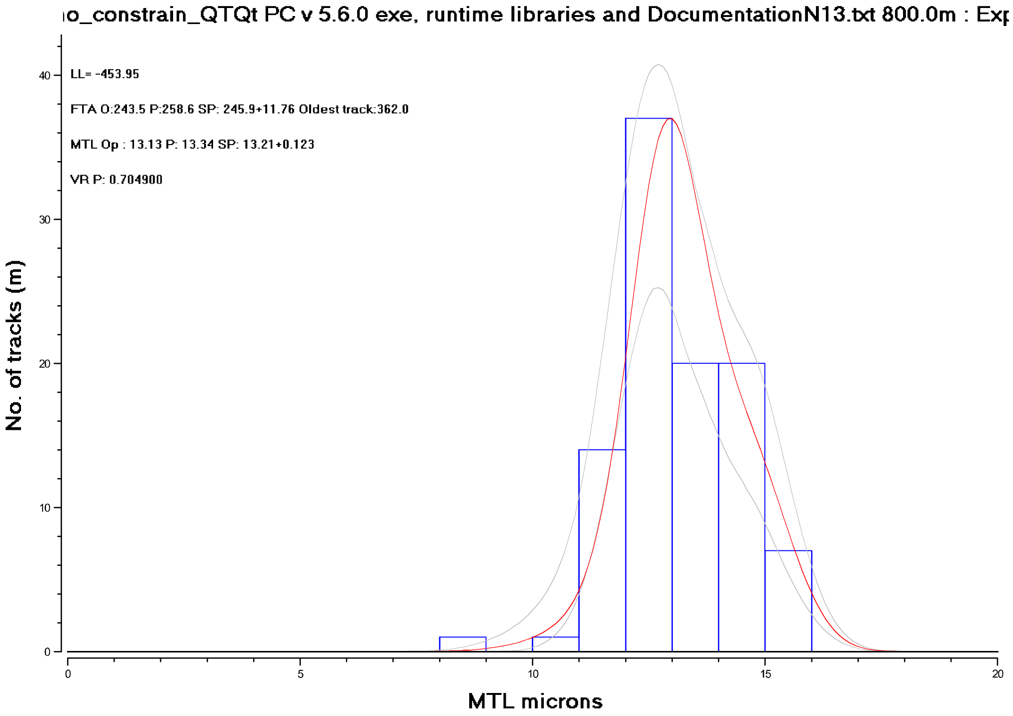
o_constrain_QTQt PC v 5.6.0 exe, runtime libraries and DocumentationN13.txt 800.0m : Exp
LL= -453.95
FTA O:243.5 P:258.6 SP: 245.9+11.76 Oldest track:362.0
MTL Op : 13.13 P: 13.34 SP: 13.21+0.123
VR P: 0.704900
No. of tracks (m)
MTL microns

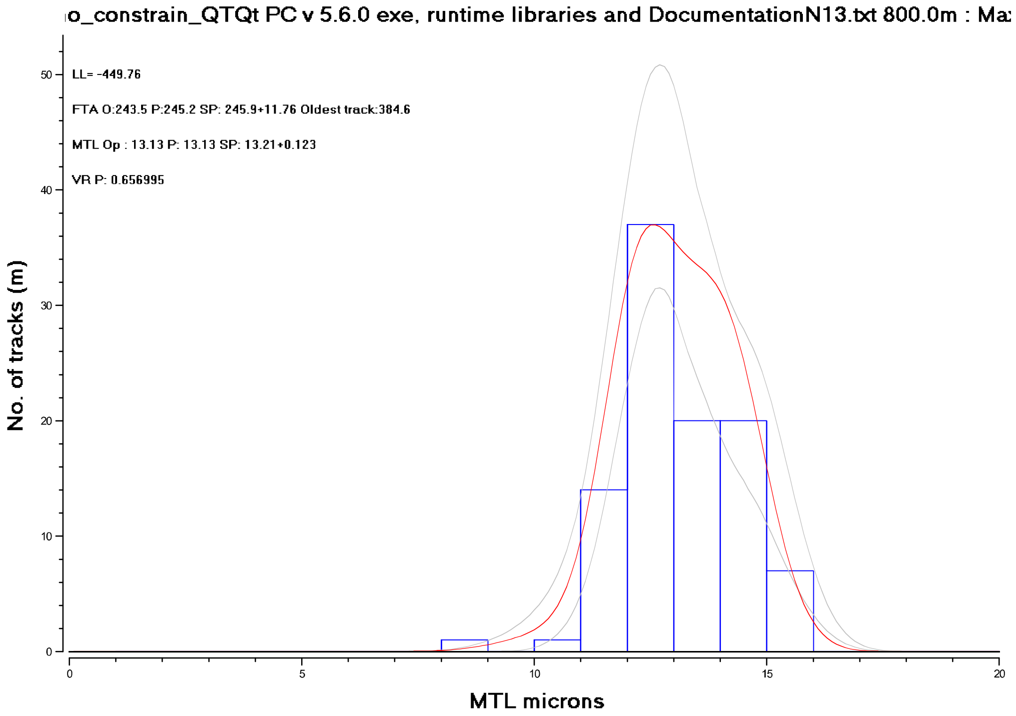
o_constrain_QTQt PC v 5.6.0 exe, runtime libraries and DocumentationN13.txt 800.0m : Ma
LL= -449.76
FTA O:243.5 P:245.2 SP: 245.9+11.76 Oldest track:384.6
MTL Op : 13.13 P: 13.13 SP: 13.21+0.123
VR P: 0.656995
No. of tracks (m)
MTL microns

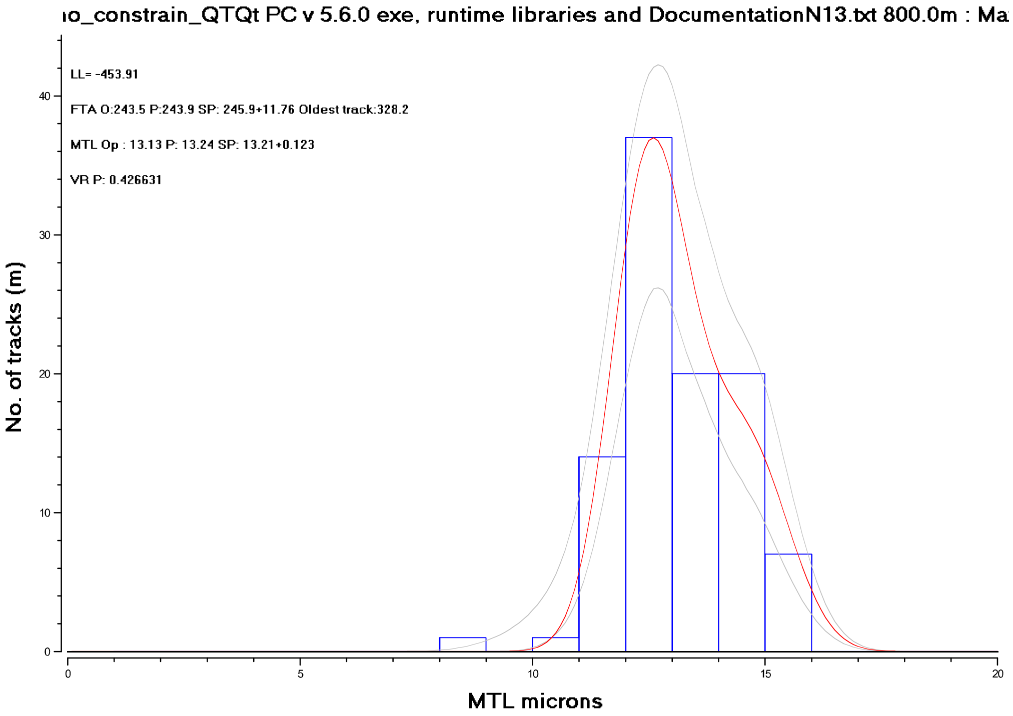
o_constrain_QTQt PC v 5.6.0 exe, runtime libraries and DocumentationN13.txt 800.0m : Ma
LL= -453.91
FTA O:243.5 P:243.9 SP: 245.9+11.76 Oldest track:328.2
MTL Op : 13.13 P: 13.24 SP: 13.21+0.123
VR P: 0.426631
No. of tracks (m)
MTL microns

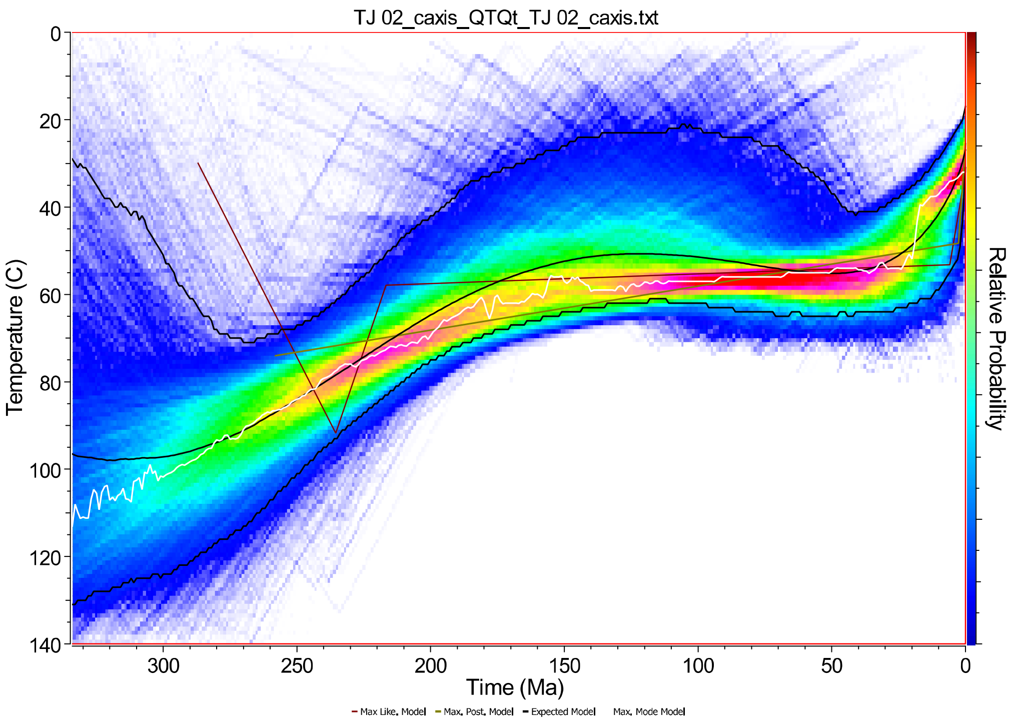
TJ 02_caxis_QTQt_TJ 02_caxis.txt
Temperature (C)
Time (Ma)
Relative Probability
Max Like. Model
Max. Post. Model
Expected Model
Max. Mode Model

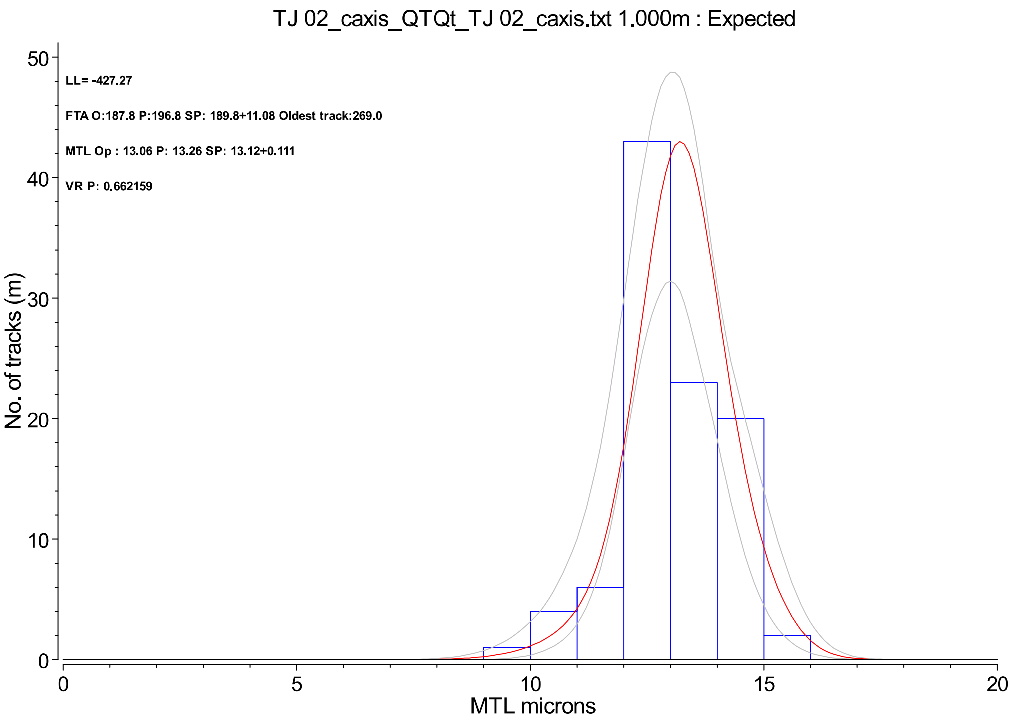
TJ 02_caxis_QTQt_TJ 02_caxis.txt 1.000m : Expected
LL= -427.27
FTA O:187.8 P:196.8 SP: 189.8+11.08 Oldest track:269.0
MTL Op : 13.06 P: 13.26 SP: 13.12+0.111
VR P: 0.662159
No. of tracks (m)
MTL microns

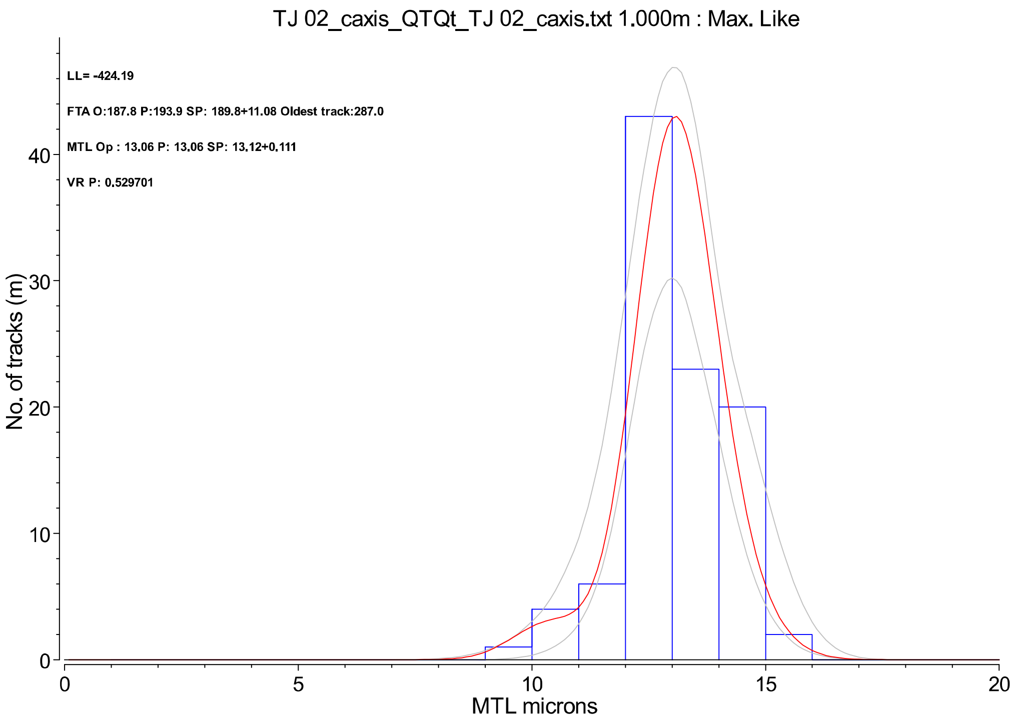
TJ 02_caxis_QTQt_TJ 02_caxis.txt 1.000m : Max. Like
LL= -424.19
FTA O:187.8 P:193.9 SP: 189.8+11.08 Oldest track:287.0
MTL Op : 13.06 P: 13.06 SP: 13.12+0.111
VR P: 0.529701
No. of tracks (m)
MTL microns

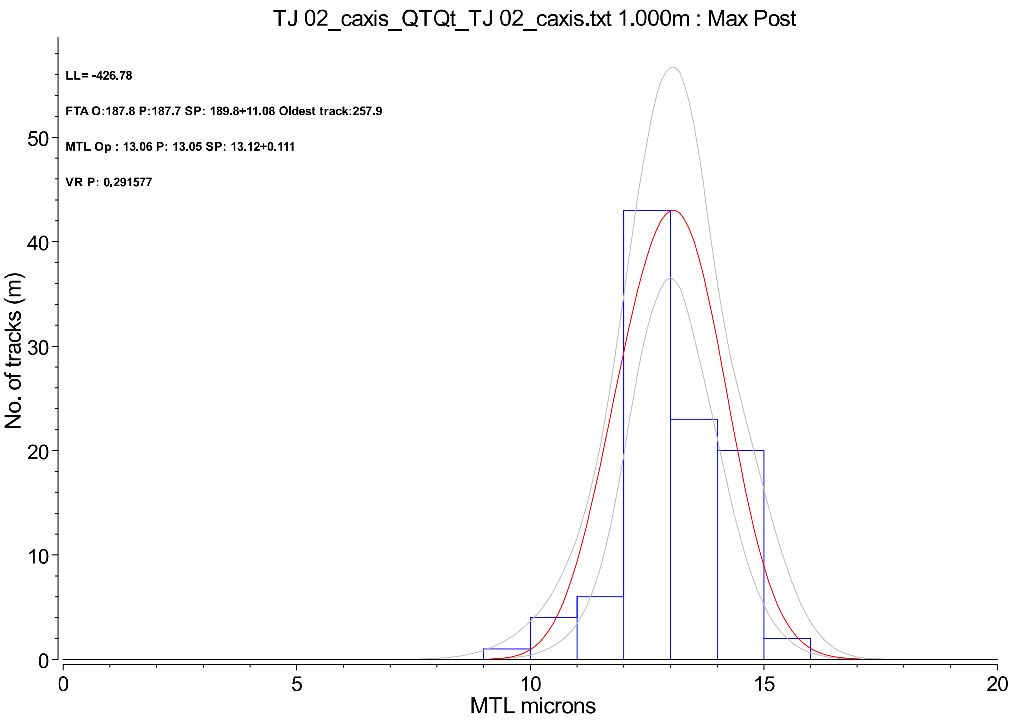
TJ 02_caxis_QTQt_TJ 02_caxis.txt 1.000m : Max Post
LL= -426.78
FTA O:187.8 P:187.7 SP: 189.8+11.08 Oldest track:257.9
MTL Op : 13.06 P: 13.05 SP: 13.12+0.111
VR P: 0.291577
No. of tracks (m)
MTL microns

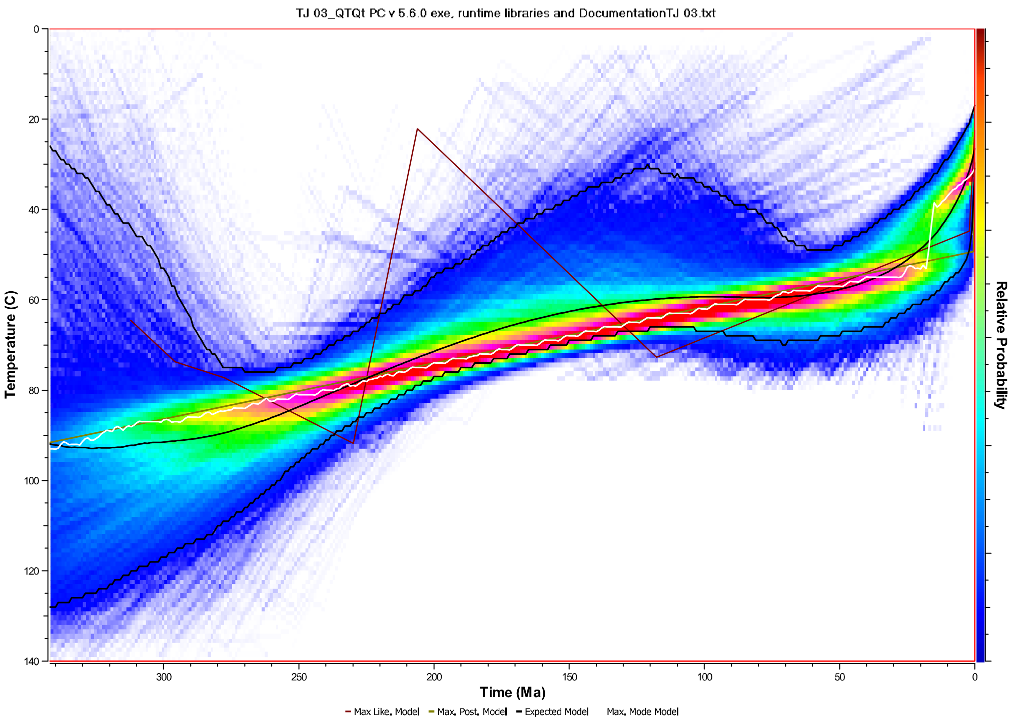
TJ 03_QTQt PC v 5.6.0 exe, runtime libraries and DocumentationTJ 03.txt
Temperature (C)
Time (Ma)
Relative Probability
Max Like. Model
Max. Post. Model
Expected Model
Max. Mode Model

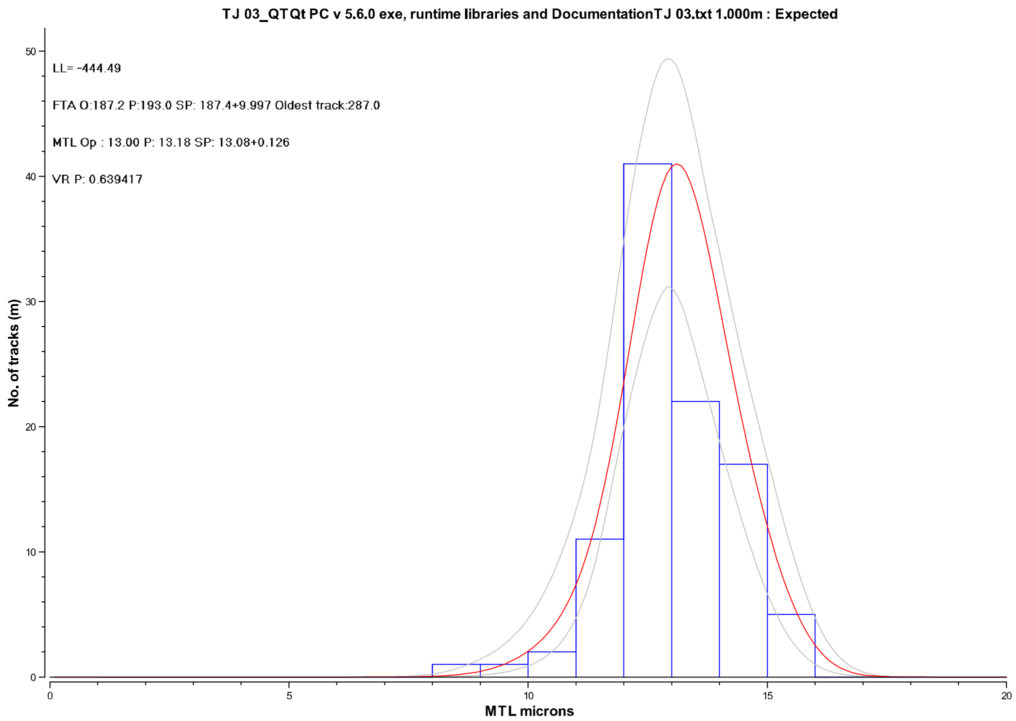
TJ 03_QTQt PC v 5.6.0 exe, runtime libraries and DocumentationTJ 03.txt 1.000m : Expected
LL= -444.49
FTA O:187.2 P:193.0 SP: 187.4+9.997 Oldest track:287.0
MTL Op : 13.00 P: 13.18 SP: 13.08+0.126
VR P: 0.639417
No. of tracks (m)
MTL microns

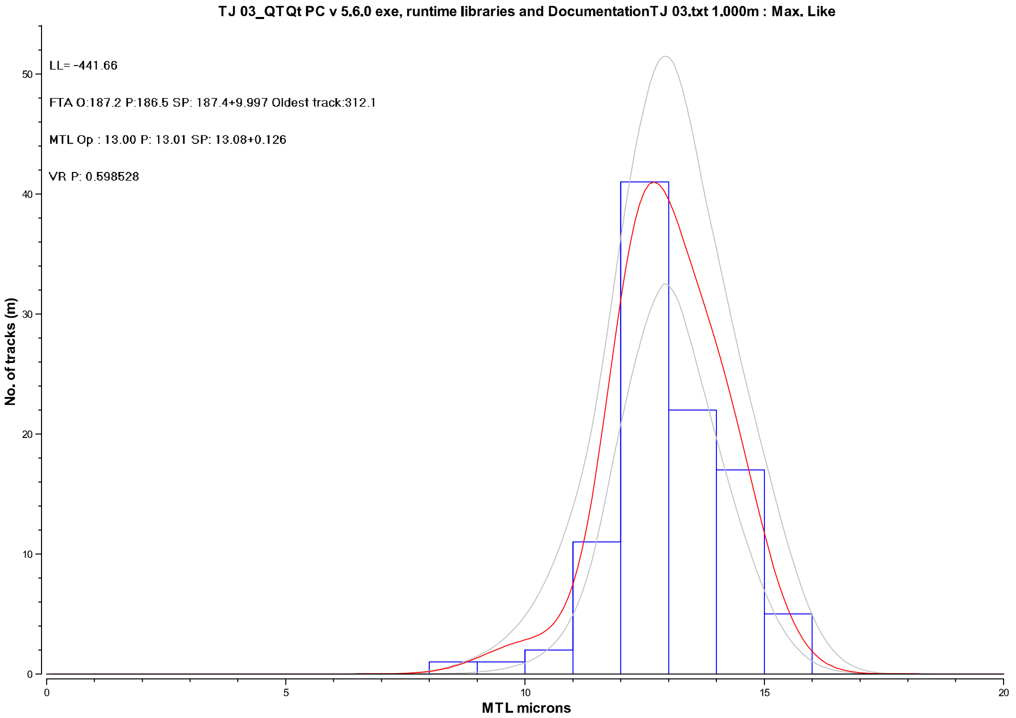
TJ 03_QTQt PC v 5.6.0 exe, runtime libraries and DocumentationTJ 03.txt 1.000m : Max. Like
LL= -441.66
FTA O:187.2 P:186.5 SP: 187.4+9.997 Oldest track:312.1
MTL Op : 13.00 P: 13.01 SP: 13.08+0.126
VR P: 0.598528
No. of tracks (m)
MTL microns

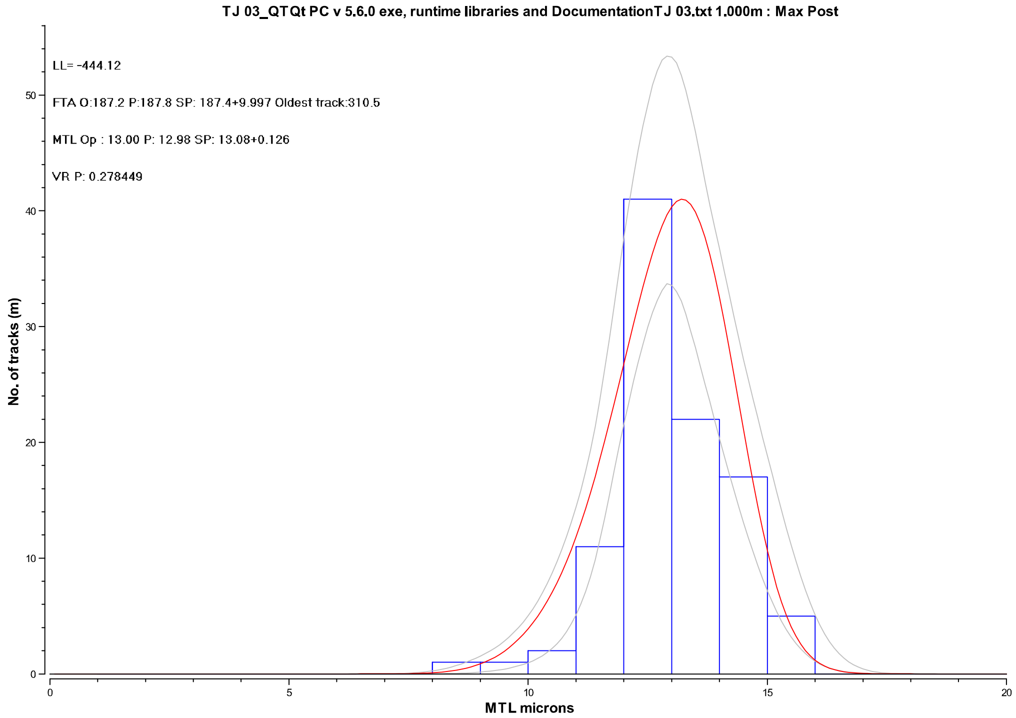
TJ 03_QTQt PC v 5.6.0 exe, runtime libraries and DocumentationTJ 03.txt 1.000m : Max Post
LL= -444.12
FTA O:187.2 P:187.8 SP: 187.4+9.997 Oldest track:310.5
MTL Op : 13.00 P: 12.98 SP: 13.08+0.126
VR P: 0.278449
No. of tracks (m)
MTL microns

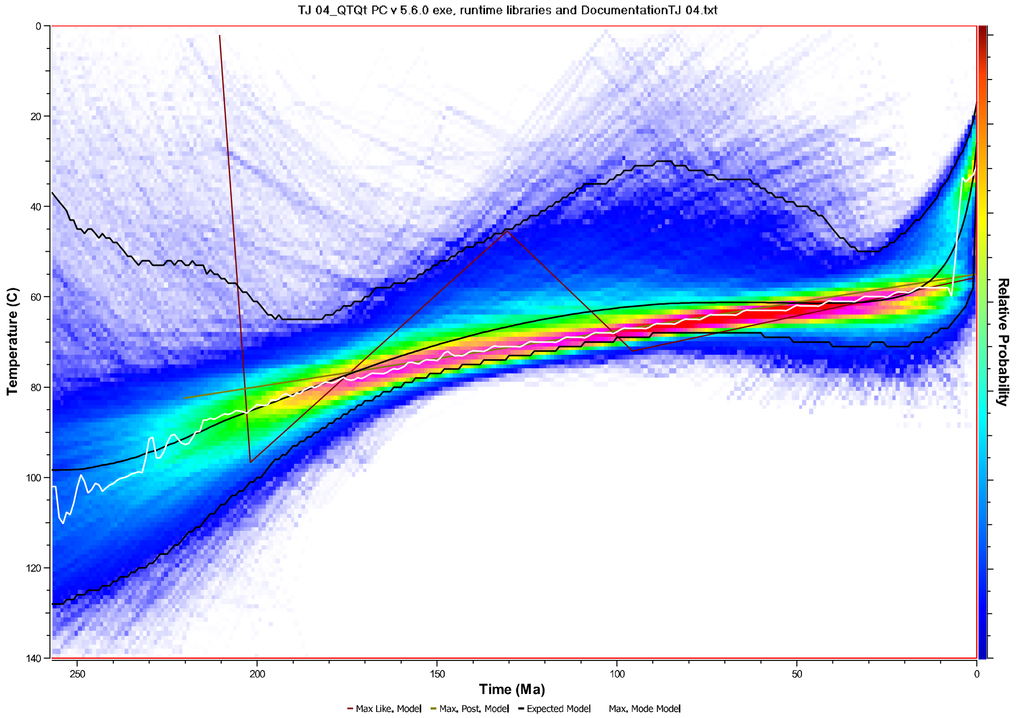
TJ 04_QTQt PC v 5.6.0 exe, runtime libraries and DocumentationTJ 04.txt
Temperature (C)
Time (Ma)
Relative Probability
Max Like. Model
Max. Post. Model
Expected Model
Max. Mode Model

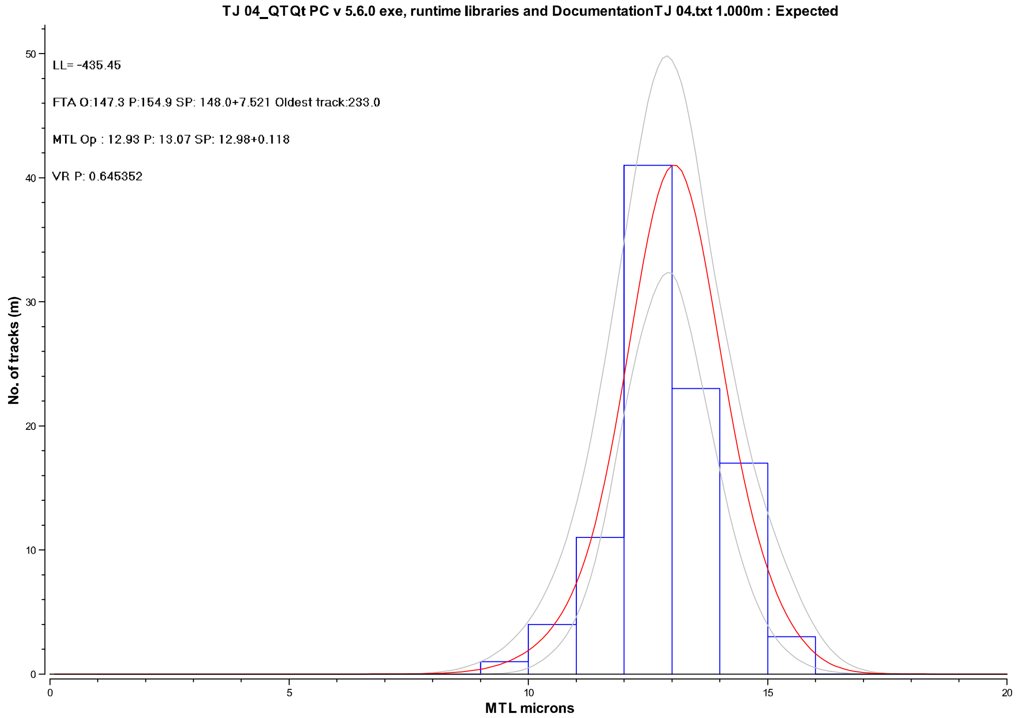
TJ 04_QTQt PC v 5.6.0 exe, runtime libraries and DocumentationTJ 04.txt 1.000m : Expected
LL= -435.45
FTA O:147.3 P:154.9 SP: 148.0+7.521 Oldest track:233.0
MTL Op : 12.93 P: 13.07 SP: 12.98+0.118
VR P: 0.645352
No. of tracks (m)
MTL microns

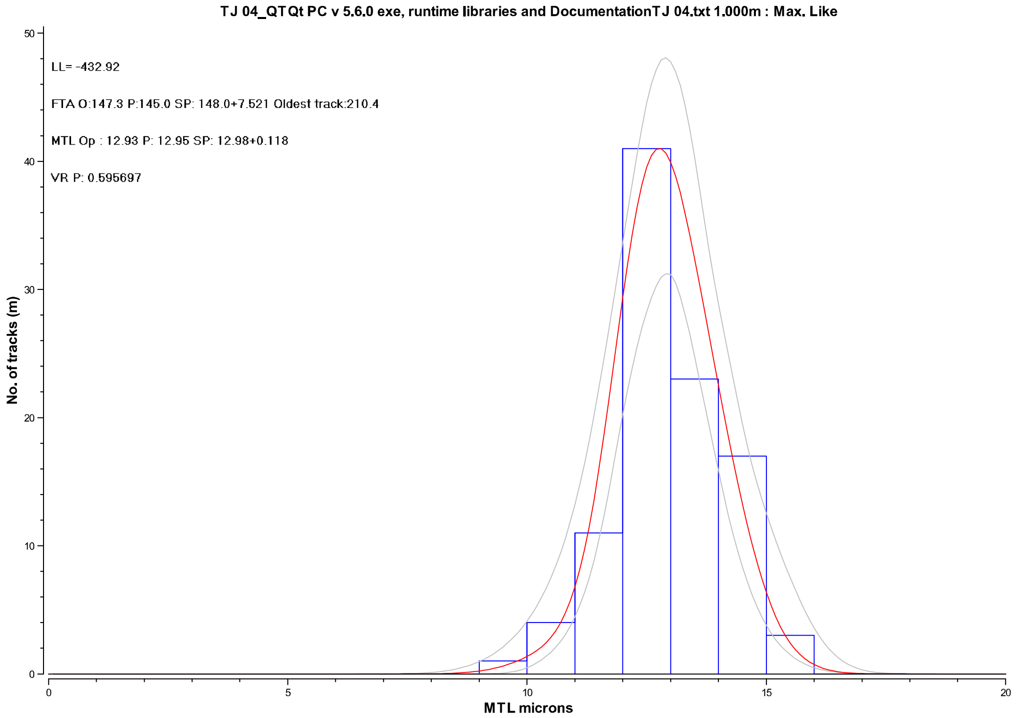
TJ 04_QTQt PC v 5.6.0 exe, runtime libraries and DocumentationTJ 04.txt 1.000m : Max. Like
LL= -432.92
FTA O:147.3 P:145.0 SP: 148.0+7.521 Oldest track:210.4
MTL Op : 12.93 P: 12.95 SP: 12.98+0.118
VR P: 0.595697
No. of tracks (m)
MTL microns

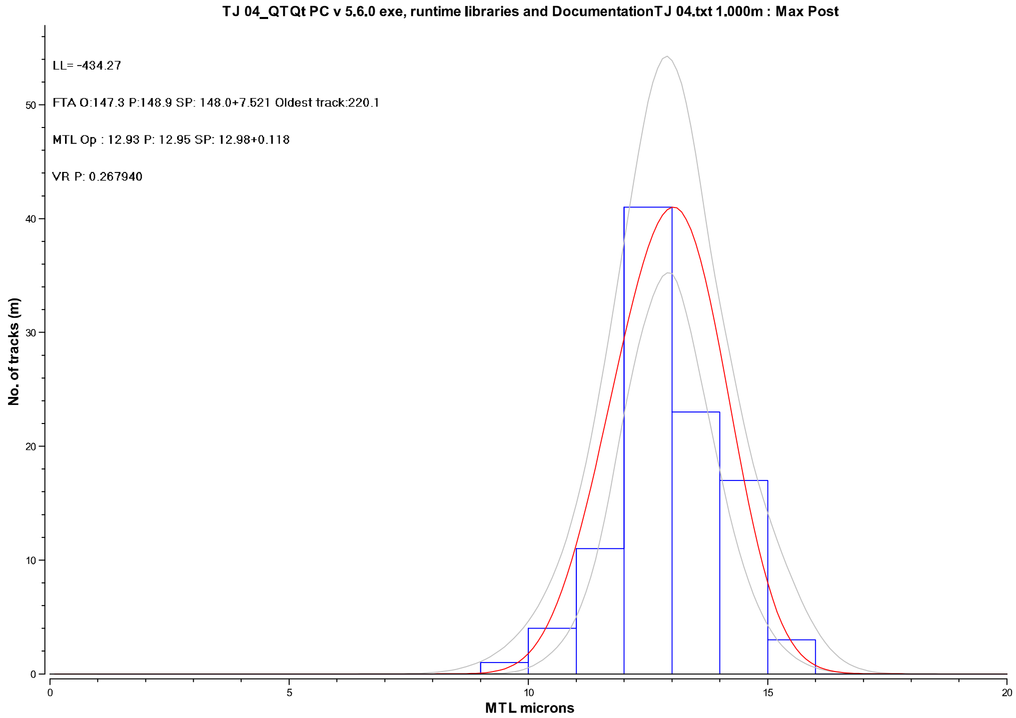
TJ 04_QTQt PC v 5.6.0 exe, runtime libraries and DocumentationTJ 04.txt 1.000m : Max Post
LL= -434.27
FTA O:147.3 P:148.9 SP: 148.0+7.521 Oldest track:220.1
MTL Op : 12.93 P: 12.95 SP: 12.98+0.118
VR P: 0.267940
No. of tracks (m)
MTL microns

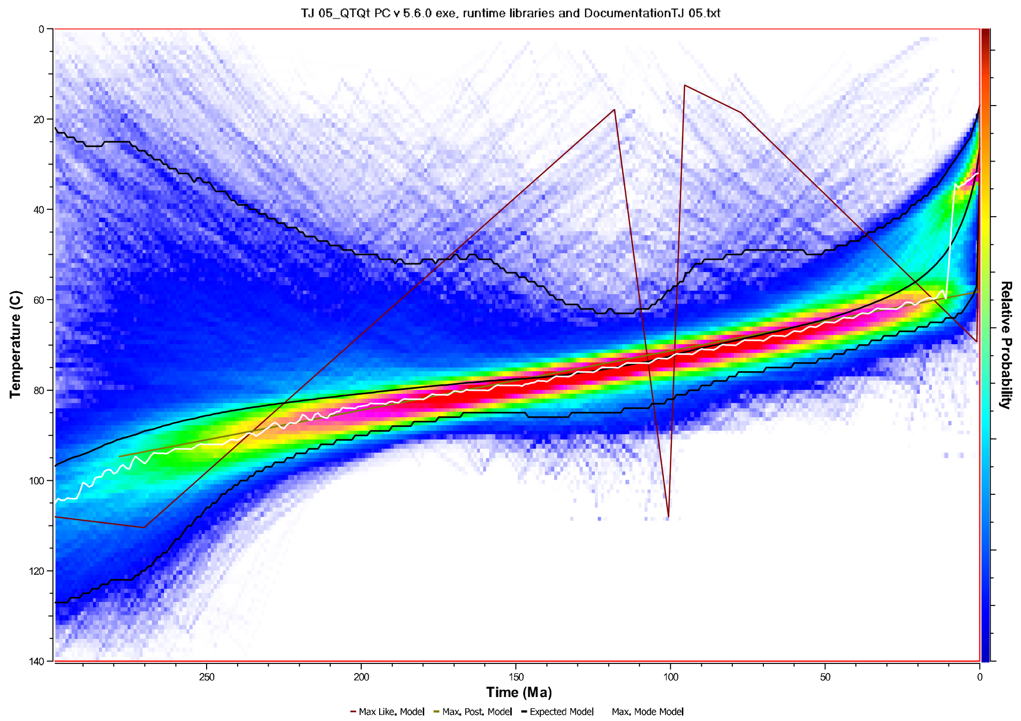
TJ 05_QTQt PC v 5.6.0 exe, runtime libraries and DocumentationTJ 05.txt
Temperature (C)
Time (Ma)
Relative Probability
Max Like. Model
Max. Post. Model
Expected Model
Max. Mode Model

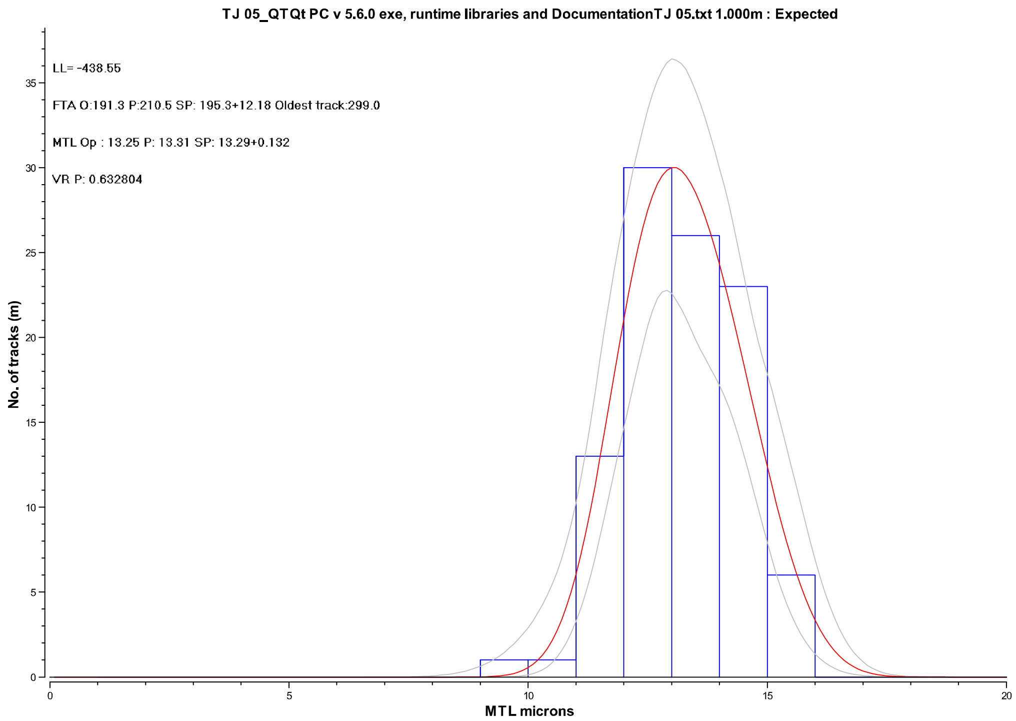
TJ 05_QTQt PC v 5.6.0 exe, runtime libraries and DocumentationTJ 05.txt 1.000m : Expected
LL= -438.55
FTA O:191.3 P:210.5 SP: 195.3+12.18 Oldest track:299.0
MTL Op : 13.25 P: 13.31 SP: 13.29+0.132
VR P: 0.632804
No. of tracks (m)
MTL microns

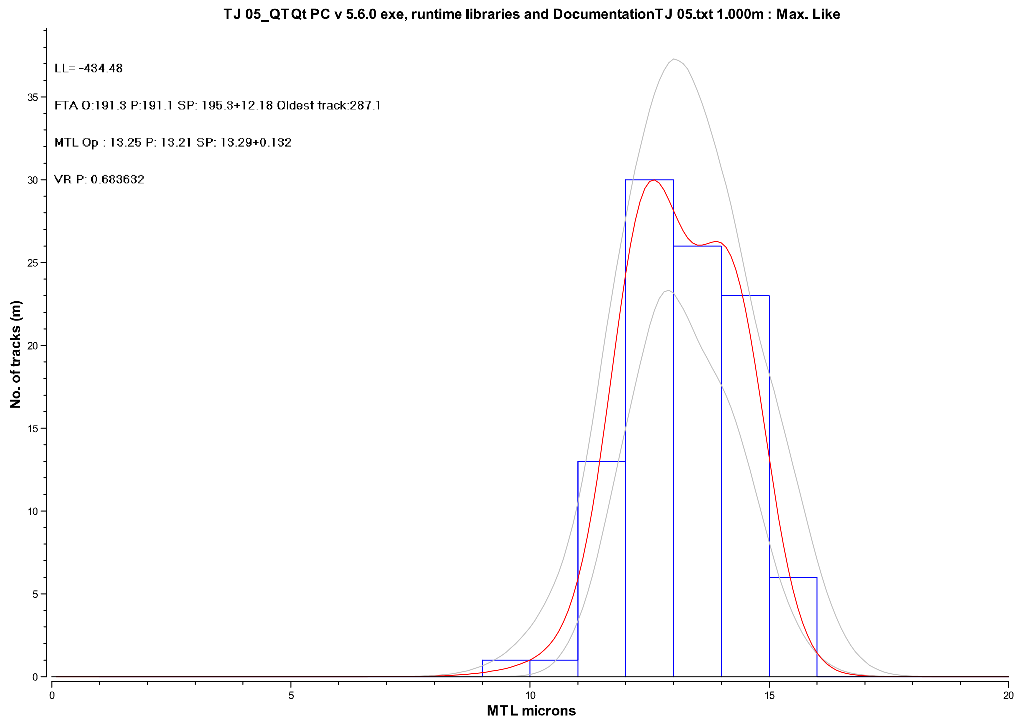
TJ 05_QTQt PC v 5.6.0 exe, runtime libraries and DocumentationTJ 05.txt 1.000m : Max. Like
LL= -434.48
FTA O:191.3 P:191.1 SP: 195.3+12.18 Oldest track:287.1
MTL Op : 13.25 P: 13.21 SP: 13.29+0.132
VR P: 0.683632
No. of tracks (m)
MTL microns

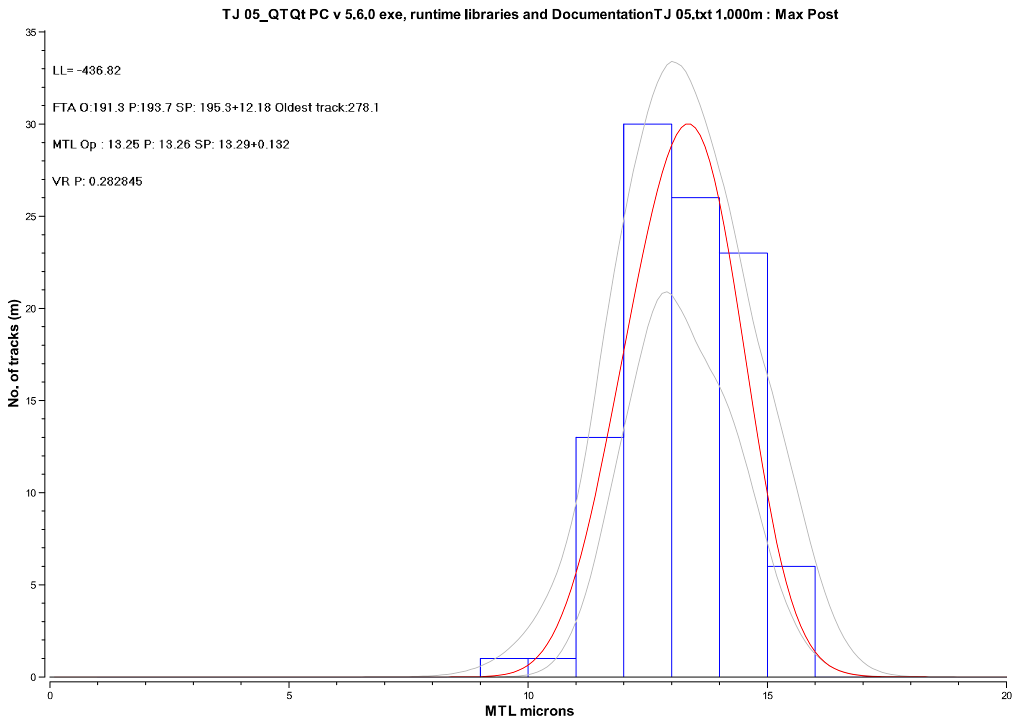
TJ 05_QTQt PC v 5.6.0 exe, runtime libraries and DocumentationTJ 05.txt 1.000m : Max Post
LL= -436.82
FTA O:191.3 P:193.7 SP: 195.3+12.18 Oldest track:278.1
MTL Op : 13.25 P: 13.26 SP: 13.29+0.132
VR P: 0.282845
No. of tracks (m)
MTL microns

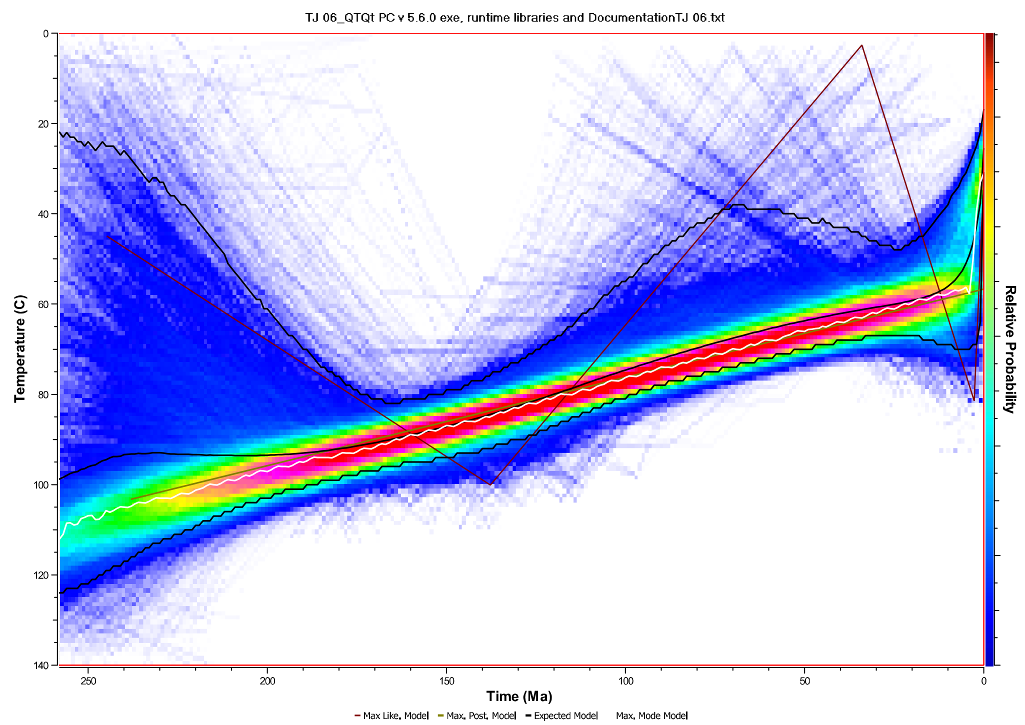
TJ 06_QTQt PC v 5.6.0 exe, runtime libraries and DocumentationTJ 06.txt
Temperature (C)
Time (Ma)
Relative Probability
Max Like. Model
Max. Post. Model
Expected Model
Max. Mode Model

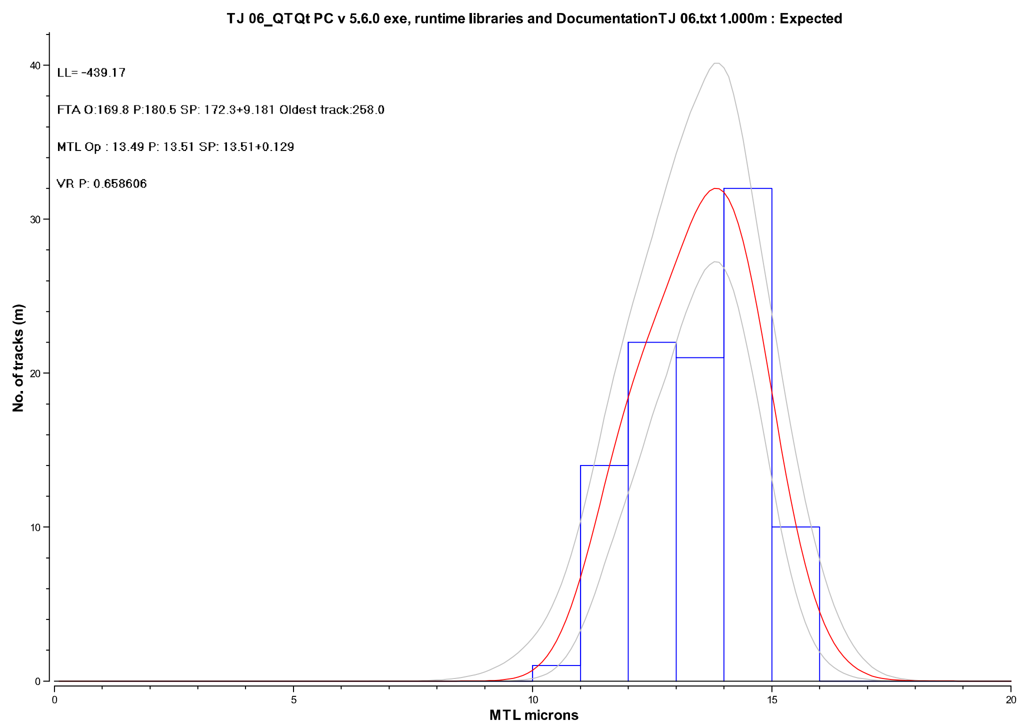
TJ 06_QTQt PC v 5.6.0 exe, runtime libraries and DocumentationTJ 06.txt 1.000m : Expected
LL= -439.17
FTA O:169.8 P:180.5 SP: 172.3+9.181 Oldest track:258.0
MTL Op : 13.49 P: 13.51 SP: 13.51+0.129
VR P: 0.658606
No. of tracks (m)
MTL microns

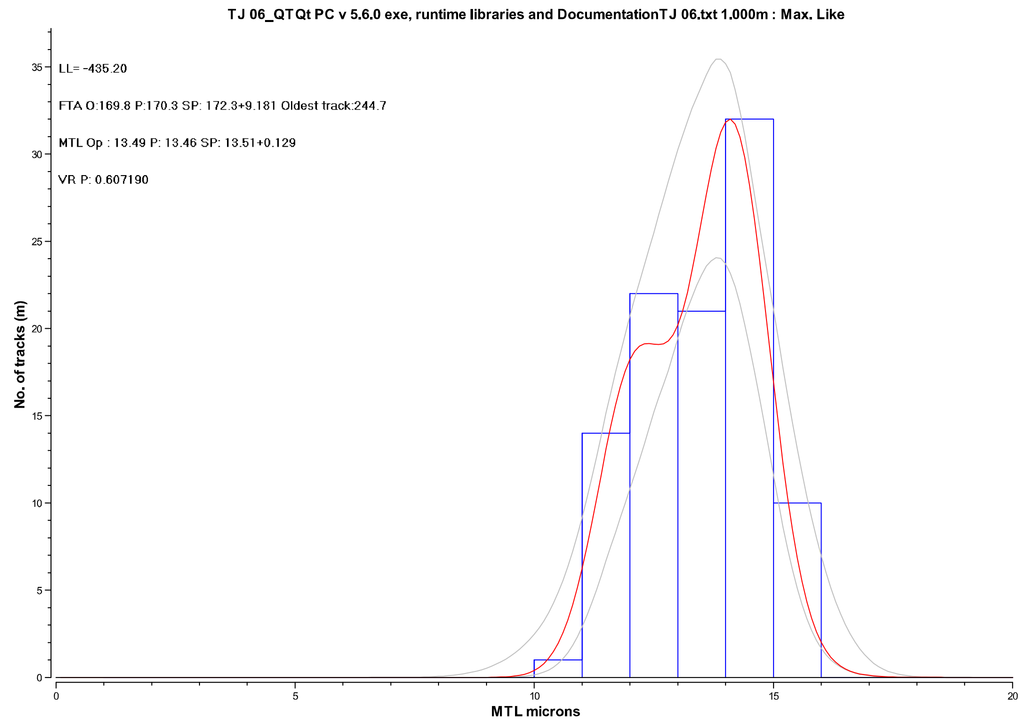
TJ 06_QTQt PC v 5.6.0 exe, runtime libraries and DocumentationTJ 06.txt 1.000m : Max. Like
LL= -435.20
FTA O:169.8 P:170.3 SP: 172.3+9.181 Oldest track:244.7
MTL Op : 13.49 P: 13.46 SP: 13.51+0.129
VR P: 0.607190
No. of tracks (m)
MTL microns

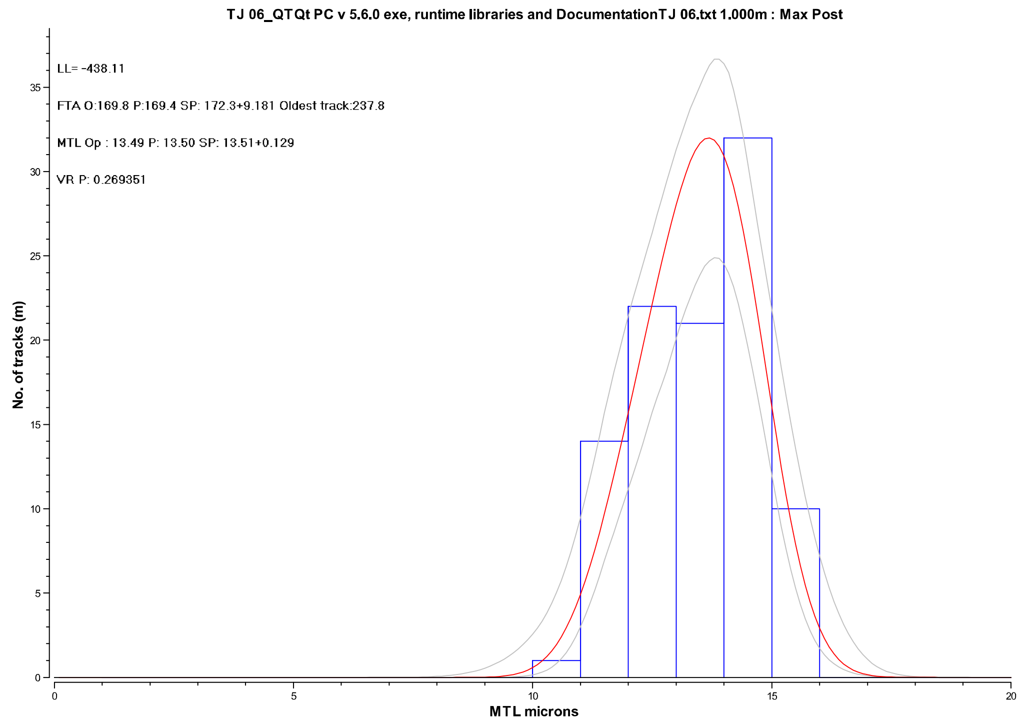
TJ 06_QTQt PC v 5.6.0 exe, runtime libraries and DocumentationTJ 06.txt 1.000m : Max Post
LL= -438.11
FTA O:169.8 P:169.4 SP: 172.3+9.181 Oldest track:237.8
MTL Op : 13.49 P: 13.50 SP: 13.51+0.129
VR P: 0.269351
No. of tracks (m)
MTL microns

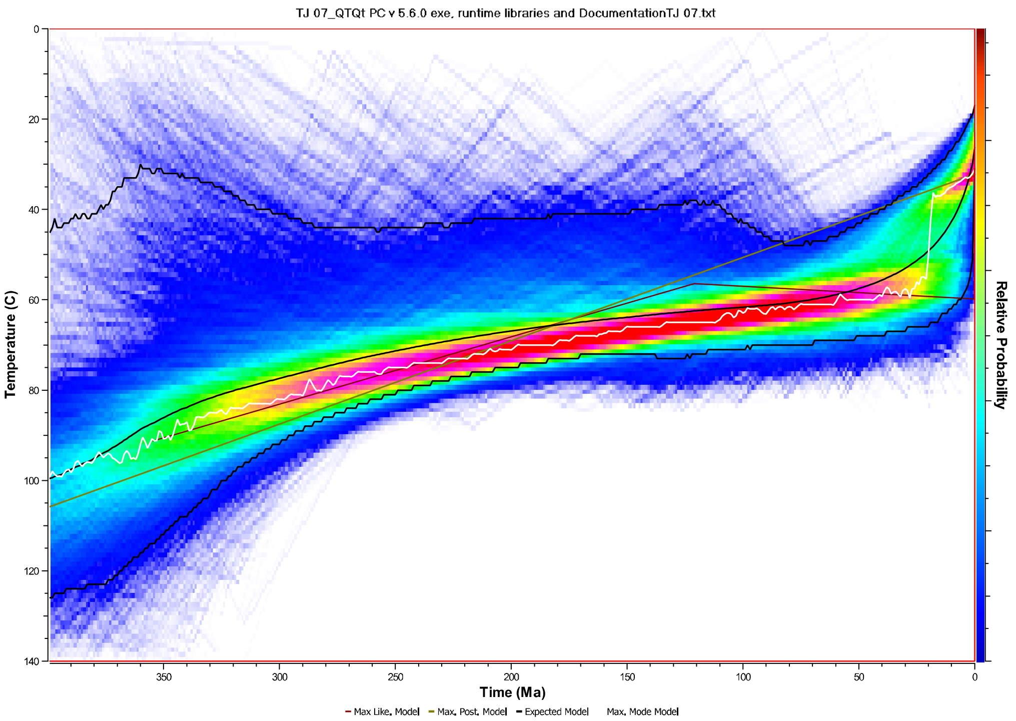
TJ 07_QTQt PC v 5.6.0 exe, runtime libraries and DocumentationTJ 07.txt
Temperature (C)
Time (Ma)
Relative Probability
Max Like. Model
Max. Post. Model
Expected Model
Max. Mode Model

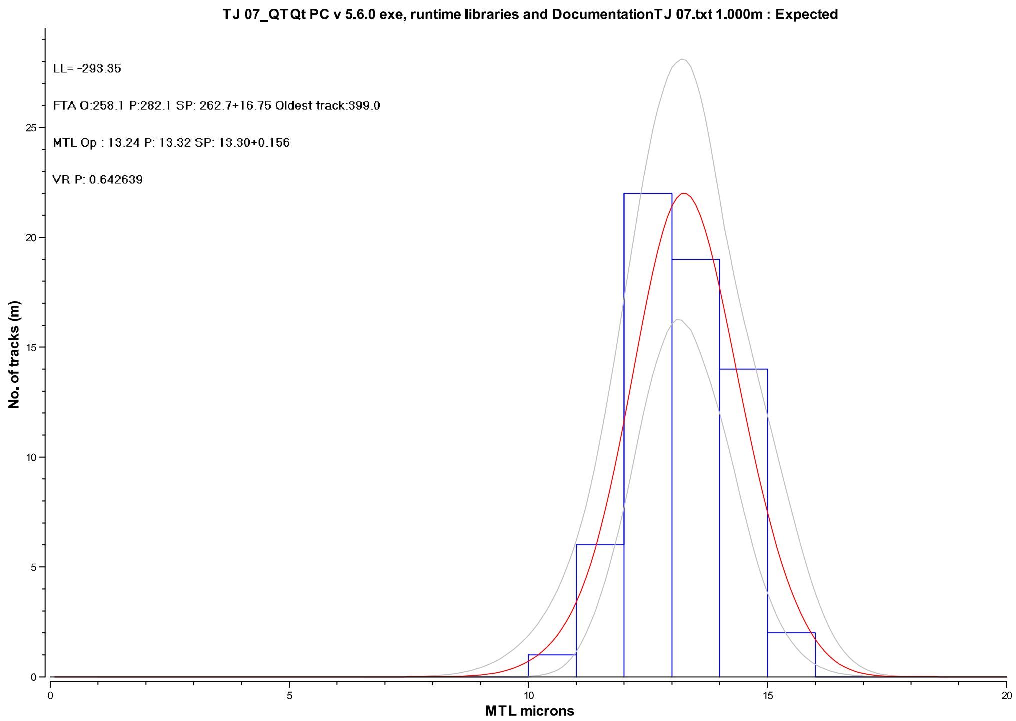
TJ 07_QTQt PC v 5.6.0 exe, runtime libraries and DocumentationTJ 07.txt 1.000m : Expected
LL= -293.35
FTA O:258.1 P:282.1 SP: 262.7+16.75 Oldest track:399.0
MTL Op : 13.24 P: 13.32 SP: 13.30+0.156
VR P: 0.642639
No. of tracks (m)
MTL microns

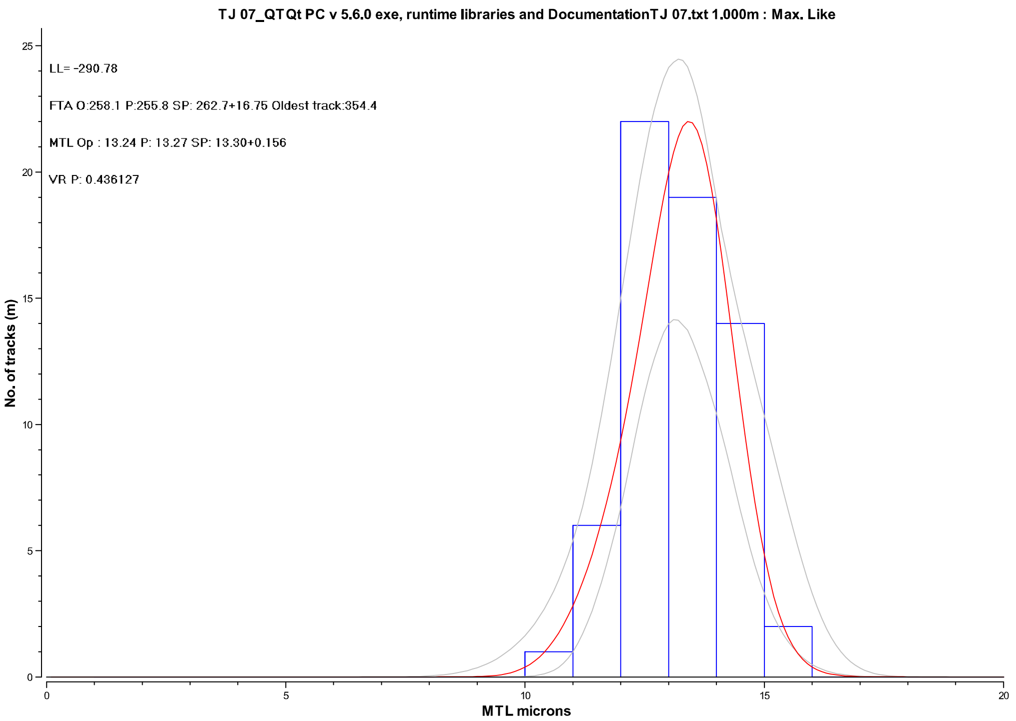
TJ 07_QTQt PC v 5.6.0 exe, runtime libraries and DocumentationTJ 07.txt 1.000m : Max. Like
LL= -290.78
FTA O:258.1 P:255.8 SP: 262.7+16.75 Oldest track:354.4
MTL Op : 13.24 P: 13.27 SP: 13.30+0.156
VR P: 0.436127
No. of tracks (m)
MTL microns

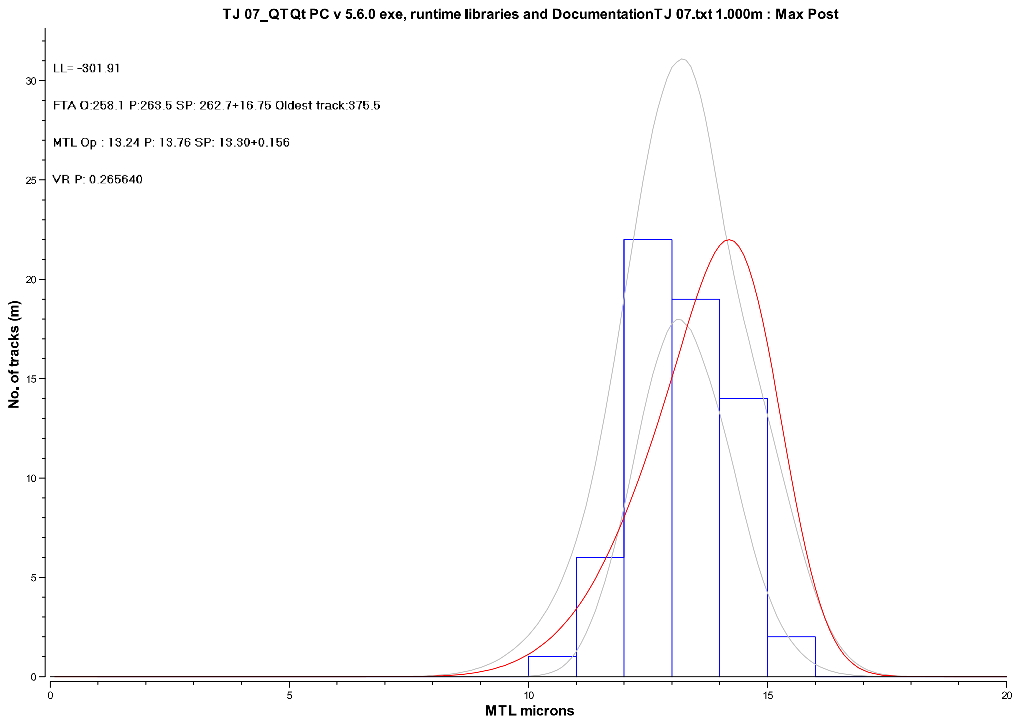
TJ 07_QTQt PC v 5.6.0 exe, runtime libraries and DocumentationTJ 07.txt 1.000m : Max Post
LL= -301.91
FTA O:258.1 P:263.5 SP: 262.7+16.75 Oldest track:375.5
MTL Op : 13.24 P: 13.76 SP: 13.30+0.156
VR P: 0.265640
No. of tracks (m)
MTL microns

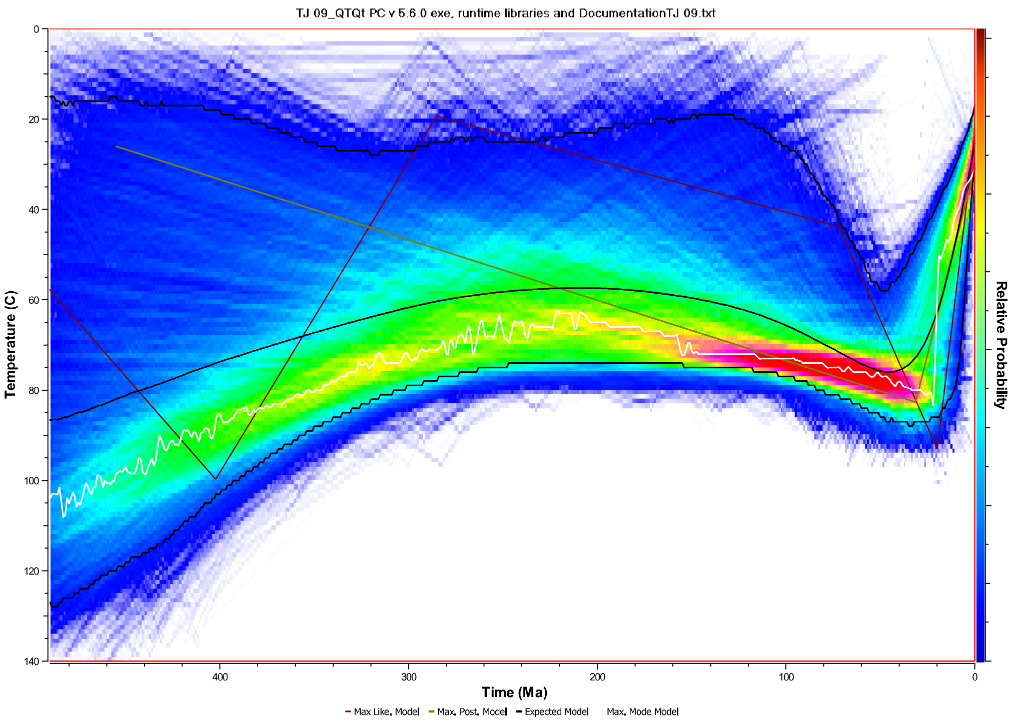
TJ 09_QTQt PC v 5.6.0 exe, runtime libraries and DocumentationTJ 09.txt
Temperature (C)
0
20
40
60
80
100
120
140
400
300
200
100
0
Time (Ma)
Relative Probability
Max Like. Model
Max. Post. Model
Expected Model
Max. Mode Model

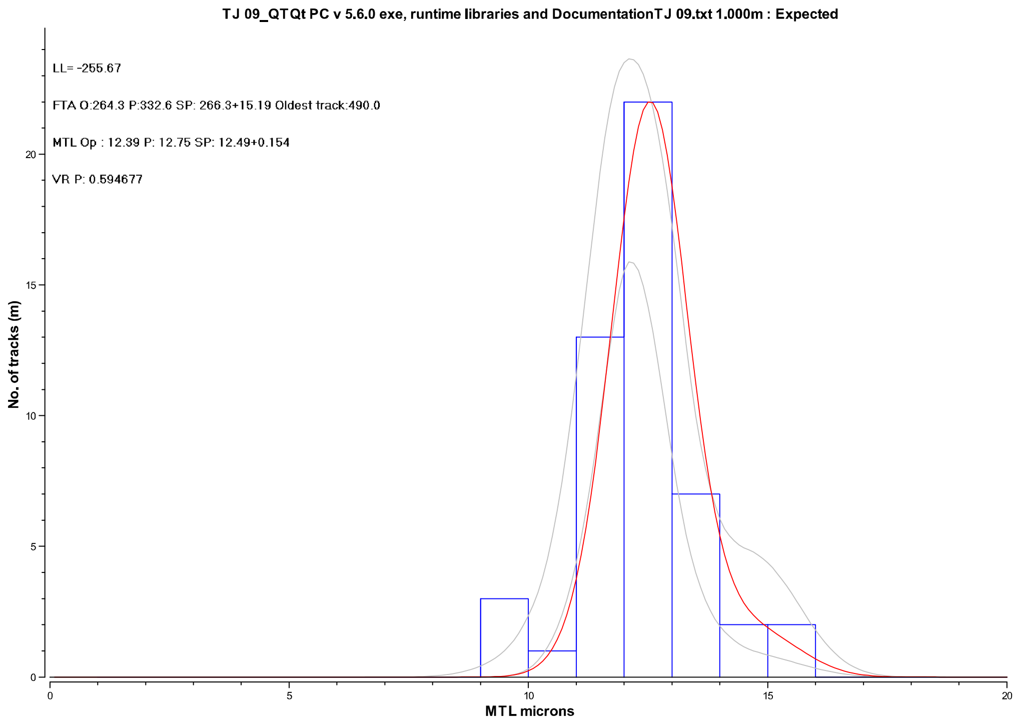
TJ 09_QTQt PC v 5.6.0 exe, runtime libraries and DocumentationTJ 09.txt 1.000m : Expected
LL= -255.67
FTA O:264.3 P:332.6 SP: 266.3+15.19 Oldest track:490.0
MTL Op : 12.39 P: 12.75 SP: 12.49+0.154
VR P: 0.594677
No. of tracks (m)
0
5
10
15
20
MTL microns

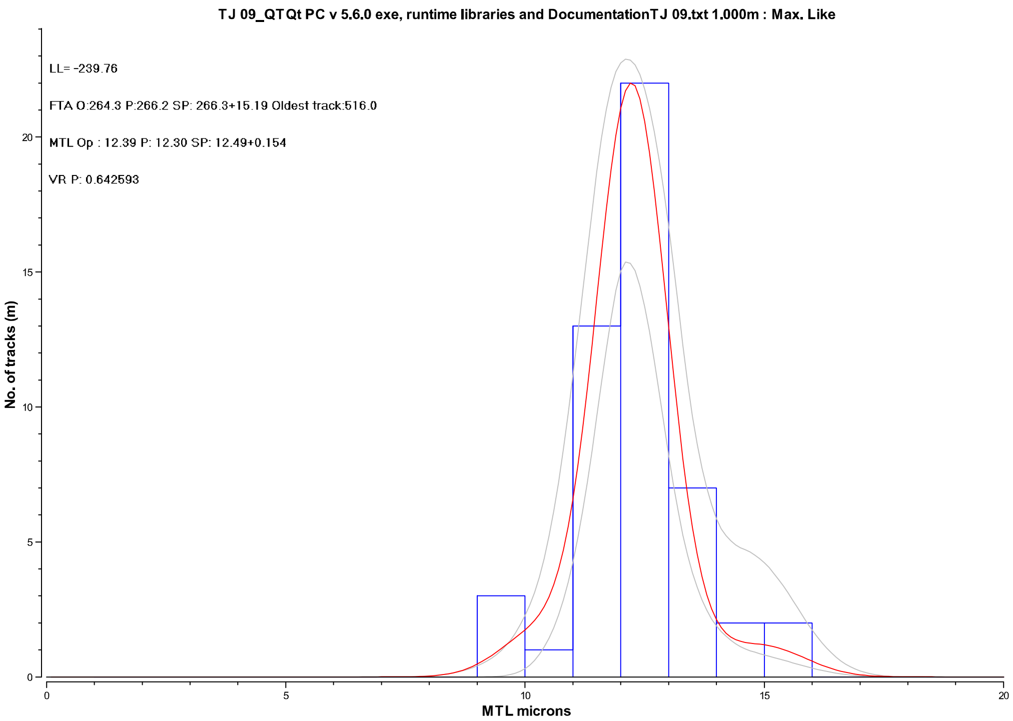
TJ 09_QTQt PC v 5.6.0 exe, runtime libraries and DocumentationTJ 09.txt 1.000m : Max. Like
LL= -239.76
FTA O:264.3 P:266.2 SP: 266.3+15.19 Oldest track:516.0
MTL Op : 12.39 P: 12.30 SP: 12.49+0.154
VR P: 0.642593
No. of tracks (m)
0
5
10
15
20
MTL microns

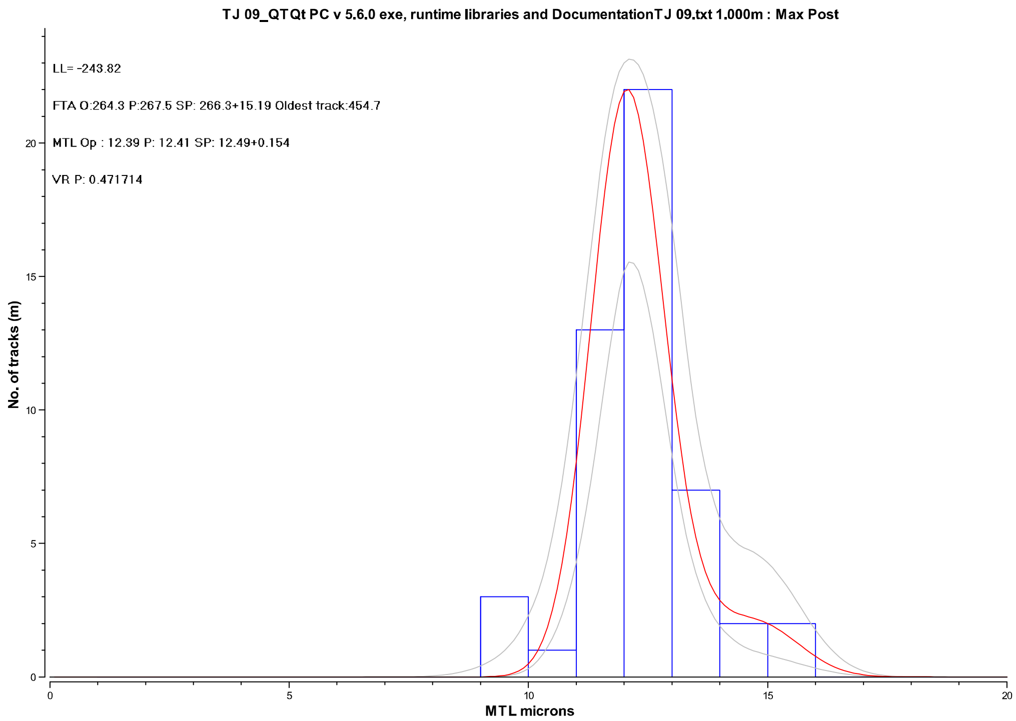
TJ 09_QTQt PC v 5.6.0 exe, runtime libraries and DocumentationTJ 09.txt 1.000m : Max Post
LL= -243.82
FTA O:264.3 P:267.5 SP: 266.3+15.19 Oldest track:454.7
MTL Op : 12.39 P: 12.41 SP: 12.49+0.154
VR P: 0.471714
No. of tracks (m)
0
5
10
15
20
MTL microns

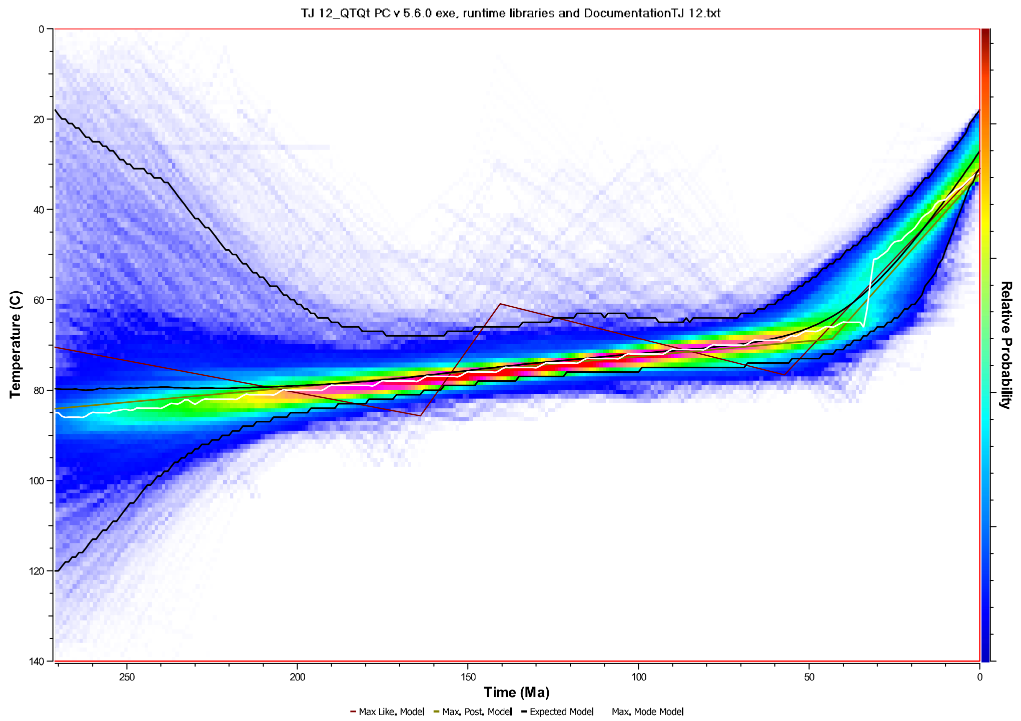
TJ 12_QTQt PC v 5.6.0 exe, runtime libraries and DocumentationTJ 12.txt
Temperature (C)
Time (Ma)
Relative Probability
Max Like. Model
Max. Post. Model
Expected Model
Max. Mode Model

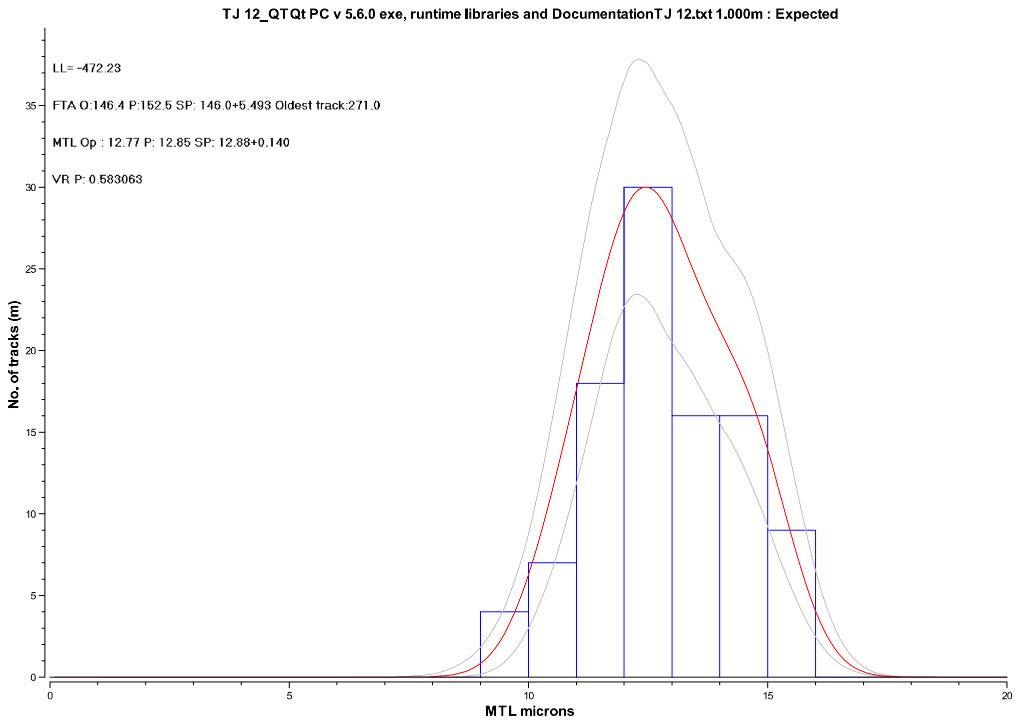
TJ 12_QTQt PC v 5.6.0 exe, runtime libraries and DocumentationTJ 12.txt 1.000m : Expected
LL= -472.23
FTA O:146.4 P:152.5 SP: 146.0+5.493 Oldest track:271.0
MTL Op : 12.77 P: 12.85 SP: 12.88+0.140
VR P: 0.583063
No. of tracks (m)
MTL microns

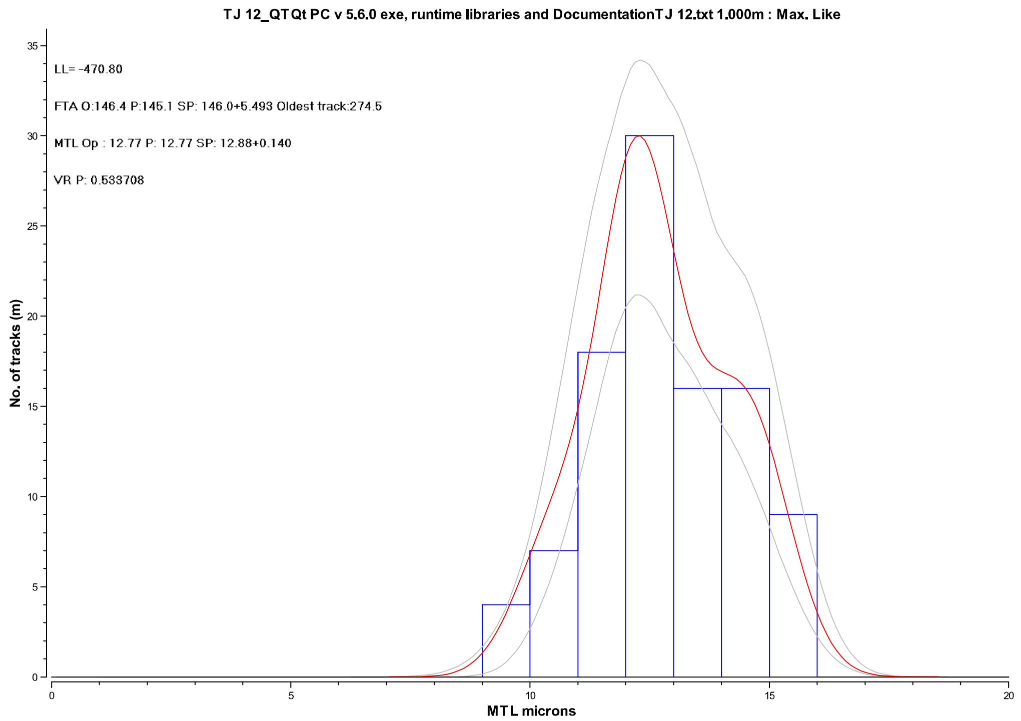
TJ 12_QTQt PC v 5.6.0 exe, runtime libraries and DocumentationTJ 12.txt 1.000m : Max. Like
LL= -470.80
FTA O:146.4 P:145.1 SP: 146.0+5.493 Oldest track:274.5
MTL Op : 12.77 P: 12.77 SP: 12.88+0.140
VR P: 0.533708
No. of tracks (m)
MTL microns

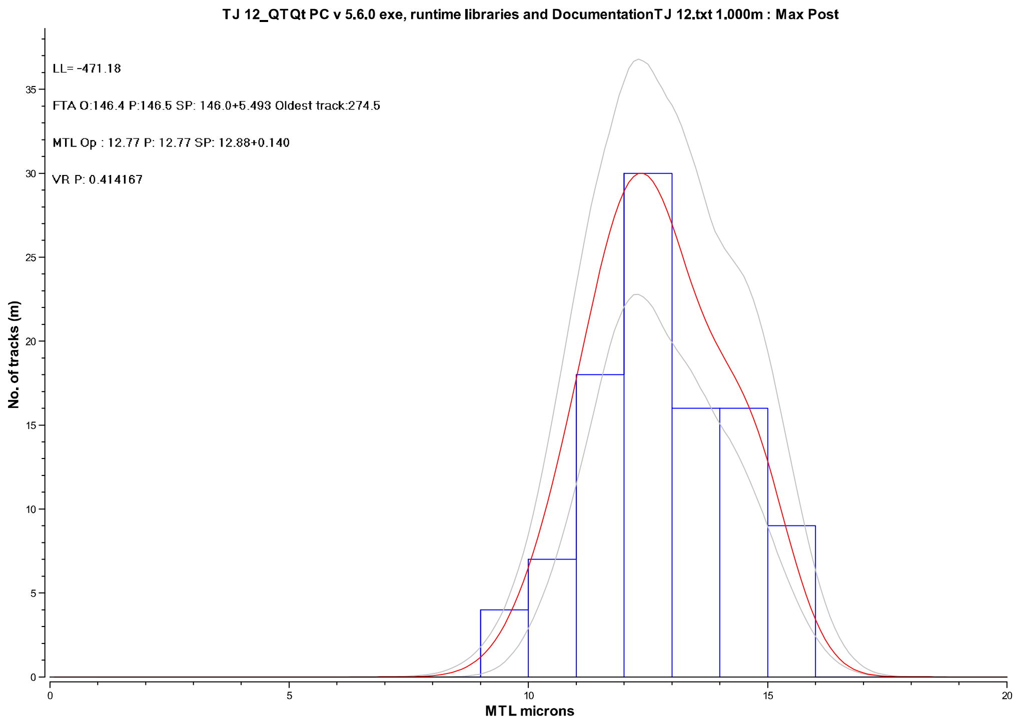
TJ 12_QTQt PC v 5.6.0 exe, runtime libraries and DocumentationTJ 12.txt 1.000m : Max Post
LL= -471.18
FTA O:146.4 P:146.5 SP: 146.0+5.493 Oldest track:274.5
MTL Op : 12.77 P: 12.77 SP: 12.88+0.140
VR P: 0.414167
No. of tracks (m)
MTL microns

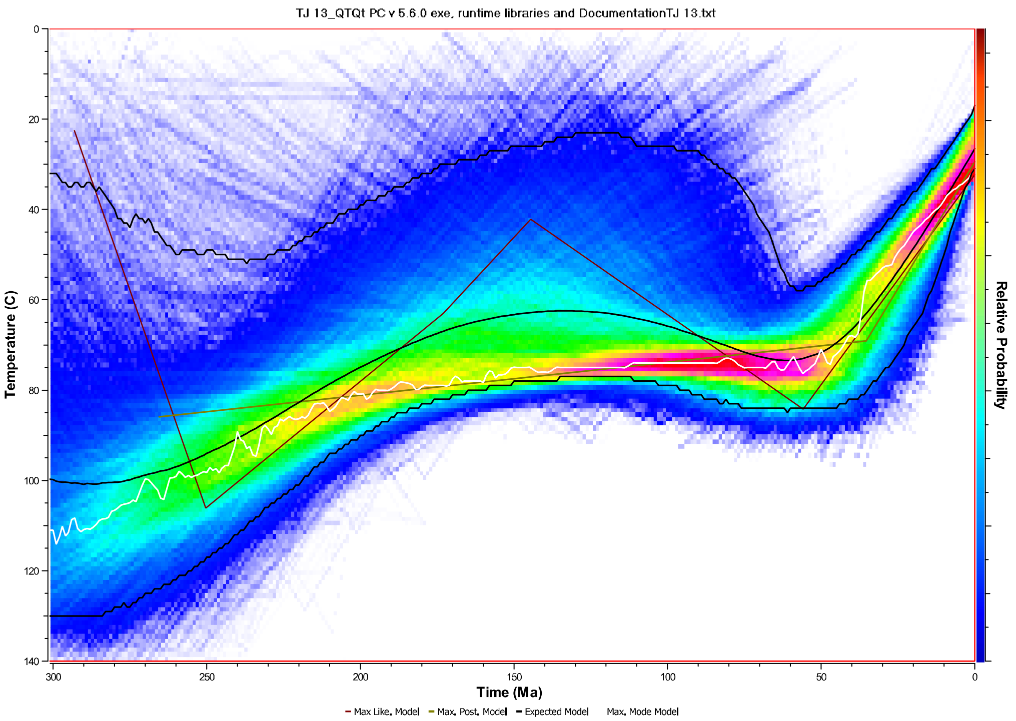
TJ 13_QTQt PC v 5.6.0 exe, runtime libraries and DocumentationTJ 13.txt
Temperature (C)
Time (Ma)
Relative Probability
Max Like. Model
Max. Post. Model
Expected Model
Max. Mode Model

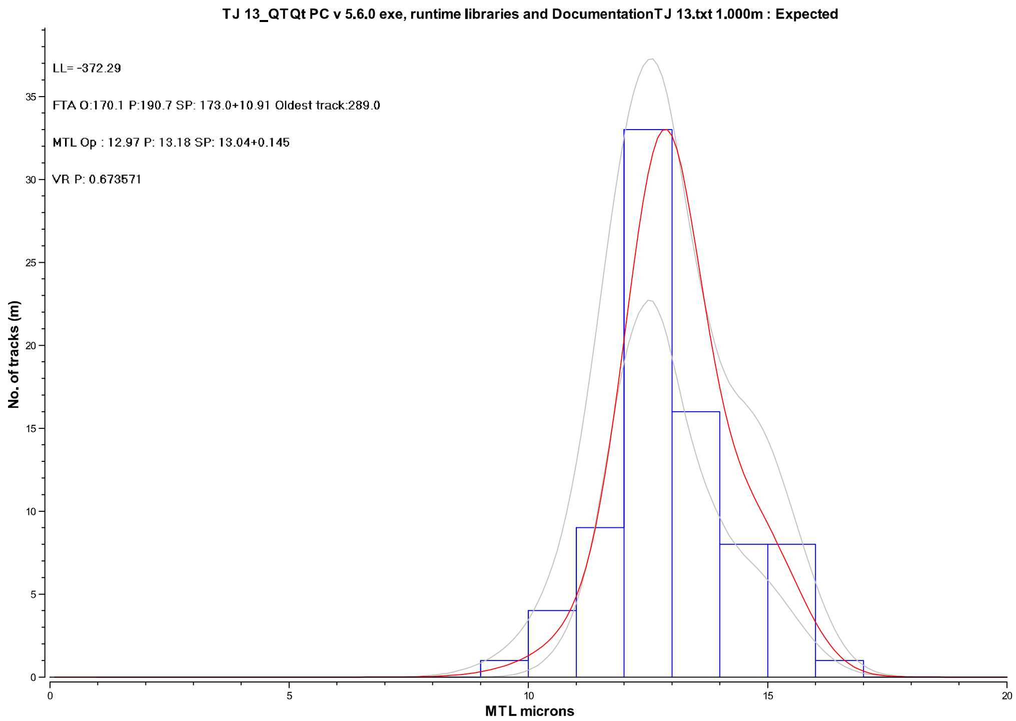
TJ 13_QTQt PC v 5.6.0 exe, runtime libraries and DocumentationTJ 13.txt 1.000m : Expected
LL= -372.29
FTA O:170.1 P:190.7 SP: 173.0+10.91 Oldest track:289.0
MTL Op : 12.97 P: 13.18 SP: 13.04+0.145
VR P: 0.673571
No. of tracks (m)
MTL microns

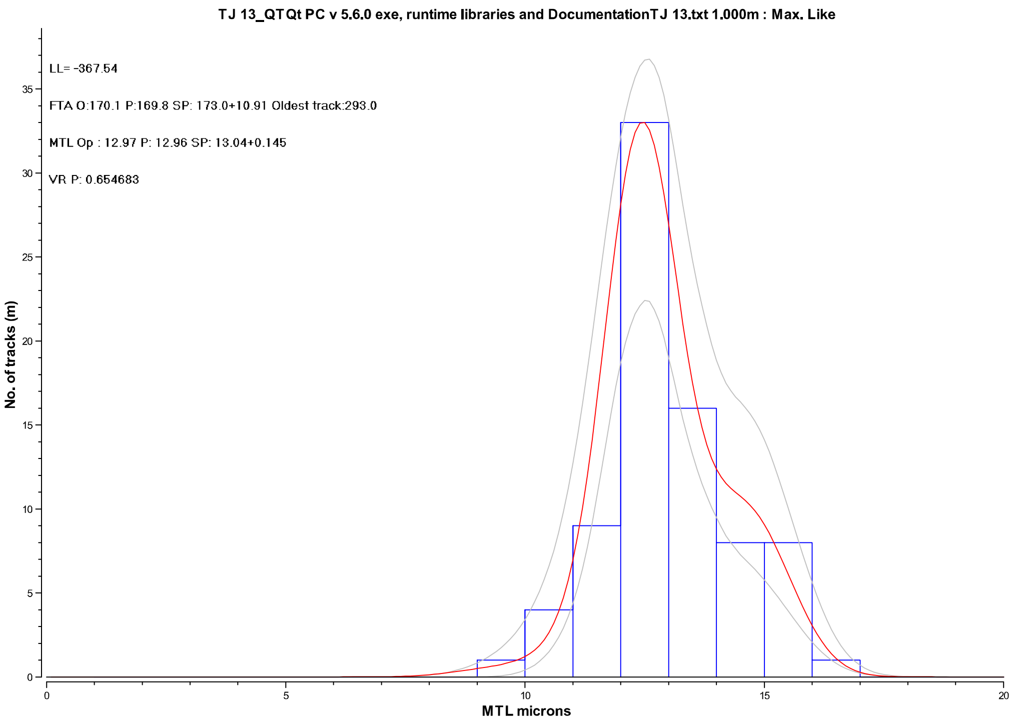
TJ 13_QTQt PC v 5.6.0 exe, runtime libraries and DocumentationTJ 13.txt 1.000m : Max. Like
LL= -367.54
FTA O:170.1 P:169.8 SP: 173.0+10.91 Oldest track:293.0
MTL Op : 12.97 P: 12.96 SP: 13.04+0.145
VR P: 0.654683
No. of tracks (m)
MTL microns

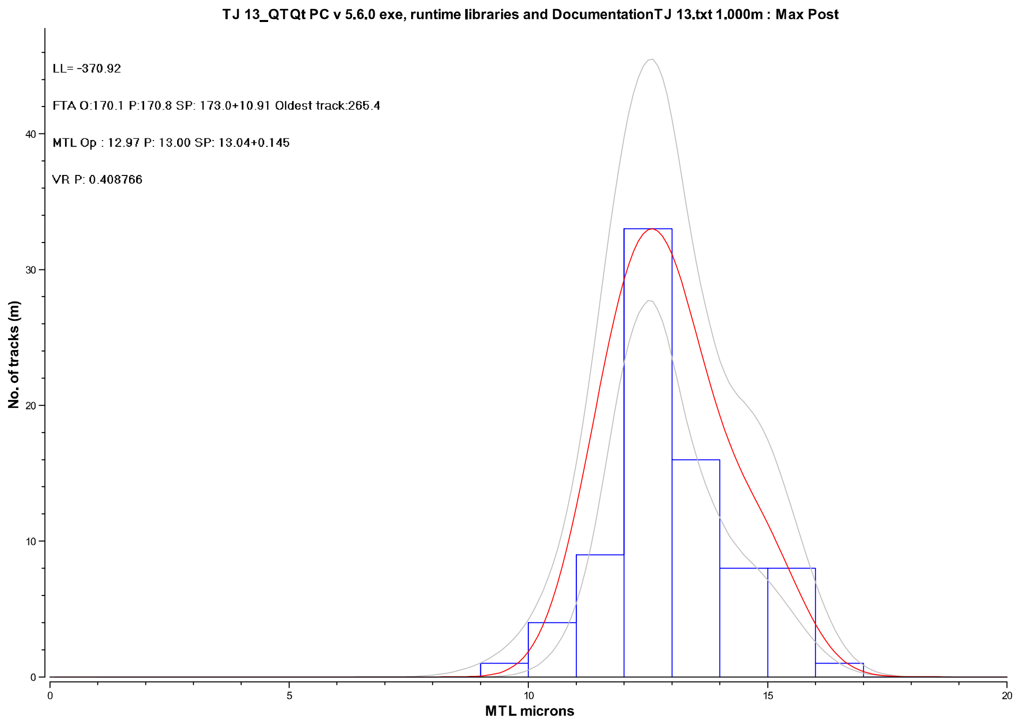
TJ 13_QTQt PC v 5.6.0 exe, runtime libraries and DocumentationTJ 13.txt 1.000m : Max Post
LL= -370.92
FTA O:170.1 P:170.8 SP: 173.0+10.91 Oldest track:265.4
MTL Op : 12.97 P: 13.00 SP: 13.04+0.145
VR P: 0.408766
No. of tracks (m)
MTL microns

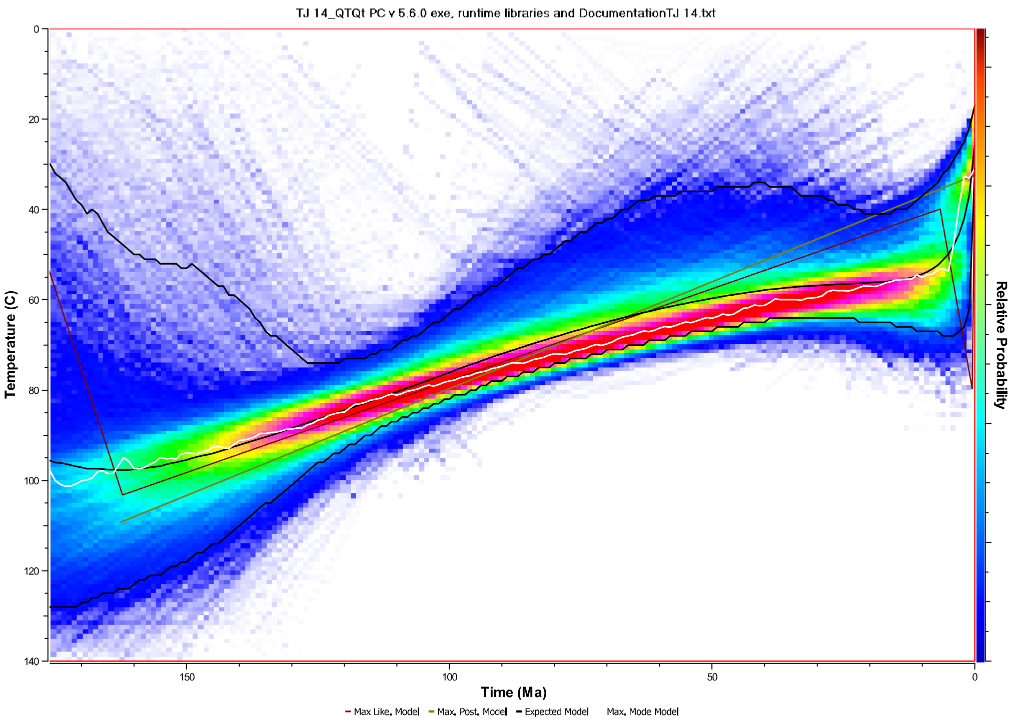
TJ 14_QTQt PC v 5.6.0 exe, runtime libraries and DocumentationTJ 14.txt
Temperature (C)
Time (Ma)
Relative Probability
Max Like. Model
Max. Post. Model
Expected Model
Max. Mode Model

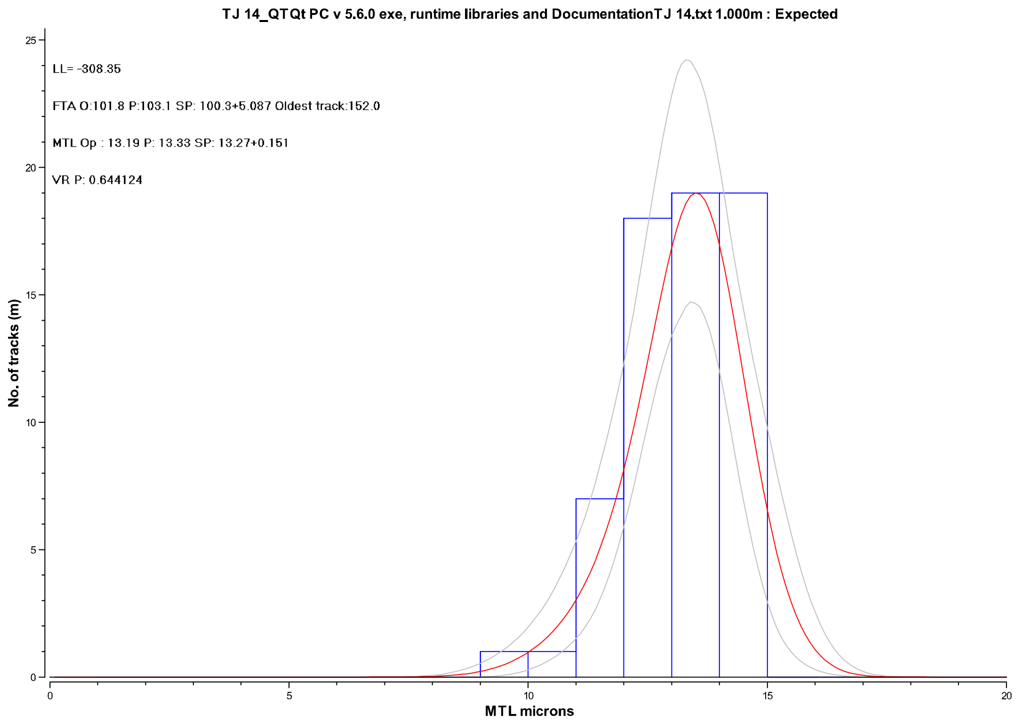
TJ 14_QTQt PC v 5.6.0 exe, runtime libraries and DocumentationTJ 14.txt 1.000m : Expected
LL= -308.35
FTA O:101.8 P:103.1 SP: 100.3+5.087 Oldest track:152.0
MTL Op : 13.19 P: 13.33 SP: 13.27+0.151
VR P: 0.644124
No. of tracks (m)
MTL microns

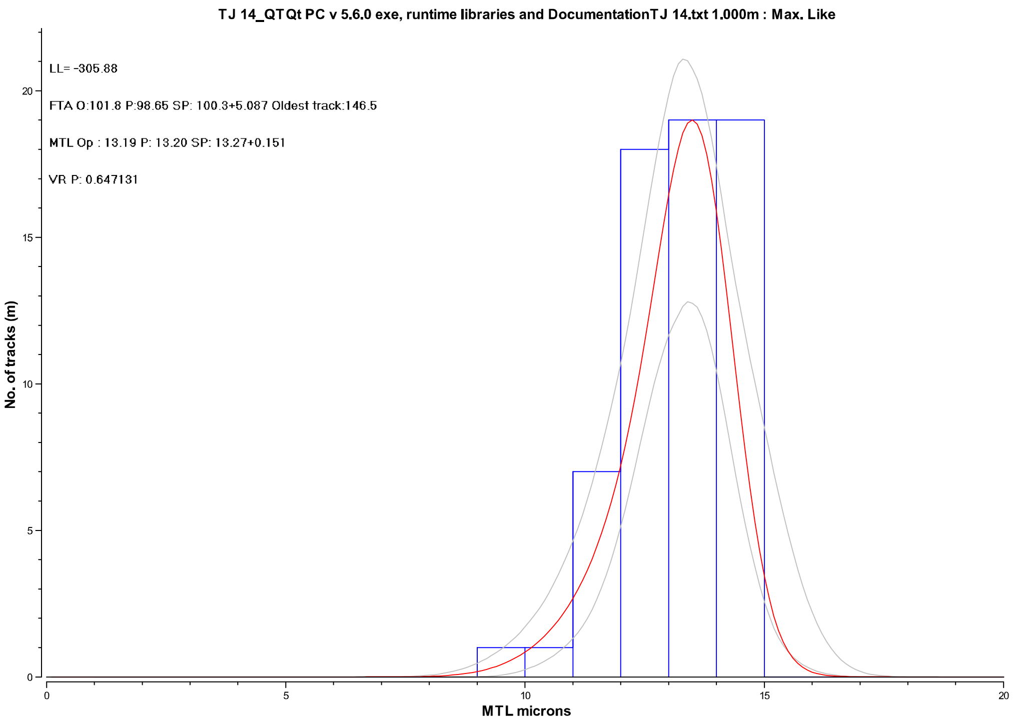
TJ 14_QTQt PC v 5.6.0 exe, runtime libraries and DocumentationTJ 14.txt 1.000m : Max. Like
LL= -305.88
FTA O:101.8 P:98.65 SP: 100.3+5.087 Oldest track:146.5
MTL Op : 13.19 P: 13.20 SP: 13.27+0.151
VR P: 0.647131
No. of tracks (m)
MTL microns

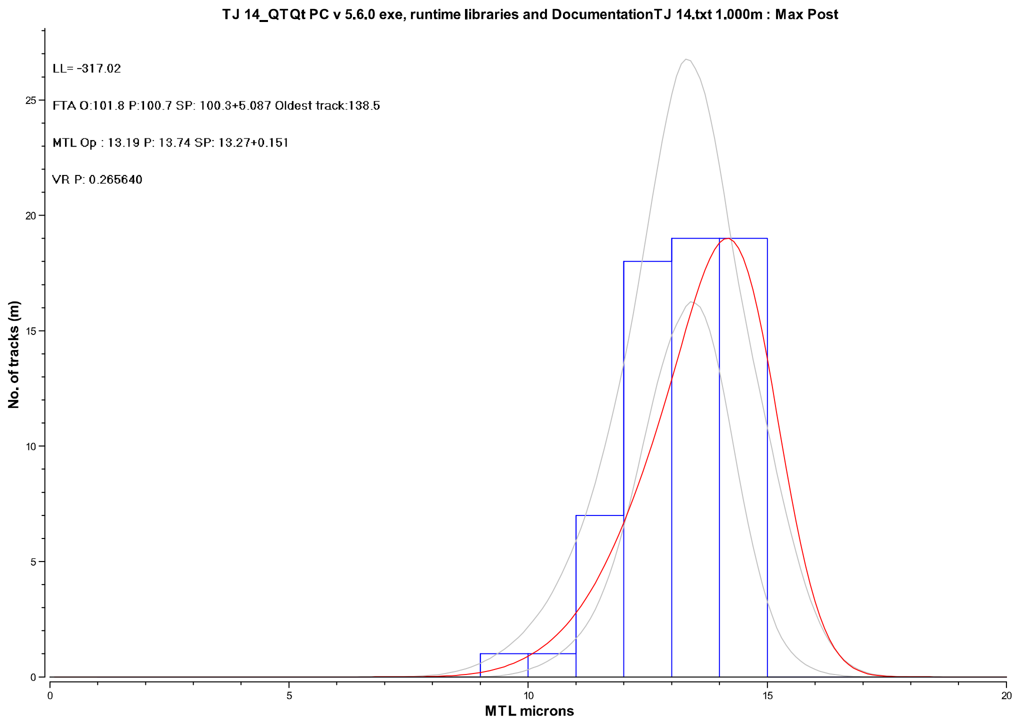
TJ 14_QTQt PC v 5.6.0 exe, runtime libraries and DocumentationTJ 14.txt 1.000m : Max Post
LL= -317.02
FTA O:101.8 P:100.7 SP: 100.3+5.087 Oldest track:138.5
MTL Op : 13.19 P: 13.74 SP: 13.27+0.151
VR P: 0.265640
No. of tracks (m)
MTL microns

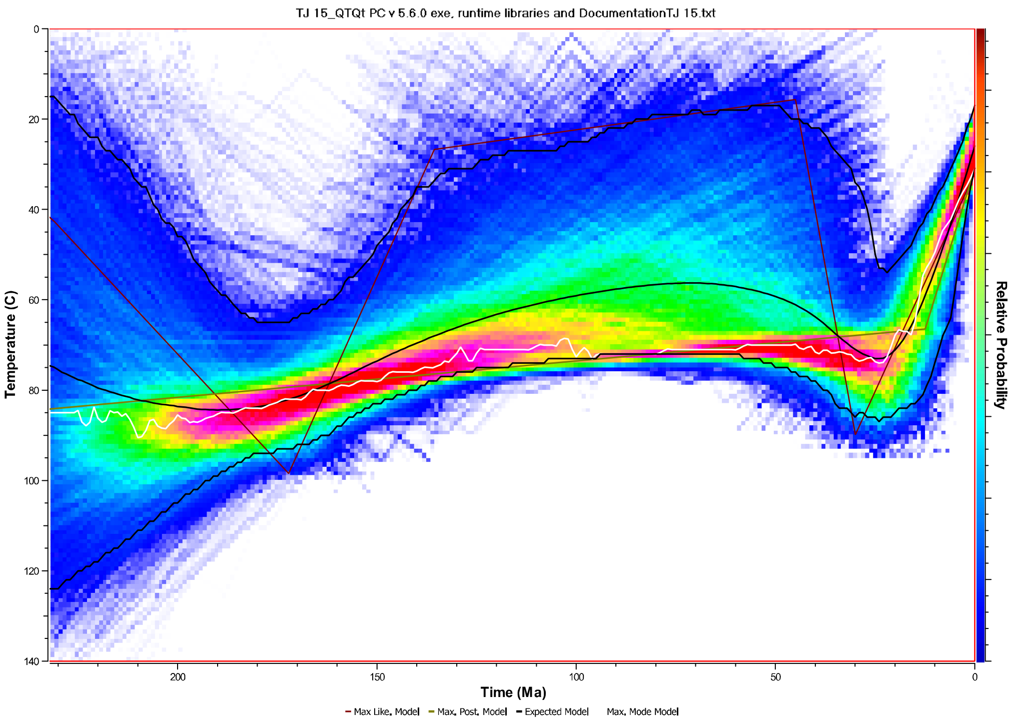
TJ 15_QTQt PC v 5.6.0 exe, runtime libraries and DocumentationTJ 15.txt
Temperature (C)
Time (Ma)
Relative Probability
Max Like. Model
Max. Post. Model
Expected Model
Max. Mode Model

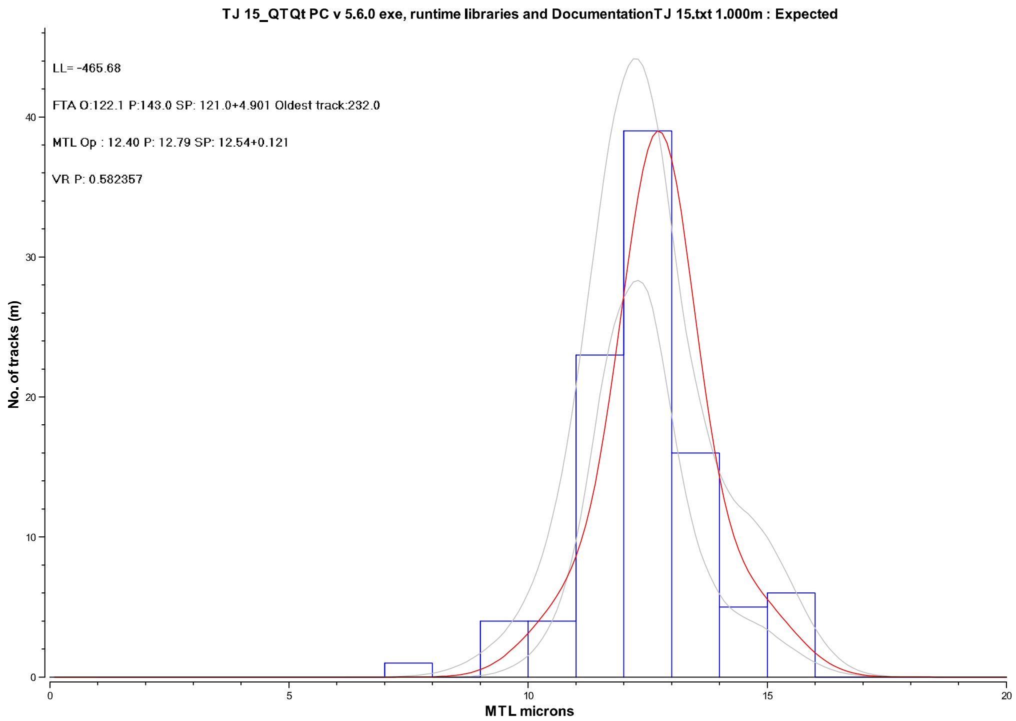
TJ 15_QTQt PC v 5.6.0 exe, runtime libraries and DocumentationTJ 15.txt 1.000m : Expected
LL= -465.68
FTA O:122.1 P:143.0 SP: 121.0+4.901 Oldest track:232.0
MTL Op : 12.40 P: 12.79 SP: 12.54+0.121
VR P: 0.582357
No. of tracks (m)
MTL microns

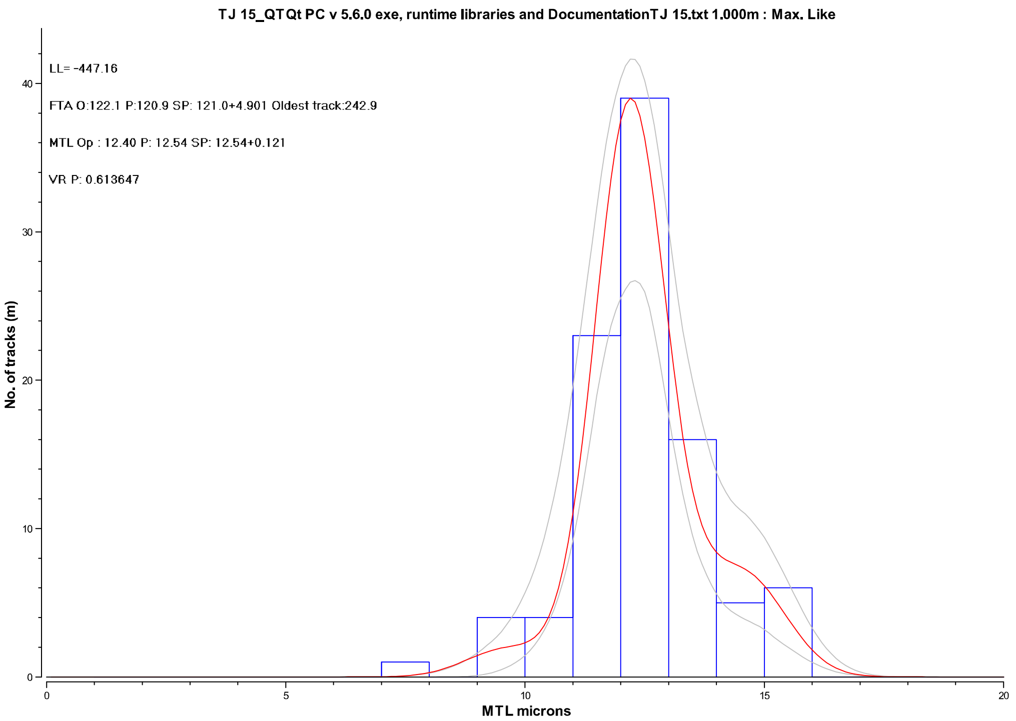
TJ 15_QTQt PC v 5.6.0 exe, runtime libraries and DocumentationTJ 15.txt 1.000m : Max. Like
LL= -447.16
FTA O:122.1 P:120.9 SP: 121.0+4.901 Oldest track:242.9
MTL Op : 12.40 P: 12.54 SP: 12.54+0.121
VR P: 0.613647
No. of tracks (m)
MTL microns

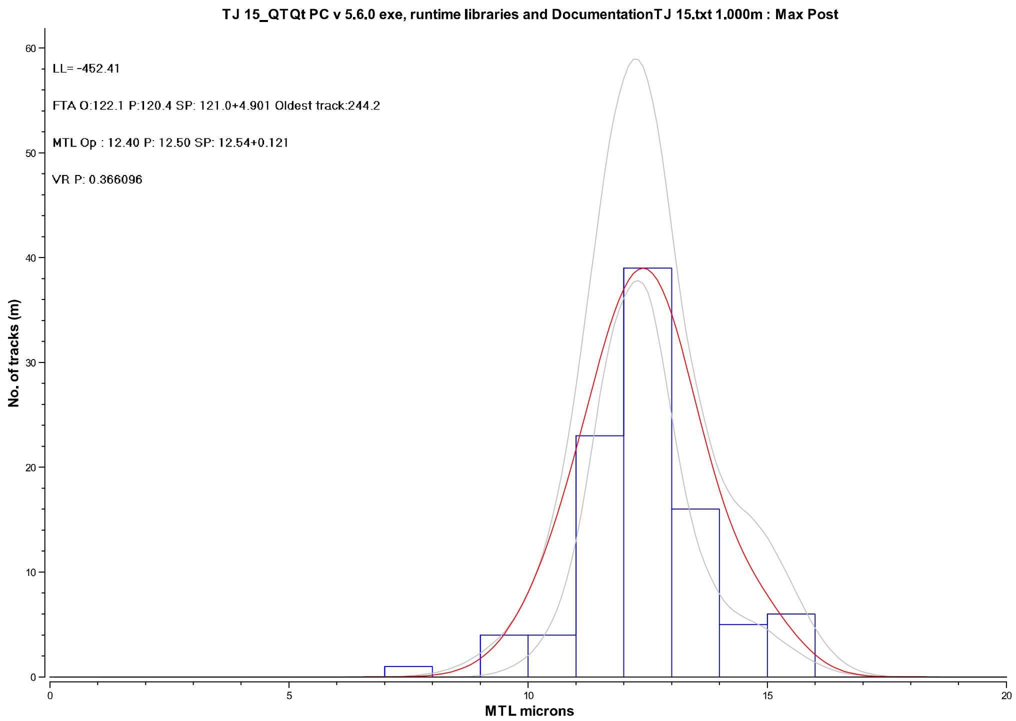
TJ 15_QTQt PC v 5.6.0 exe, runtime libraries and DocumentationTJ 15.txt 1.000m : Max Post
LL= -452.41
FTA O:122.1 P:120.4 SP: 121.0+4.901 Oldest track:244.2
MTL Op : 12.40 P: 12.50 SP: 12.54+0.121
VR P: 0.366096
No. of tracks (m)
MTL microns

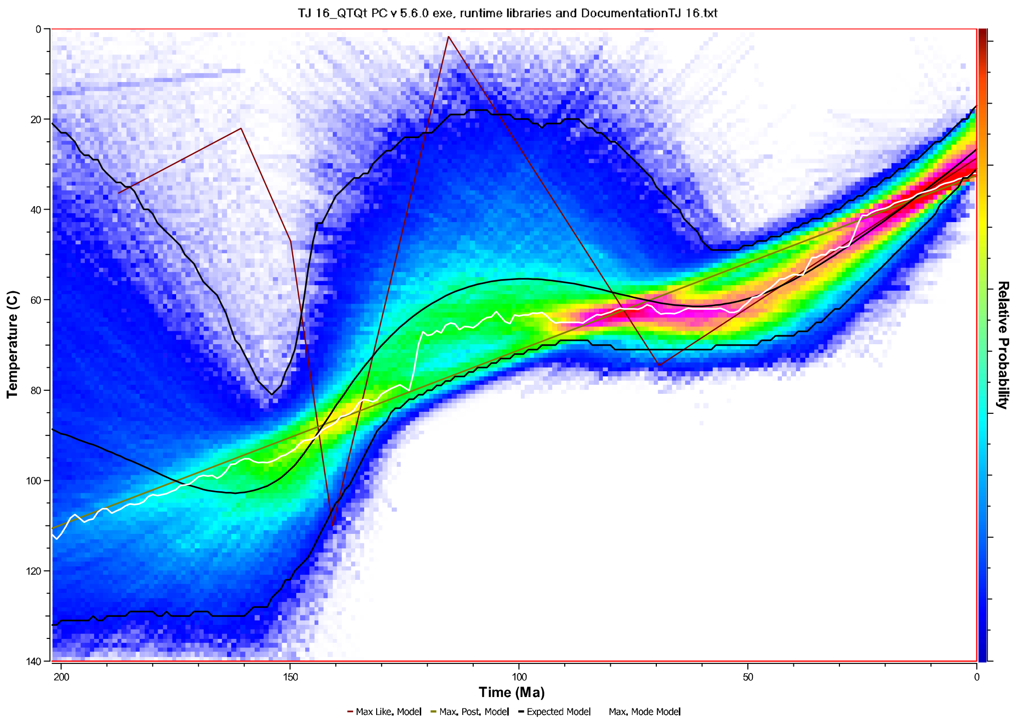
TJ 16_QTQt PC v 5.6.0 exe, runtime libraries and DocumentationTJ 16.txt
Temperature (C)
Time (Ma)
Relative Probability
0
20
40
60
80
100
120
140
200
150
100
50
0
Max Like. Model
Max. Post. Model
Expected Model
Max. Mode Model

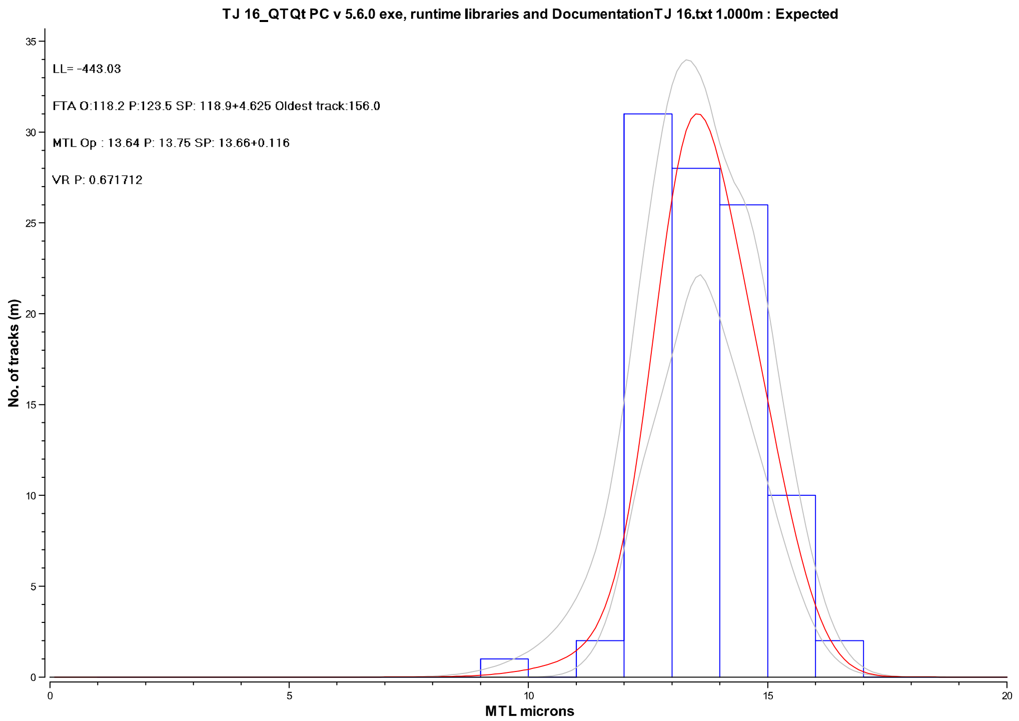
TJ 16_QTQt PC v 5.6.0 exe, runtime libraries and DocumentationTJ 16.txt 1.000m : Expected
LL= -443.03
FTA O:118.2 P:123.5 SP: 118.9+4.625 Oldest track:156.0
MTL Op : 13.64 P: 13.75 SP: 13.66+0.116
VR P: 0.671712
No. of tracks (m)
MTL microns
0
5
10
15
20
25
30
35

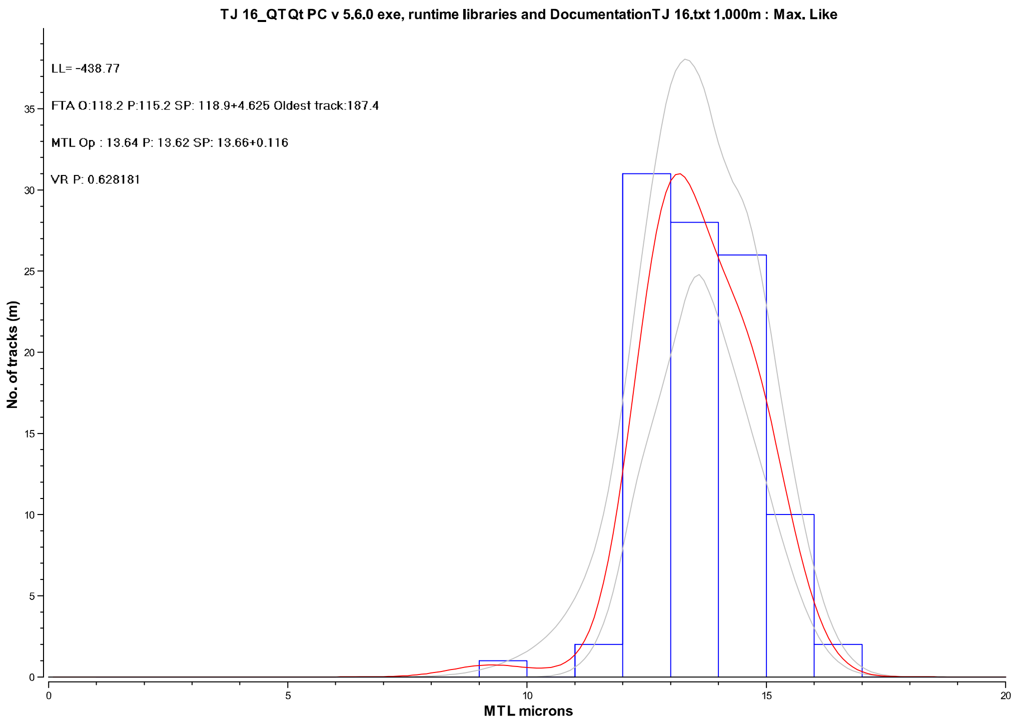
TJ 16_QTQt PC v 5.6.0 exe, runtime libraries and DocumentationTJ 16.txt 1.000m : Max. Like
LL= -438.77
FTA O:118.2 P:115.2 SP: 118.9+4.625 Oldest track:187.4
MTL Op : 13.64 P: 13.62 SP: 13.66+0.116
VR P: 0.628181
No. of tracks (m)
MTL microns
0
5
10
15
20
25
30
35

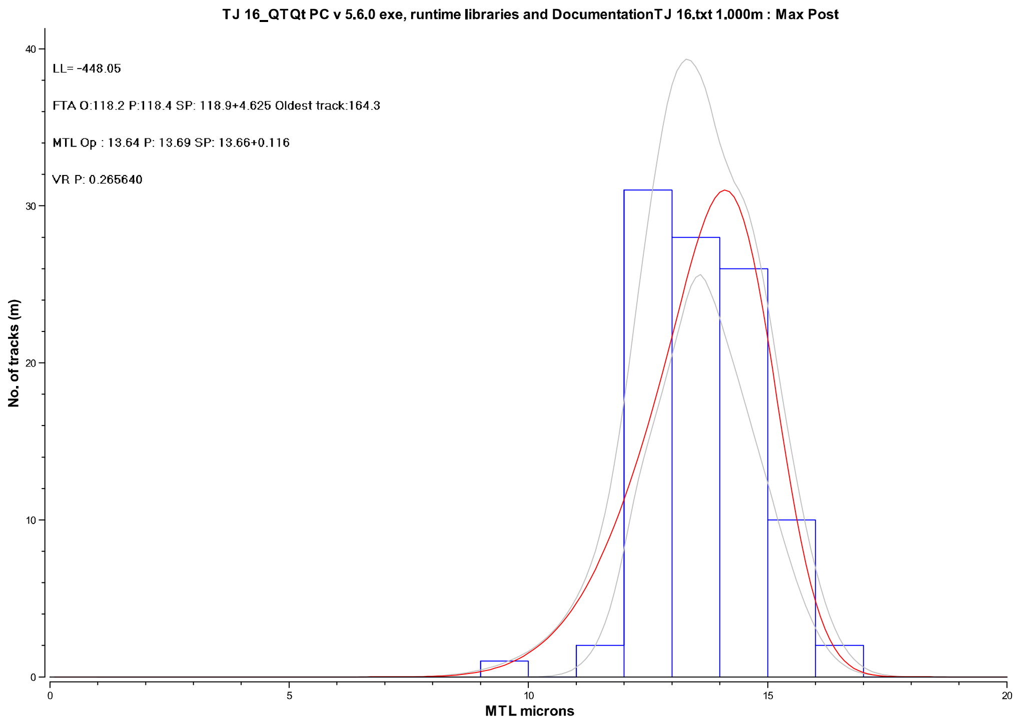
TJ 16_QTQt PC v 5.6.0 exe, runtime libraries and DocumentationTJ 16.txt 1.000m : Max Post
LL= -448.05
FTA O:118.2 P:118.4 SP: 118.9+4.625 Oldest track:164.3
MTL Op : 13.64 P: 13.69 SP: 13.66+0.116
VR P: 0.265640
No. of tracks (m)
MTL microns
0
5
10
15
20
30
40

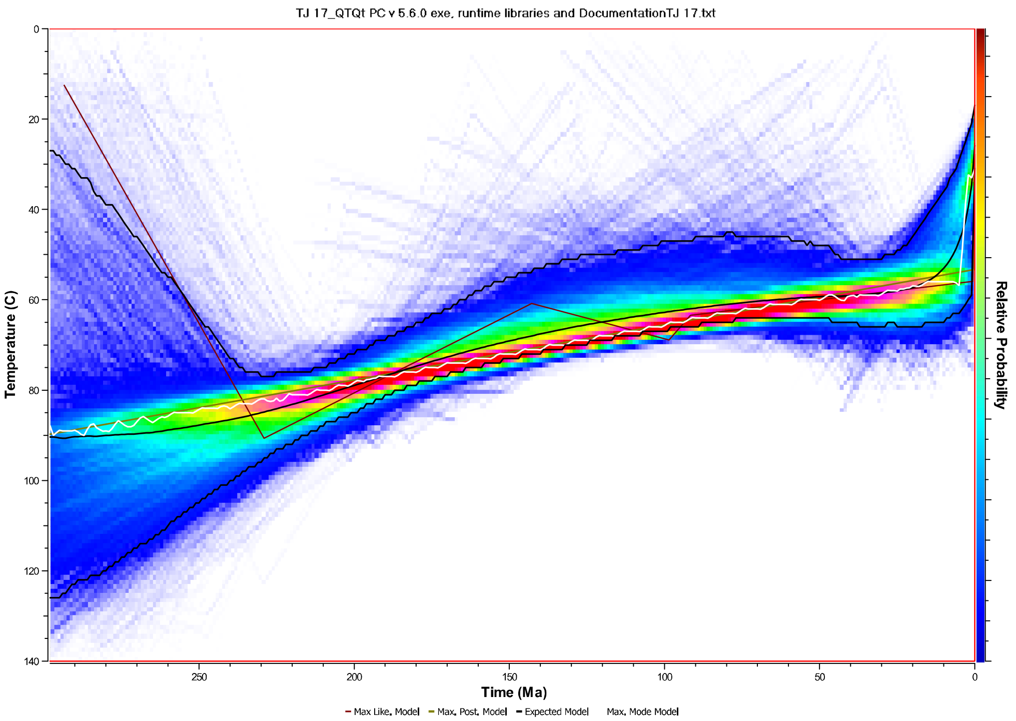
TJ 17_QTQt PC v 5.6.0 exe, runtime libraries and DocumentationTJ 17.txt
Temperature (C)
Time (Ma)
Relative Probability
Max Like. Model
Max. Post. Model
Expected Model
Max. Mode Model

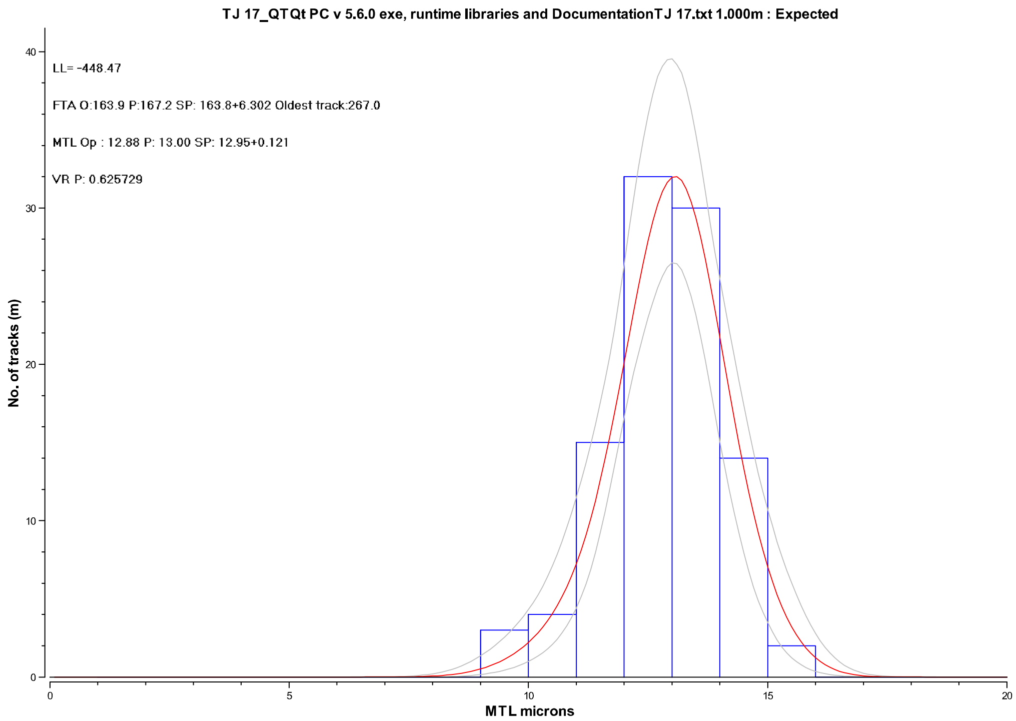
TJ 17_QTQt PC v 5.6.0 exe, runtime libraries and DocumentationTJ 17.txt 1.000m : Expected
LL= -448.47
FTA O:163.9 P:167.2 SP: 163.8+6.302 Oldest track:267.0
MTL Op : 12.88 P: 13.00 SP: 12.95+0.121
VR P: 0.625729
No. of tracks (m)
MTL microns

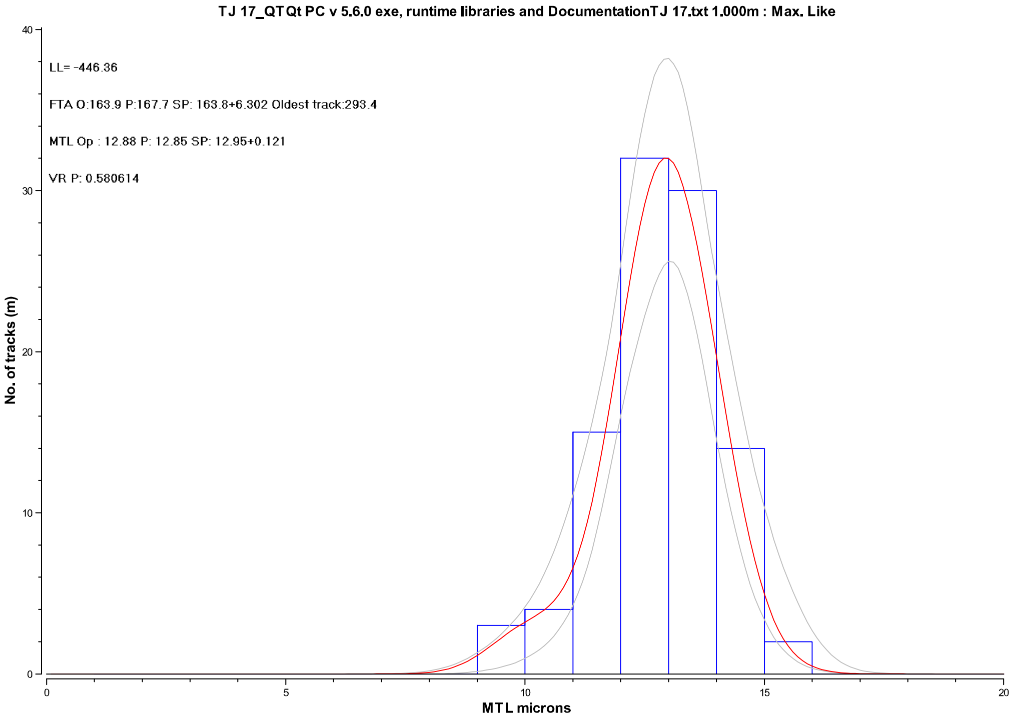
TJ 17_QTQt PC v 5.6.0 exe, runtime libraries and DocumentationTJ 17.txt 1.000m : Max. Like
LL= -446.36
FTA O:163.9 P:167.7 SP: 163.8+6.302 Oldest track:293.4
MTL Op : 12.88 P: 12.85 SP: 12.95+0.121
VR P: 0.580614
No. of tracks (m)
MTL microns

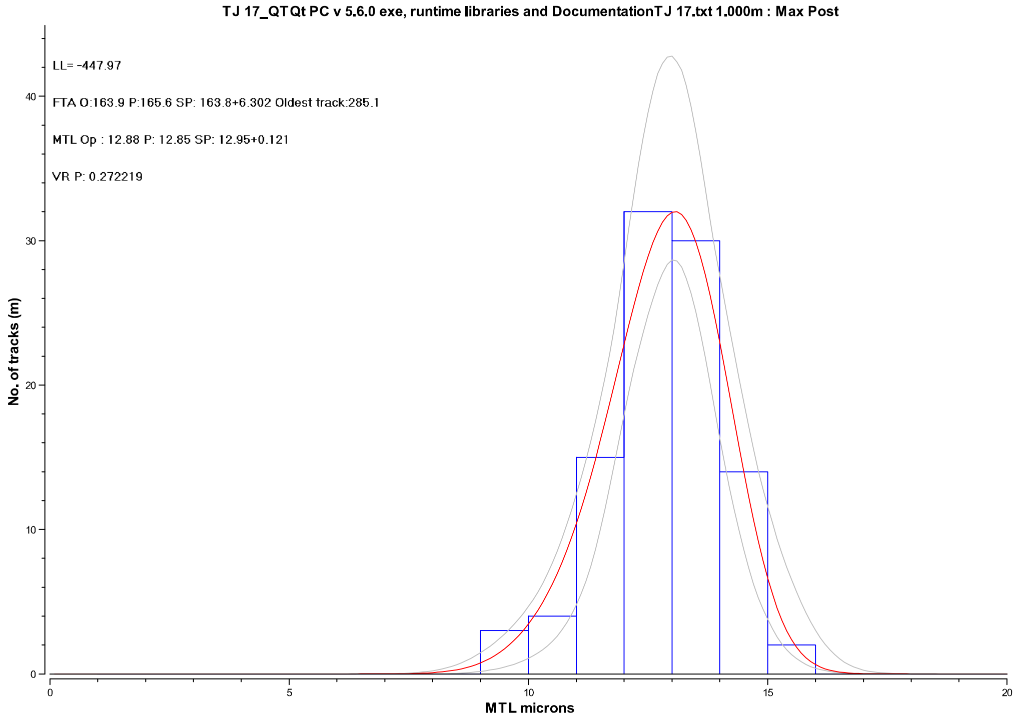
TJ 17_QTQt PC v 5.6.0 exe, runtime libraries and DocumentationTJ 17.txt 1.000m : Max Post
LL= -447.97
FTA O:163.9 P:165.6 SP: 163.8+6.302 Oldest track:285.1
MTL Op : 12.88 P: 12.85 SP: 12.95+0.121
VR P: 0.272219
No. of tracks (m)
MTL microns

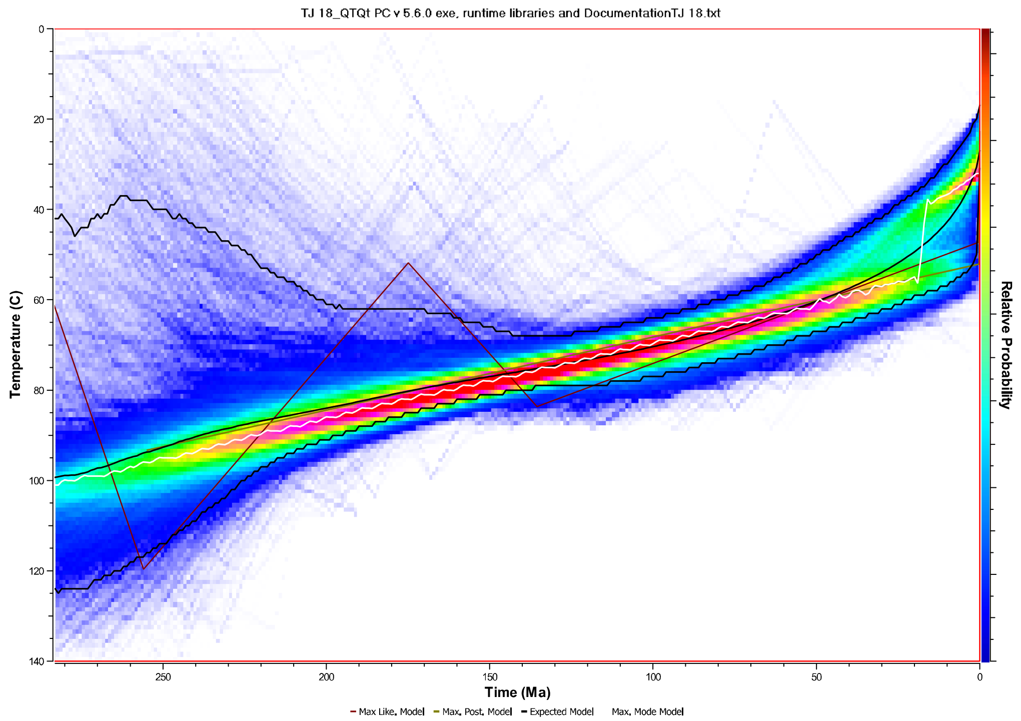
TJ 18_QTQt PC v 5.6.0 exe, runtime libraries and DocumentationTJ 18.txt
Temperature (C)
Time (Ma)
Relative Probability
Max Like. Model
Max. Post. Model
Expected Model
Max. Mode Model

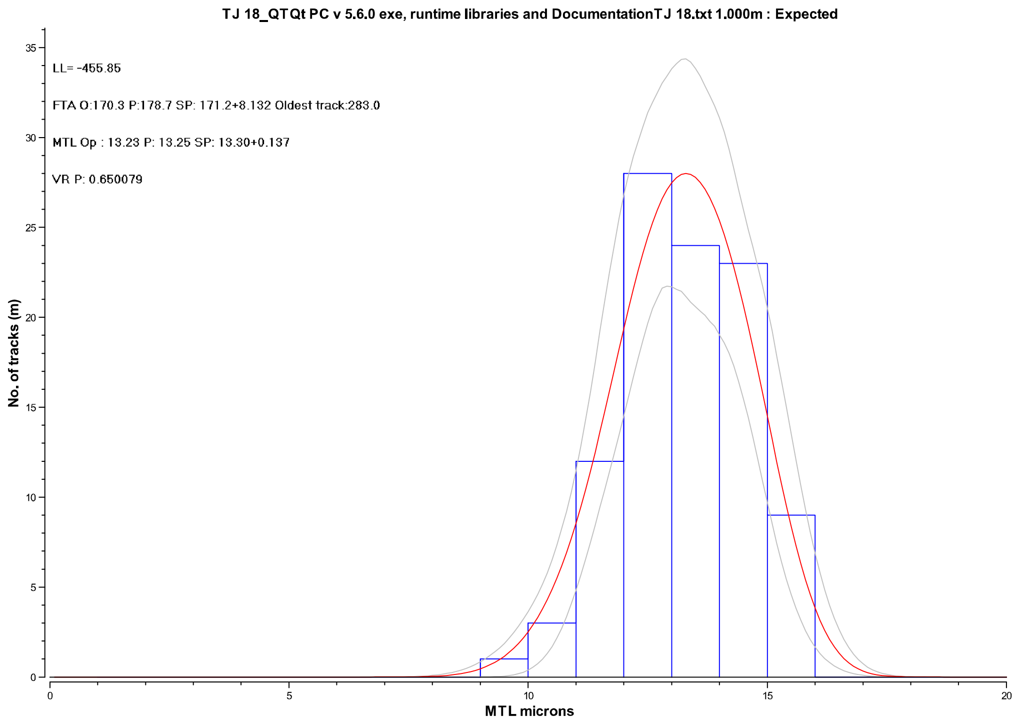
TJ 18_QTQt PC v 5.6.0 exe, runtime libraries and DocumentationTJ 18.txt 1.000m : Expected
LL= -455.85
FTA O:170.3 P:178.7 SP: 171.2+8.132 Oldest track:283.0
MTL Op : 13.23 P: 13.25 SP: 13.30+0.137
VR P: 0.650079
No. of tracks (m)
MTL microns

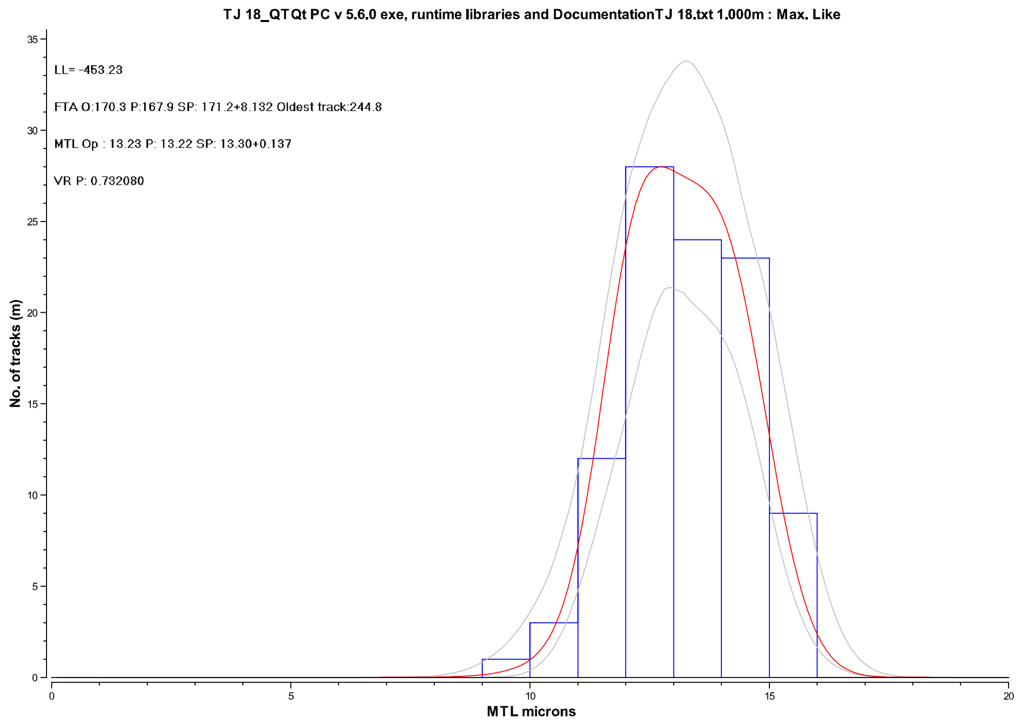
TJ 18_QTQt PC v 5.6.0 exe, runtime libraries and DocumentationTJ 18.txt 1.000m : Max. Like
LL= -453.23
FTA O:170.3 P:167.9 SP: 171.2+8.132 Oldest track:244.8
MTL Op : 13.23 P: 13.22 SP: 13.30+0.137
VR P: 0.732080
No. of tracks (m)
MTL microns

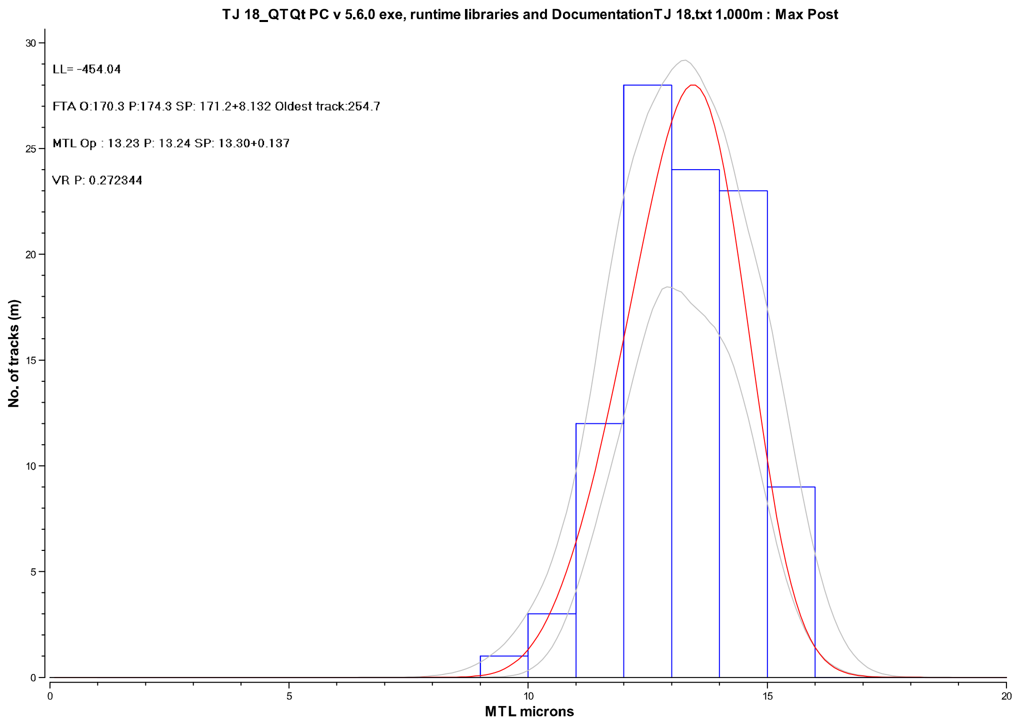
TJ 18_QTQt PC v 5.6.0 exe, runtime libraries and DocumentationTJ 18.txt 1.000m : Max Post
LL= -454.04
FTA O:170.3 P:174.3 SP: 171.2+8.132 Oldest track:254.7
MTL Op : 13.23 P: 13.24 SP: 13.30+0.137
VR P: 0.272344
No. of tracks (m)
MTL microns

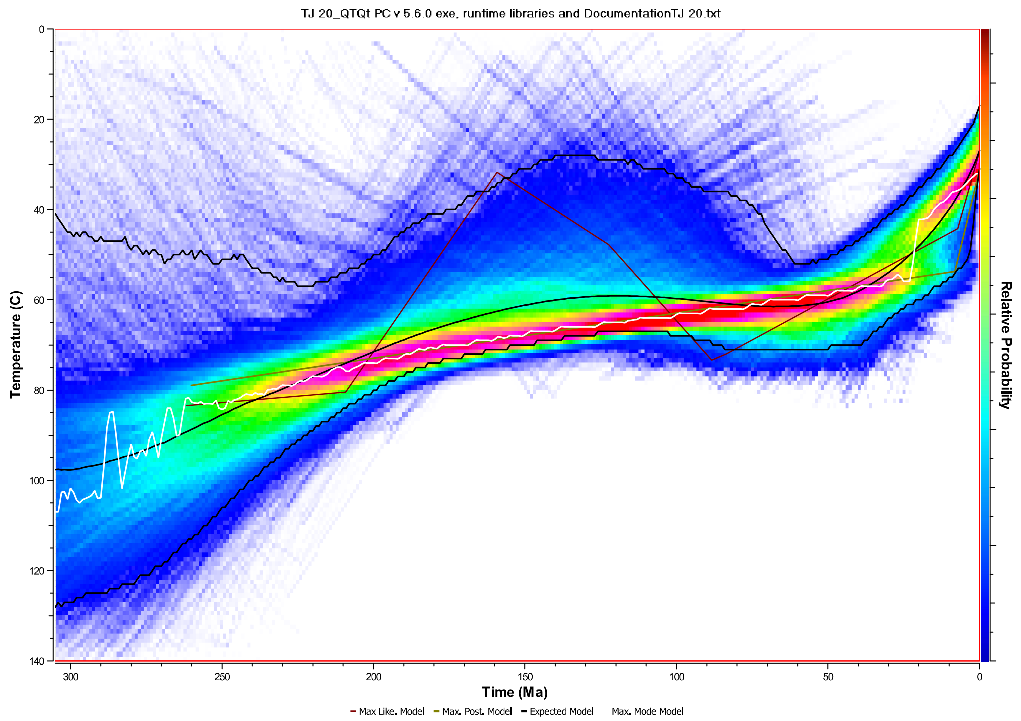

TJ 20_QTQt PC v 5.6.0 exe, runtime libraries and DocumentationTJ 20.txt
Temperature (C)
Time (Ma)
Relative Probability
Max Like. Model
Max. Post. Model
Expected Model
Max. Mode Model

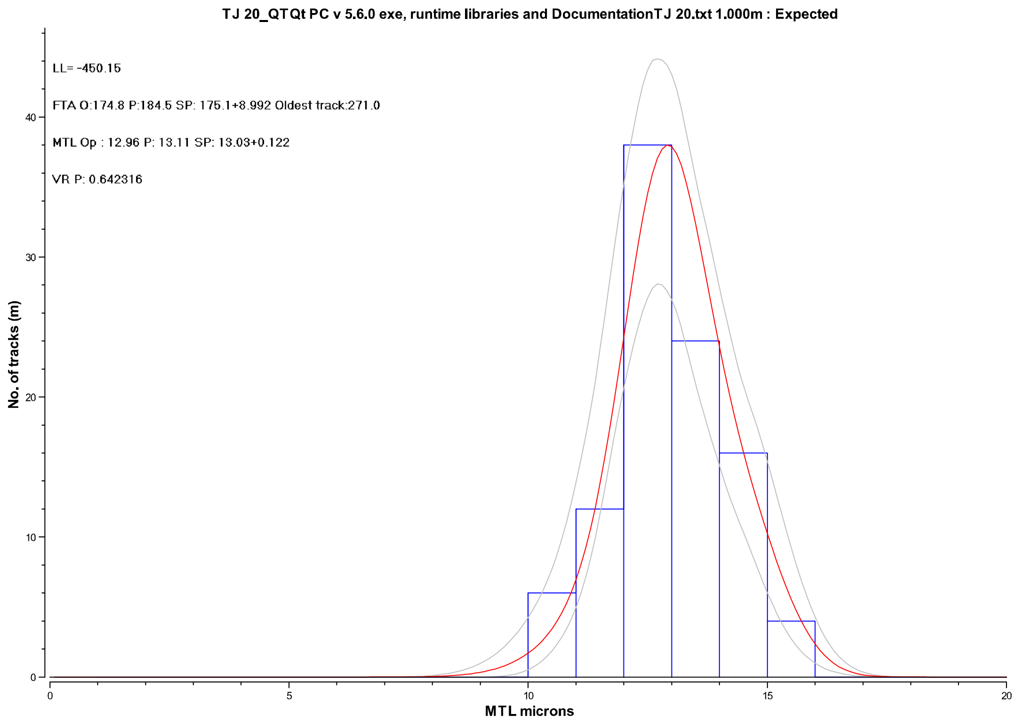

TJ 20_QTQt PC v 5.6.0 exe, runtime libraries and DocumentationTJ 20.txt 1.000m : Expected
LL= -450.15
FTA O:174.8 P:184.5 SP: 175.1+8.992 Oldest track:271.0
MTL Op : 12.96 P: 13.11 SP: 13.03+0.122
VR P: 0.642316
No. of tracks (m)
MTL microns

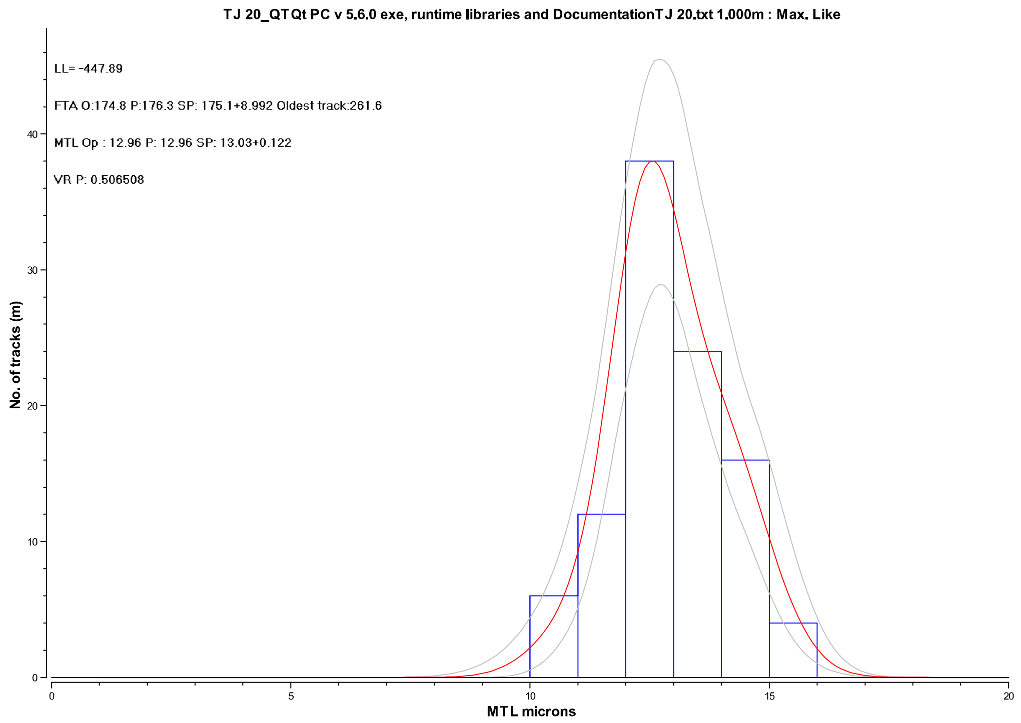

TJ 20_QTQt PC v 5.6.0 exe, runtime libraries and DocumentationTJ 20.txt 1.000m : Max. Like
LL= -447.89
FTA O:174.8 P:176.3 SP: 175.1+8.992 Oldest track:261.6
MTL Op : 12.96 P: 12.96 SP: 13.03+0.122
VR P: 0.506508
No. of tracks (m)
MTL microns

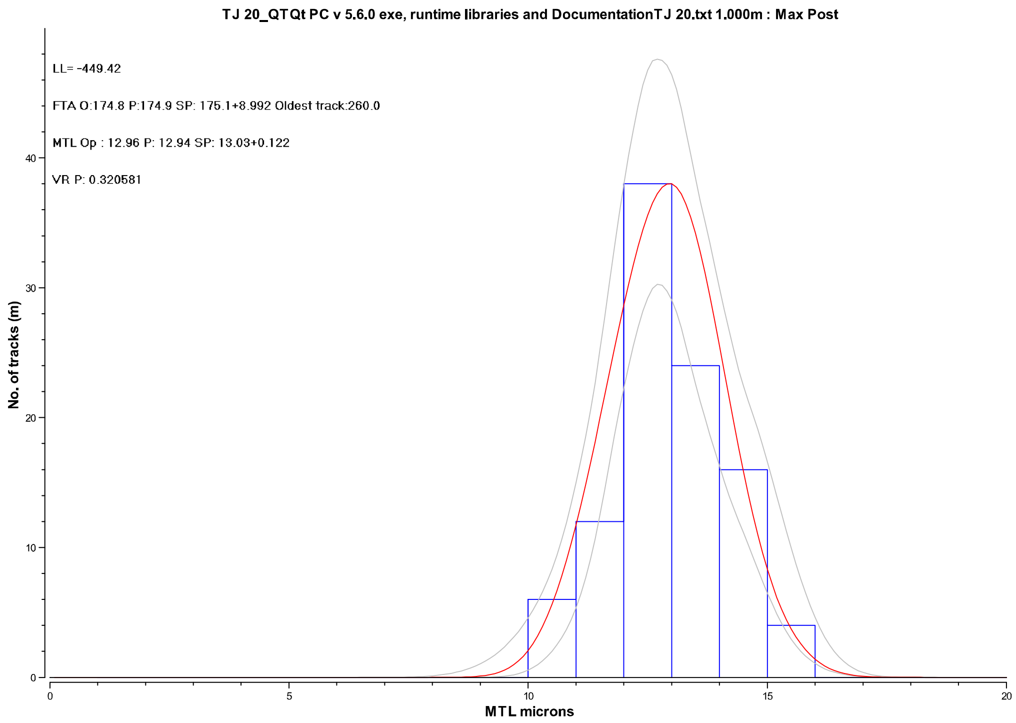

TJ 20_QTQt PC v 5.6.0 exe, runtime libraries and DocumentationTJ 20.txt 1.000m : Max Post
LL= -449.42
FTA O:174.8 P:174.9 SP: 175.1+8.992 Oldest track:260.0
MTL Op : 12.96 P: 12.94 SP: 13.03+0.122
VR P: 0.320581
No. of tracks (m)
MTL microns

TJ 21_QTQt PC v 5.6.0 exe, runtime libraries and DocumentationTJ 21.txt

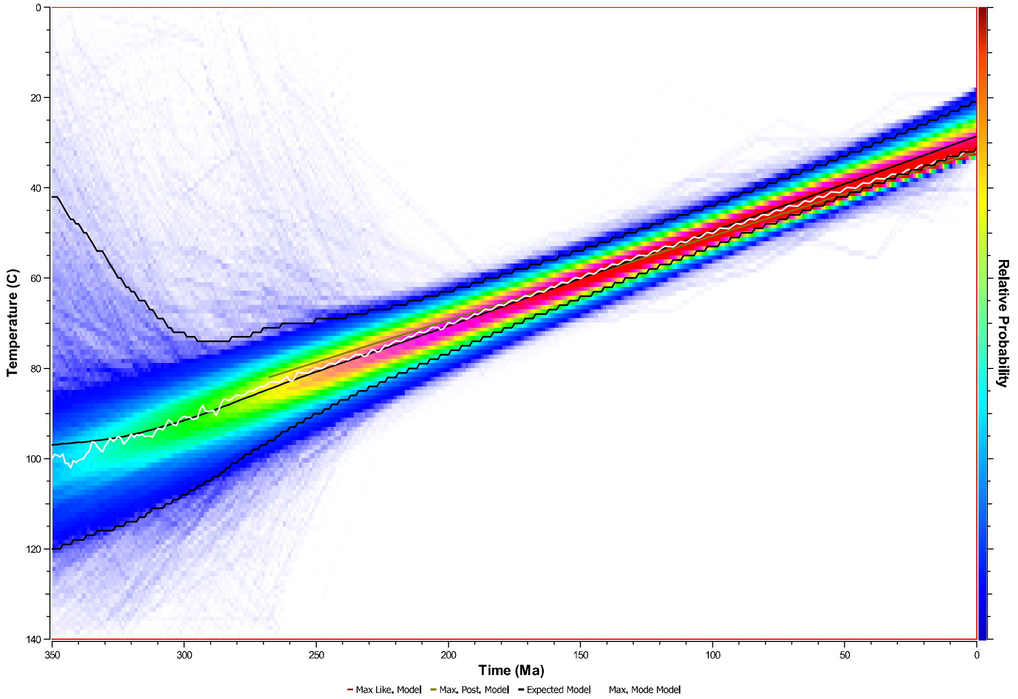

TJ 21_QTQt PC v 5.6.0 exe, runtime libraries and DocumentationTJ 21.txt 1.000m : Expected

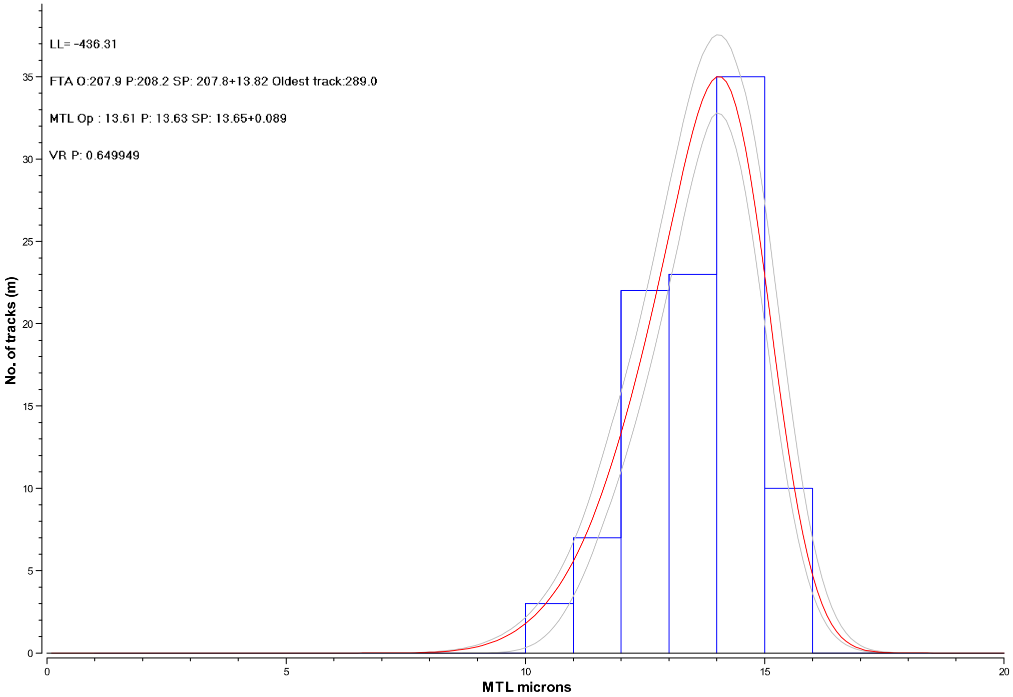

TJ 21_QTQt PC v 5.6.0 exe, runtime libraries and DocumentationTJ 21.txt 1.000m : Max. Like

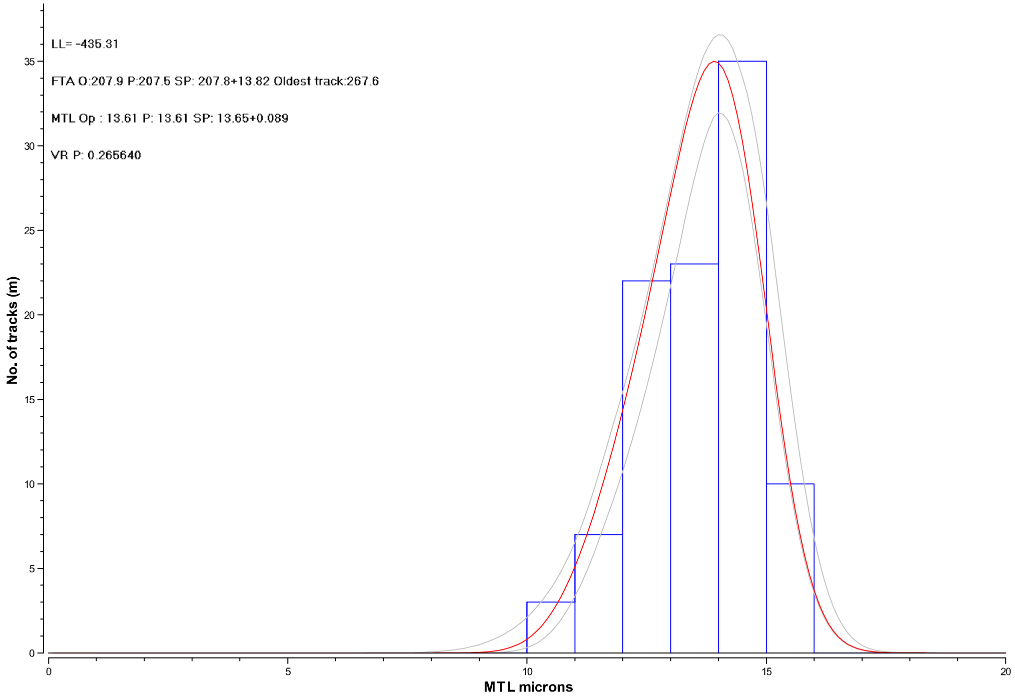

TJ 21_QTQt PC v 5.6.0 exe, runtime libraries and DocumentationTJ 21.txt 1.000m : Max Post

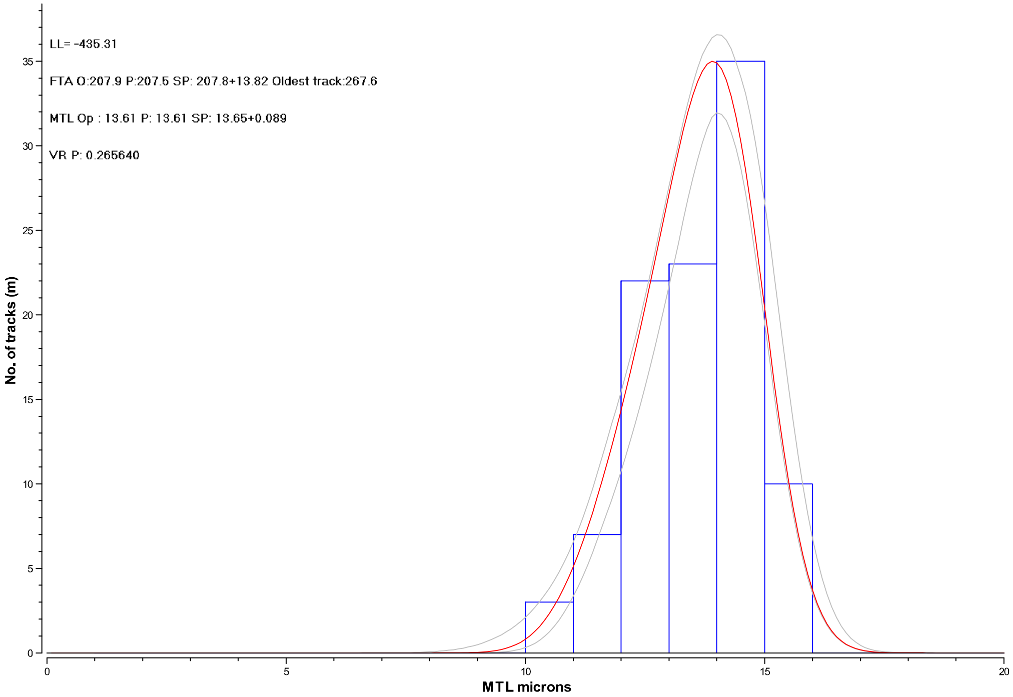

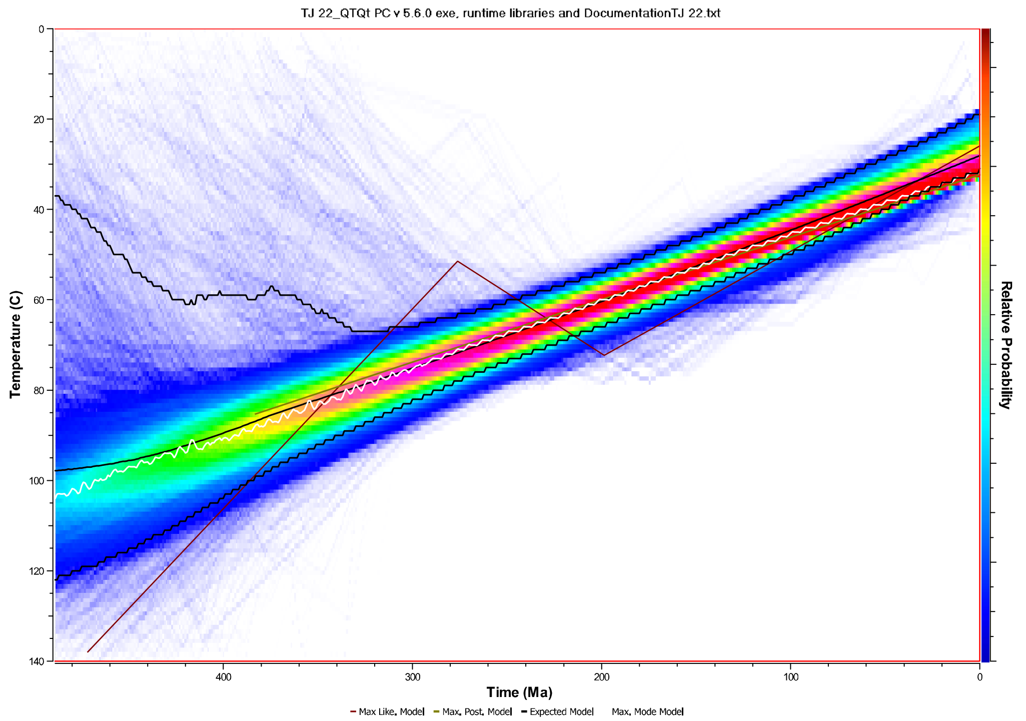
TJ 22_QTQt PC v 5.6.0 exe, runtime libraries and DocumentationTJ 22.txt
Temperature (C)
Time (Ma)
Relative Probability
Max Like. Model
Max. Post. Model
Expected Model
Max. Mode Model

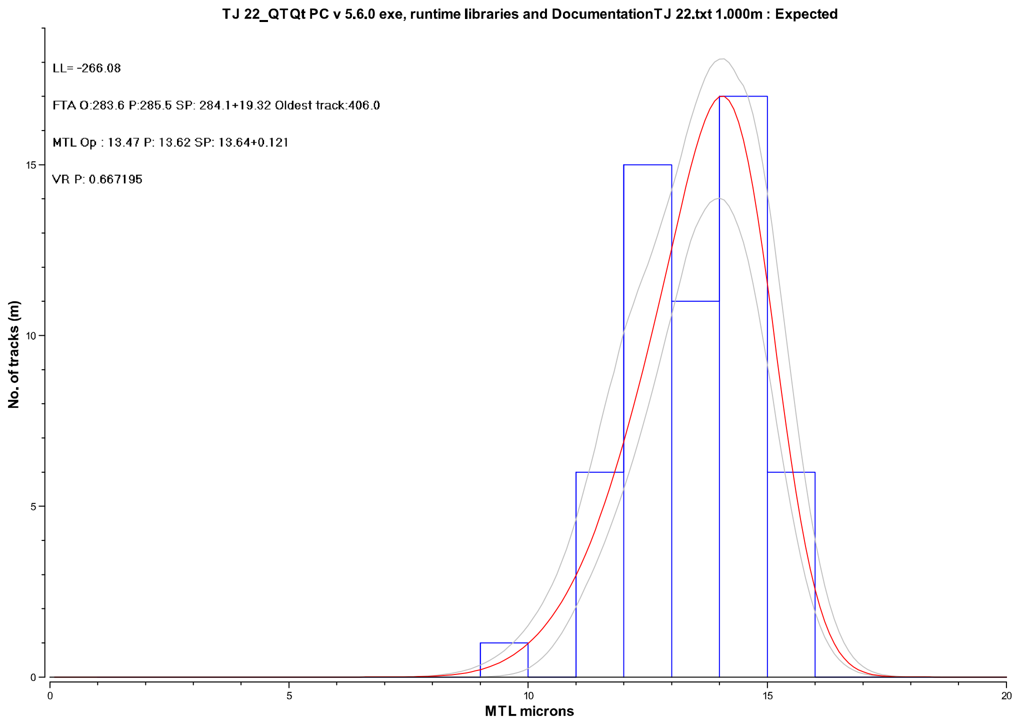
TJ 22_QTQt PC v 5.6.0 exe, runtime libraries and DocumentationTJ 22.txt 1.000m : Expected
LL= -266.08
FTA O:283.6 P:285.5 SP: 284.1+19.32 Oldest track:406.0
MTL Op : 13.47 P: 13.62 SP: 13.64+0.121
VR P: 0.667195
No. of tracks (m)
MTL microns

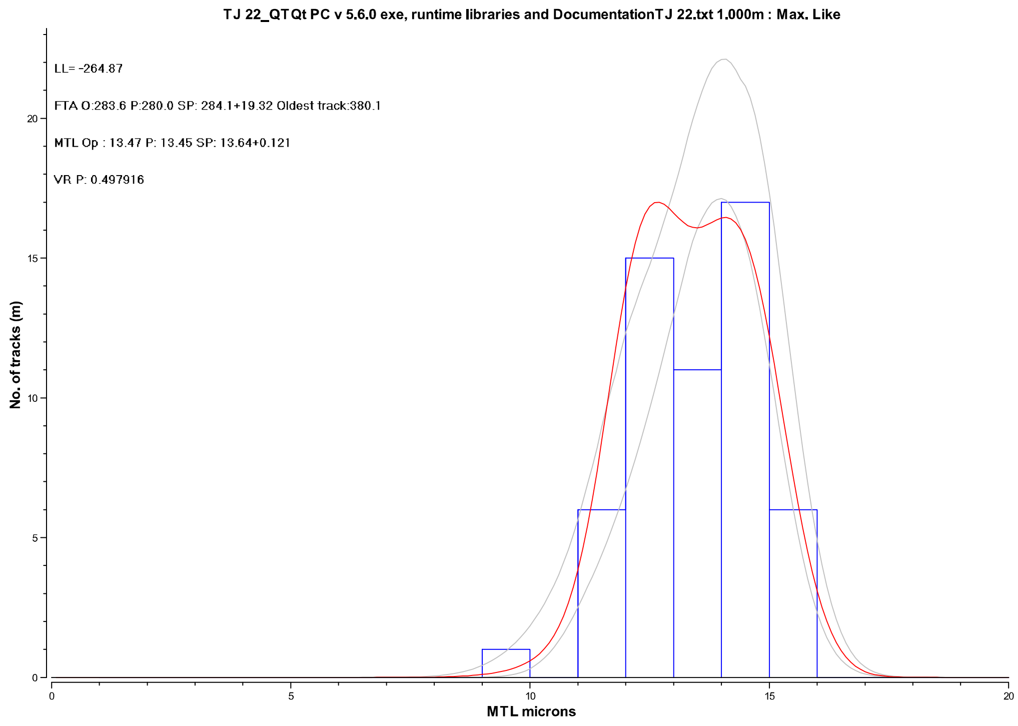
TJ 22_QTQt PC v 5.6.0 exe, runtime libraries and DocumentationTJ 22.txt 1.000m : Max. Like
LL= -264.87
FTA O:283.6 P:280.0 SP: 284.1+19.32 Oldest track:380.1
MTL Op : 13.47 P: 13.45 SP: 13.64+0.121
VR P: 0.497916
No. of tracks (m)
MTL microns

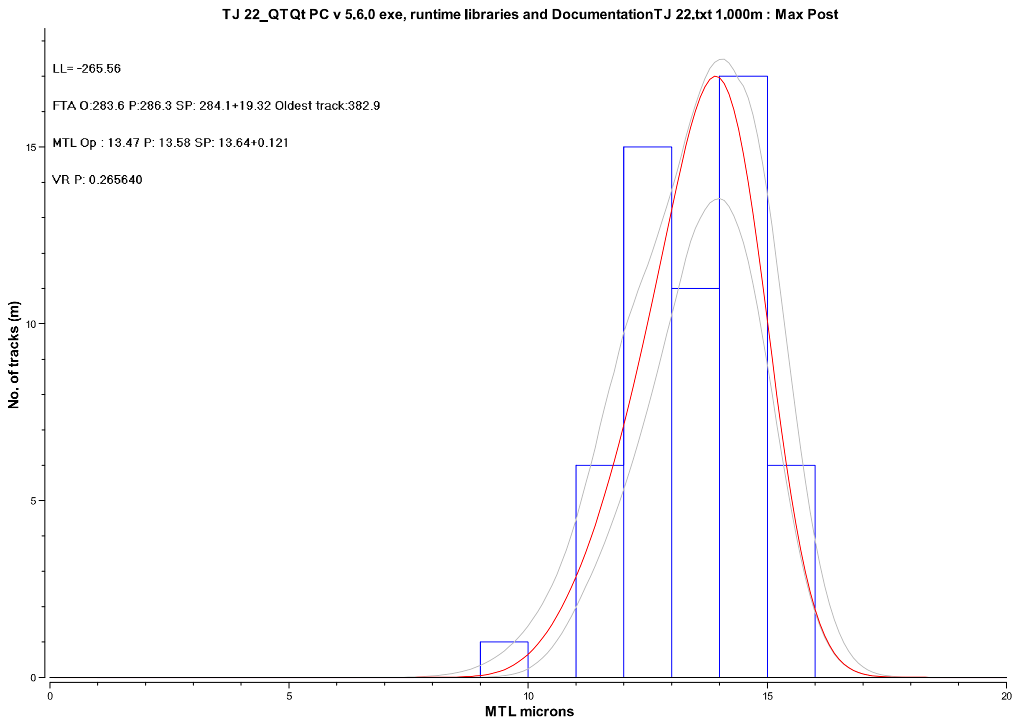
TJ 22_QTQt PC v 5.6.0 exe, runtime libraries and DocumentationTJ 22.txt 1.000m : Max Post
LL= -265.56
FTA O:283.6 P:286.3 SP: 284.1+19.32 Oldest track:382.9
MTL Op : 13.47 P: 13.58 SP: 13.64+0.121
VR P: 0.265640
No. of tracks (m)
MTL microns

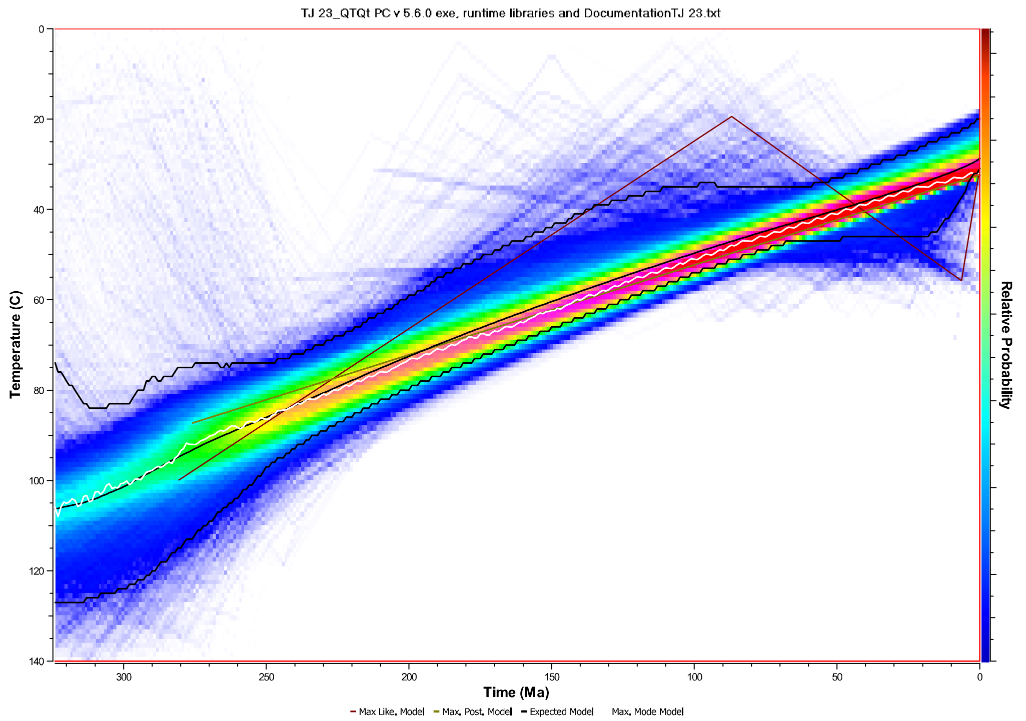
TJ 23_QTQt PC v 5.6.0 exe, runtime libraries and DocumentationTJ 23.txt
Temperature (C)
Time (Ma)
Relative Probability
Max Like. Model
Max. Post. Model
Expected Model
Max. Mode Model

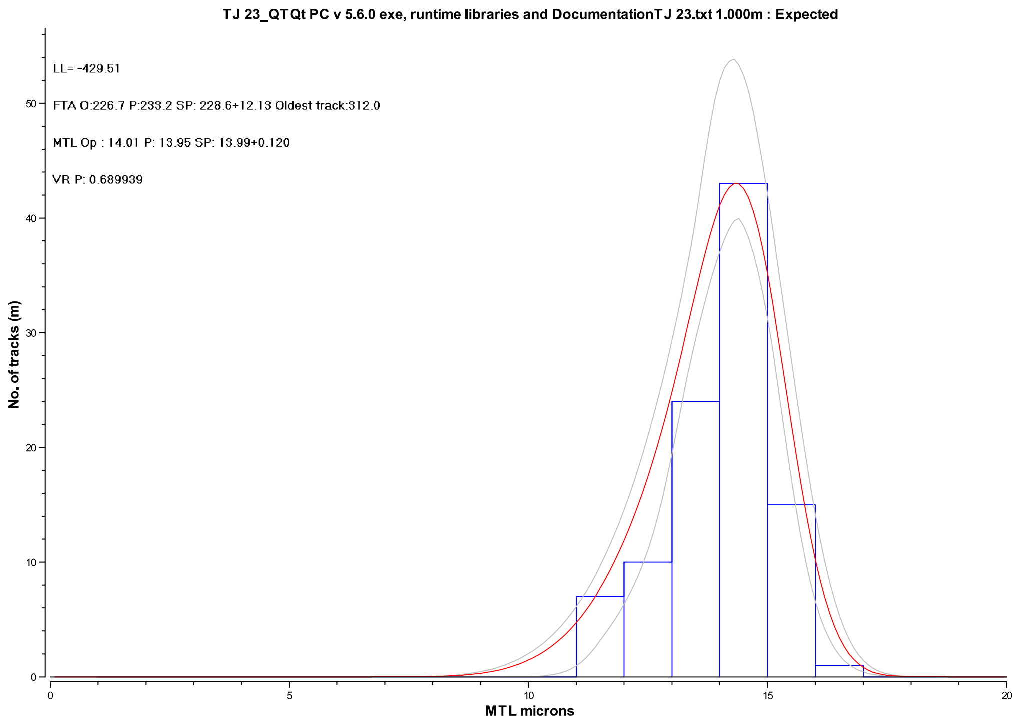
TJ 23_QTQt PC v 5.6.0 exe, runtime libraries and DocumentationTJ 23.txt 1.000m : Expected
LL= -429.51
FTA O:226.7 P:233.2 SP: 228.6+12.13 Oldest track:312.0
MTL Op : 14.01 P: 13.95 SP: 13.99+0.120
VR P: 0.689939
No. of tracks (m)
MTL microns

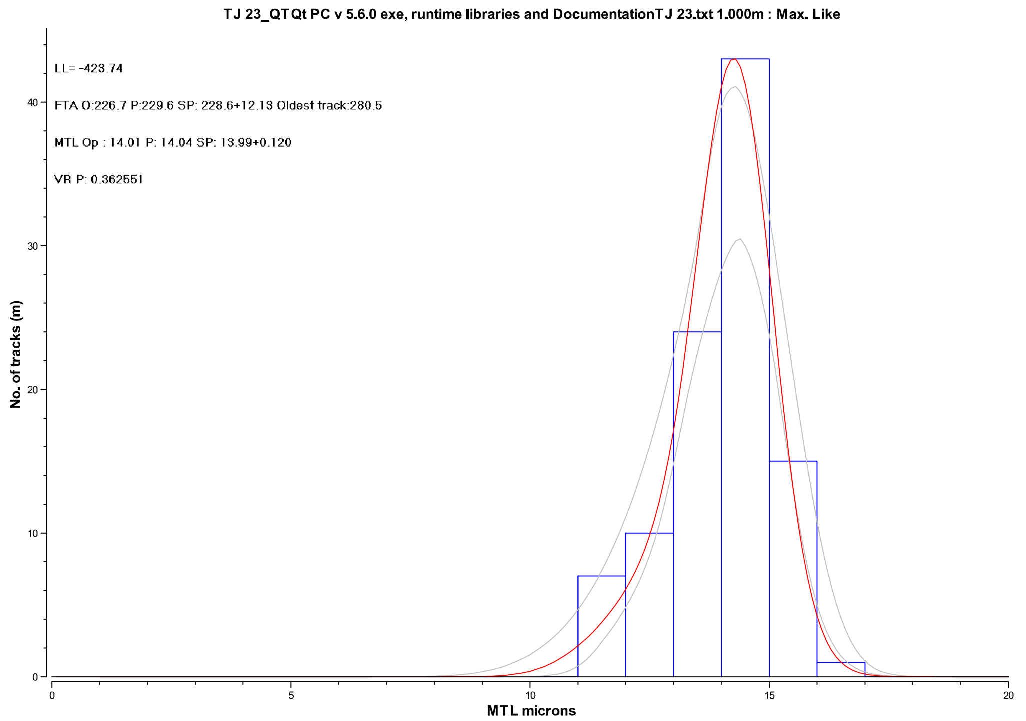
TJ 23_QTQt PC v 5.6.0 exe, runtime libraries and DocumentationTJ 23.txt 1.000m : Max. Like
LL= -423.74
FTA O:226.7 P:229.6 SP: 228.6+12.13 Oldest track:280.5
MTL Op : 14.01 P: 14.04 SP: 13.99+0.120
VR P: 0.362551
No. of tracks (m)
MTL microns

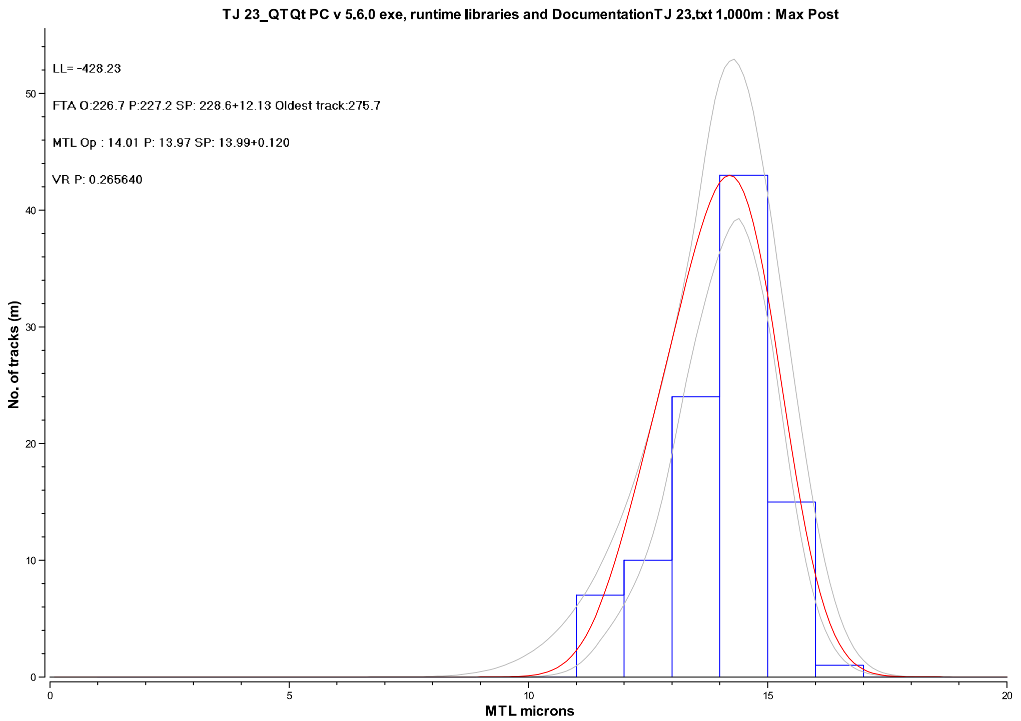
TJ 23_QTQt PC v 5.6.0 exe, runtime libraries and DocumentationTJ 23.txt 1.000m : Max Post
LL= -428.23
FTA O:226.7 P:227.2 SP: 228.6+12.13 Oldest track:275.7
MTL Op : 14.01 P: 13.97 SP: 13.99+0.120
VR P: 0.265640
No. of tracks (m)
MTL microns

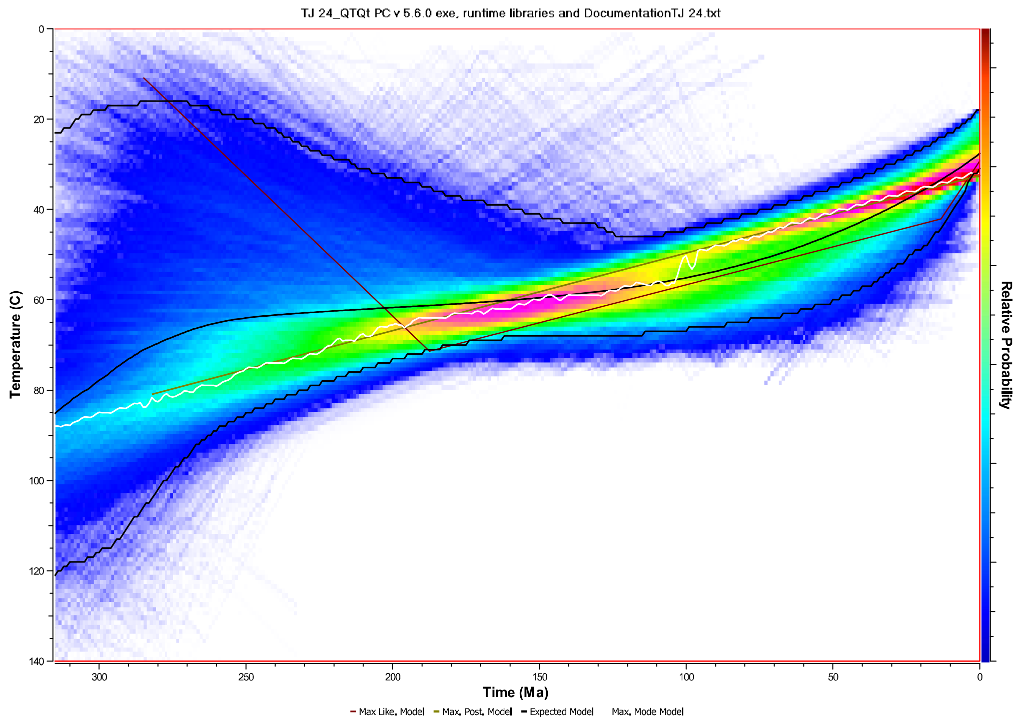
TJ 24_QTQt PC v 5.6.0 exe, runtime libraries and DocumentationTJ 24.txt
Temperature (C)
Time (Ma)
Relative Probability
Max Like. Model
Max. Post. Model
Expected Model
Max. Mode Model

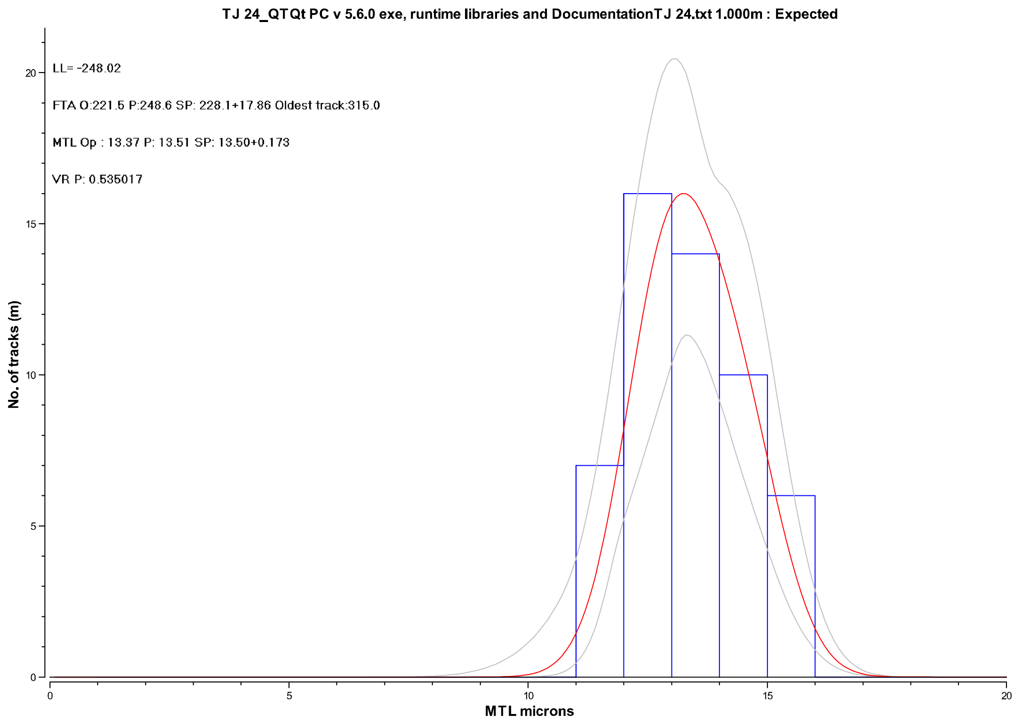
TJ 24_QTQt PC v 5.6.0 exe, runtime libraries and DocumentationTJ 24.txt 1.000m : Expected
LL= -248.02
FTA O:221.5 P:248.6 SP: 228.1+17.86 Oldest track:315.0
MTL Op : 13.37 P: 13.51 SP: 13.50+0.173
VR P: 0.535017
No. of tracks (m)
MTL microns

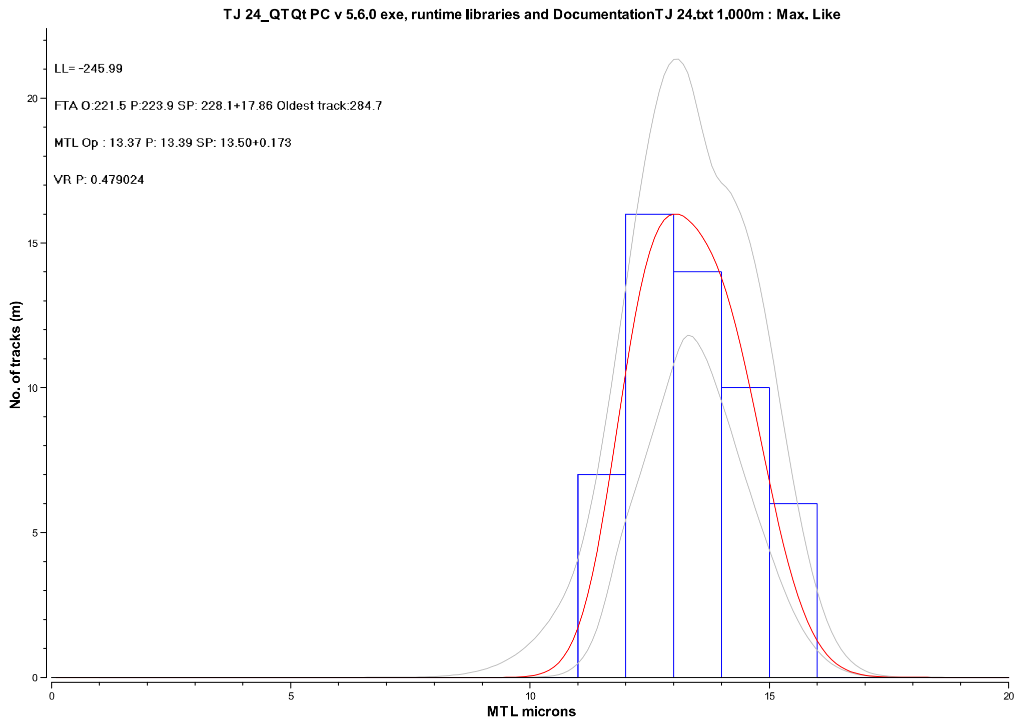
TJ 24_QTQt PC v 5.6.0 exe, runtime libraries and DocumentationTJ 24.txt 1.000m : Max. Like
LL= -245.99
FTA O:221.5 P:223.9 SP: 228.1+17.86 Oldest track:284.7
MTL Op : 13.37 P: 13.39 SP: 13.50+0.173
VR P: 0.479024
No. of tracks (m)
MTL microns

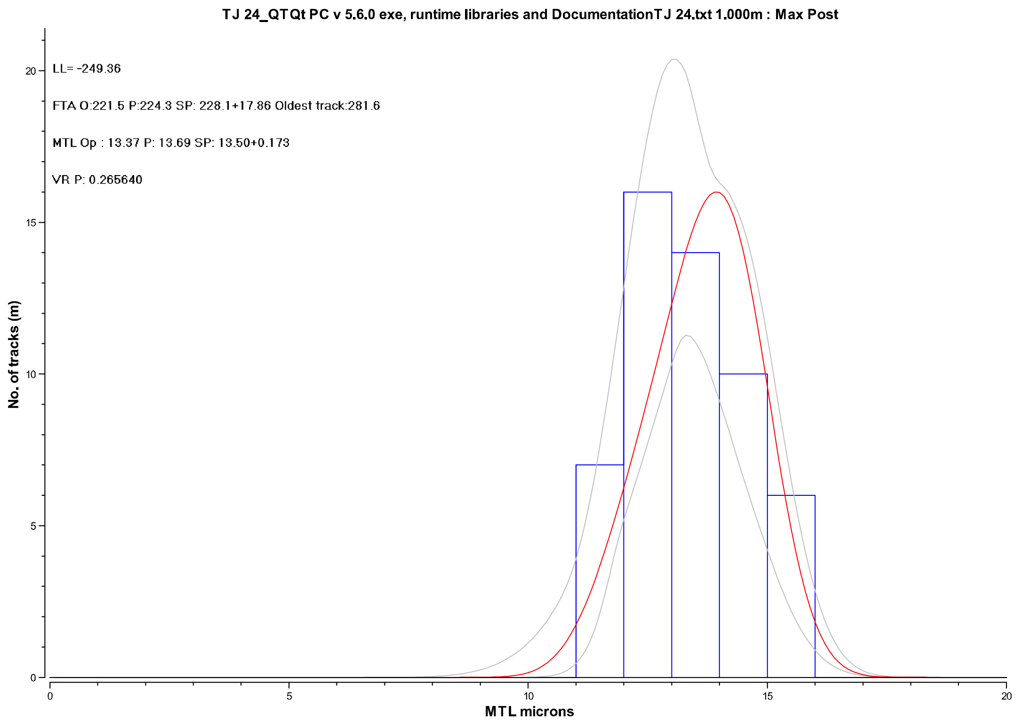
TJ 24_QTQt PC v 5.6.0 exe, runtime libraries and DocumentationTJ 24.txt 1.000m : Max Post
LL= -249.36
FTA O:221.5 P:224.3 SP: 228.1+17.86 Oldest track:281.6
MTL Op : 13.37 P: 13.69 SP: 13.50+0.173
VR P: 0.265640
No. of tracks (m)
MTL microns

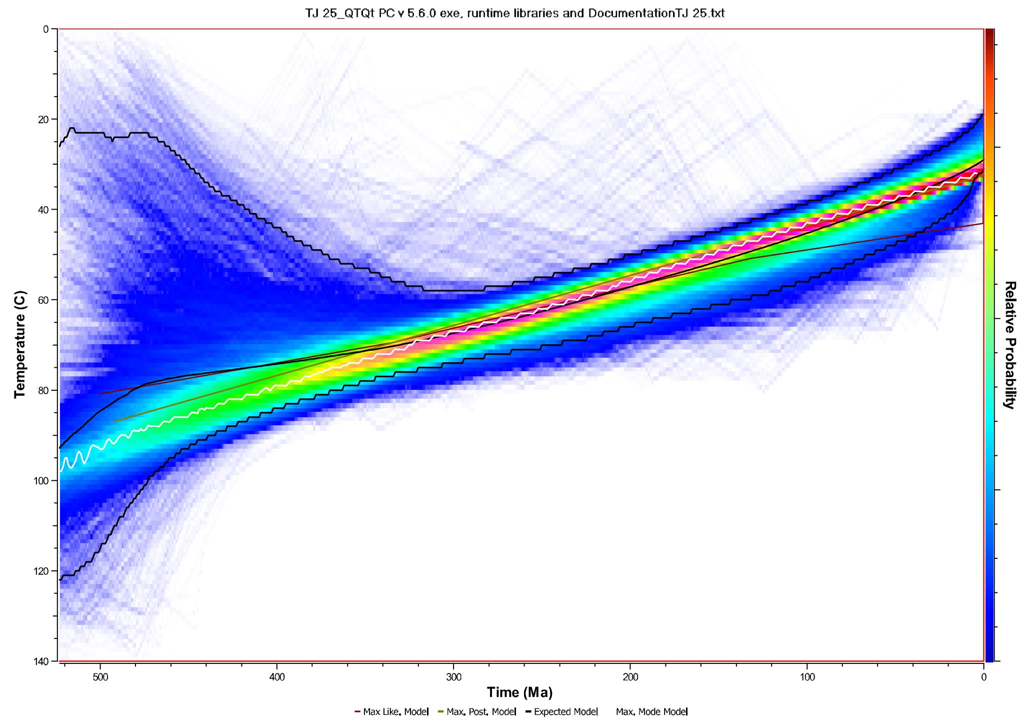
TJ 25_QTQt PC v 5.6.0 exe, runtime libraries and DocumentationTJ 25.txt
Temperature (C)
Time (Ma)
Relative Probability
Max Like. Model
Max. Post. Model
Expected Model
Max. Mode Model

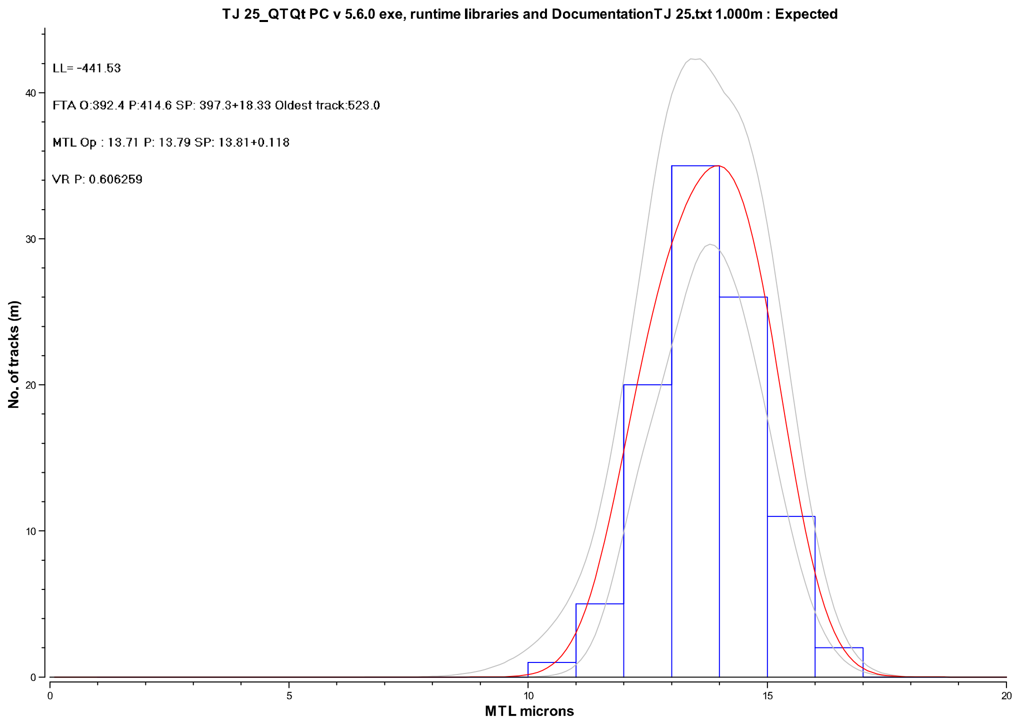
TJ 25_QTQt PC v 5.6.0 exe, runtime libraries and DocumentationTJ 25.txt 1.000m : Expected
LL= -441.53
FTA O:392.4 P:414.6 SP: 397.3+18.33 Oldest track:523.0
MTL Op : 13.71 P: 13.79 SP: 13.81+0.118
VR P: 0.606259
No. of tracks (m)
MTL microns

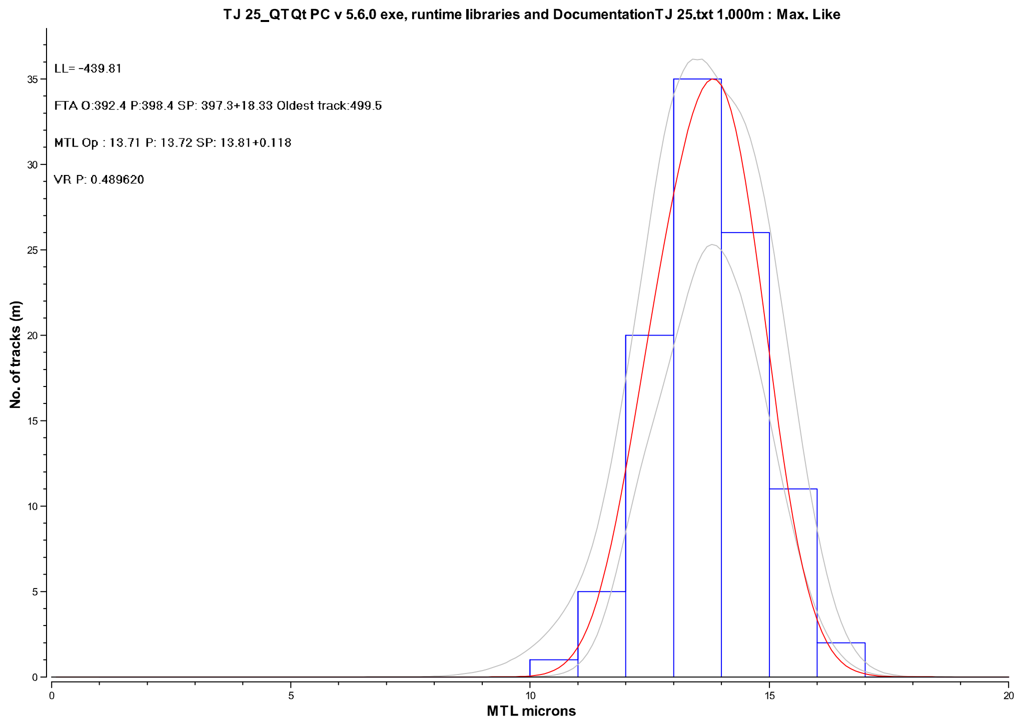
TJ 25_QTQt PC v 5.6.0 exe, runtime libraries and DocumentationTJ 25.txt 1.000m : Max. Like
LL= -439.81
FTA O:392.4 P:398.4 SP: 397.3+18.33 Oldest track:499.5
MTL Op : 13.71 P: 13.72 SP: 13.81+0.118
VR P: 0.489620
No. of tracks (m)
MTL microns

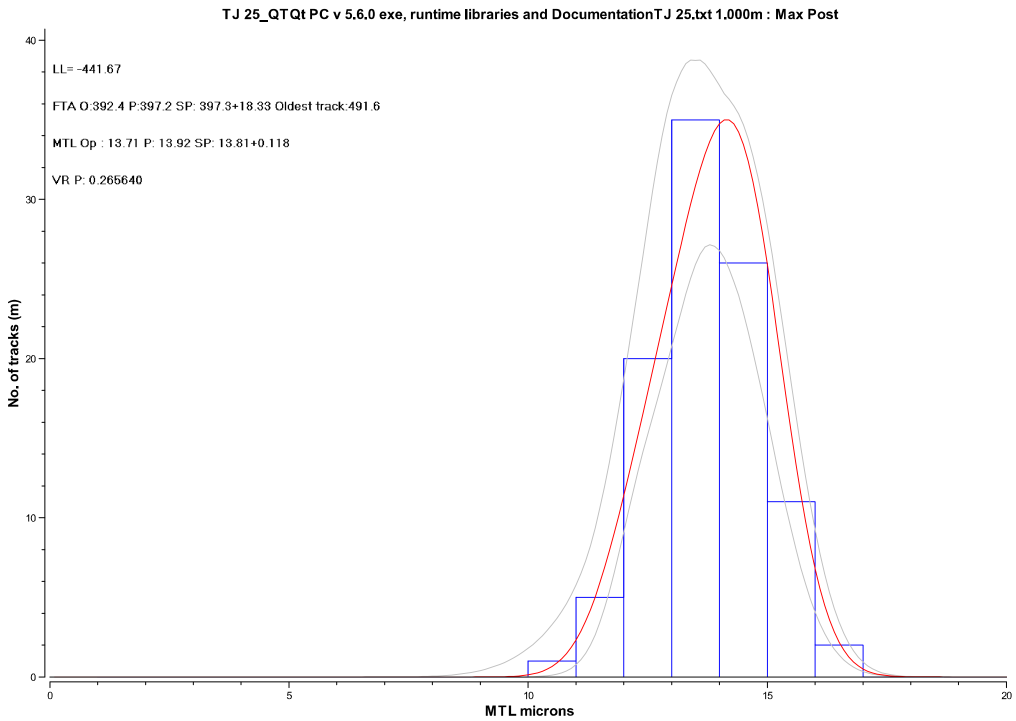
TJ 25_QTQt PC v 5.6.0 exe, runtime libraries and DocumentationTJ 25.txt 1.000m : Max Post
LL= -441.67
FTA O:392.4 P:397.2 SP: 397.3+18.33 Oldest track:491.6
MTL Op : 13.71 P: 13.92 SP: 13.81+0.118
VR P: 0.265640
No. of tracks (m)
MTL microns

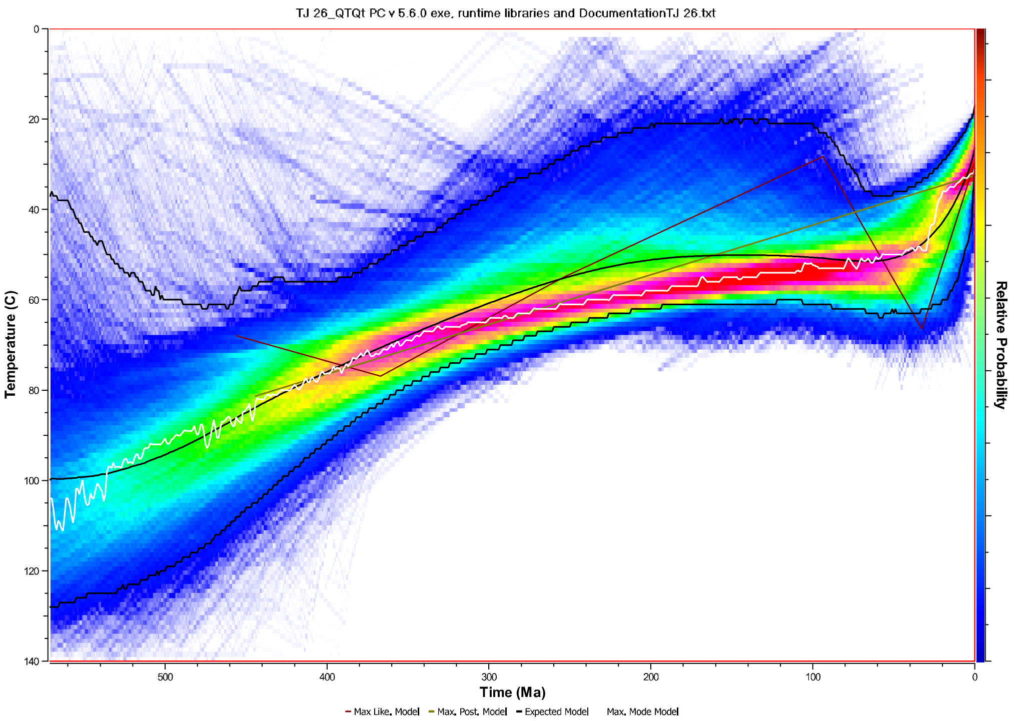
TJ 26_QTQt PC v 5.6.0 exe, runtime libraries and DocumentationTJ 26.txt
Temperature (C)
Time (Ma)
Relative Probability
Max Like. Model
Max. Post. Model
Expected Model
Max. Mode Model

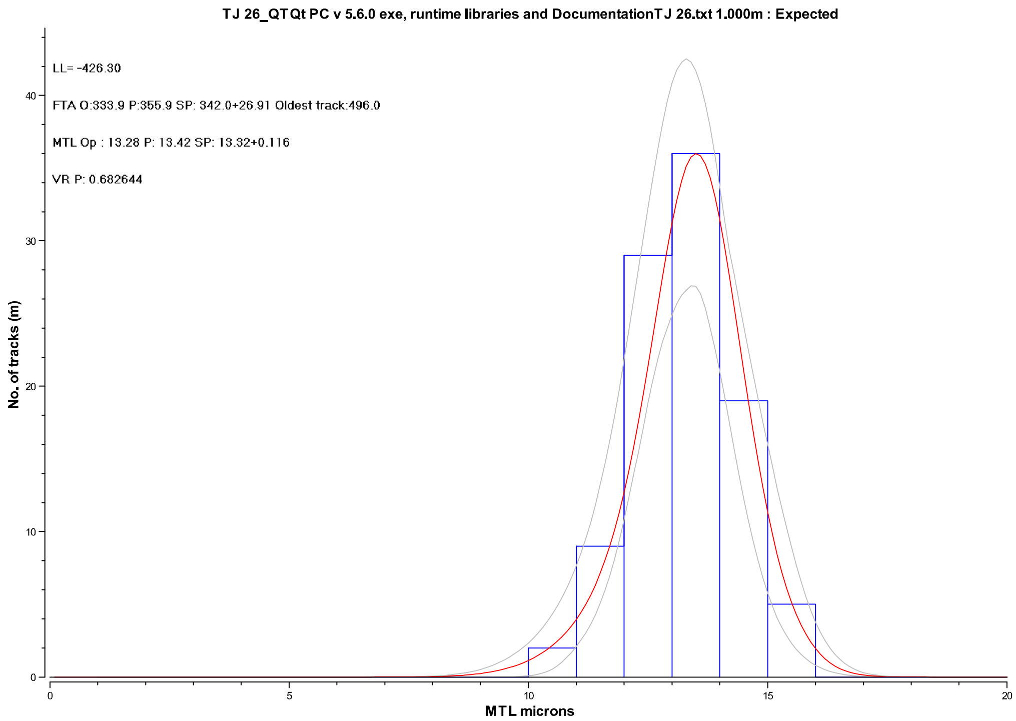
TJ 26_QTQt PC v 5.6.0 exe, runtime libraries and DocumentationTJ 26.txt 1.000m : Expected
LL= -426.30
FTA O:333.9 P:355.9 SP: 342.0+26.91 Oldest track:496.0
MTL Op : 13.28 P: 13.42 SP: 13.32+0.116
VR P: 0.682644
No. of tracks (m)
MTL microns

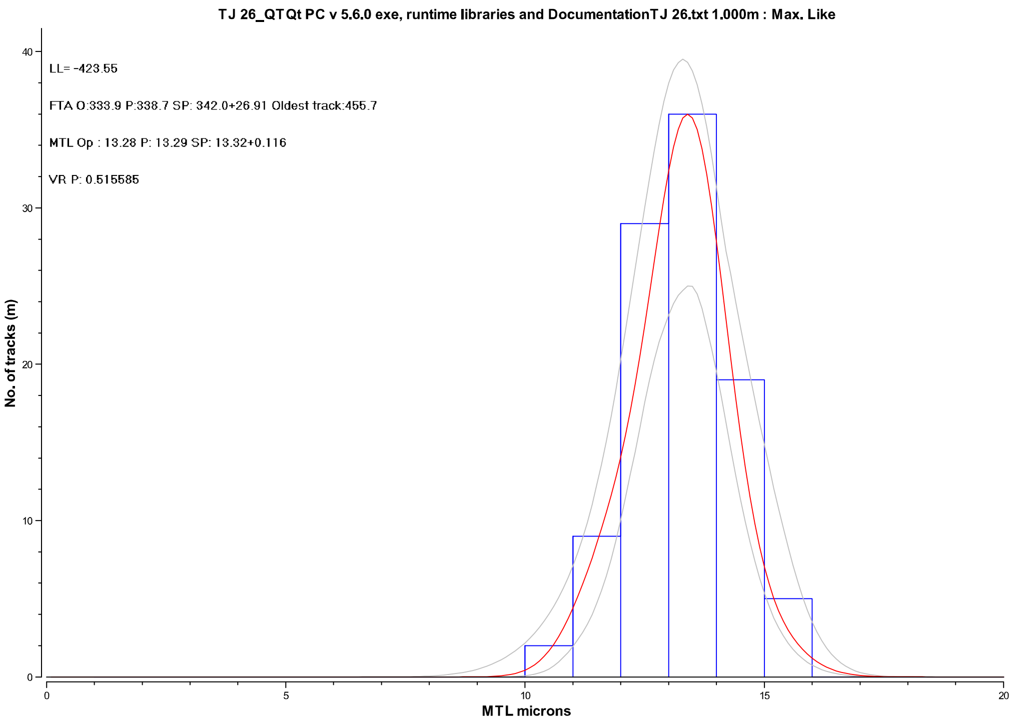
TJ 26_QTQt PC v 5.6.0 exe, runtime libraries and DocumentationTJ 26.txt 1.000m : Max. Like
LL= -423.55
FTA O:333.9 P:338.7 SP: 342.0+26.91 Oldest track:455.7
MTL Op : 13.28 P: 13.29 SP: 13.32+0.116
VR P: 0.515585
No. of tracks (m)
MTL microns

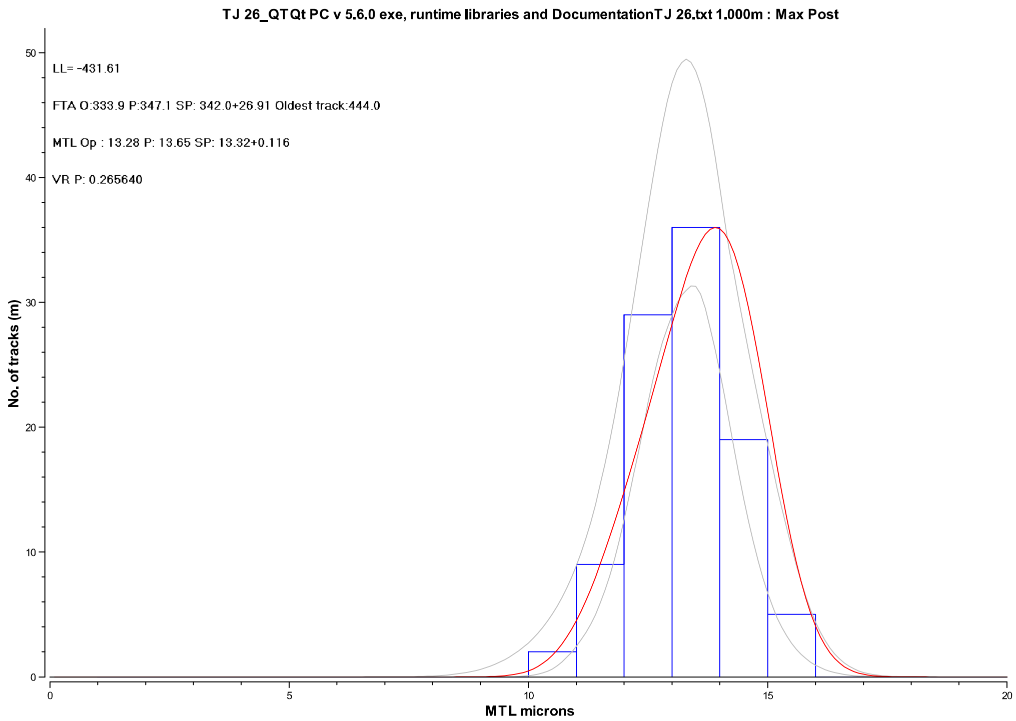
TJ 26_QTQt PC v 5.6.0 exe, runtime libraries and DocumentationTJ 26.txt 1.000m : Max Post
LL= -431.61
FTA O:333.9 P:347.1 SP: 342.0+26.91 Oldest track:444.0
MTL Op : 13.28 P: 13.65 SP: 13.32+0.116
VR P: 0.265640
No. of tracks (m)
MTL microns

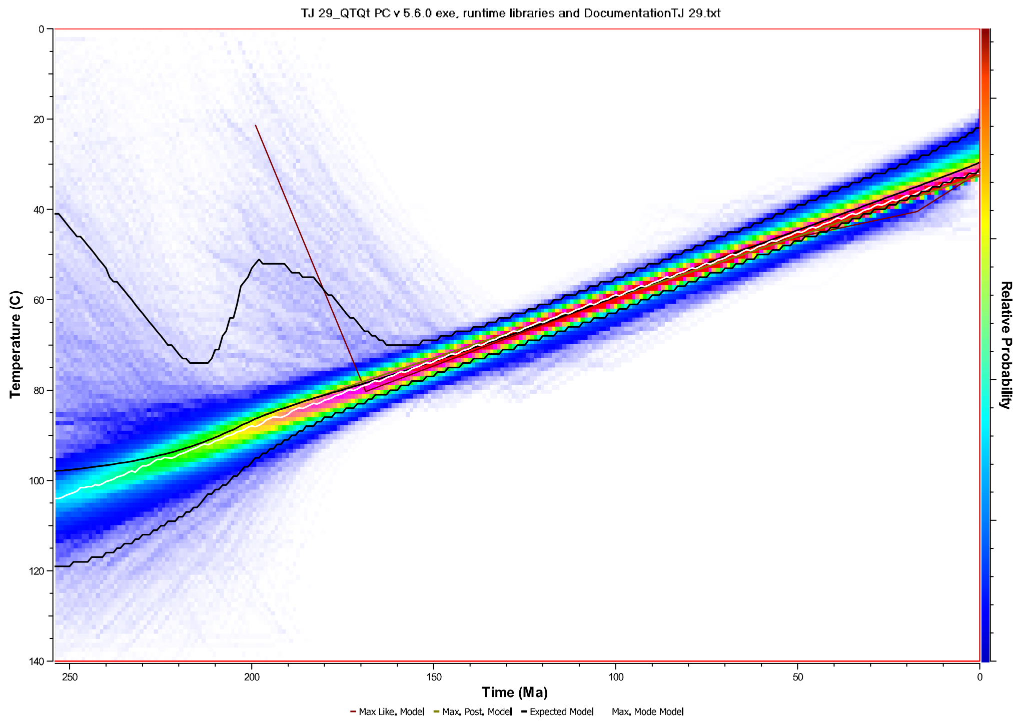
TJ 29_QTQt PC v 5.6.0 exe, runtime libraries and DocumentationTJ 29.txt
Temperature (C)
Time (Ma)
Relative Probability
Max Like. Model
Max. Post. Model
Expected Model
Max. Mode Model

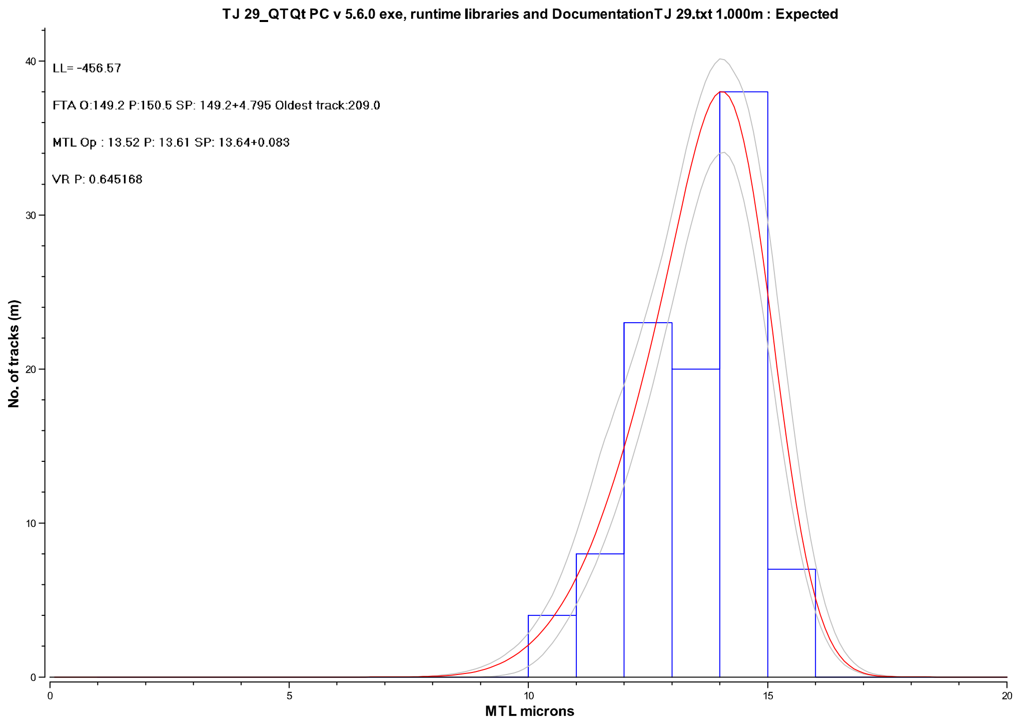
TJ 29_QTQt PC v 5.6.0 exe, runtime libraries and DocumentationTJ 29.txt 1.000m : Expected
LL= -456.57
FTA O:149.2 P:150.5 SP: 149.2+4.795 Oldest track:209.0
MTL Op : 13.52 P: 13.61 SP: 13.64+0.083
VR P: 0.645168
No. of tracks (m)
MTL microns

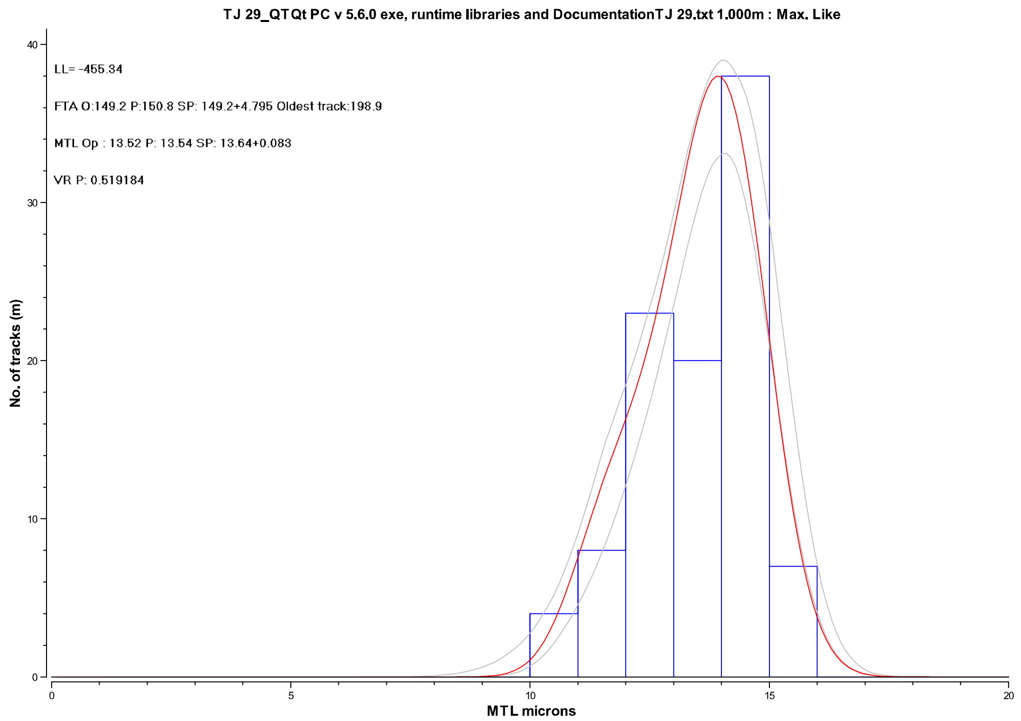
TJ 29_QTQt PC v 5.6.0 exe, runtime libraries and DocumentationTJ 29.txt 1.000m : Max. Like
LL= -455.34
FTA O:149.2 P:150.8 SP: 149.2+4.795 Oldest track:198.9
MTL Op : 13.52 P: 13.54 SP: 13.64+0.083
VR P: 0.519184
No. of tracks (m)
MTL microns

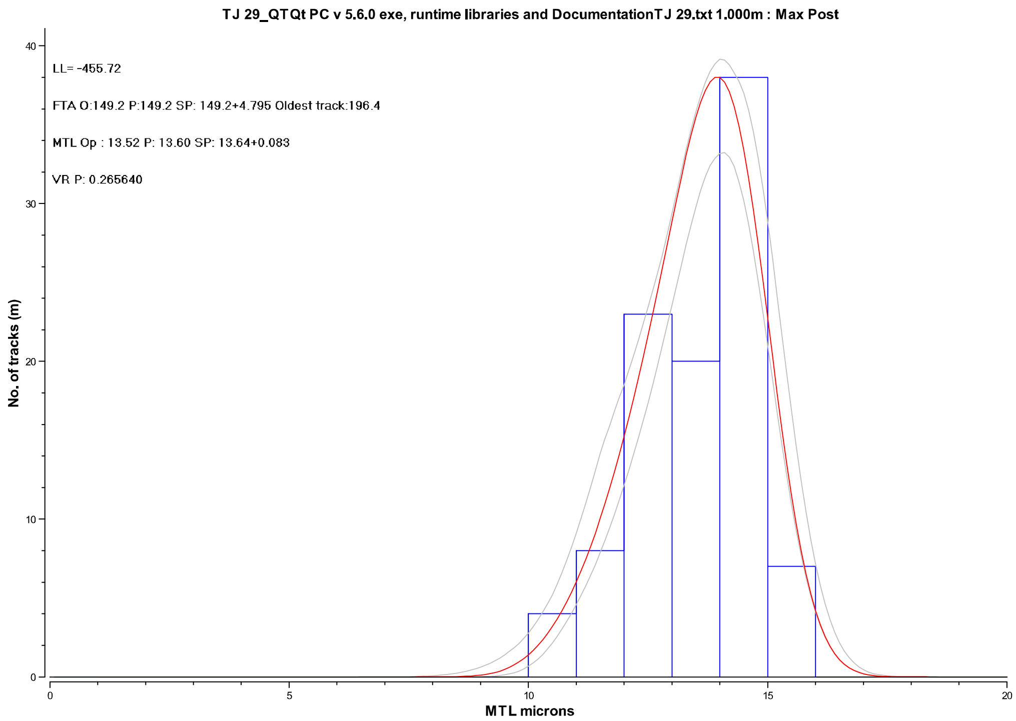
TJ 29_QTQt PC v 5.6.0 exe, runtime libraries and DocumentationTJ 29.txt 1.000m : Max Post
LL= -455.72
FTA O:149.2 P:149.2 SP: 149.2+4.795 Oldest track:196.4
MTL Op : 13.52 P: 13.60 SP: 13.64+0.083
VR P: 0.265640
No. of tracks (m)
MTL microns

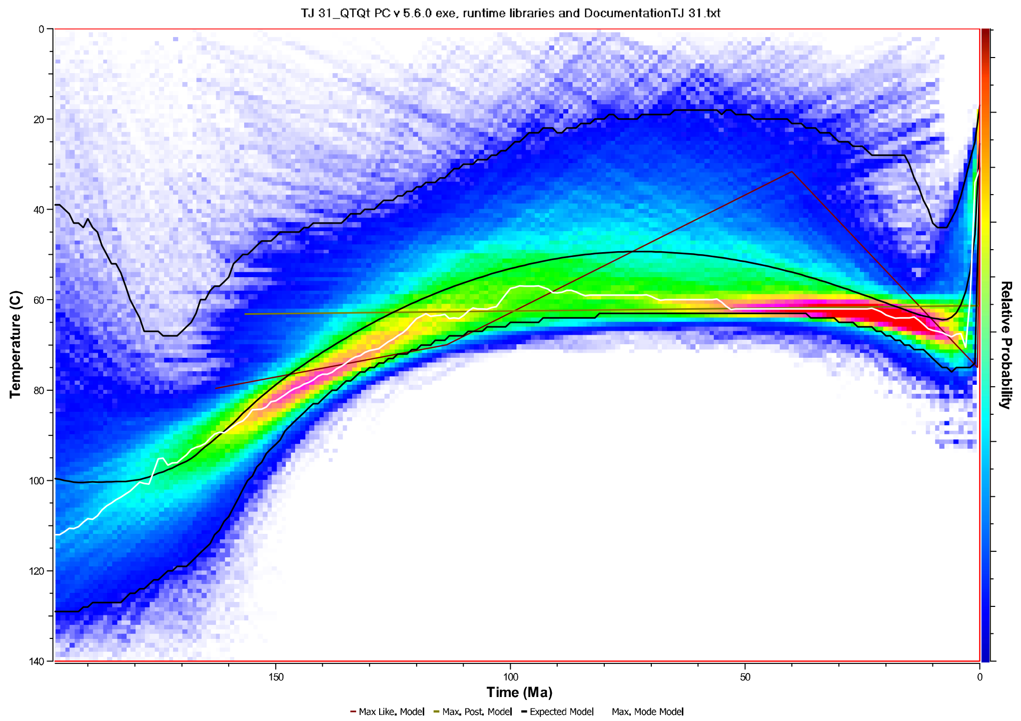
TJ 31_QTQt PC v 5.6.0 exe, runtime libraries and DocumentationTJ 31.txt
Temperature (C)
Time (Ma)
Relative Probability
Max Like. Model
Max. Post. Model
Expected Model
Max. Mode Model

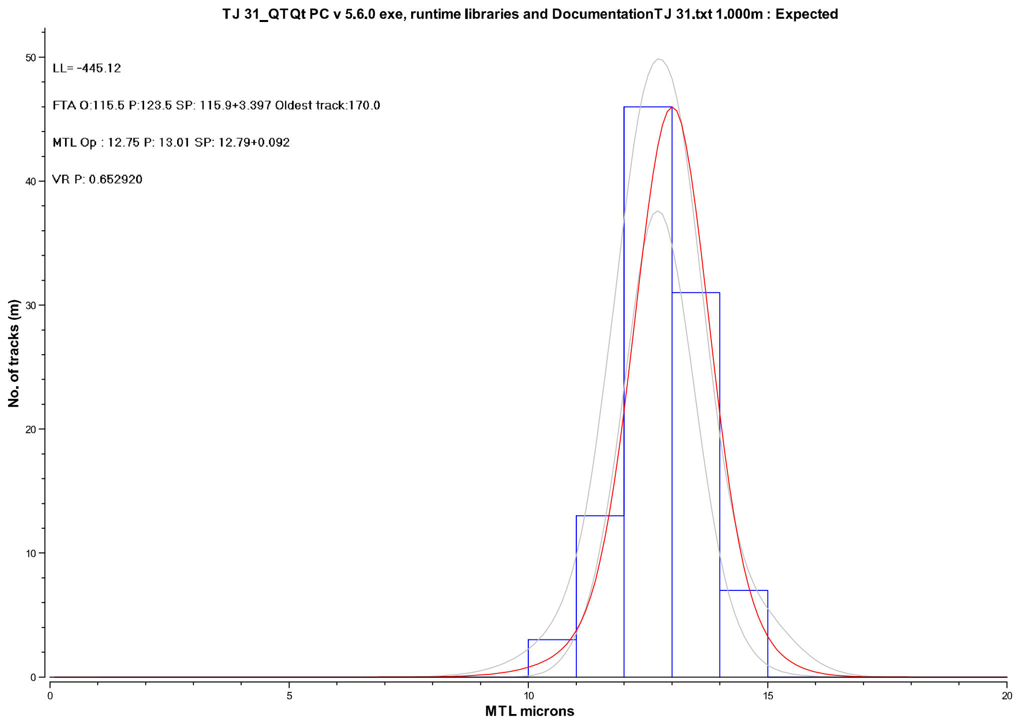
TJ 31_QTQt PC v 5.6.0 exe, runtime libraries and DocumentationTJ 31.txt 1.000m : Expected
LL= -445.12
FTA O:115.5 P:123.5 SP: 115.9+3.397 Oldest track:170.0
MTL Op : 12.75 P: 13.01 SP: 12.79+0.092
VR P: 0.652920
No. of tracks (m)
MTL microns

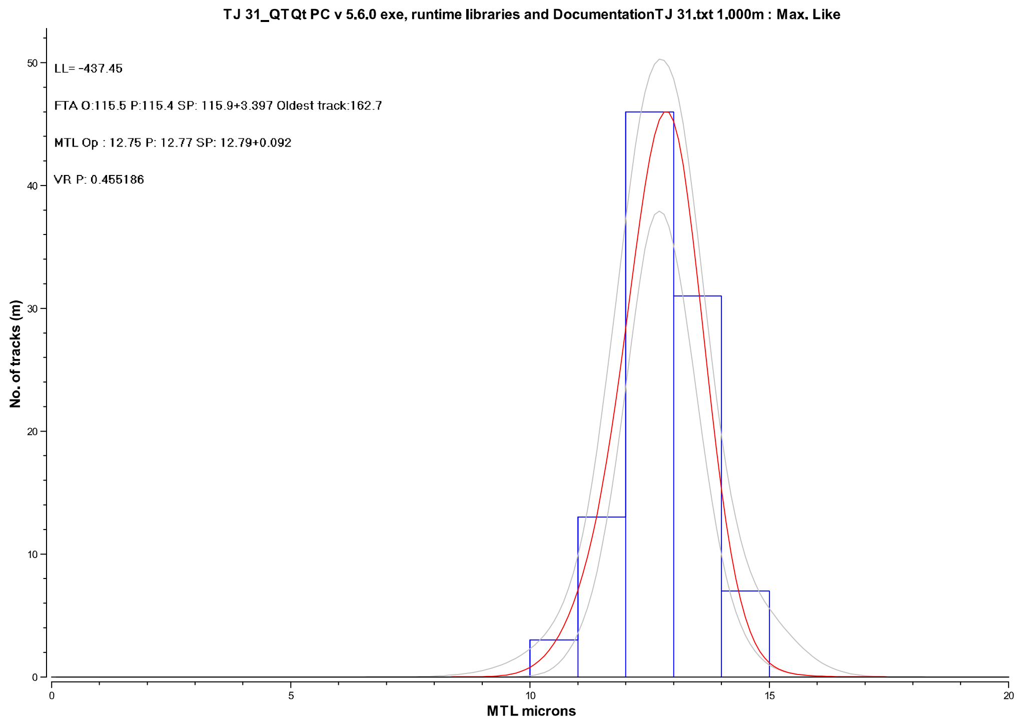
TJ 31_QTQt PC v 5.6.0 exe, runtime libraries and DocumentationTJ 31.txt 1.000m : Max. Like
LL= -437.45
FTA O:115.5 P:115.4 SP: 115.9+3.397 Oldest track:162.7
MTL Op : 12.75 P: 12.77 SP: 12.79+0.092
VR P: 0.455186
No. of tracks (m)
MTL microns

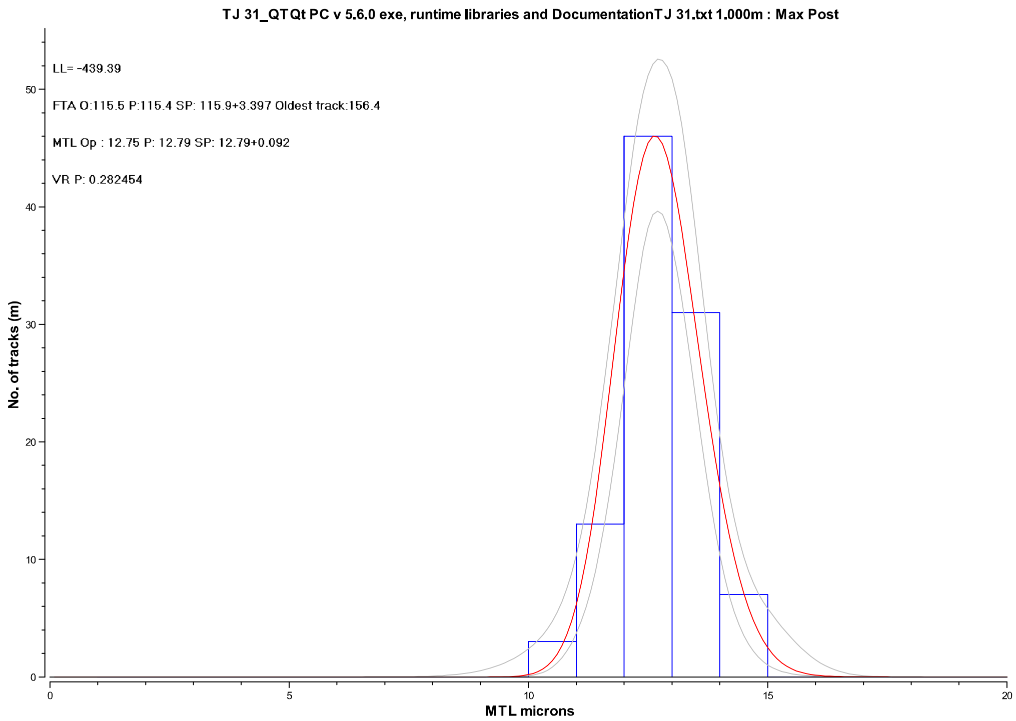
TJ 31_QTQt PC v 5.6.0 exe, runtime libraries and DocumentationTJ 31.txt 1.000m : Max Post
LL= -439.39
FTA O:115.5 P:115.4 SP: 115.9+3.397 Oldest track:156.4
MTL Op : 12.75 P: 12.79 SP: 12.79+0.092
VR P: 0.282454
No. of tracks (m)
MTL microns

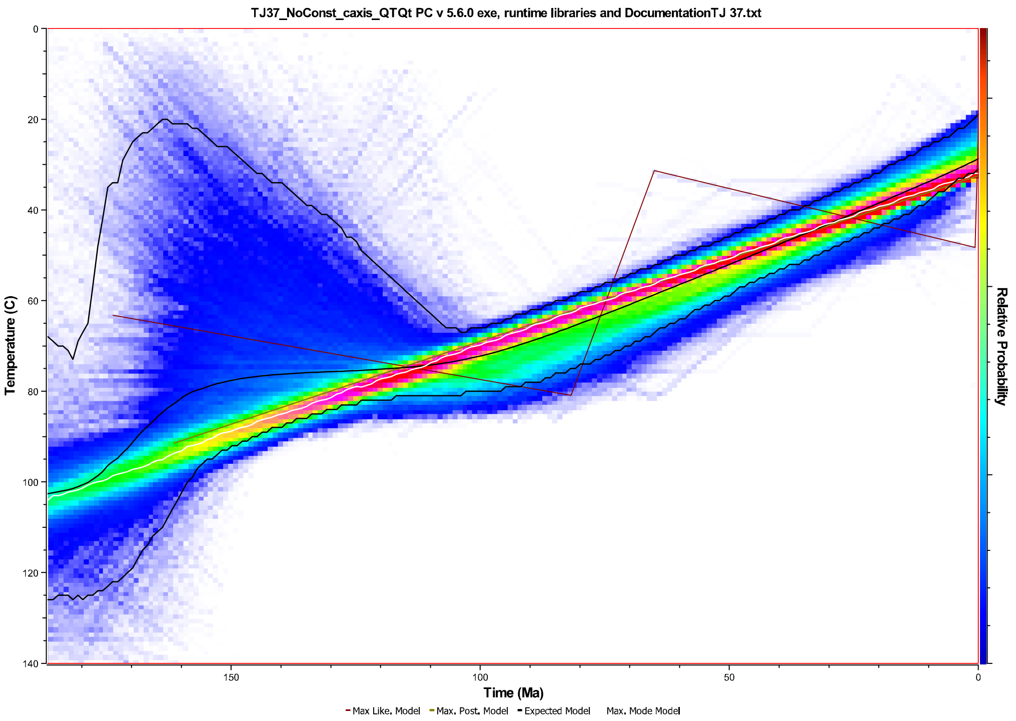
TJ37_NoConst_caxis_QTQt PC v 5.6.0 exe, runtime libraries and DocumentationTJ 37.txt
Temperature (C)
Time (Ma)
Relative Probability
Max Like. Model
Max. Post. Model
Expected Model
Max. Mode Model

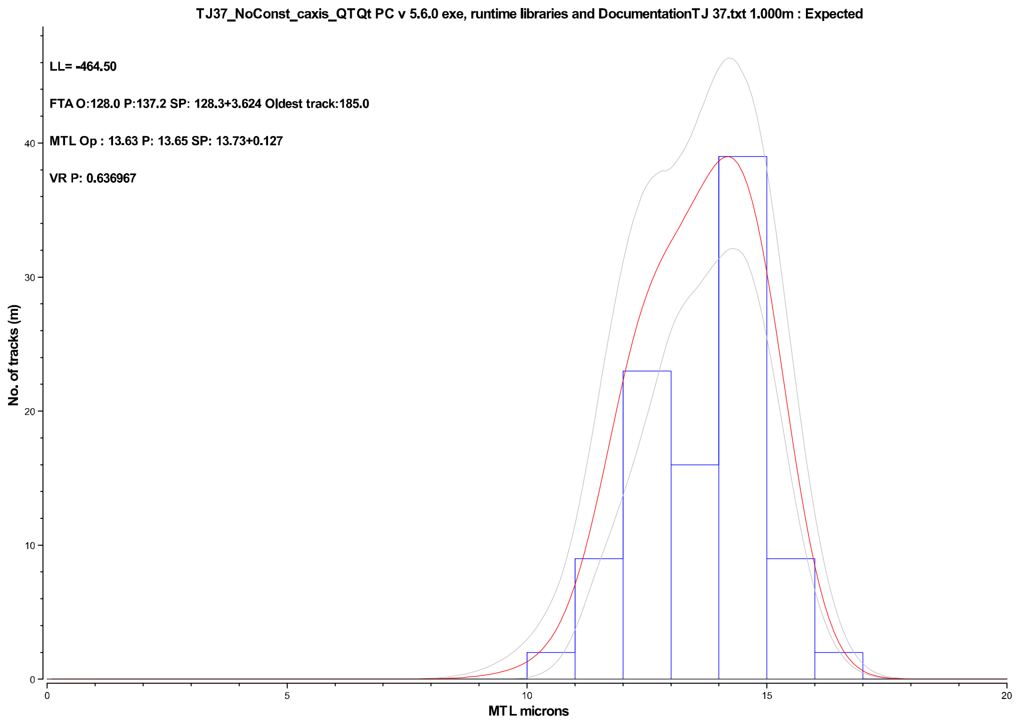
TJ37_NoConst_caxis_QTQt PC v 5.6.0 exe, runtime libraries and DocumentationTJ 37.txt 1.000m : Expected
LL= -464.50
FTA O:128.0 P:137.2 SP: 128.3+3.624 Oldest track:185.0
MTL Op : 13.63 P: 13.65 SP: 13.73+0.127
VR P: 0.636967
No. of tracks (m)
MTL microns

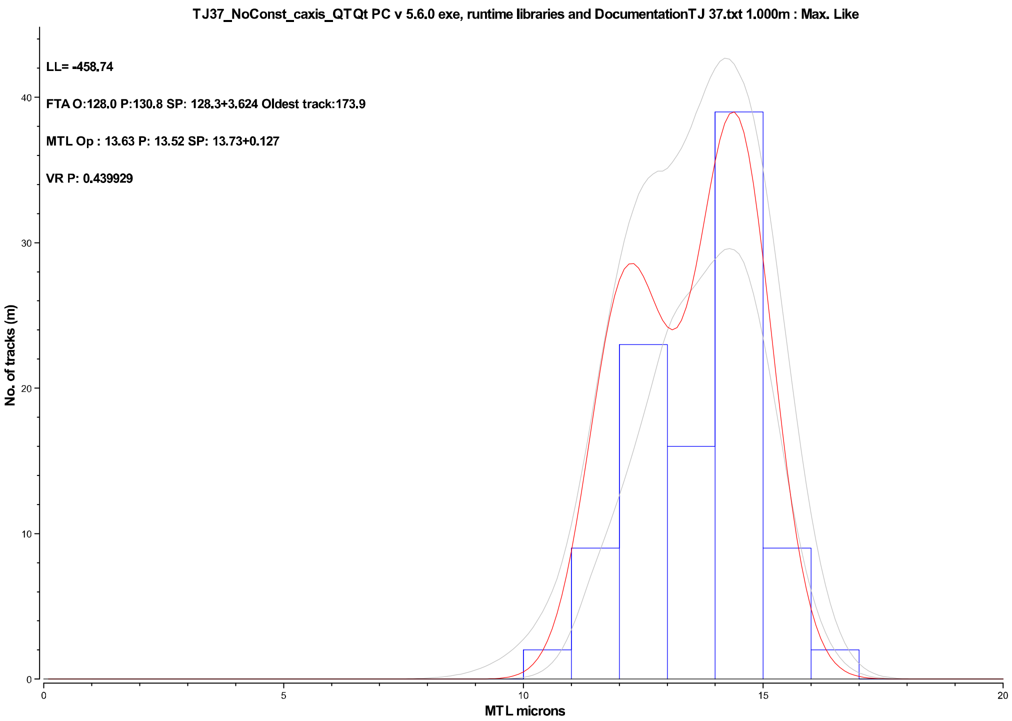
TJ37_NoConst_caxis_QTQt PC v 5.6.0 exe, runtime libraries and DocumentationTJ 37.txt 1.000m : Max. Like
LL= -458.74
FTA O:128.0 P:130.8 SP: 128.3+3.624 Oldest track:173.9
MTL Op : 13.63 P: 13.52 SP: 13.73+0.127
VR P: 0.439929
No. of tracks (m)
MTL microns

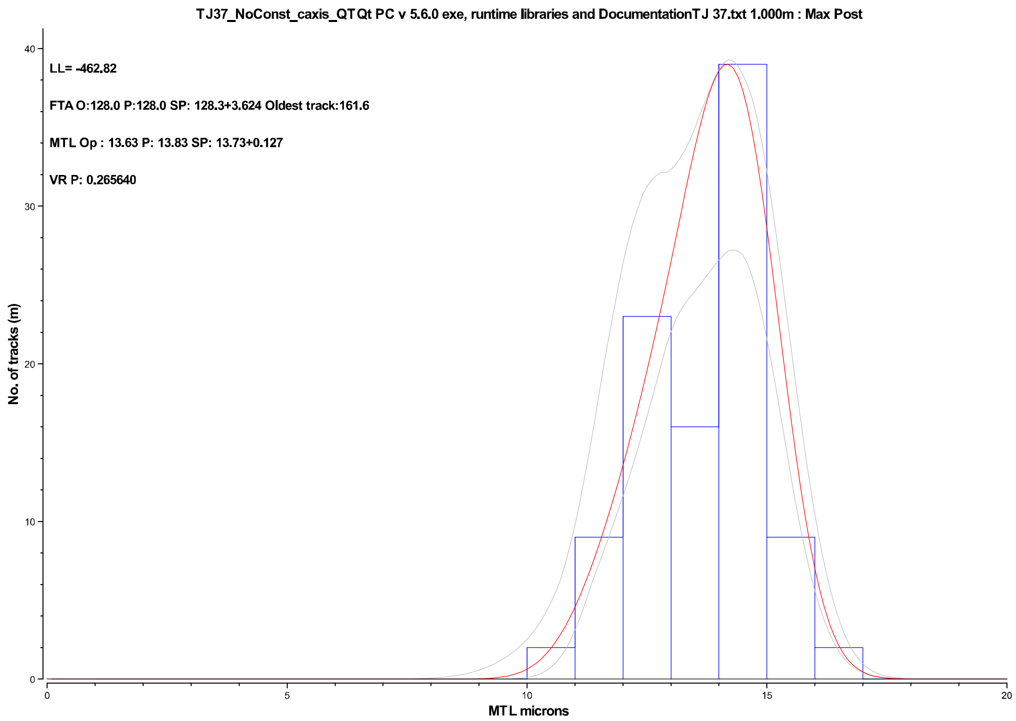
TJ37_NoConst_caxis_QTQt PC v 5.6.0 exe, runtime libraries and DocumentationTJ 37.txt 1.000m : Max Post
LL= -462.82
FTA O:128.0 P:128.0 SP: 128.3+3.624 Oldest track:161.6
MTL Op : 13.63 P: 13.83 SP: 13.73+0.127
VR P: 0.265640
No. of tracks (m)
MTL microns

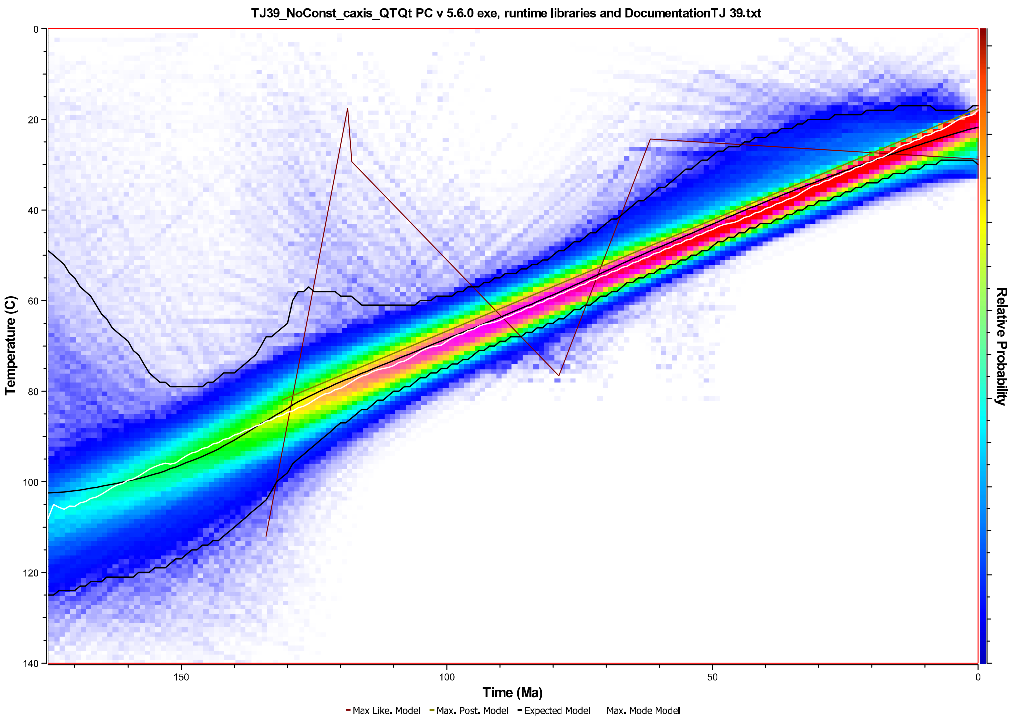
TJ39_NoConst_caxis_QTQt PC v 5.6.0 exe, runtime libraries and DocumentationTJ 39.txt
Temperature (C)
Time (Ma)
Relative Probability
Max Like. Model
Max. Post. Model
Expected Model
Max. Mode Model

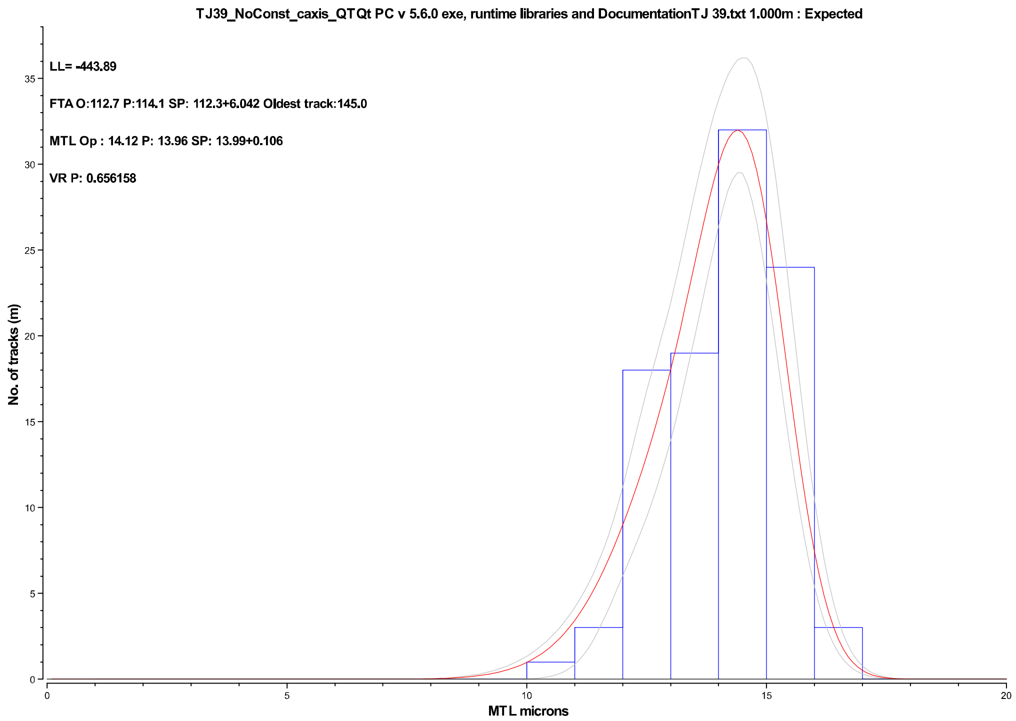
TJ39_NoConst_caxis_QTQt PC v 5.6.0 exe, runtime libraries and DocumentationTJ 39.txt 1.000m : Expected
LL= -443.89
FTA O:112.7 P:114.1 SP: 112.3+6.042 Oldest track:145.0
MTL Op : 14.12 P: 13.96 SP: 13.99+0.106
VR P: 0.656158
No. of tracks (m)
MTL microns

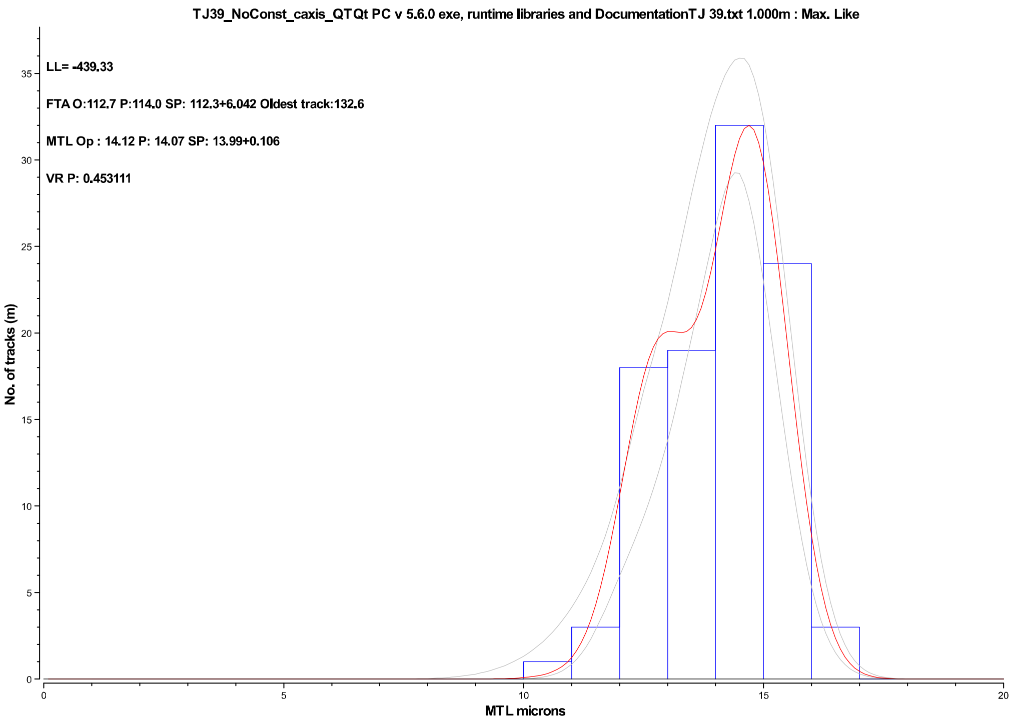
TJ39_NoConst_caxis_QTQt PC v 5.6.0 exe, runtime libraries and DocumentationTJ 39.txt 1.000m : Max. Like
LL= -439.33
FTA O:112.7 P:114.0 SP: 112.3+6.042 Oldest track:132.6
MTL Op : 14.12 P: 14.07 SP: 13.99+0.106
VR P: 0.453111
No. of tracks (m)
MTL microns

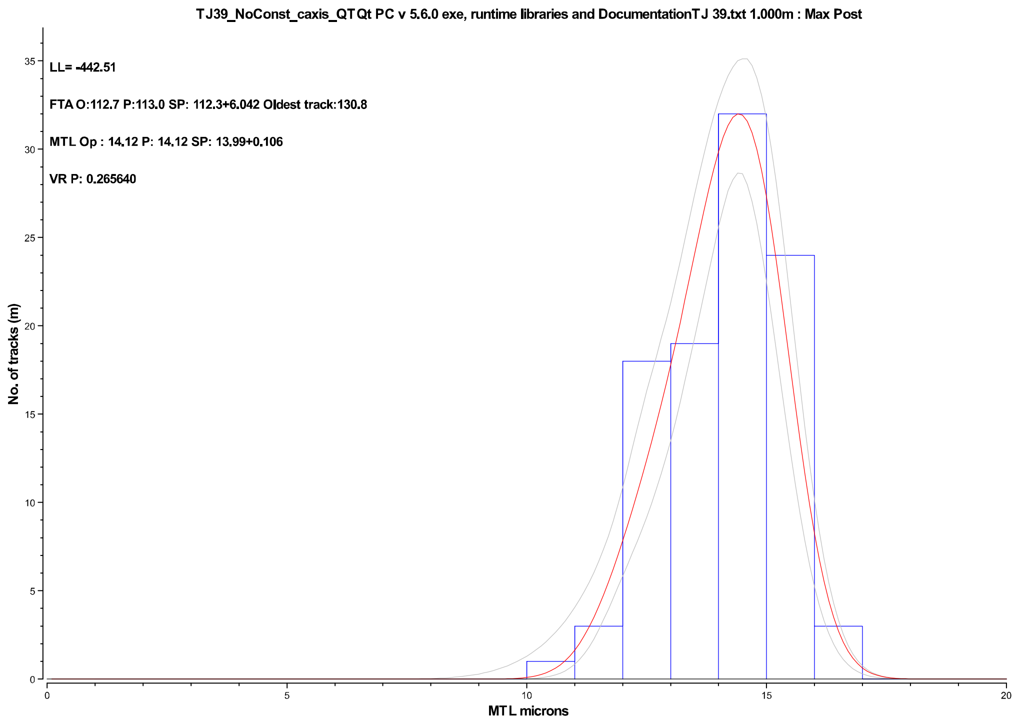
TJ39_NoConst_caxis_QTQt PC v 5.6.0 exe, runtime libraries and DocumentationTJ 39.txt 1.000m : Max Post
LL= -442.51
FTA O:112.7 P:113.0 SP: 112.3+6.042 Oldest track:130.8
MTL Op : 14.12 P: 14.12 SP: 13.99+0.106
VR P: 0.265640
No. of tracks (m)
MTL microns

TJ42_NoConst_caxis_QTQt PC v 5.6.0 exe, runtime libraries and DocumentationTJ 42.txt

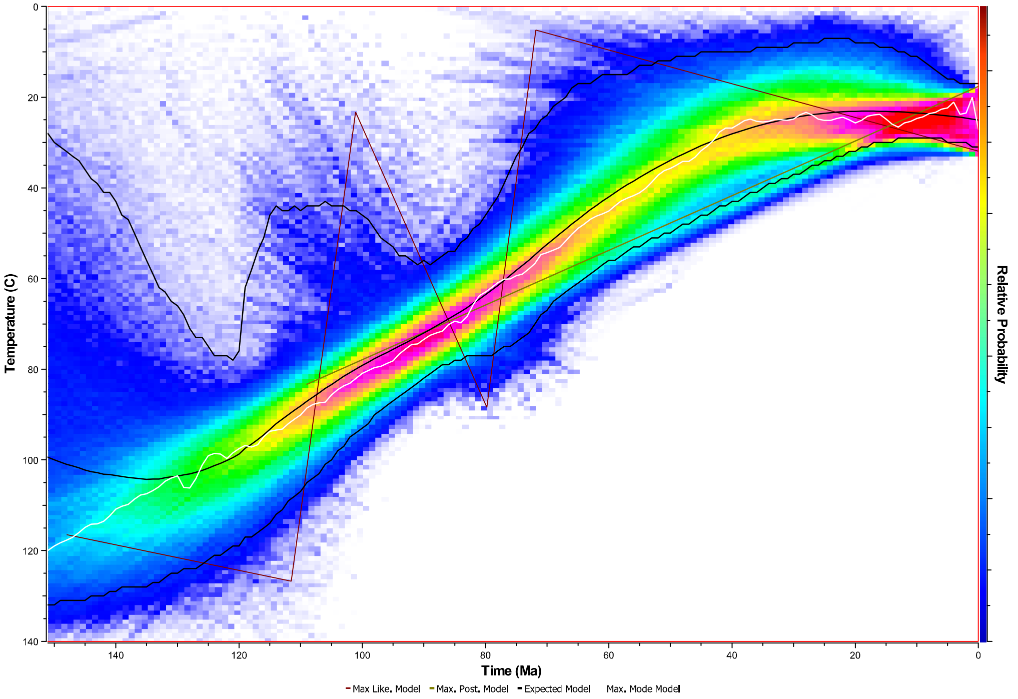

TJ42_NoConst_caxis_QTQt PC v 5.6.0 exe, runtime libraries and DocumentationTJ 42.txt 1.000m : Expected

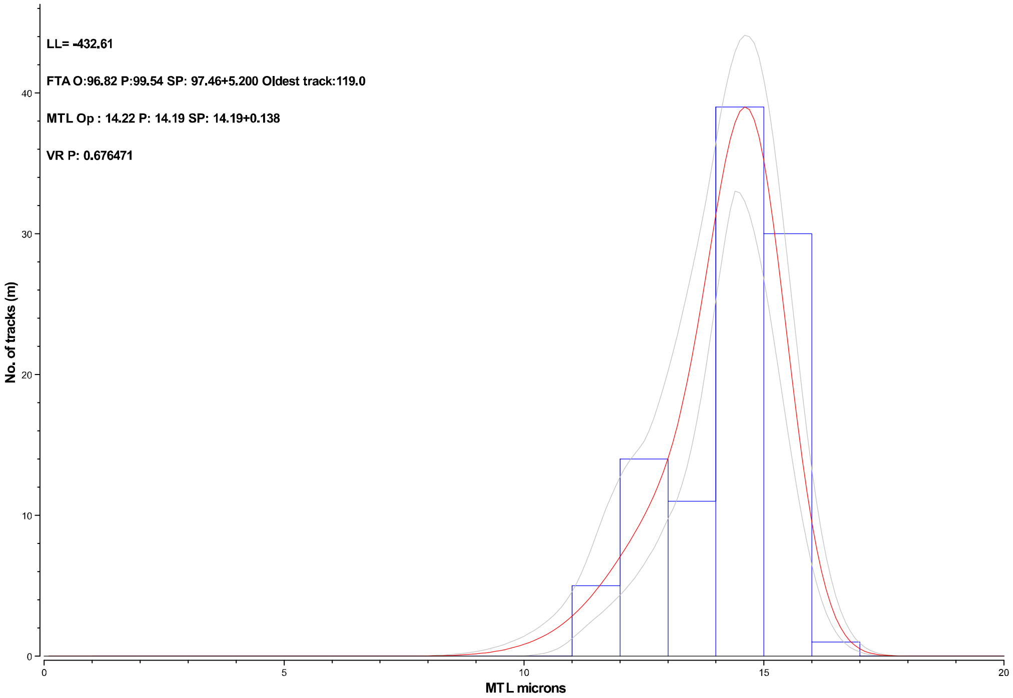

TJ42_NoConst_caxis_QTQt PC v 5.6.0 exe, runtime libraries and DocumentationTJ 42.txt 1.000m : Max. Like

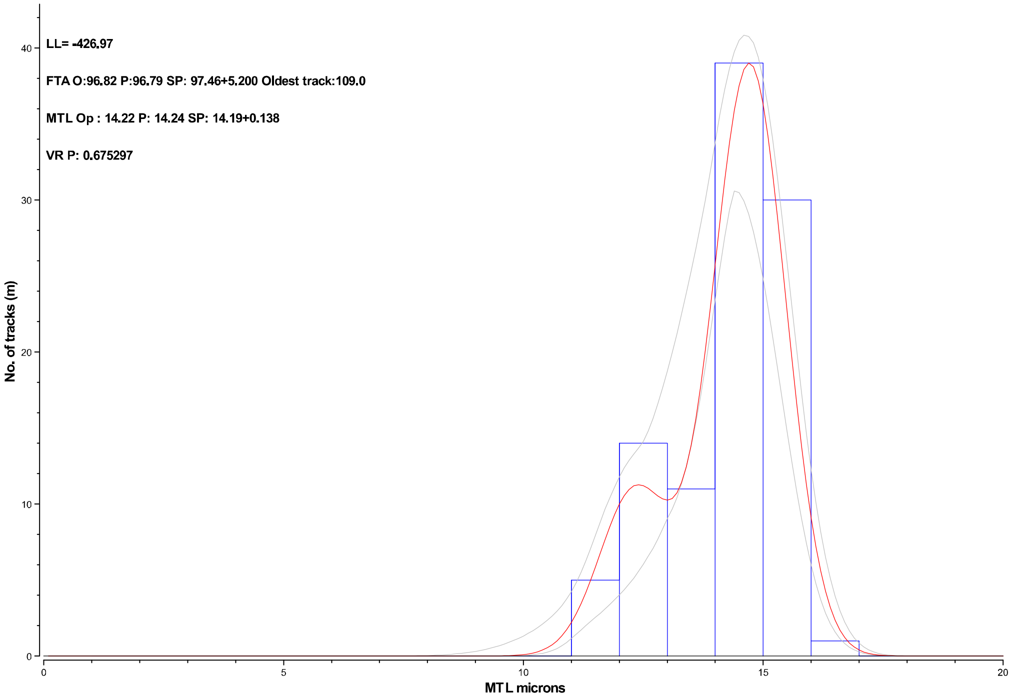

TJ42_NoConst_caxis_QTQt PC v 5.6.0 exe, runtime libraries and DocumentationTJ 42.txt 1.000m : Max Post

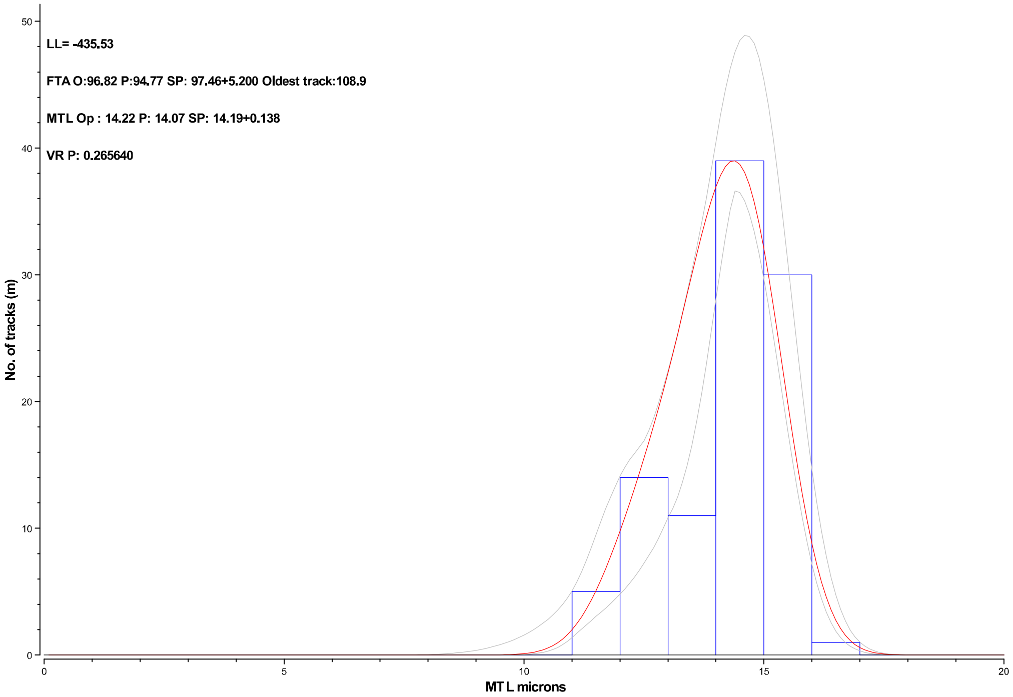

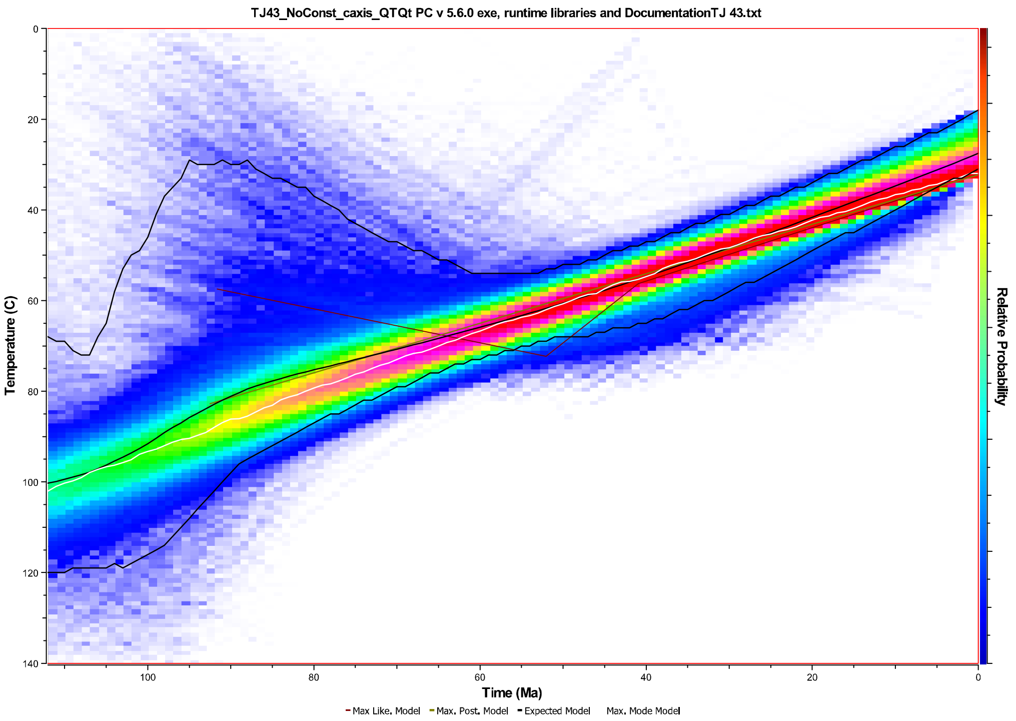

TJ43_NoConst_caxis_QTQt PC v 5.6.0 exe, runtime libraries and DocumentationTJ 43.txt
Temperature (C)
Time (Ma)
Relative Probability
Max Like. Model
Max. Post. Model
Expected Model
Max. Mode Model

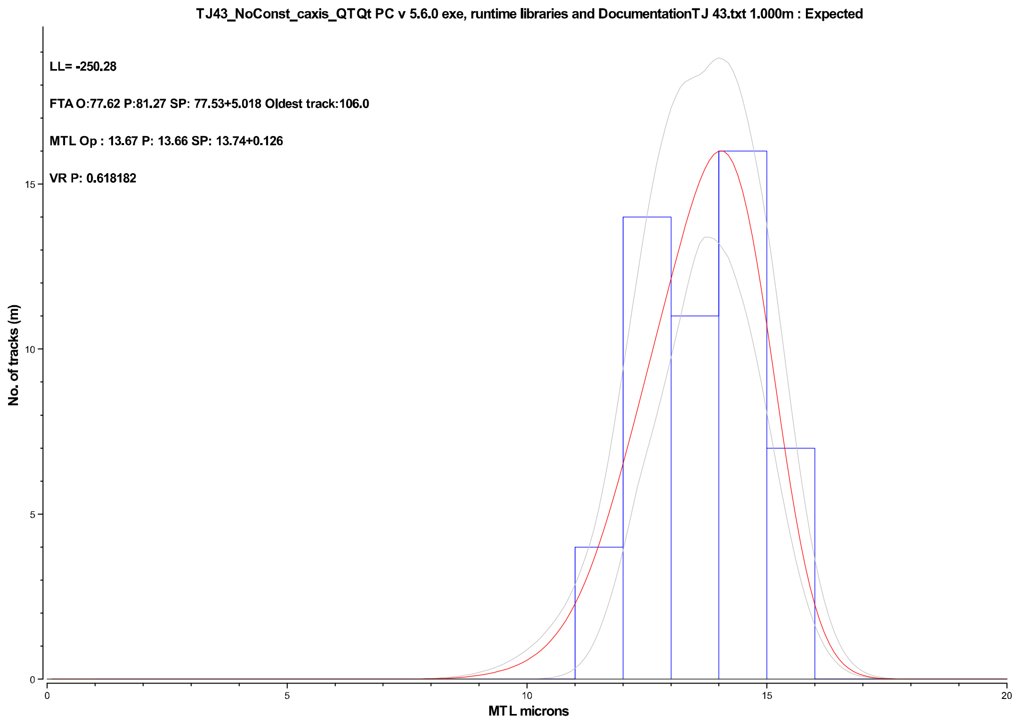

TJ43_NoConst_caxis_QTQt PC v 5.6.0 exe, runtime libraries and DocumentationTJ 43.txt 1.000m : Expected
LL= -250.28
FTA O:77.62 P:81.27 SP: 77.53+5.018 Oldest track:106.0
MTL Op : 13.67 P: 13.66 SP: 13.74+0.126
VR P: 0.618182
No. of tracks (m)
MTL microns

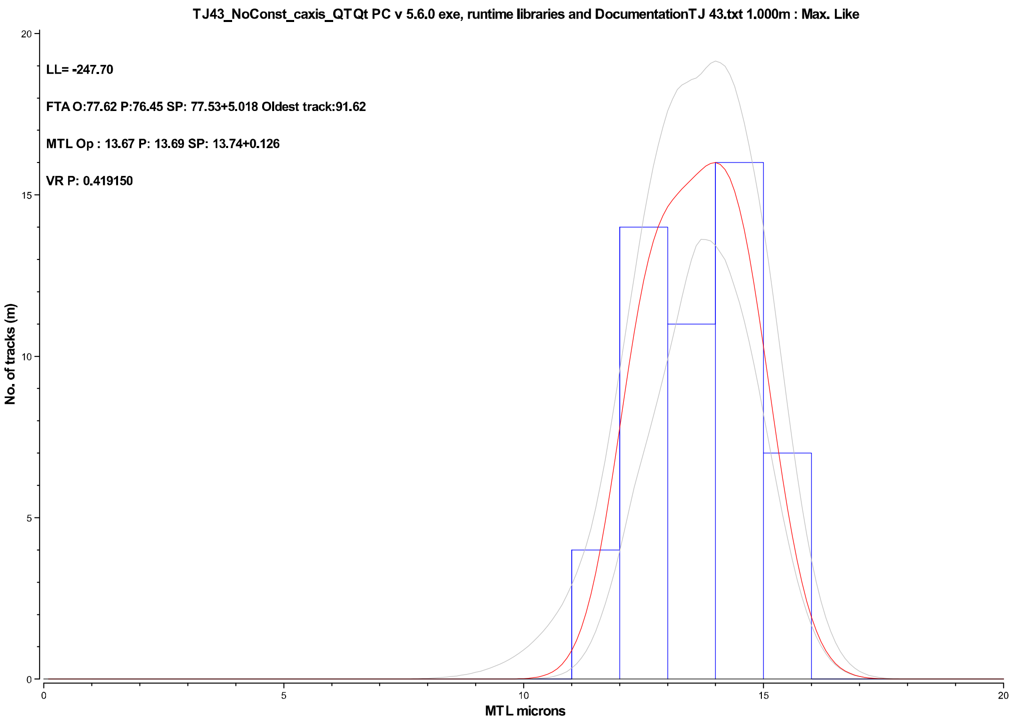

TJ43_NoConst_caxis_QTQt PC v 5.6.0 exe, runtime libraries and DocumentationTJ 43.txt 1.000m : Max. Like
LL= -247.70
FTA O:77.62 P:76.45 SP: 77.53+5.018 Oldest track:91.62
MTL Op : 13.67 P: 13.69 SP: 13.74+0.126
VR P: 0.419150
No. of tracks (m)
MTL microns

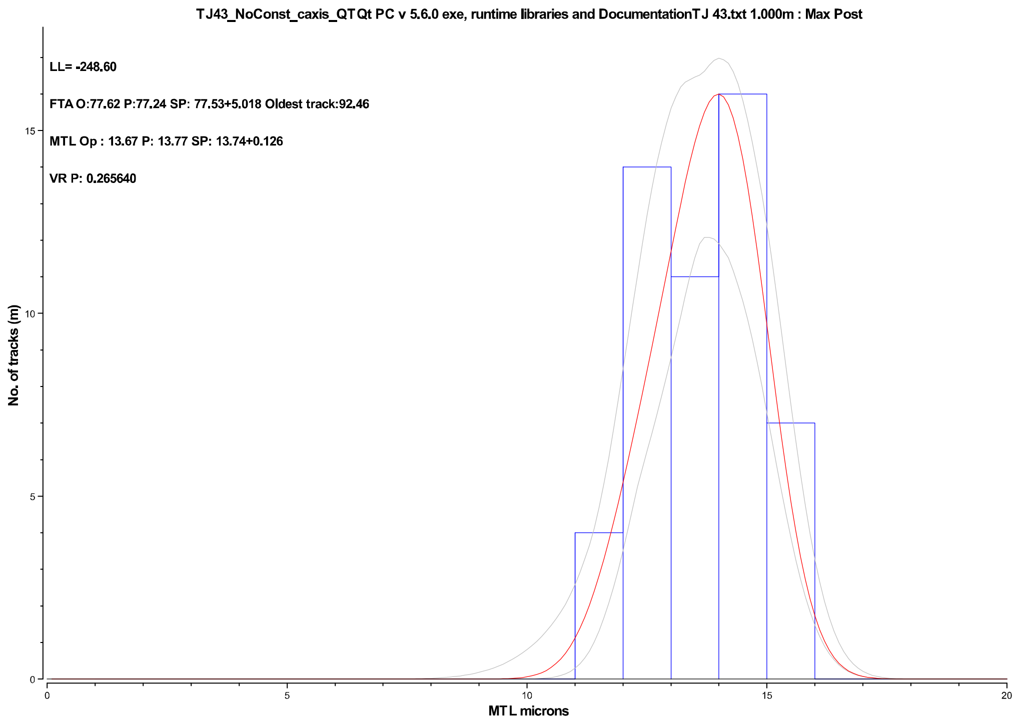

TJ43_NoConst_caxis_QTQt PC v 5.6.0 exe, runtime libraries and DocumentationTJ 43.txt 1.000m : Max Post
LL= -248.60
FTA O:77.62 P:77.24 SP: 77.53+5.018 Oldest track:92.46
MTL Op : 13.67 P: 13.77 SP: 13.74+0.126
VR P: 0.265640
No. of tracks (m)
MTL microns

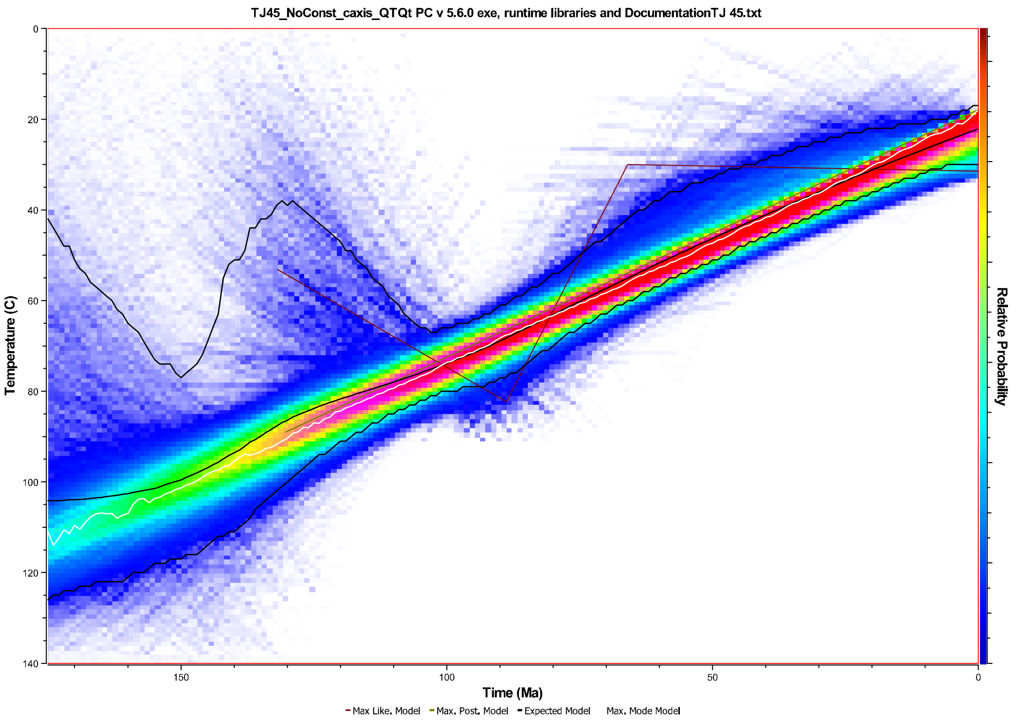
TJ45_NoConst_caxis_QTQt PC v 5.6.0 exe, runtime libraries and DocumentationTJ 45.txt
Temperature (C)
Time (Ma)
Relative Probability
Max Like. Model
Max. Post. Model
Expected Model
Max. Mode Model

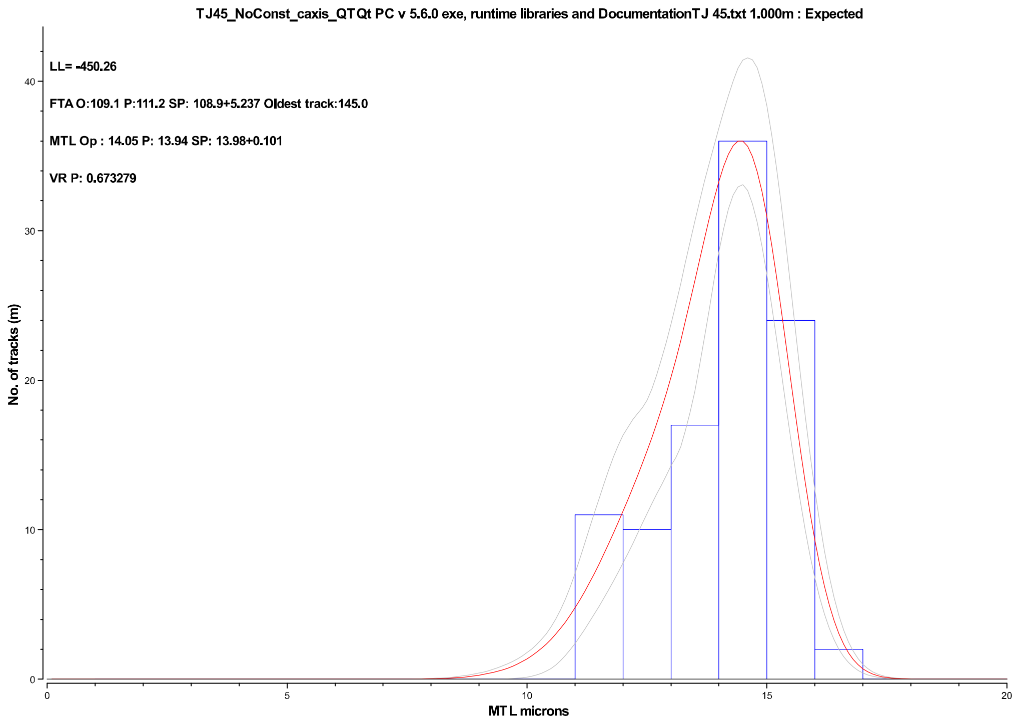
TJ45_NoConst_caxis_QTQt PC v 5.6.0 exe, runtime libraries and DocumentationTJ 45.txt 1.000m : Expected
LL= -450.26
FTA O:109.1 P:111.2 SP: 108.9+5.237 Oldest track:145.0
MTL Op : 14.05 P: 13.94 SP: 13.98+0.101
VR P: 0.673279
No. of tracks (m)
MTL microns

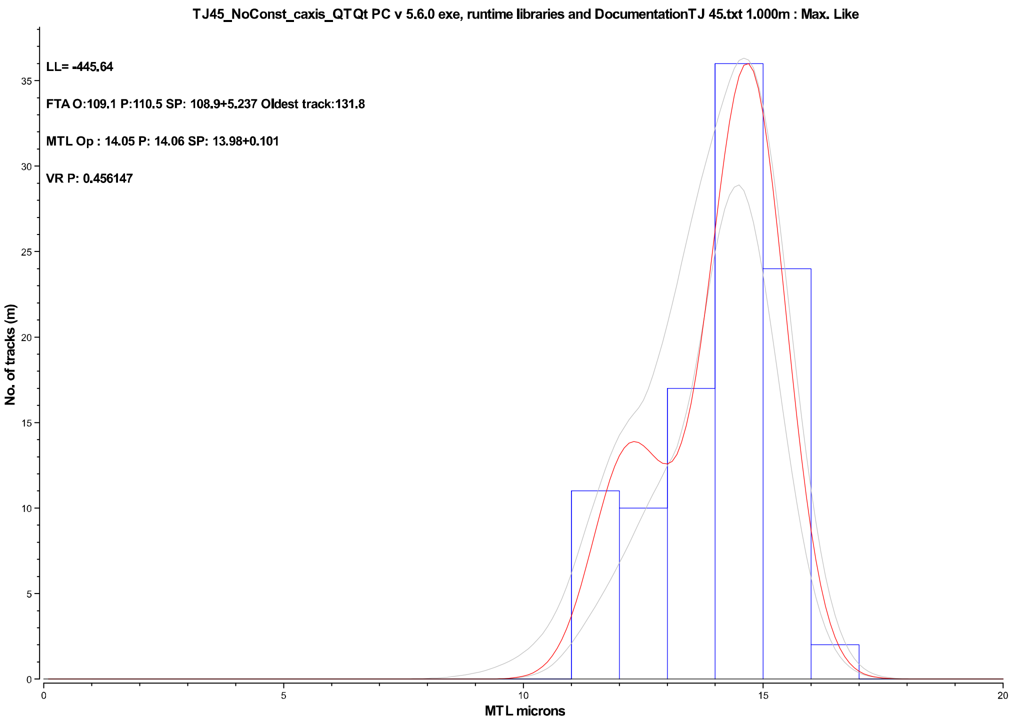
TJ45_NoConst_caxis_QTQt PC v 5.6.0 exe, runtime libraries and DocumentationTJ 45.txt 1.000m : Max. Like
LL= -445.64
FTA O:109.1 P:110.5 SP: 108.9+5.237 Oldest track:131.8
MTL Op : 14.05 P: 14.06 SP: 13.98+0.101
VR P: 0.456147
No. of tracks (m)
MTL microns

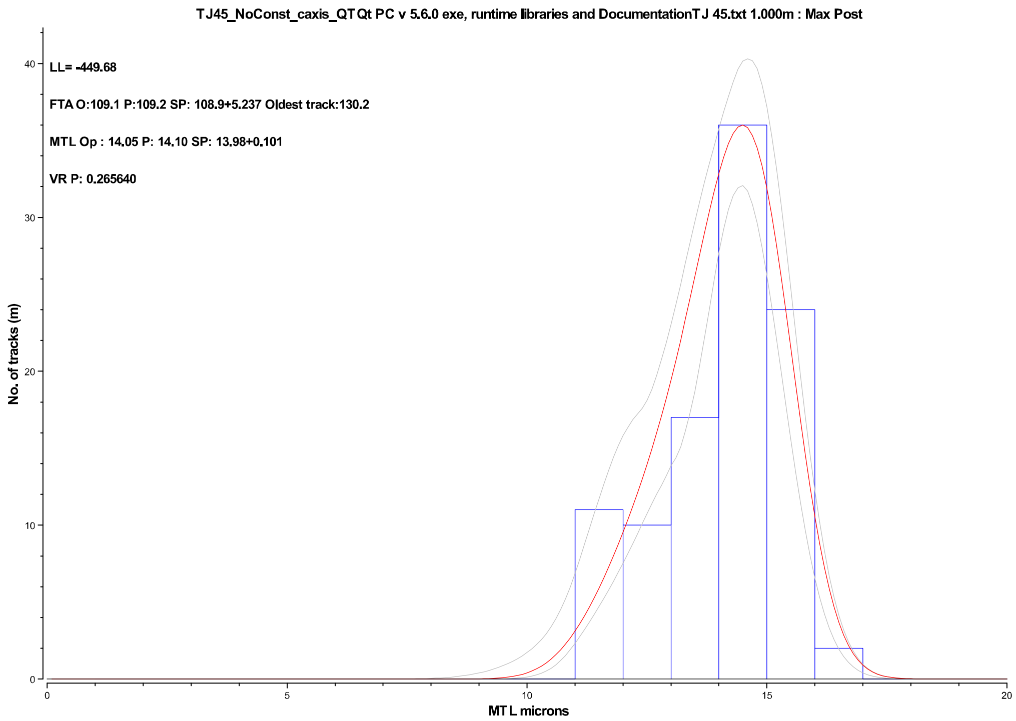
TJ45_NoConst_caxis_QTQt PC v 5.6.0 exe, runtime libraries and DocumentationTJ 45.txt 1.000m : Max Post
LL= -449.68
FTA O:109.1 P:109.2 SP: 108.9+5.237 Oldest track:130.2
MTL Op : 14.05 P: 14.10 SP: 13.98+0.101
VR P: 0.265640
No. of tracks (m)
MTL microns

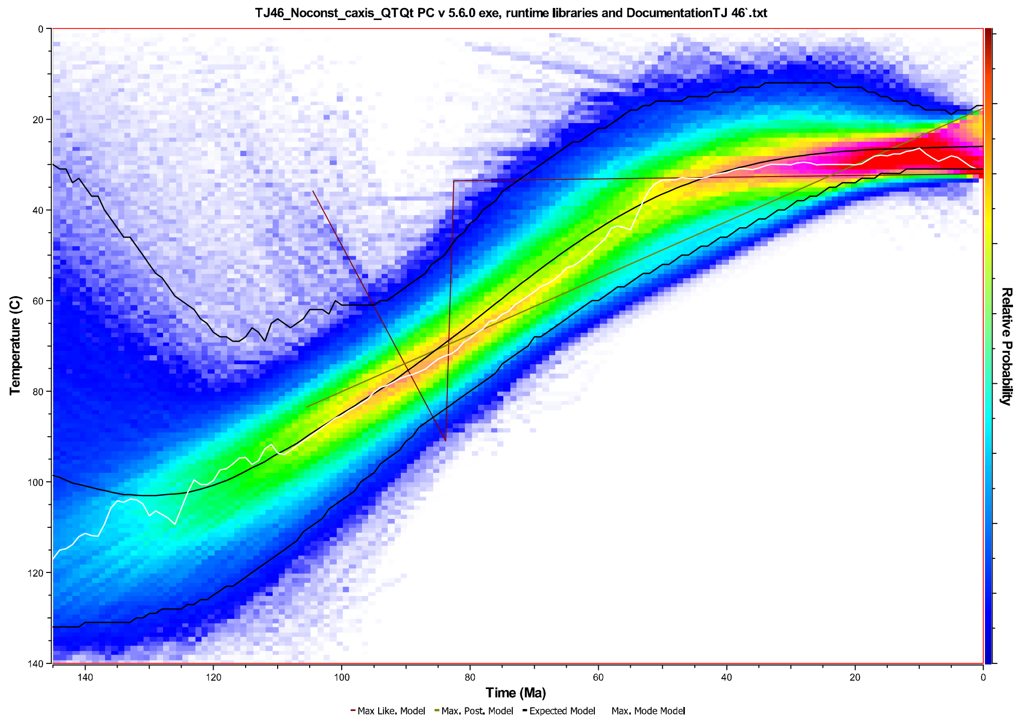
TJ46_Noconst_caxis_QTQt PC v 5.6.0 exe, runtime libraries and DocumentationTJ 46`.txt
Temperature (C)
Time (Ma)
Relative Probability
Max Like. Model
Max. Post. Model
Expected Model
Max. Mode Model

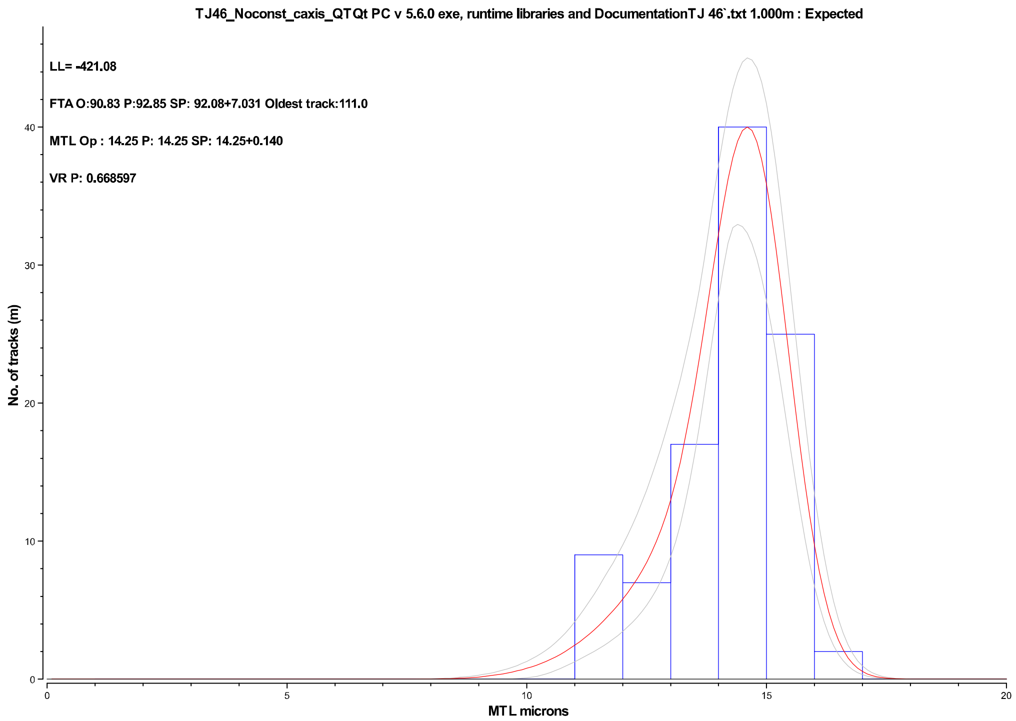
TJ46_Noconst_caxis_QTQt PC v 5.6.0 exe, runtime libraries and DocumentationTJ 46`.txt 1.000m : Expected
LL= -421.08
FTA O:90.83 P:92.85 SP: 92.08+7.031 Oldest track:111.0
MTL Op : 14.25 P: 14.25 SP: 14.25+0.140
VR P: 0.668597
No. of tracks (m)
MTL microns

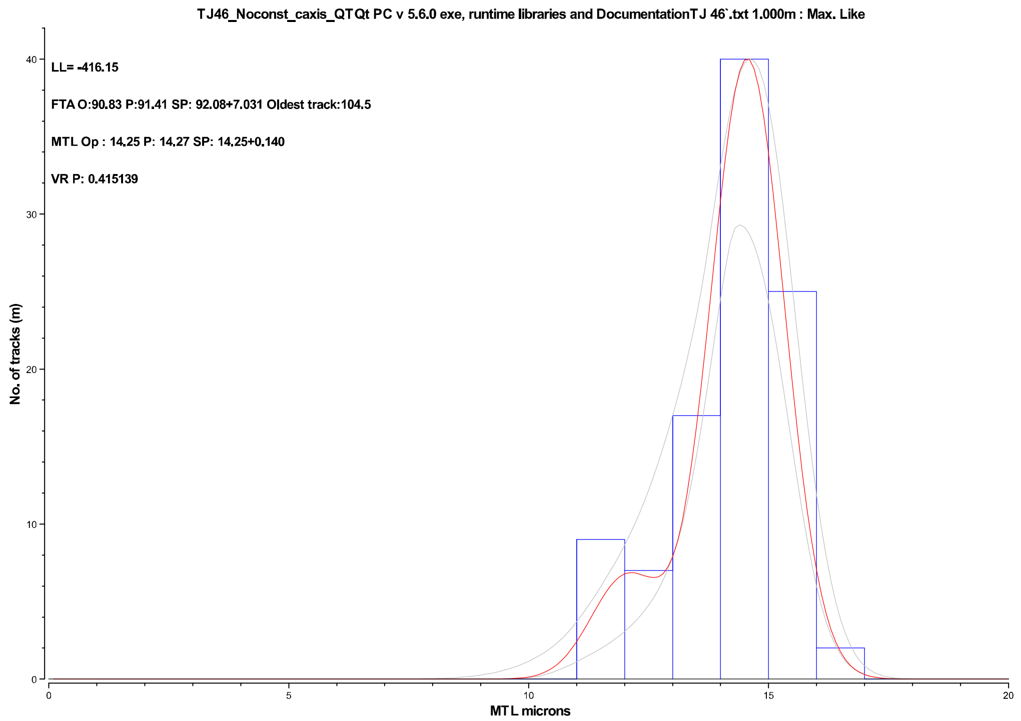
TJ46_Noconst_caxis_QTQt PC v 5.6.0 exe, runtime libraries and DocumentationTJ 46`.txt 1.000m : Max. Like
LL= -416.15
FTA O:90.83 P:91.41 SP: 92.08+7.031 Oldest track:104.5
MTL Op : 14.25 P: 14.27 SP: 14.25+0.140
VR P: 0.415139
No. of tracks (m)
MTL microns

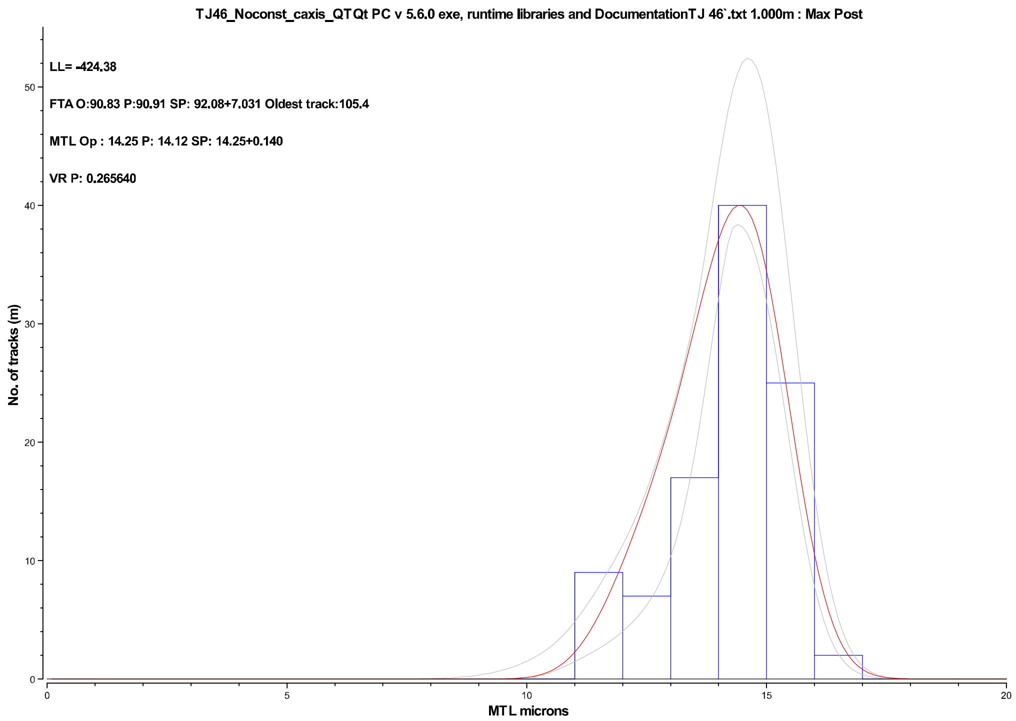
TJ46_Noconst_caxis_QTQt PC v 5.6.0 exe, runtime libraries and DocumentationTJ 46`.txt 1.000m : Max Post
LL= -424.38
FTA O:90.83 P:90.91 SP: 92.08+7.031 Oldest track:105.4
MTL Op : 14.25 P: 14.12 SP: 14.25+0.140
VR P: 0.265640
No. of tracks (m)
MTL microns

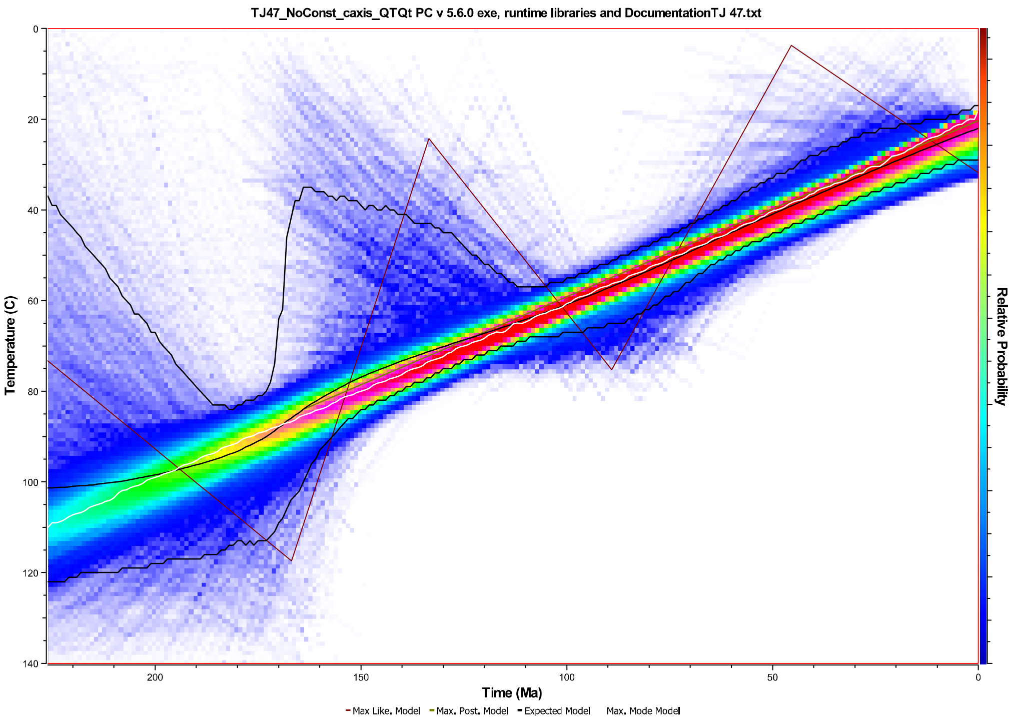
TJ47_NoConst_caxis_QTQt PC v 5.6.0 exe, runtime libraries and DocumentationTJ 47.txt
Temperature (C)
Time (Ma)
Relative Probability
Max Like. Model
Max. Post. Model
Expected Model
Max. Mode Model

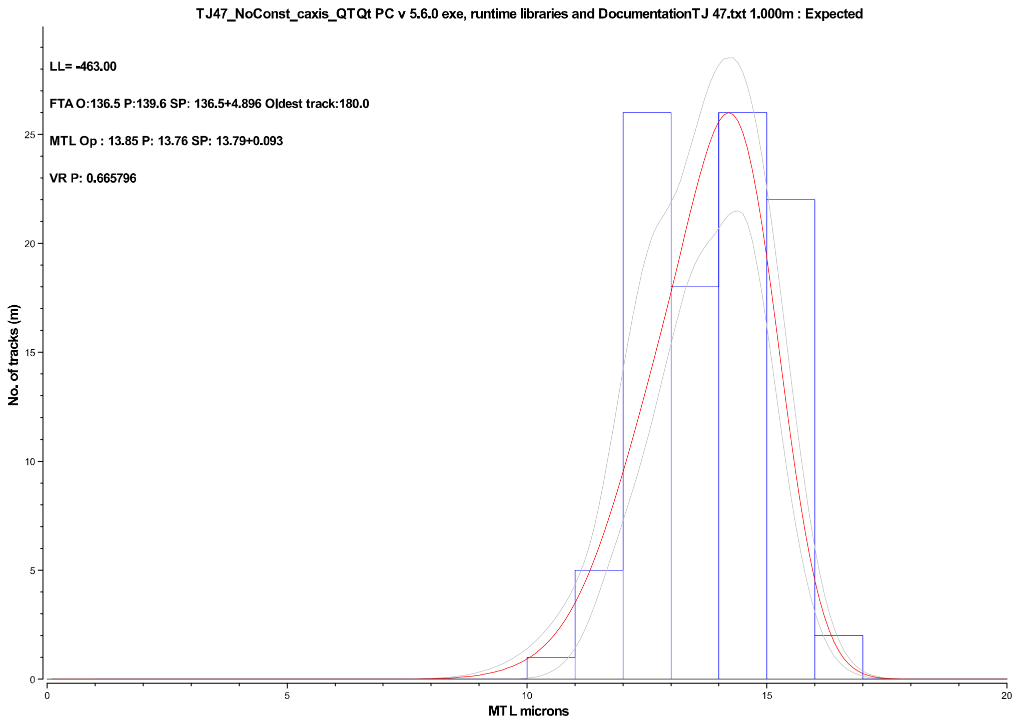
TJ47_NoConst_caxis_QTQt PC v 5.6.0 exe, runtime libraries and DocumentationTJ 47.txt 1.000m : Expected
LL= -463.00
FTA O:136.5 P:139.6 SP: 136.5+4.896 Oldest track:180.0
MTL Op : 13.85 P: 13.76 SP: 13.79+0.093
VR P: 0.665796
No. of tracks (m)
MTL microns

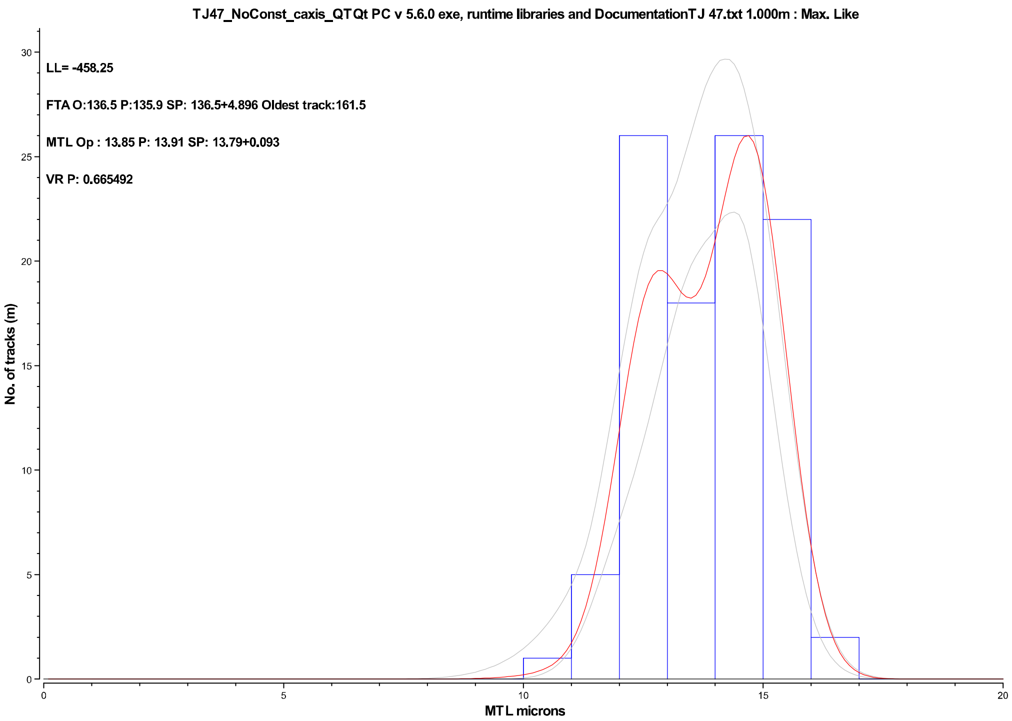
TJ47_NoConst_caxis_QTQt PC v 5.6.0 exe, runtime libraries and DocumentationTJ 47.txt 1.000m : Max. Like
LL= -458.25
FTA O:136.5 P:135.9 SP: 136.5+4.896 Oldest track:161.5
MTL Op : 13.85 P: 13.91 SP: 13.79+0.093
VR P: 0.665492
No. of tracks (m)
MTL microns

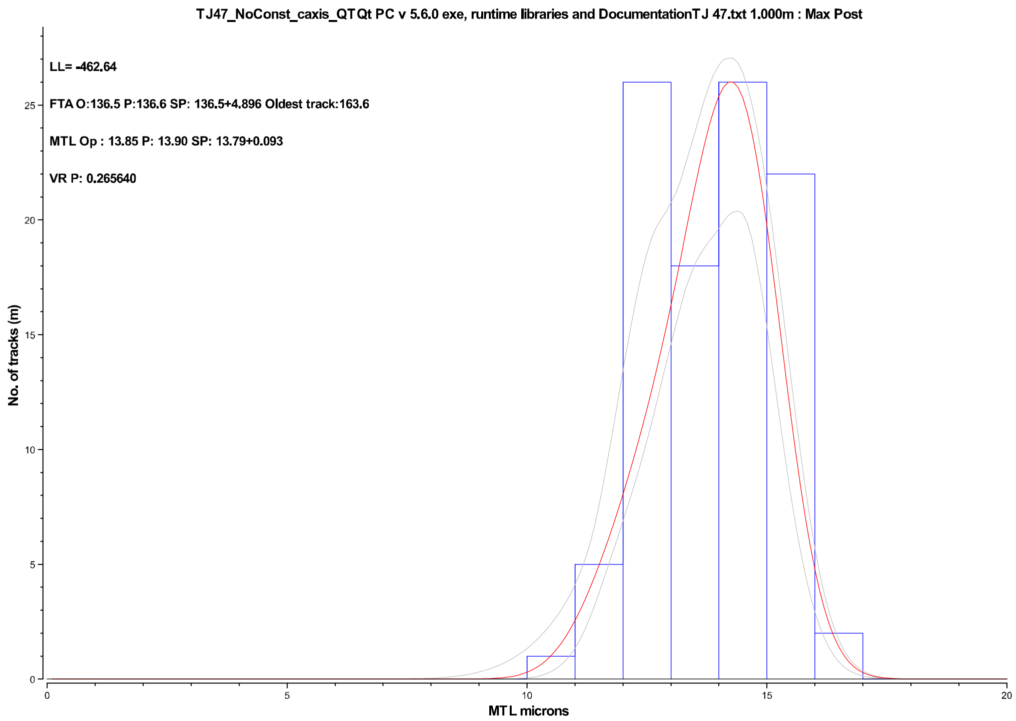
TJ47_NoConst_caxis_QTQt PC v 5.6.0 exe, runtime libraries and DocumentationTJ 47.txt 1.000m : Max Post
LL= -462.64
FTA O:136.5 P:136.6 SP: 136.5+4.896 Oldest track:163.6
MTL Op : 13.85 P: 13.90 SP: 13.79+0.093
VR P: 0.265640
No. of tracks (m)
MTL microns

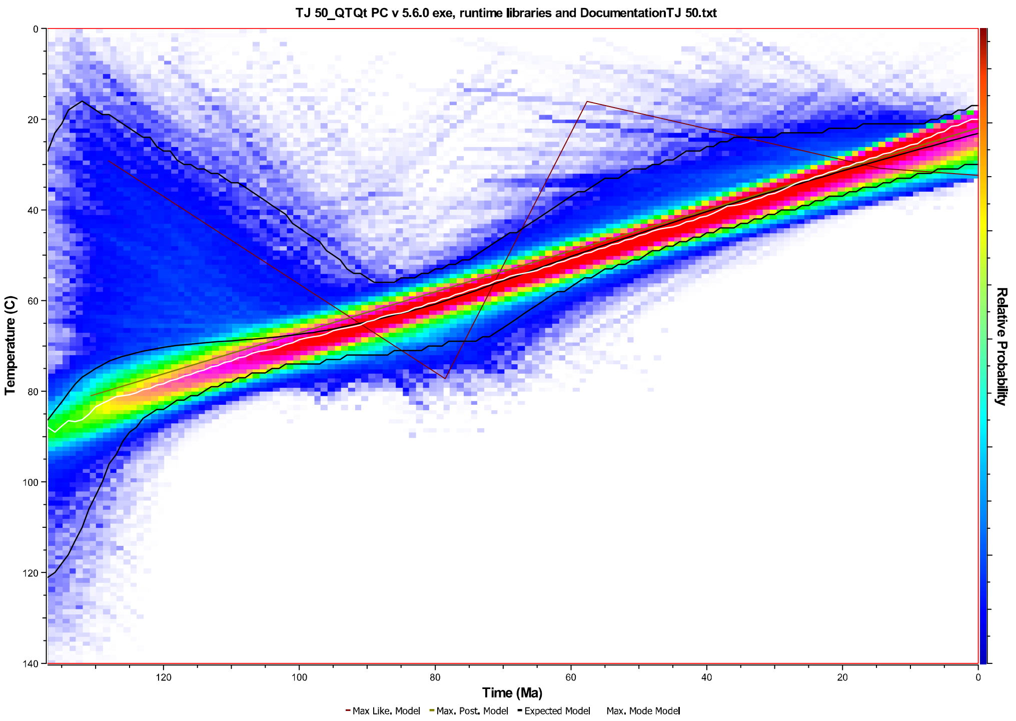
TJ 50_QTQt PC v 5.6.0 exe, runtime libraries and DocumentationTJ 50.txt
Temperature (C)
Time (Ma)
Relative Probability
Max Like. Model
Max. Post. Model
Expected Model
Max. Mode Model

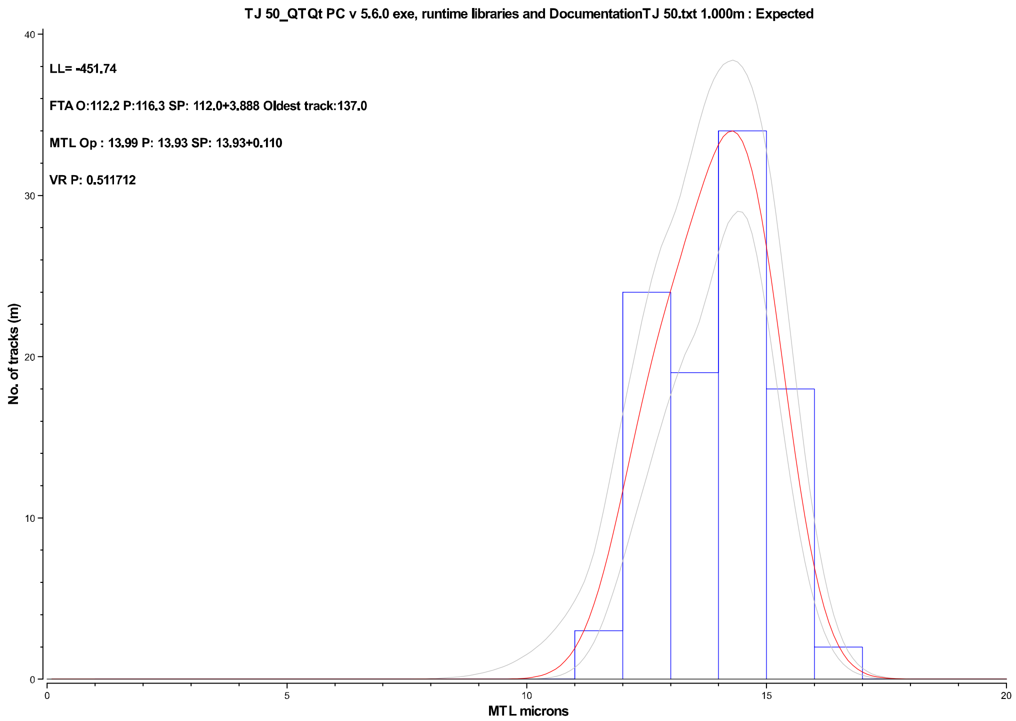
TJ 50_QTQt PC v 5.6.0 exe, runtime libraries and DocumentationTJ 50.txt 1.000m : Expected
LL= -451.74
FTA O:112.2 P:116.3 SP: 112.0+3.888 Oldest track:137.0
MTL Op : 13.99 P: 13.93 SP: 13.93+0.110
VR P: 0.511712
No. of tracks (m)
MTL microns

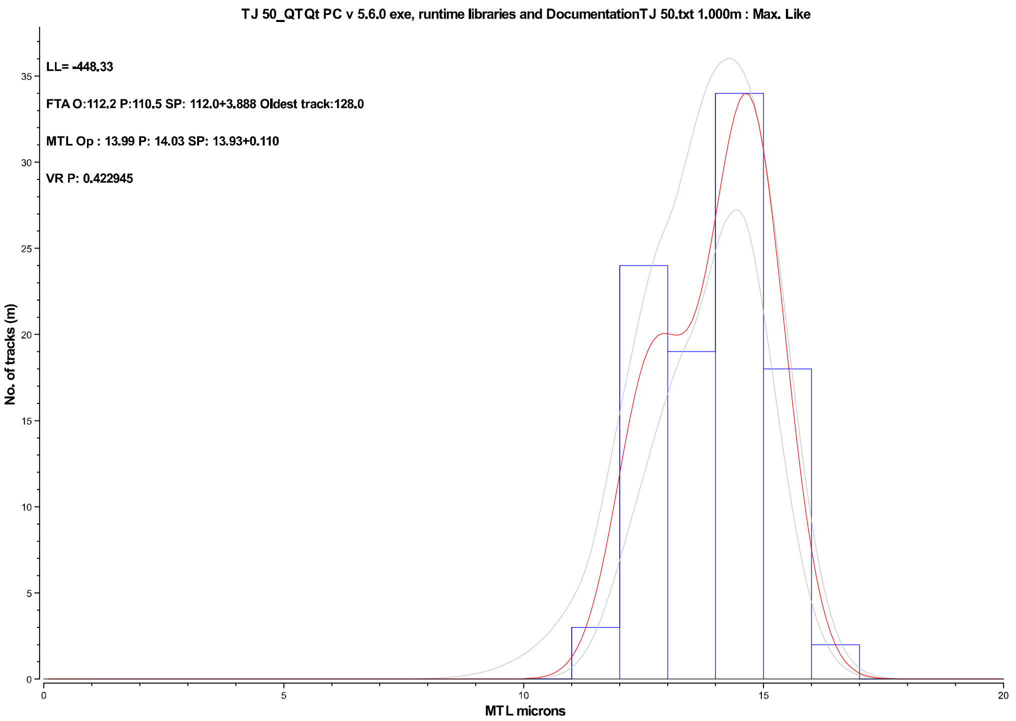
TJ 50_QTQt PC v 5.6.0 exe, runtime libraries and DocumentationTJ 50.txt 1.000m : Max. Like
LL= -448.33
FTA O:112.2 P:110.5 SP: 112.0+3.888 Oldest track:128.0
MTL Op : 13.99 P: 14.03 SP: 13.93+0.110
VR P: 0.422945
No. of tracks (m)
MTL microns

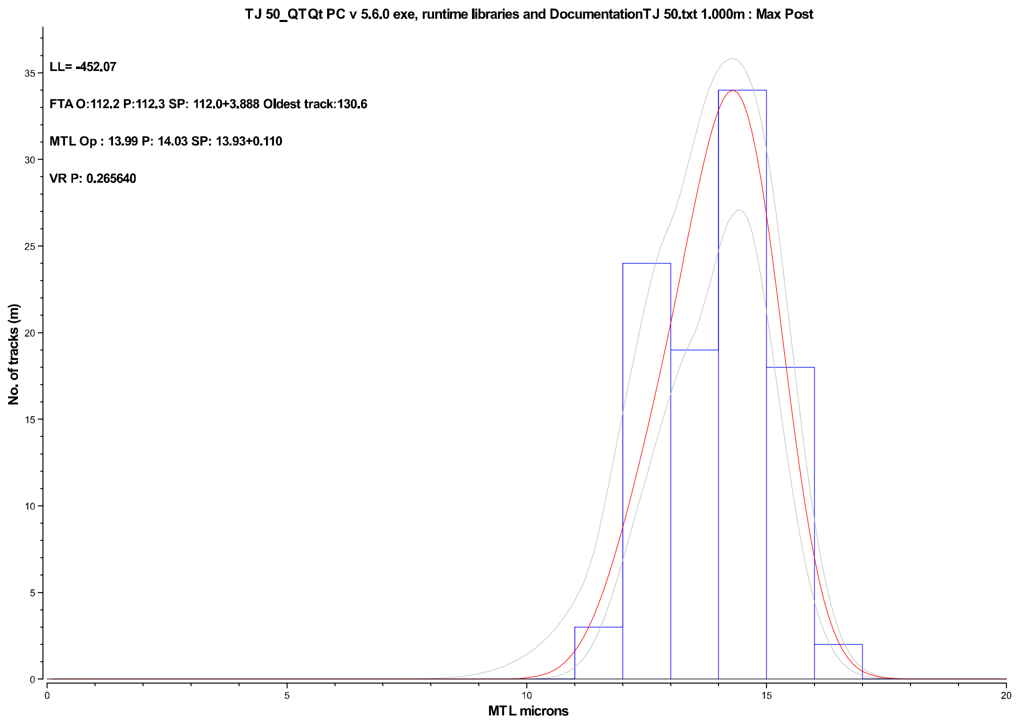
TJ 50_QTQt PC v 5.6.0 exe, runtime libraries and DocumentationTJ 50.txt 1.000m : Max Post
LL= -452.07
FTA O:112.2 P:112.3 SP: 112.0+3.888 Oldest track:130.6
MTL Op : 13.99 P: 14.03 SP: 13.93+0.110
VR P: 0.265640
No. of tracks (m)
MTL microns

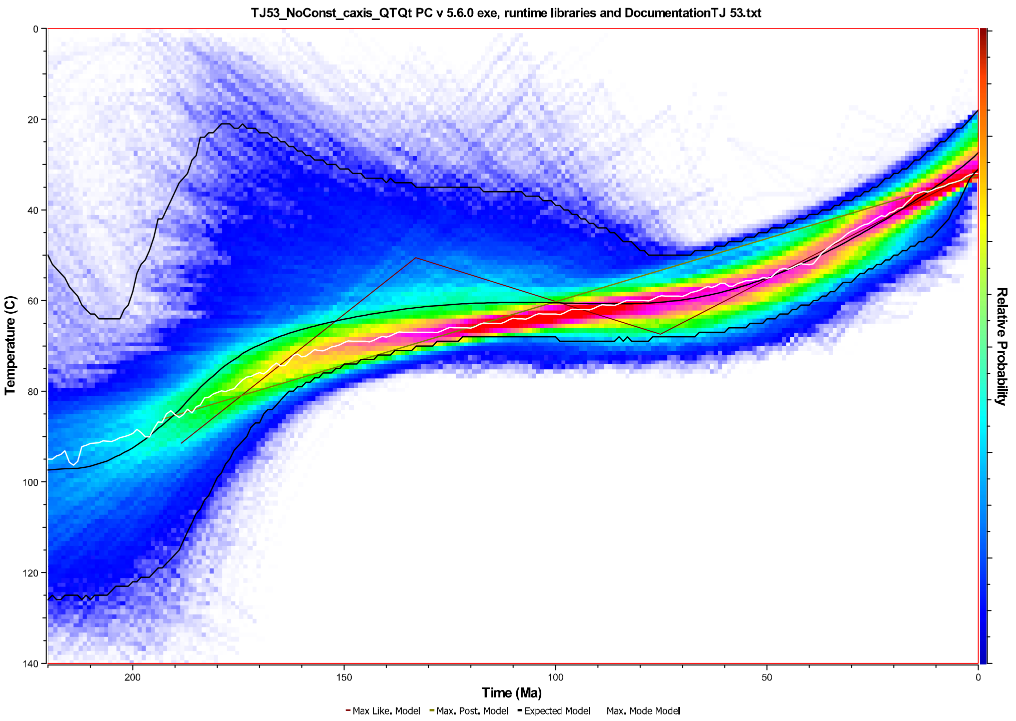

TJ53_NoConst_caxis_QTQt PC v 5.6.0 exe, runtime libraries and DocumentationTJ 53.txt
Temperature (C)
Time (Ma)
Relative Probability
Max Like. Model
Max. Post. Model
Expected Model
Max. Mode Model

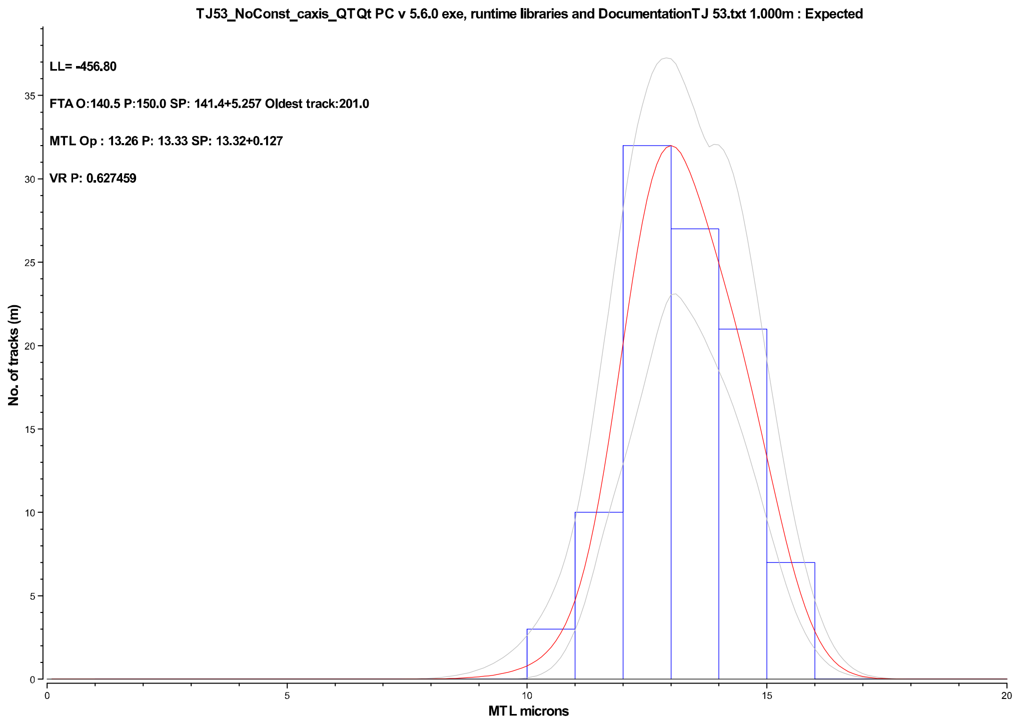

TJ53_NoConst_caxis_QTQt PC v 5.6.0 exe, runtime libraries and DocumentationTJ 53.txt 1.000m : Expected
LL= -456.80
FTA O:140.5 P:150.0 SP: 141.4+5.257 Oldest track:201.0
MTL Op : 13.26 P: 13.33 SP: 13.32+0.127
VR P: 0.627459
No. of tracks (m)
MTL microns

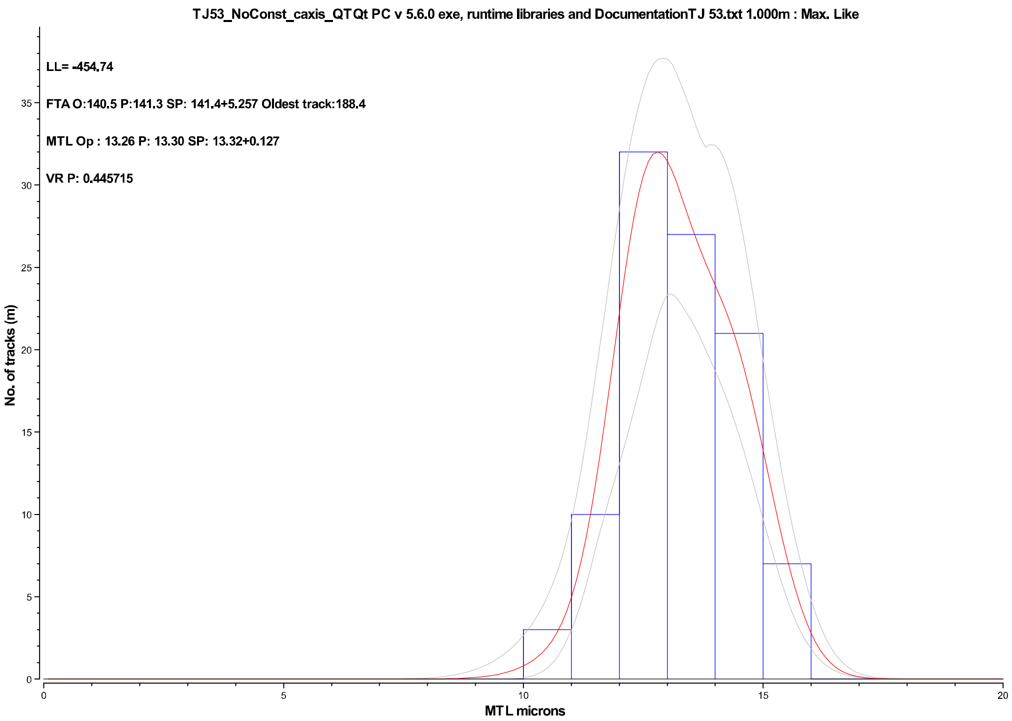

TJ53_NoConst_caxis_QTQt PC v 5.6.0 exe, runtime libraries and DocumentationTJ 53.txt 1.000m : Max. Like
LL= -454.74
FTA O:140.5 P:141.3 SP: 141.4+5.257 Oldest track:188.4
MTL Op : 13.26 P: 13.30 SP: 13.32+0.127
VR P: 0.445715
No. of tracks (m)
MTL microns

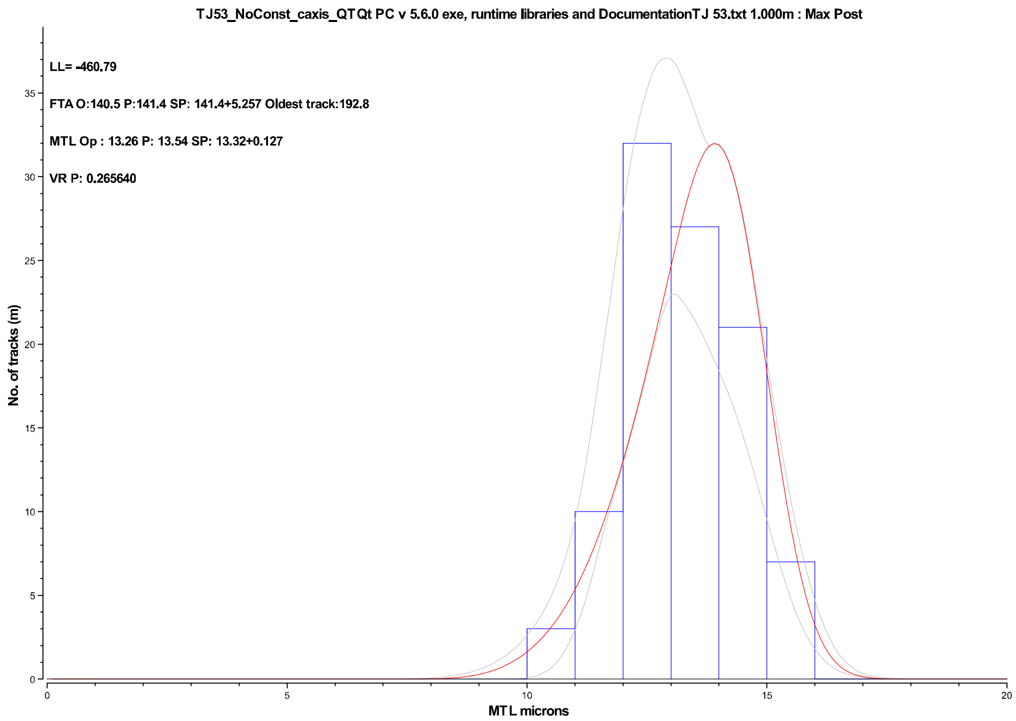

TJ53_NoConst_caxis_QTQt PC v 5.6.0 exe, runtime libraries and DocumentationTJ 53.txt 1.000m : Max Post
LL= -460.79
FTA O:140.5 P:141.4 SP: 141.4+5.257 Oldest track:192.8
MTL Op : 13.26 P: 13.54 SP: 13.32+0.127
VR P: 0.265640
No. of tracks (m)
MTL microns

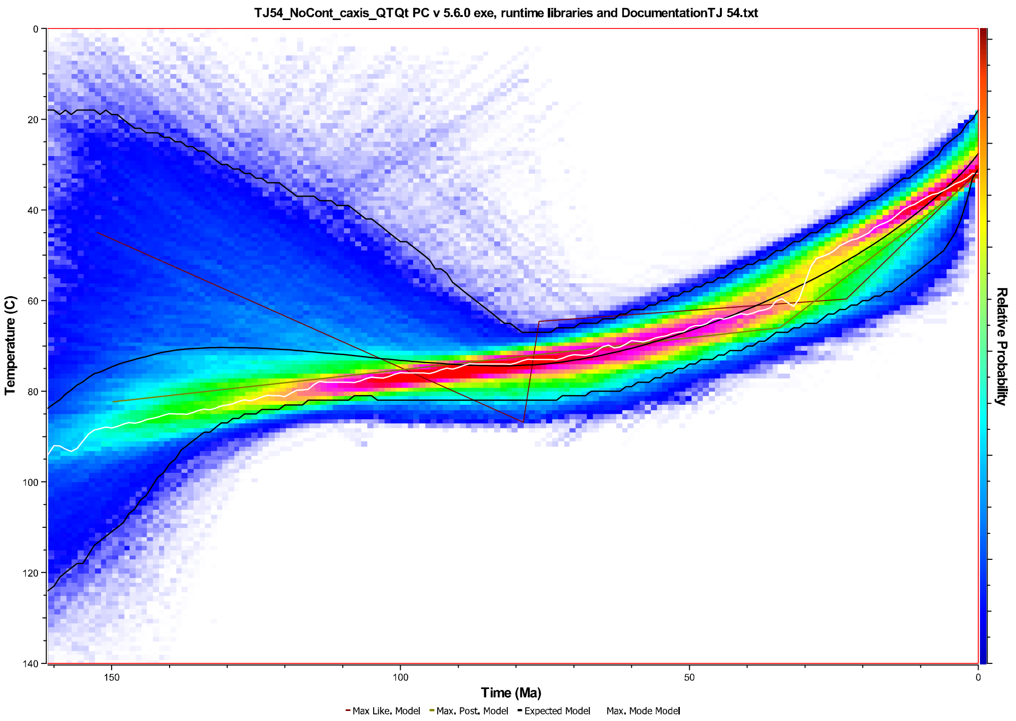
TJ54_NoCont_caxis_QTQt PC v 5.6.0 exe, runtime libraries and DocumentationTJ 54.txt
Temperature (C)
Time (Ma)
Relative Probability
Max Like. Model
Max. Post. Model
Expected Model
Max. Mode Model

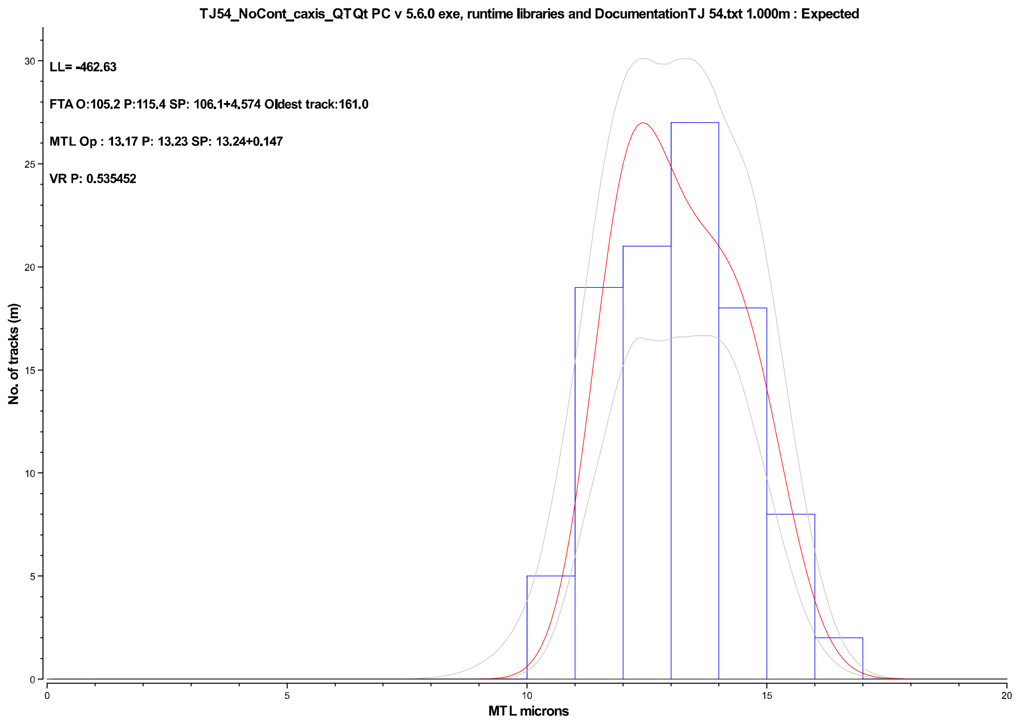
TJ54_NoCont_caxis_QTQt PC v 5.6.0 exe, runtime libraries and DocumentationTJ 54.txt 1.000m : Expected
LL= -462.63
FTA O:105.2 P:115.4 SP: 106.1+4.574 Oldest track:161.0
MTL Op : 13.17 P: 13.23 SP: 13.24+0.147
VR P: 0.535452
No. of tracks (m)
MTL microns

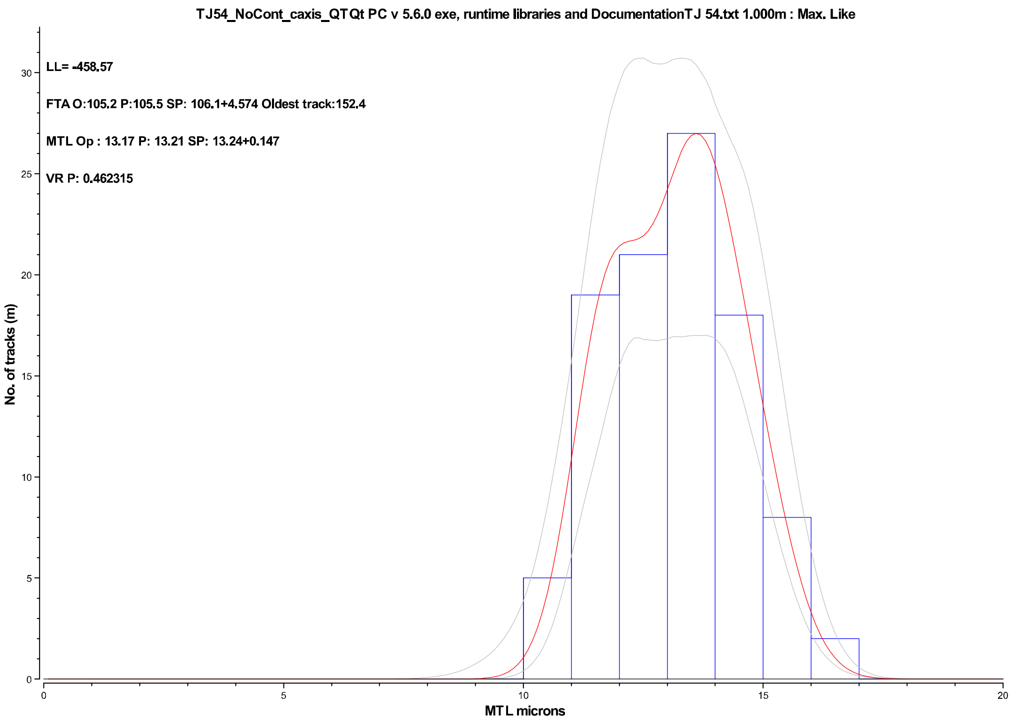
TJ54_NoCont_caxis_QTQt PC v 5.6.0 exe, runtime libraries and DocumentationTJ 54.txt 1.000m : Max. Like
LL= -458.57
FTA O:105.2 P:105.5 SP: 106.1+4.574 Oldest track:152.4
MTL Op : 13.17 P: 13.21 SP: 13.24+0.147
VR P: 0.462315
No. of tracks (m)
MTL microns

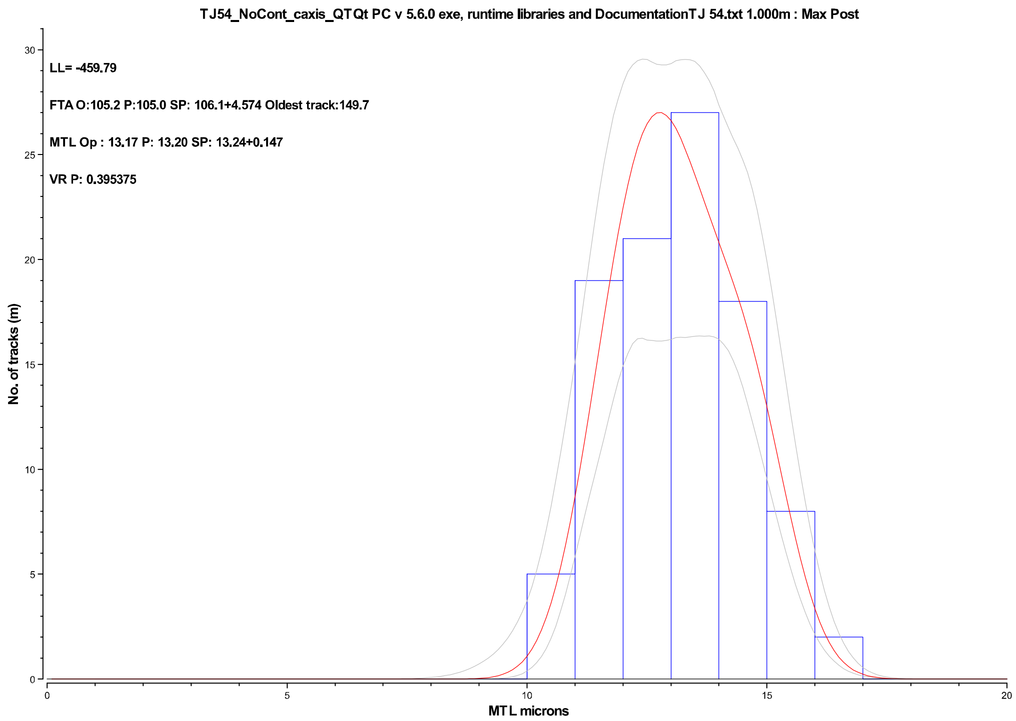
TJ54_NoCont_caxis_QTQt PC v 5.6.0 exe, runtime libraries and DocumentationTJ 54.txt 1.000m : Max Post
LL= -459.79
FTA O:105.2 P:105.0 SP: 106.1+4.574 Oldest track:149.7
MTL Op : 13.17 P: 13.20 SP: 13.24+0.147
VR P: 0.395375
No. of tracks (m)
MTL microns

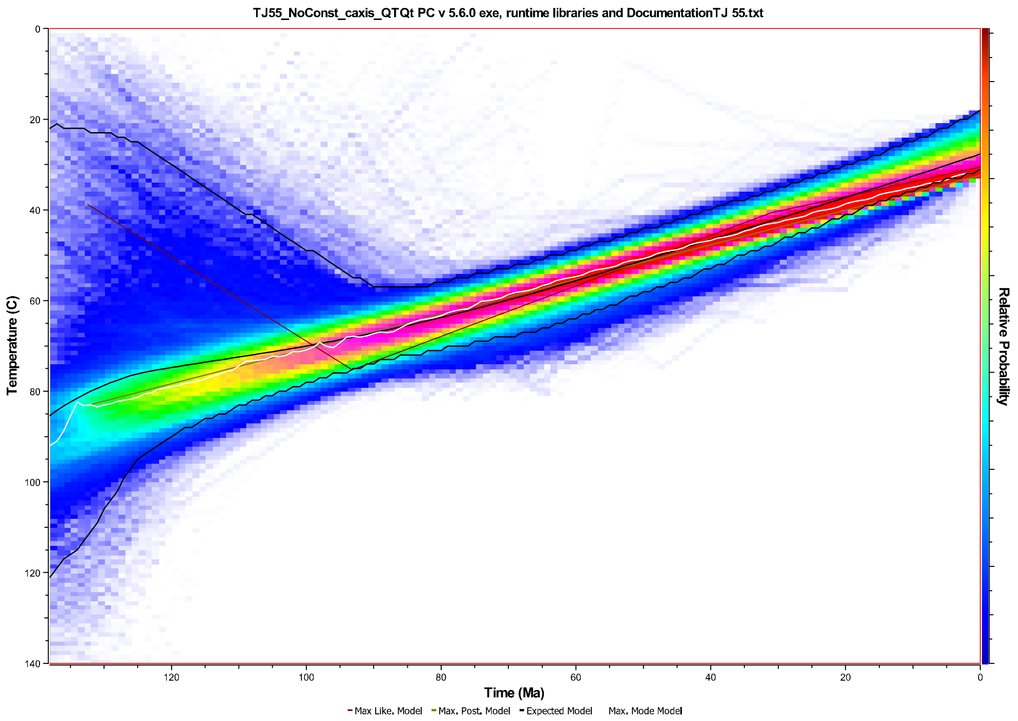
TJ55_NoConst_caxis_QTQt PC v 5.6.0 exe, runtime libraries and DocumentationTJ 55.txt
Temperature (C)
Time (Ma)
Relative Probability
Max Like. Model
Max. Post. Model
Expected Model
Max. Mode Model

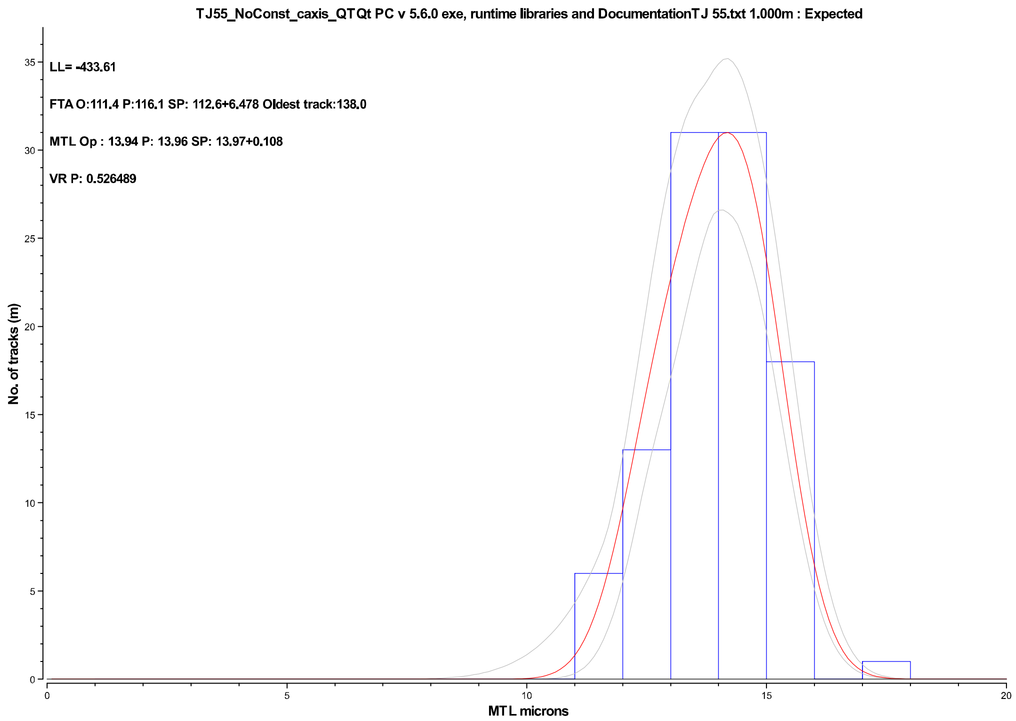
TJ55_NoConst_caxis_QTQt PC v 5.6.0 exe, runtime libraries and DocumentationTJ 55.txt 1.000m : Expected
LL= -433.61
FTA O:111.4 P:116.1 SP: 112.6+6.478 Oldest track:138.0
MTL Op : 13.94 P: 13.96 SP: 13.97+0.108
VR P: 0.526489
No. of tracks (m)
MTL microns

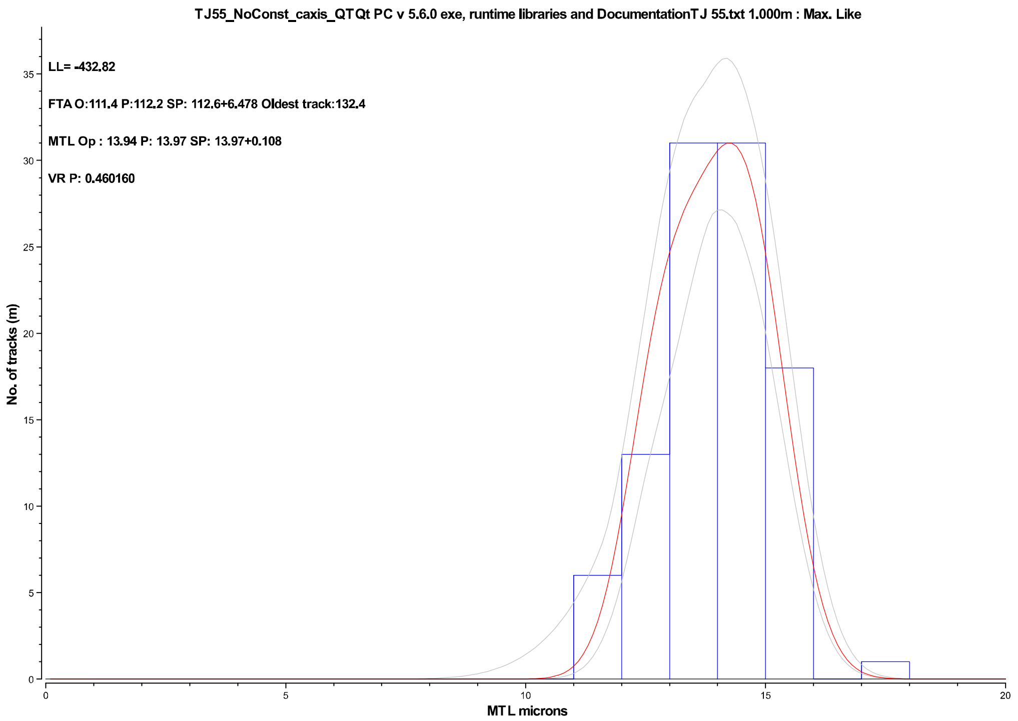
TJ55_NoConst_caxis_QTQt PC v 5.6.0 exe, runtime libraries and DocumentationTJ 55.txt 1.000m : Max. Like
LL= -432.82
FTA O:111.4 P:112.2 SP: 112.6+6.478 Oldest track:132.4
MTL Op : 13.94 P: 13.97 SP: 13.97+0.108
VR P: 0.460160
No. of tracks (m)
MTL microns

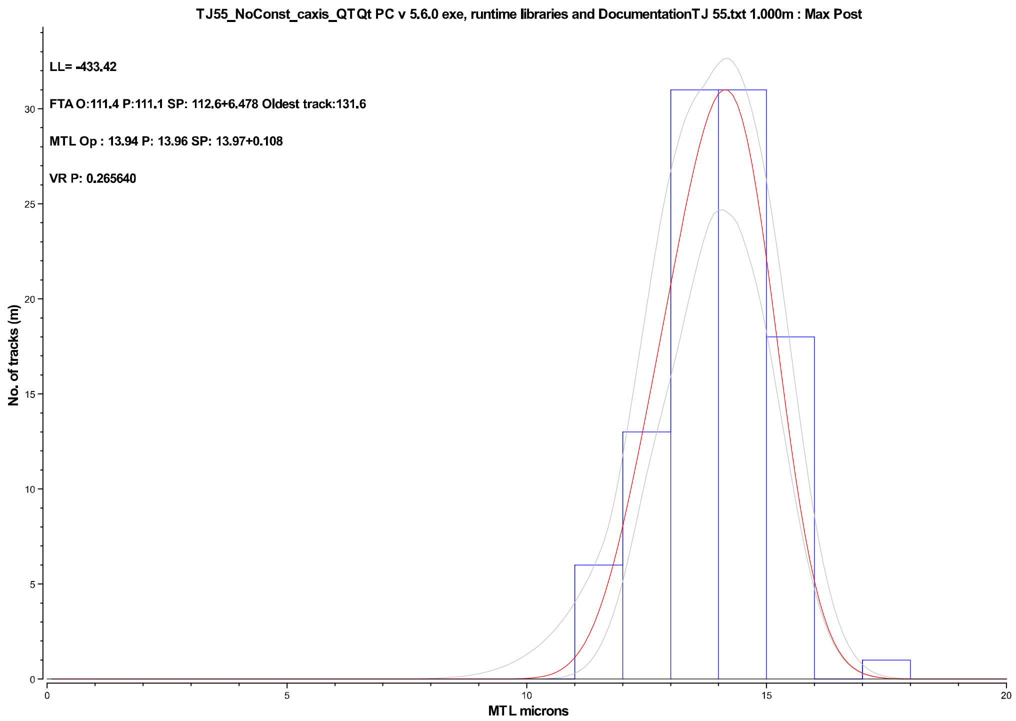
TJ55_NoConst_caxis_QTQt PC v 5.6.0 exe, runtime libraries and DocumentationTJ 55.txt 1.000m : Max Post
LL= -433.42
FTA O:111.4 P:111.1 SP: 112.6+6.478 Oldest track:131.6
MTL Op : 13.94 P: 13.96 SP: 13.97+0.108
VR P: 0.265640
No. of tracks (m)
MTL microns

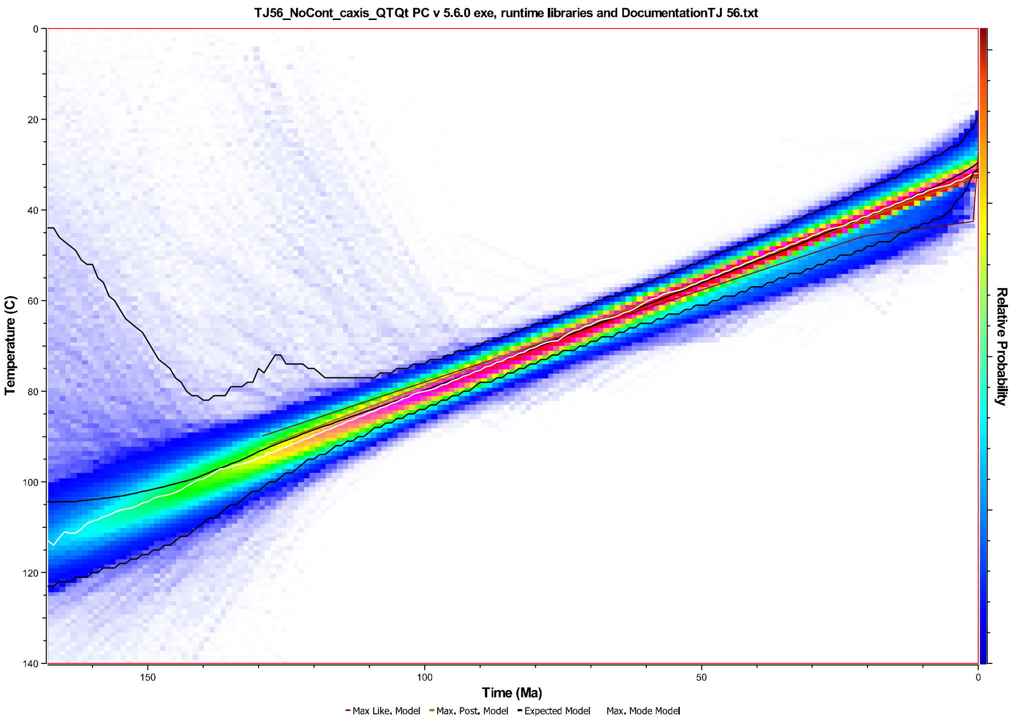
TJ56_NoCont_caxis_QTQt PC v 5.6.0 exe, runtime libraries and DocumentationTJ 56.txt
Temperature (C)
Time (Ma)
Relative Probability
Max Like. Model
Max. Post. Model
Expected Model
Max. Mode Model

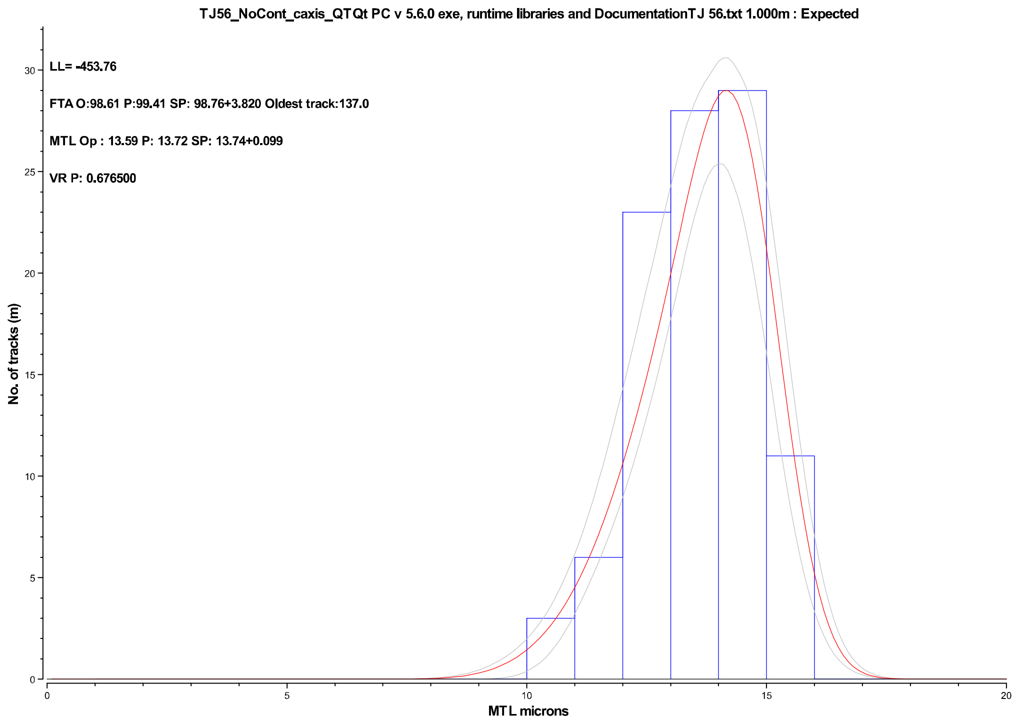
TJ56_NoCont_caxis_QTQt PC v 5.6.0 exe, runtime libraries and DocumentationTJ 56.txt 1.000m : Expected
LL= -453.76
FTA O:98.61 P:99.41 SP: 98.76+3.820 Oldest track:137.0
MTL Op : 13.59 P: 13.72 SP: 13.74+0.099
VR P: 0.676500
No. of tracks (m)
MTL microns

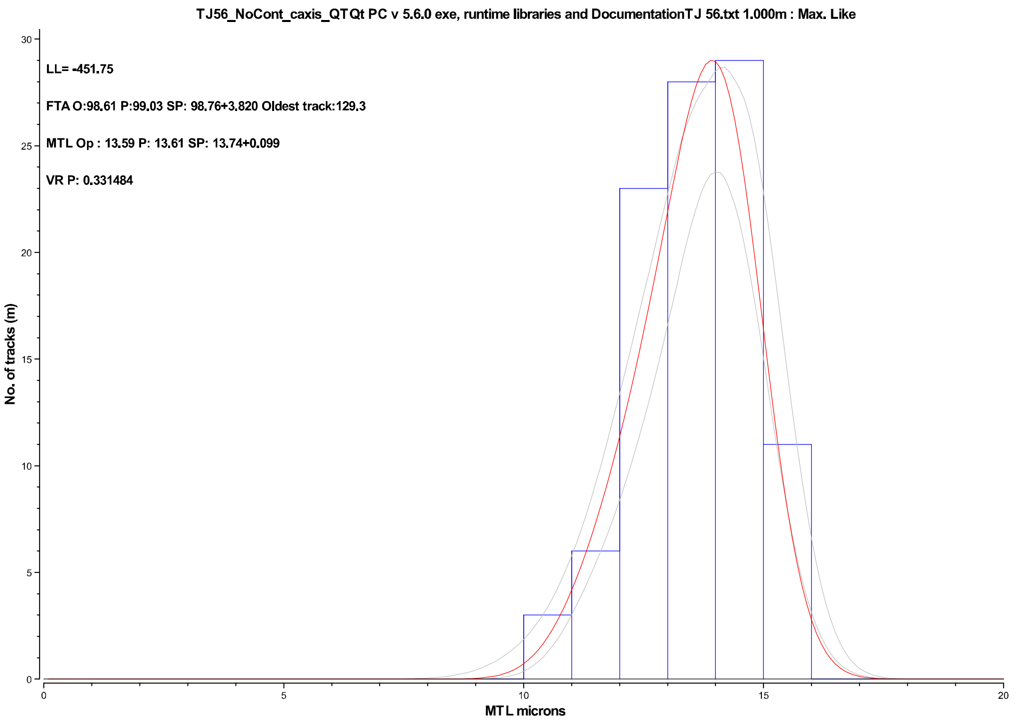
TJ56_NoCont_caxis_QTQt PC v 5.6.0 exe, runtime libraries and DocumentationTJ 56.txt 1.000m : Max. Like
LL= -451.75
FTA O:98.61 P:99.03 SP: 98.76+3.820 Oldest track:129.3
MTL Op : 13.59 P: 13.61 SP: 13.74+0.099
VR P: 0.331484
No. of tracks (m)
MTL microns

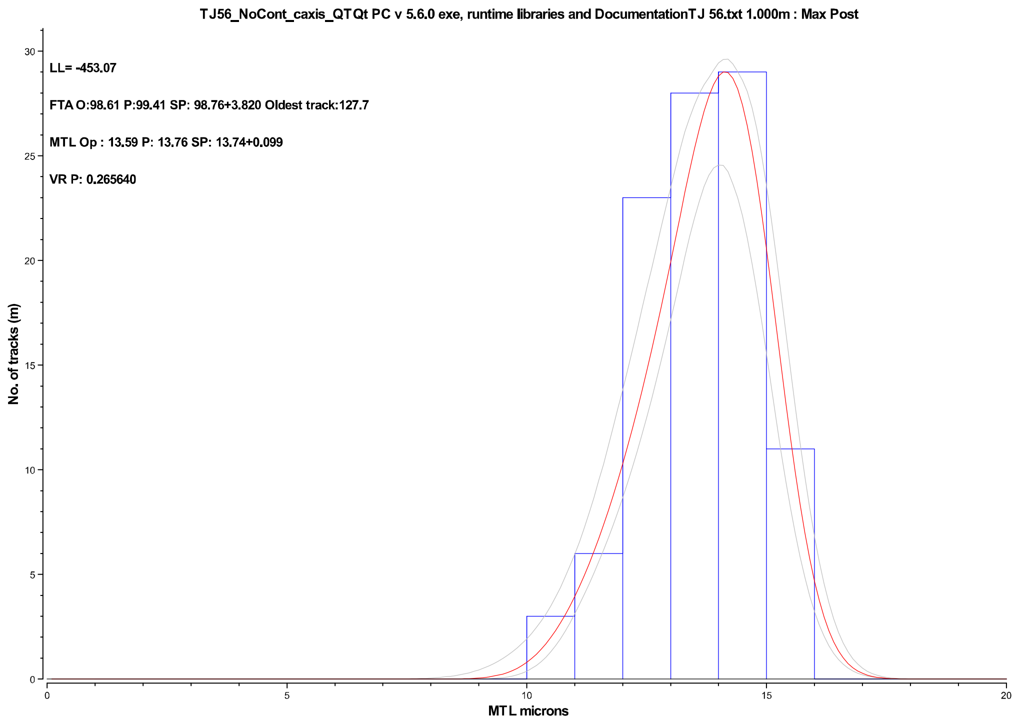
TJ56_NoCont_caxis_QTQt PC v 5.6.0 exe, runtime libraries and DocumentationTJ 56.txt 1.000m : Max Post
LL= -453.07
FTA O:98.61 P:99.41 SP: 98.76+3.820 Oldest track:127.7
MTL Op : 13.59 P: 13.76 SP: 13.74+0.099
VR P: 0.265640
No. of tracks (m)
MTL microns

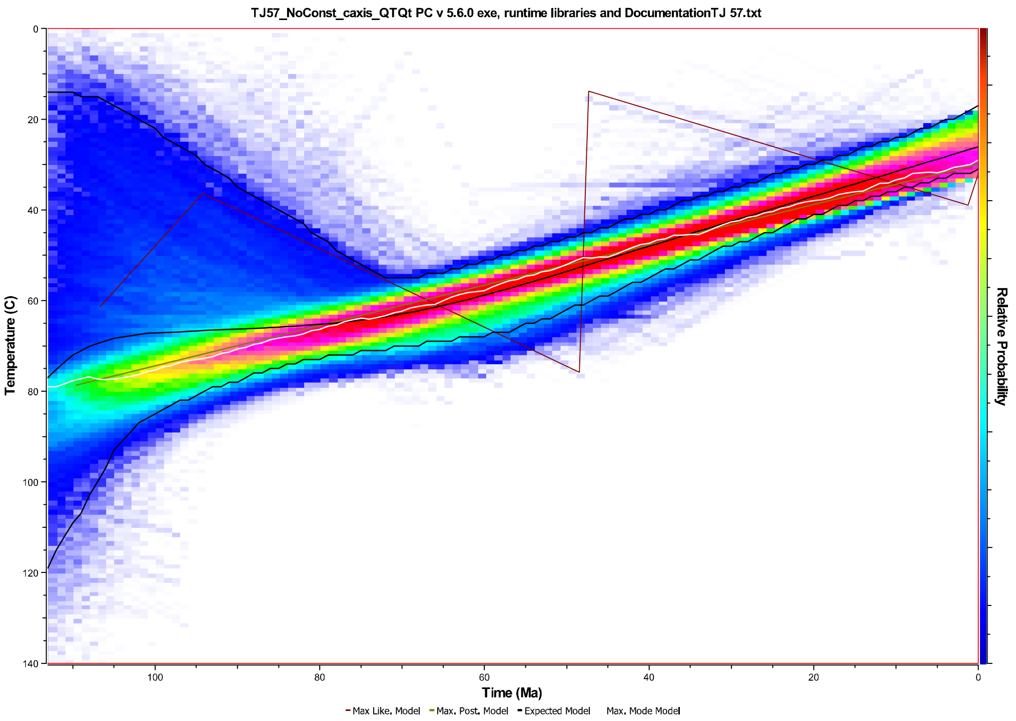
TJ57_NoConst_caxis_QTQt PC v 5.6.0 exe, runtime libraries and DocumentationTJ 57.txt
Temperature (C)
Time (Ma)
Relative Probability
Max Like. Model
Max. Post. Model
Expected Model
Max. Mode Model

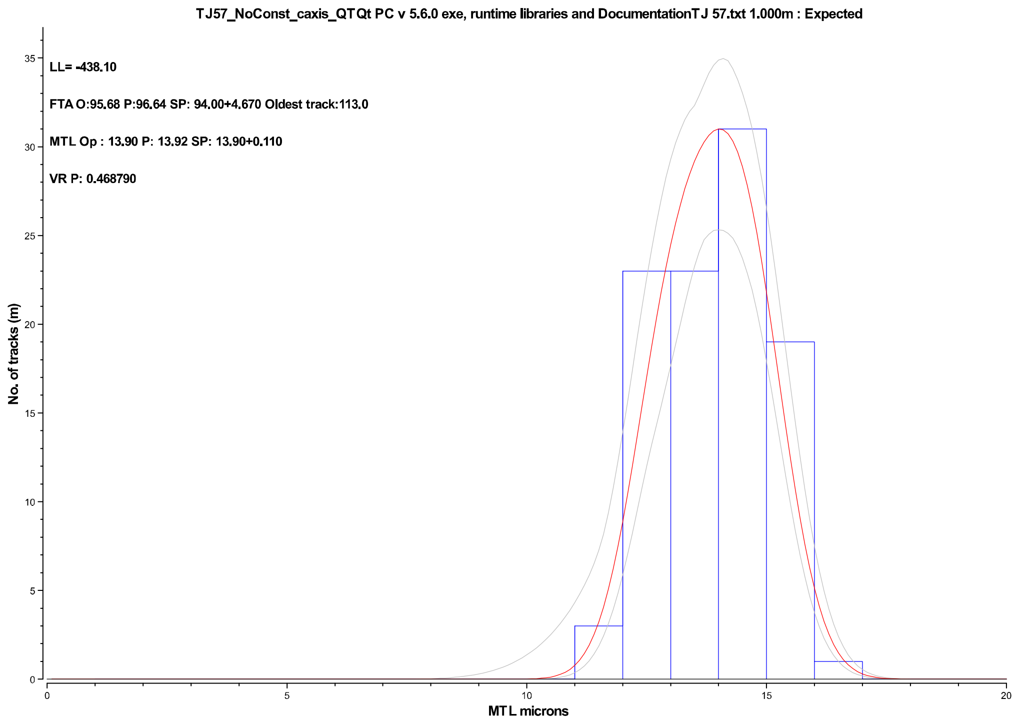
TJ57_NoConst_caxis_QTQt PC v 5.6.0 exe, runtime libraries and DocumentationTJ 57.txt 1.000m : Expected
LL= -438.10
FTA O:95.68 P:96.64 SP: 94.00+4.670 Oldest track:113.0
MTL Op : 13.90 P: 13.92 SP: 13.90+0.110
VR P: 0.468790
No. of tracks (m)
MTL microns

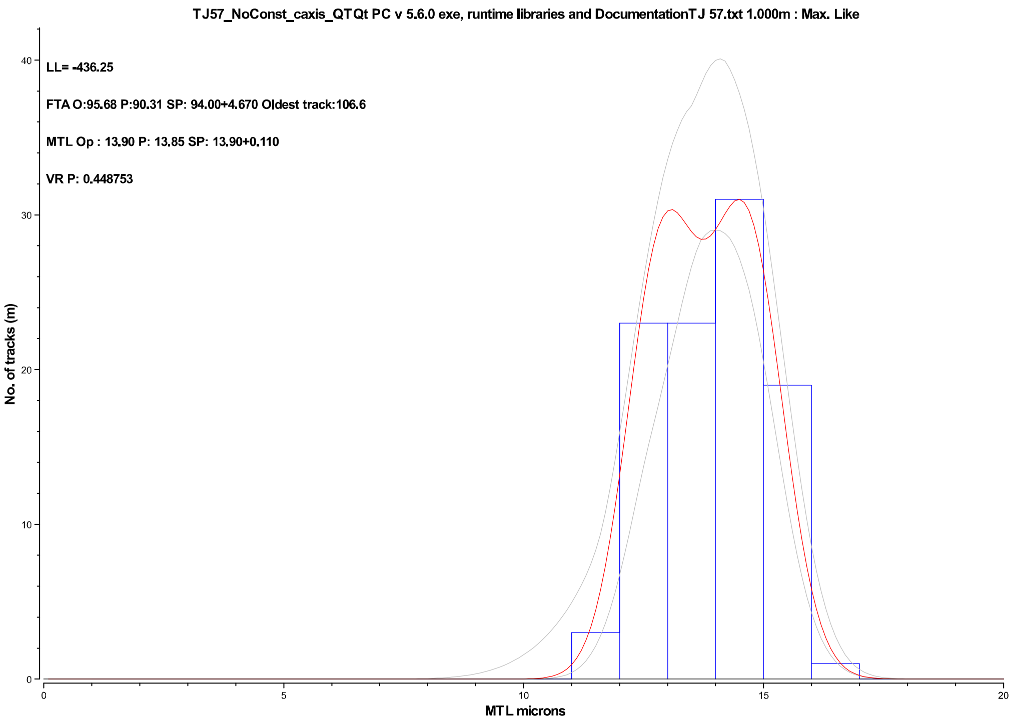
TJ57_NoConst_caxis_QTQt PC v 5.6.0 exe, runtime libraries and DocumentationTJ 57.txt 1.000m : Max. Like
LL= -436.25
FTA O:95.68 P:90.31 SP: 94.00+4.670 Oldest track:106.6
MTL Op : 13.90 P: 13.85 SP: 13.90+0.110
VR P: 0.448753
No. of tracks (m)
MTL microns

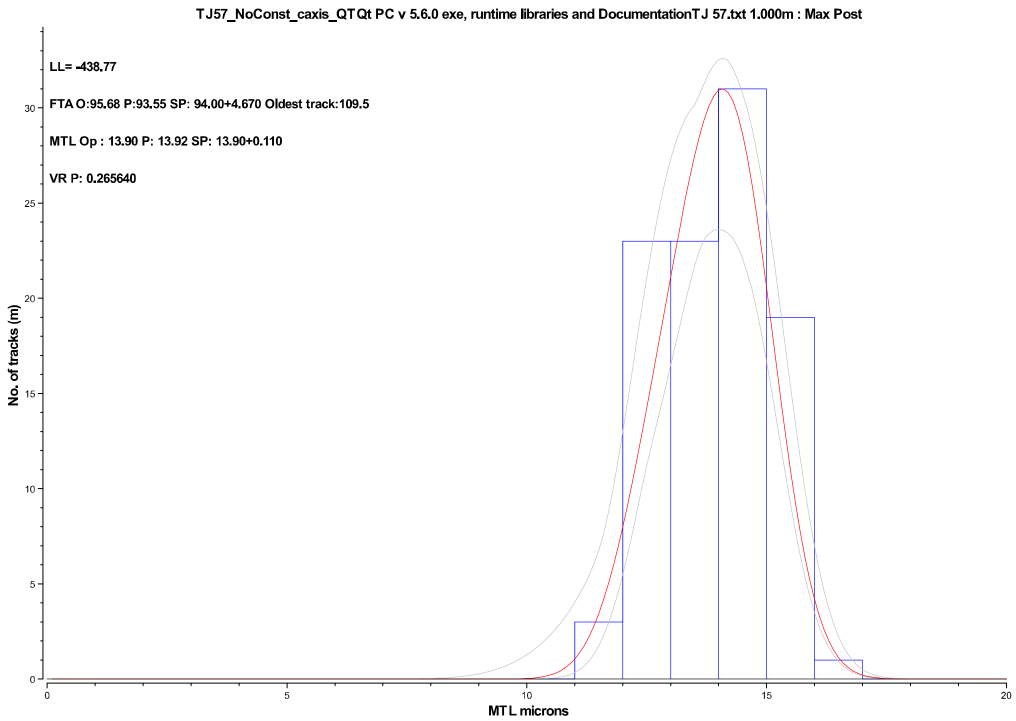
TJ57_NoConst_caxis_QTQt PC v 5.6.0 exe, runtime libraries and DocumentationTJ 57.txt 1.000m : Max Post
LL= -438.77
FTA O:95.68 P:93.55 SP: 94.00+4.670 Oldest track:109.5
MTL Op : 13.90 P: 13.92 SP: 13.90+0.110
VR P: 0.265640
No. of tracks (m)
MTL microns

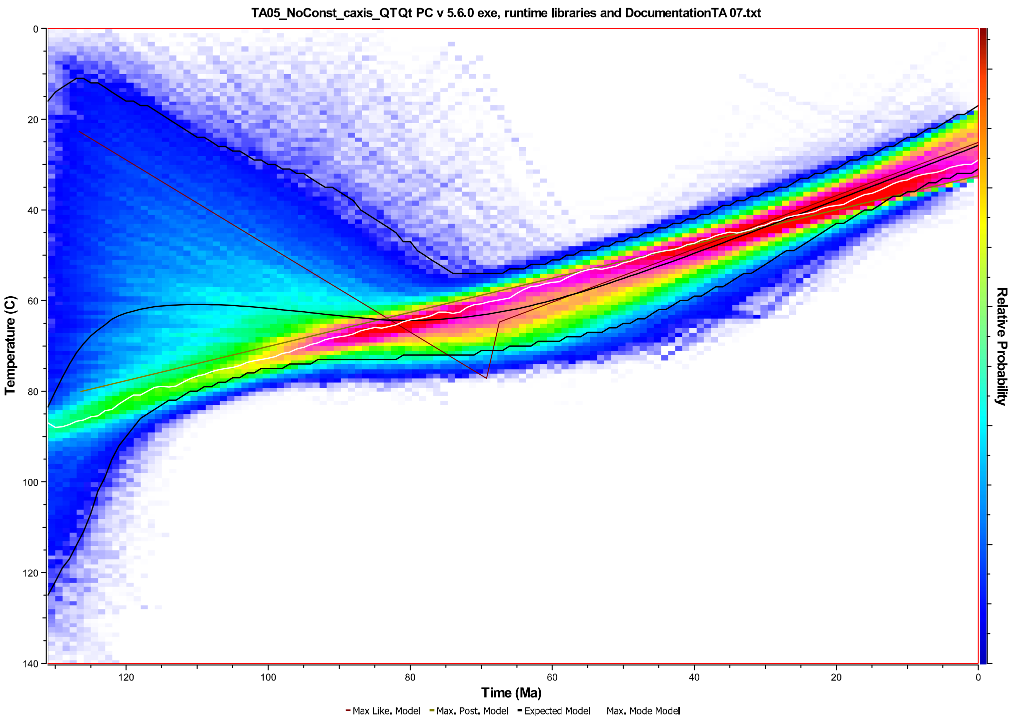
TA05_NoConst_caxis_QTQt PC v 5.6.0 exe, runtime libraries and DocumentationTA 07.txt
Temperature (C)
Time (Ma)
Relative Probability
Max Like. Model
Max. Post. Model
Expected Model
Max. Mode Model

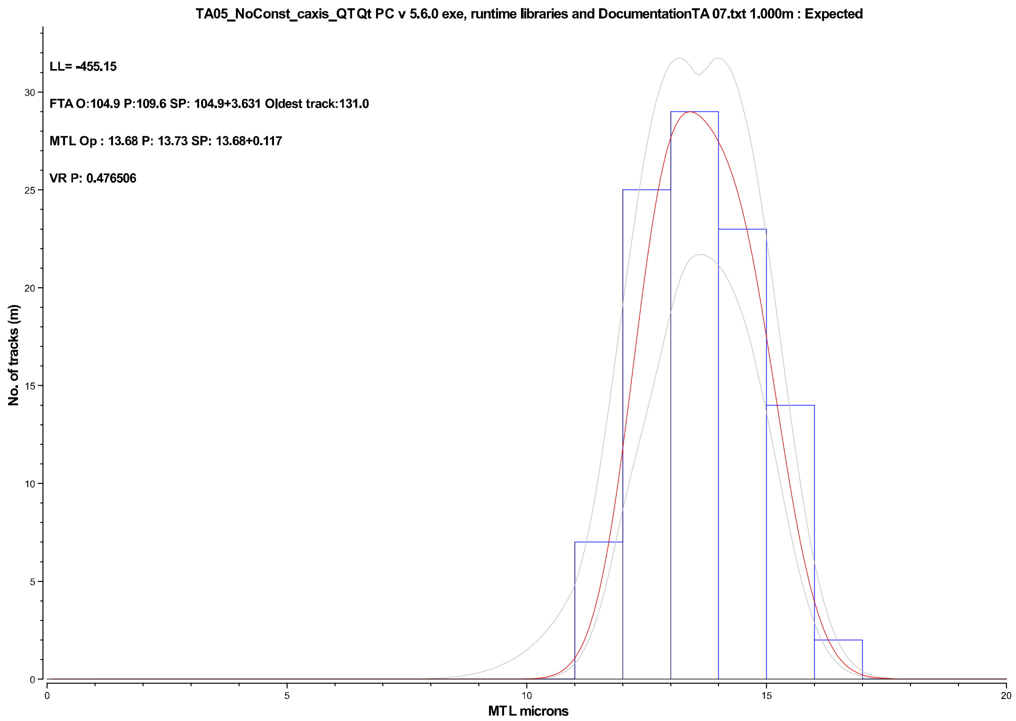
TA05_NoConst_caxis_QTQt PC v 5.6.0 exe, runtime libraries and DocumentationTA 07.txt 1.000m : Expected
LL= -455.15
FTA O:104.9 P:109.6 SP: 104.9+3.631 Oldest track:131.0
MTL Op : 13.68 P: 13.73 SP: 13.68+0.117
VR P: 0.476506
No. of tracks (m)
MTL microns

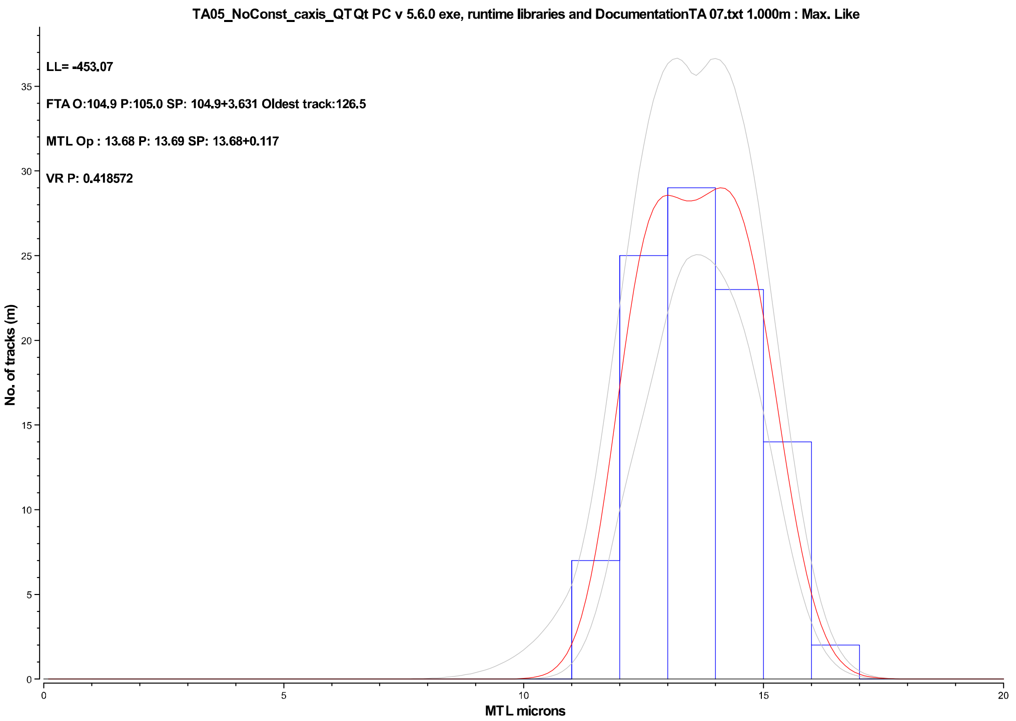
TA05_NoConst_caxis_QTQt PC v 5.6.0 exe, runtime libraries and DocumentationTA 07.txt 1.000m : Max. Like
LL= -453.07
FTA O:104.9 P:105.0 SP: 104.9+3.631 Oldest track:126.5
MTL Op : 13.68 P: 13.69 SP: 13.68+0.117
VR P: 0.418572
No. of tracks (m)
MTL microns

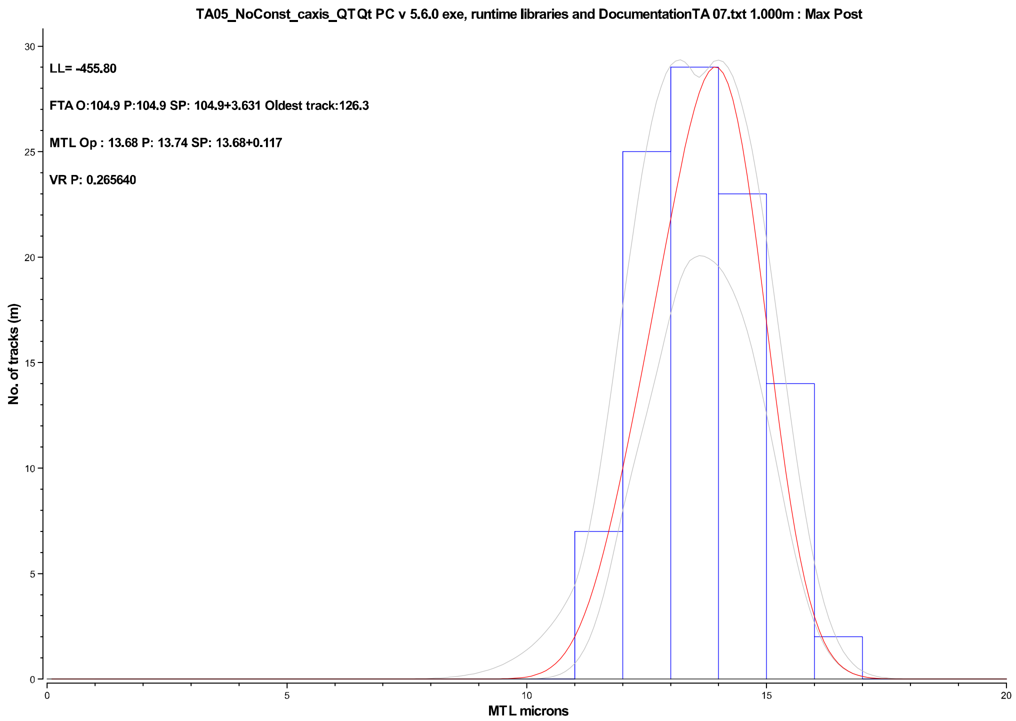
TA05_NoConst_caxis_QTQt PC v 5.6.0 exe, runtime libraries and DocumentationTA 07.txt 1.000m : Max Post
LL= -455.80
FTA O:104.9 P:104.9 SP: 104.9+3.631 Oldest track:126.3
MTL Op : 13.68 P: 13.74 SP: 13.68+0.117
VR P: 0.265640
No. of tracks (m)
MTL microns

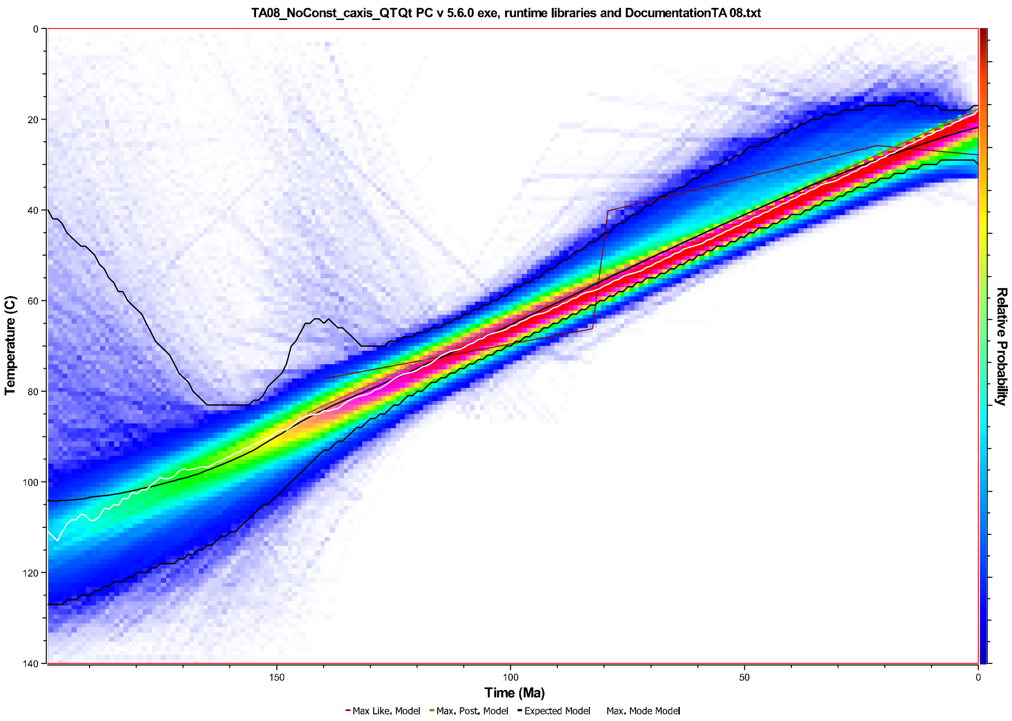
TA08_NoConst_caxis_QTQt PC v 5.6.0 exe, runtime libraries and DocumentationTA 08.txt
Temperature (C)
Time (Ma)
Relative Probability
Max Like. Model
Max. Post. Model
Expected Model
Max. Mode Model

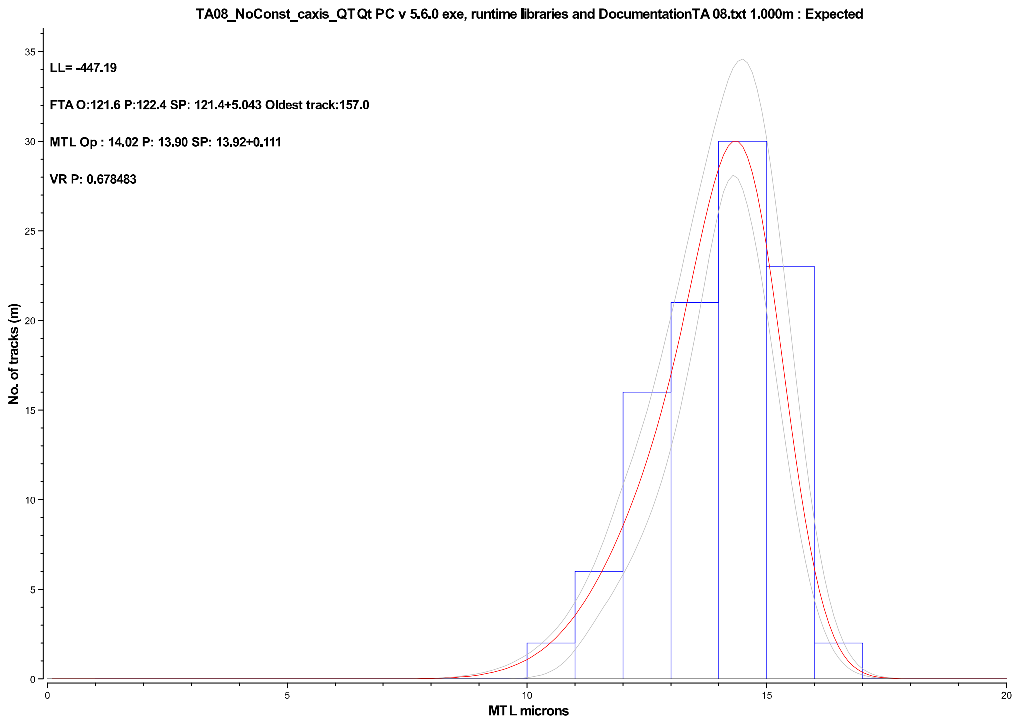
TA08_NoConst_caxis_QTQt PC v 5.6.0 exe, runtime libraries and DocumentationTA 08.txt 1.000m : Expected
LL= -447.19
FTA O:121.6 P:122.4 SP: 121.4+5.043 Oldest track:157.0
MTL Op : 14.02 P: 13.90 SP: 13.92+0.111
VR P: 0.678483
No. of tracks (m)
MTL microns

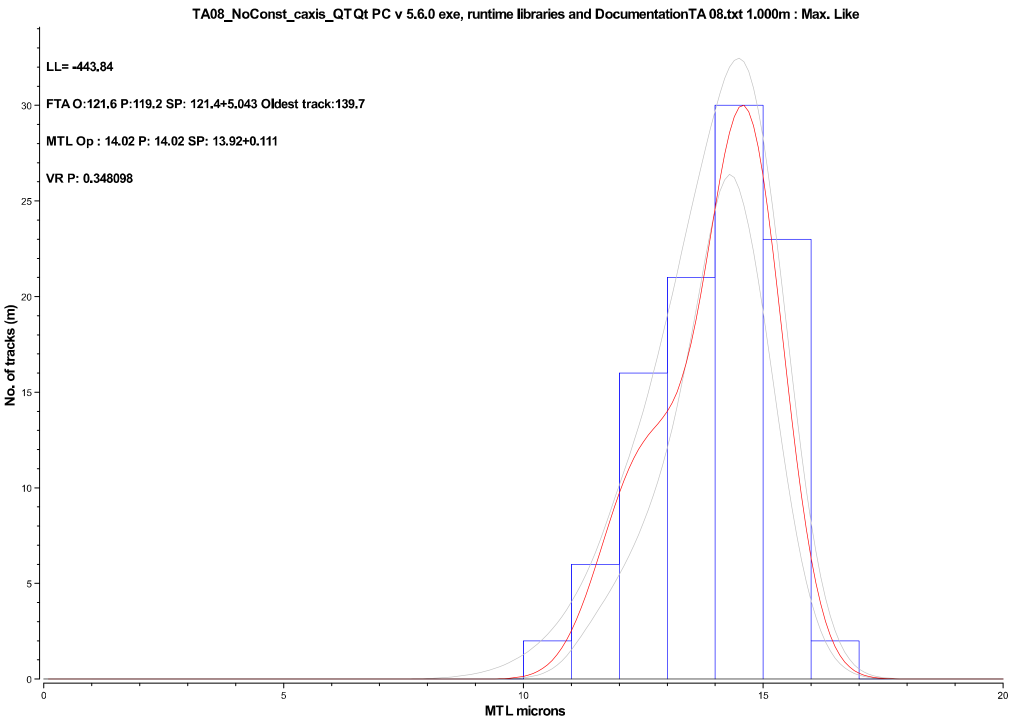
TA08_NoConst_caxis_QTQt PC v 5.6.0 exe, runtime libraries and DocumentationTA 08.txt 1.000m : Max. Like
LL= -443.84
FTA O:121.6 P:119.2 SP: 121.4+5.043 Oldest track:139.7
MTL Op : 14.02 P: 14.02 SP: 13.92+0.111
VR P: 0.348098
No. of tracks (m)
MTL microns

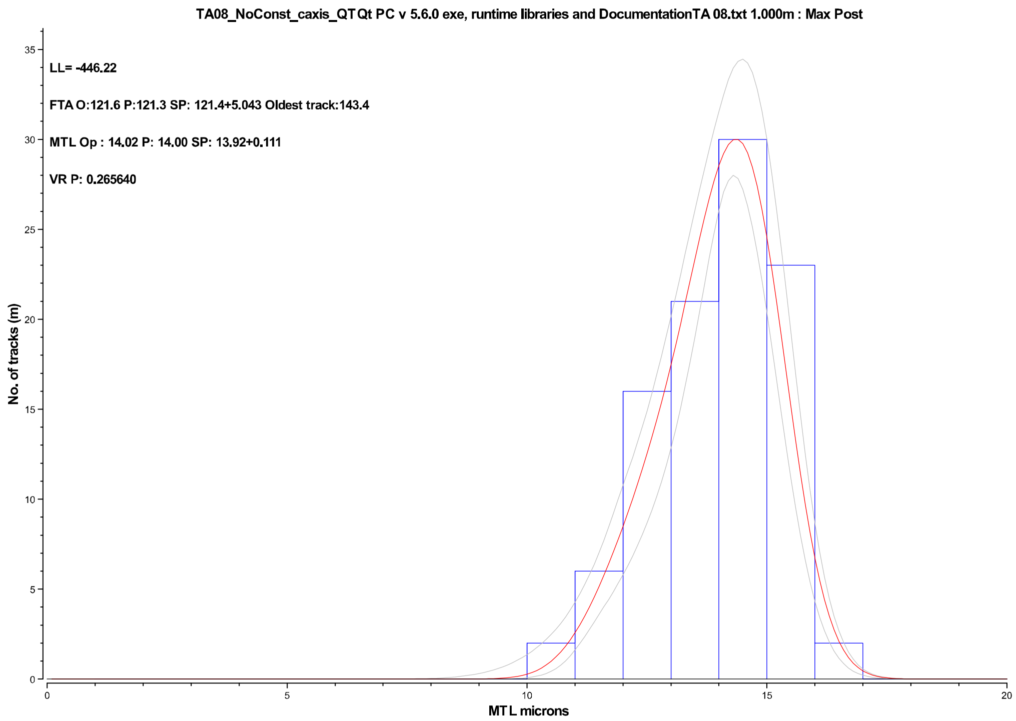
TA08_NoConst_caxis_QTQt PC v 5.6.0 exe, runtime libraries and DocumentationTA 08.txt 1.000m : Max Post
LL= -446.22
FTA O:121.6 P:121.3 SP: 121.4+5.043 Oldest track:143.4
MTL Op : 14.02 P: 14.00 SP: 13.92+0.111
VR P: 0.265640
No. of tracks (m)
MTL microns

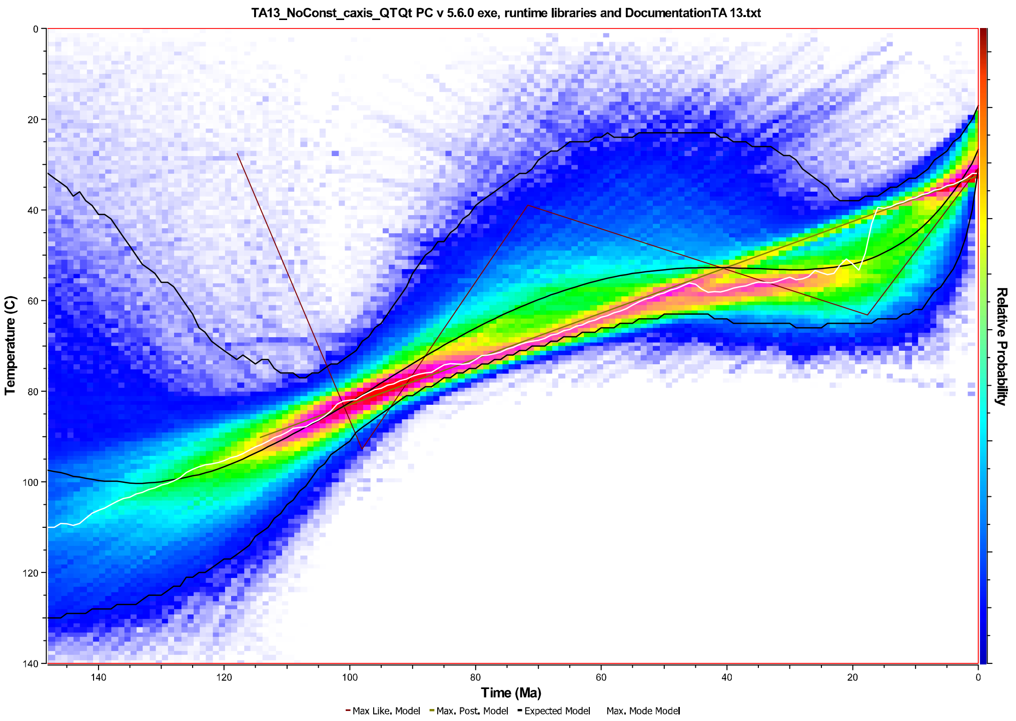
TA13_NoConst_caxis_QTQt PC v 5.6.0 exe, runtime libraries and DocumentationTA 13.txt
Temperature (C)
Time (Ma)
Relative Probability
Max Like. Model
Max. Post. Model
Expected Model
Max. Mode Model

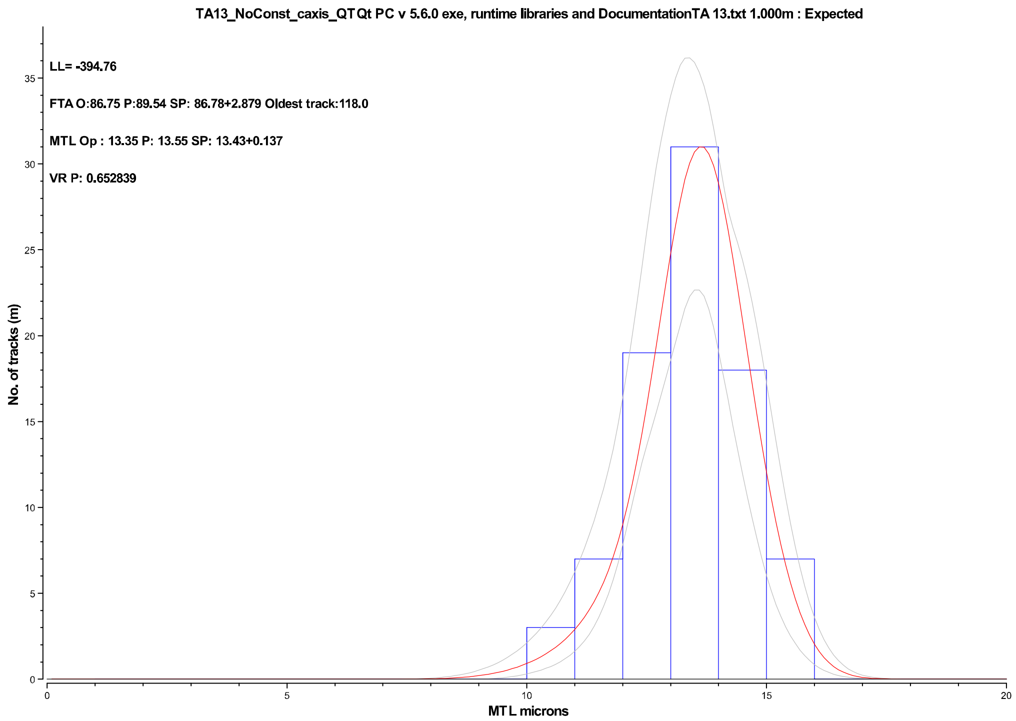
TA13_NoConst_caxis_QTQt PC v 5.6.0 exe, runtime libraries and DocumentationTA 13.txt 1.000m : Expected
LL= -394.76
FTA O:86.75 P:89.54 SP: 86.78+2.879 Oldest track:118.0
MTL Op : 13.35 P: 13.55 SP: 13.43+0.137
VR P: 0.652839
No. of tracks (m)
MTL microns

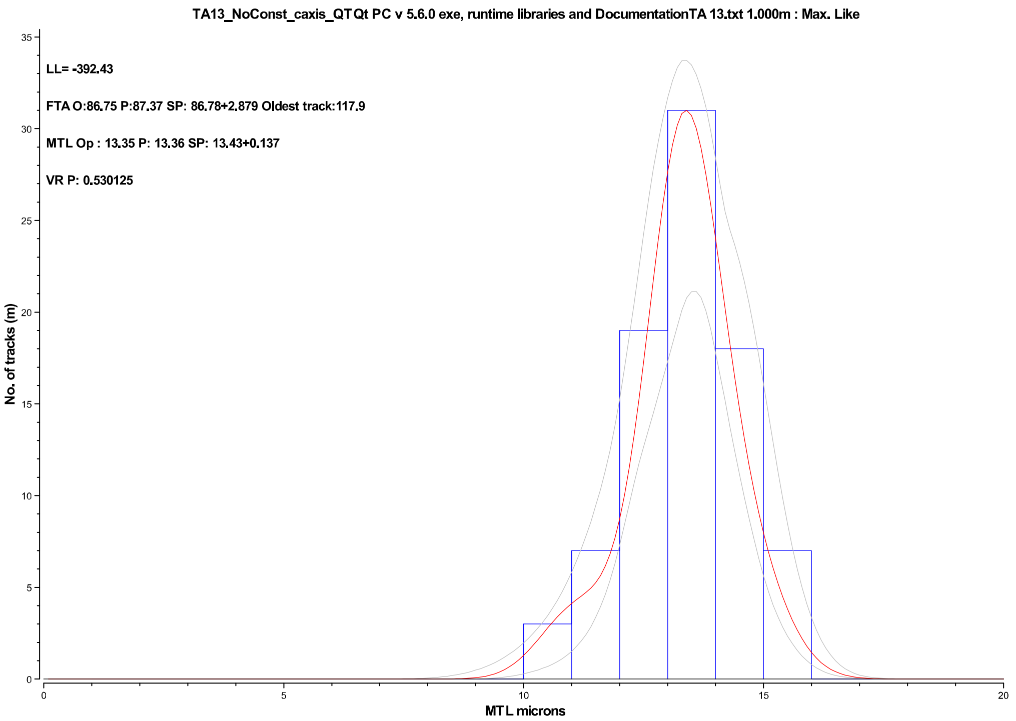
TA13_NoConst_caxis_QTQt PC v 5.6.0 exe, runtime libraries and DocumentationTA 13.txt 1.000m : Max. Like
LL= -392.43
FTA O:86.75 P:87.37 SP: 86.78+2.879 Oldest track:117.9
MTL Op : 13.35 P: 13.36 SP: 13.43+0.137
VR P: 0.530125
No. of tracks (m)
MTL microns

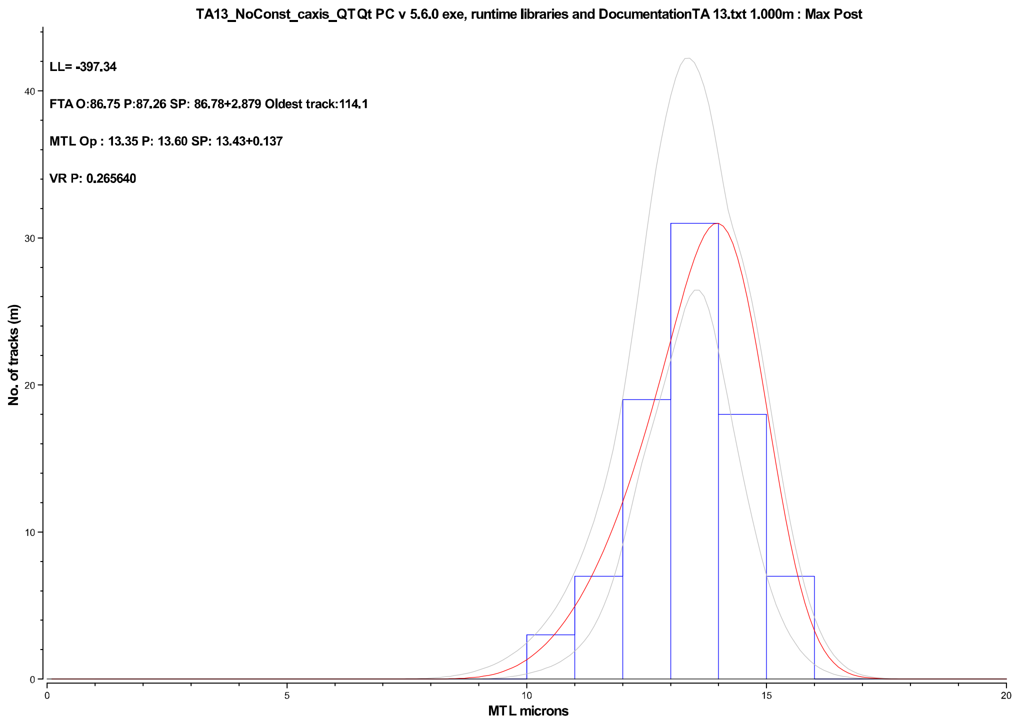
TA13_NoConst_caxis_QTQt PC v 5.6.0 exe, runtime libraries and DocumentationTA 13.txt 1.000m : Max Post
LL= -397.34
FTA O:86.75 P:87.26 SP: 86.78+2.879 Oldest track:114.1
MTL Op : 13.35 P: 13.60 SP: 13.43+0.137
VR P: 0.265640
No. of tracks (m)
MTL microns

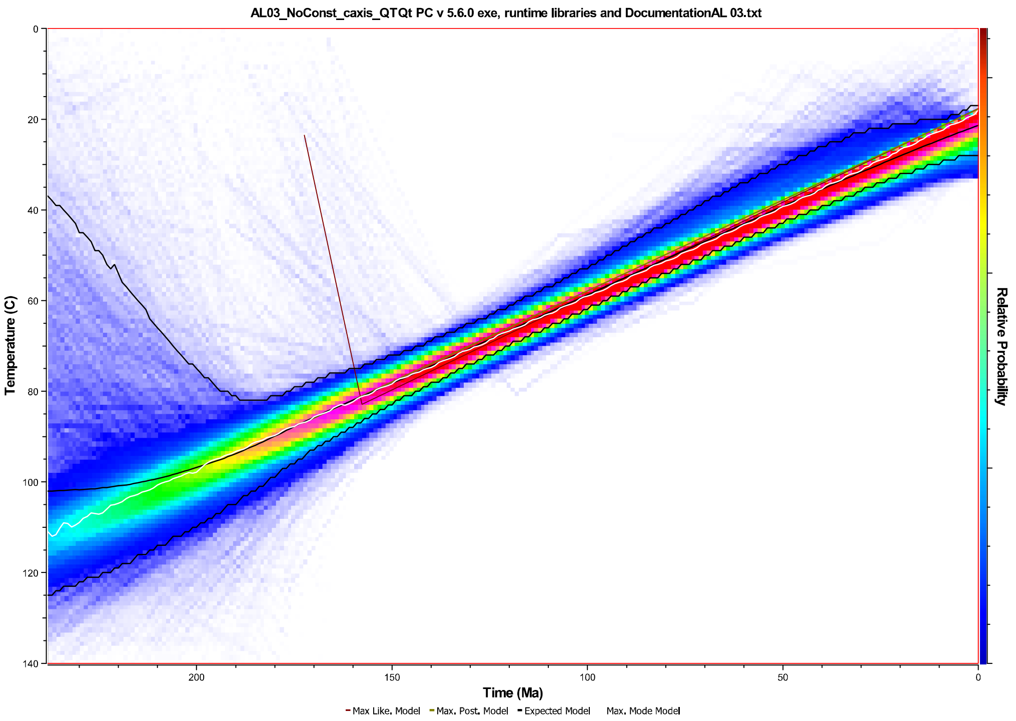
AL03_NoConst_caxis_QTQt PC v 5.6.0 exe, runtime libraries and DocumentationAL 03.txt
Temperature (C)
Time (Ma)
Relative Probability
Max Like. Model
Max. Post. Model
Expected Model
Max. Mode Model

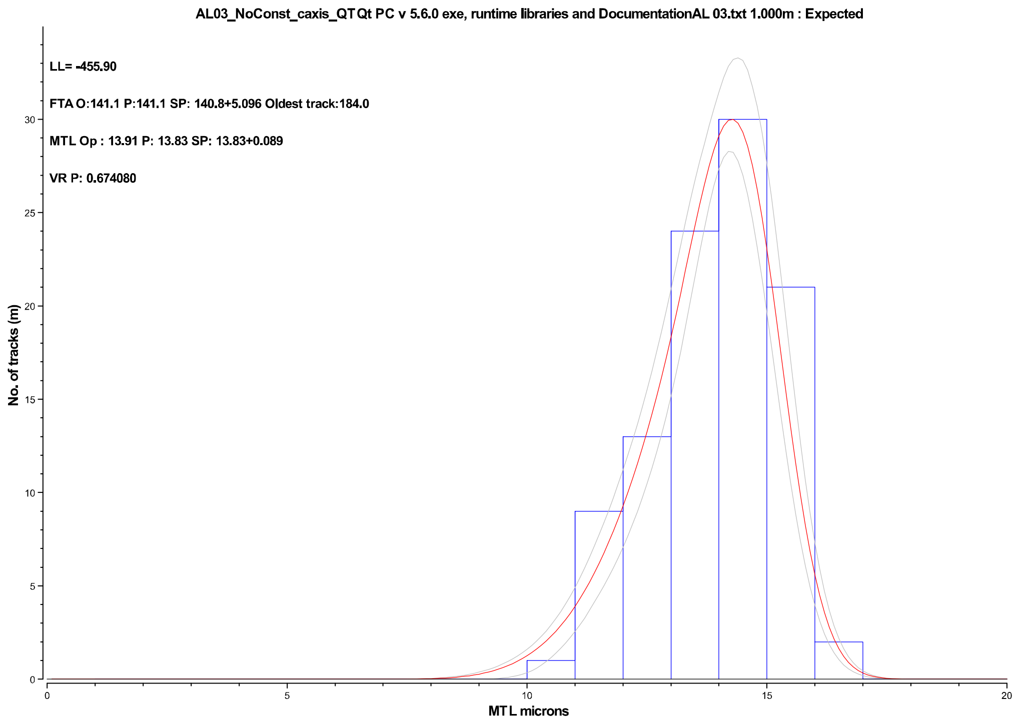
AL03_NoConst_caxis_QTQt PC v 5.6.0 exe, runtime libraries and DocumentationAL 03.txt 1.000m : Expected
LL= -455.90
FTA O:141.1 P:141.1 SP: 140.8+5.096 Oldest track:184.0
MTL Op : 13.91 P: 13.83 SP: 13.83+0.089
VR P: 0.674080
No. of tracks (m)
MTL microns

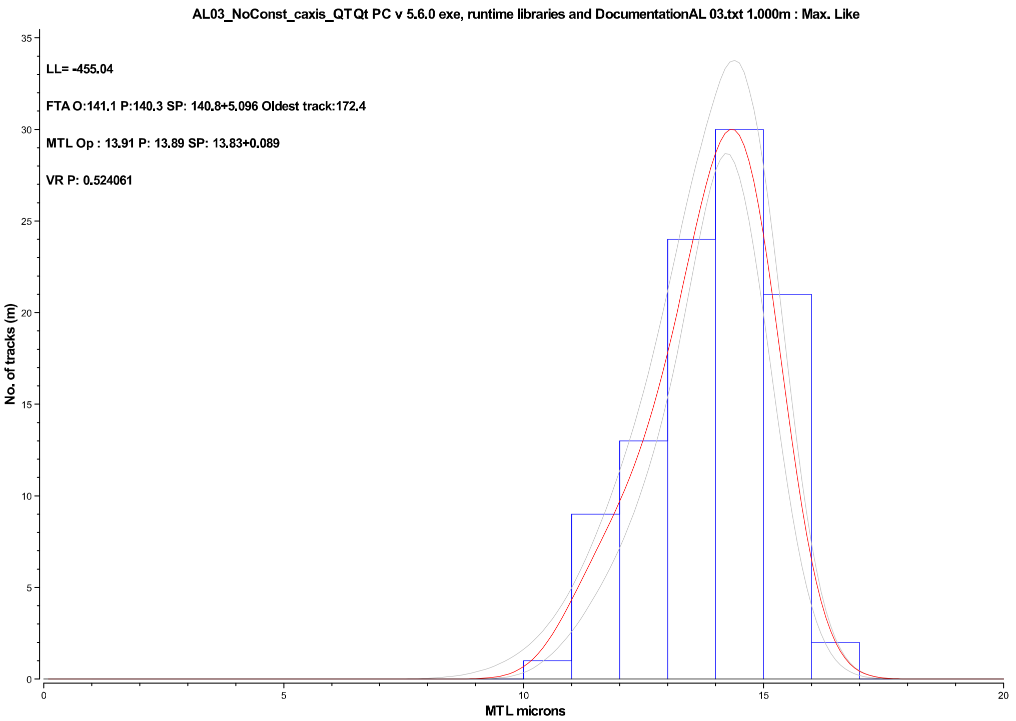
AL03_NoConst_caxis_QTQt PC v 5.6.0 exe, runtime libraries and DocumentationAL 03.txt 1.000m : Max. Like
LL= -455.04
FTA O:141.1 P:140.3 SP: 140.8+5.096 Oldest track:172.4
MTL Op : 13.91 P: 13.89 SP: 13.83+0.089
VR P: 0.524061
No. of tracks (m)
MTL microns

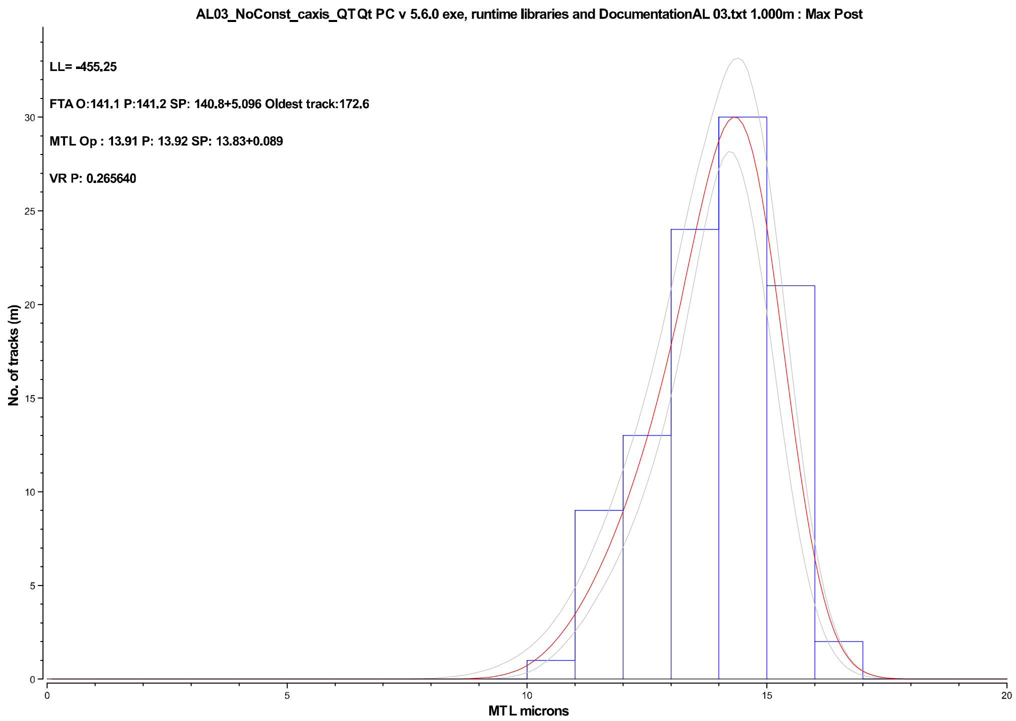
AL03_NoConst_caxis_QTQt PC v 5.6.0 exe, runtime libraries and DocumentationAL 03.txt 1.000m : Max Post
LL= -455.25
FTA O:141.1 P:141.2 SP: 140.8+5.096 Oldest track:172.6
MTL Op : 13.91 P: 13.92 SP: 13.83+0.089
VR P: 0.265640
No. of tracks (m)
MTL microns

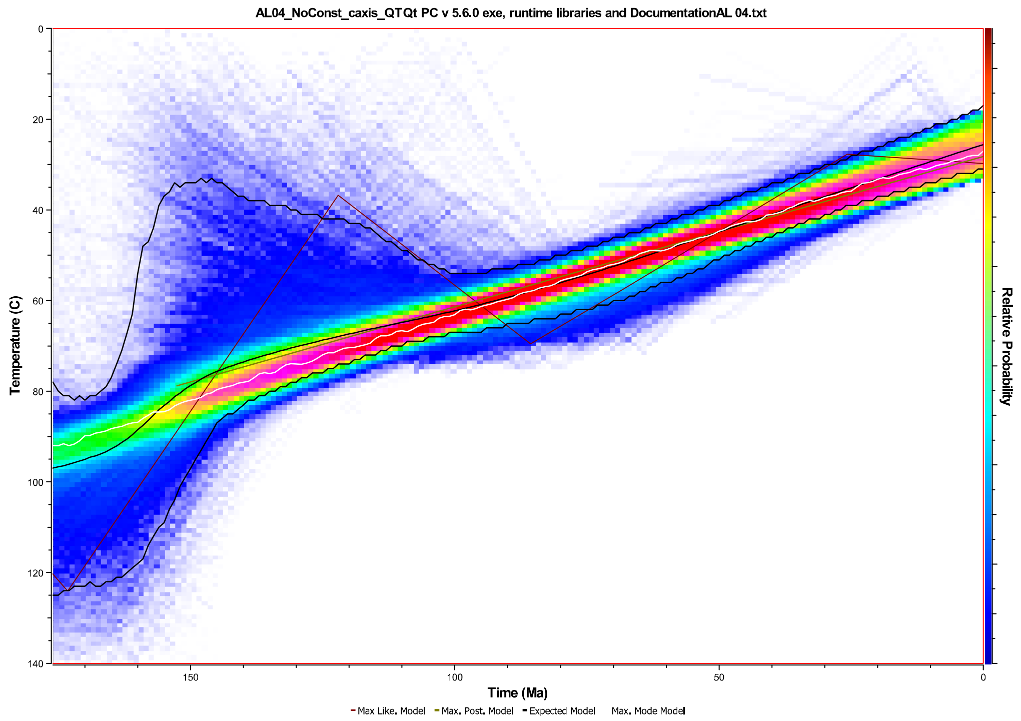
AL04_NoConst_caxis_QTQt PC v 5.6.0 exe, runtime libraries and DocumentationAL 04.txt
Temperature (C)
Time (Ma)
Relative Probability
Max Like. Model
Max. Post. Model
Expected Model
Max. Mode Model

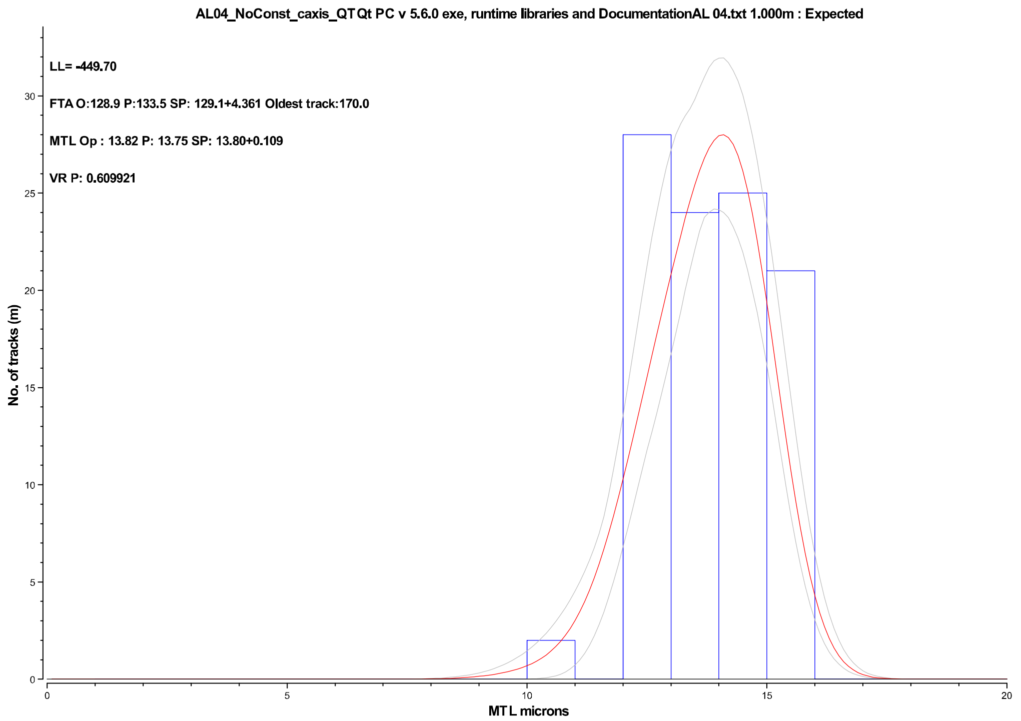
AL04_NoConst_caxis_QTQt PC v 5.6.0 exe, runtime libraries and DocumentationAL 04.txt 1.000m : Expected
LL= -449.70
FTA O:128.9 P:133.5 SP: 129.1+4.361 Oldest track:170.0
MTL Op : 13.82 P: 13.75 SP: 13.80+0.109
VR P: 0.609921
No. of tracks (m)
MTL microns

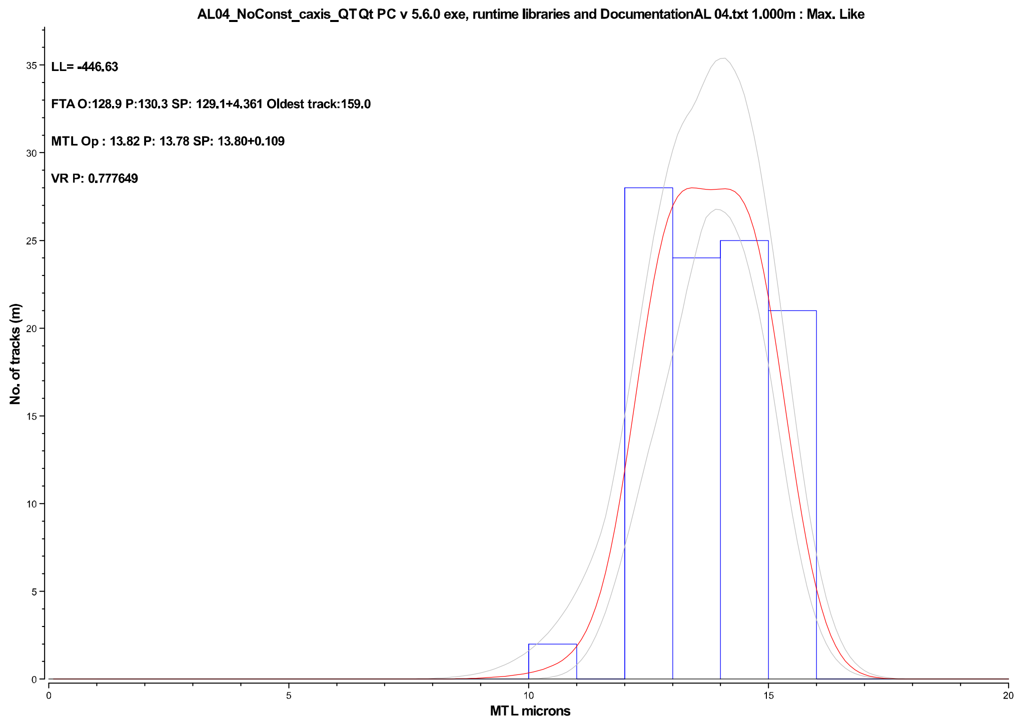
AL04_NoConst_caxis_QTQt PC v 5.6.0 exe, runtime libraries and DocumentationAL 04.txt 1.000m : Max. Like
LL= -446.63
FTA O:128.9 P:130.3 SP: 129.1+4.361 Oldest track:159.0
MTL Op : 13.82 P: 13.78 SP: 13.80+0.109
VR P: 0.777649
No. of tracks (m)
MTL microns

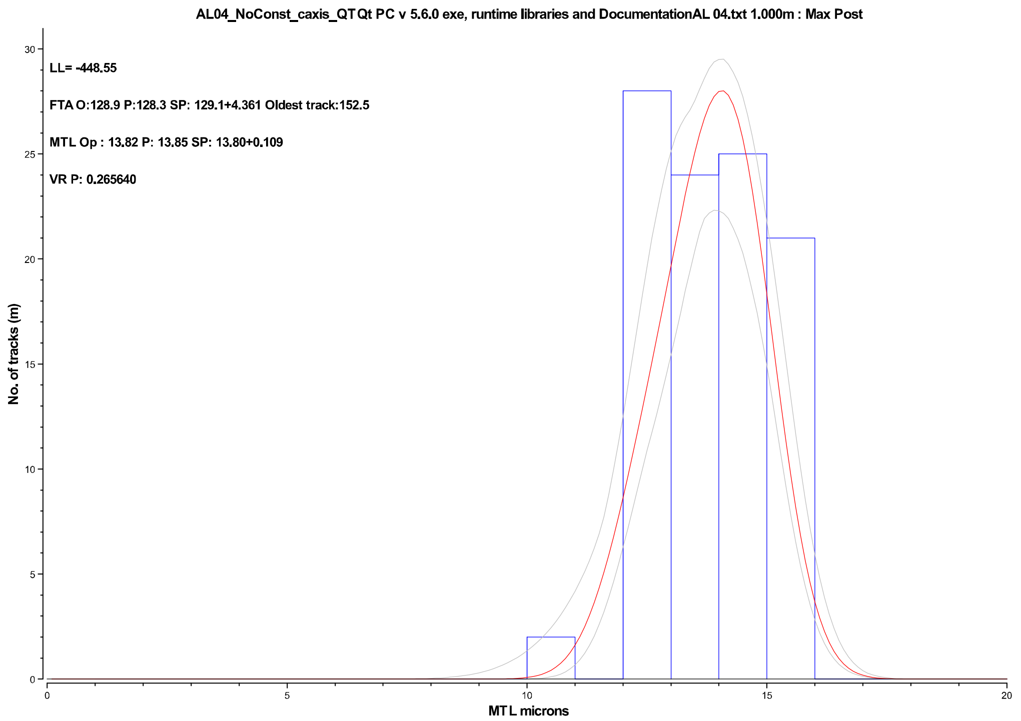
AL04_NoConst_caxis_QTQt PC v 5.6.0 exe, runtime libraries and DocumentationAL 04.txt 1.000m : Max Post
LL= -448.55
FTA O:128.9 P:128.3 SP: 129.1+4.361 Oldest track:152.5
MTL Op : 13.82 P: 13.85 SP: 13.80+0.109
VR P: 0.265640
No. of tracks (m)
MTL microns

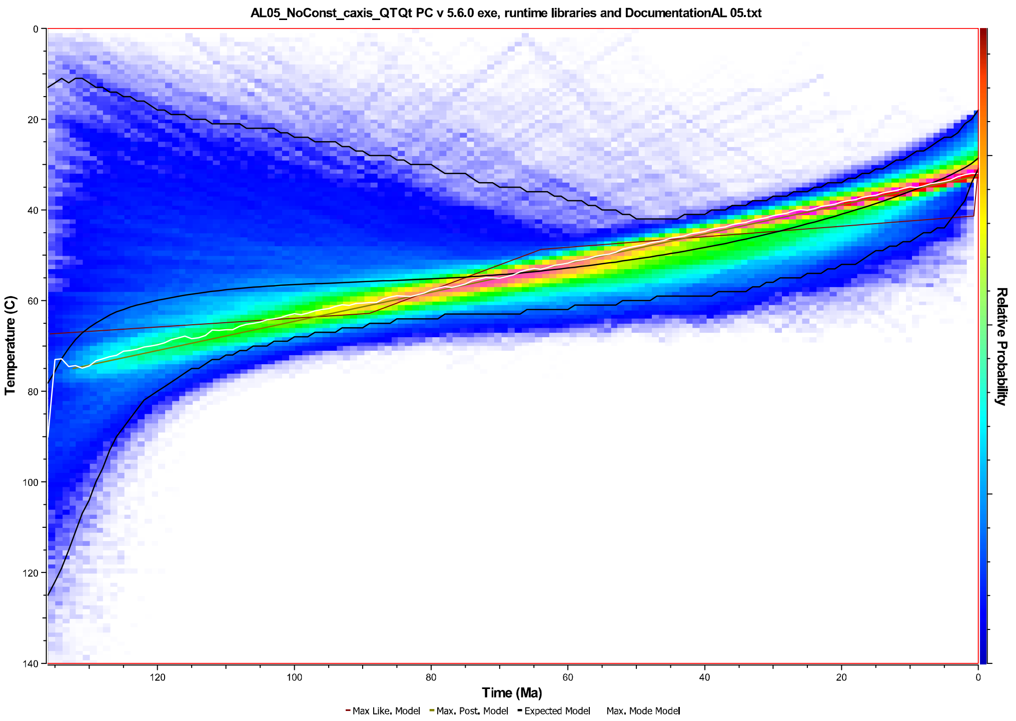
AL05_NoConst_caxis_QTQt PC v 5.6.0 exe, runtime libraries and DocumentationAL 05.txt
Temperature (C)
Time (Ma)
Relative Probability
Max Like. Model
Max. Post. Model
Expected Model
Max. Mode Model

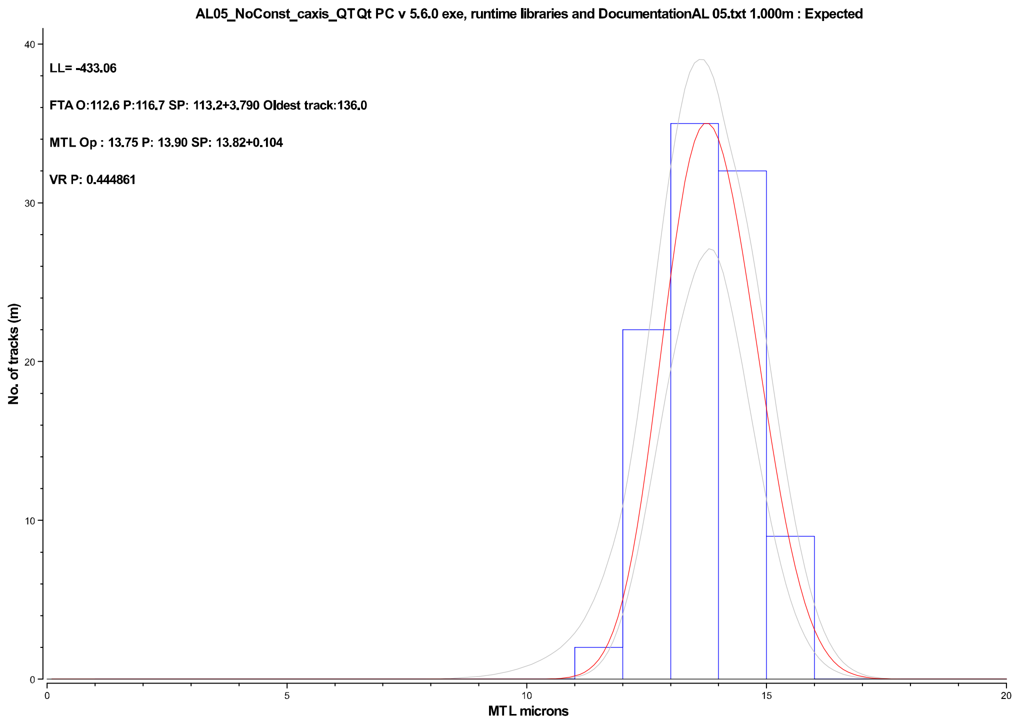
AL05_NoConst_caxis_QTQt PC v 5.6.0 exe, runtime libraries and DocumentationAL 05.txt 1.000m : Expected
LL= -433.06
FTA O:112.6 P:116.7 SP: 113.2+3.790 Oldest track:136.0
MTL Op : 13.75 P: 13.90 SP: 13.82+0.104
VR P: 0.444861
No. of tracks (m)
MTL microns

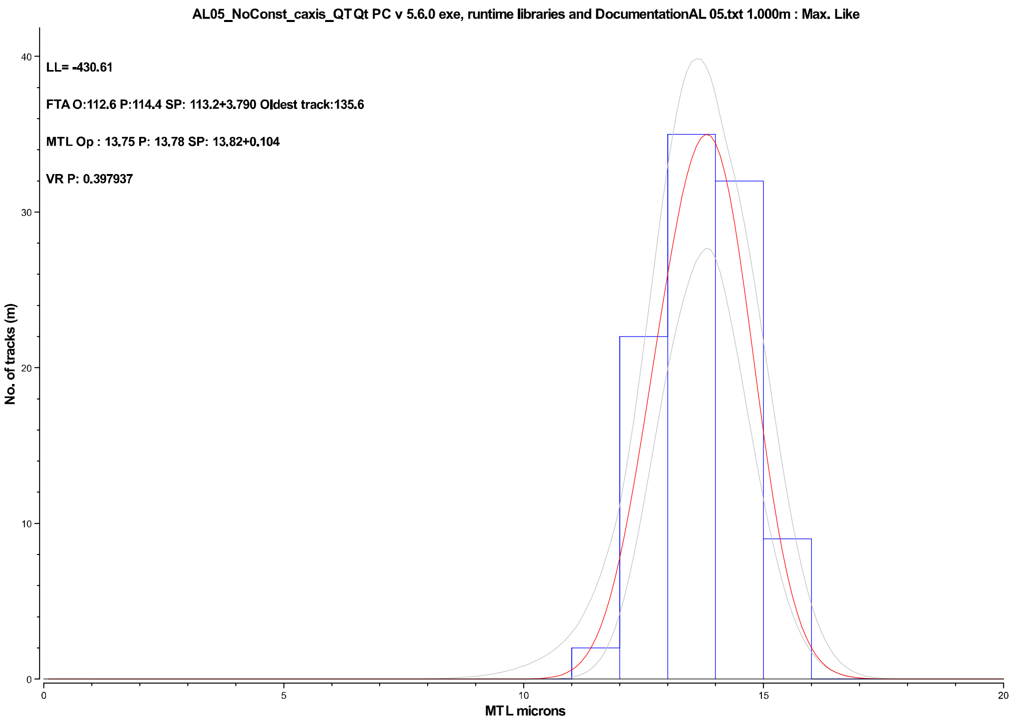
AL05_NoConst_caxis_QTQt PC v 5.6.0 exe, runtime libraries and DocumentationAL 05.txt 1.000m : Max. Like
LL= -430.61
FTA O:112.6 P:114.4 SP: 113.2+3.790 Oldest track:135.6
MTL Op : 13.75 P: 13.78 SP: 13.82+0.104
VR P: 0.397937
No. of tracks (m)
MTL microns

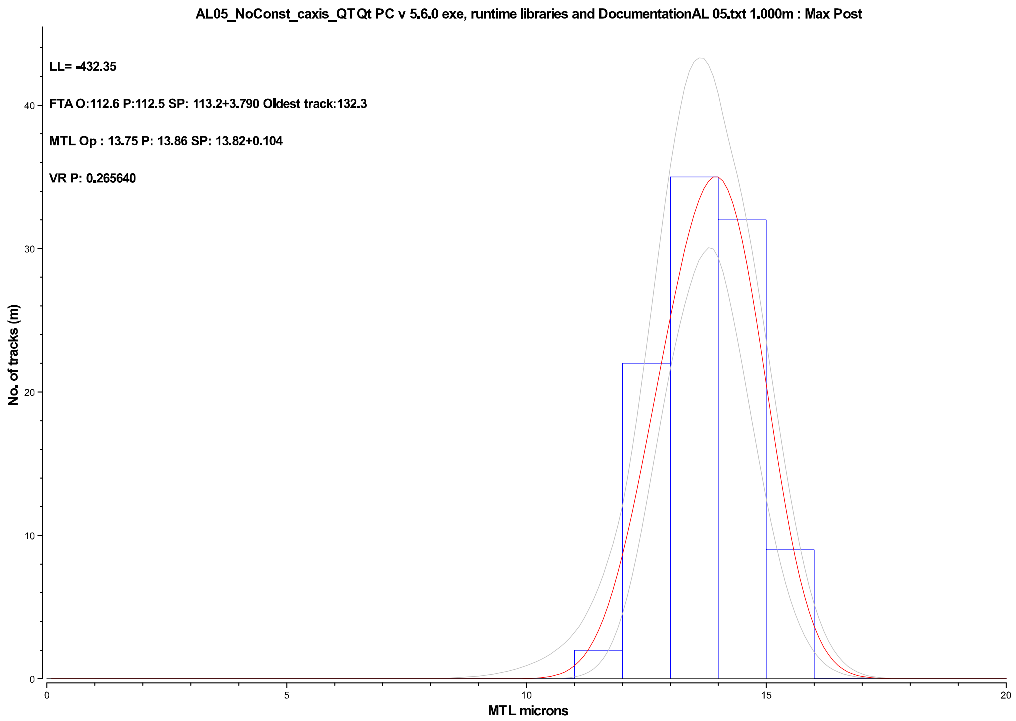
AL05_NoConst_caxis_QTQt PC v 5.6.0 exe, runtime libraries and DocumentationAL 05.txt 1.000m : Max Post
LL= -432.35
FTA O:112.6 P:112.5 SP: 113.2+3.790 Oldest track:132.3
MTL Op : 13.75 P: 13.86 SP: 13.82+0.104
VR P: 0.265640
No. of tracks (m)
MTL microns

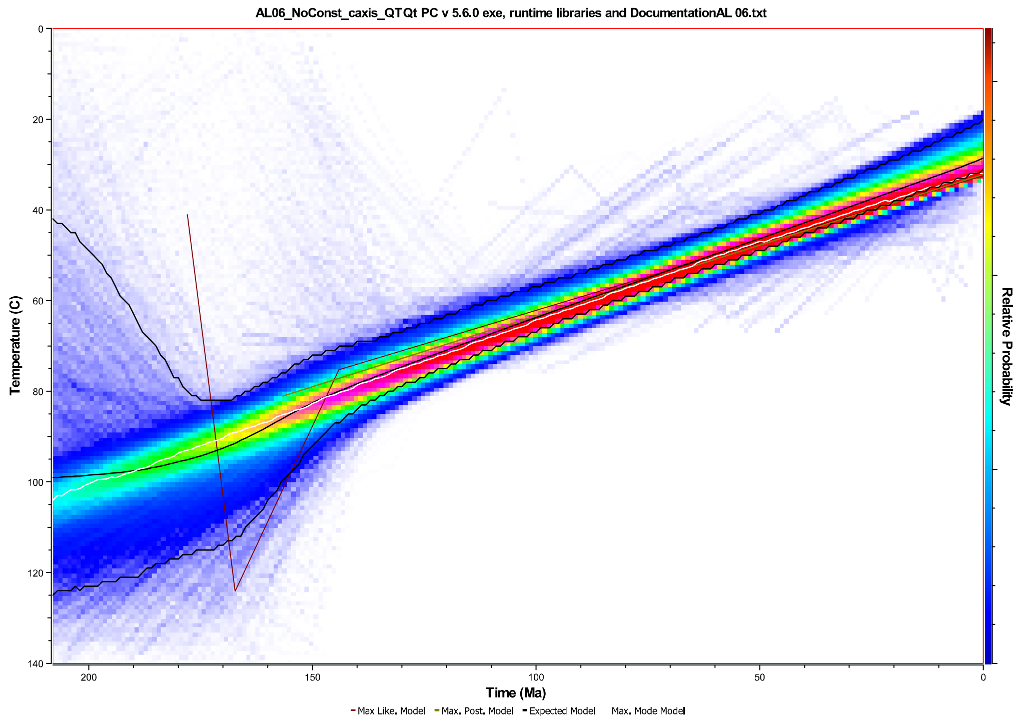
AL06_NoConst_caxis_QTQt PC v 5.6.0 exe, runtime libraries and DocumentationAL 06.txt
Temperature (C)
Time (Ma)
Relative Probability
Max Like. Model
Max. Post. Model
Expected Model
Max. Mode Model

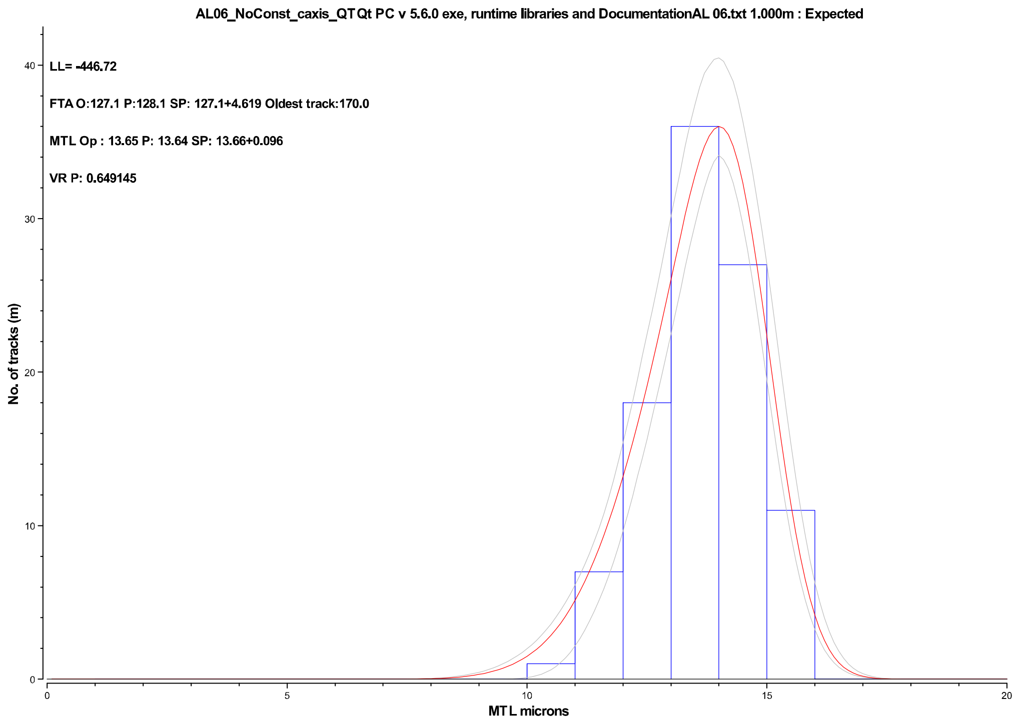
AL06_NoConst_caxis_QTQt PC v 5.6.0 exe, runtime libraries and DocumentationAL 06.txt 1.000m : Expected
LL= -446.72
FTA O:127.1 P:128.1 SP: 127.1+4.619 Oldest track:170.0
MTL Op : 13.65 P: 13.64 SP: 13.66+0.096
VR P: 0.649145
No. of tracks (m)
MTL microns

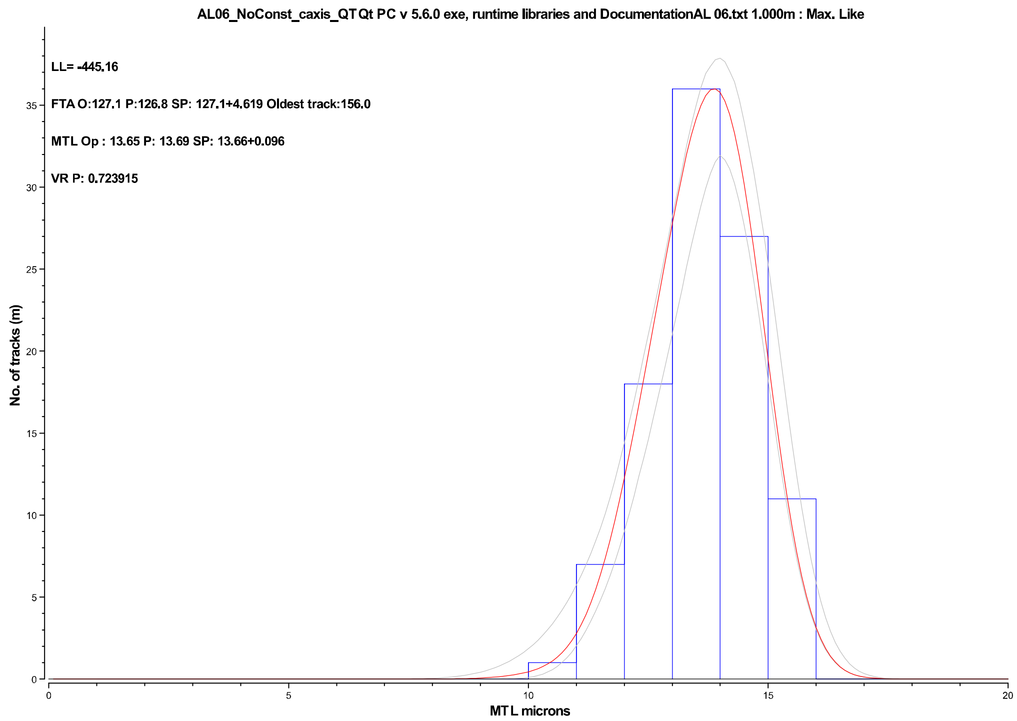
AL06_NoConst_caxis_QTQt PC v 5.6.0 exe, runtime libraries and DocumentationAL 06.txt 1.000m : Max. Like
LL= -445.16
FTA O:127.1 P:126.8 SP: 127.1+4.619 Oldest track:156.0
MTL Op : 13.65 P: 13.69 SP: 13.66+0.096
VR P: 0.723915
No. of tracks (m)
MTL microns

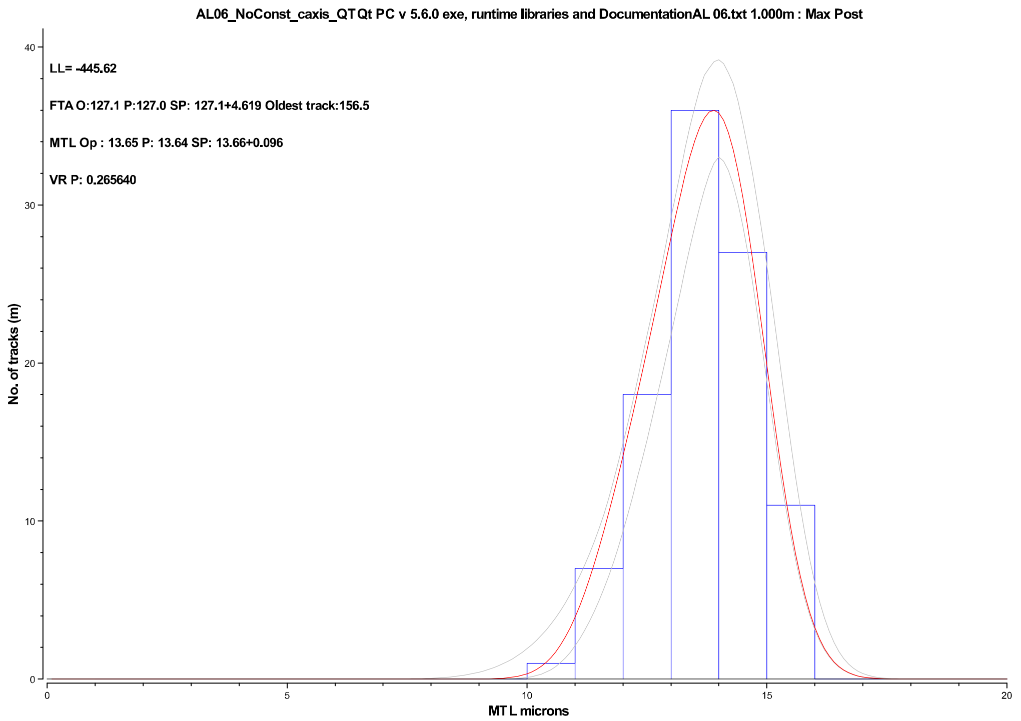
AL06_NoConst_caxis_QTQt PC v 5.6.0 exe, runtime libraries and DocumentationAL 06.txt 1.000m : Max Post
LL= -445.62
FTA O:127.1 P:127.0 SP: 127.1+4.619 Oldest track:156.5
MTL Op : 13.65 P: 13.64 SP: 13.66+0.096
VR P: 0.265640
No. of tracks (m)
MTL microns

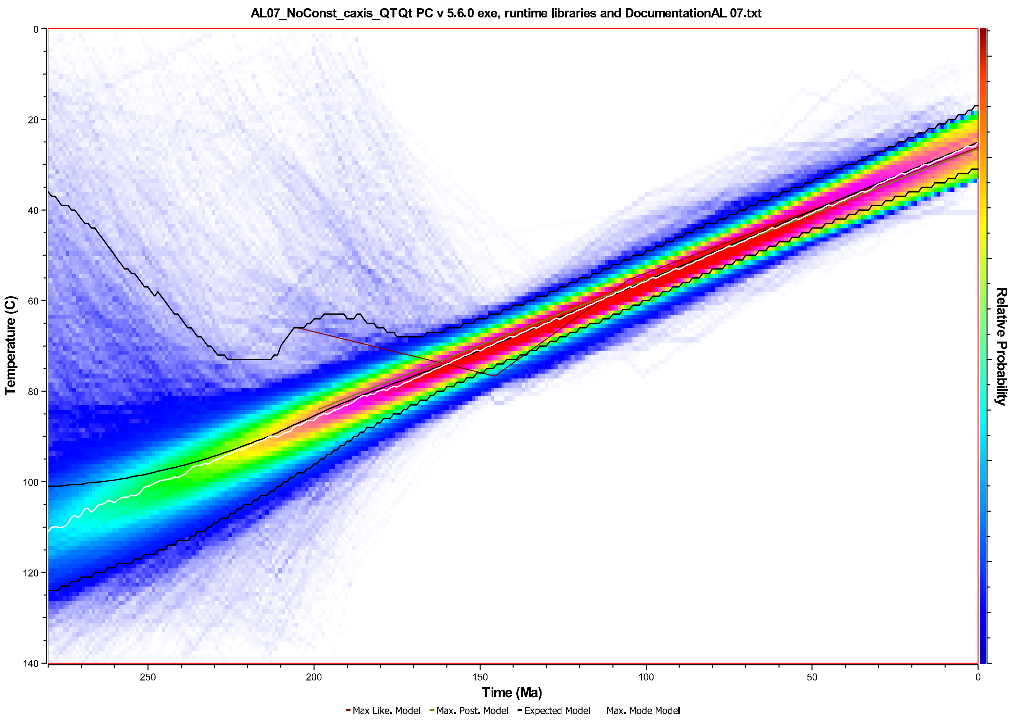
AL07_NoConst_caxis_QTQt PC v 5.6.0 exe, runtime libraries and DocumentationAL 07.txt
Temperature (C)
Time (Ma)
Relative Probability
Max Like. Model
Max. Post. Model
Expected Model
Max. Mode Model

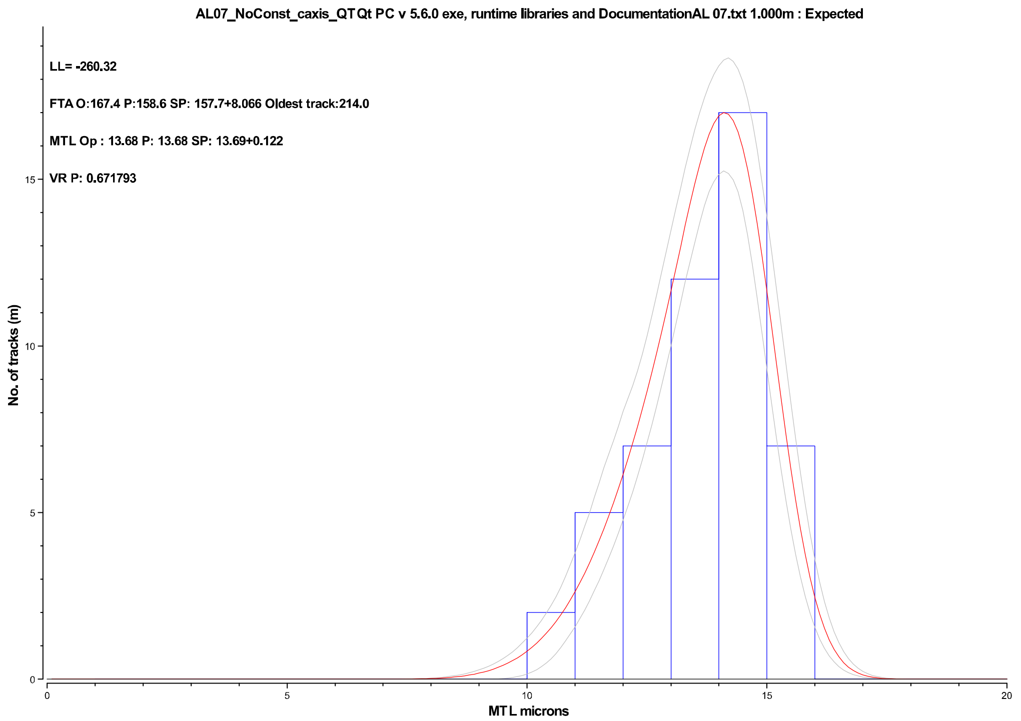
AL07_NoConst_caxis_QTQt PC v 5.6.0 exe, runtime libraries and DocumentationAL 07.txt 1.000m : Expected
LL= -260.32
FTA O:167.4 P:158.6 SP: 157.7+8.066 Oldest track:214.0
MTL Op : 13.68 P: 13.68 SP: 13.69+0.122
VR P: 0.671793
No. of tracks (m)
MTL microns

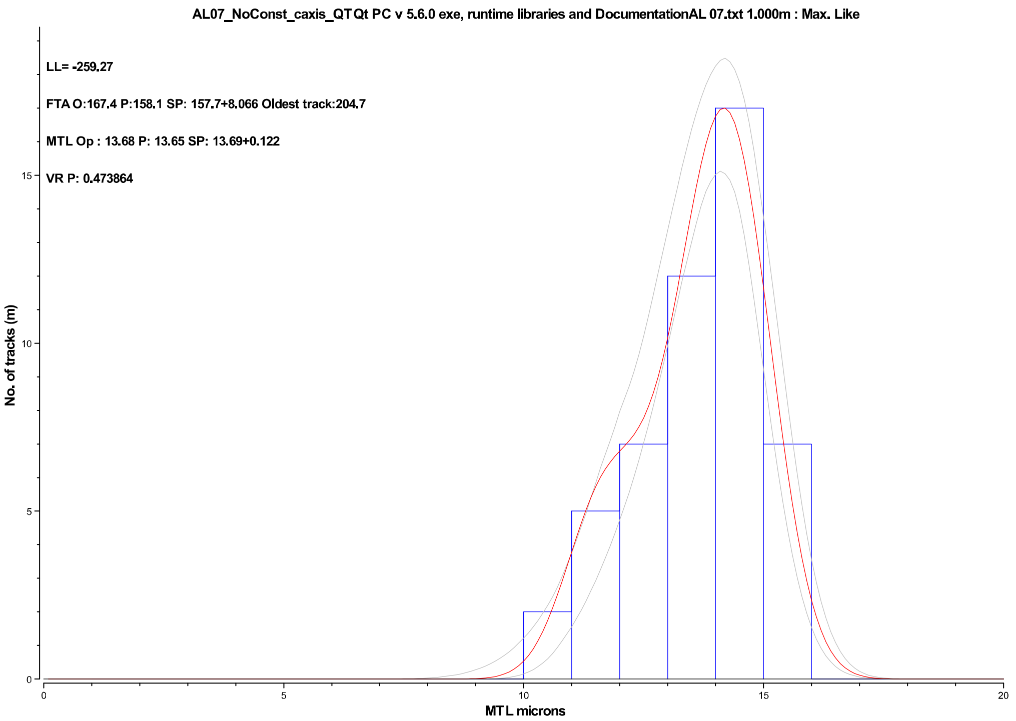
AL07_NoConst_caxis_QTQt PC v 5.6.0 exe, runtime libraries and DocumentationAL 07.txt 1.000m : Max. Like
LL= -259.27
FTA O:167.4 P:158.1 SP: 157.7+8.066 Oldest track:204.7
MTL Op : 13.68 P: 13.65 SP: 13.69+0.122
VR P: 0.473864
No. of tracks (m)
MTL microns

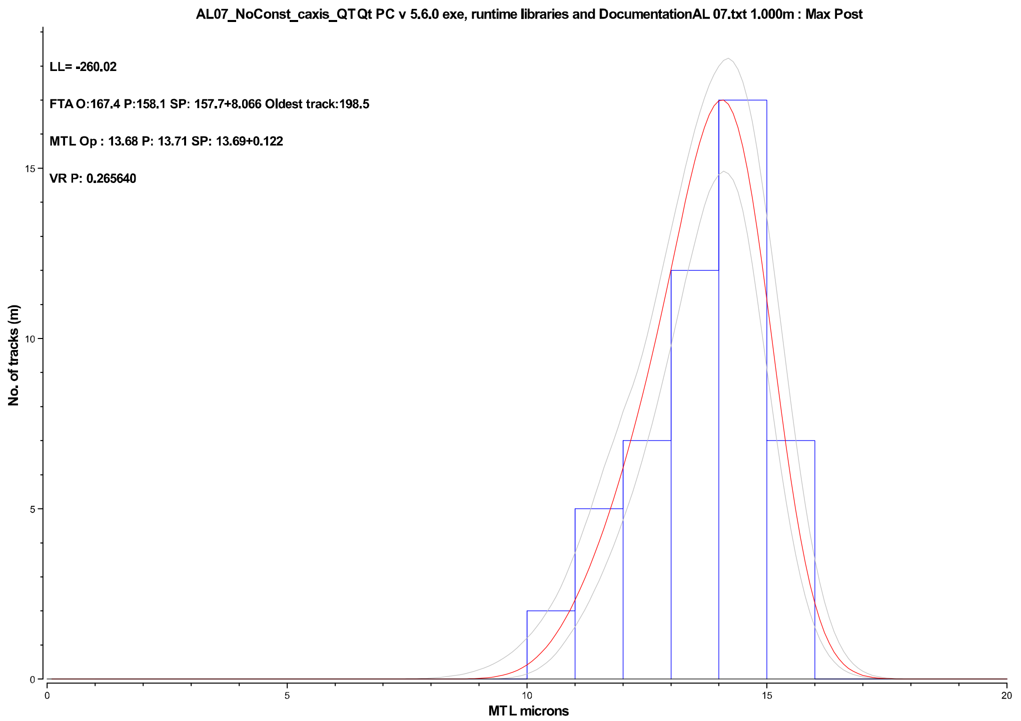
AL07_NoConst_caxis_QTQt PC v 5.6.0 exe, runtime libraries and DocumentationAL 07.txt 1.000m : Max Post
LL= -260.02
FTA O:167.4 P:158.1 SP: 157.7+8.066 Oldest track:198.5
MTL Op : 13.68 P: 13.71 SP: 13.69+0.122
VR P: 0.265640
No. of tracks (m)
MTL microns

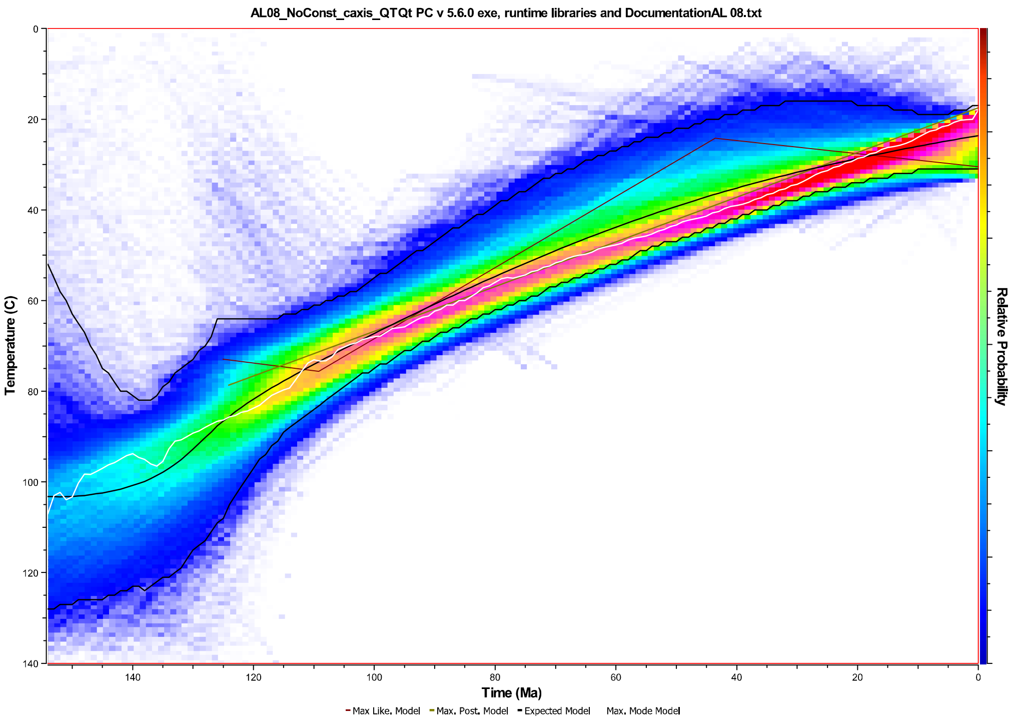
AL08_NoConst_caxis_QTQt PC v 5.6.0 exe, runtime libraries and DocumentationAL 08.txt
Temperature (C)
Time (Ma)
Relative Probability
Max Like. Model
Max. Post. Model
Expected Model
Max. Mode Model

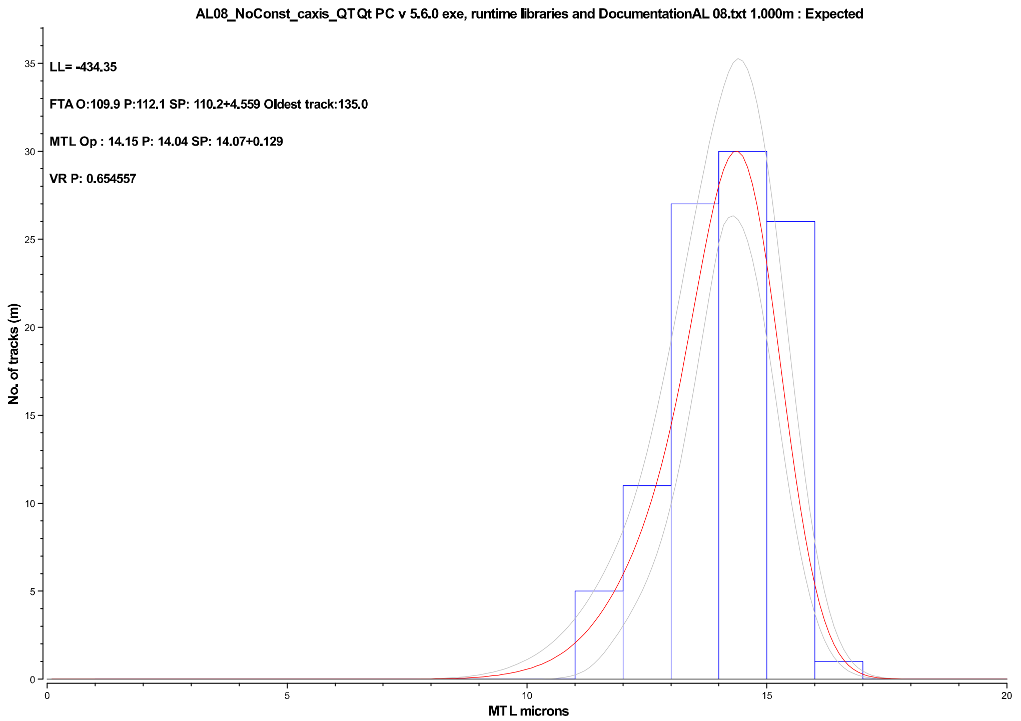
AL08_NoConst_caxis_QTQt PC v 5.6.0 exe, runtime libraries and DocumentationAL 08.txt 1.000m : Expected
LL= -434.35
FTA O:109.9 P:112.1 SP: 110.2+4.559 Oldest track:135.0
MTL Op : 14.15 P: 14.04 SP: 14.07+0.129
VR P: 0.654557
No. of tracks (m)
MTL microns

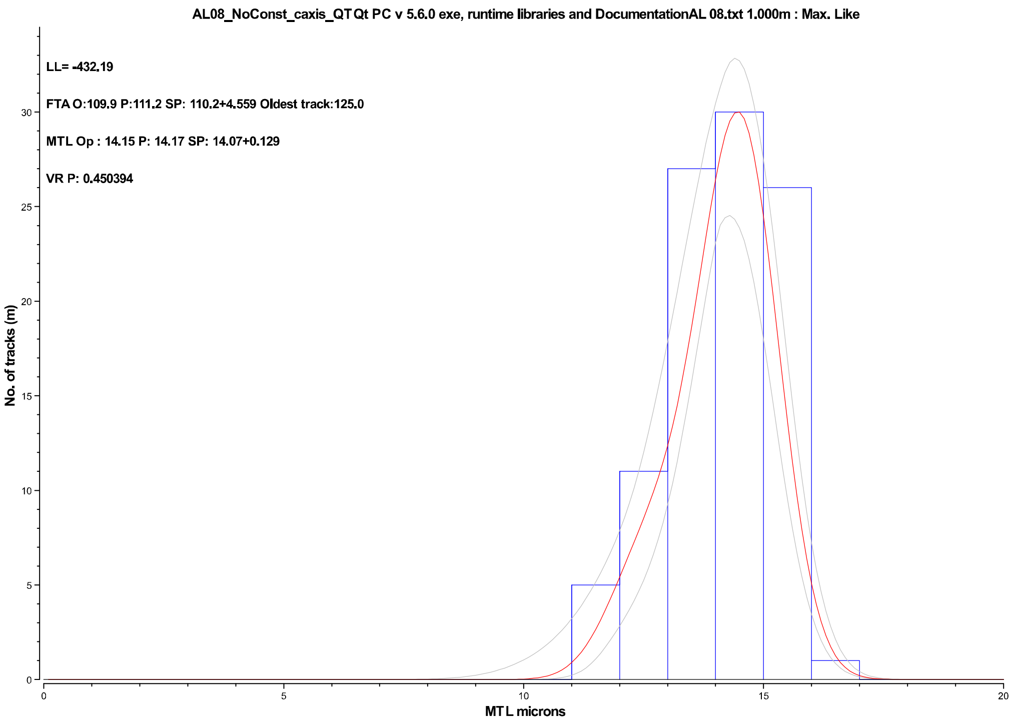
AL08_NoConst_caxis_QTQt PC v 5.6.0 exe, runtime libraries and DocumentationAL 08.txt 1.000m : Max. Like
LL= -432.19
FTA O:109.9 P:111.2 SP: 110.2+4.559 Oldest track:125.0
MTL Op : 14.15 P: 14.17 SP: 14.07+0.129
VR P: 0.450394
No. of tracks (m)
MTL microns

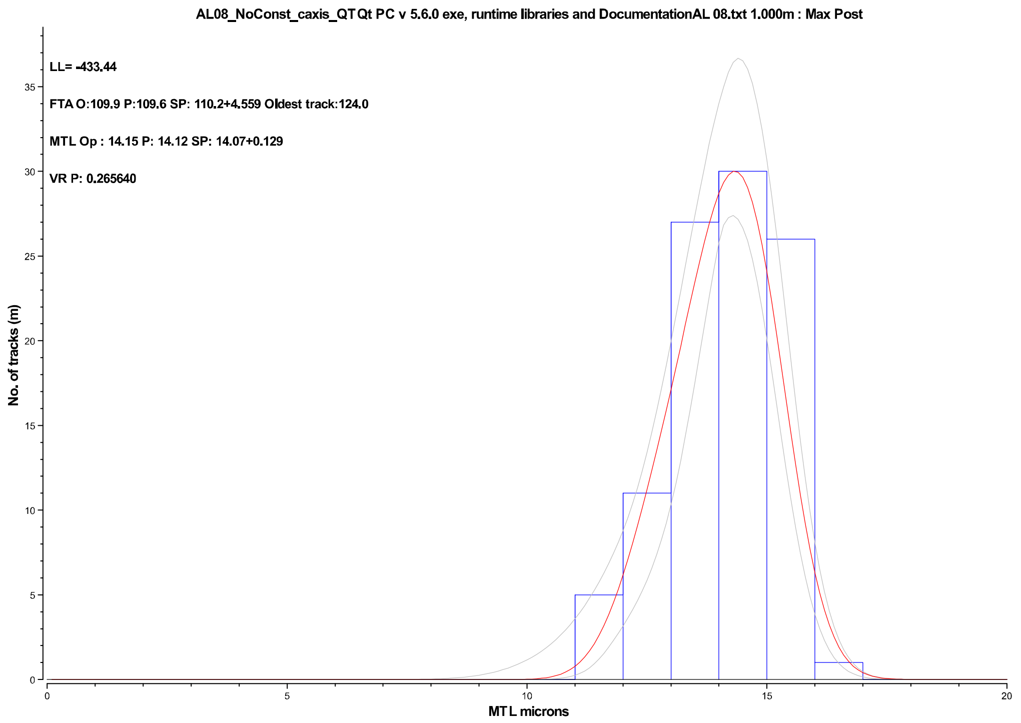
AL08_NoConst_caxis_QTQt PC v 5.6.0 exe, runtime libraries and DocumentationAL 08.txt 1.000m : Max Post
LL= -433.44
FTA O:109.9 P:109.6 SP: 110.2+4.559 Oldest track:124.0
MTL Op : 14.15 P: 14.12 SP: 14.07+0.129
VR P: 0.265640
No. of tracks (m)
MTL microns

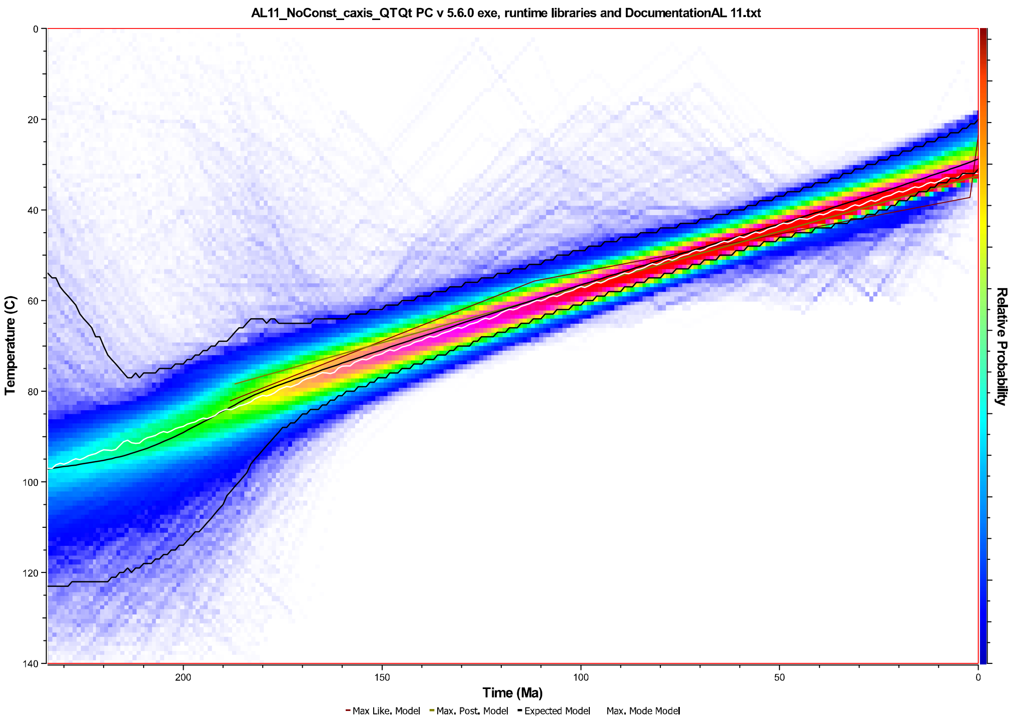
AL11_NoConst_caxis_QTQt PC v 5.6.0 exe, runtime libraries and DocumentationAL 11.txt
Temperature (C)
Time (Ma)
Relative Probability
Max Like. Model
Max. Post. Model
Expected Model
Max. Mode Model

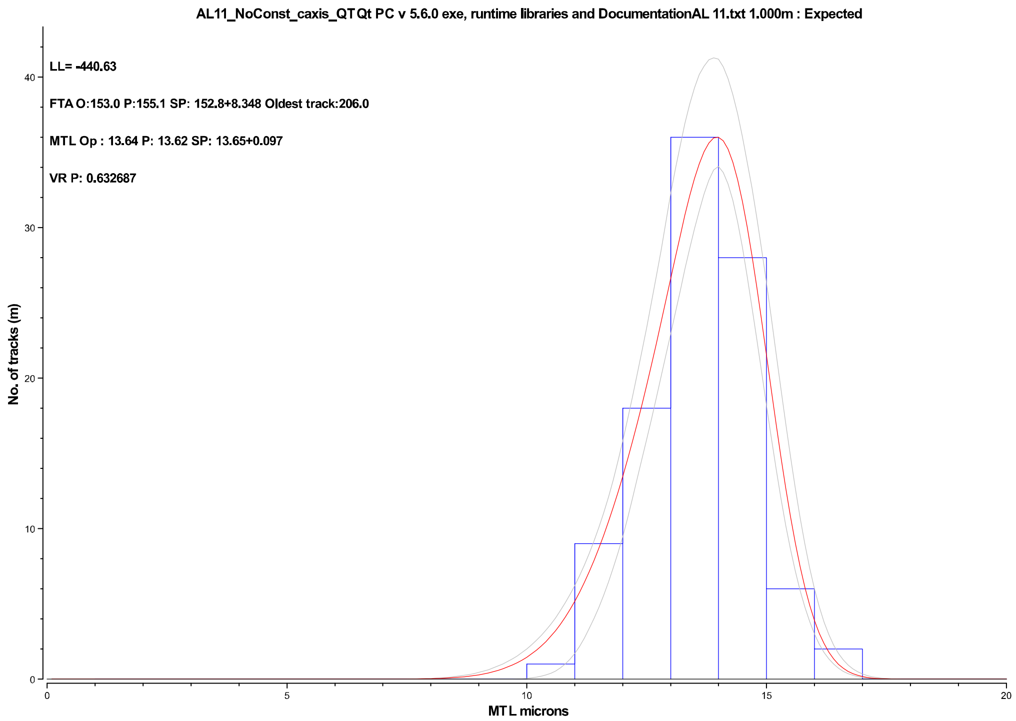
AL11_NoConst_caxis_QTQt PC v 5.6.0 exe, runtime libraries and DocumentationAL 11.txt 1.000m : Expected
LL= -440.63
FTA O:153.0 P:155.1 SP: 152.8+8.348 Oldest track:206.0
MTL Op : 13.64 P: 13.62 SP: 13.65+0.097
VR P: 0.632687
No. of tracks (m)
MTL microns

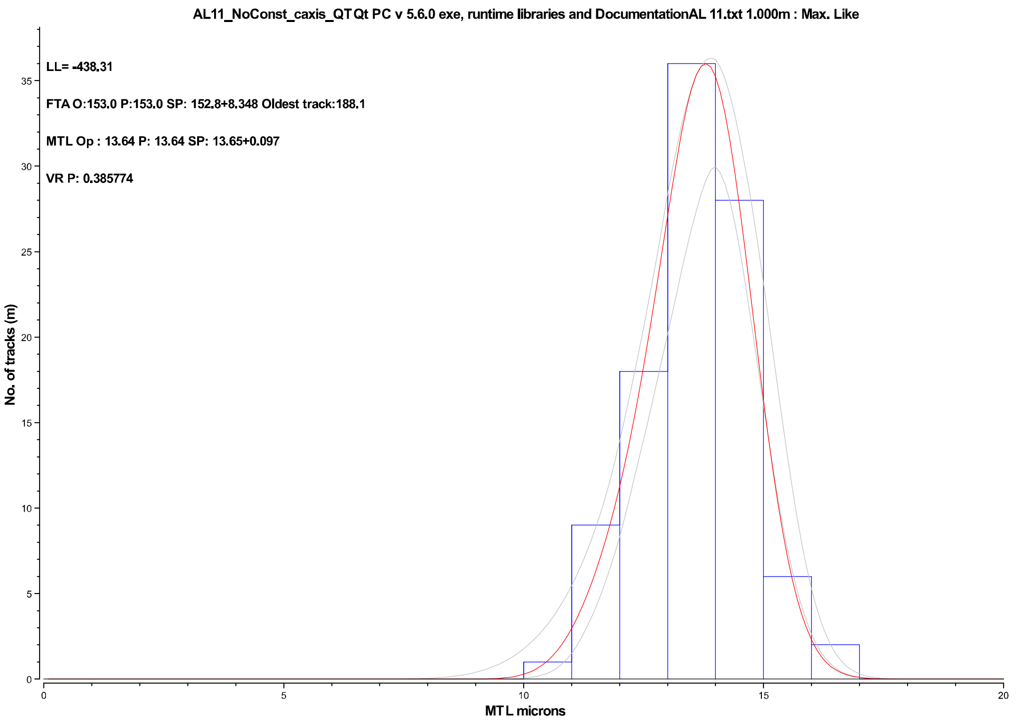
AL11_NoConst_caxis_QTQt PC v 5.6.0 exe, runtime libraries and DocumentationAL 11.txt 1.000m : Max. Like
LL= -438.31
FTA O:153.0 P:153.0 SP: 152.8+8.348 Oldest track:188.1
MTL Op : 13.64 P: 13.64 SP: 13.65+0.097
VR P: 0.385774
No. of tracks (m)
MTL microns

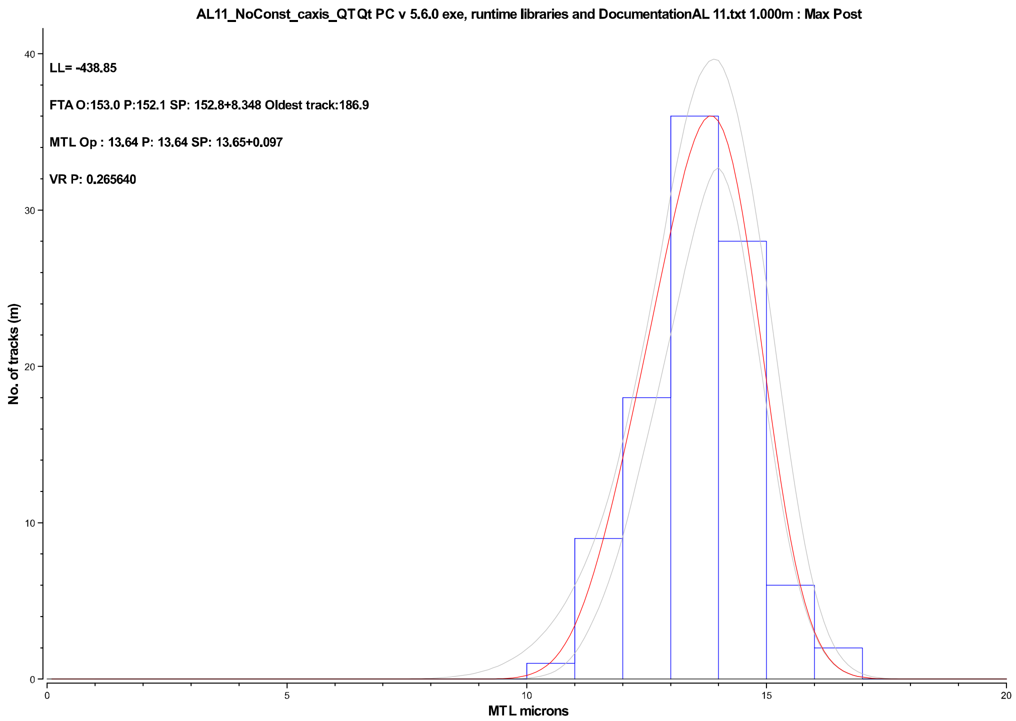
AL11_NoConst_caxis_QTQt PC v 5.6.0 exe, runtime libraries and DocumentationAL 11.txt 1.000m : Max Post
LL= -438.85
FTA O:153.0 P:152.1 SP: 152.8+8.348 Oldest track:186.9
MTL Op : 13.64 P: 13.64 SP: 13.65+0.097
VR P: 0.265640
No. of tracks (m)
MTL microns

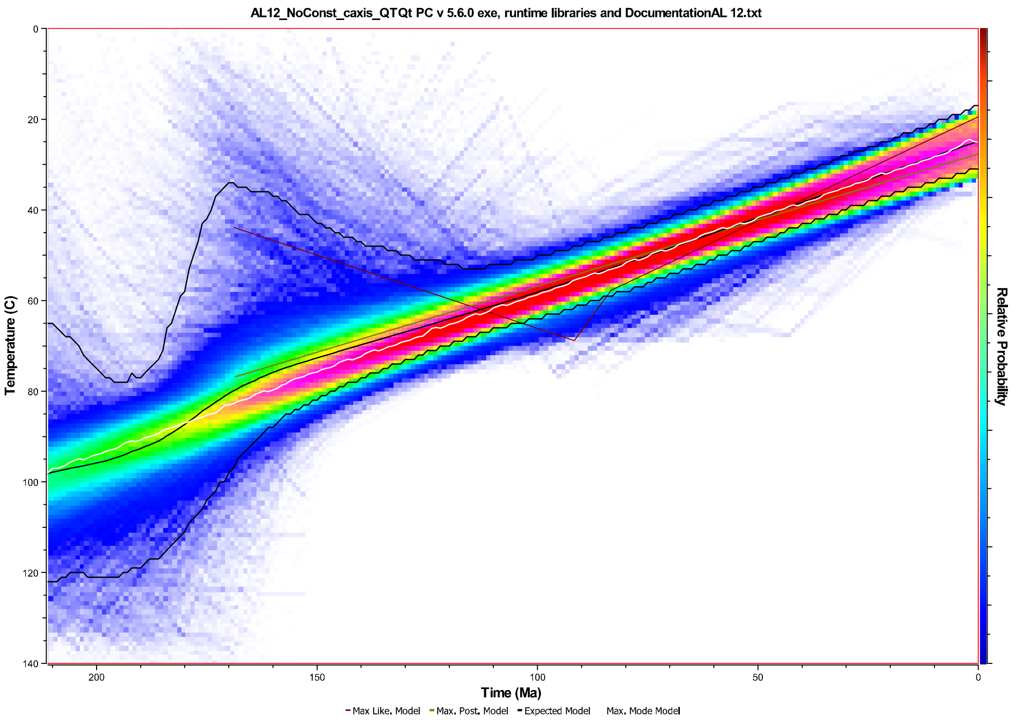

AL12_NoConst_caxis_QTQt PC v 5.6.0 exe, runtime libraries and DocumentationAL 12.txt
Temperature (C)
Time (Ma)
Relative Probability
Max Like. Model
Max. Post. Model
Expected Model
Max. Mode Model

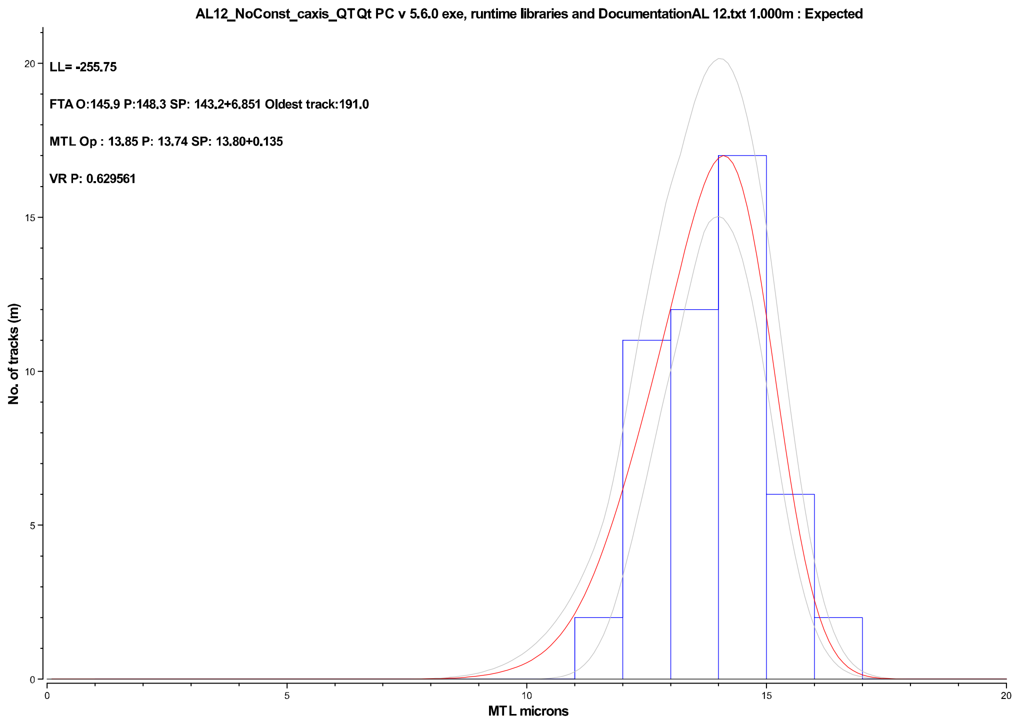

AL12_NoConst_caxis_QTQt PC v 5.6.0 exe, runtime libraries and DocumentationAL 12.txt 1.000m : Expected
LL= -255.75
FTA O:145.9 P:148.3 SP: 143.2+6.851 Oldest track:191.0
MTL Op : 13.85 P: 13.74 SP: 13.80+0.135
VR P: 0.629561
No. of tracks (m)
MTL microns

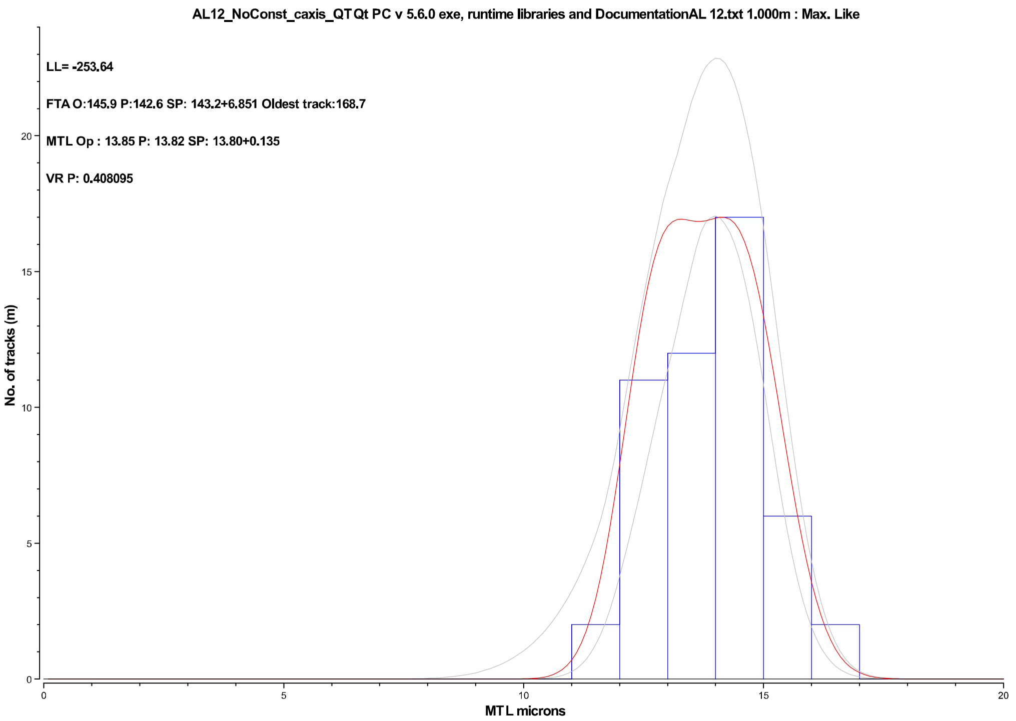

AL12_NoConst_caxis_QTQt PC v 5.6.0 exe, runtime libraries and DocumentationAL 12.txt 1.000m : Max. Like
LL= -253.64
FTA O:145.9 P:142.6 SP: 143.2+6.851 Oldest track:168.7
MTL Op : 13.85 P: 13.82 SP: 13.80+0.135
VR P: 0.408095
No. of tracks (m)
MTL microns

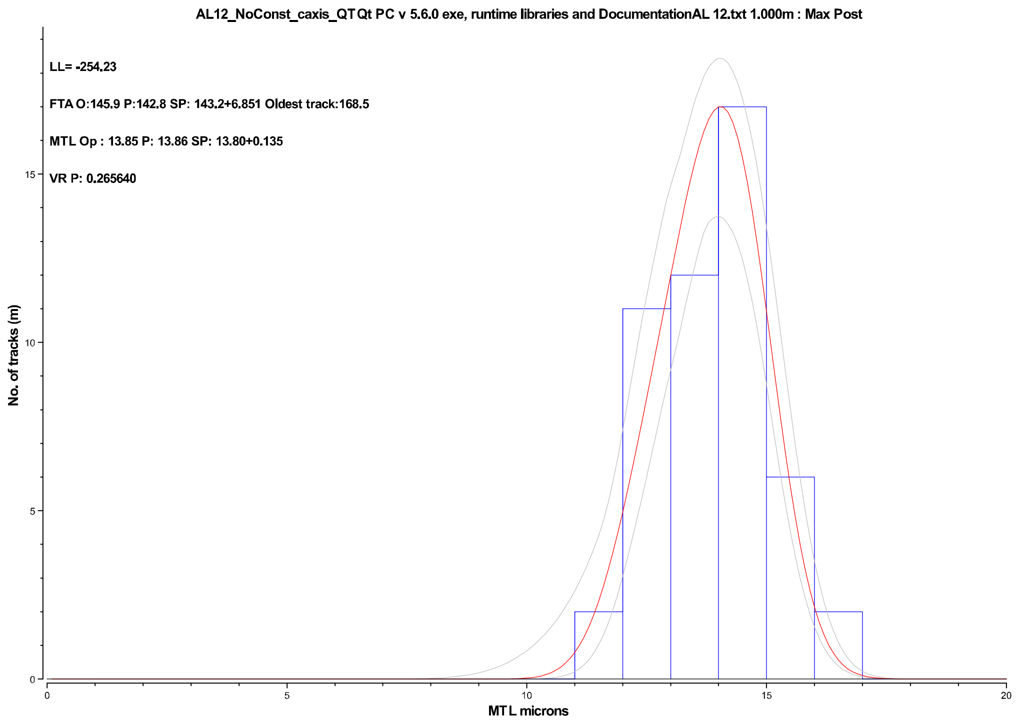

AL12_NoConst_caxis_QTQt PC v 5.6.0 exe, runtime libraries and DocumentationAL 12.txt 1.000m : Max Post
LL= -254.23
FTA O:145.9 P:142.8 SP: 143.2+6.851 Oldest track:168.5
MTL Op : 13.85 P: 13.86 SP: 13.80+0.135
VR P: 0.265640
No. of tracks (m)
MTL microns

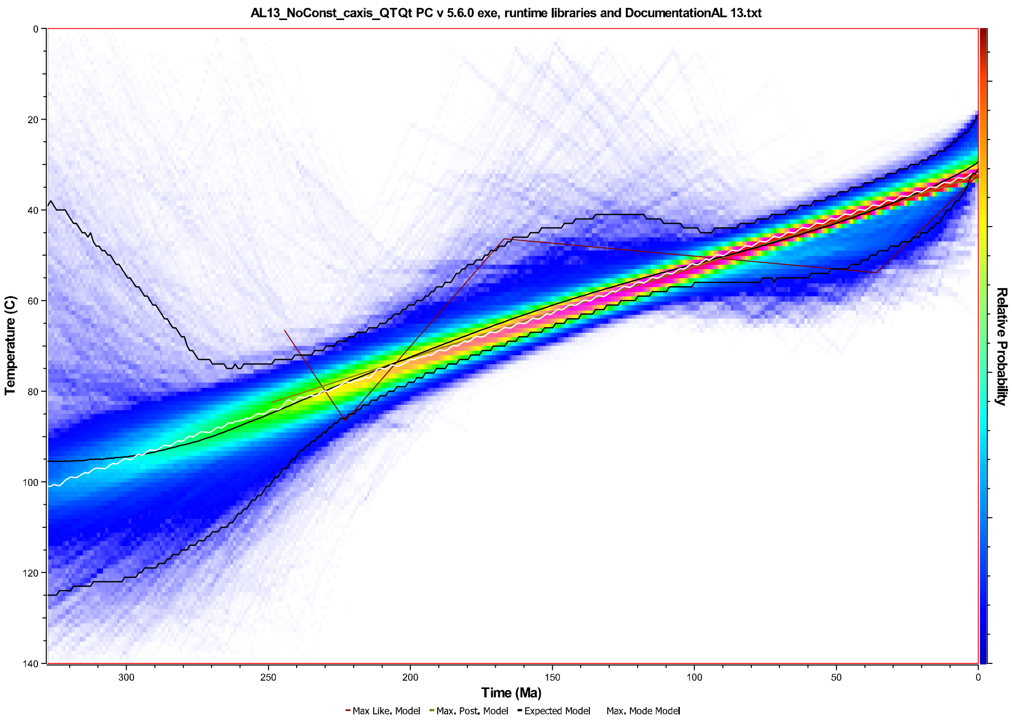
AL13_NoConst_caxis_QTQt PC v 5.6.0 exe, runtime libraries and DocumentationAL 13.txt
Temperature (C)
Time (Ma)
Relative Probability
Max Like. Model
Max. Post. Model
Expected Model
Max. Mode Model

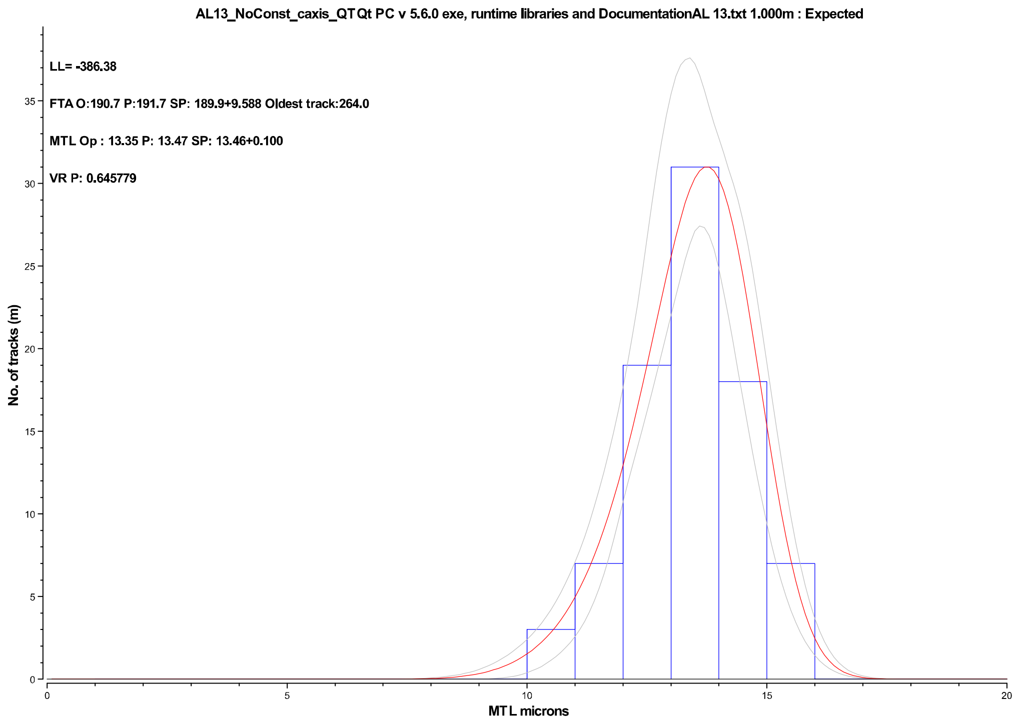
AL13_NoConst_caxis_QTQt PC v 5.6.0 exe, runtime libraries and DocumentationAL 13.txt 1.000m : Expected
LL= -386.38
FTA O:190.7 P:191.7 SP: 189.9+9.588 Oldest track:264.0
MTL Op : 13.35 P: 13.47 SP: 13.46+0.100
VR P: 0.645779
No. of tracks (m)
MTL microns

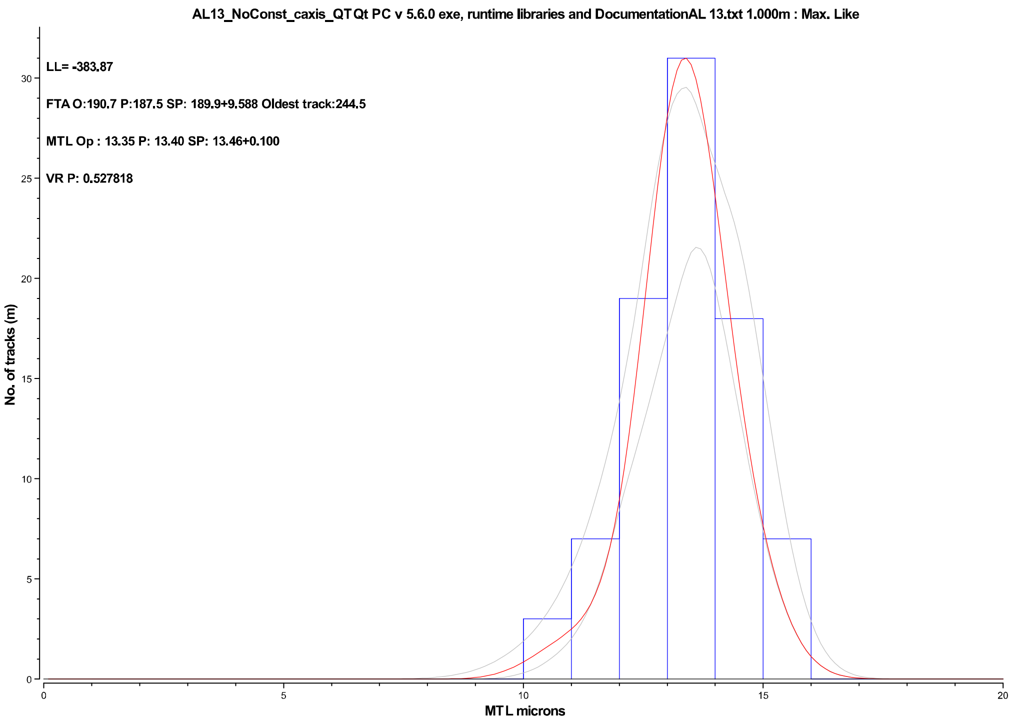
AL13_NoConst_caxis_QTQt PC v 5.6.0 exe, runtime libraries and DocumentationAL 13.txt 1.000m : Max. Like
LL= -383.87
FTA O:190.7 P:187.5 SP: 189.9+9.588 Oldest track:244.5
MTL Op : 13.35 P: 13.40 SP: 13.46+0.100
VR P: 0.527818
No. of tracks (m)
MTL microns

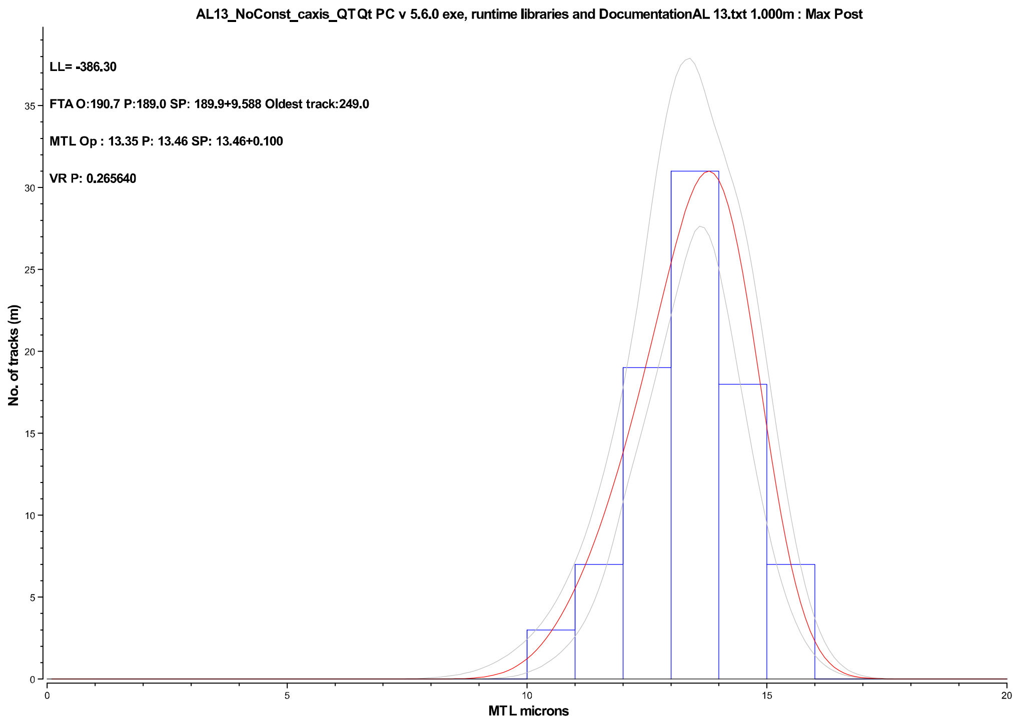
AL13_NoConst_caxis_QTQt PC v 5.6.0 exe, runtime libraries and DocumentationAL 13.txt 1.000m : Max Post
LL= -386.30
FTA O:190.7 P:189.0 SP: 189.9+9.588 Oldest track:249.0
MTL Op : 13.35 P: 13.46 SP: 13.46+0.100
VR P: 0.265640
No. of tracks (m)
MTL microns

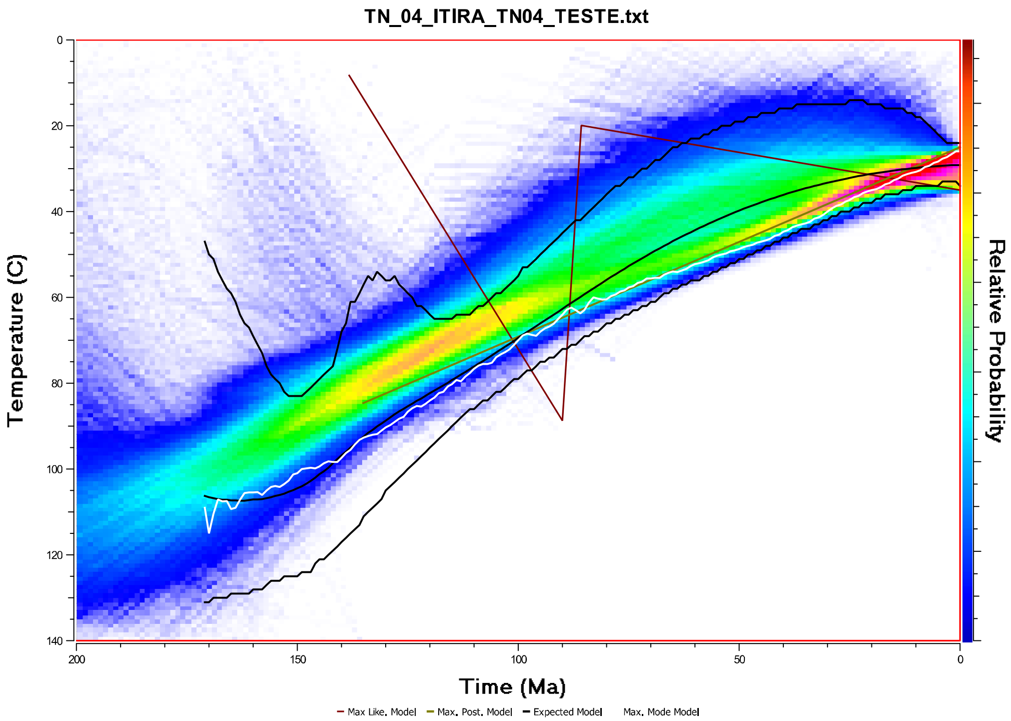
TN_04_ITIRA_TN04_TESTE.txt
Temperature (C)
Time (Ma)
Relative Probability
Max Like. Model
Max. Post. Model
Expected Model
Max. Mode Model

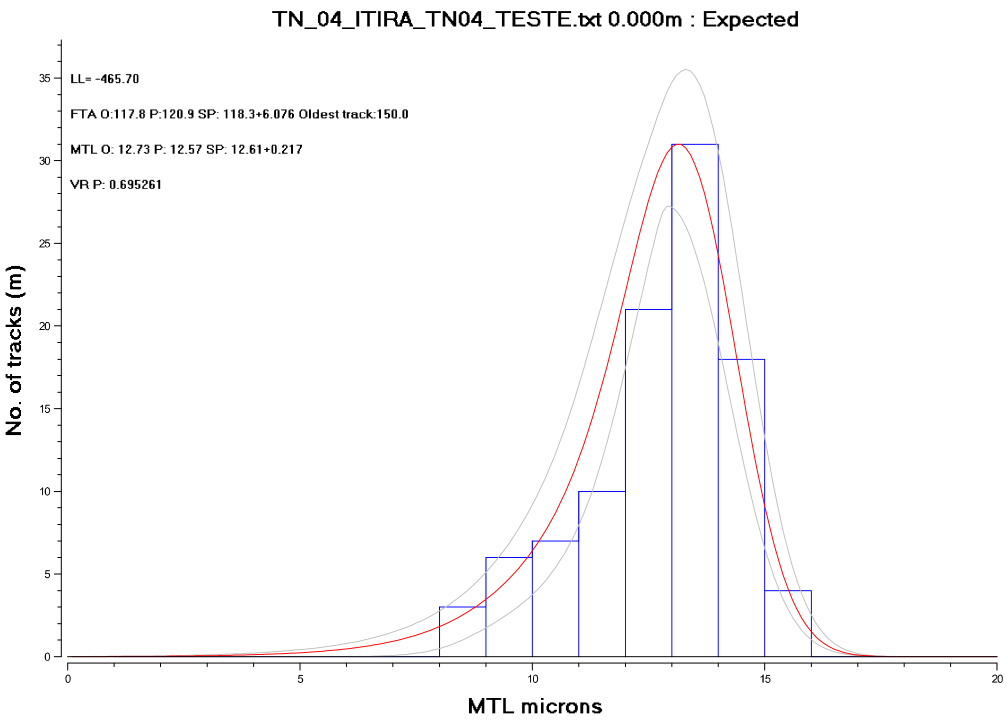
TN_04_ITIRA_TN04_TESTE.txt 0.000m : Expected
LL= -465.70
FTA O:117.8 P:120.9 SP: 118.3+6.076 Oldest track:150.0
MTL O: 12.73 P: 12.57 SP: 12.61+0.217
VR P: 0.695261
No. of tracks (m)
MTL microns

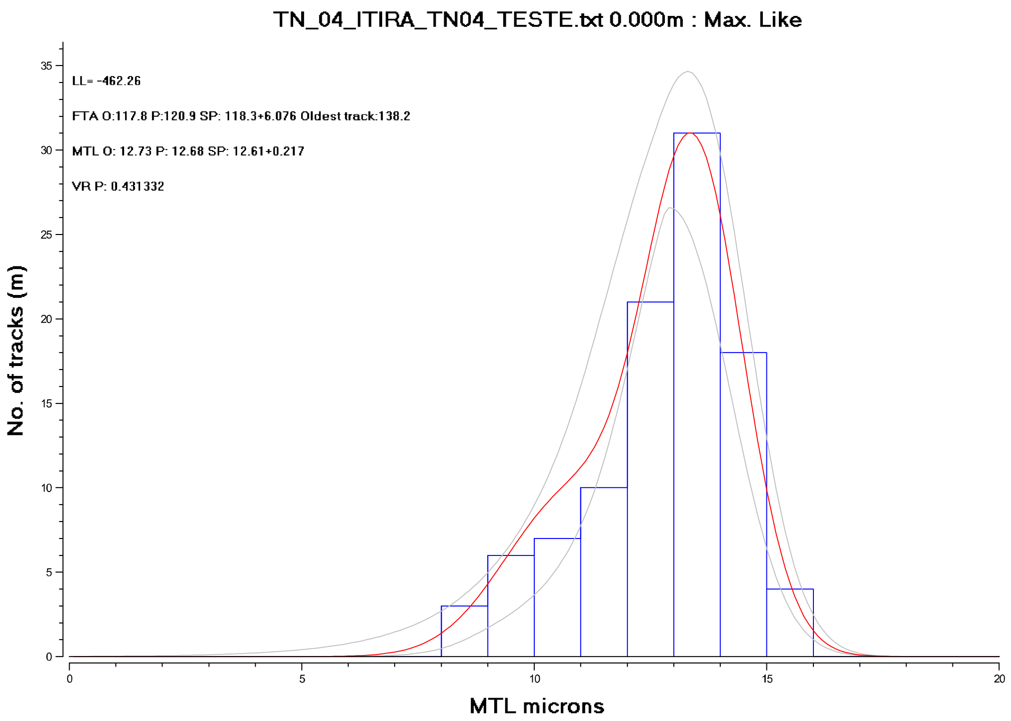
TN_04_ITIRA_TN04_TESTE.txt 0.000m : Max. Like
LL= -462.26
FTA O:117.8 P:120.9 SP: 118.3+6.076 Oldest track:138.2
MTL O: 12.73 P: 12.68 SP: 12.61+0.217
VR P: 0.431332
No. of tracks (m)
MTL microns

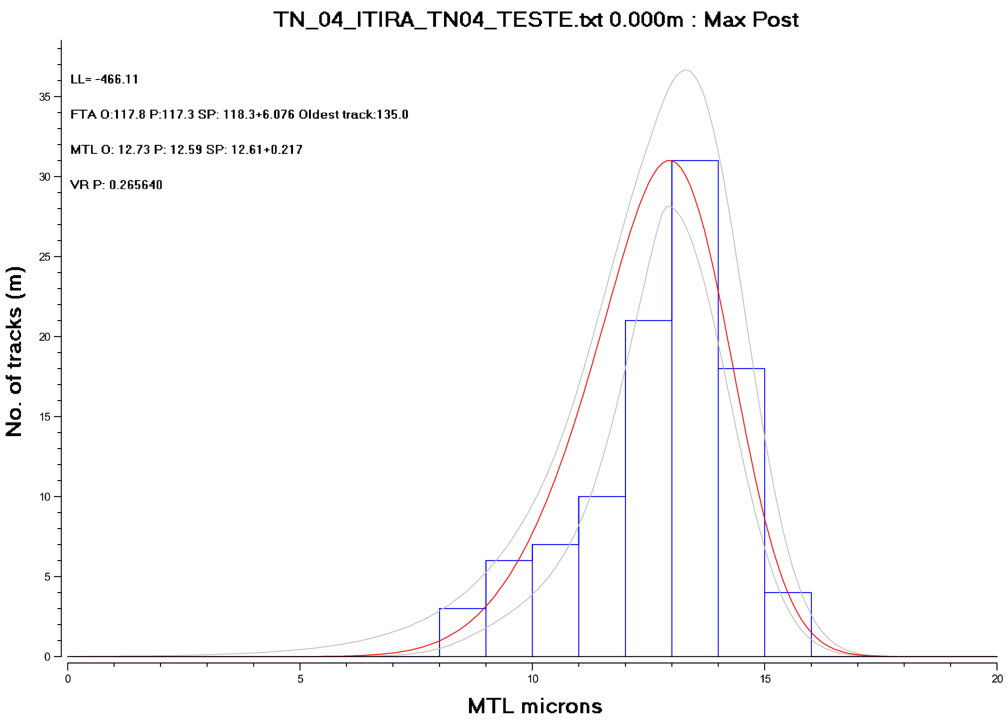
TN_04_ITIRA_TN04_TESTE.txt 0.000m : Max Post
LL= -466.11
FTA O:117.8 P:117.3 SP: 118.3+6.076 Oldest track:135.0
MTL O: 12.73 P: 12.59 SP: 12.61+0.217
VR P: 0.265640
No. of tracks (m)
MTL microns

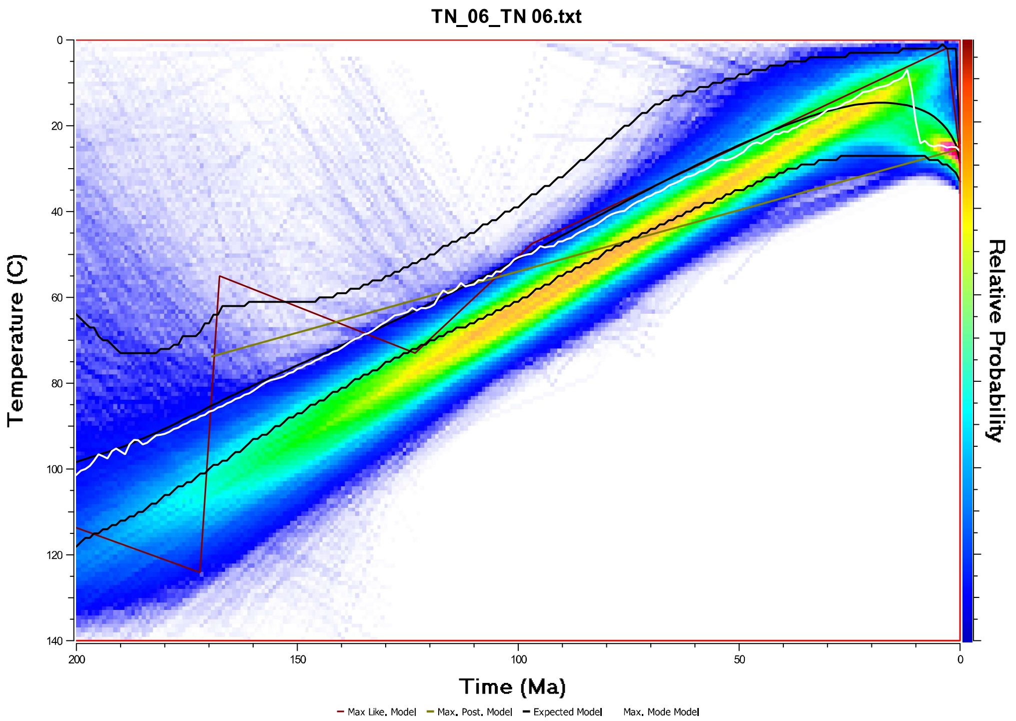
TN_06_TN 06.txt
Temperature (C)
Time (Ma)
Relative Probability
Max Like. Model
Max. Post. Model
Expected Model
Max. Mode Model

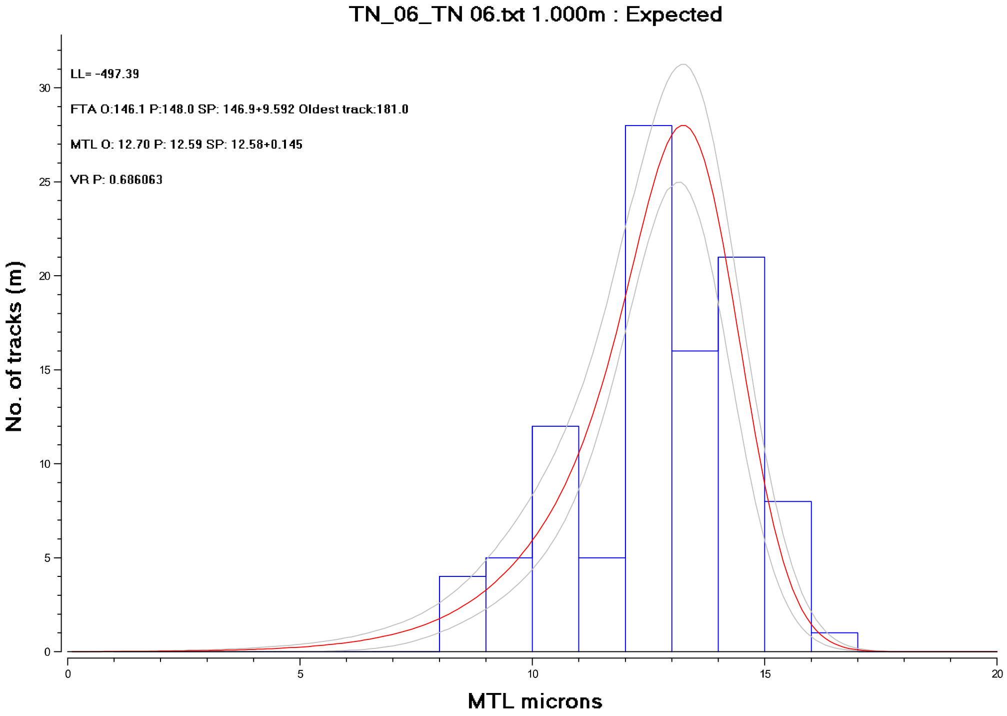
TN_06_TN 06.txt 1.000m : Expected
LL= -497.39
FTA O:146.1 P:148.0 SP: 146.9+9.592 Oldest track:181.0
MTL O: 12.70 P: 12.59 SP: 12.58+0.145
VR P: 0.686063
No. of tracks (m)
MTL microns

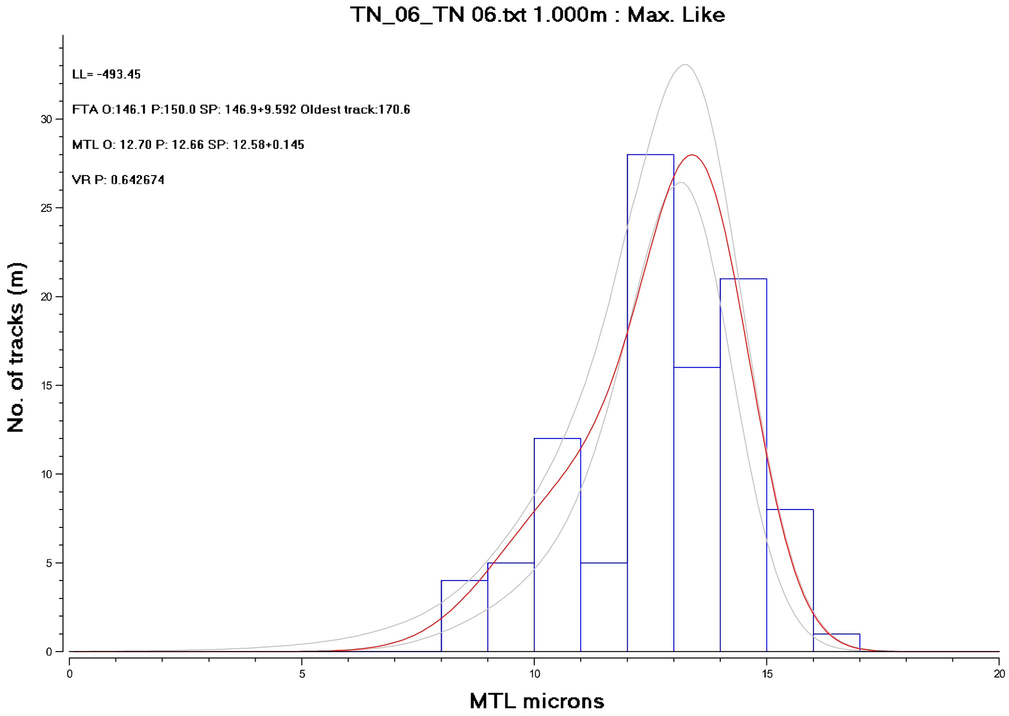
TN_06_TN 06.txt 1.000m : Max. Like
LL= -493.45
FTA O:146.1 P:150.0 SP: 146.9+9.592 Oldest track:170.6
MTL O: 12.70 P: 12.66 SP: 12.58+0.145
VR P: 0.642674
No. of tracks (m)
MTL microns

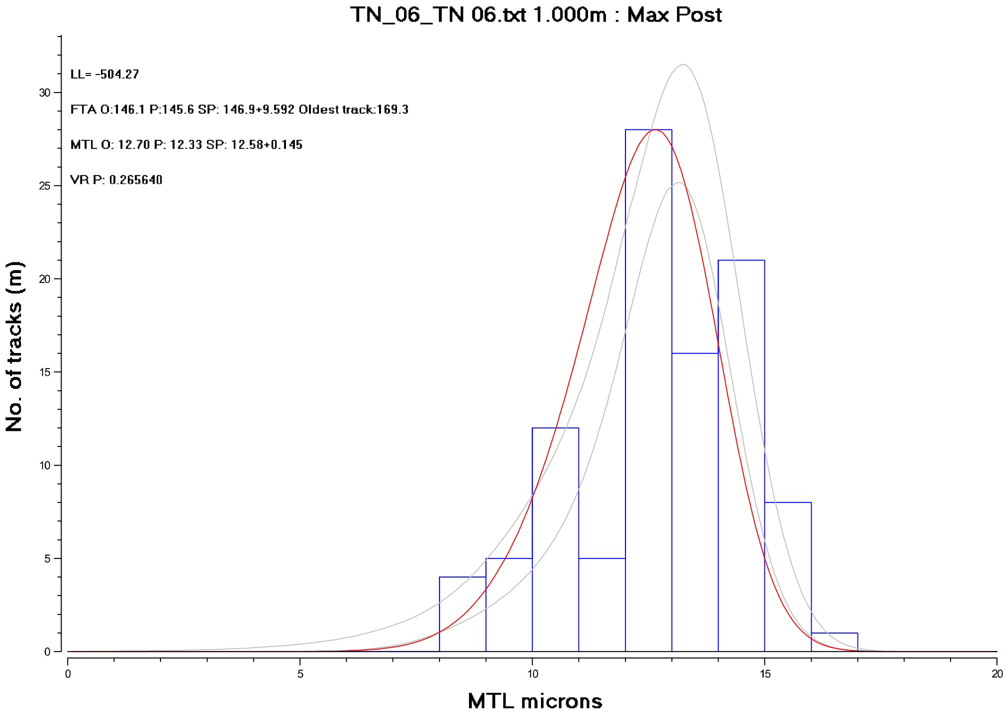
TN_06_TN 06.txt 1.000m : Max Post
LL= -504.27
FTA O:146.1 P:145.6 SP: 146.9+9.592 Oldest track:169.3
MTL O: 12.70 P: 12.33 SP: 12.58+0.145
VR P: 0.265640
No. of tracks (m)
MTL microns

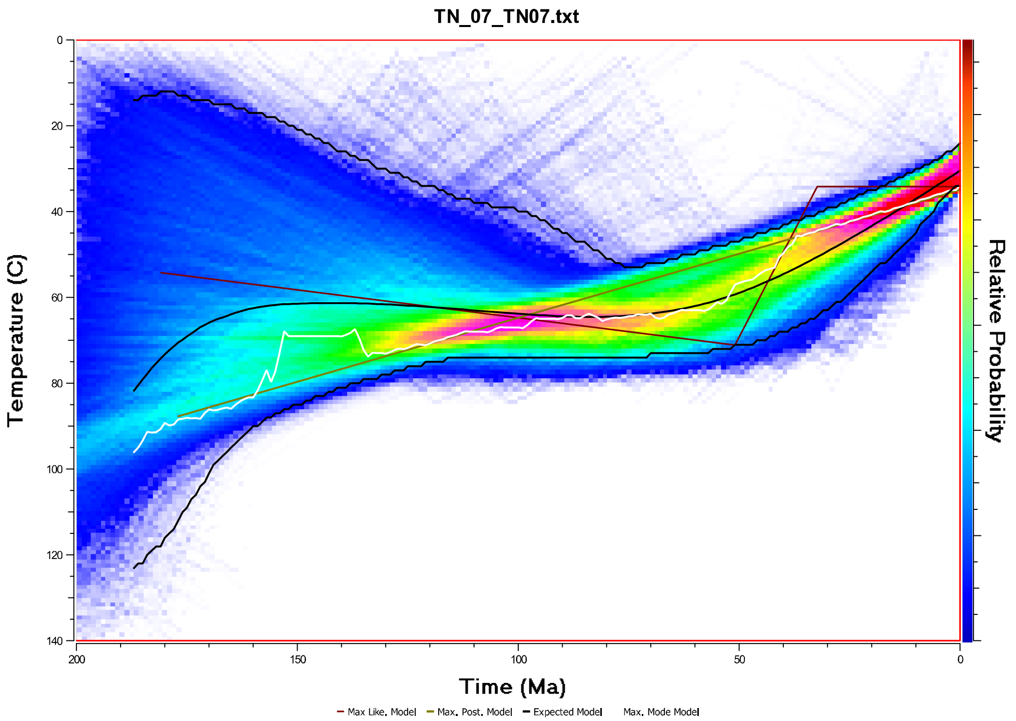
TN_07_TN07.txt
Temperature (C)
Time (Ma)
Relative Probability
Max Like. Model
Max. Post. Model
Expected Model
Max. Mode Model

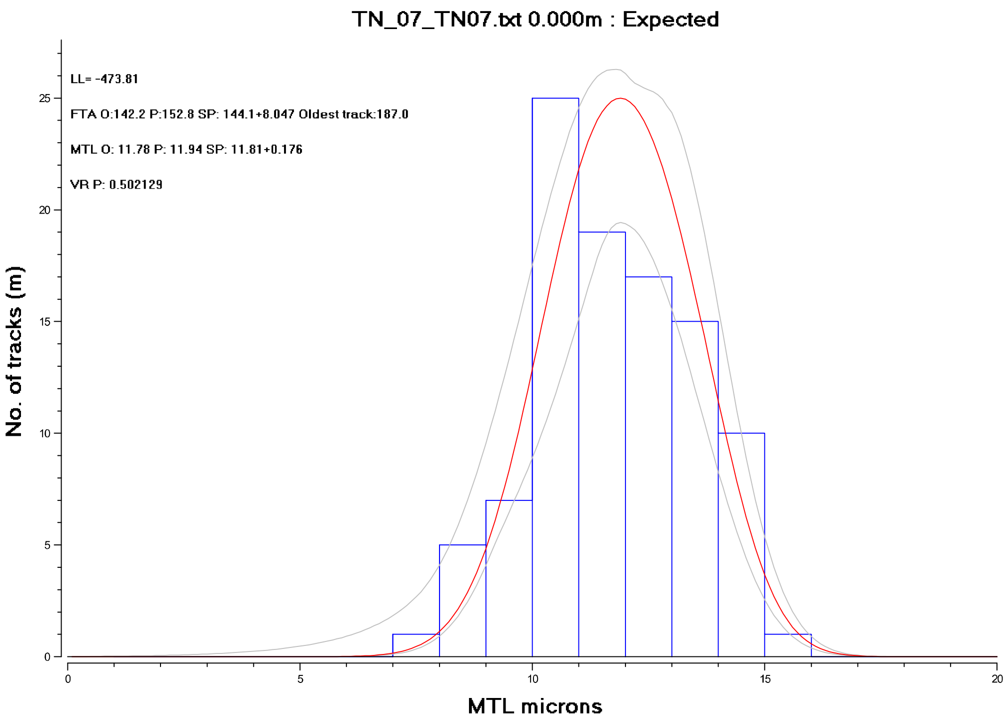
TN_07_TN07.txt 0.000m : Expected
LL= -473.81
FTA O:142.2 P:152.8 SP: 144.1+8.047 Oldest track:187.0
MTL O: 11.78 P: 11.94 SP: 11.81+0.176
VR P: 0.502129
No. of tracks (m)
MTL microns

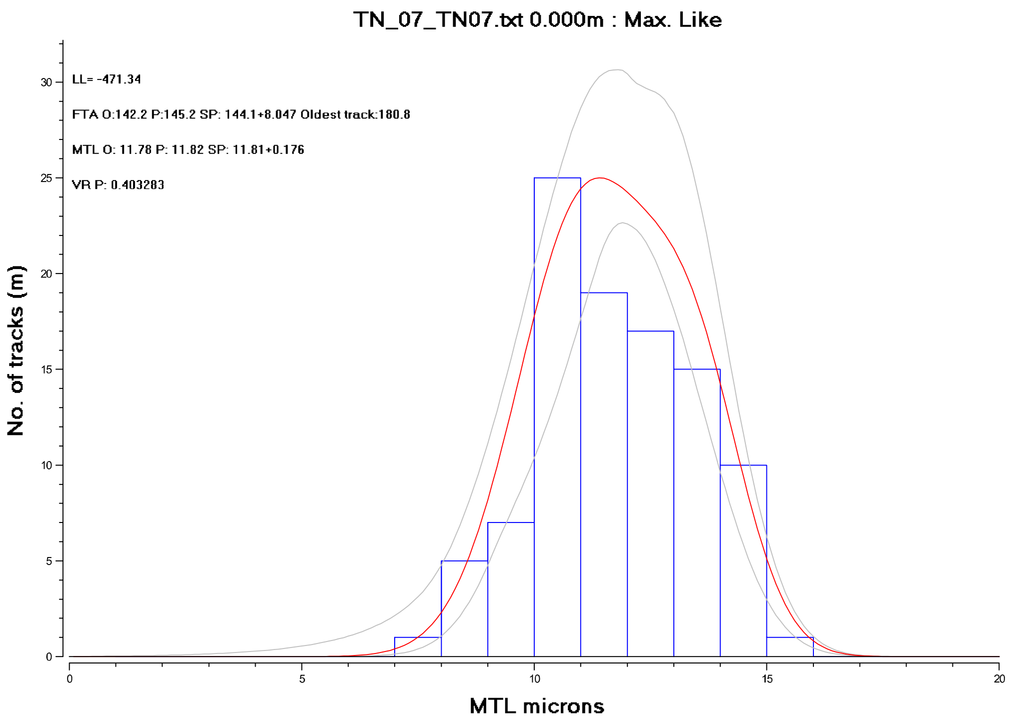
TN_07_TN07.txt 0.000m : Max. Like
LL= -471.34
FTA O:142.2 P:145.2 SP: 144.1+8.047 Oldest track:180.8
MTL O: 11.78 P: 11.82 SP: 11.81+0.176
VR P: 0.403283
No. of tracks (m)
MTL microns

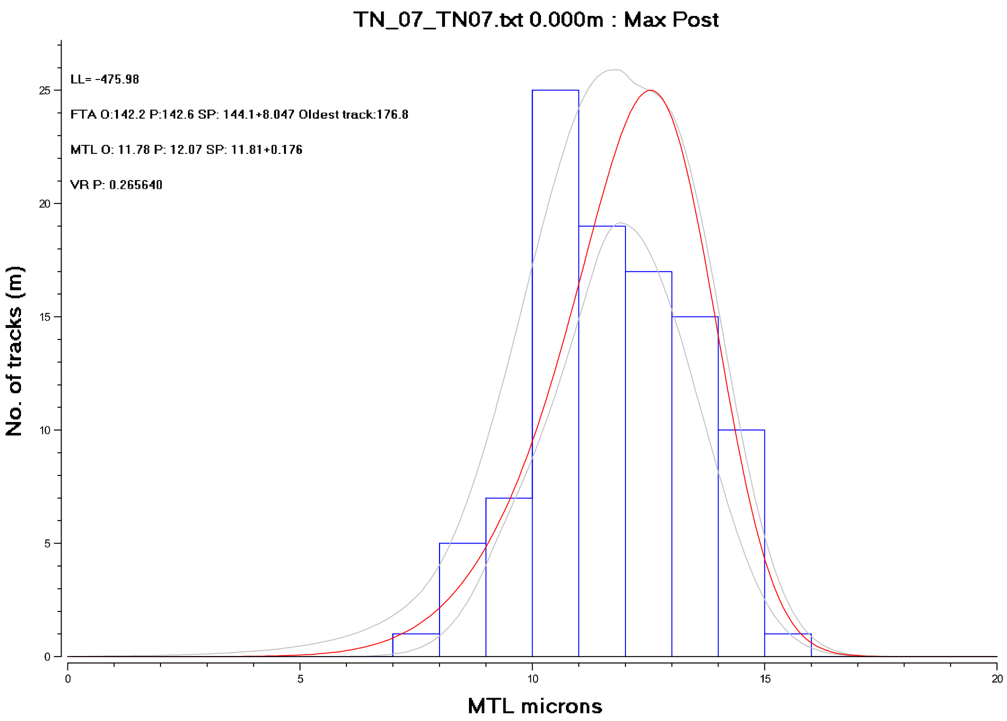
TN_07_TN07.txt 0.000m : Max Post
LL= -475.98
FTA O:142.2 P:142.6 SP: 144.1+8.047 Oldest track:176.8
MTL O: 11.78 P: 12.07 SP: 11.81+0.176
VR P: 0.265640
No. of tracks (m)
MTL microns

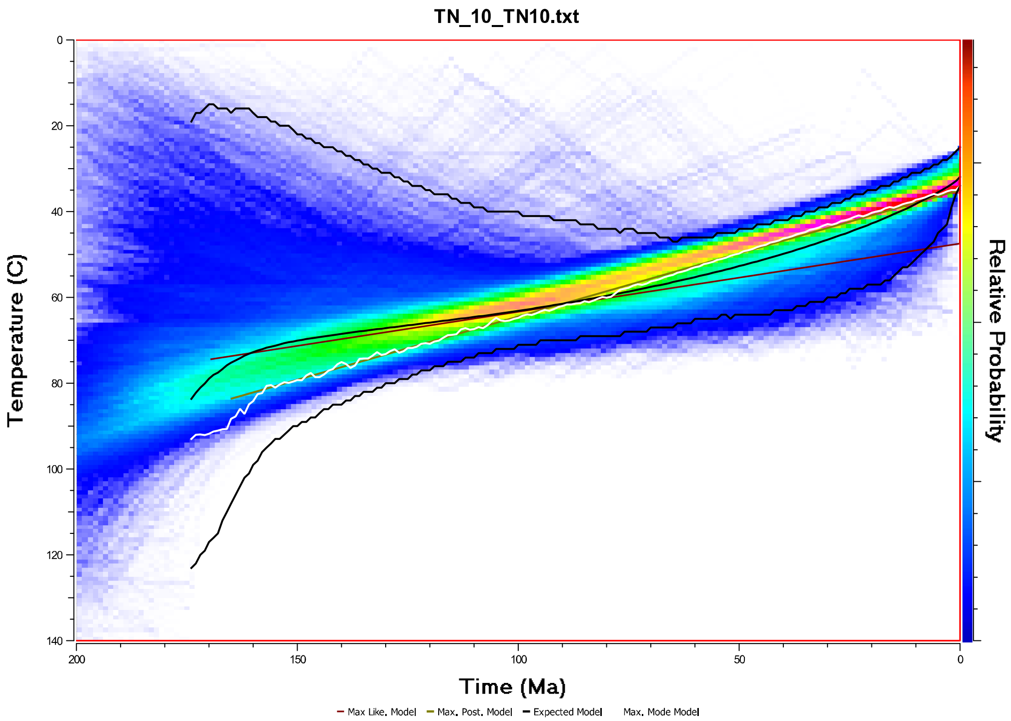

TN_10_TN10.txt
Temperature (C)
Time (Ma)
Relative Probability
Max Like. Model
Max. Post. Model
Expected Model
Max. Mode Model

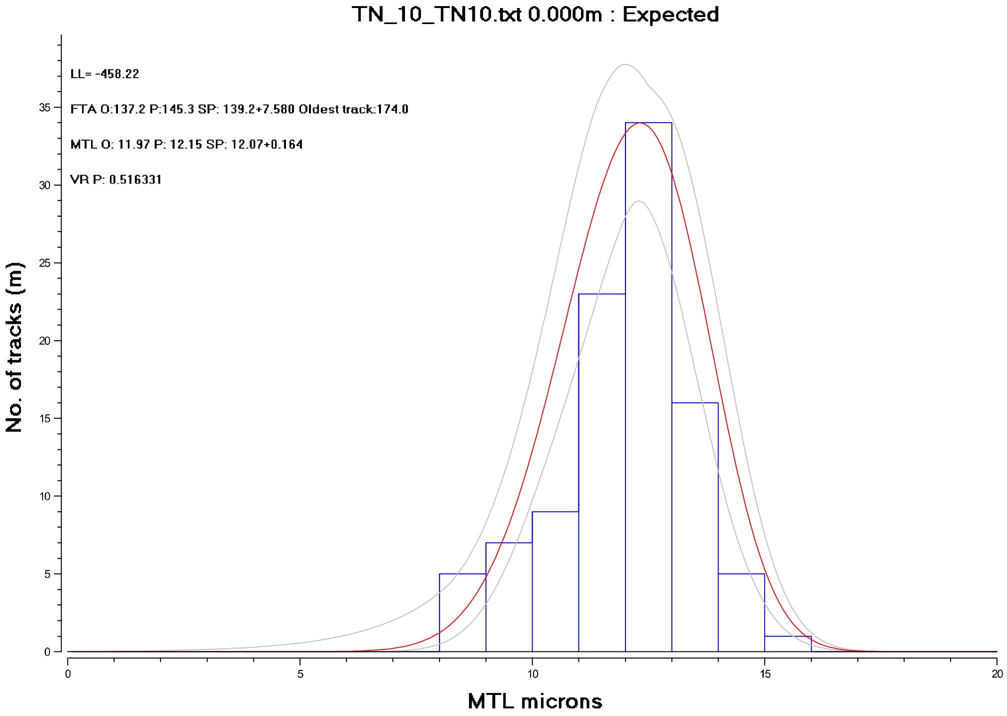

TN_10_TN10.txt 0.000m : Expected
LL= -458.22
FTA O:137.2 P:145.3 SP: 139.2+7.580 Oldest track:174.0
MTL O: 11.97 P: 12.15 SP: 12.07+0.164
VR P: 0.516331
No. of tracks (m)
MTL microns

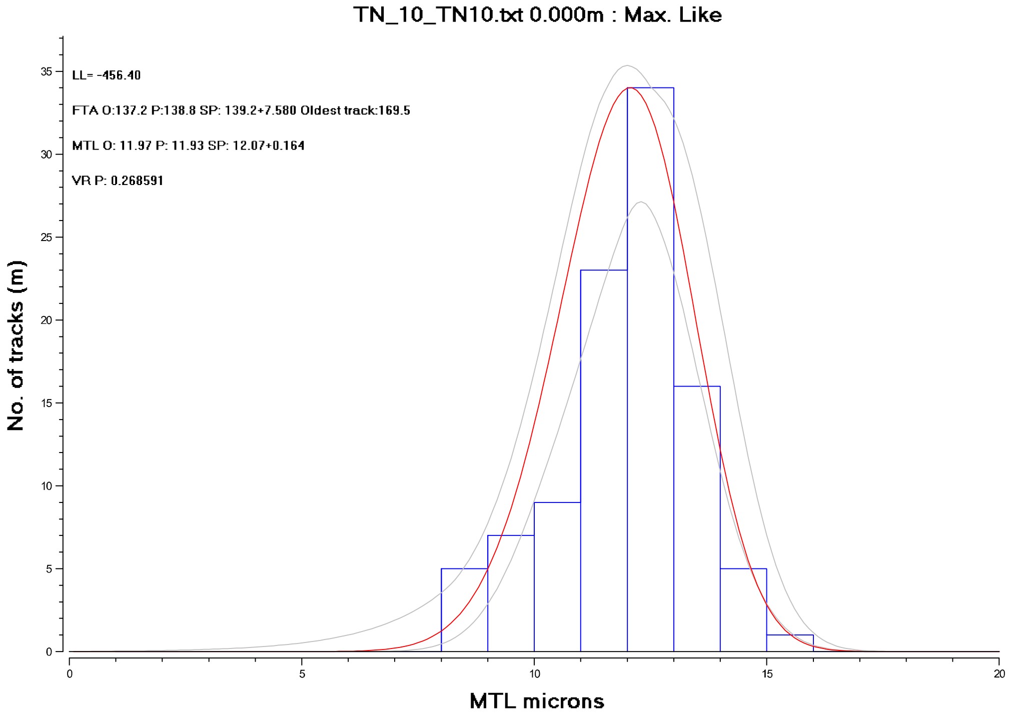

TN_10_TN10.txt 0.000m : Max. Like
LL= -456.40
FTA O:137.2 P:138.8 SP: 139.2+7.580 Oldest track:169.5
MTL O: 11.97 P: 11.93 SP: 12.07+0.164
VR P: 0.268591
No. of tracks (m)
MTL microns

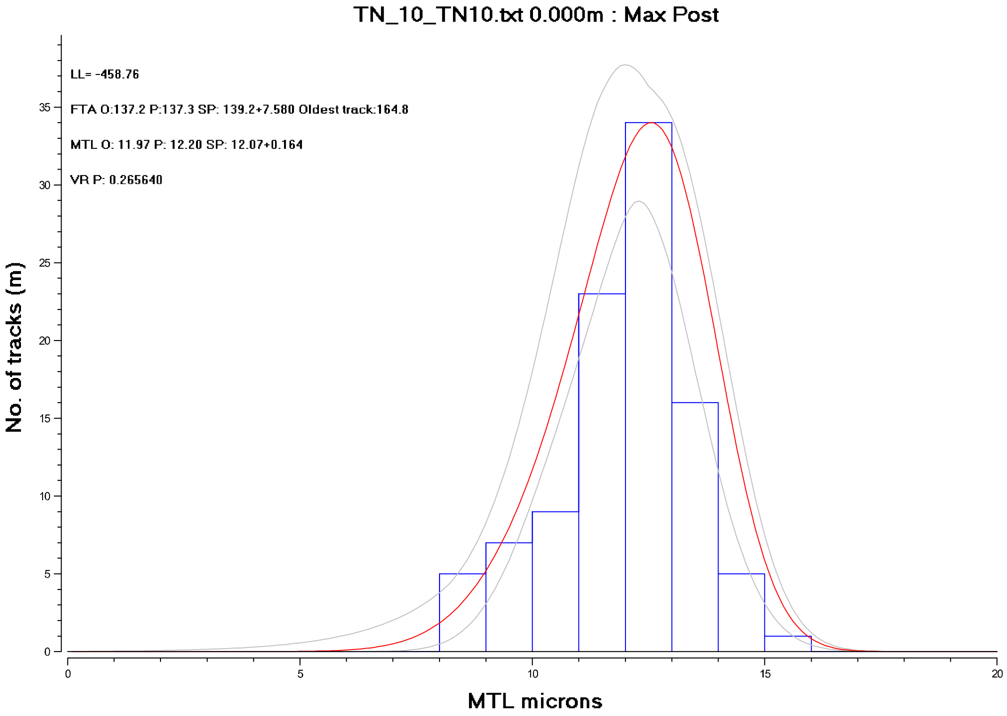

TN_10_TN10.txt 0.000m : Max Post
LL= -458.76
FTA O:137.2 P:137.3 SP: 139.2+7.580 Oldest track:164.8
MTL O: 11.97 P: 12.20 SP: 12.07+0.164
VR P: 0.265640
No. of tracks (m)
MTL microns

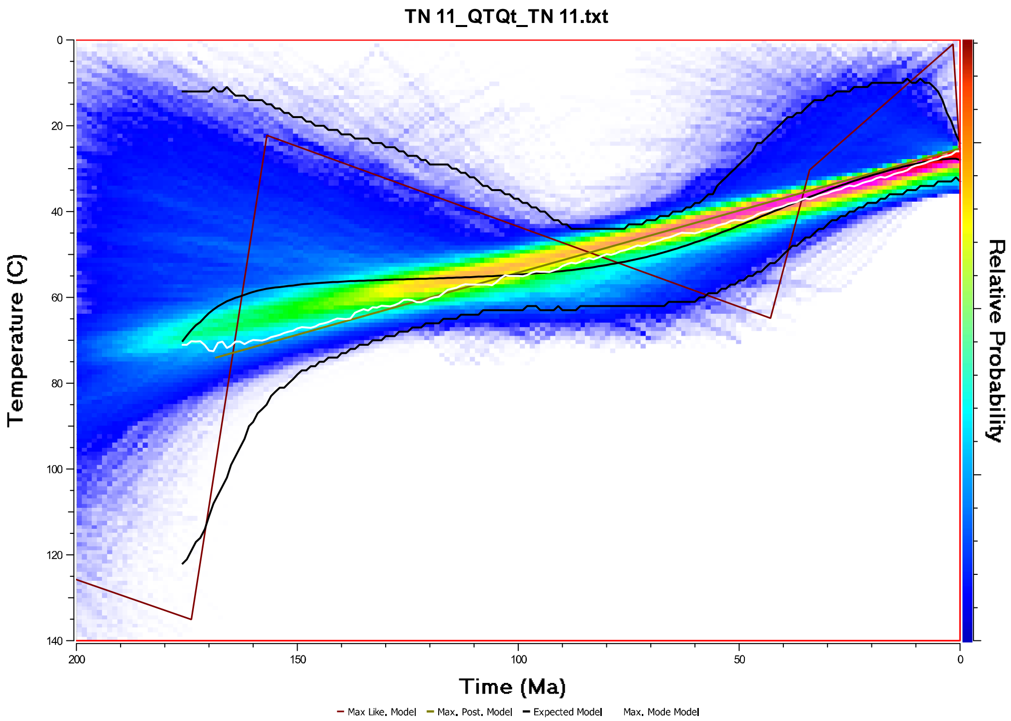
TN 11_QTQt_TN 11.txt
Temperature (C)
Time (Ma)
Relative Probability
Max Like. Model
Max. Post. Model
Expected Model
Max. Mode Model

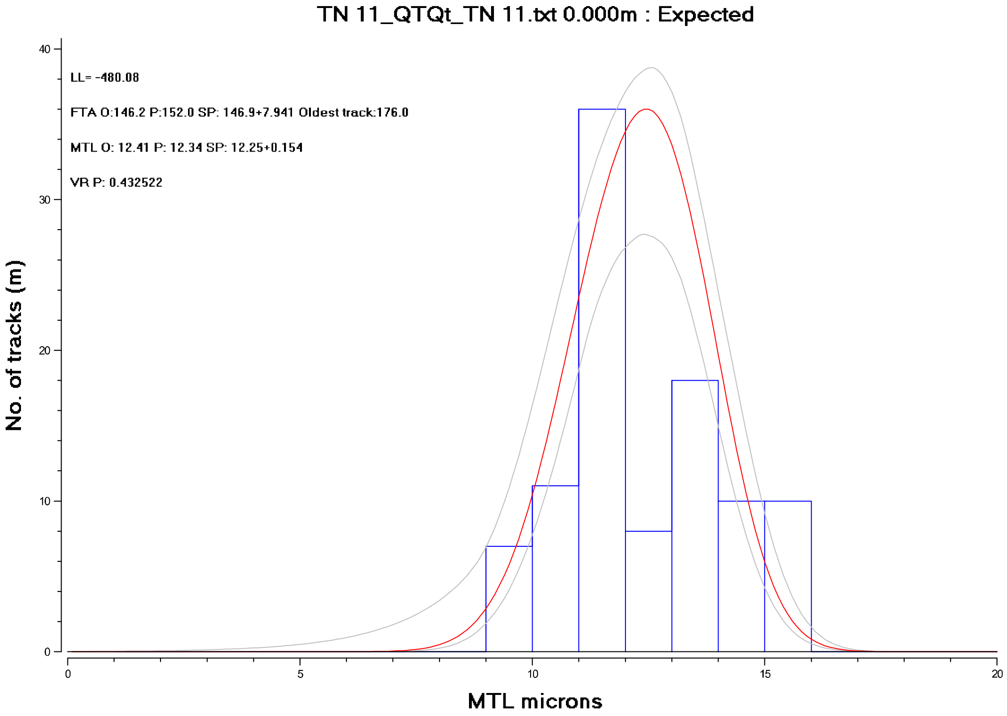
TN 11_QTQt_TN 11.txt 0.000m : Expected
LL= -480.08
FTA O:146.2 P:152.0 SP: 146.9+7.941 Oldest track:176.0
MTL O: 12.41 P: 12.34 SP: 12.25+0.154
VR P: 0.432522
No. of tracks (m)
MTL microns

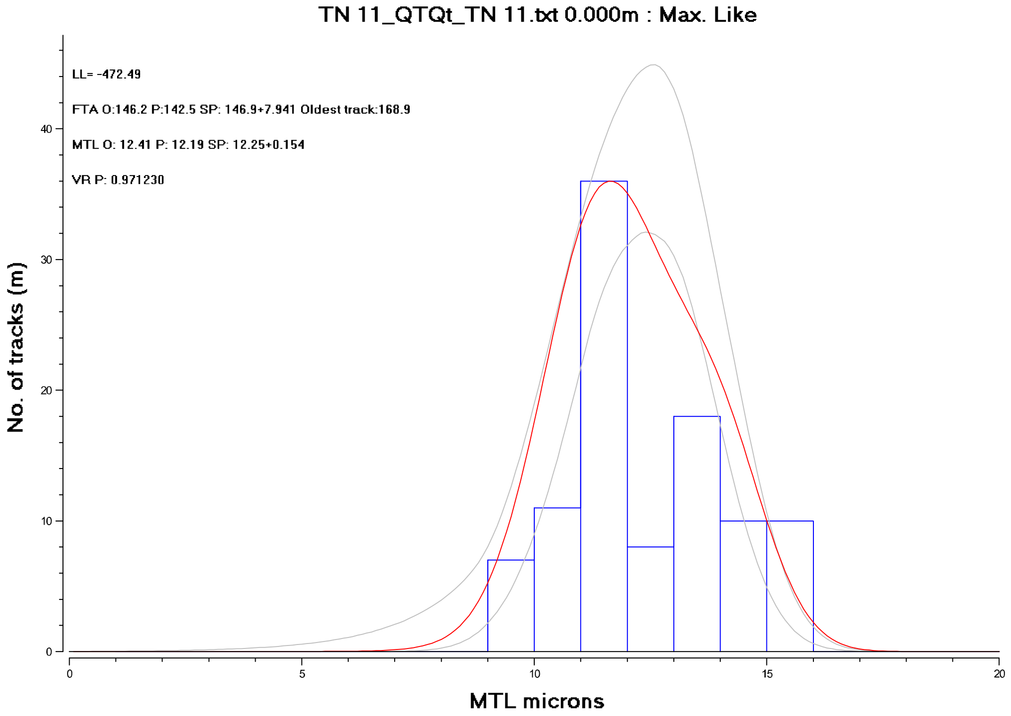
TN 11_QTQt_TN 11.txt 0.000m : Max. Like
LL= -472.49
FTA O:146.2 P:142.5 SP: 146.9+7.941 Oldest track:168.9
MTL O: 12.41 P: 12.19 SP: 12.25+0.154
VR P: 0.971230
No. of tracks (m)
MTL microns

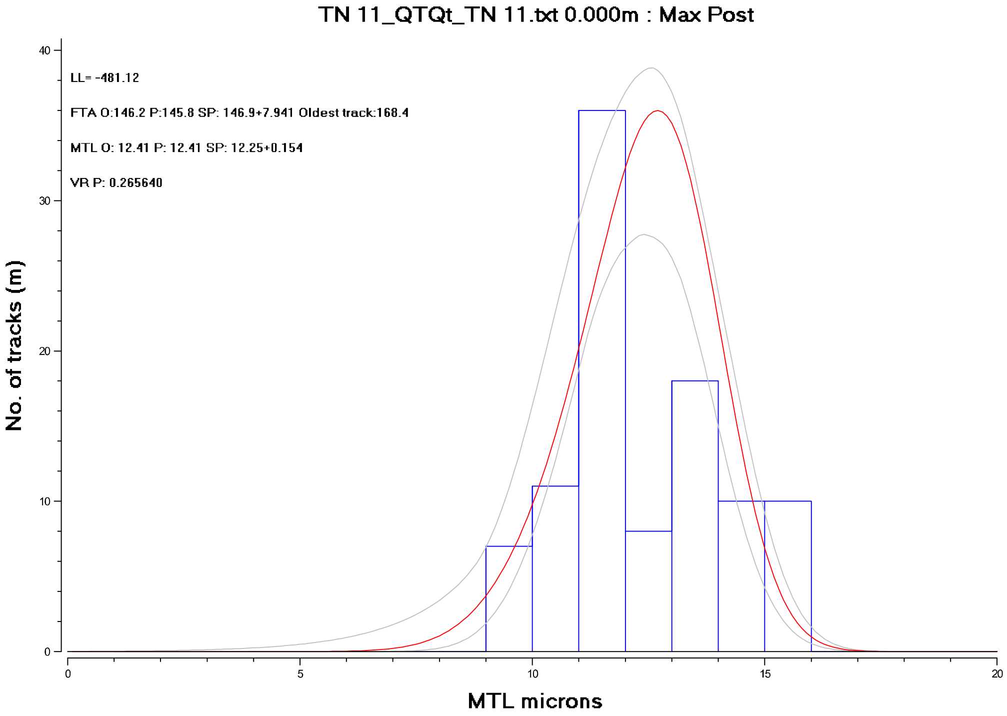
TN 11_QTQt_TN 11.txt 0.000m : Max Post
LL= -481.12
FTA O:146.2 P:145.8 SP: 146.9+7.941 Oldest track:168.4
MTL O: 12.41 P: 12.41 SP: 12.25+0.154
VR P: 0.265640
No. of tracks (m)
MTL microns

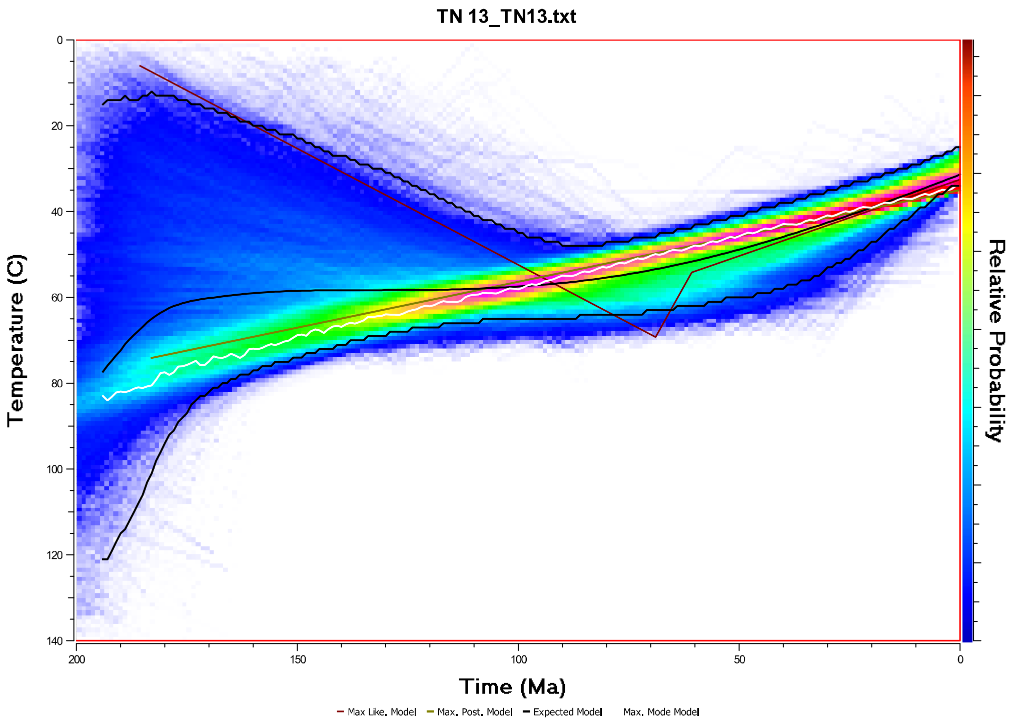

TN 13_TN13.txt
Temperature (C)
Time (Ma)
Relative Probability
Max Like. Model
Max. Post. Model
Expected Model
Max. Mode Model

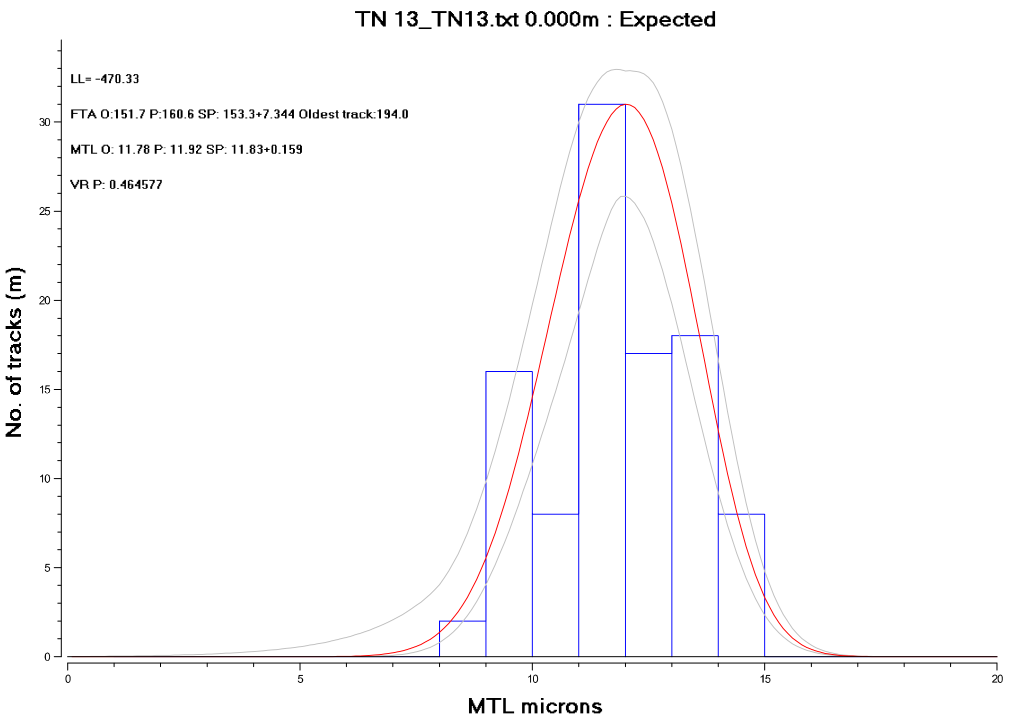

TN 13_TN13.txt 0.000m : Expected
LL= -470.33
FTA O:151.7 P:160.6 SP: 153.3+7.344 Oldest track:194.0
MTL O: 11.78 P: 11.92 SP: 11.83+0.159
VR P: 0.464577
No. of tracks (m)
MTL microns

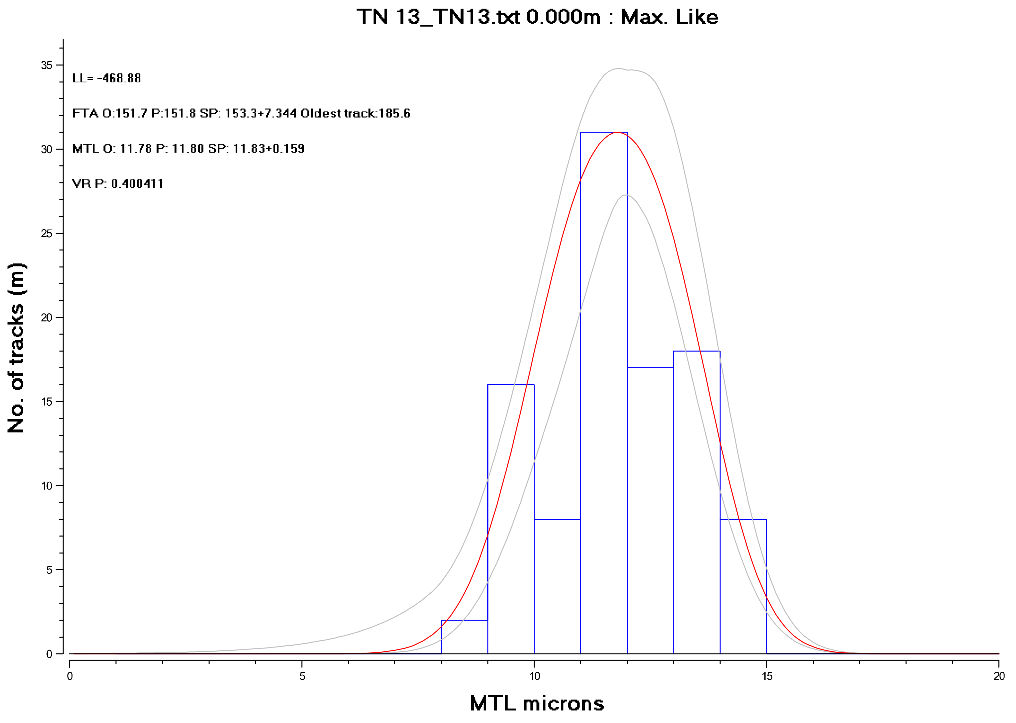

TN 13_TN13.txt 0.000m : Max. Like
LL= -468.88
FTA O:151.7 P:151.8 SP: 153.3+7.344 Oldest track:185.6
MTL O: 11.78 P: 11.80 SP: 11.83+0.159
VR P: 0.400411
No. of tracks (m)
MTL microns

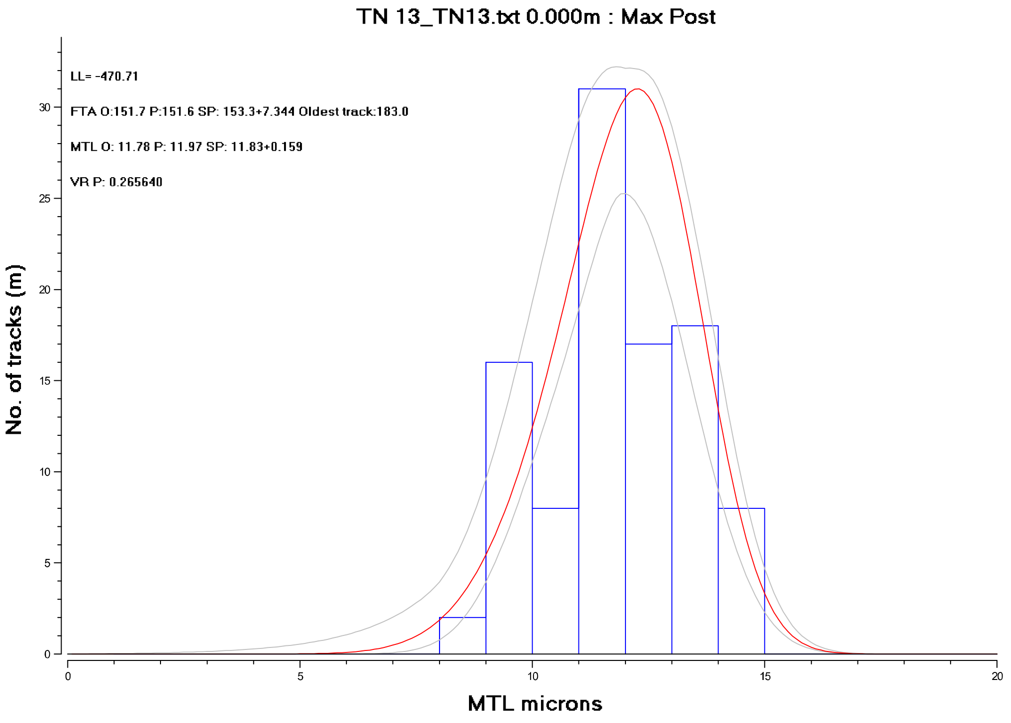

TN 13_TN13.txt 0.000m : Max Post
LL= -470.71
FTA O:151.7 P:151.6 SP: 153.3+7.344 Oldest track:183.0
MTL O: 11.78 P: 11.97 SP: 11.83+0.159
VR P: 0.265640
No. of tracks (m)
MTL microns

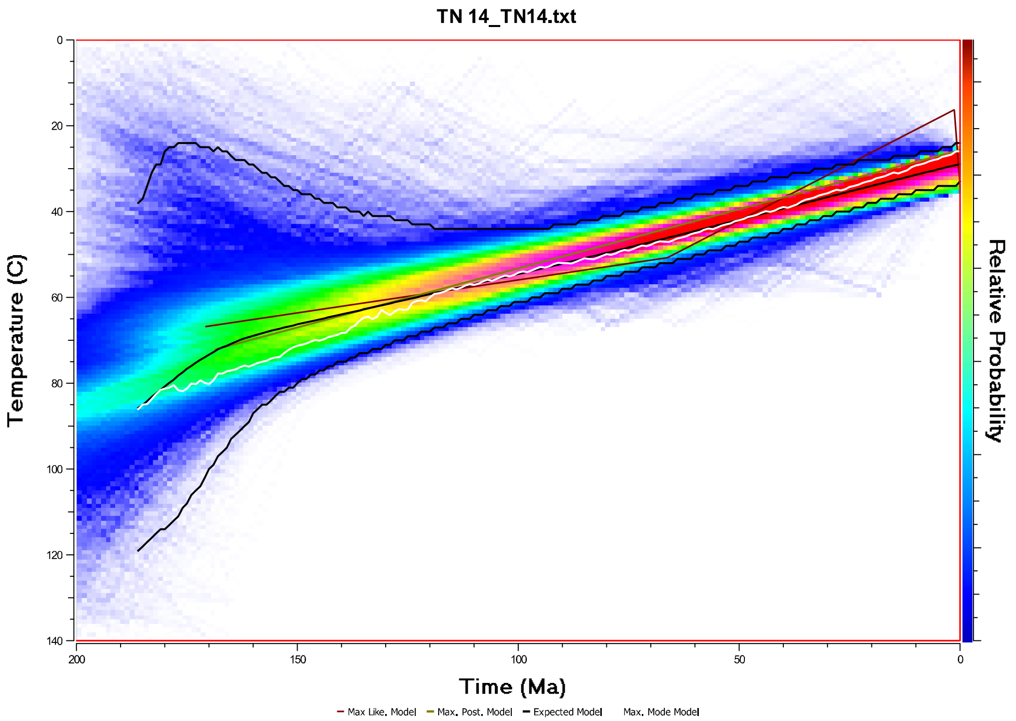
TN 14_TN14.txt
Temperature (C)
Time (Ma)
Relative Probability
Max Like. Model
Max. Post. Model
Expected Model
Max. Mode Model

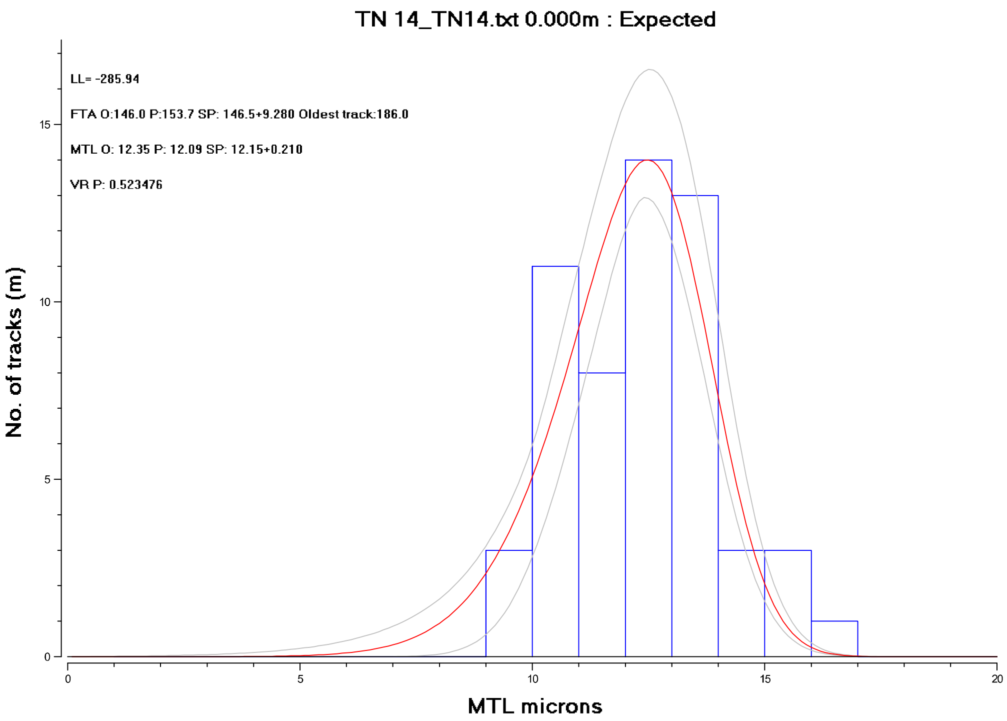
TN 14_TN14.txt 0.000m : Expected
LL= -285.94
FTA O:146.0 P:153.7 SP: 146.5+9.280 Oldest track:186.0
MTL O: 12.35 P: 12.09 SP: 12.15+0.210
VR P: 0.523476
No. of tracks (m)
MTL microns

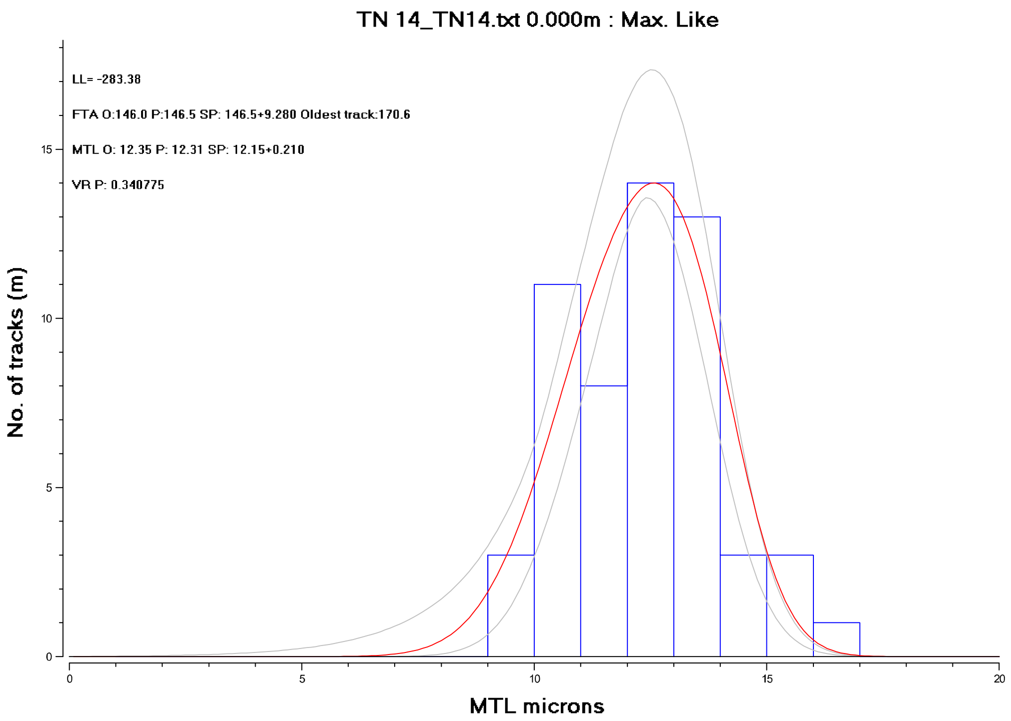
TN 14_TN14.txt 0.000m : Max. Like
LL= -283.38
FTA O:146.0 P:146.5 SP: 146.5+9.280 Oldest track:170.6
MTL O: 12.35 P: 12.31 SP: 12.15+0.210
VR P: 0.340775
No. of tracks (m)
MTL microns

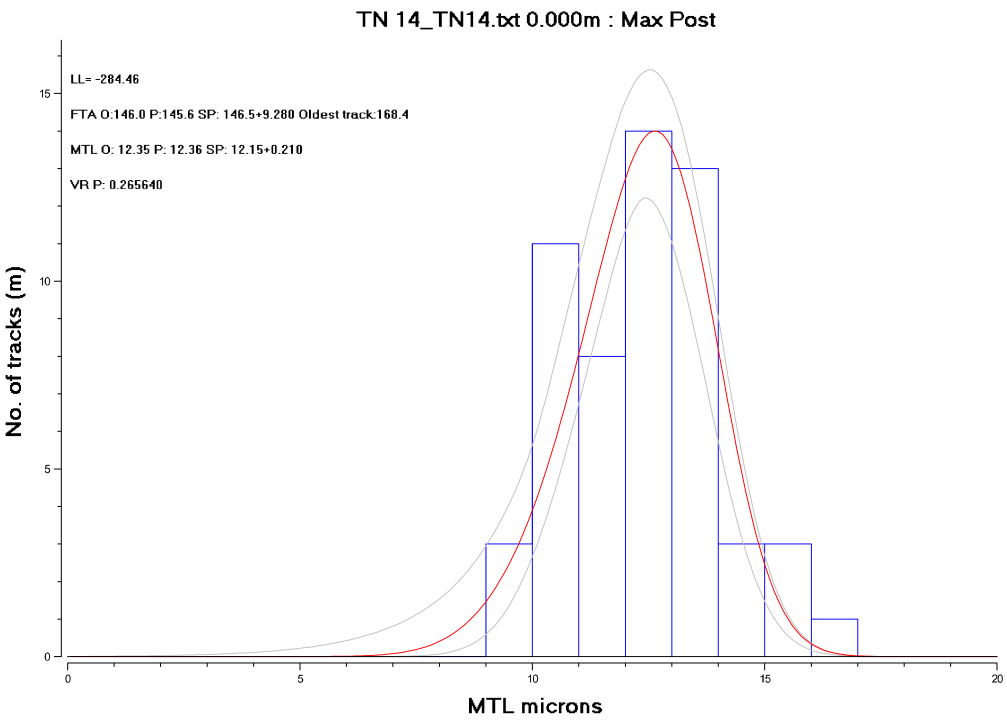
TN 14_TN14.txt 0.000m : Max Post
LL= -284.46
FTA O:146.0 P:145.6 SP: 146.5+9.280 Oldest track:168.4
MTL O: 12.35 P: 12.36 SP: 12.15+0.210
VR P: 0.265640
No. of tracks (m)
MTL microns

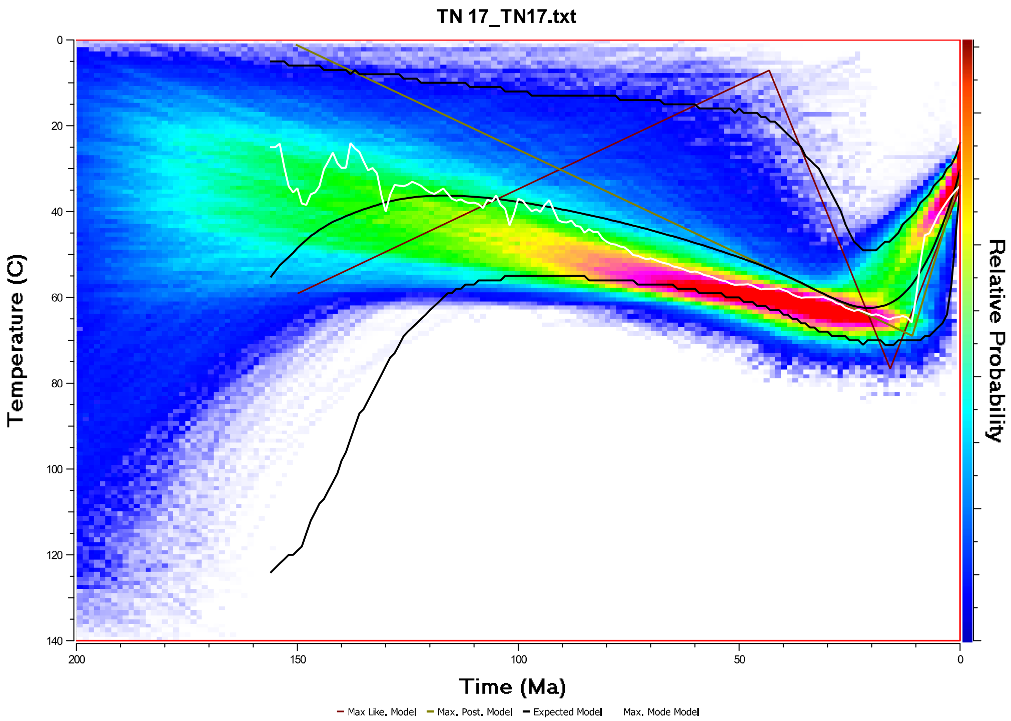
TN 17_TN17.txt
Temperature (C)
Time (Ma)
Relative Probability
Max Like. Model
Max. Post. Model
Expected Model
Max. Mode Model

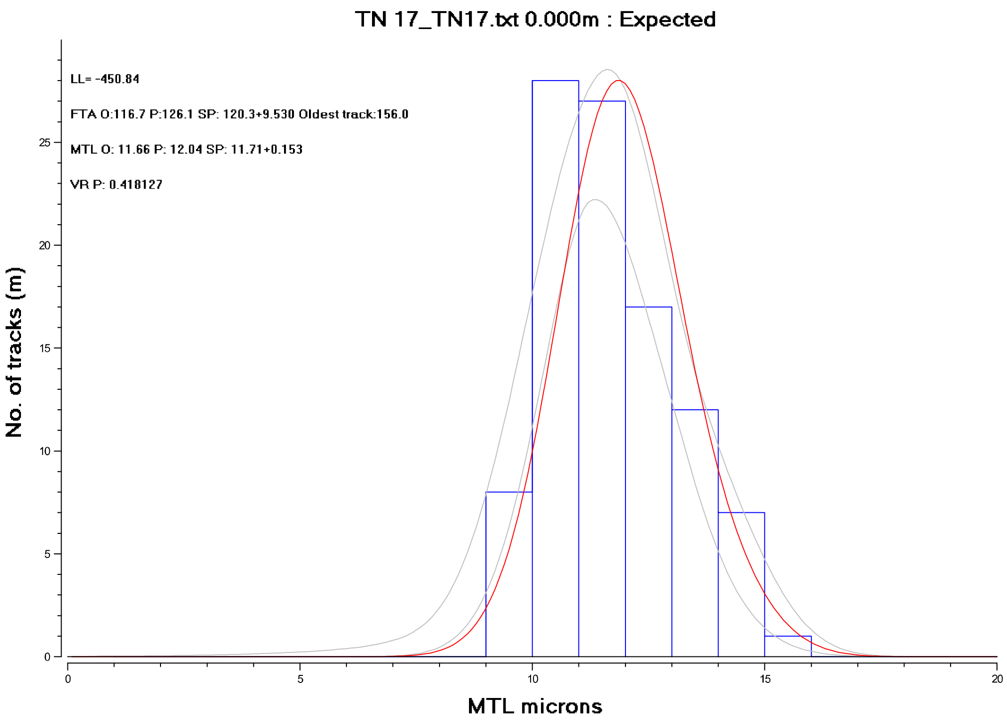
TN 17_TN17.txt 0.000m : Expected
LL= -450.84
FTA O:116.7 P:126.1 SP: 120.3+9.530 Oldest track:156.0
MTL O: 11.66 P: 12.04 SP: 11.71+0.153
VR P: 0.418127
No. of tracks (m)
MTL microns

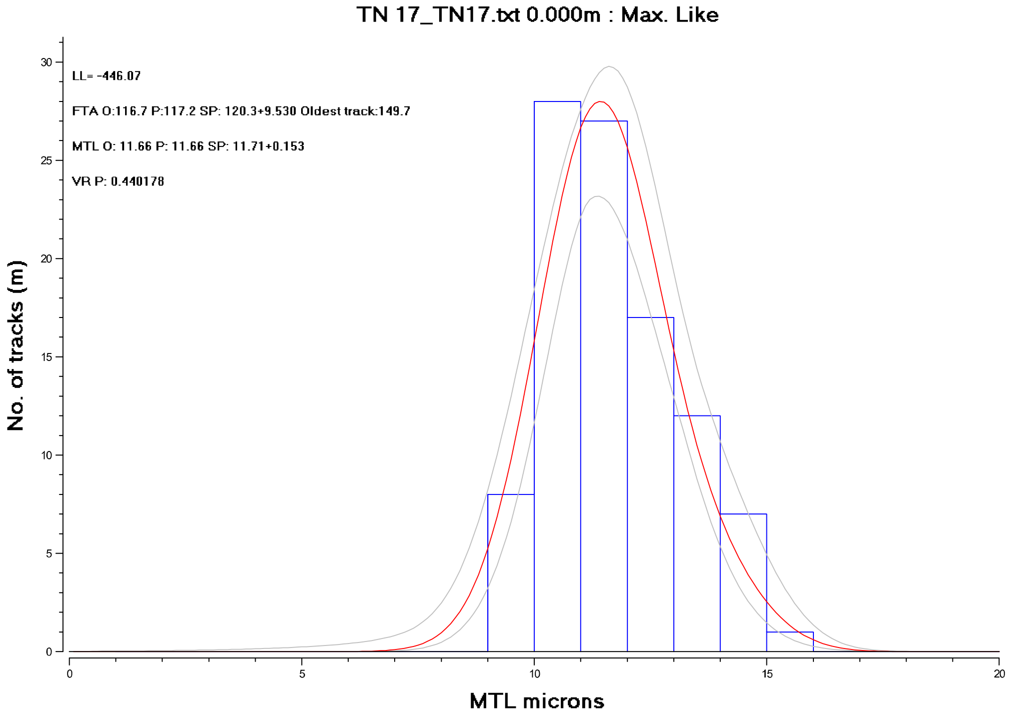
TN 17_TN17.txt 0.000m : Max. Like
LL= -446.07
FTA O:116.7 P:117.2 SP: 120.3+9.530 Oldest track:149.7
MTL O: 11.66 P: 11.66 SP: 11.71+0.153
VR P: 0.440178
No. of tracks (m)
MTL microns

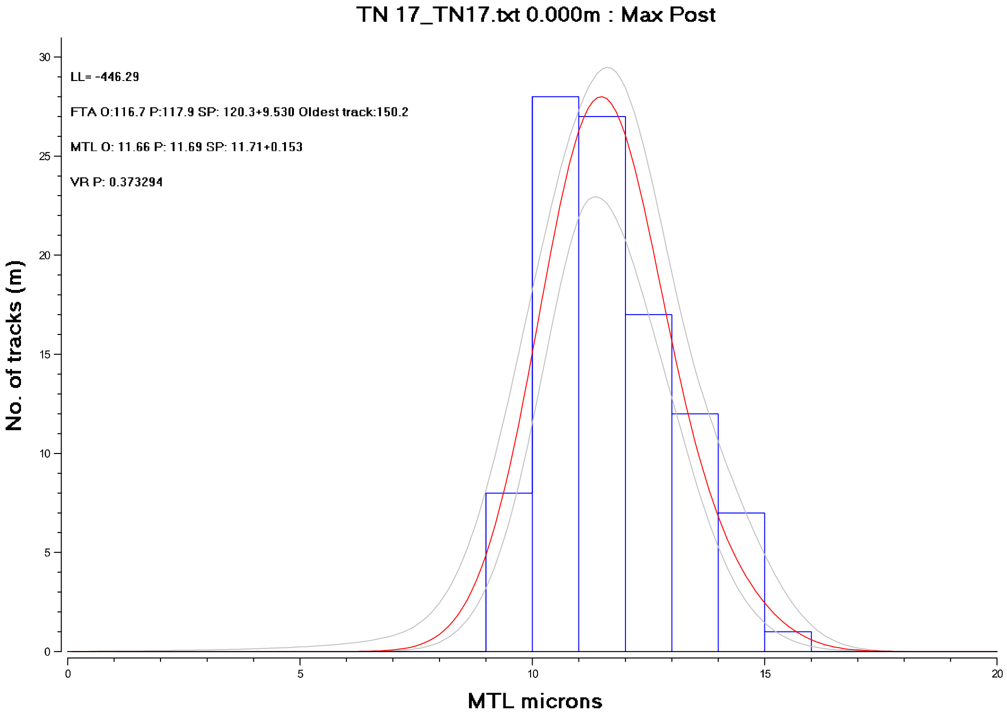
TN 17_TN17.txt 0.000m : Max Post
LL= -446.29
FTA O:116.7 P:117.9 SP: 120.3+9.530 Oldest track:150.2
MTL O: 11.66 P: 11.69 SP: 11.71+0.153
VR P: 0.373294
No. of tracks (m)
MTL microns

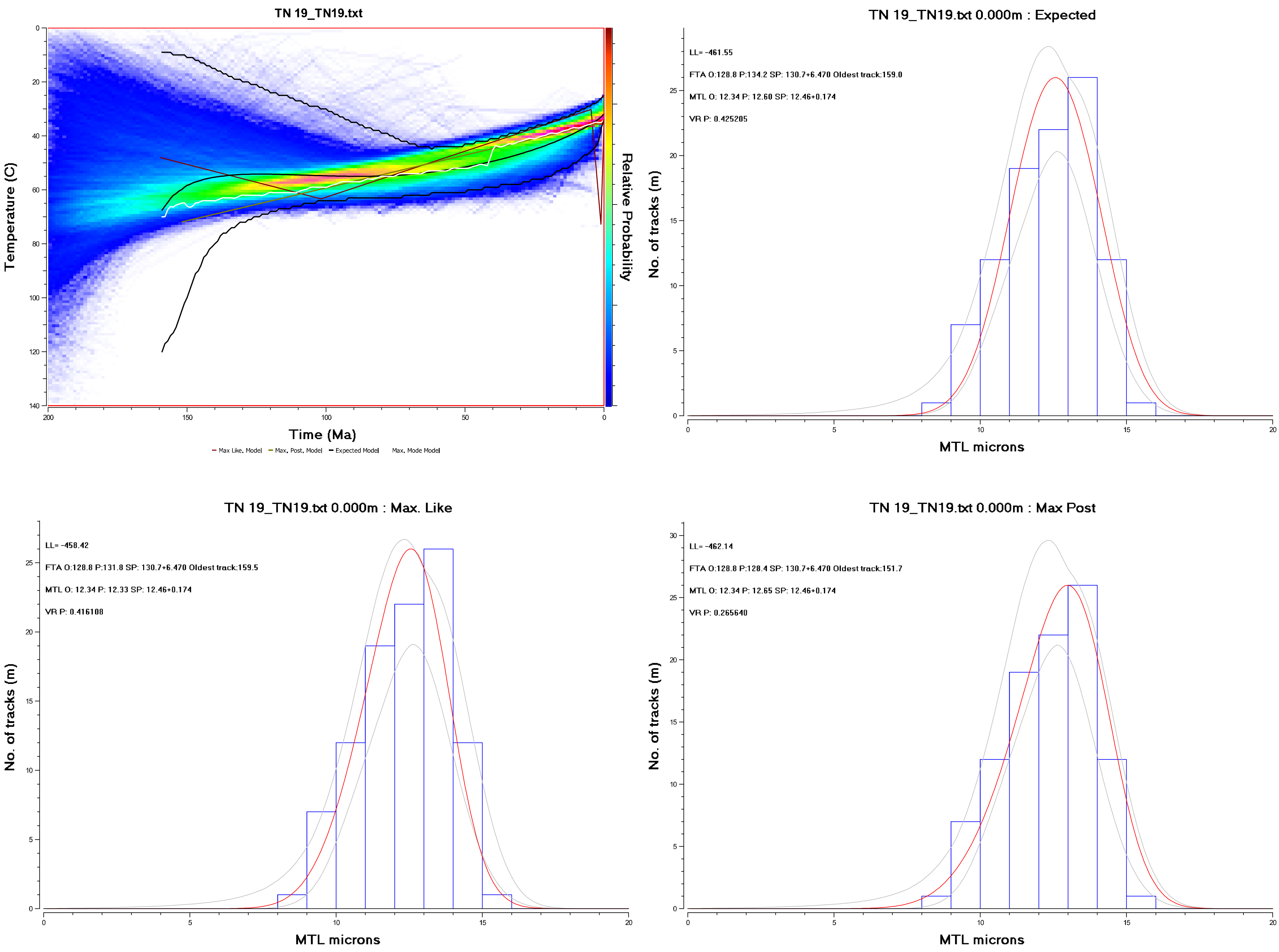
TN 19_TN19.txt
Temperature (C)
Time (Ma)
Relative Probability
Max Like. Model
Max. Post. Model
Expected Model
Max. Mode Model
TN 19_TN19.txt 0.000m : Expected
LL= -461.55
FTA O:128.8 P:134.2 SP: 130.7+6.470 Oldest track:159.0
MTL O: 12.34 P: 12.60 SP: 12.46+0.174
VR P: 0.425205
No. of tracks (m)
MTL microns
TN 19_TN19.txt 0.000m : Max. Like
LL= -458.42
FTA O:128.8 P:131.8 SP: 130.7+6.470 Oldest track:159.5
MTL O: 12.34 P: 12.33 SP: 12.46+0.174
VR P: 0.416108
No. of tracks (m)
MTL microns
TN 19_TN19.txt 0.000m : Max Post
LL= -462.14
FTA O:128.8 P:128.4 SP: 130.7+6.470 Oldest track:151.7
MTL O: 12.34 P: 12.65 SP: 12.46+0.174
VR P: 0.265640
No. of tracks (m)
MTL microns

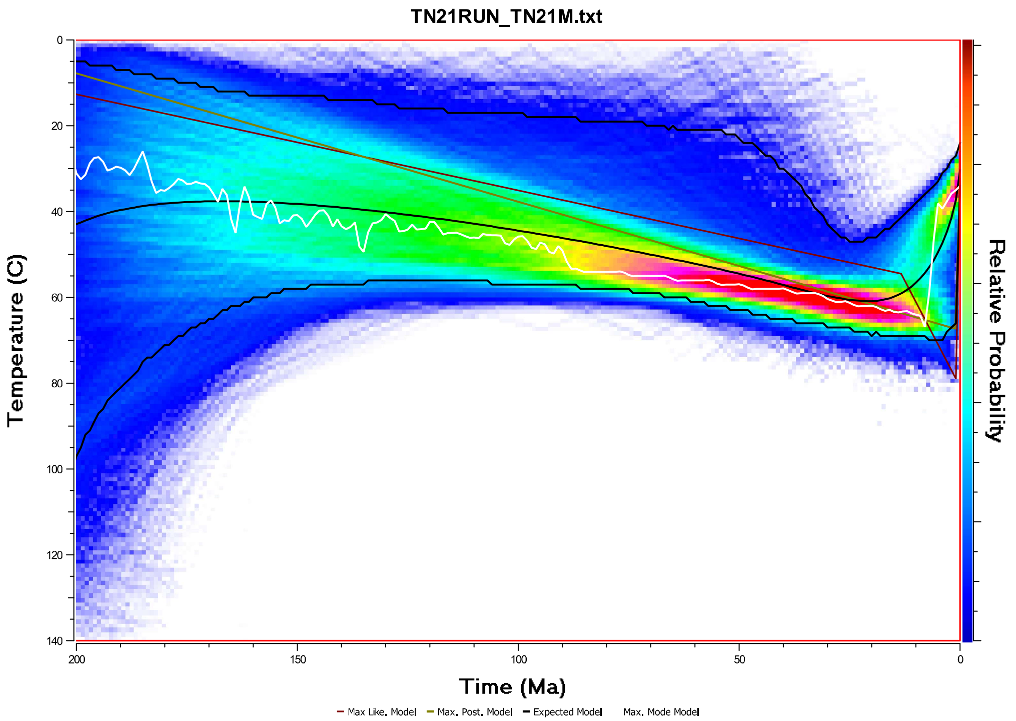
TN21RUN_TN21M.txt
Temperature (C)
Time (Ma)
Relative Probability
Max Like. Model
Max. Post. Model
Expected Model
Max. Mode Model

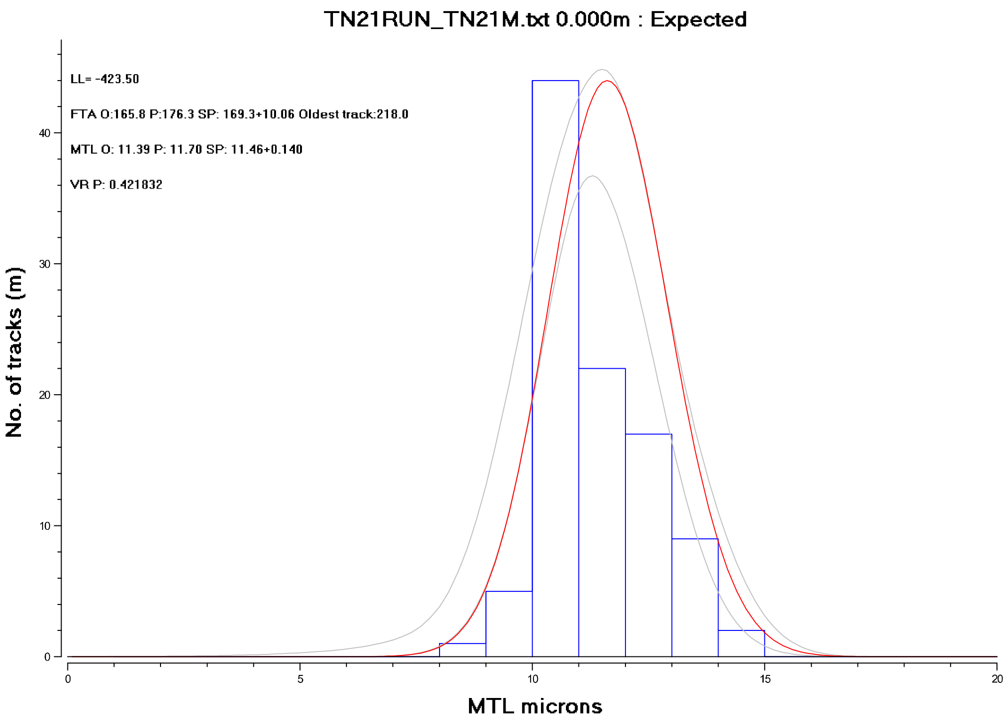
TN21RUN_TN21M.txt 0.000m : Expected
LL= -423.50
FTA O:165.8 P:176.3 SP: 169.3+10.06 Oldest track:218.0
MTL O: 11.39 P: 11.70 SP: 11.46+0.140
VR P: 0.421832
No. of tracks (m)
MTL microns

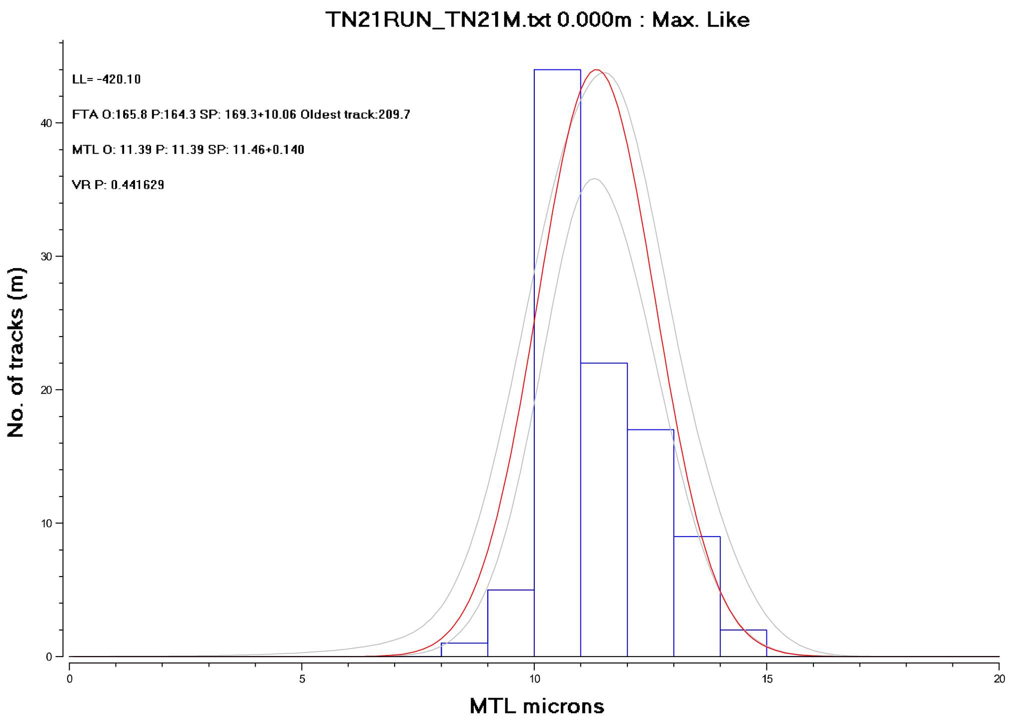
TN21RUN_TN21M.txt 0.000m : Max. Like
LL= -420.10
FTA O:165.8 P:164.3 SP: 169.3+10.06 Oldest track:209.7
MTL O: 11.39 P: 11.39 SP: 11.46+0.140
VR P: 0.441629
No. of tracks (m)
MTL microns

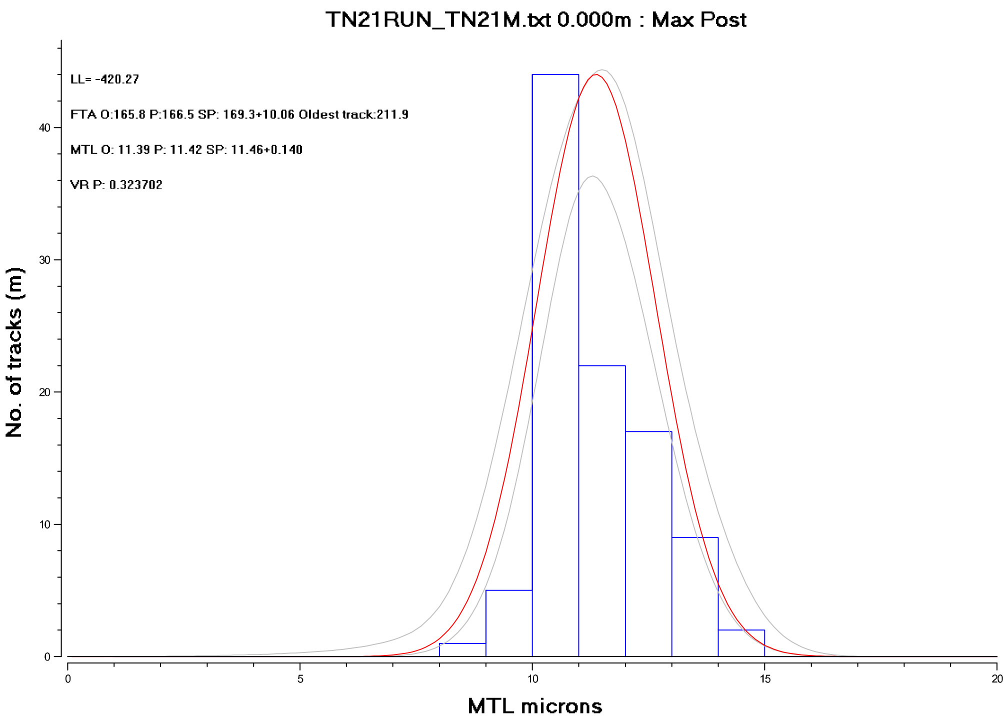
TN21RUN_TN21M.txt 0.000m : Max Post
LL= -420.27
FTA O:165.8 P:166.5 SP: 169.3+10.06 Oldest track:211.9
MTL O: 11.39 P: 11.42 SP: 11.46+0.140
VR P: 0.323702
No. of tracks (m)
MTL microns

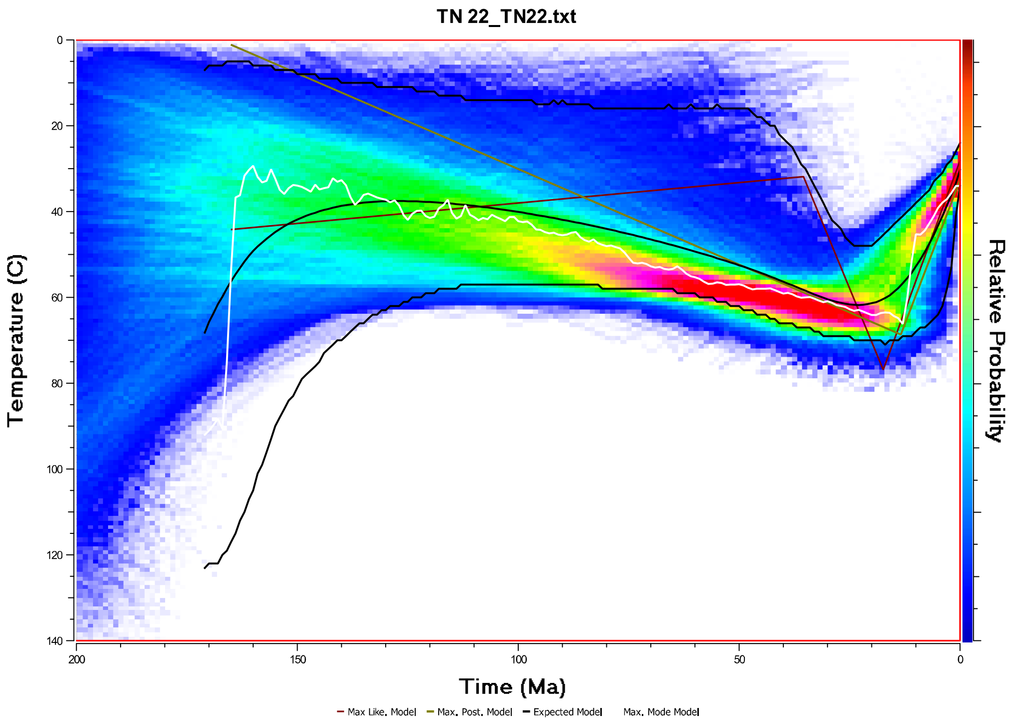
TN 22_TN22.txt
Temperature (C)
Time (Ma)
Relative Probability
Max Like. Model
Max. Post. Model
Expected Model
Max. Mode Model

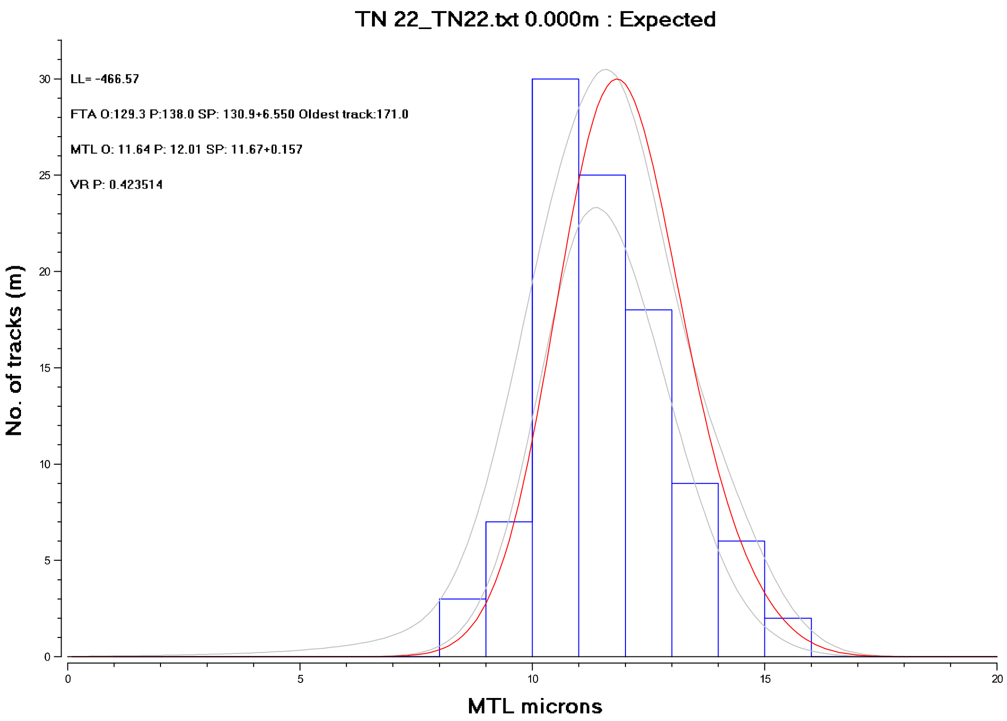
TN 22_TN22.txt 0.000m : Expected
LL= -466.57
FTA O:129.3 P:138.0 SP: 130.9+6.550 Oldest track:171.0
MTL O: 11.64 P: 12.01 SP: 11.67+0.157
VR P: 0.423514
No. of tracks (m)
MTL microns

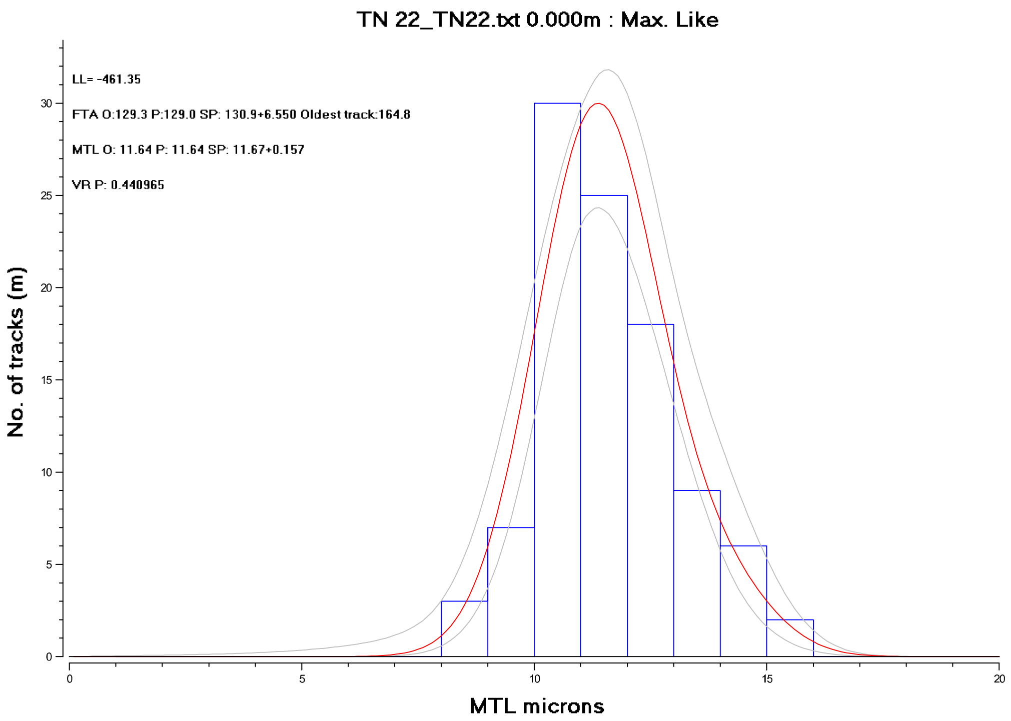
TN 22_TN22.txt 0.000m : Max. Like
LL= -461.35
FTA O:129.3 P:129.0 SP: 130.9+6.550 Oldest track:164.8
MTL O: 11.64 P: 11.64 SP: 11.67+0.157
VR P: 0.440965
No. of tracks (m)
MTL microns

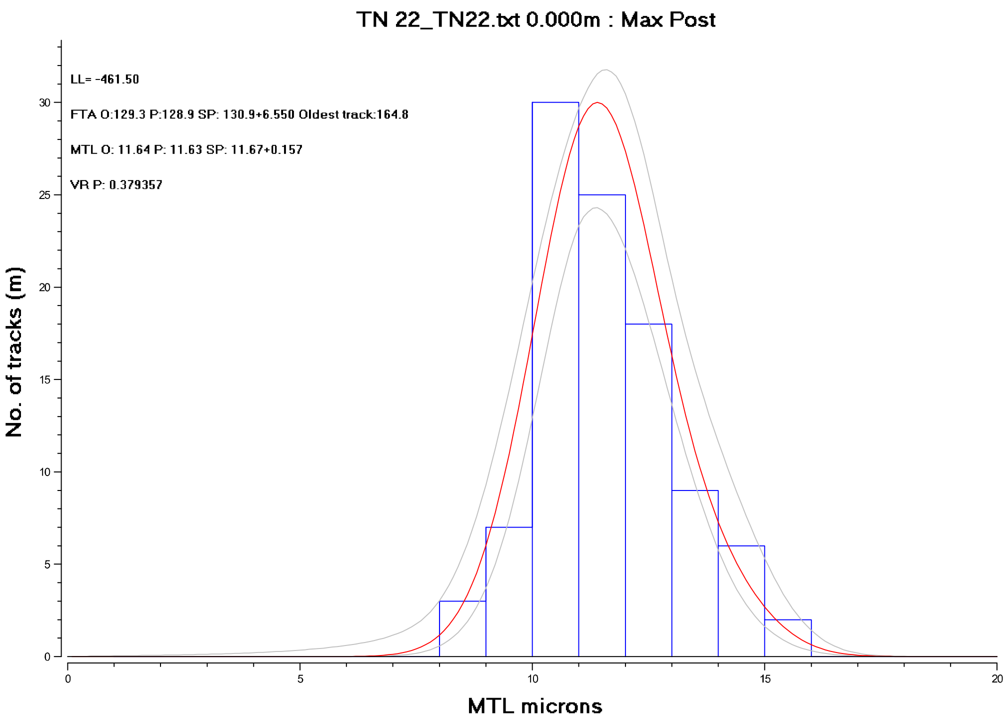
TN 22_TN22.txt 0.000m : Max Post
LL= -461.50
FTA O:129.3 P:128.9 SP: 130.9+6.550 Oldest track:164.8
MTL O: 11.64 P: 11.63 SP: 11.67+0.157
VR P: 0.379357
No. of tracks (m)
MTL microns

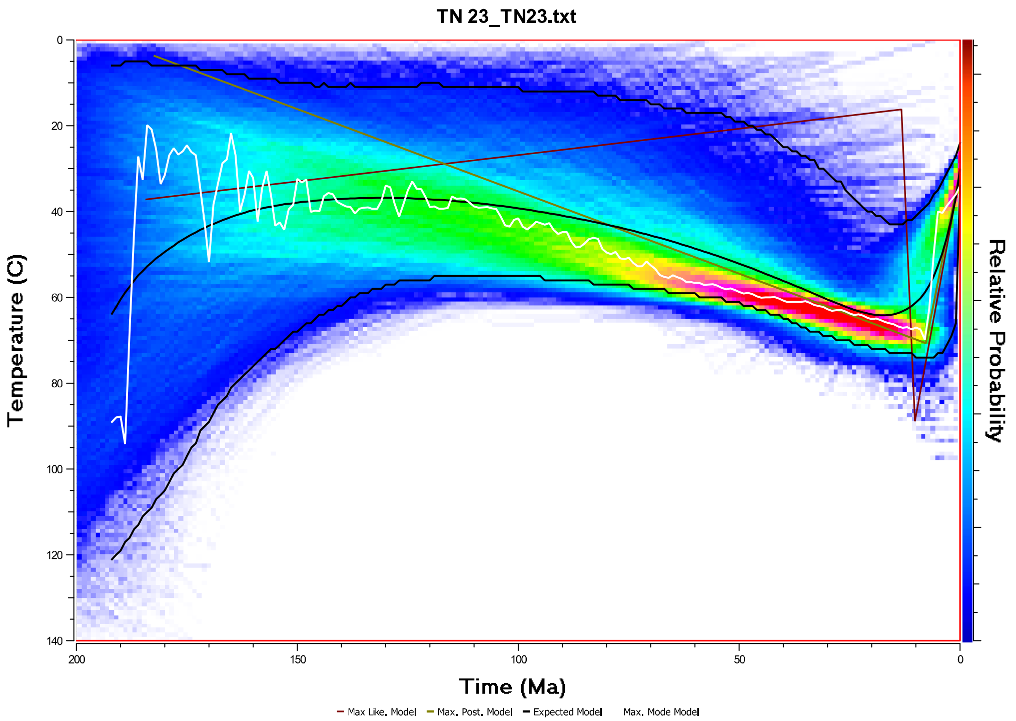
TN 23_TN23.txt
Temperature (C)
Time (Ma)
Relative Probability
Max Like. Model
Max. Post. Model
Expected Model
Max. Mode Model

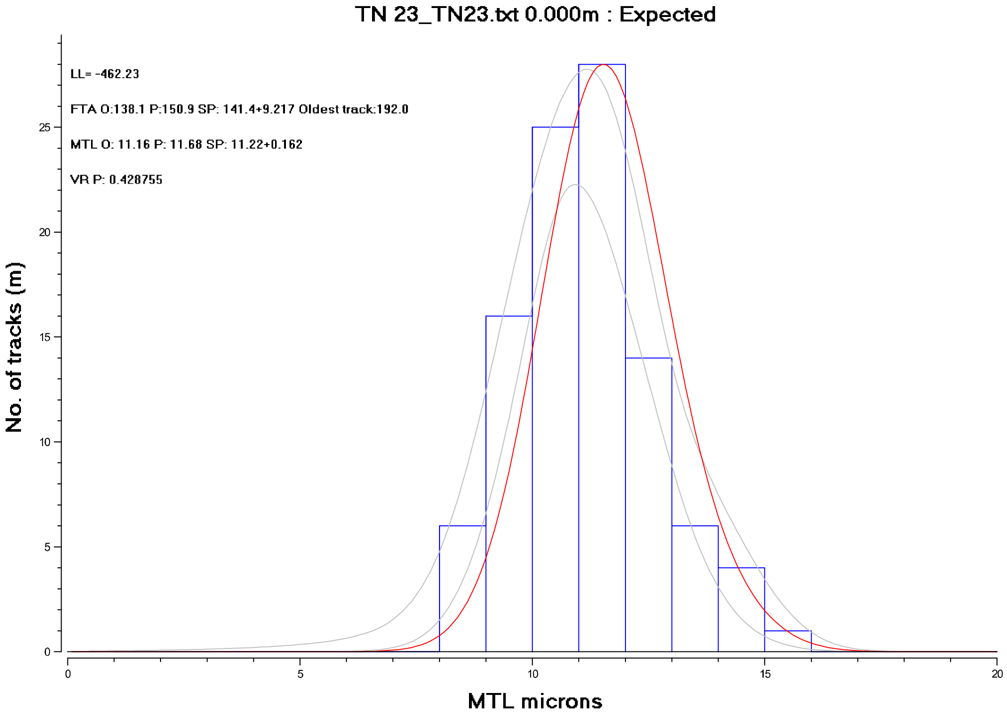
TN 23_TN23.txt 0.000m : Expected
LL= -462.23
FTA O:138.1 P:150.9 SP: 141.4+9.217 Oldest track:192.0
MTL O: 11.16 P: 11.68 SP: 11.22+0.162
VR P: 0.428755
No. of tracks (m)
MTL microns

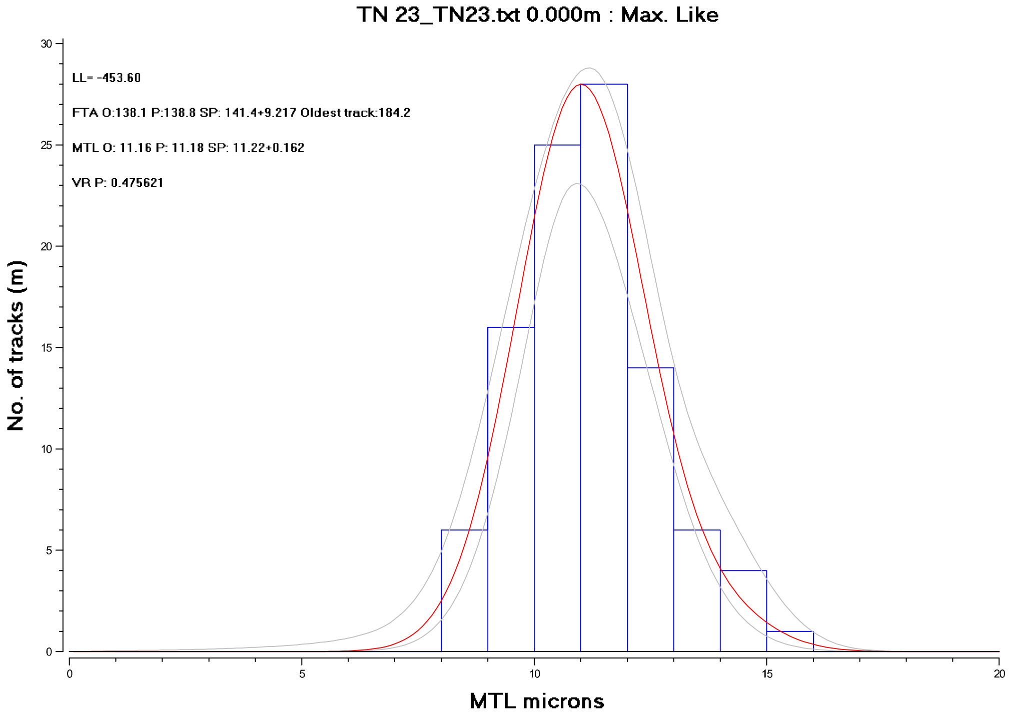
TN 23_TN23.txt 0.000m : Max. Like
LL= -453.60
FTA O:138.1 P:138.8 SP: 141.4+9.217 Oldest track:184.2
MTL O: 11.16 P: 11.18 SP: 11.22+0.162
VR P: 0.475621
No. of tracks (m)
MTL microns

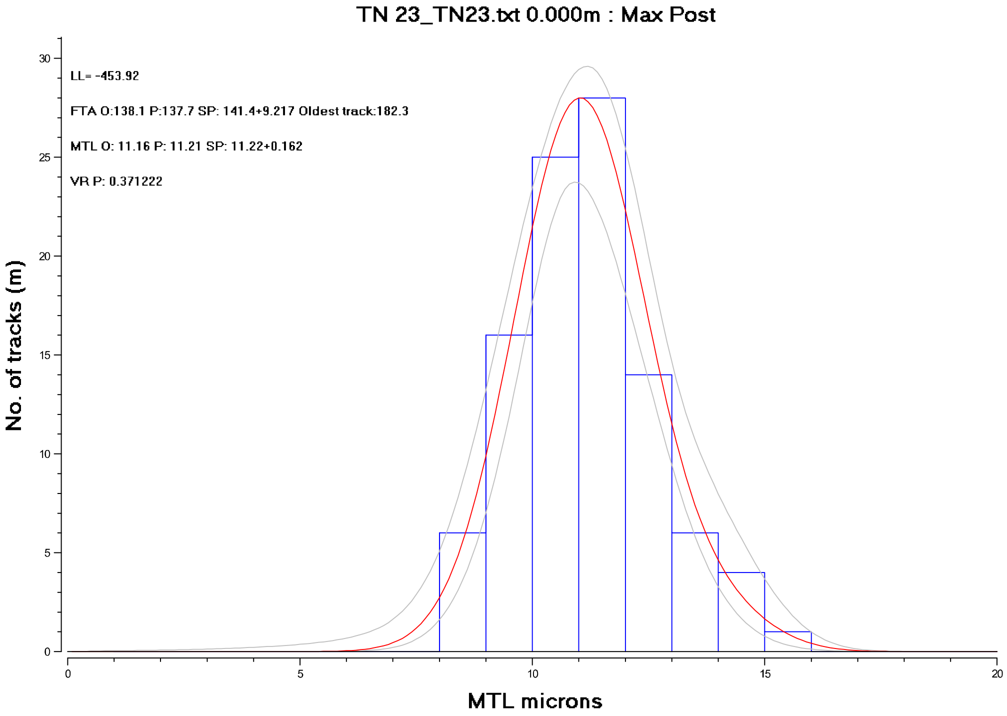
TN 23_TN23.txt 0.000m : Max Post
LL= -453.92
FTA O:138.1 P:137.7 SP: 141.4+9.217 Oldest track:182.3
MTL O: 11.16 P: 11.21 SP: 11.22+0.162
VR P: 0.371222
No. of tracks (m)
MTL microns

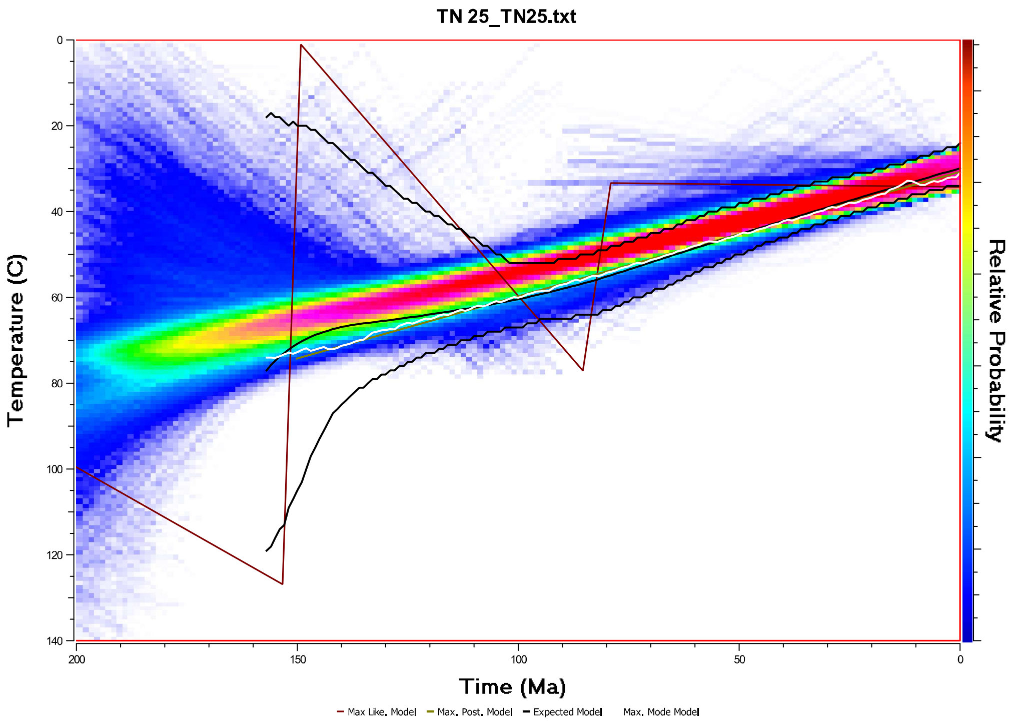
TN 25_TN25.txt
Temperature (C)
Time (Ma)
Relative Probability
Max Like. Model
Max. Post. Model
Expected Model
Max. Mode Model

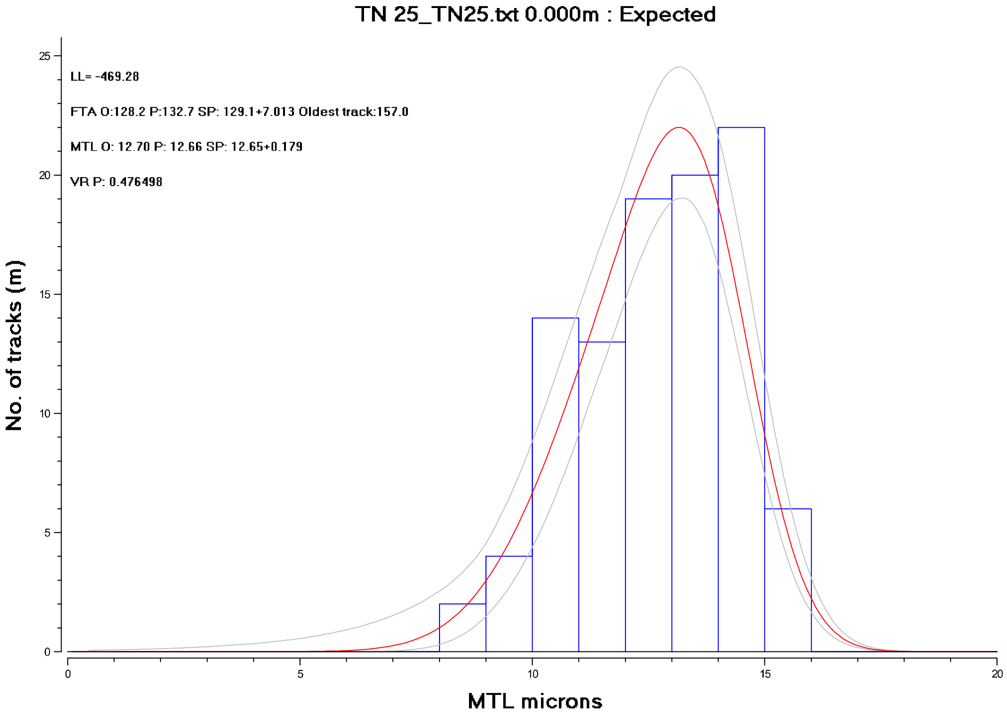
TN 25_TN25.txt 0.000m : Expected
LL= -469.28
FTA O:128.2 P:132.7 SP: 129.1+7.013 Oldest track:157.0
MTL O: 12.70 P: 12.66 SP: 12.65+0.179
VR P: 0.476498
No. of tracks (m)
MTL microns

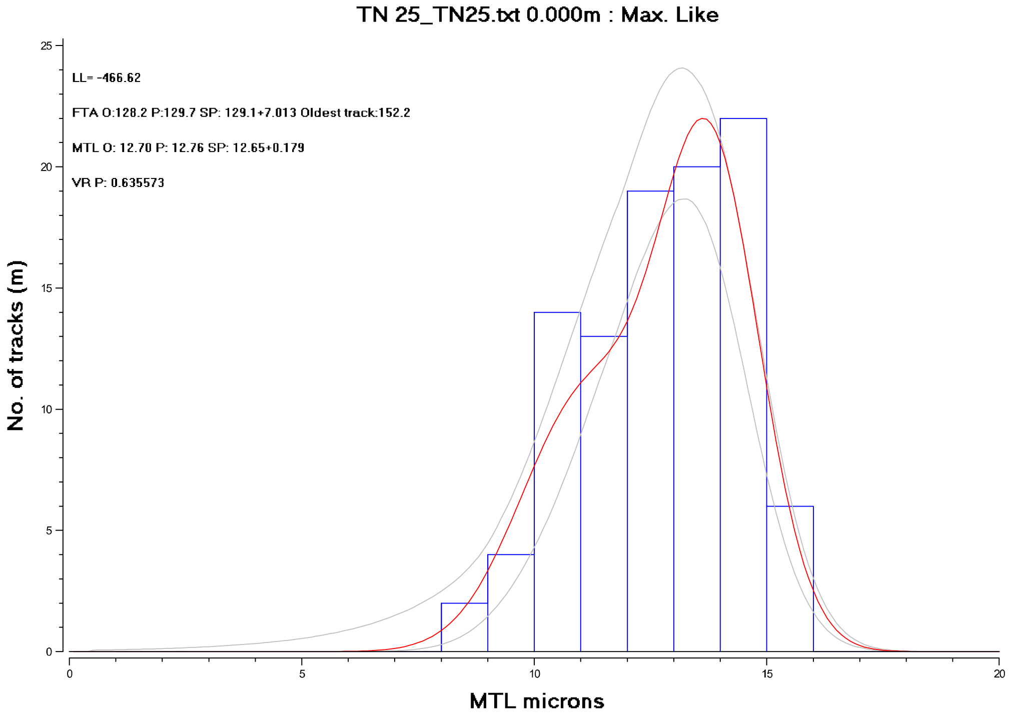
TN 25_TN25.txt 0.000m : Max. Like
LL= -466.62
FTA O:128.2 P:129.7 SP: 129.1+7.013 Oldest track:152.2
MTL O: 12.70 P: 12.76 SP: 12.65+0.179
VR P: 0.635573
No. of tracks (m)
MTL microns

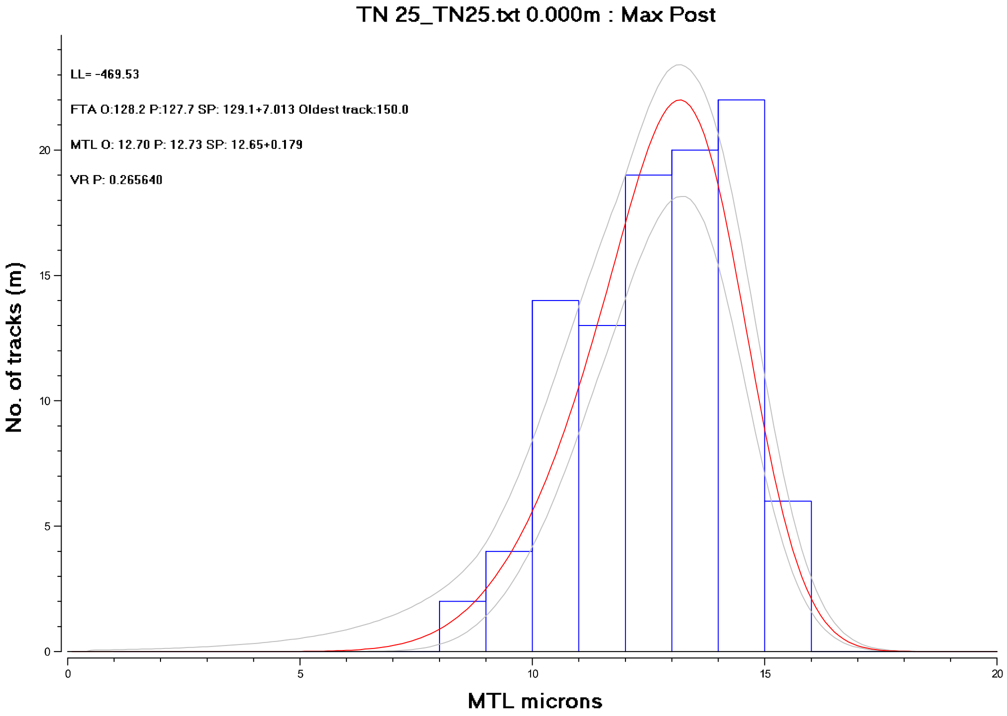
TN 25_TN25.txt 0.000m : Max Post
LL= -469.53
FTA O:128.2 P:127.7 SP: 129.1+7.013 Oldest track:150.0
MTL O: 12.70 P: 12.73 SP: 12.65+0.179
VR P: 0.265640
No. of tracks (m)
MTL microns

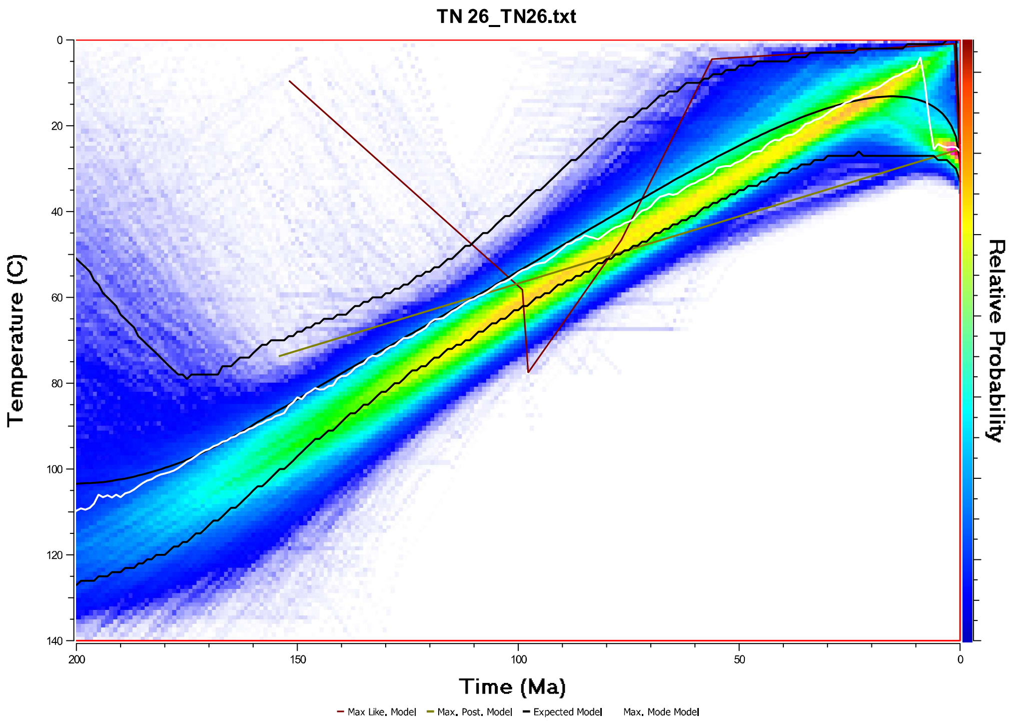
TN 26_TN26.txt
Temperature (C)
Time (Ma)
Relative Probability
Max Like. Model
Max. Post. Model
Expected Model
Max. Mode Model

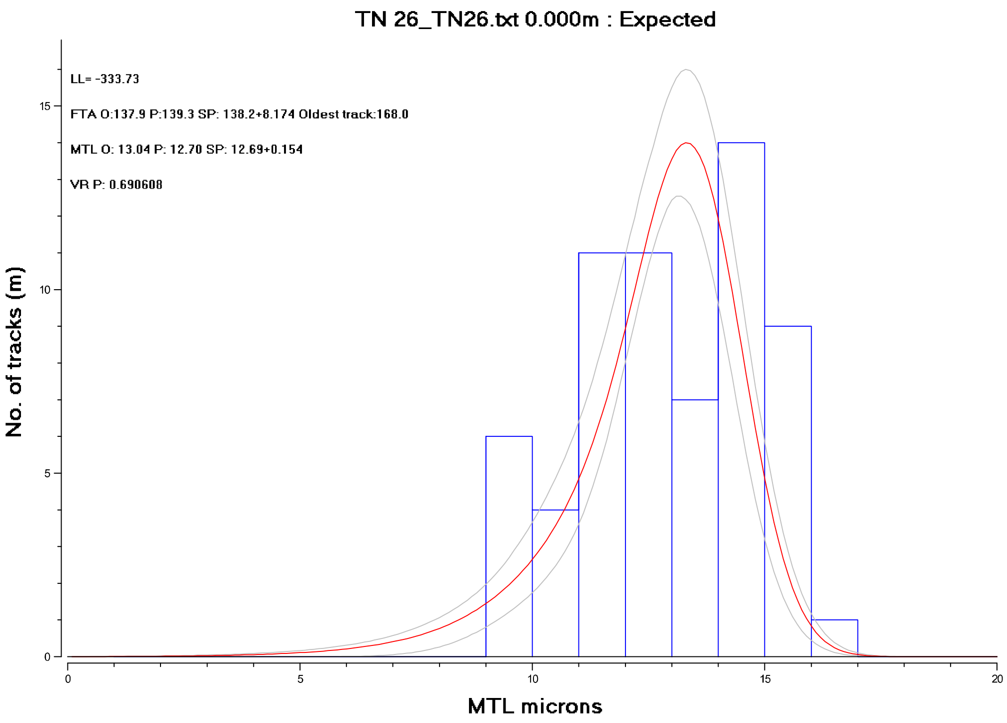
TN 26_TN26.txt 0.000m : Expected
LL= -333.73
FTA O:137.9 P:139.3 SP: 138.2+8.174 Oldest track:168.0
MTL O: 13.04 P: 12.70 SP: 12.69+0.154
VR P: 0.690608
No. of tracks (m)
MTL microns

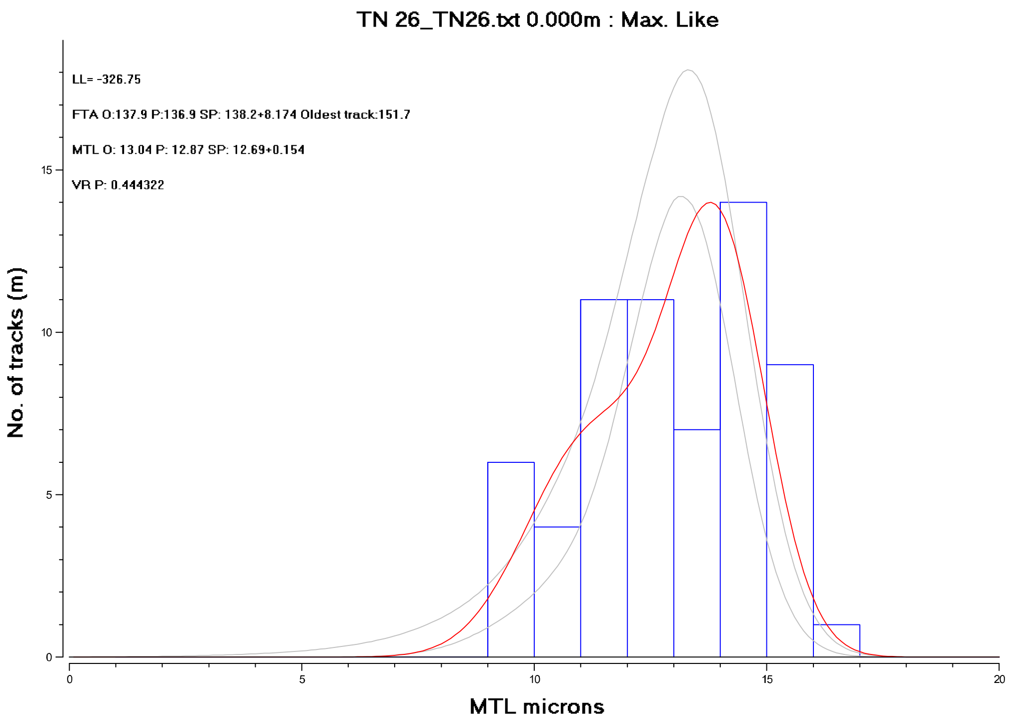
TN 26_TN26.txt 0.000m : Max. Like
LL= -326.75
FTA O:137.9 P:136.9 SP: 138.2+8.174 Oldest track:151.7
MTL O: 13.04 P: 12.87 SP: 12.69+0.154
VR P: 0.444322
No. of tracks (m)
MTL microns

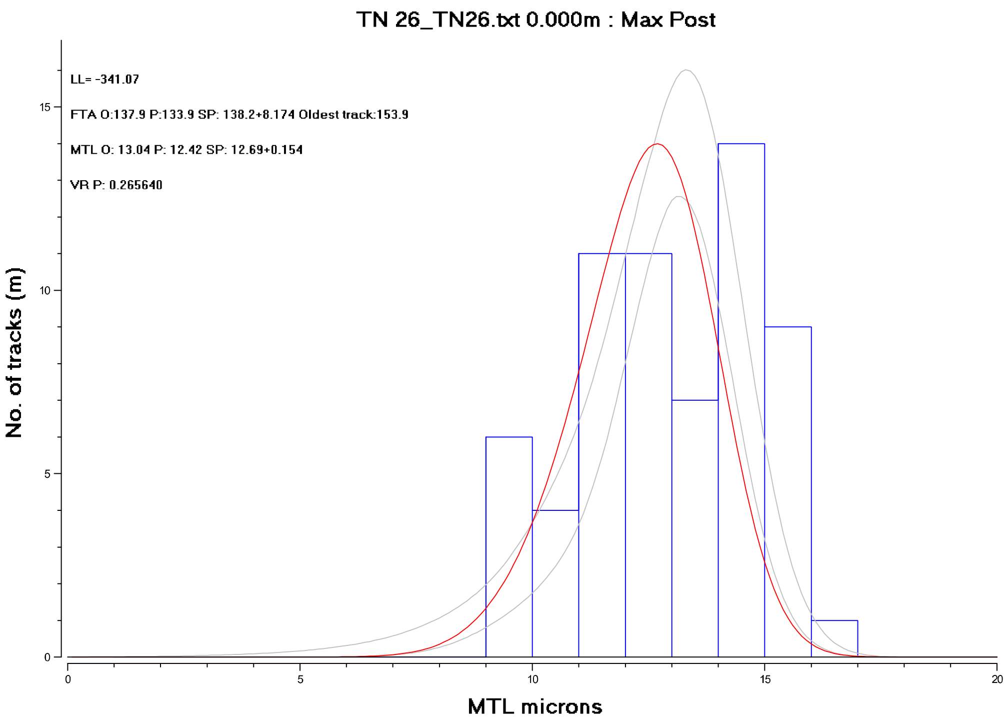
TN 26_TN26.txt 0.000m : Max Post
LL= -341.07
FTA O:137.9 P:133.9 SP: 138.2+8.174 Oldest track:153.9
MTL O: 13.04 P: 12.42 SP: 12.69+0.154
VR P: 0.265640
No. of tracks (m)
MTL microns

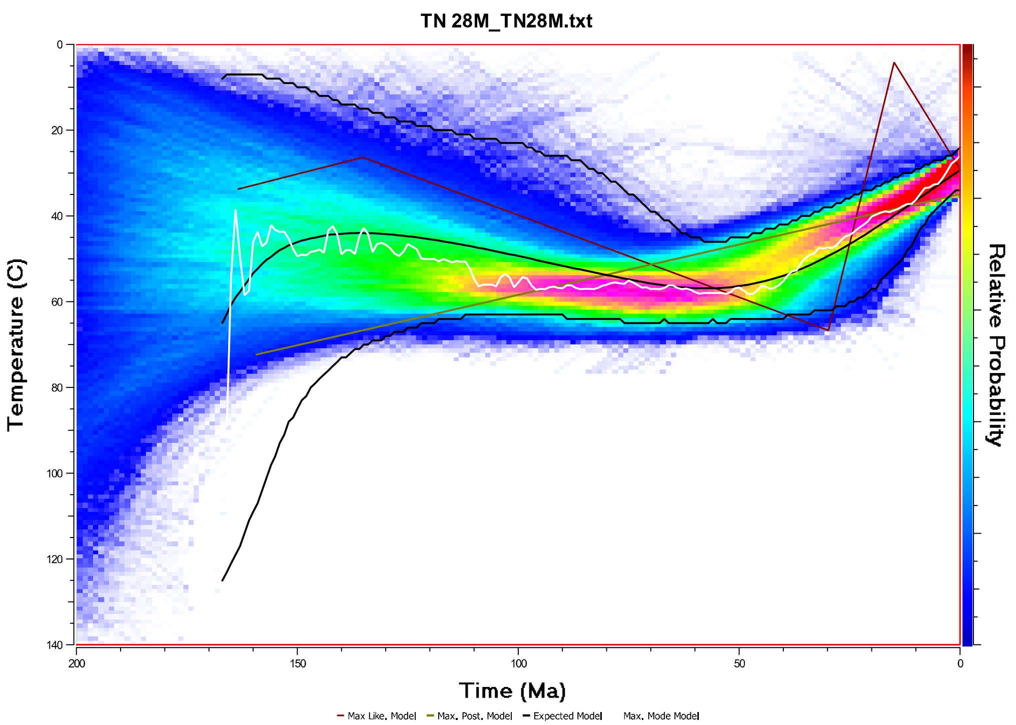
TN 28M_TN28M.txt
Temperature (C)
Time (Ma)
Relative Probability
Max Like. Model
Max. Post. Model
Expected Model
Max. Mode Model

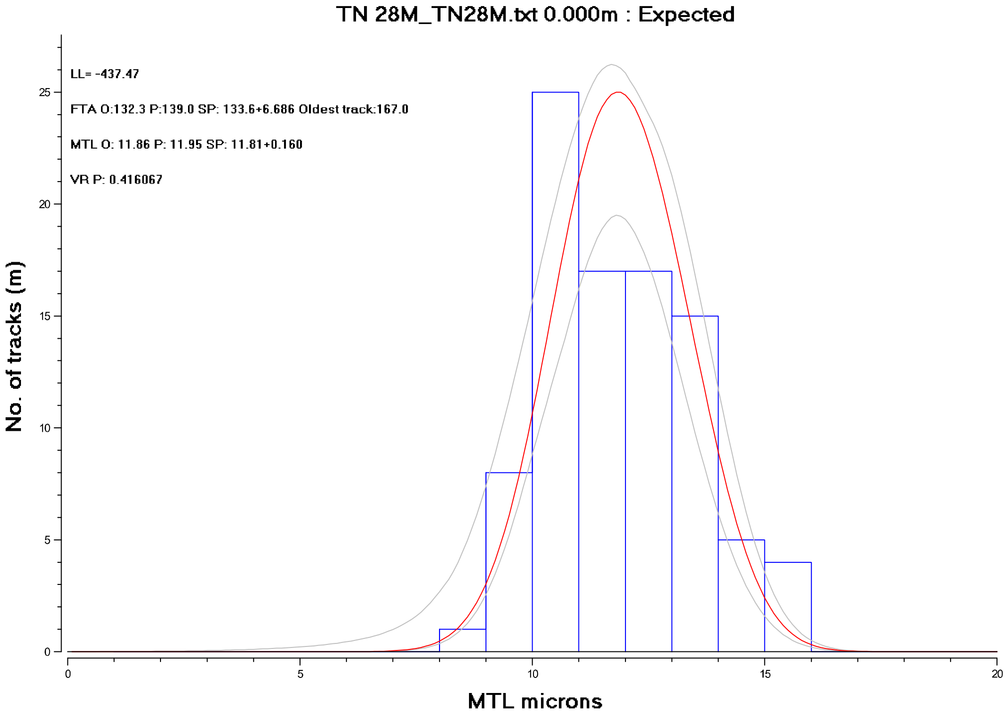
TN 28M_TN28M.txt 0.000m : Expected
LL= -437.47
FTA O:132.3 P:139.0 SP: 133.6+6.686 Oldest track:167.0
MTL O: 11.86 P: 11.95 SP: 11.81+0.160
VR P: 0.416067
No. of tracks (m)
MTL microns

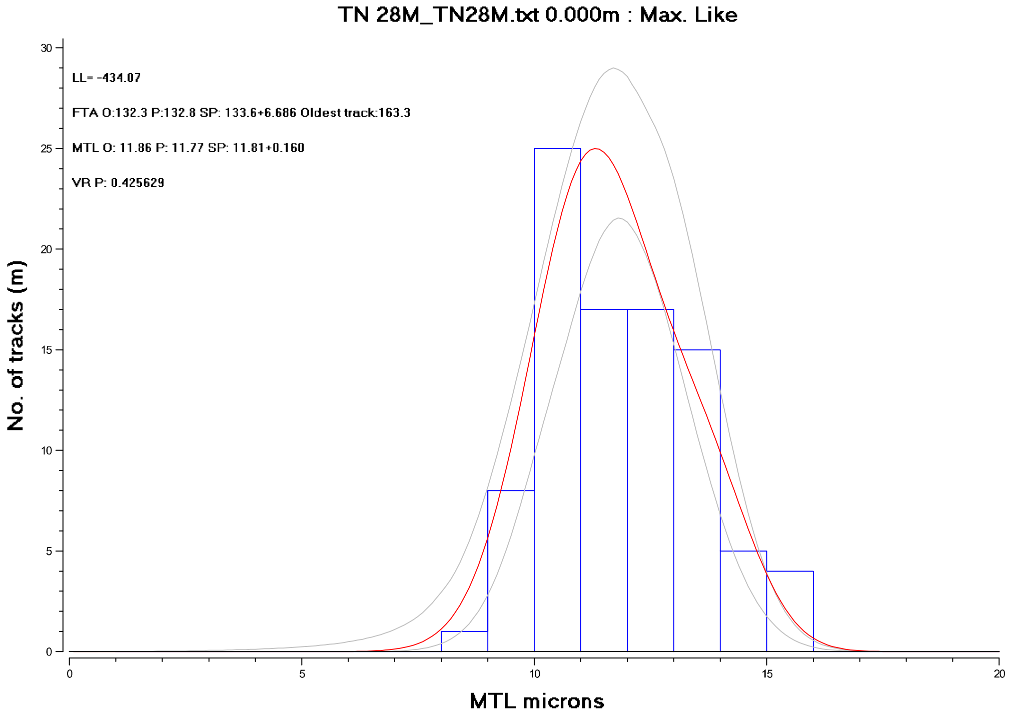
TN 28M_TN28M.txt 0.000m : Max. Like
LL= -434.07
FTA O:132.3 P:132.8 SP: 133.6+6.686 Oldest track:163.3
MTL O: 11.86 P: 11.77 SP: 11.81+0.160
VR P: 0.425629
No. of tracks (m)
MTL microns

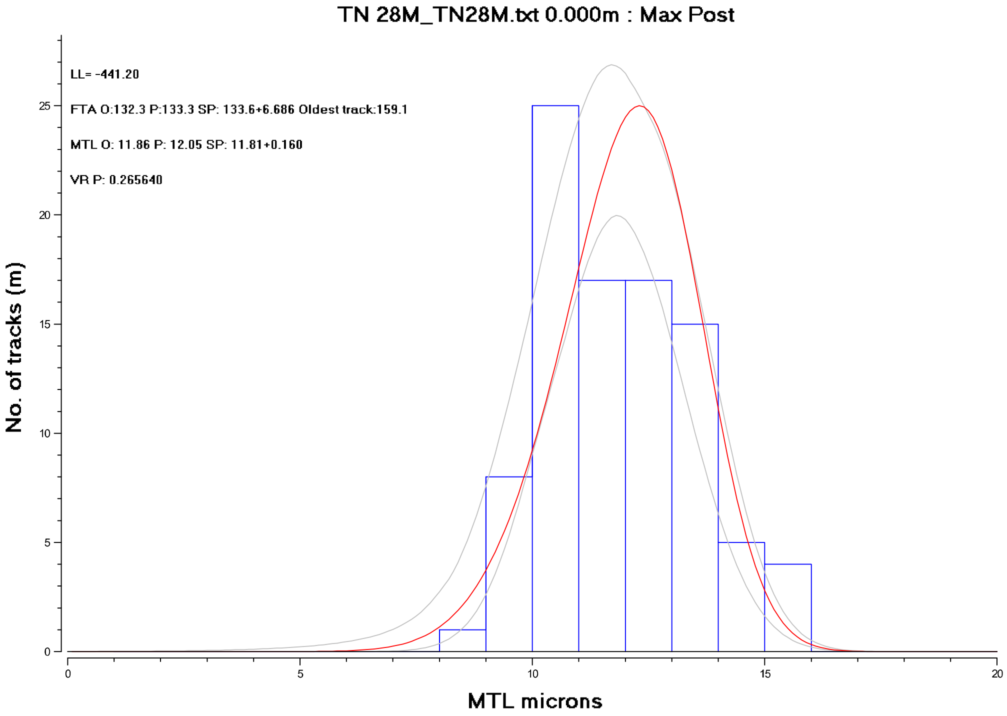
TN 28M_TN28M.txt 0.000m : Max Post
LL= -441.20
FTA O:132.3 P:133.3 SP: 133.6+6.686 Oldest track:159.1
MTL O: 11.86 P: 12.05 SP: 11.81+0.160
VR P: 0.265640
No. of tracks (m)
MTL microns

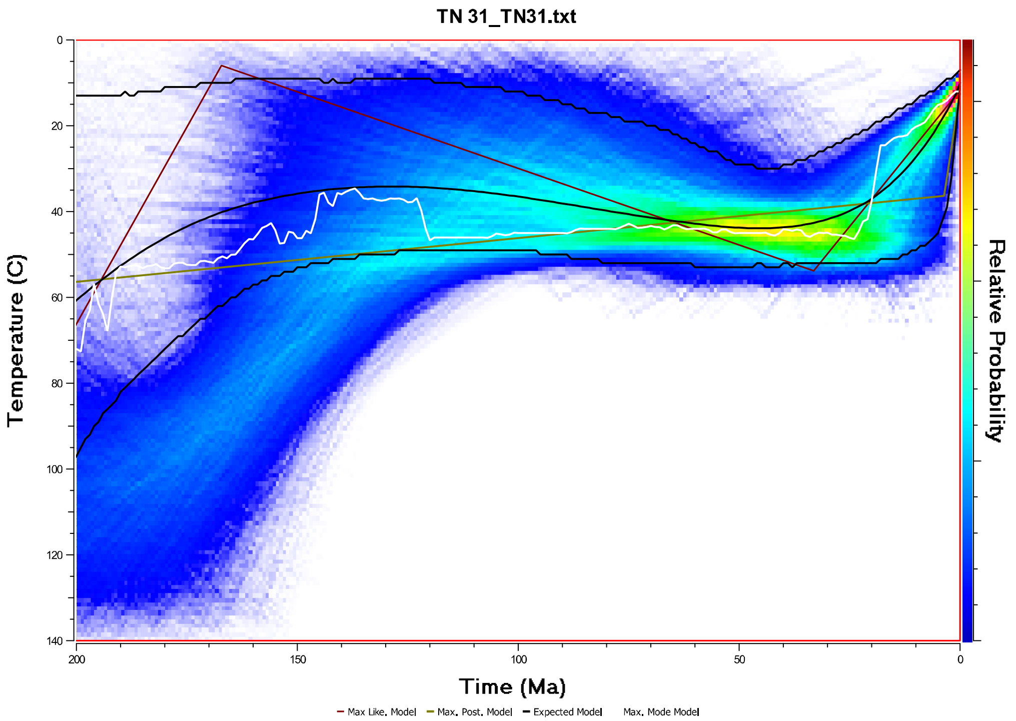
TN 31_TN31.txt
Temperature (C)
Time (Ma)
Relative Probability
Max Like. Model
Max. Post. Model
Expected Model
Max. Mode Model

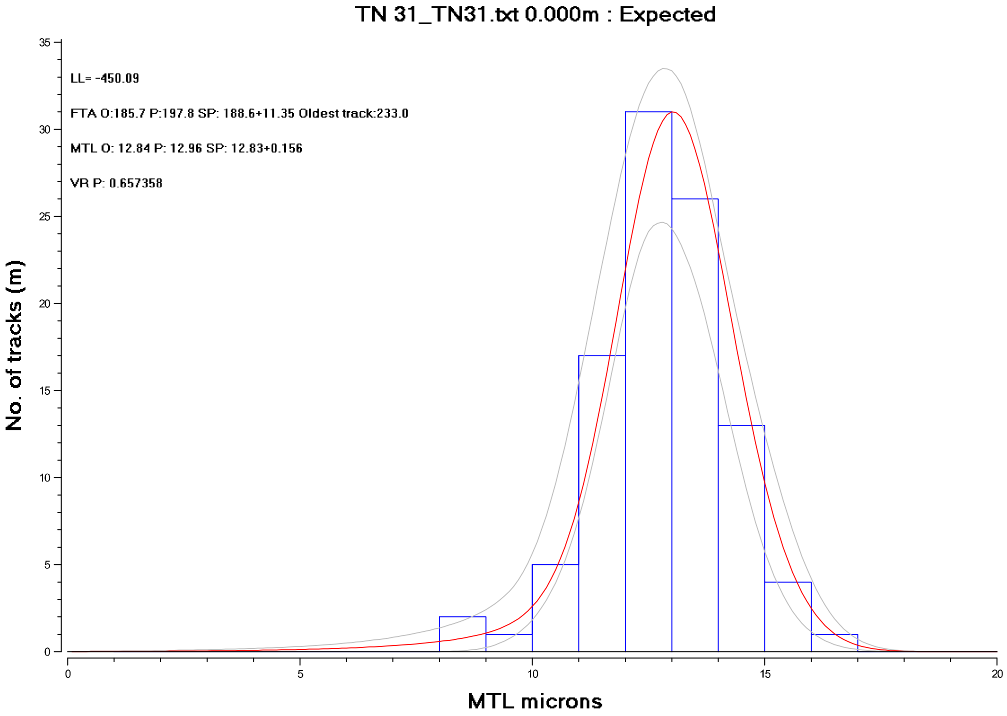
TN 31_TN31.txt 0.000m : Expected
LL= -450.09
FTA O:185.7 P:197.8 SP: 188.6+11.35 Oldest track:233.0
MTL O: 12.84 P: 12.96 SP: 12.83+0.156
VR P: 0.657358
No. of tracks (m)
MTL microns

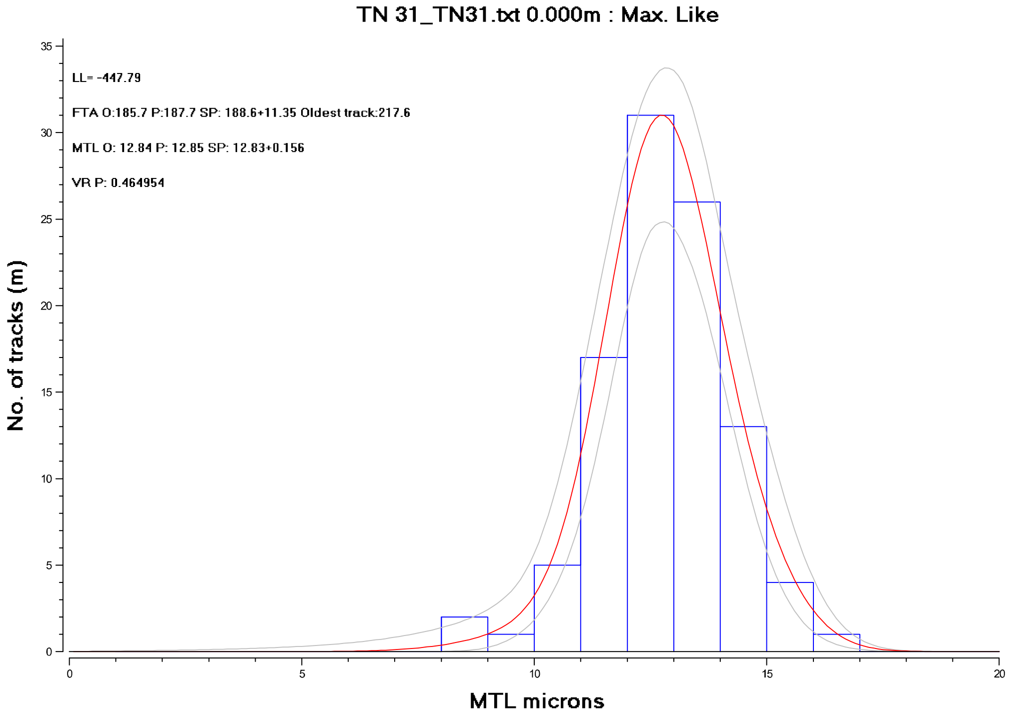
TN 31_TN31.txt 0.000m : Max. Like
LL= -447.79
FTA O:185.7 P:187.7 SP: 188.6+11.35 Oldest track:217.6
MTL O: 12.84 P: 12.85 SP: 12.83+0.156
VR P: 0.464954
No. of tracks (m)
MTL microns

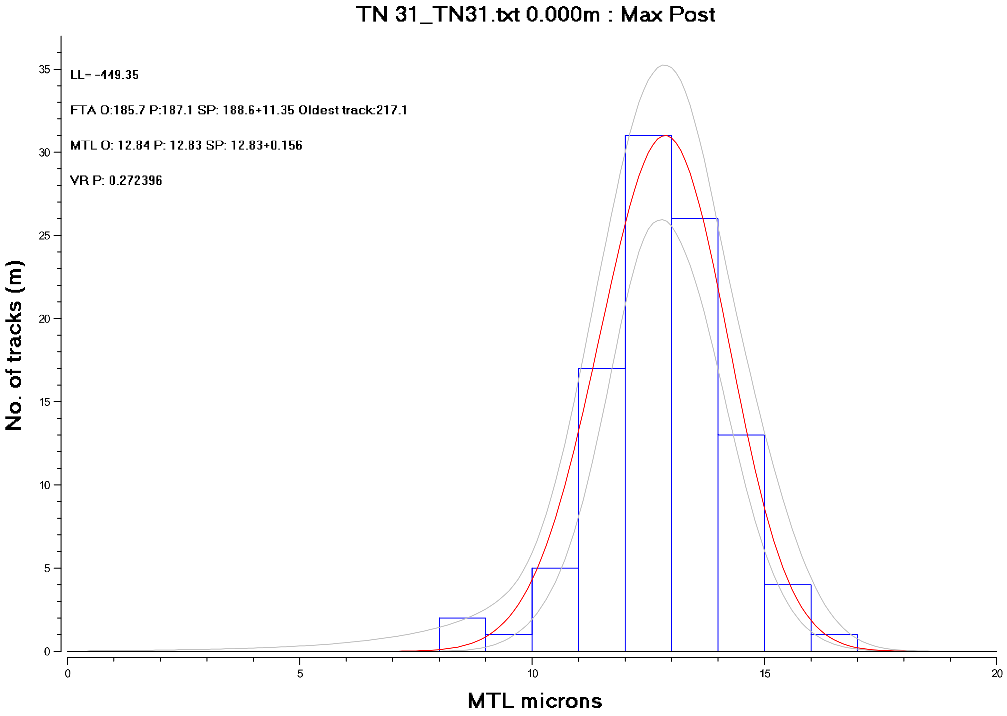
TN 31_TN31.txt 0.000m : Max Post
LL= -449.35
FTA O:185.7 P:187.1 SP: 188.6+11.35 Oldest track:217.1
MTL O: 12.84 P: 12.83 SP: 12.83+0.156
VR P: 0.272396
No. of tracks (m)
MTL microns

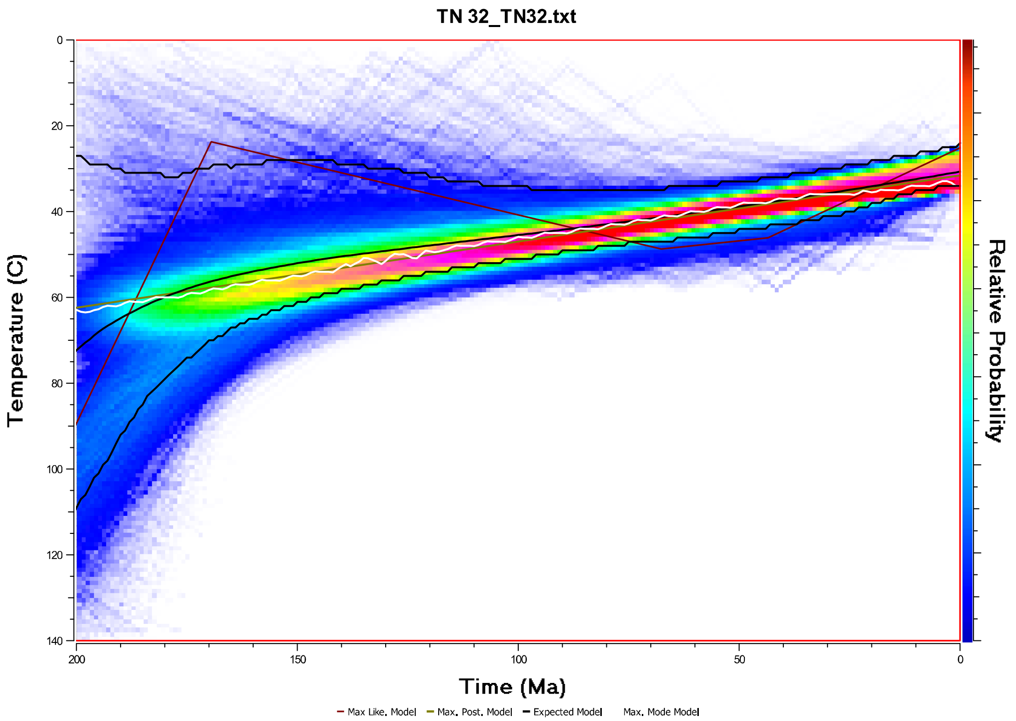

TN 32_TN32.txt
Temperature (C)
Time (Ma)
Relative Probability
Max Like. Model
Max. Post. Model
Expected Model
Max. Mode Model

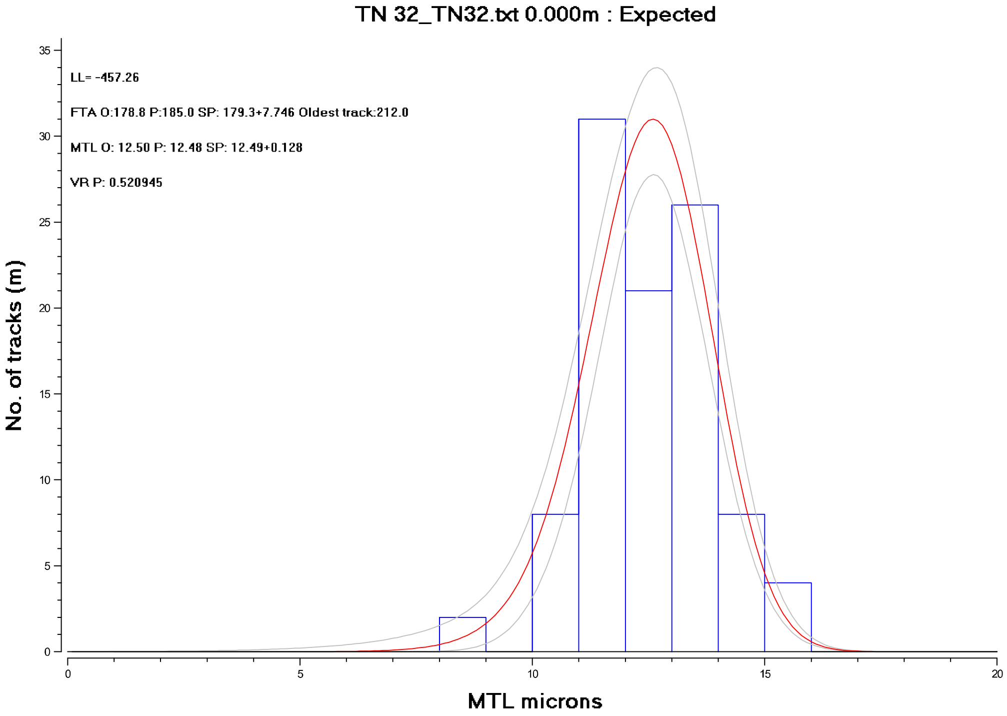

TN 32_TN32.txt 0.000m : Expected
LL= -457.26
FTA O:178.8 P:185.0 SP: 179.3+7.746 Oldest track:212.0
MTL O: 12.50 P: 12.48 SP: 12.49+0.128
VR P: 0.520945
No. of tracks (m)
MTL microns

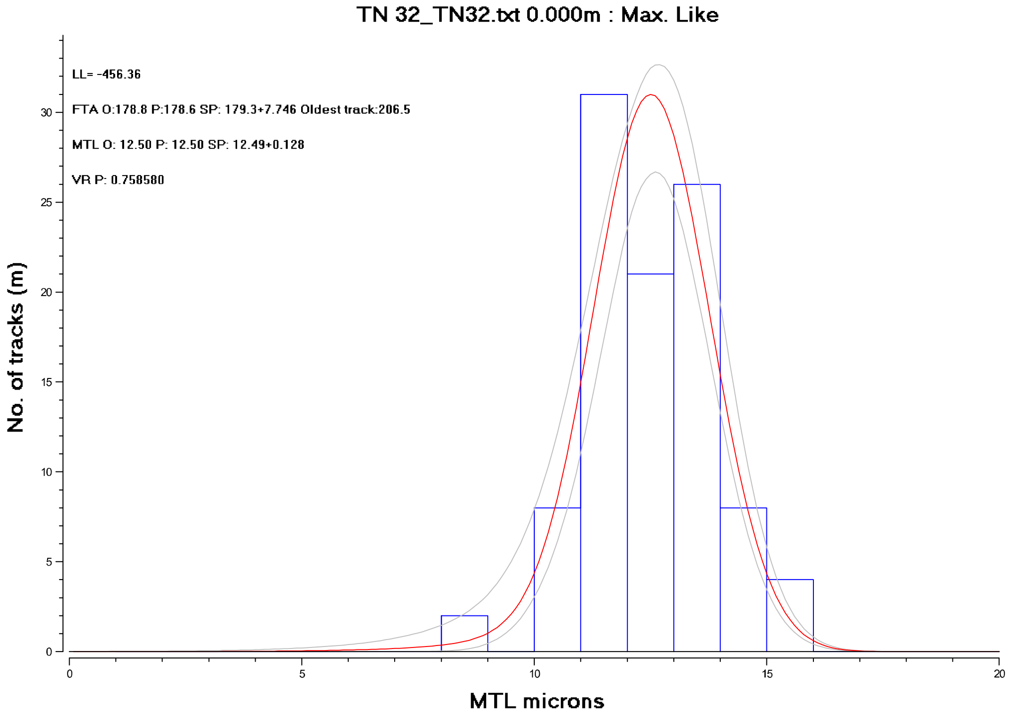

TN 32_TN32.txt 0.000m : Max. Like
LL= -456.36
FTA O:178.8 P:178.6 SP: 179.3+7.746 Oldest track:206.5
MTL O: 12.50 P: 12.50 SP: 12.49+0.128
VR P: 0.758580
No. of tracks (m)
MTL microns

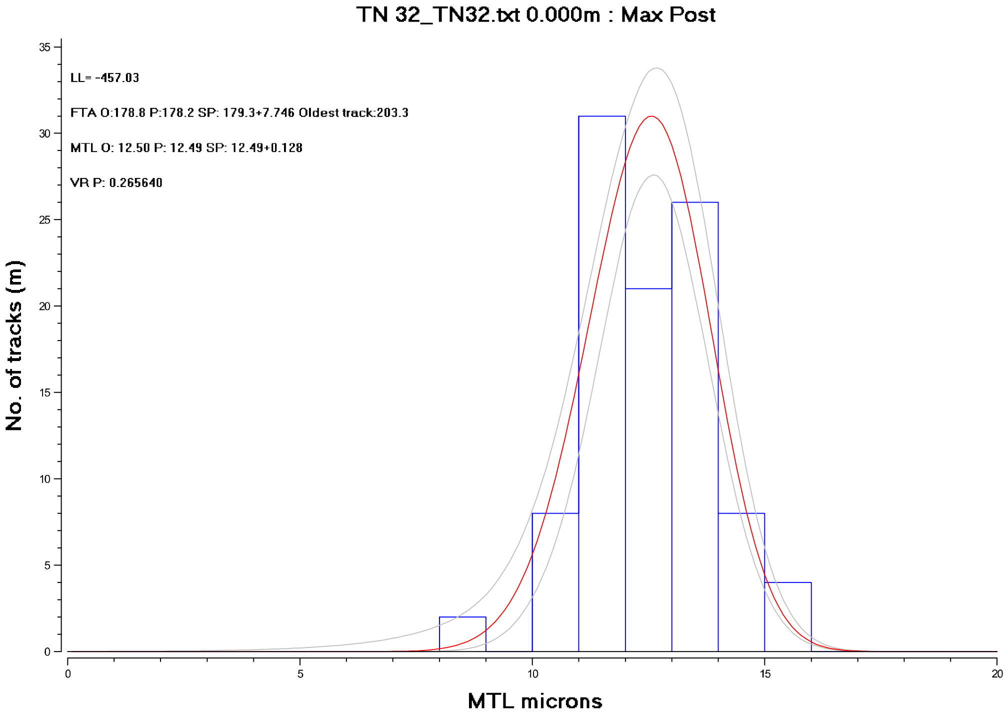

TN 32_TN32.txt 0.000m : Max Post
LL= -457.03
FTA O:178.8 P:178.2 SP: 179.3+7.746 Oldest track:203.3
MTL O: 12.50 P: 12.49 SP: 12.49+0.128
VR P: 0.265640
No. of tracks (m)
MTL microns

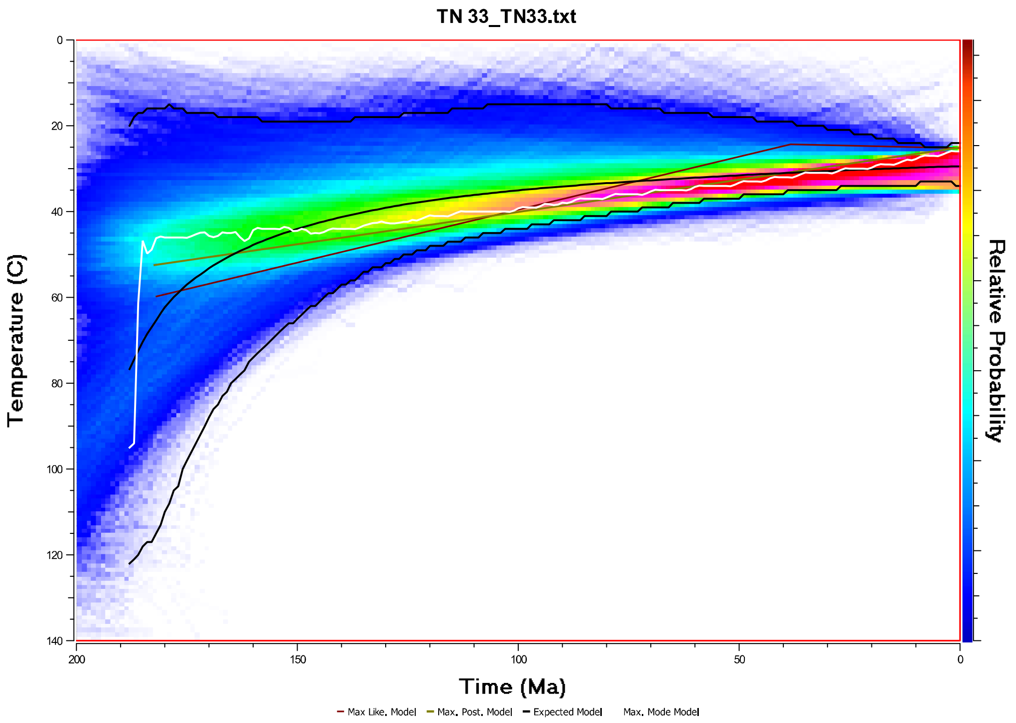
TN 33_TN33.txt
Temperature (C)
Time (Ma)
Relative Probability
Max Like. Model
Max. Post. Model
Expected Model
Max. Mode Model

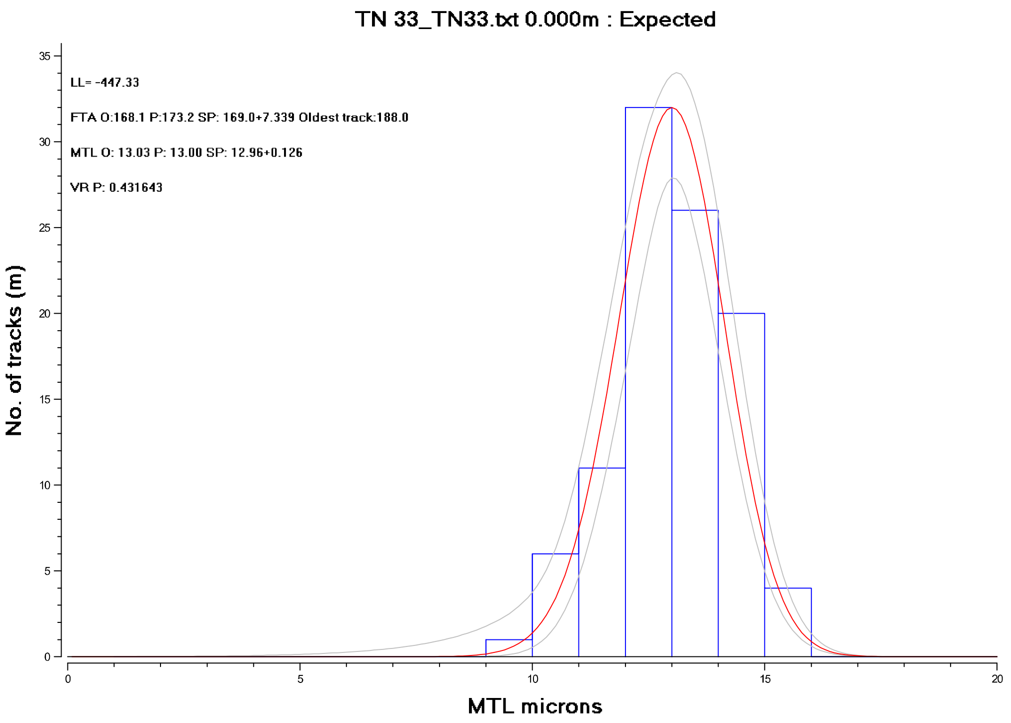
TN 33_TN33.txt 0.000m : Expected
LL= -447.33
FTA O:168.1 P:173.2 SP: 169.0+7.339 Oldest track:188.0
MTL O: 13.03 P: 13.00 SP: 12.96+0.126
VR P: 0.431643
No. of tracks (m)
MTL microns

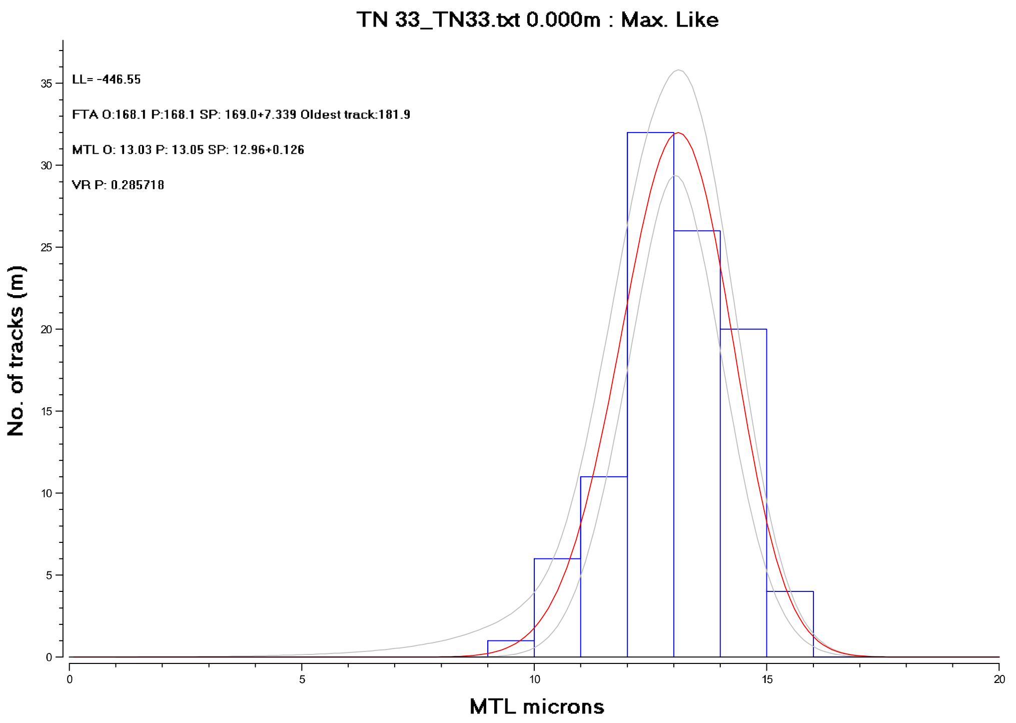
TN 33_TN33.txt 0.000m : Max. Like
LL= -446.55
FTA O:168.1 P:168.1 SP: 169.0+7.339 Oldest track:181.9
MTL O: 13.03 P: 13.05 SP: 12.96+0.126
VR P: 0.205718
No. of tracks (m)
MTL microns

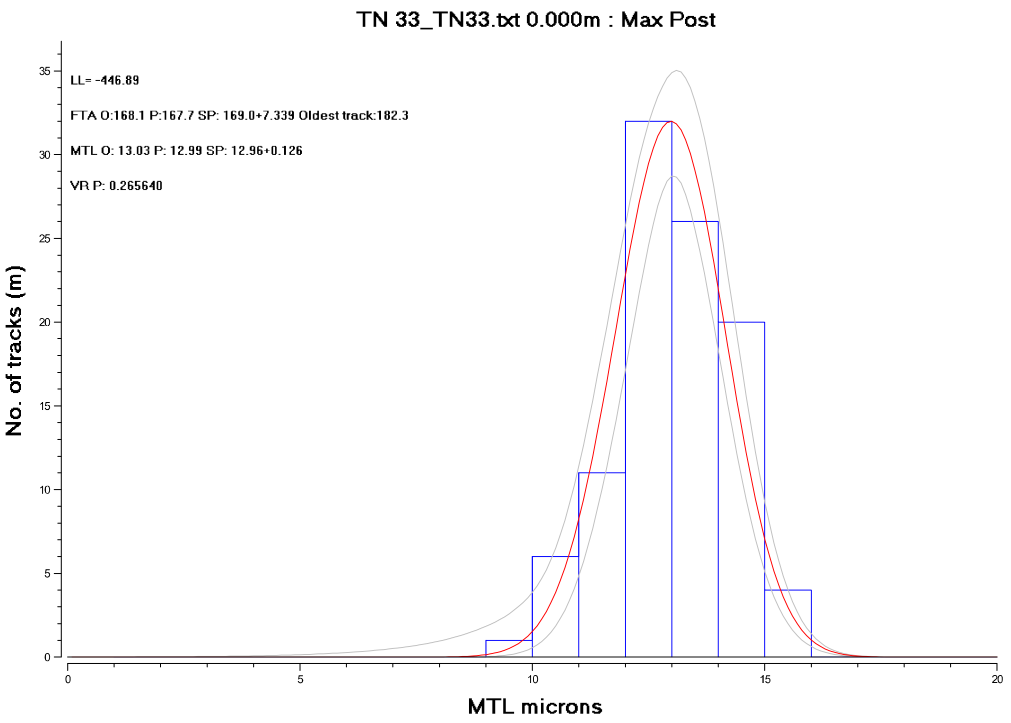
TN 33_TN33.txt 0.000m : Max Post
LL= -446.89
FTA O:168.1 P:167.7 SP: 169.0+7.339 Oldest track:182.3
MTL O: 13.03 P: 12.99 SP: 12.96+0.126
VR P: 0.265640
No. of tracks (m)
MTL microns

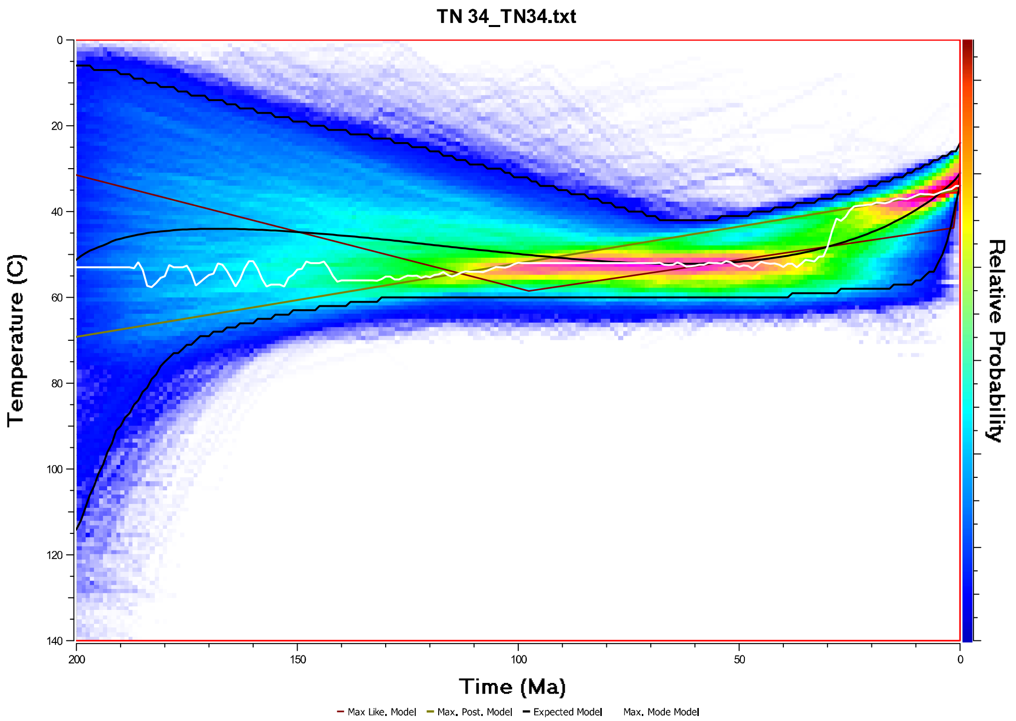
TN 34_TN34.txt
Temperature (C)
Time (Ma)
Relative Probability
Max Like. Model
Max. Post. Model
Expected Model
Max. Mode Model

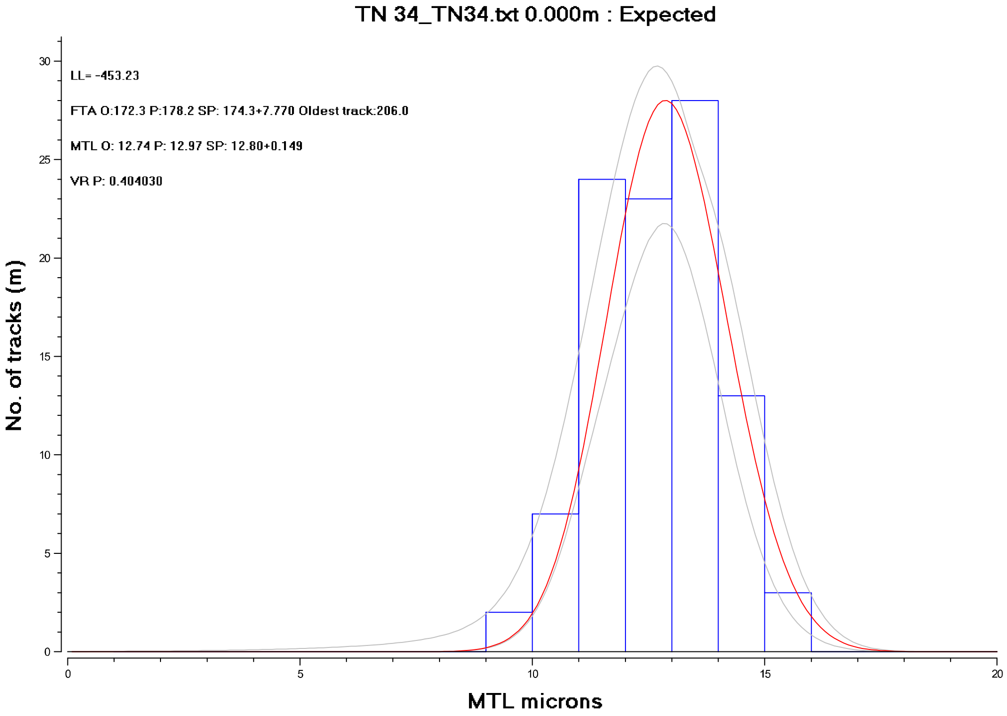
TN 34_TN34.txt 0.000m : Expected
LL= -453.23
FTA O:172.3 P:178.2 SP: 174.3+7.770 Oldest track:206.0
MTL O: 12.74 P: 12.97 SP: 12.80+0.149
VR P: 0.404030
No. of tracks (m)
MTL microns

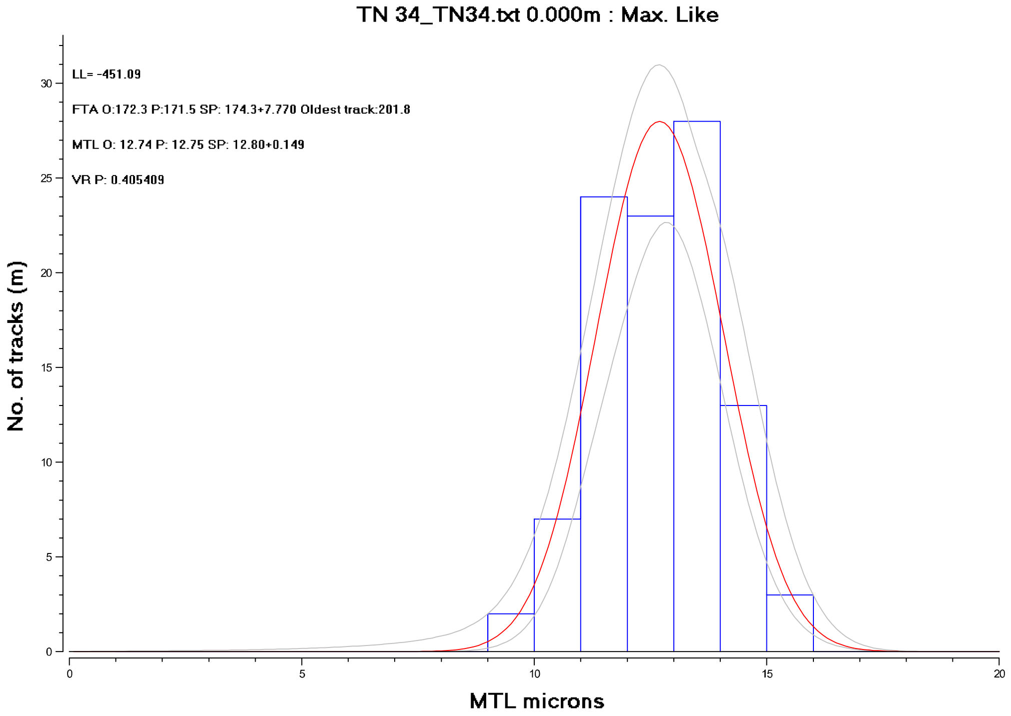
TN 34_TN34.txt 0.000m : Max. Like
LL= -451.09
FTA O:172.3 P:171.5 SP: 174.3+7.770 Oldest track:201.8
MTL O: 12.74 P: 12.75 SP: 12.80+0.149
VR P: 0.405409
No. of tracks (m)
MTL microns

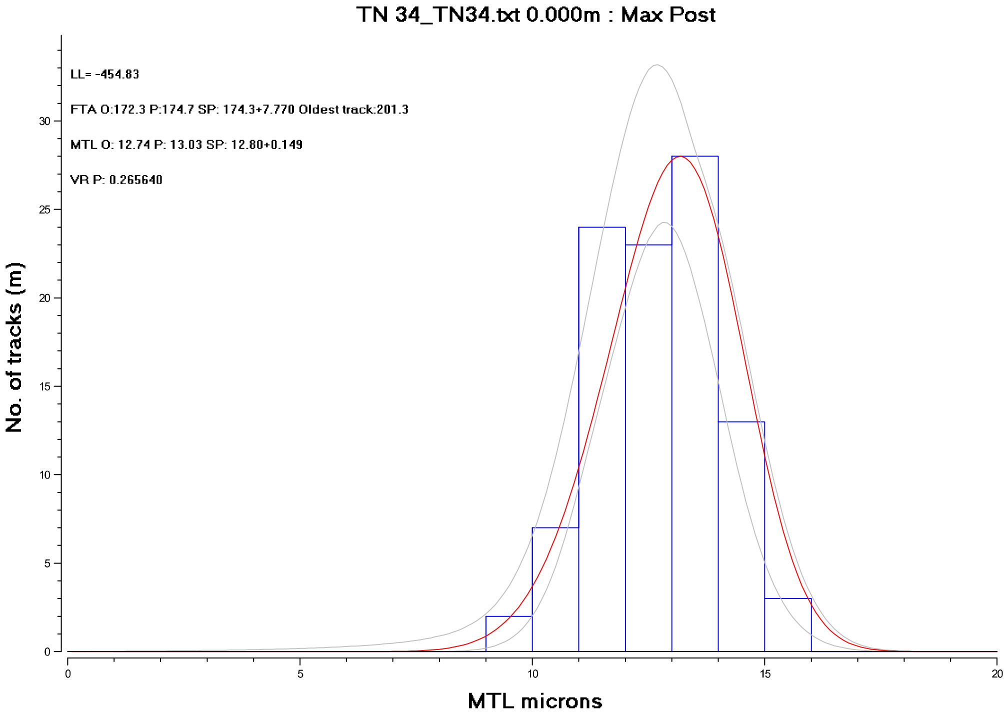
TN 34_TN34.txt 0.000m : Max Post
LL= -454.83
FTA O:172.3 P:174.7 SP: 174.3+7.770 Oldest track:201.3
MTL O: 12.74 P: 13.03 SP: 12.80+0.149
VR P: 0.265640
No. of tracks (m)
MTL microns

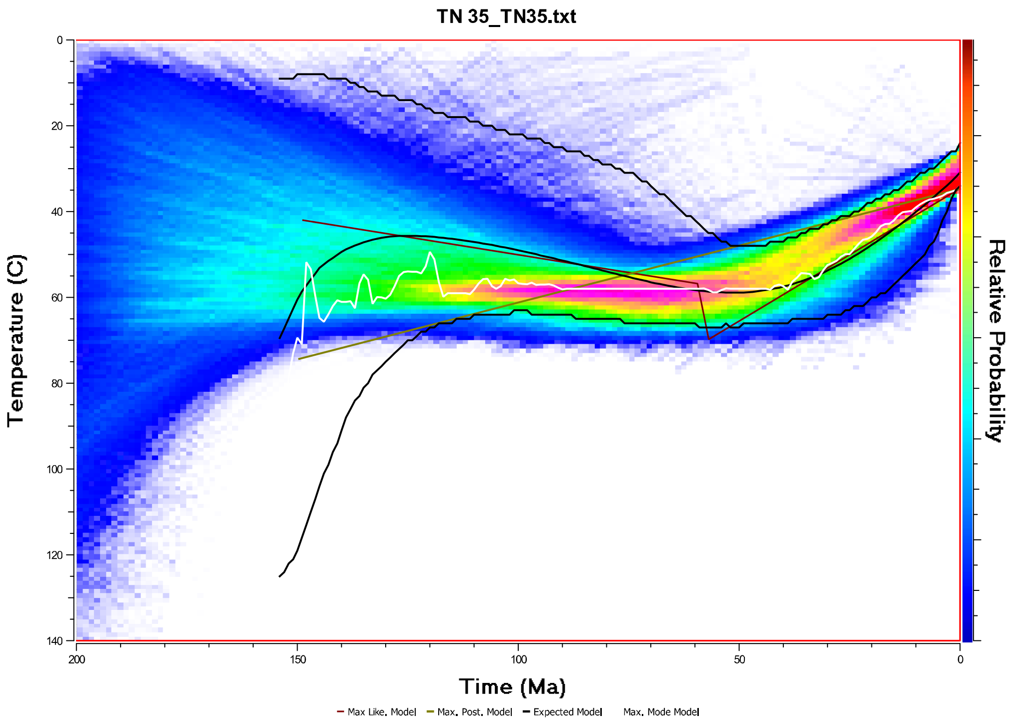
TN 35_TN35.txt
Temperature (C)
Time (Ma)
Relative Probability
Max Like. Model
Max. Post. Model
Expected Model
Max. Mode Model

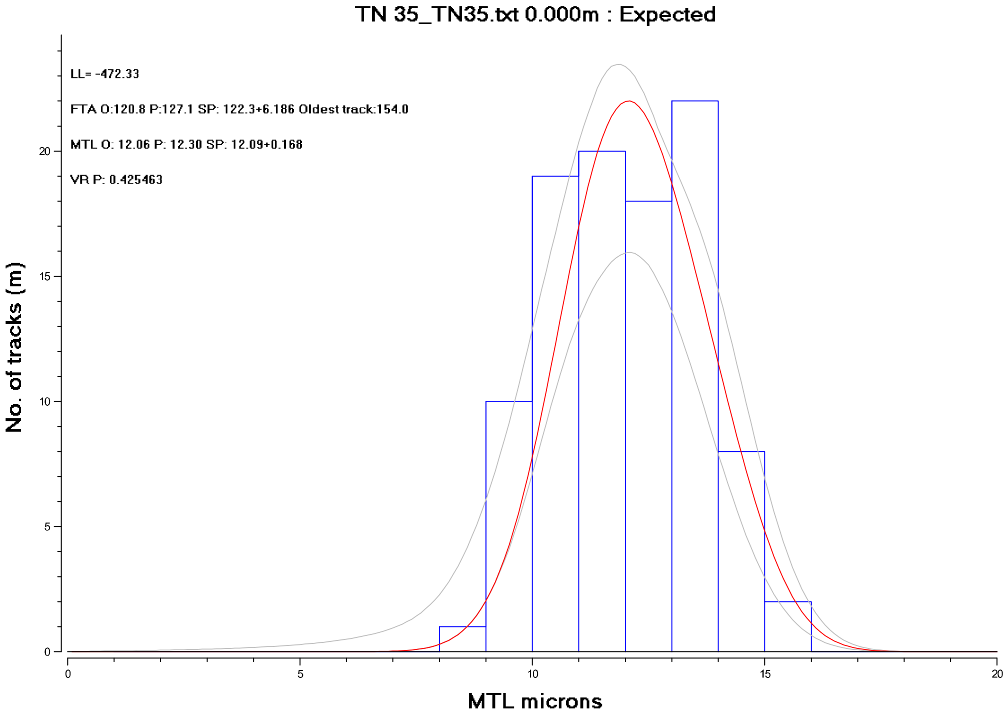
TN 35_TN35.txt 0.000m : Expected
LL= -472.33
FTA O:120.8 P:127.1 SP: 122.3+6.186 Oldest track:154.0
MTL O: 12.06 P: 12.30 SP: 12.09+0.168
VR P: 0.425463
No. of tracks (m)
MTL microns

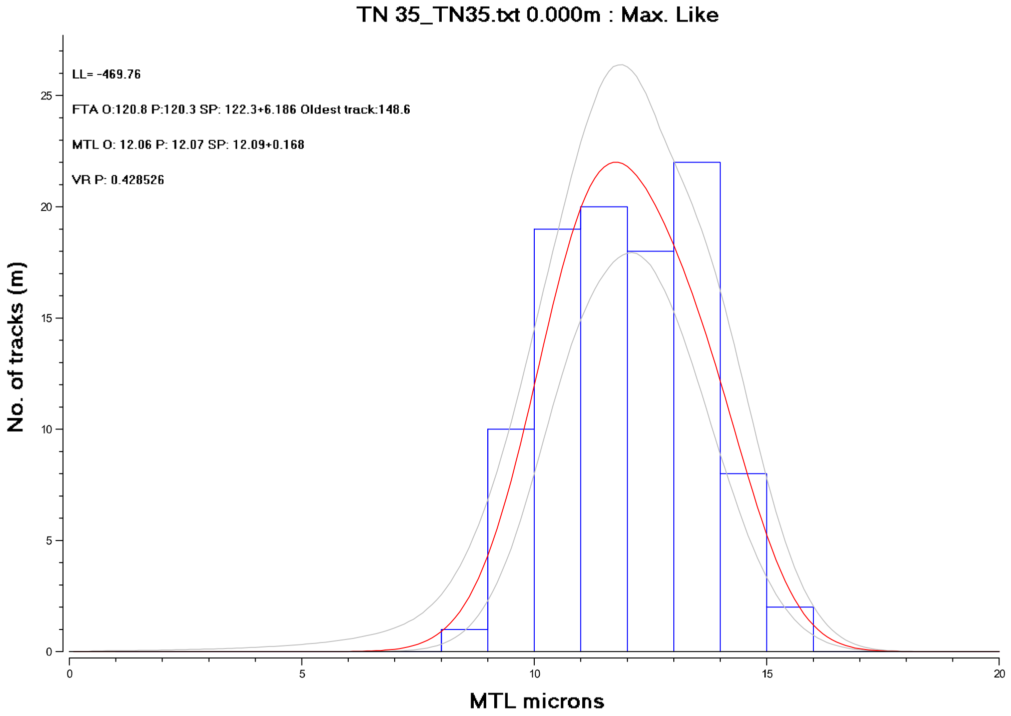
TN 35_TN35.txt 0.000m : Max. Like
LL= -469.76
FTA O:120.8 P:120.3 SP: 122.3+6.186 Oldest track:148.6
MTL O: 12.06 P: 12.07 SP: 12.09+0.168
VR P: 0.428526
No. of tracks (m)
MTL microns

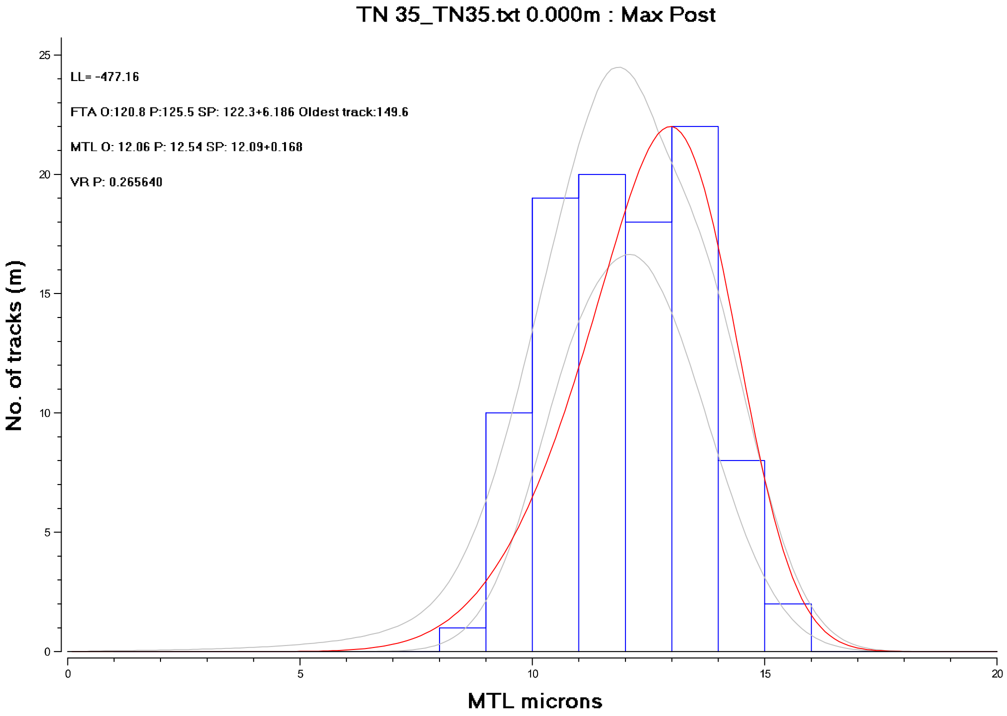
TN 35_TN35.txt 0.000m : Max Post
LL= -477.16
FTA O:120.8 P:125.5 SP: 122.3+6.186 Oldest track:149.6
MTL O: 12.06 P: 12.54 SP: 12.09+0.168
VR P: 0.265640
No. of tracks (m)
MTL microns

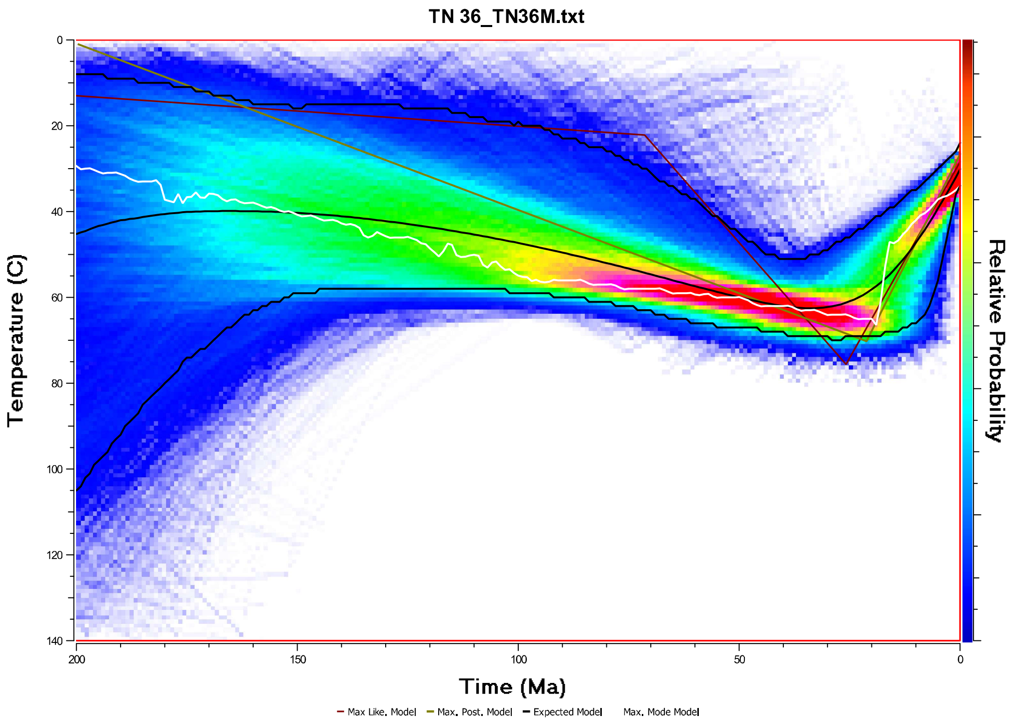

TN 36_TN36M.txt
Temperature (C)
Time (Ma)
Relative Probability
Max Like. Model
Max. Post. Model
Expected Model
Max. Mode Model

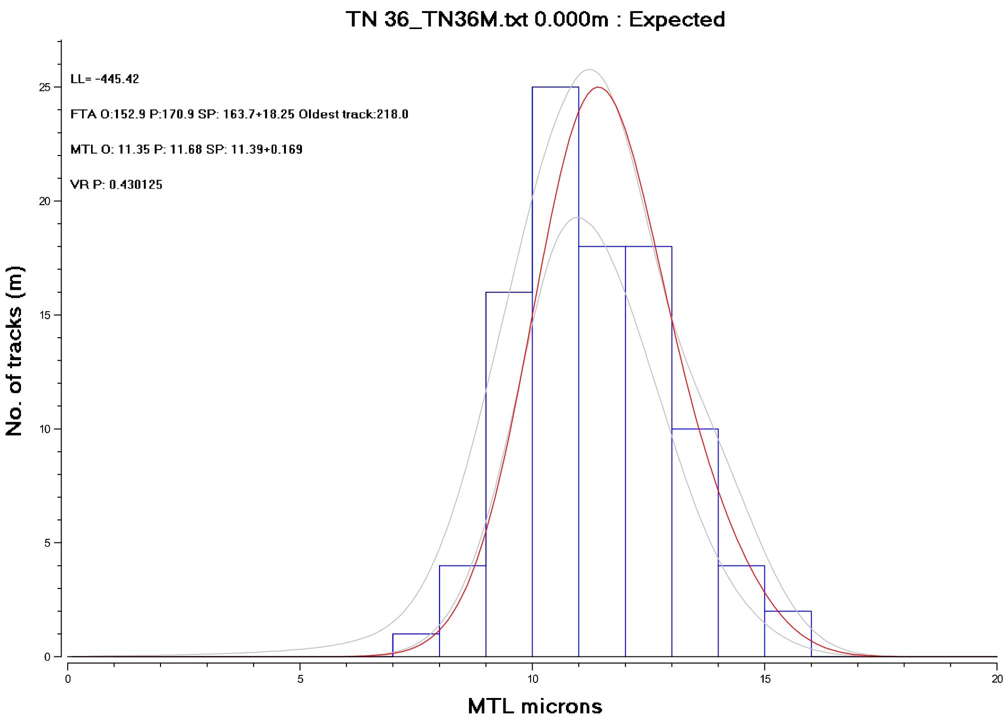

TN 36_TN36M.txt 0.000m : Expected
LL= -445.42
FTA O:152.9 P:170.9 SP: 163.7+18.25 Oldest track:218.0
MTL O: 11.35 P: 11.68 SP: 11.39+0.169
VR P: 0.430125
No. of tracks (m)
MTL microns

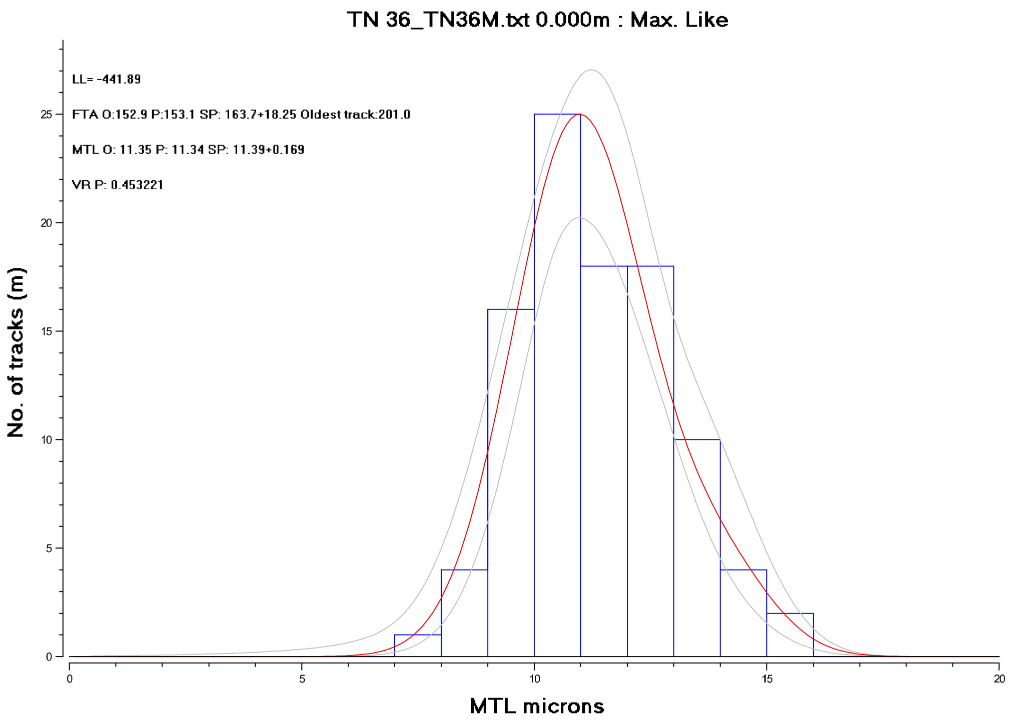

TN 36_TN36M.txt 0.000m : Max. Like
LL= -441.89
FTA O:152.9 P:153.1 SP: 163.7+18.25 Oldest track:201.0
MTL O: 11.35 P: 11.34 SP: 11.39+0.169
VR P: 0.453221
No. of tracks (m)
MTL microns

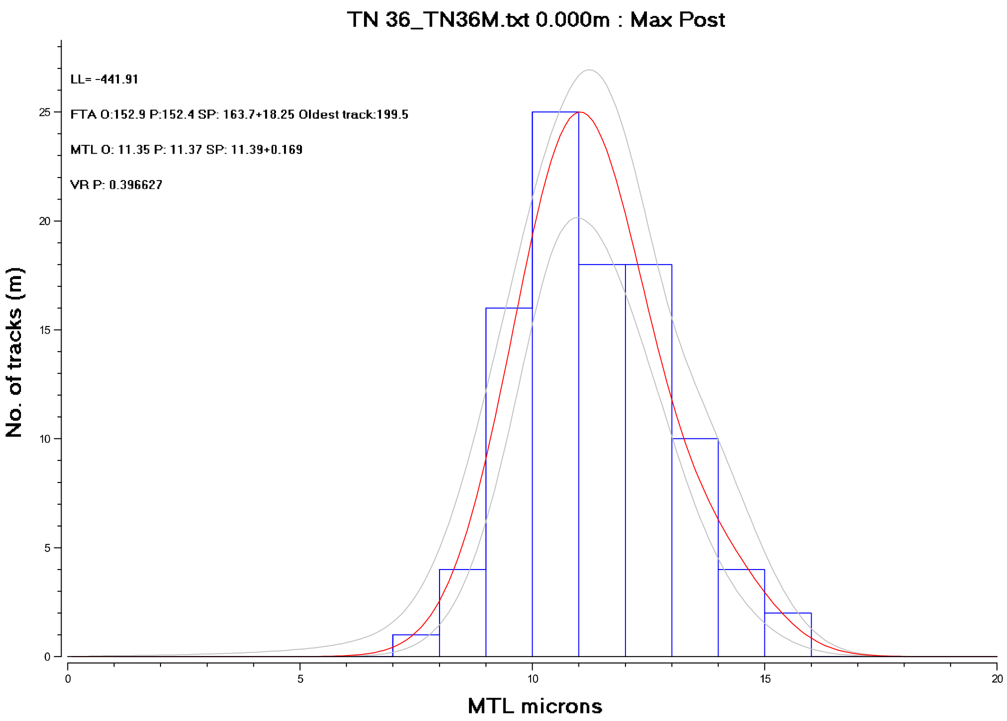

TN 36_TN36M.txt 0.000m : Max Post
LL= -441.91
FTA O:152.9 P:152.4 SP: 163.7+18.25 Oldest track:199.5
MTL O: 11.35 P: 11.37 SP: 11.39+0.169
VR P: 0.396627
No. of tracks (m)
MTL microns

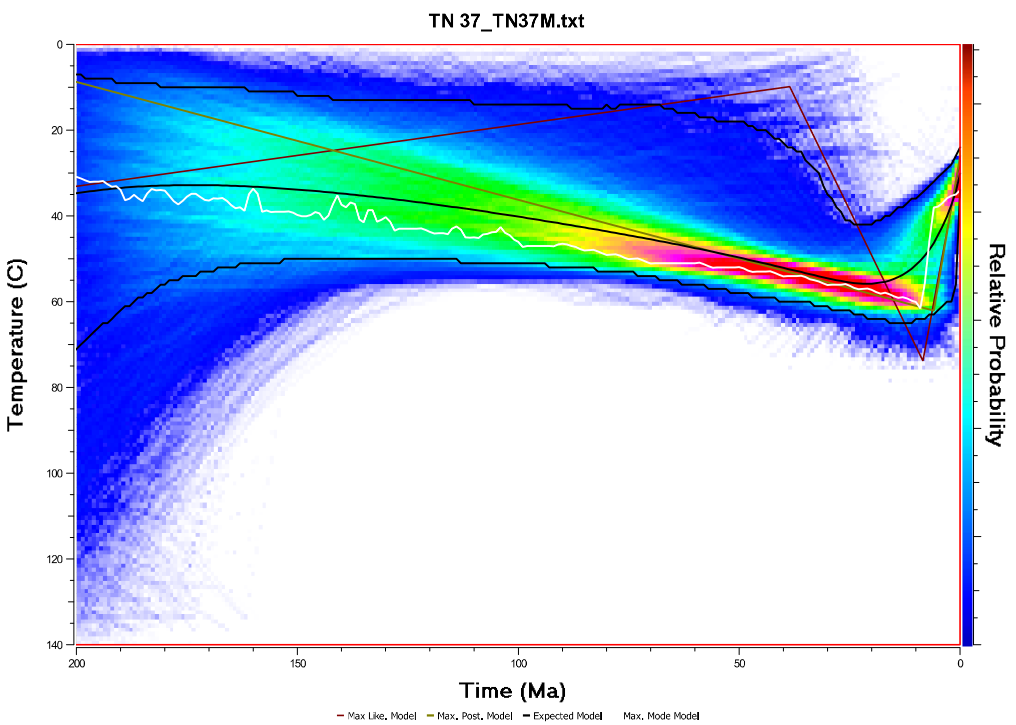
TN 37_TN37M.txt
Temperature (C)
Time (Ma)
Relative Probability
Max Like. Model
Max. Post. Model
Expected Model
Max. Mode Model

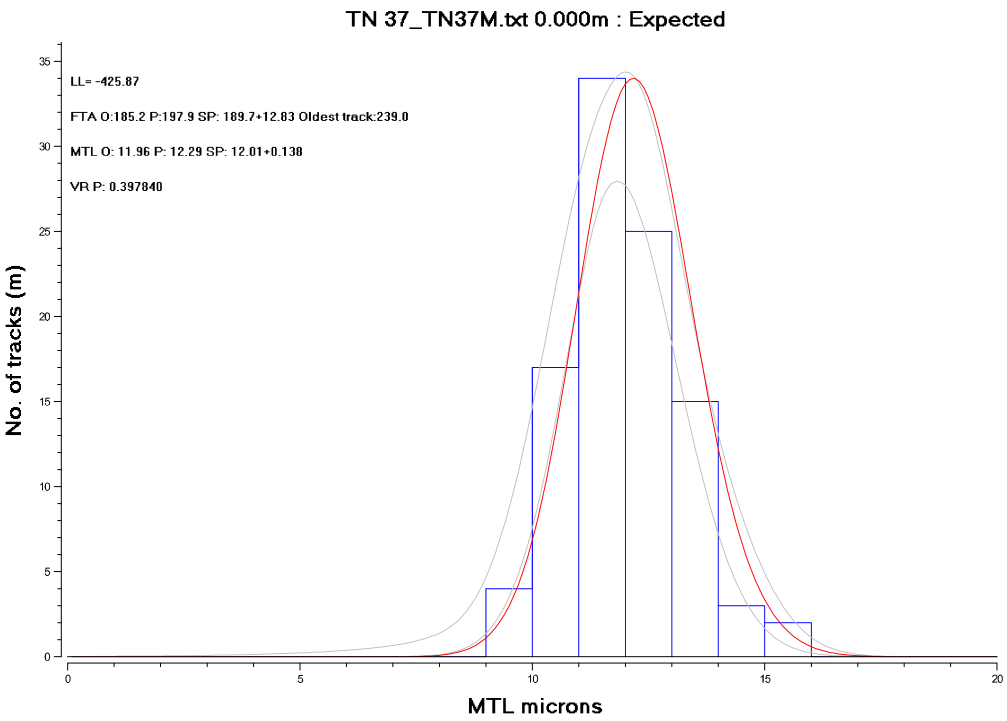
TN 37_TN37M.txt 0.000m : Expected
LL= -425.87
FTA O:185.2 P:197.9 SP: 189.7+12.83 Oldest track:239.0
MTL O: 11.96 P: 12.29 SP: 12.01+0.138
VR P: 0.397840
No. of tracks (m)
MTL microns

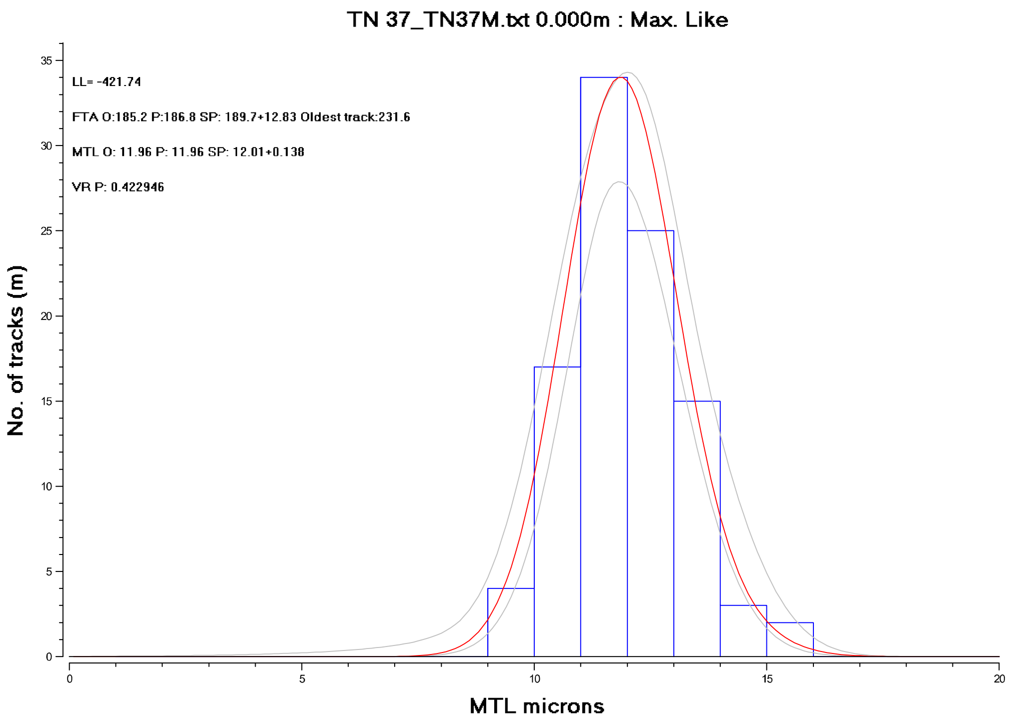
TN 37_TN37M.txt 0.000m : Max. Like
LL= -421.74
FTA O:185.2 P:186.8 SP: 189.7+12.83 Oldest track:231.6
MTL O: 11.96 P: 11.96 SP: 12.01+0.138
VR P: 0.422946
No. of tracks (m)
MTL microns

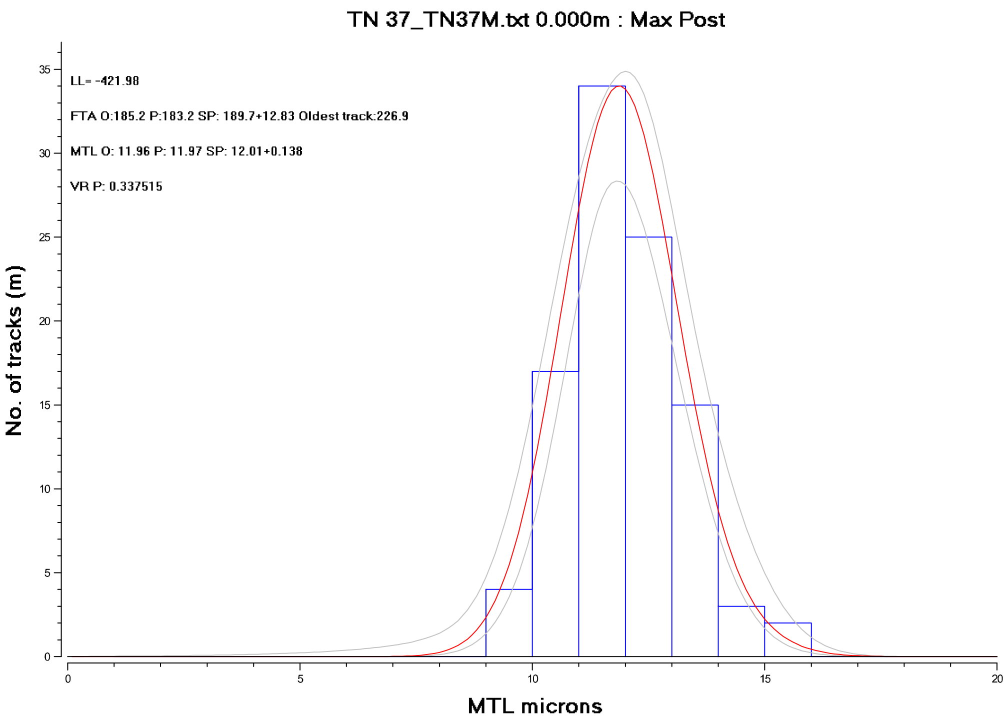
TN 37_TN37M.txt 0.000m : Max Post
LL= -421.98
FTA O:185.2 P:183.2 SP: 189.7+12.83 Oldest track:226.9
MTL O: 11.96 P: 11.97 SP: 12.01+0.138
VR P: 0.337515
No. of tracks (m)
MTL microns

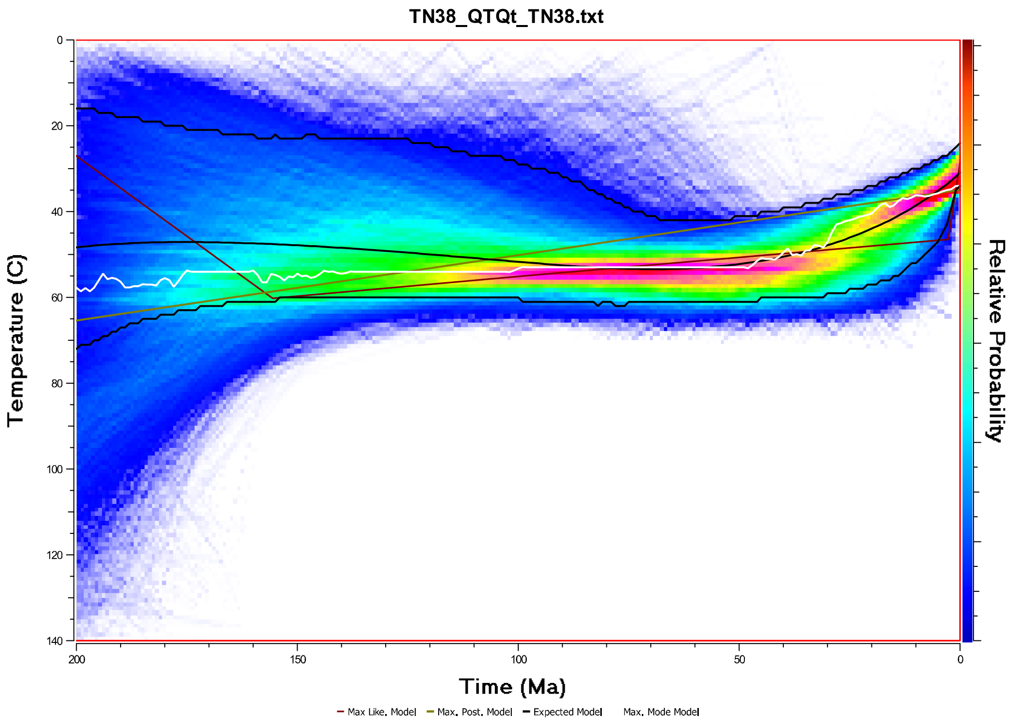
TN38_QTQt_TN38.txt
Temperature (C)
Time (Ma)
Relative Probability
Max Like. Model
Max. Post. Model
Expected Model
Max. Mode Model

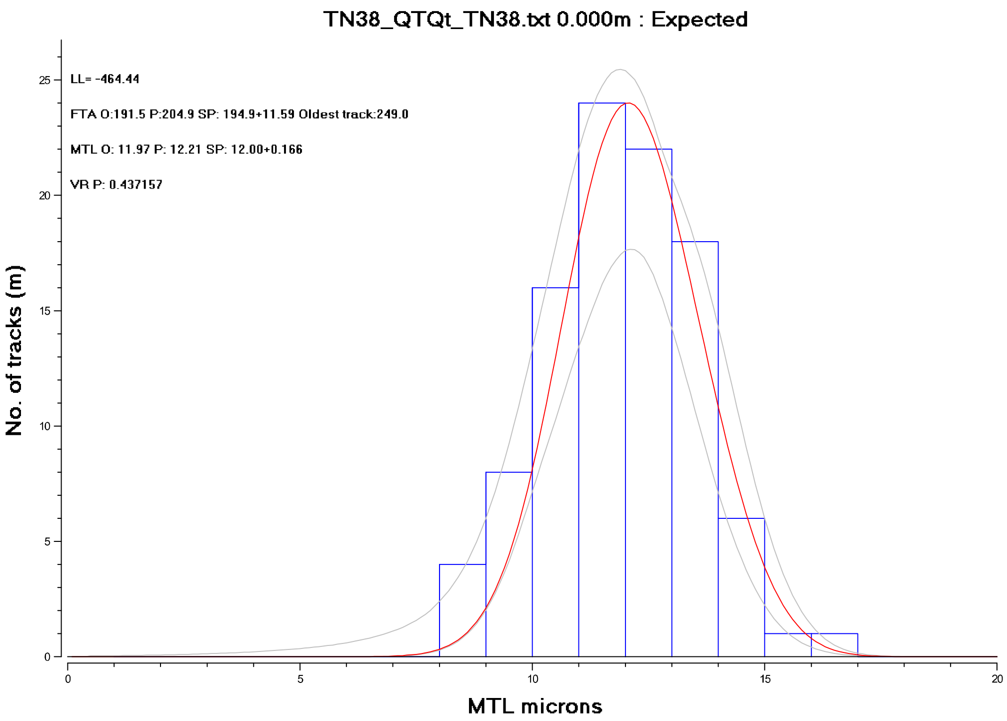
TN38_QTQt_TN38.txt 0.000m : Expected
LL= -464.44
FTA O:191.5 P:204.9 SP: 194.9+11.59 Oldest track:249.0
MTL O: 11.97 P: 12.21 SP: 12.00+0.166
VR P: 0.437157
No. of tracks (m)
MTL microns

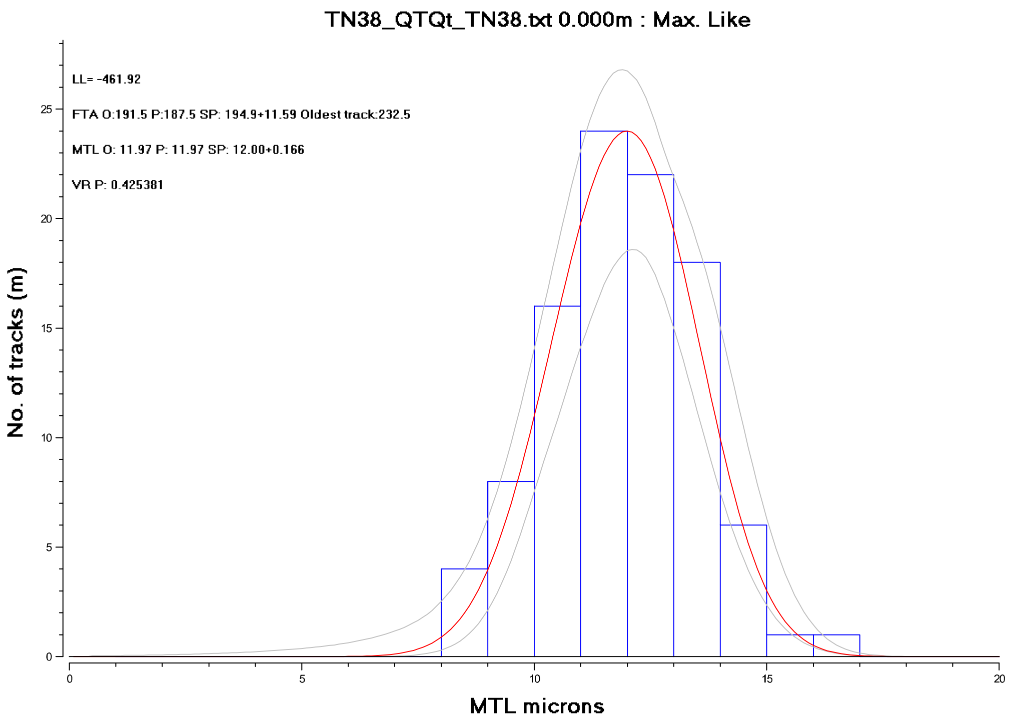
TN38_QTQt_TN38.txt 0.000m : Max. Like
LL= -461.92
FTA O:191.5 P:187.5 SP: 194.9+11.59 Oldest track:232.5
MTL O: 11.97 P: 11.97 SP: 12.00+0.166
VR P: 0.425381
No. of tracks (m)
MTL microns

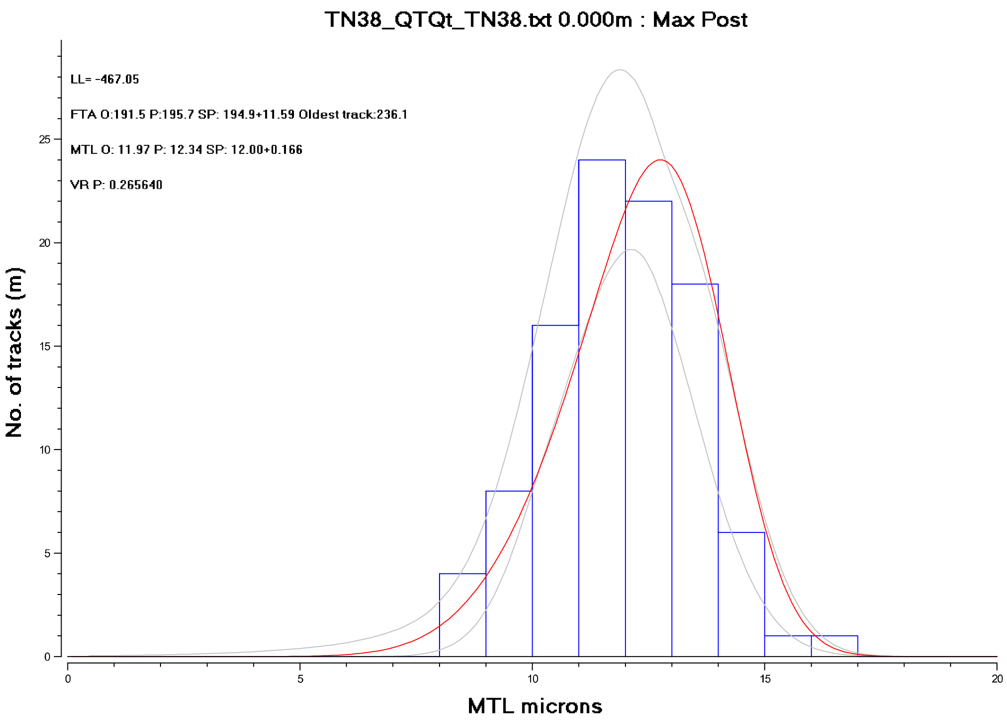
TN38_QTQt_TN38.txt 0.000m : Max Post
LL= -467.05
FTA O:191.5 P:195.7 SP: 194.9+11.59 Oldest track:236.1
MTL O: 11.97 P: 12.34 SP: 12.00+0.166
VR P: 0.265640
No. of tracks (m)
MTL microns

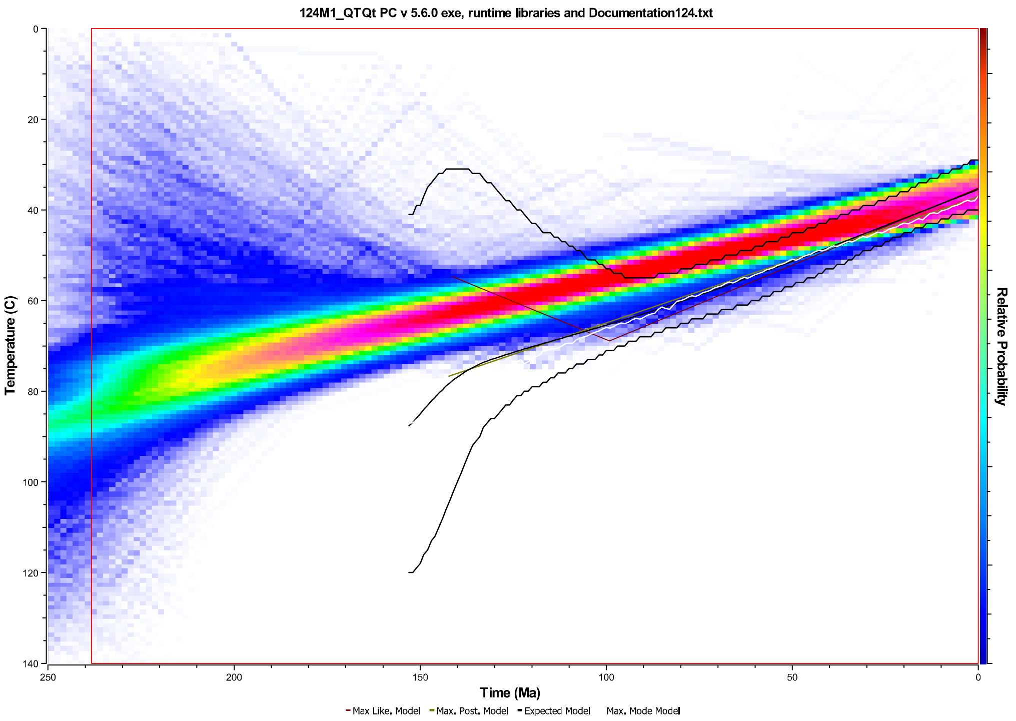
124M1_QTQt PC v 5.6.0 exe, runtime libraries and Documentation124.txt
Temperature (C)
Time (Ma)
Relative Probability
Max Like. Model
Max. Post. Model
Expected Model
Max. Mode Model

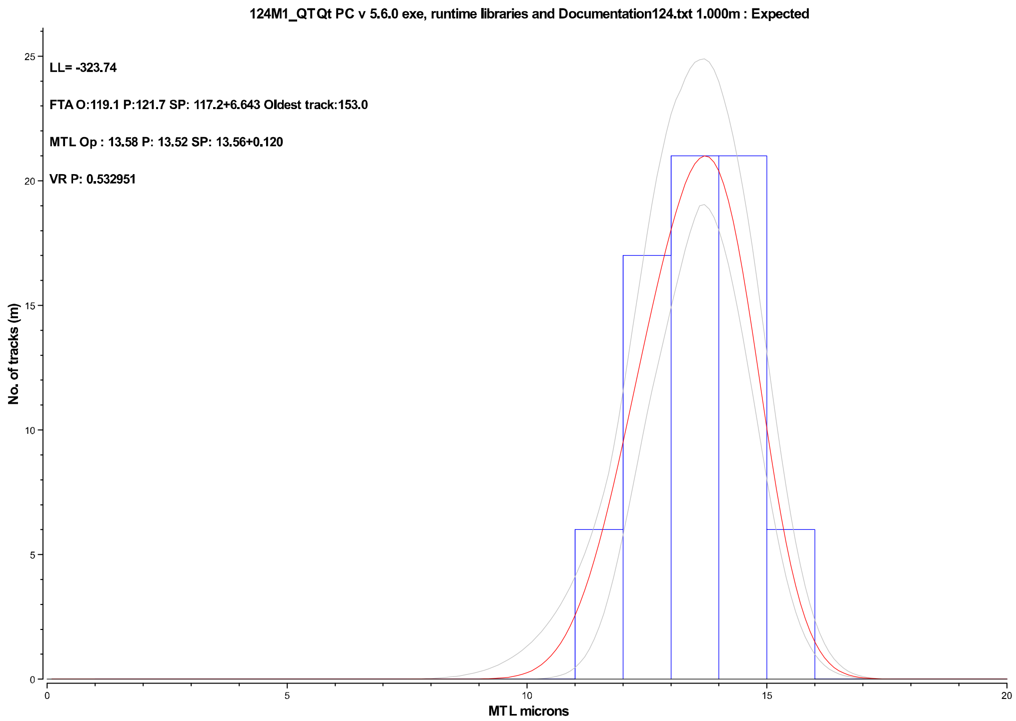
124M1_QTQt PC v 5.6.0 exe, runtime libraries and Documentation124.txt 1.000m : Expected
LL= -323.74
FTA O:119.1 P:121.7 SP: 117.2+6.643 Oldest track:153.0
MTL Op : 13.58 P: 13.52 SP: 13.56+0.120
VR P: 0.532951
No. of tracks (m)
MTL microns

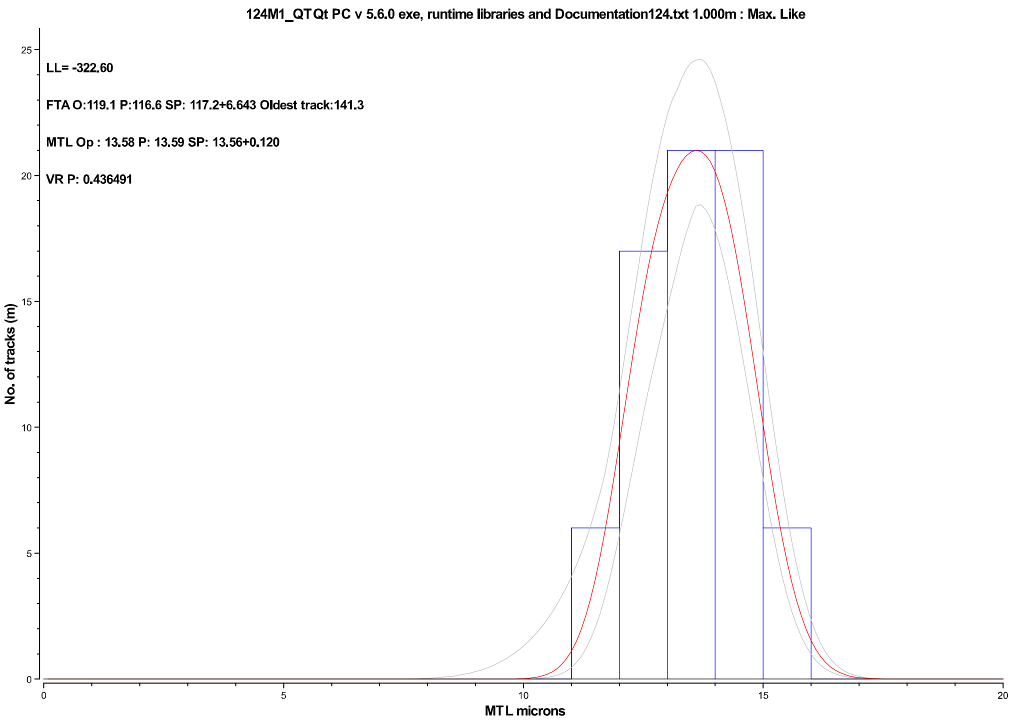
124M1_QTQt PC v 5.6.0 exe, runtime libraries and Documentation124.txt 1.000m : Max. Like
LL= -322.60
FTA O:119.1 P:116.6 SP: 117.2+6.643 Oldest track:141.3
MTL Op : 13.58 P: 13.59 SP: 13.56+0.120
VR P: 0.436491
No. of tracks (m)
MTL microns

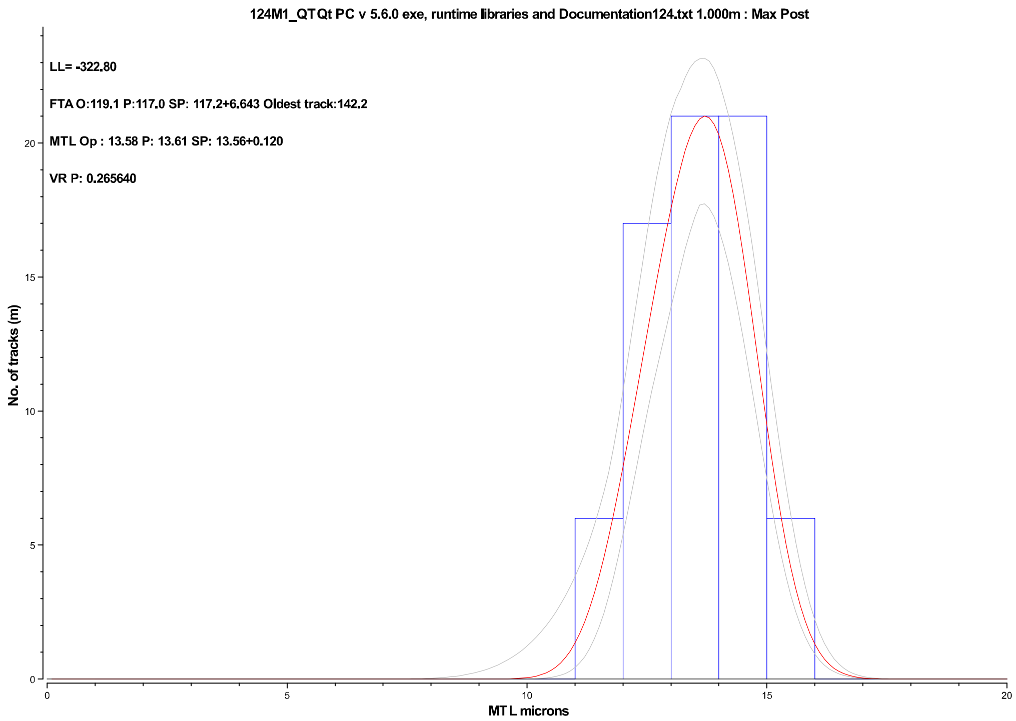
124M1_QTQt PC v 5.6.0 exe, runtime libraries and Documentation124.txt 1.000m : Max Post
LL= -322.80
FTA O:119.1 P:117.0 SP: 117.2+6.643 Oldest track:142.2
MTL Op : 13.58 P: 13.61 SP: 13.56+0.120
VR P: 0.265640
No. of tracks (m)
MTL microns

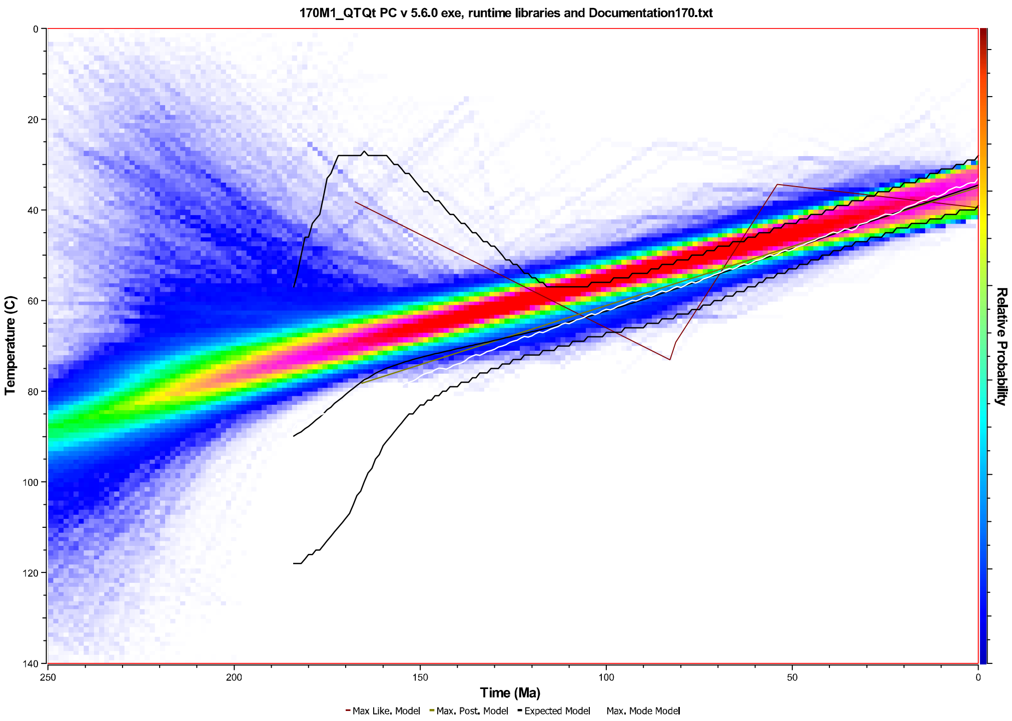
170M1_QTQt PC v 5.6.0 exe, runtime libraries and Documentation170.txt
Temperature (C)
Time (Ma)
Relative Probability
Max Like. Model
Max. Post. Model
Expected Model
Max. Mode Model

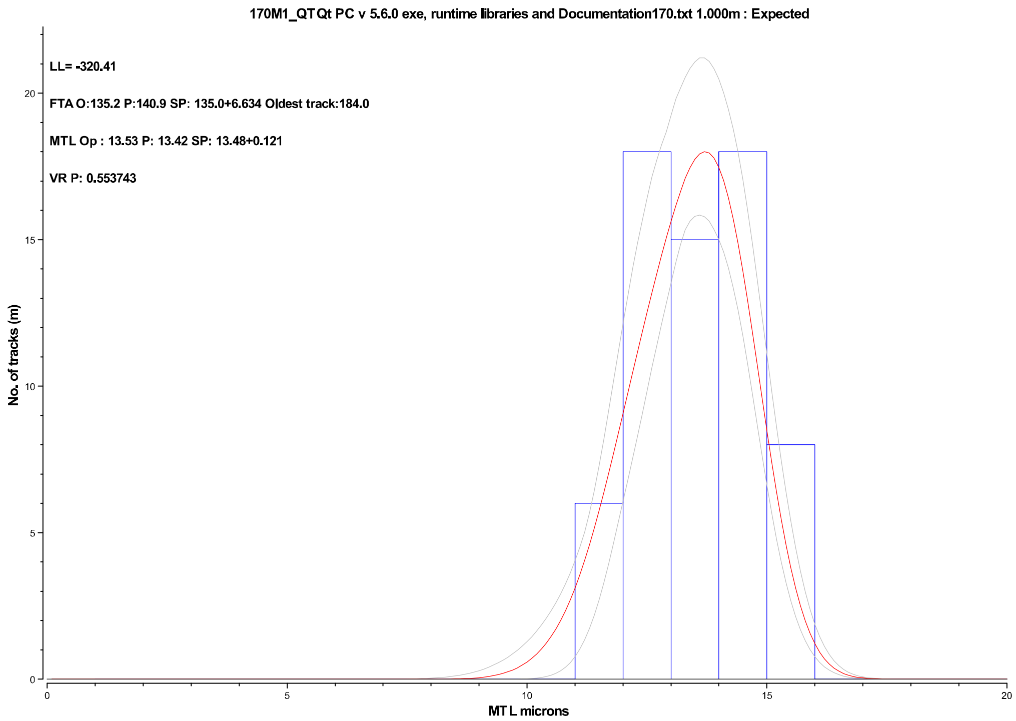
170M1_QTQt PC v 5.6.0 exe, runtime libraries and Documentation170.txt 1.000m : Expected
LL= -320.41
FTA O:135.2 P:140.9 SP: 135.0+6.634 Oldest track:184.0
MTL Op : 13.53 P: 13.42 SP: 13.48+0.121
VR P: 0.553743
No. of tracks (m)
MTL microns

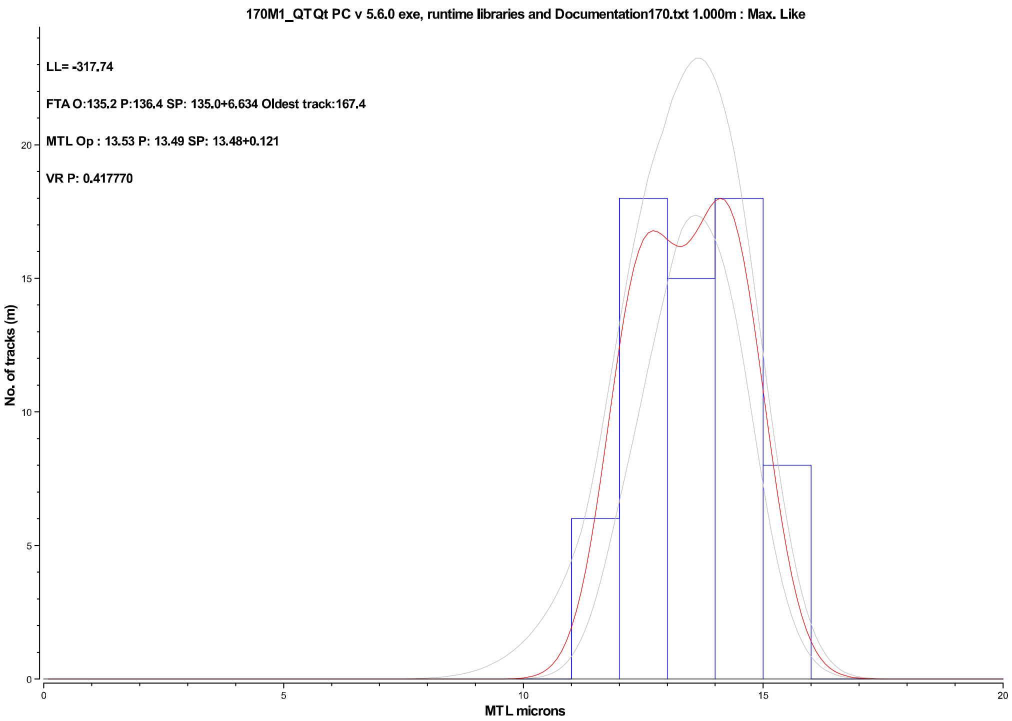
170M1_QTQt PC v 5.6.0 exe, runtime libraries and Documentation170.txt 1.000m : Max. Like
LL= -317.74
FTA O:135.2 P:136.4 SP: 135.0+6.634 Oldest track:167.4
MTL Op : 13.53 P: 13.49 SP: 13.48+0.121
VR P: 0.417770
No. of tracks (m)
MTL microns

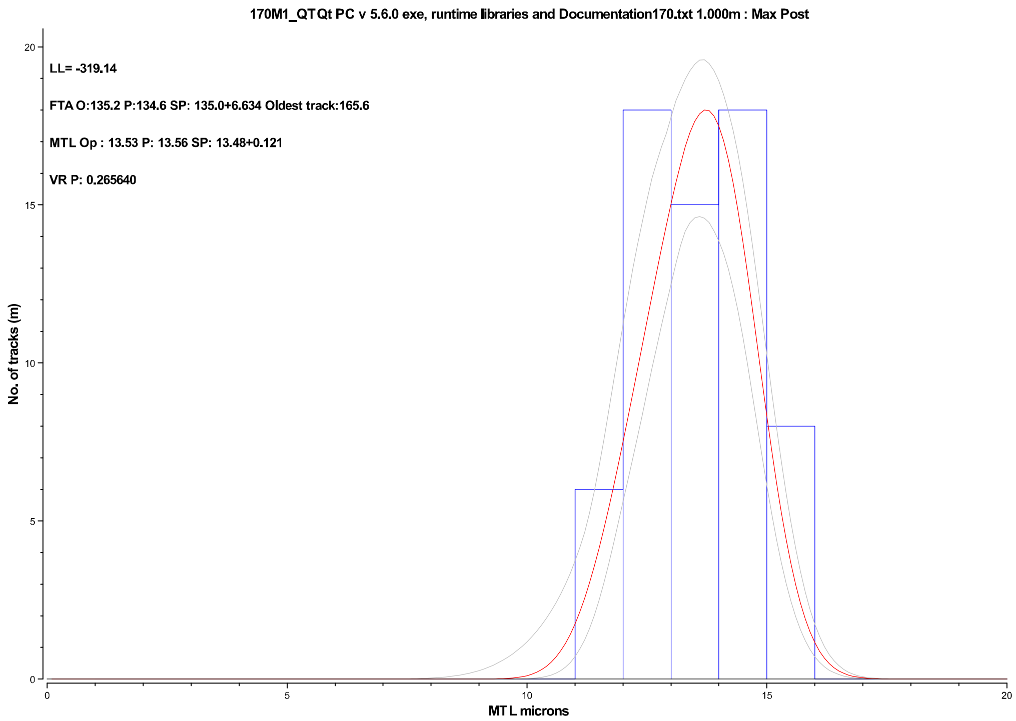
170M1_QTQt PC v 5.6.0 exe, runtime libraries and Documentation170.txt 1.000m : Max Post
LL= -319.14
FTA O:135.2 P:134.6 SP: 135.0+6.634 Oldest track:165.6
MTL Op : 13.53 P: 13.56 SP: 13.48+0.121
VR P: 0.265640
No. of tracks (m)
MTL microns

171M1_QTQt PC v 5.6.0 exe, runtime libraries and Documentation171.txt

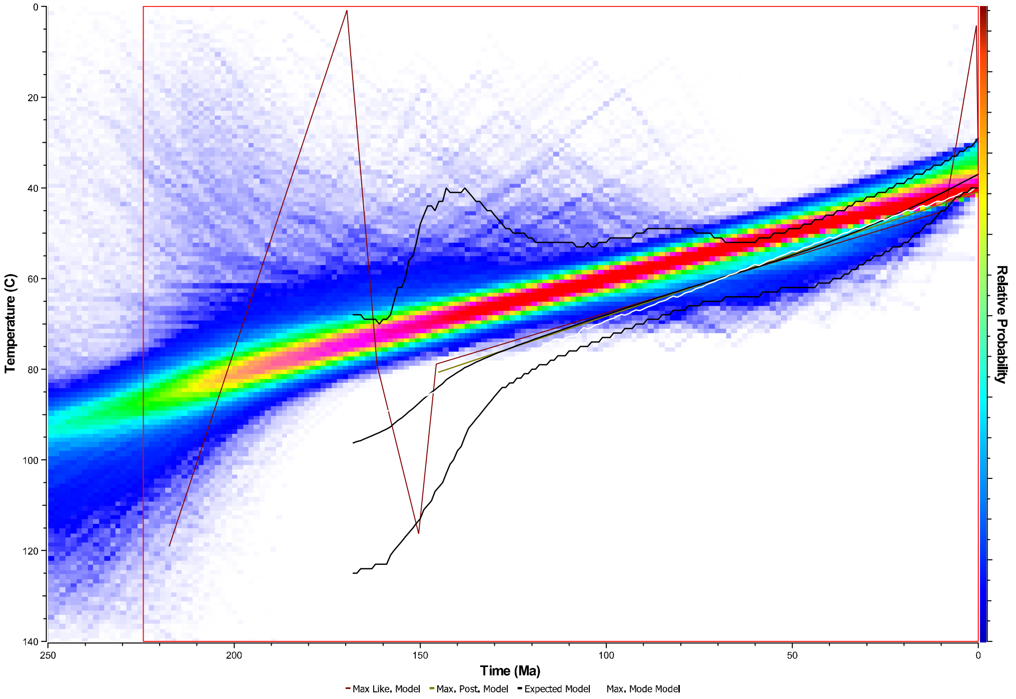

171M1_QTQt PC v 5.6.0 exe, runtime libraries and Documentation171.txt 1.000m : Expected

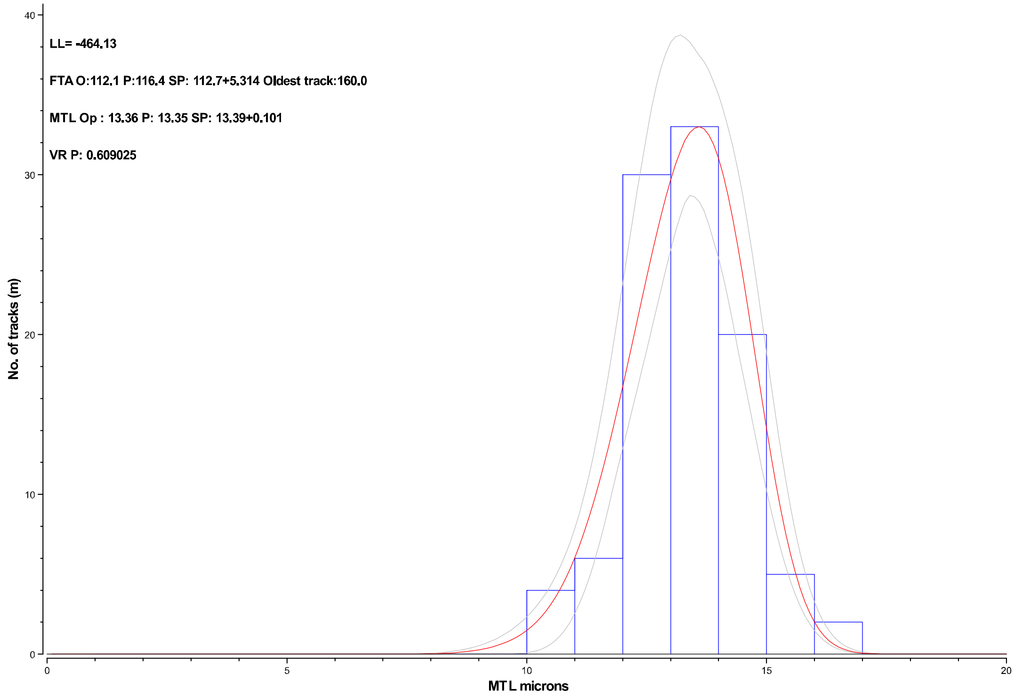

171M1_QTQt PC v 5.6.0 exe, runtime libraries and Documentation171.txt 1.000m : Max. Like

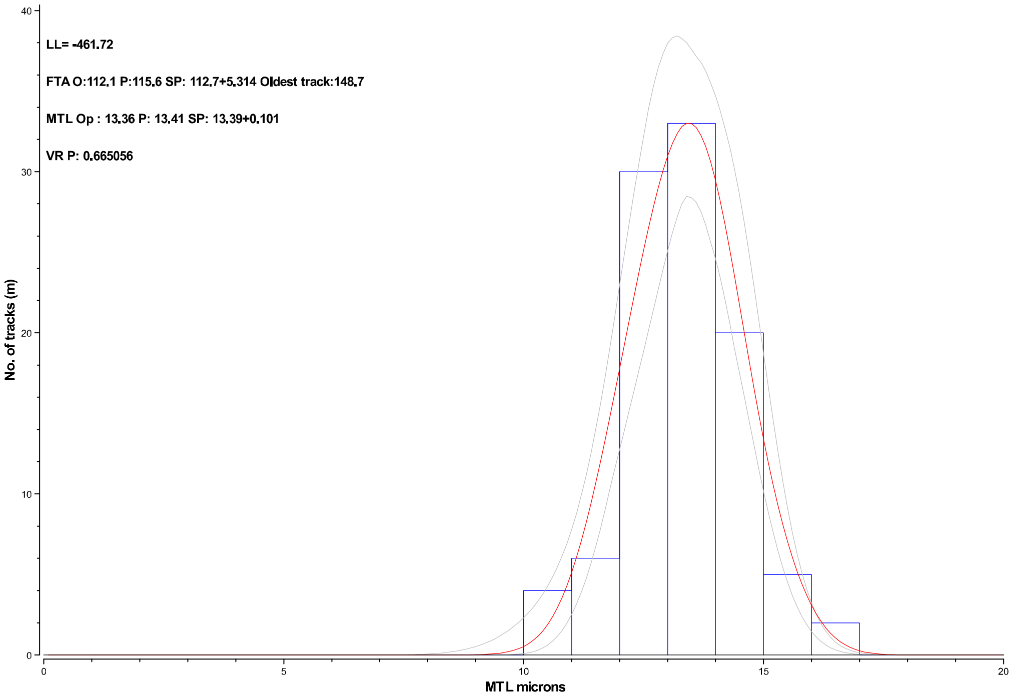

171M1_QTQt PC v 5.6.0 exe, runtime libraries and Documentation171.txt 1.000m : Max Post

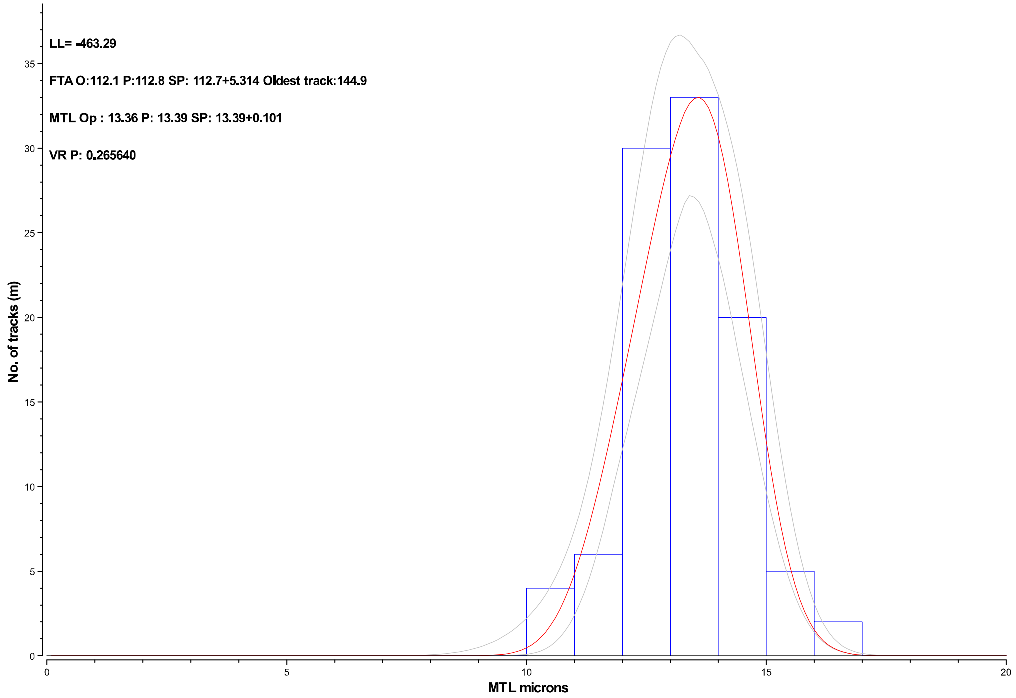

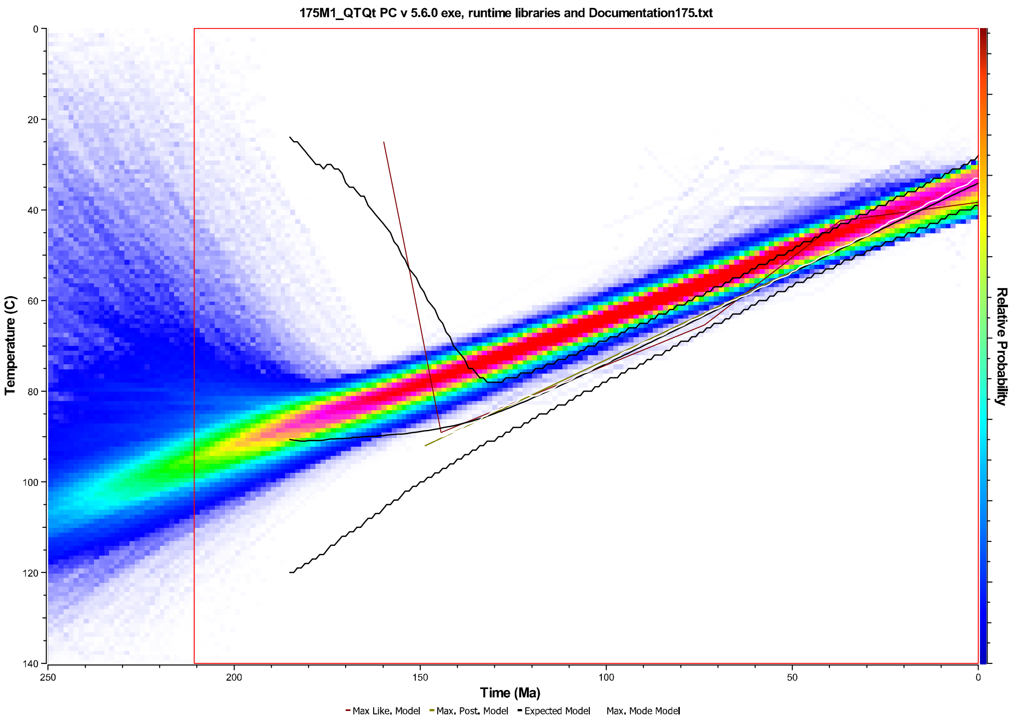
175M1_QTQt PC v 5.6.0 exe, runtime libraries and Documentation175.txt
Temperature (C)
Time (Ma)
Relative Probability
Max Like. Model
Max. Post. Model
Expected Model
Max. Mode Model

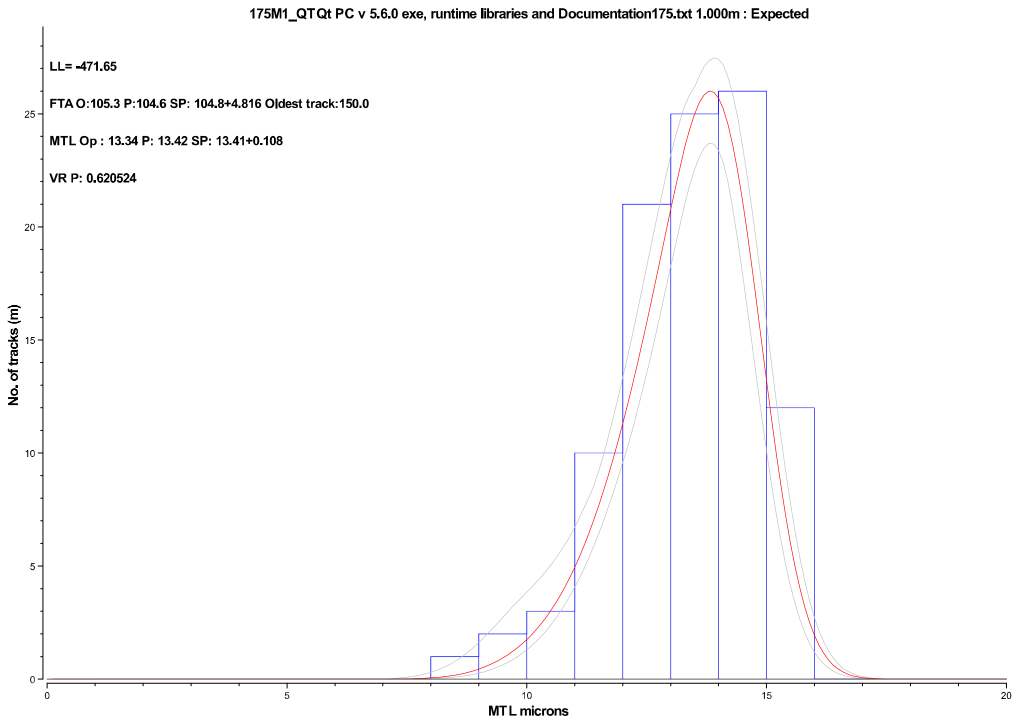
175M1_QTQt PC v 5.6.0 exe, runtime libraries and Documentation175.txt 1.000m : Expected
LL= -471.65
FTA O:105.3 P:104.6 SP: 104.8+4.816 Oldest track:150.0
MTL Op : 13.34 P: 13.42 SP: 13.41+0.108
VR P: 0.620524
No. of tracks (m)
MTL microns

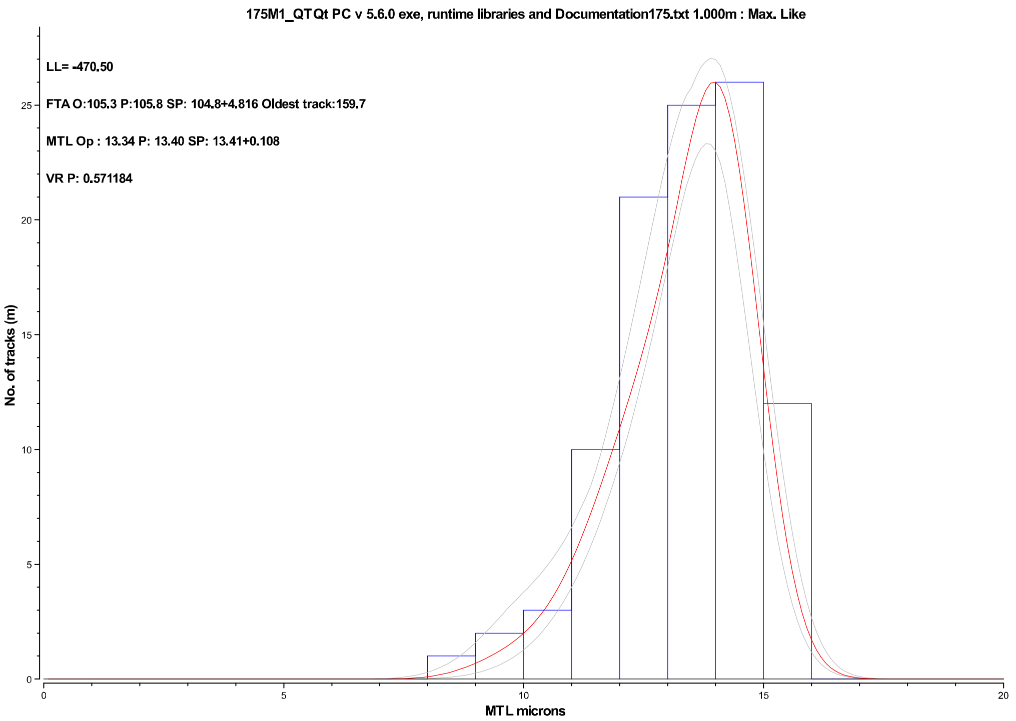
175M1_QTQt PC v 5.6.0 exe, runtime libraries and Documentation175.txt 1.000m : Max. Like
LL= -470.50
FTA O:105.3 P:105.8 SP: 104.8+4.816 Oldest track:159.7
MTL Op : 13.34 P: 13.40 SP: 13.41+0.108
VR P: 0.571184
No. of tracks (m)
MTL microns

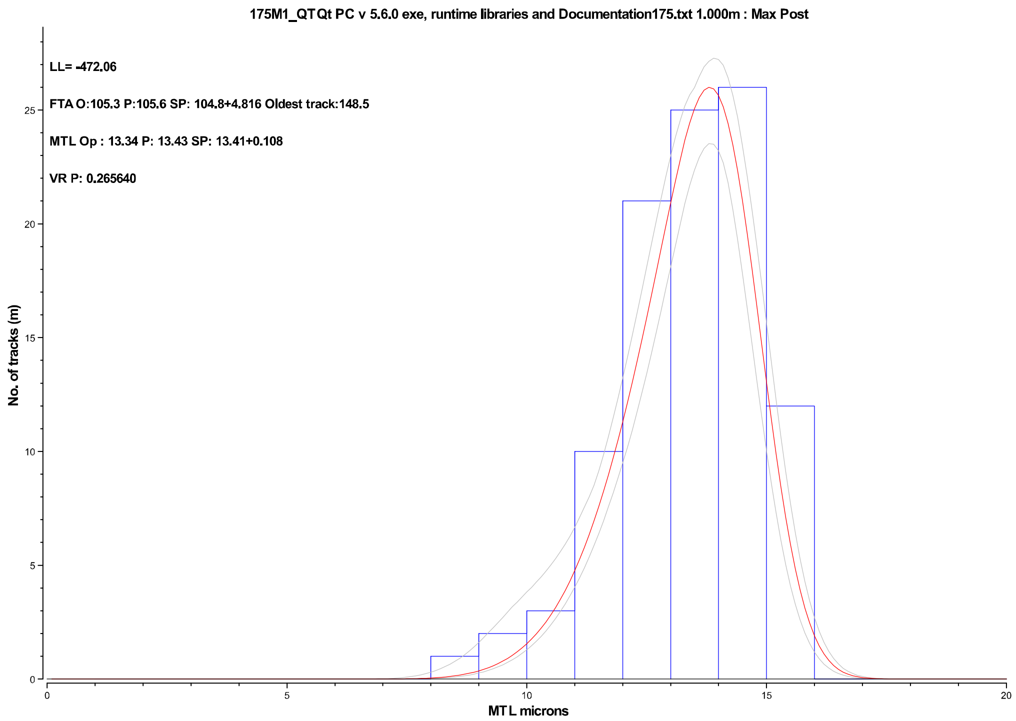
175M1_QTQt PC v 5.6.0 exe, runtime libraries and Documentation175.txt 1.000m : Max Post
LL= -472.06
FTA O:105.3 P:105.6 SP: 104.8+4.816 Oldest track:148.5
MTL Op : 13.34 P: 13.43 SP: 13.41+0.108
VR P: 0.265640
No. of tracks (m)
MTL microns

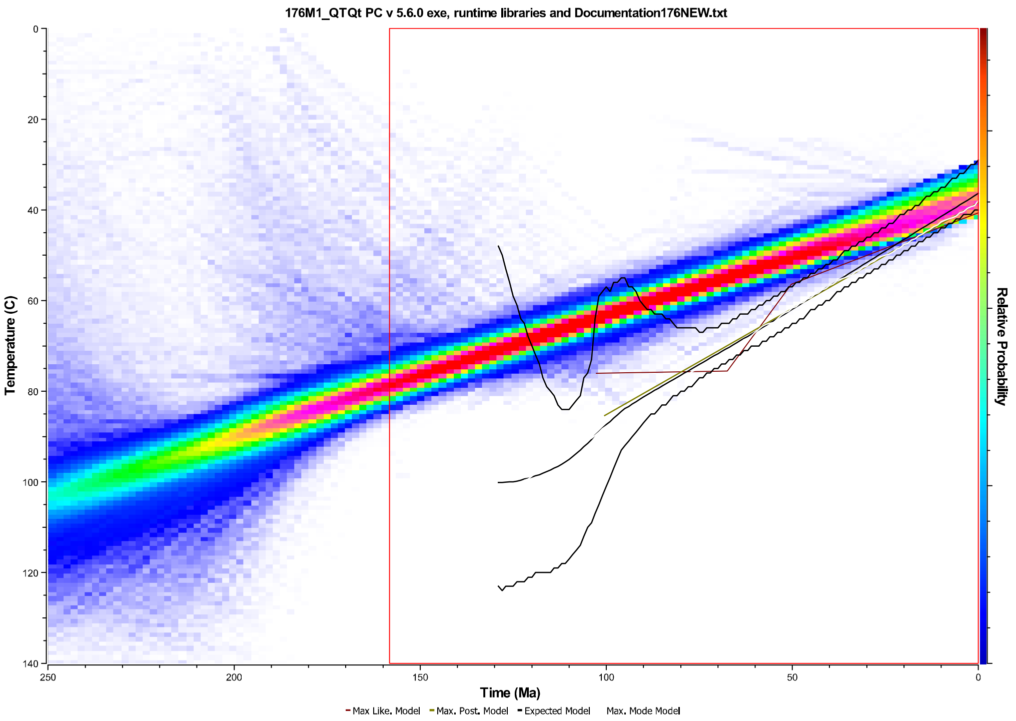
176M1_QTQt PC v 5.6.0 exe, runtime libraries and Documentation176NEW.txt
Temperature (C)
Time (Ma)
Relative Probability
Max Like. Model
Max. Post. Model
Expected Model
Max. Mode Model

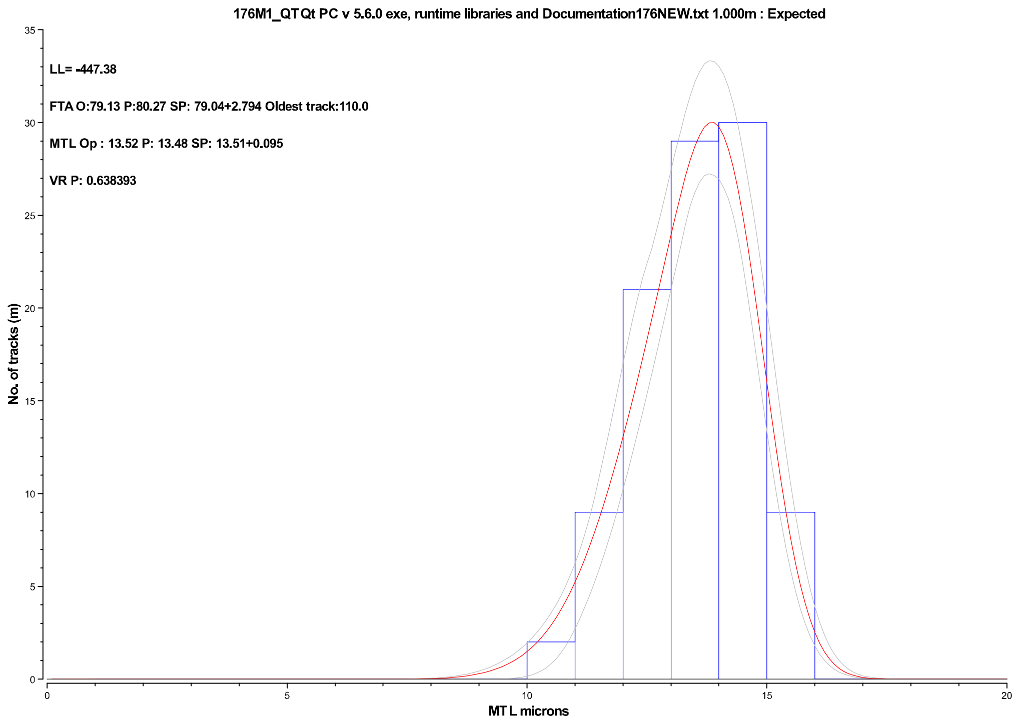
176M1_QTQt PC v 5.6.0 exe, runtime libraries and Documentation176NEW.txt 1.000m : Expected
LL= -447.38
FTA O:79.13 P:80.27 SP: 79.04+2.794 Oldest track:110.0
MTL Op : 13.52 P: 13.48 SP: 13.51+0.095
VR P: 0.638393
No. of tracks (m)
MTL microns

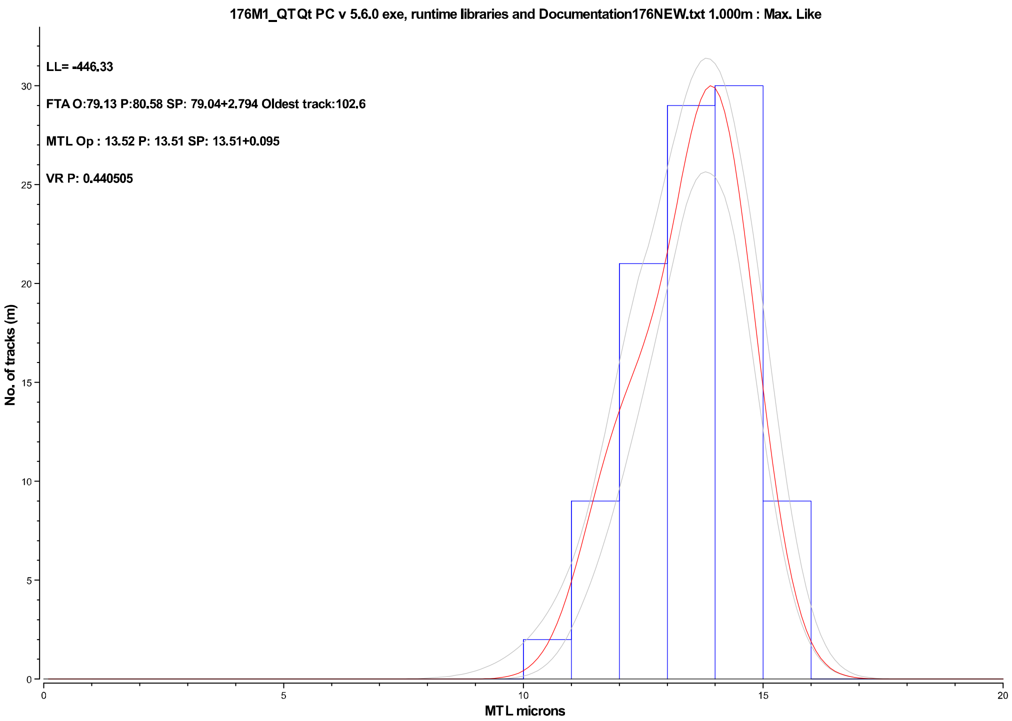
176M1_QTQt PC v 5.6.0 exe, runtime libraries and Documentation176NEW.txt 1.000m : Max. Like
LL= -446.33
FTA O:79.13 P:80.58 SP: 79.04+2.794 Oldest track:102.6
MTL Op : 13.52 P: 13.51 SP: 13.51+0.095
VR P: 0.440505
No. of tracks (m)
MTL microns

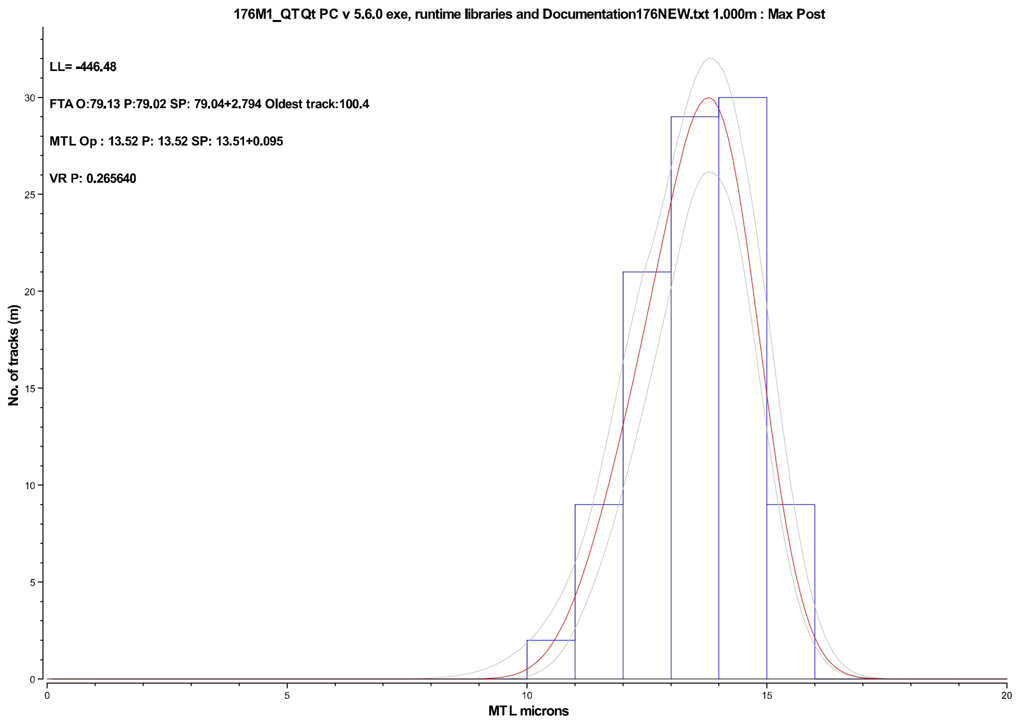
176M1_QTQt PC v 5.6.0 exe, runtime libraries and Documentation176NEW.txt 1.000m : Max Post
LL= -446.48
FTA O:79.13 P:79.02 SP: 79.04+2.794 Oldest track:100.4
MTL Op : 13.52 P: 13.52 SP: 13.51+0.095
VR P: 0.265640
No. of tracks (m)
MTL microns

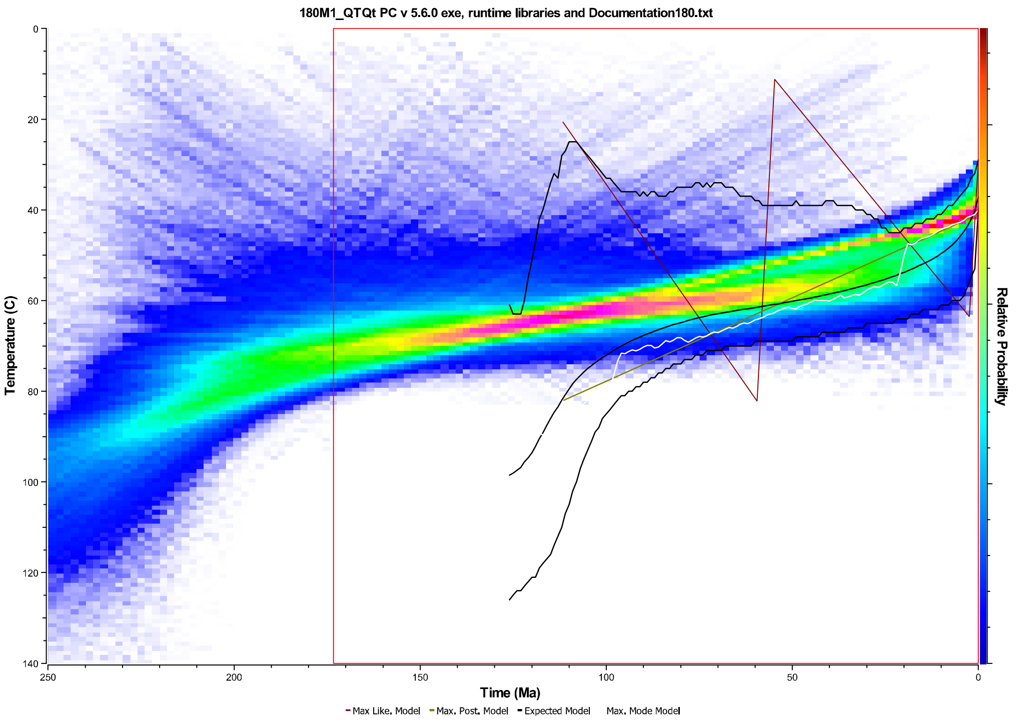
180M1_QTQt PC v 5.6.0 exe, runtime libraries and Documentation180.txt
Temperature (C)
Time (Ma)
Relative Probability
Max Like. Model
Max. Post. Model
Expected Model
Max. Mode Model

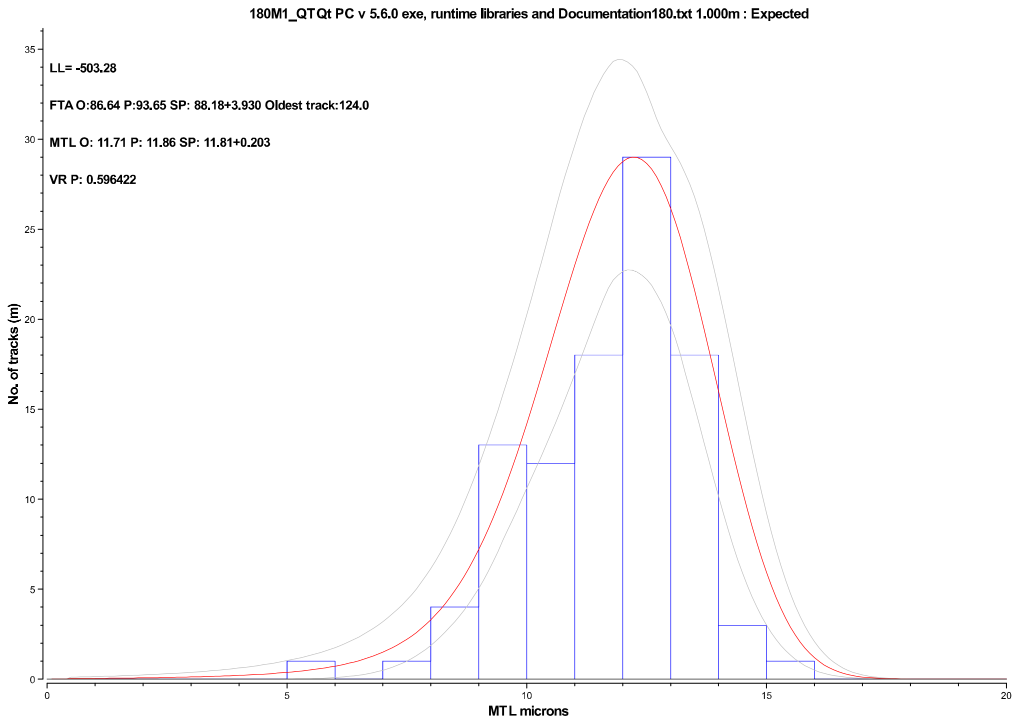
180M1_QTQt PC v 5.6.0 exe, runtime libraries and Documentation180.txt 1.000m : Expected
LL= -503.28
FTA O:86.64 P:93.65 SP: 88.18+3.930 Oldest track:124.0
MTL O: 11.71 P: 11.86 SP: 11.81+0.203
VR P: 0.596422
No. of tracks (m)
MTL microns

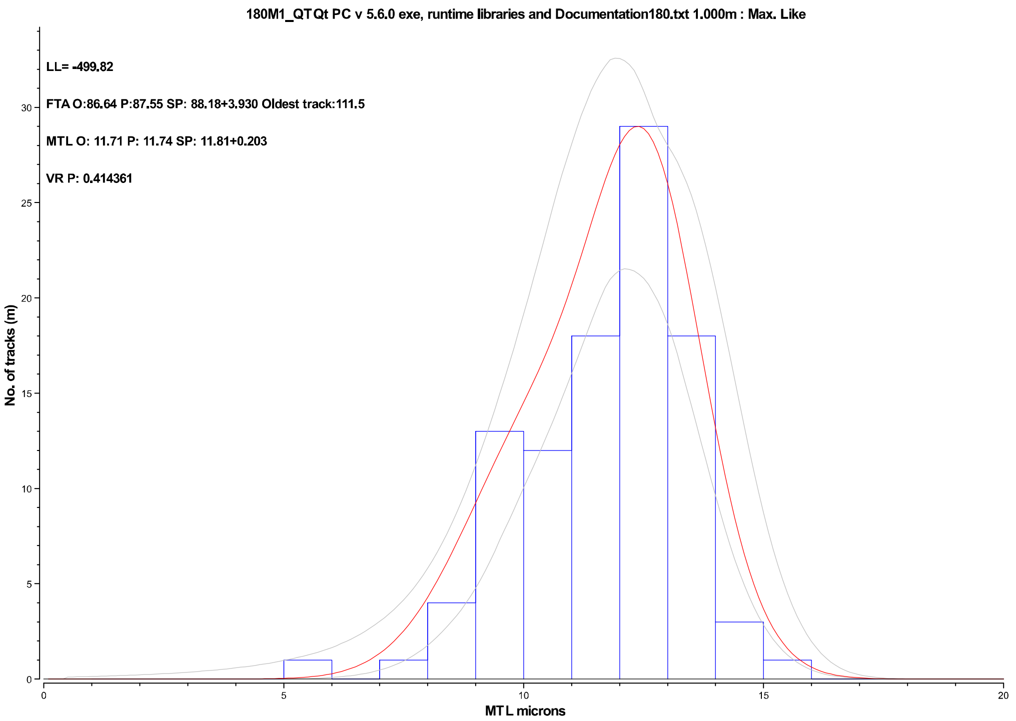
180M1_QTQt PC v 5.6.0 exe, runtime libraries and Documentation180.txt 1.000m : Max. Like
LL= -499.82
FTA O:86.64 P:87.55 SP: 88.18+3.930 Oldest track:111.5
MTL O: 11.71 P: 11.74 SP: 11.81+0.203
VR P: 0.414361
No. of tracks (m)
MTL microns

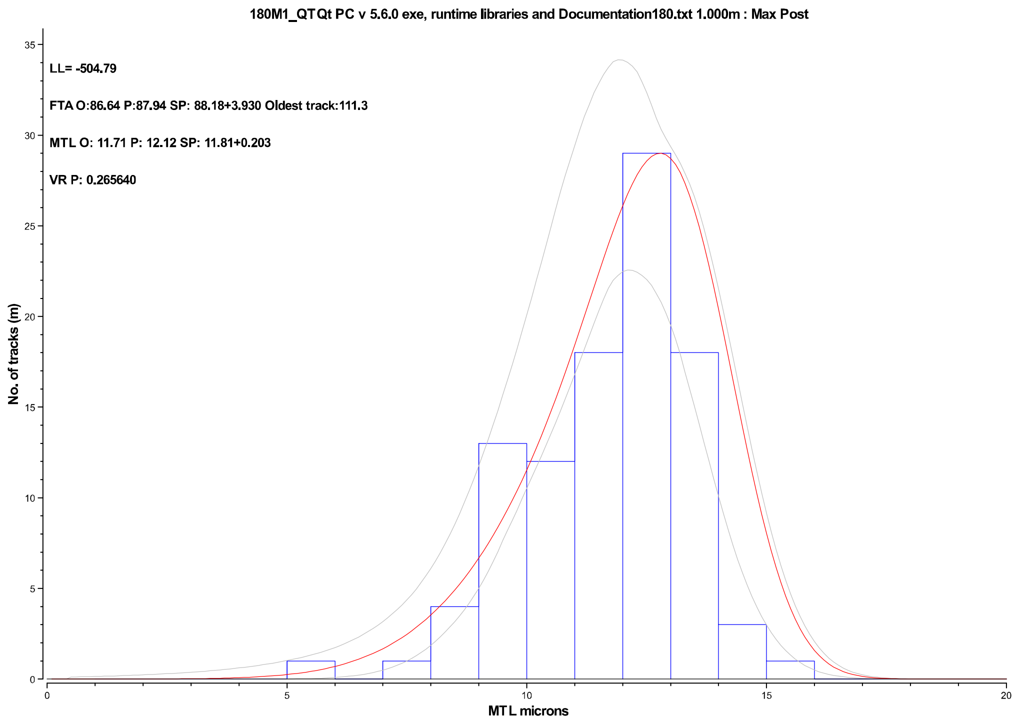
180M1_QTQt PC v 5.6.0 exe, runtime libraries and Documentation180.txt 1.000m : Max Post
LL= -504.79
FTA O:86.64 P:87.94 SP: 88.18+3.930 Oldest track:111.3
MTL O: 11.71 P: 12.12 SP: 11.81+0.203
VR P: 0.265640
No. of tracks (m)
MTL microns

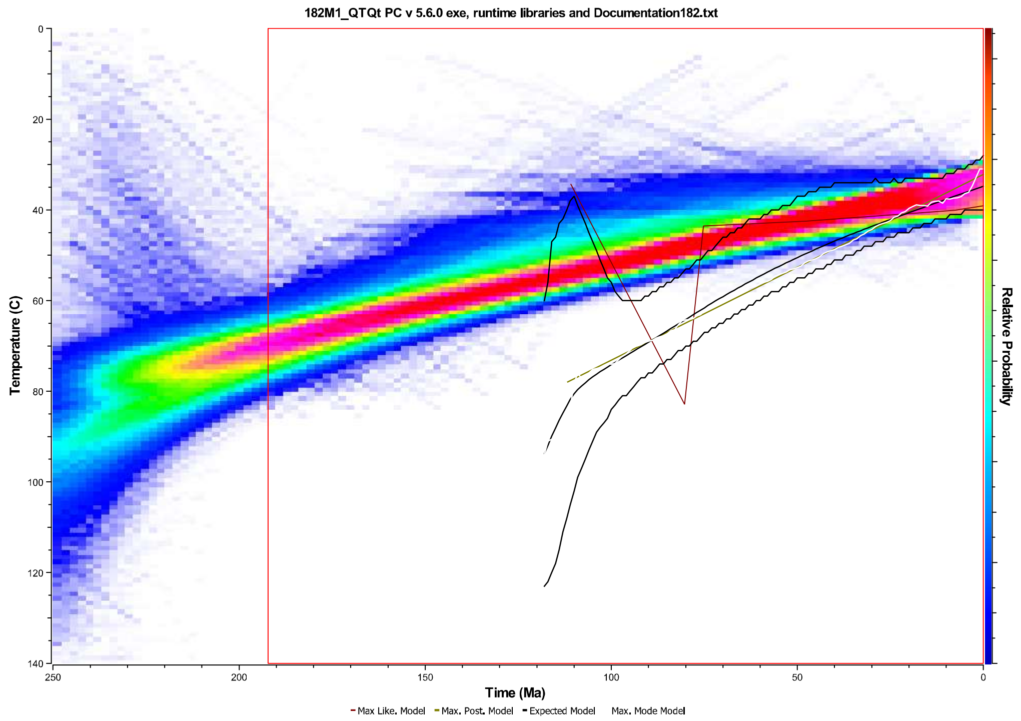
182M1_QTQt PC v 5.6.0 exe, runtime libraries and Documentation182.txt
Temperature (C)
Time (Ma)
Relative Probability
Max Like. Model
Max. Post. Model
Expected Model
Max. Mode Model

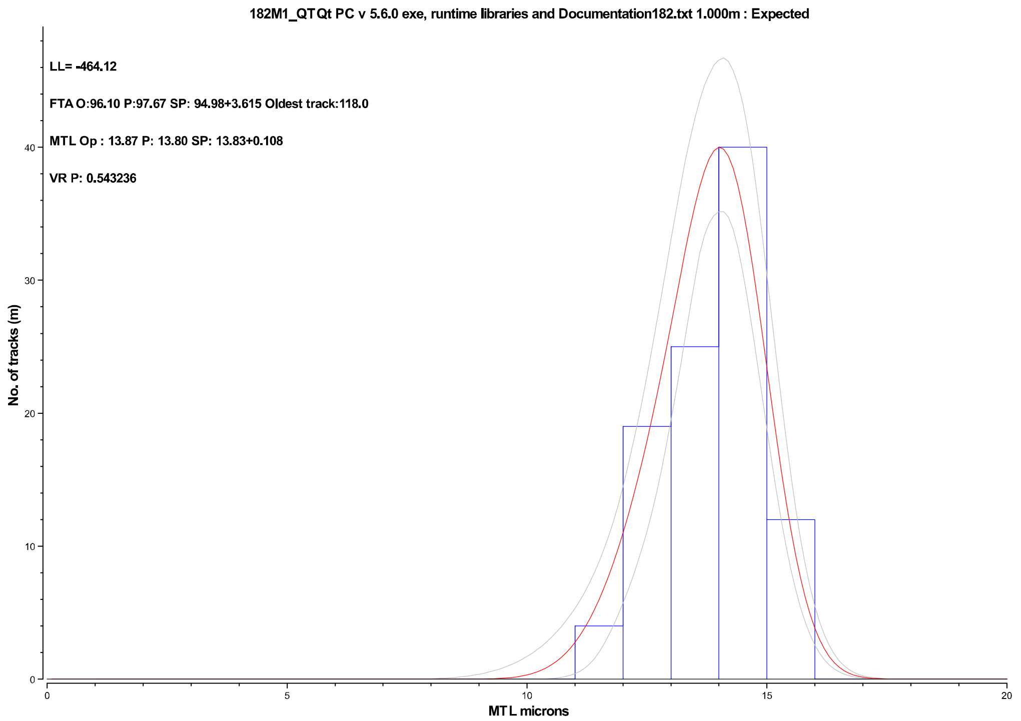
182M1_QTQt PC v 5.6.0 exe, runtime libraries and Documentation182.txt 1.000m : Expected
LL= -464.12
FTA O:96.10 P:97.67 SP: 94.98+3.615 Oldest track:118.0
MTL Op : 13.87 P: 13.80 SP: 13.83+0.108
VR P: 0.543236
No. of tracks (m)
MTL microns

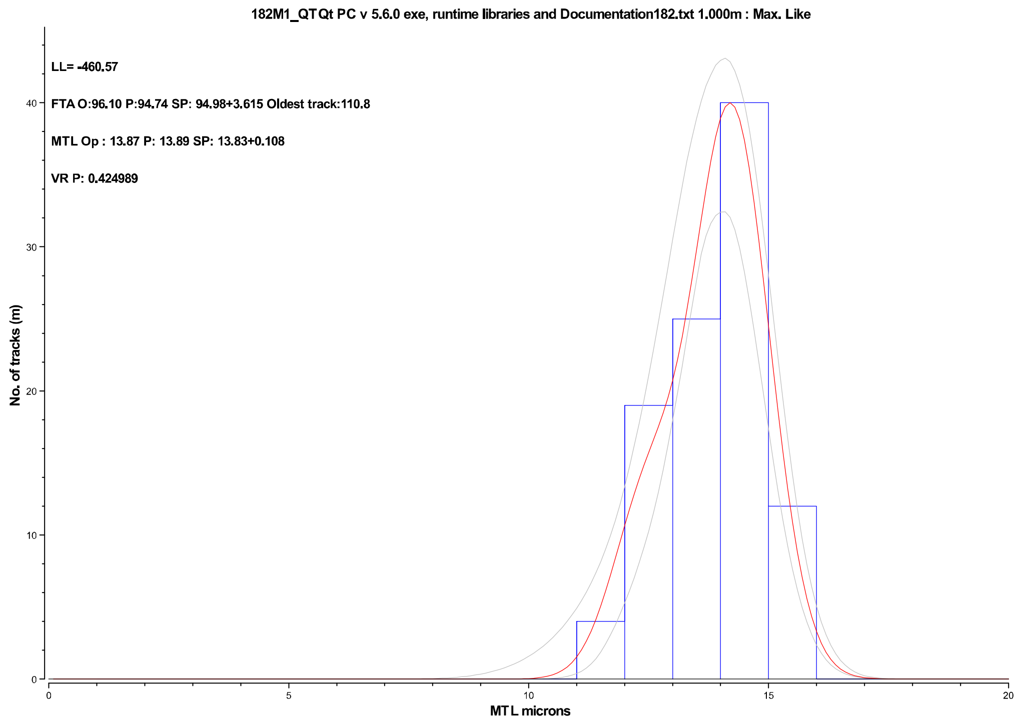
182M1_QTQt PC v 5.6.0 exe, runtime libraries and Documentation182.txt 1.000m : Max. Like
LL= -460.57
FTA O:96.10 P:94.74 SP: 94.98+3.615 Oldest track:110.8
MTL Op : 13.87 P: 13.89 SP: 13.83+0.108
VR P: 0.424989
No. of tracks (m)
MTL microns

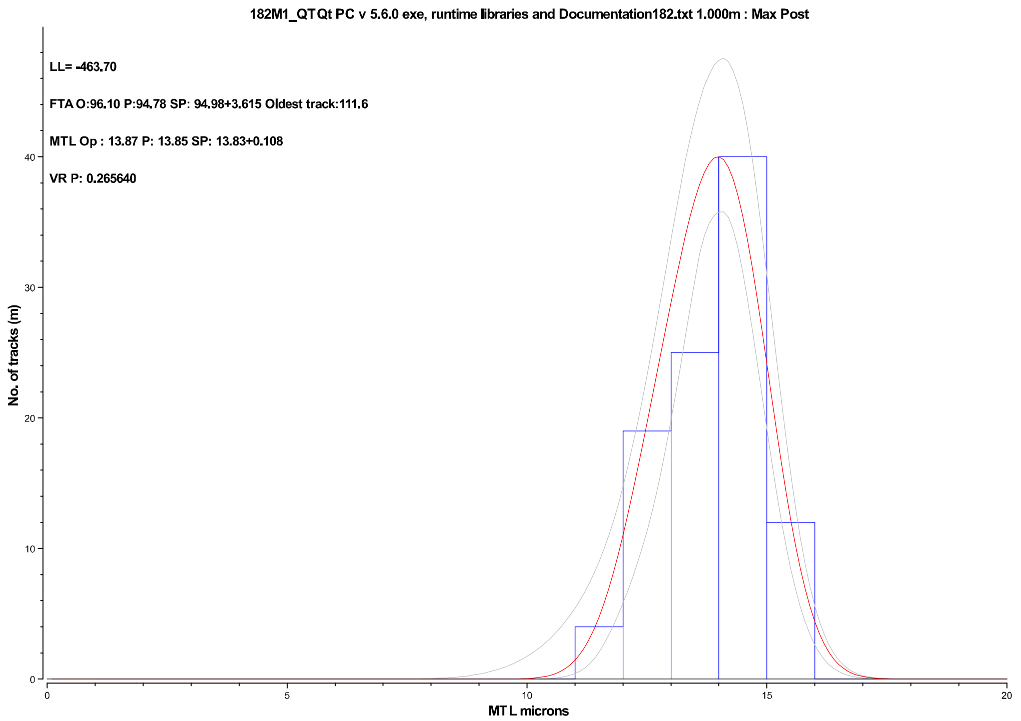
182M1_QTQt PC v 5.6.0 exe, runtime libraries and Documentation182.txt 1.000m : Max Post
LL= -463.70
FTA O:96.10 P:94.78 SP: 94.98+3.615 Oldest track:111.6
MTL Op : 13.87 P: 13.85 SP: 13.83+0.108
VR P: 0.265640
No. of tracks (m)
MTL microns

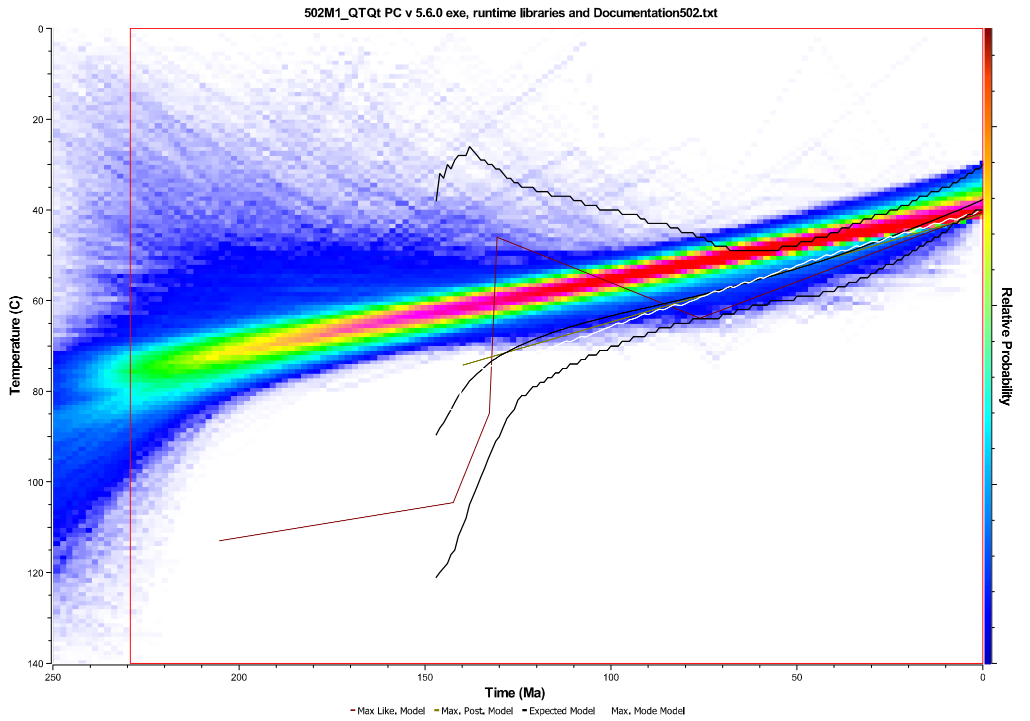
502M1_QTQt PC v 5.6.0 exe, runtime libraries and Documentation502.txt
Temperature (C)
Time (Ma)
Relative Probability
Max Like. Model
Max. Post. Model
Expected Model
Max. Mode Model

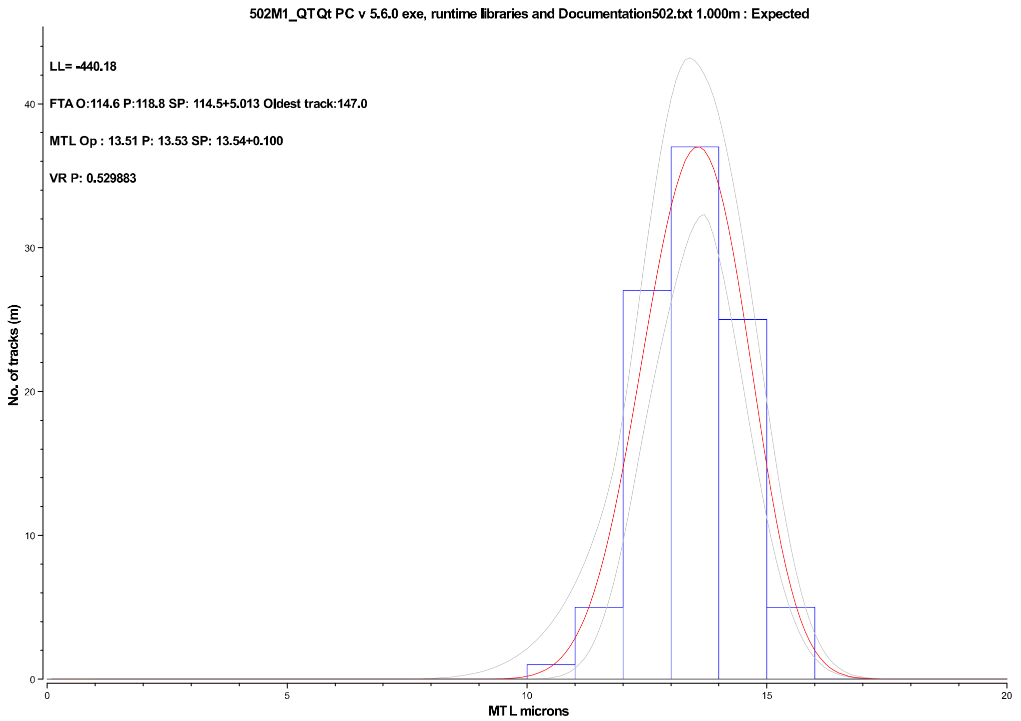
502M1_QTQt PC v 5.6.0 exe, runtime libraries and Documentation502.txt 1.000m : Expected
LL= -440.18
FTA O:114.6 P:118.8 SP: 114.5+5.013 Oldest track:147.0
MTL Op : 13.51 P: 13.53 SP: 13.54+0.100
VR P: 0.529883
No. of tracks (m)
MTL microns

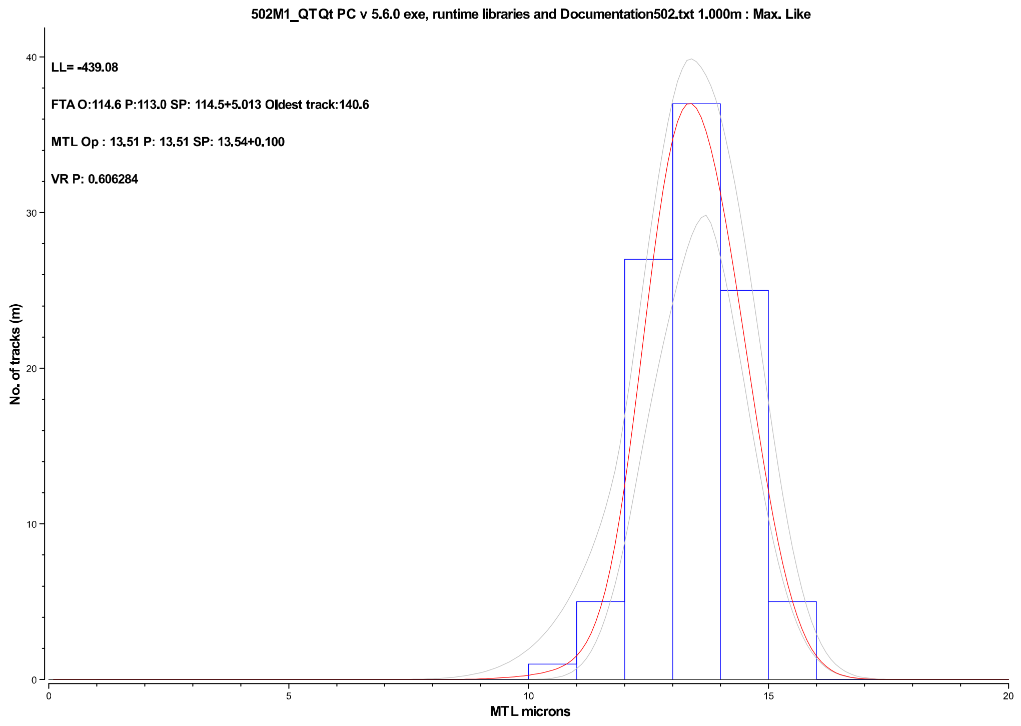
502M1_QTQt PC v 5.6.0 exe, runtime libraries and Documentation502.txt 1.000m : Max. Like
LL= -439.08
FTA O:114.6 P:113.0 SP: 114.5+5.013 Oldest track:140.6
MTL Op : 13.51 P: 13.51 SP: 13.54+0.100
VR P: 0.606284
No. of tracks (m)
MTL microns

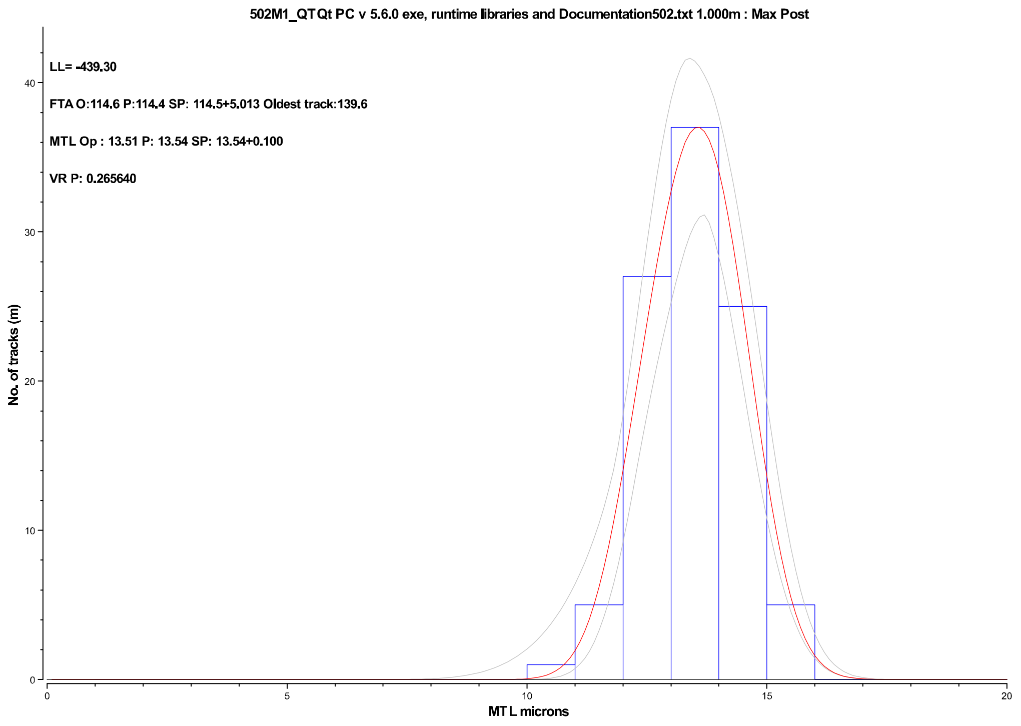
502M1_QTQt PC v 5.6.0 exe, runtime libraries and Documentation502.txt 1.000m : Max Post
LL= -439.30
FTA O:114.6 P:114.4 SP: 114.5+5.013 Oldest track:139.6
MTL Op : 13.51 P: 13.54 SP: 13.54+0.100
VR P: 0.265640
No. of tracks (m)
MTL microns

505_QTQt PC v 5.6.0 exe, runtime libraries and Documentation505.txt

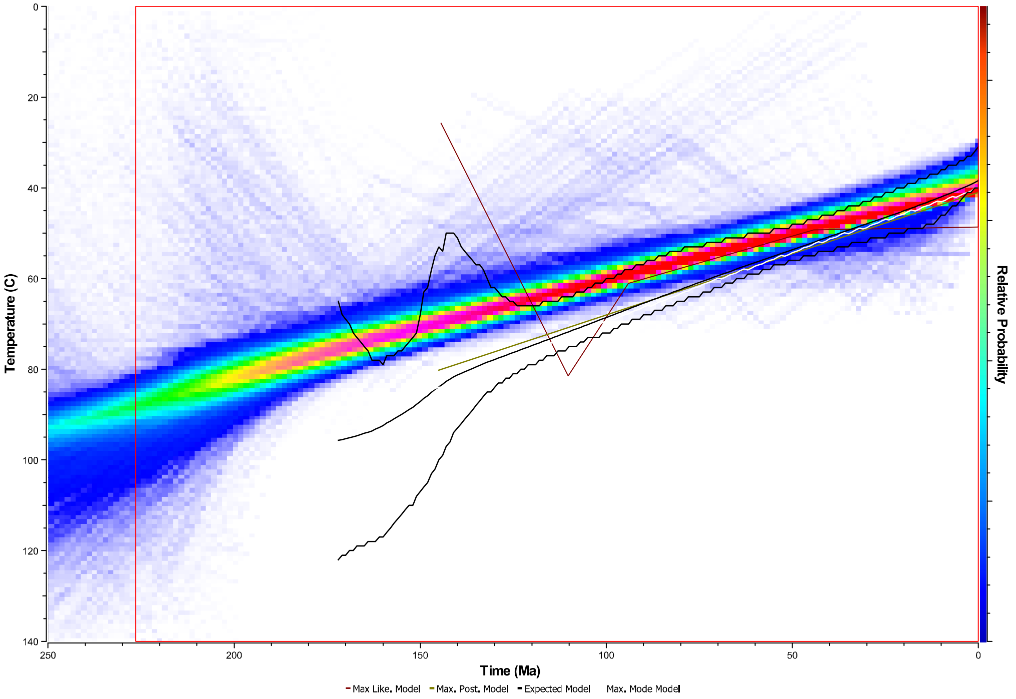

505_QTQt PC v 5.6.0 exe, runtime libraries and Documentation505.txt 1.000m : Expected

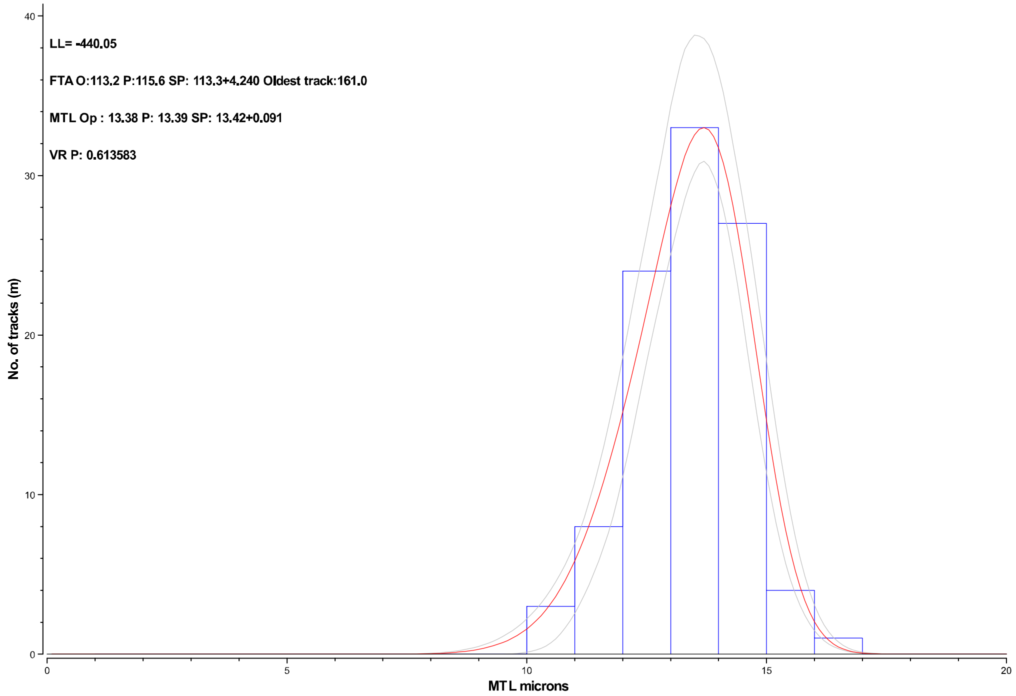

505_QTQt PC v 5.6.0 exe, runtime libraries and Documentation505.txt 1.000m : Max. Like

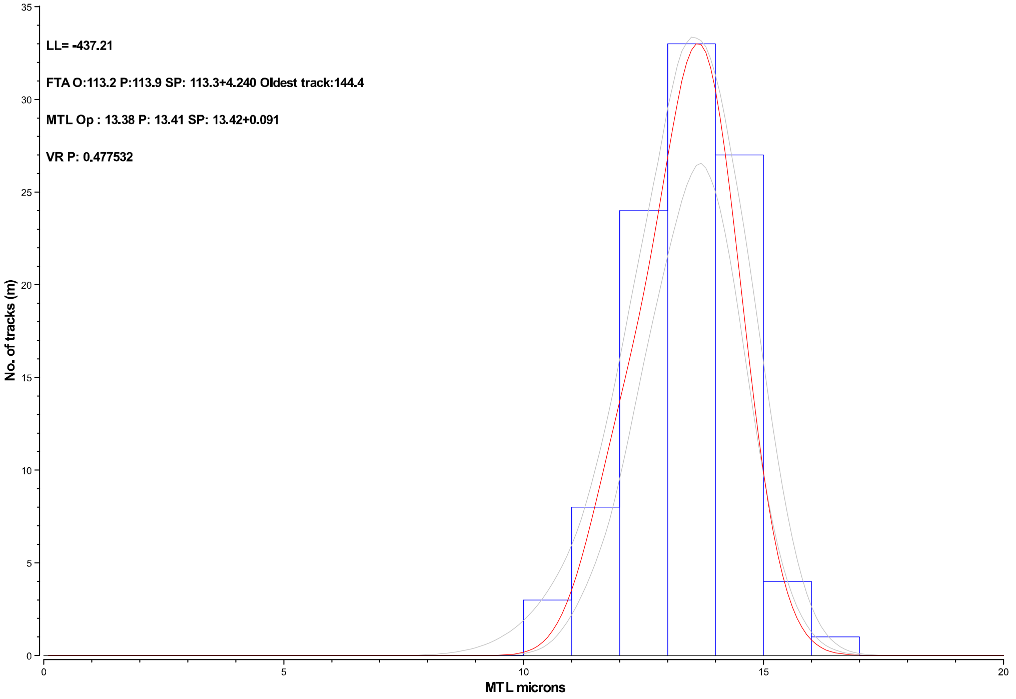

505_QTQt PC v 5.6.0 exe, runtime libraries and Documentation505.txt 1.000m : Max Post

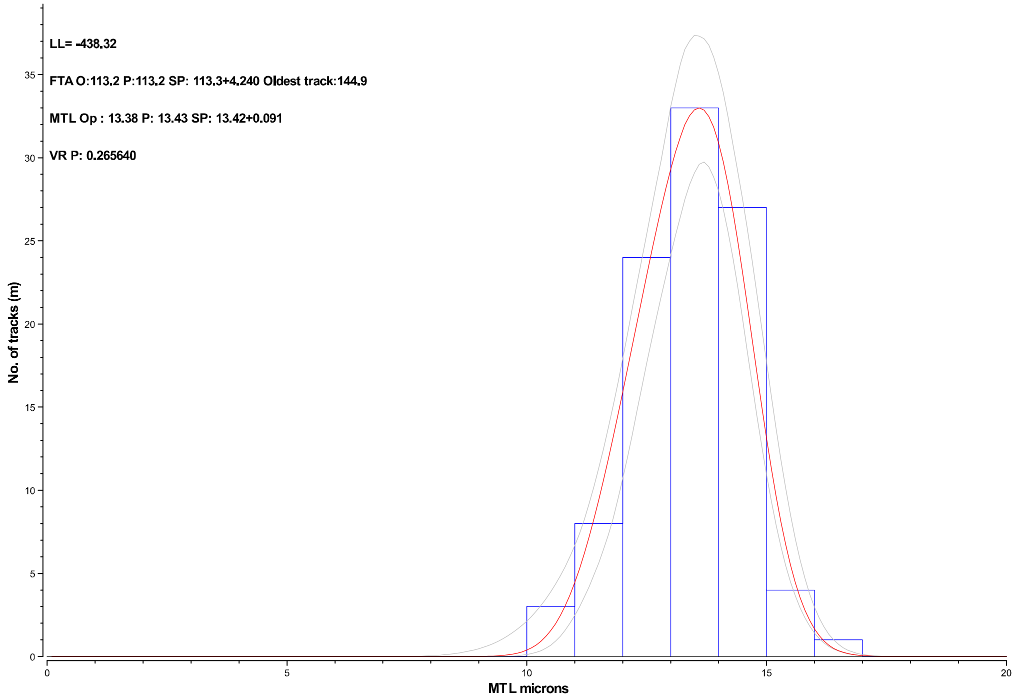

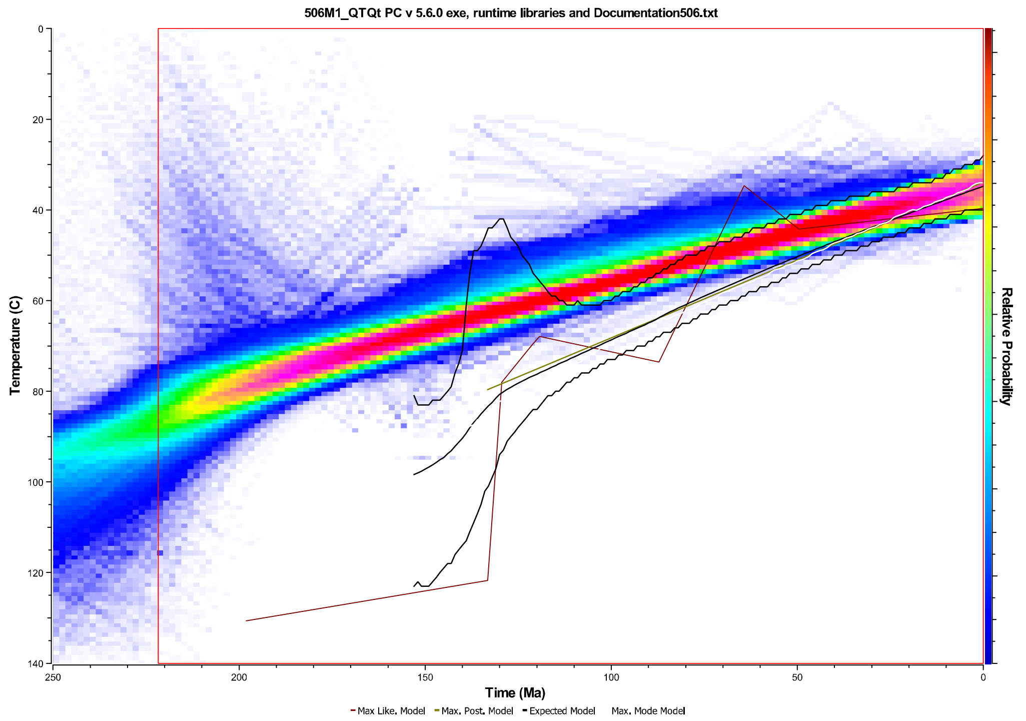
506M1_QTQt PC v 5.6.0 exe, runtime libraries and Documentation506.txt
Temperature (C)
Time (Ma)
Relative Probability
Max Like. Model
Max. Post. Model
Expected Model
Max. Mode Model

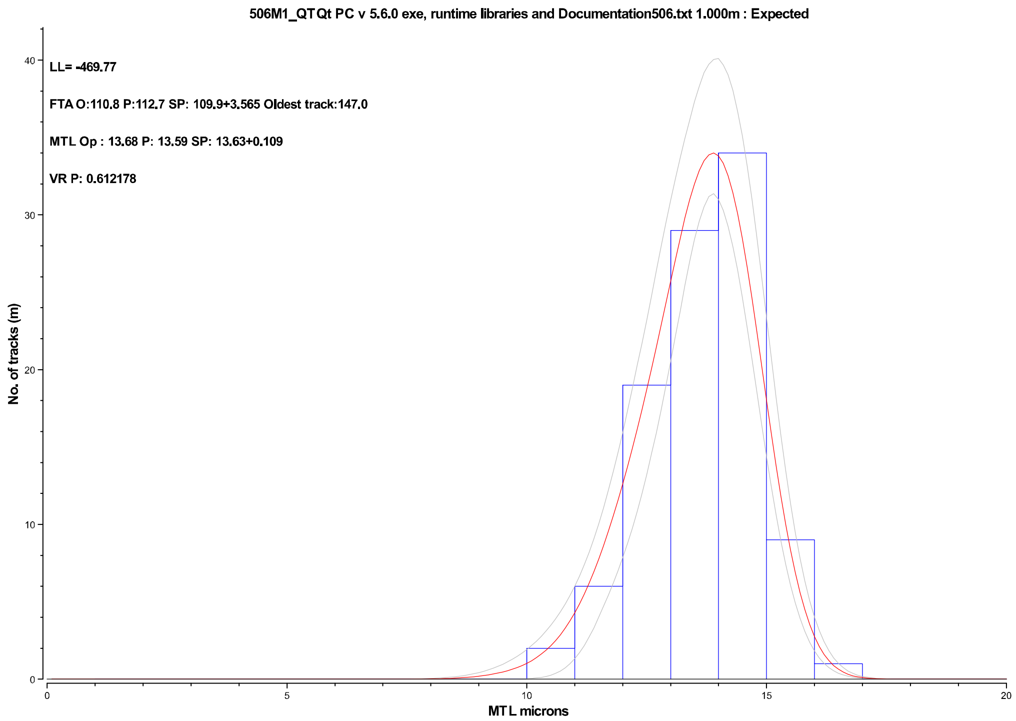
506M1_QTQt PC v 5.6.0 exe, runtime libraries and Documentation506.txt 1.000m : Expected
LL= -469.77
FTA O:110.8 P:112.7 SP: 109.9+3.565 Oldest track:147.0
MTL Op : 13.68 P: 13.59 SP: 13.63+0.109
VR P: 0.612178
No. of tracks (m)
MTL microns

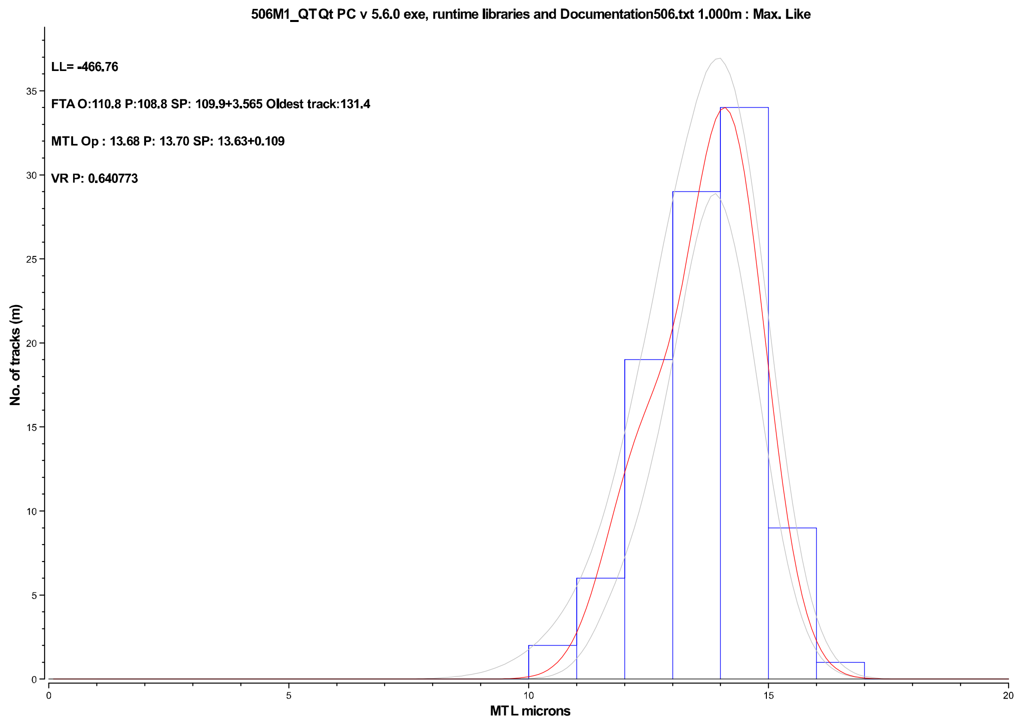
506M1_QTQt PC v 5.6.0 exe, runtime libraries and Documentation506.txt 1.000m : Max. Like
LL= -466.76
FTA O:110.8 P:108.8 SP: 109.9+3.565 Oldest track:131.4
MTL Op : 13.68 P: 13.70 SP: 13.63+0.109
VR P: 0.640773
No. of tracks (m)
MTL microns

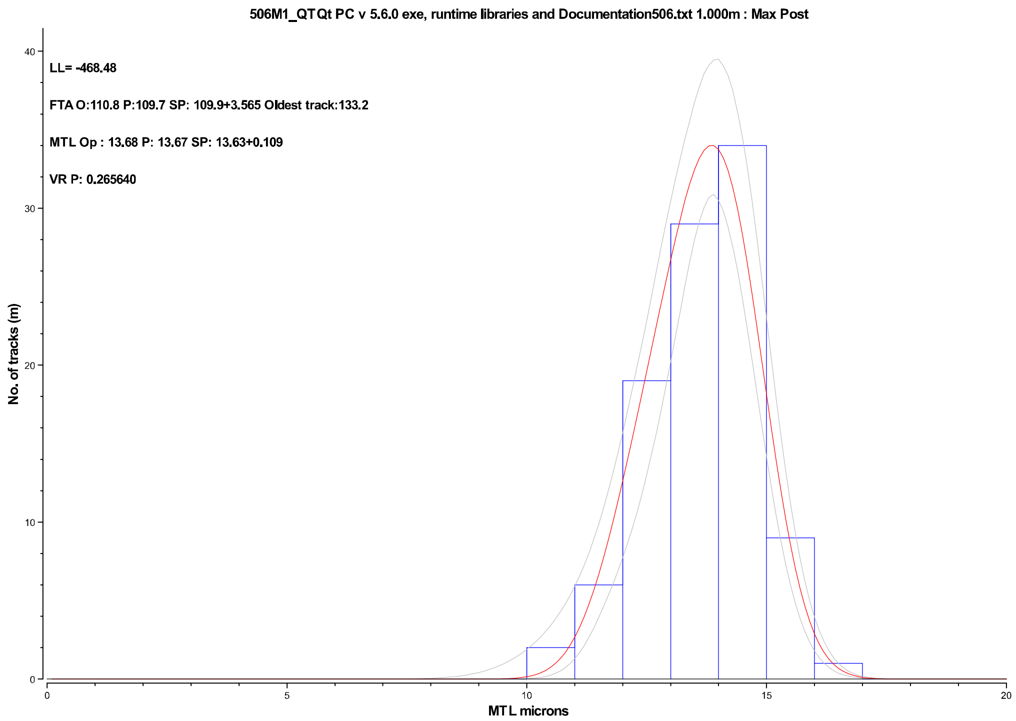
506M1_QTQt PC v 5.6.0 exe, runtime libraries and Documentation506.txt 1.000m : Max Post
LL= -468.48
FTA O:110.8 P:109.7 SP: 109.9+3.565 Oldest track:133.2
MTL Op : 13.68 P: 13.67 SP: 13.63+0.109
VR P: 0.265640
No. of tracks (m)
MTL microns

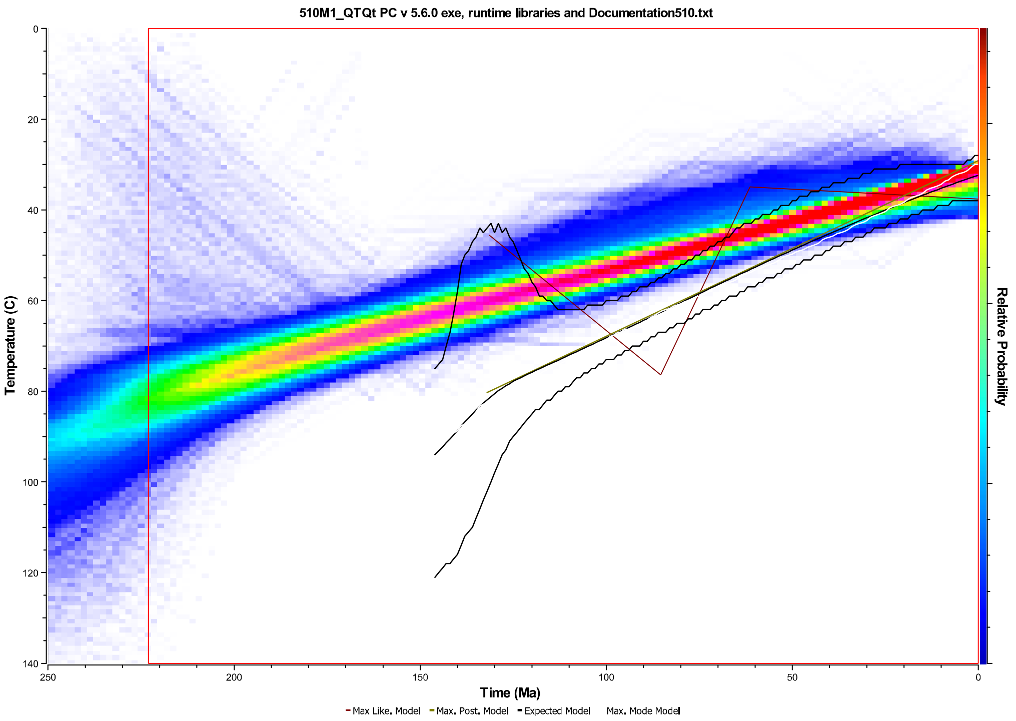
510M1_QTQt PC v 5.6.0 exe, runtime libraries and Documentation510.txt
Temperature (C)
Time (Ma)
Relative Probability
Max Like. Model
Max. Post. Model
Expected Model
Max. Mode Model

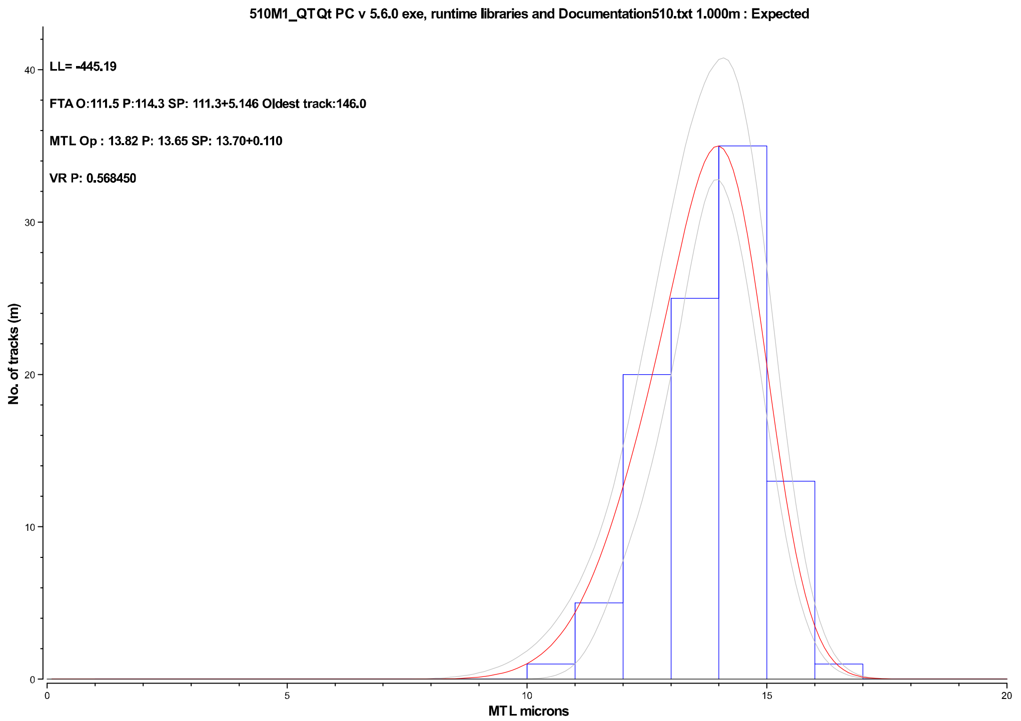
510M1_QTQt PC v 5.6.0 exe, runtime libraries and Documentation510.txt 1.000m : Expected
LL= -445.19
FTA O:111.5 P:114.3 SP: 111.3+5.146 Oldest track:146.0
MTL Op : 13.82 P: 13.65 SP: 13.70+0.110
VR P: 0.568450
No. of tracks (m)
MTL microns

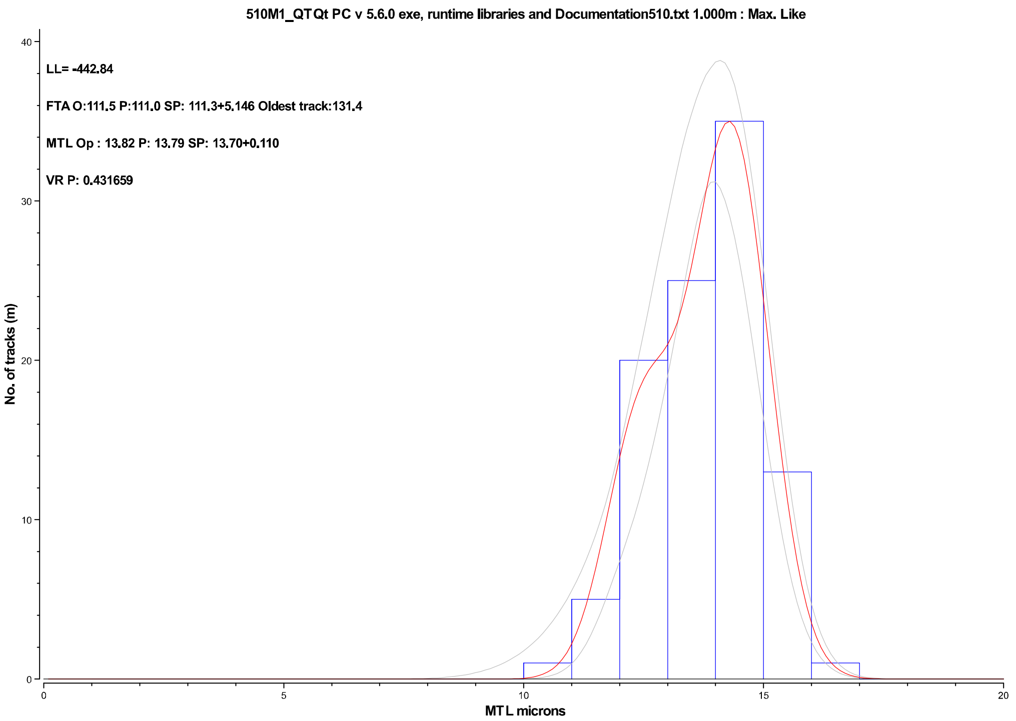
510M1_QTQt PC v 5.6.0 exe, runtime libraries and Documentation510.txt 1.000m : Max. Like
LL= -442.84
FTA O:111.5 P:111.0 SP: 111.3+5.146 Oldest track:131.4
MTL Op : 13.82 P: 13.79 SP: 13.70+0.110
VR P: 0.431659
No. of tracks (m)
MTL microns

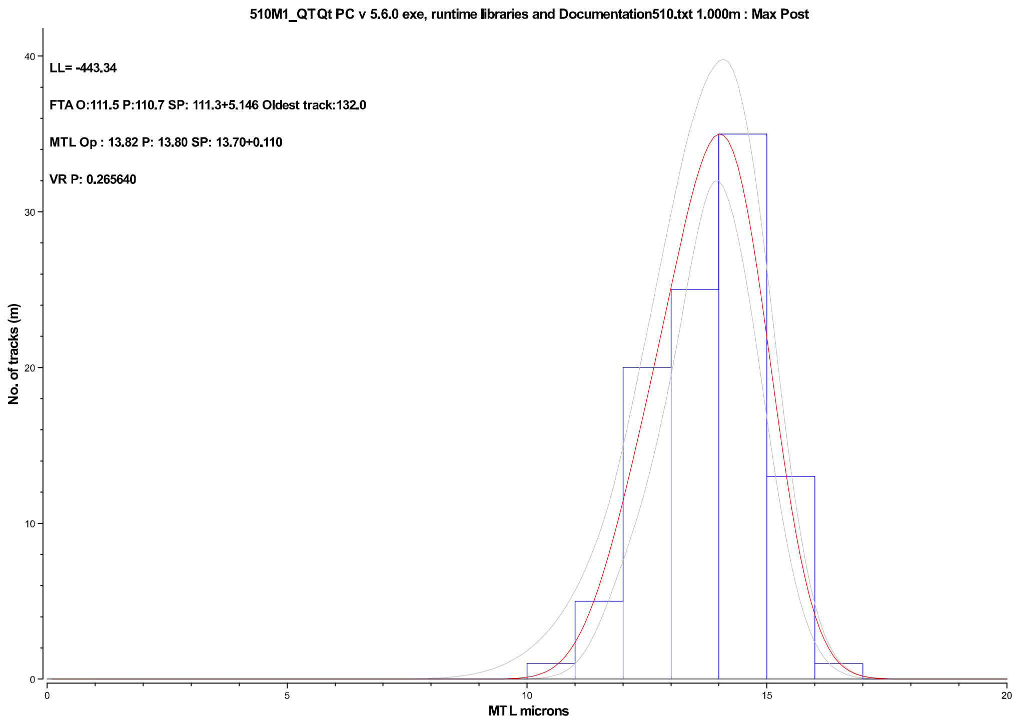
510M1_QTQt PC v 5.6.0 exe, runtime libraries and Documentation510.txt 1.000m : Max Post
LL= -443.34
FTA O:111.5 P:110.7 SP: 111.3+5.146 Oldest track:132.0
MTL Op : 13.82 P: 13.80 SP: 13.70+0.110
VR P: 0.265640
No. of tracks (m)
MTL microns

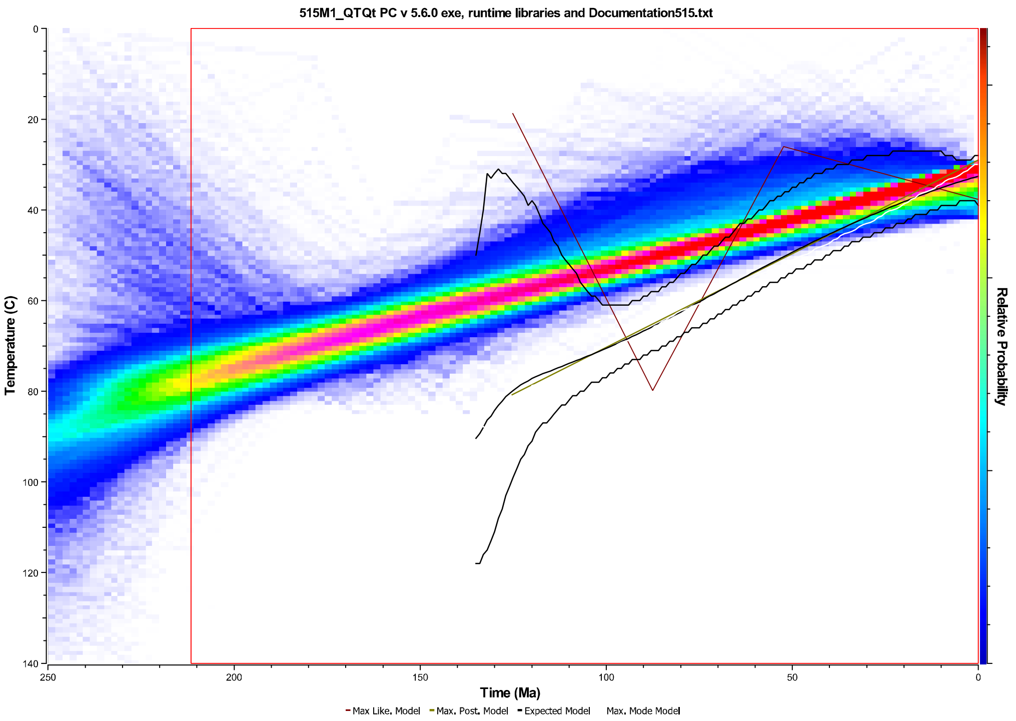
515M1_QTQt PC v 5.6.0 exe, runtime libraries and Documentation515.txt
Temperature (C)
Time (Ma)
Relative Probability
Max Like. Model
Max. Post. Model
Expected Model
Max. Mode Model

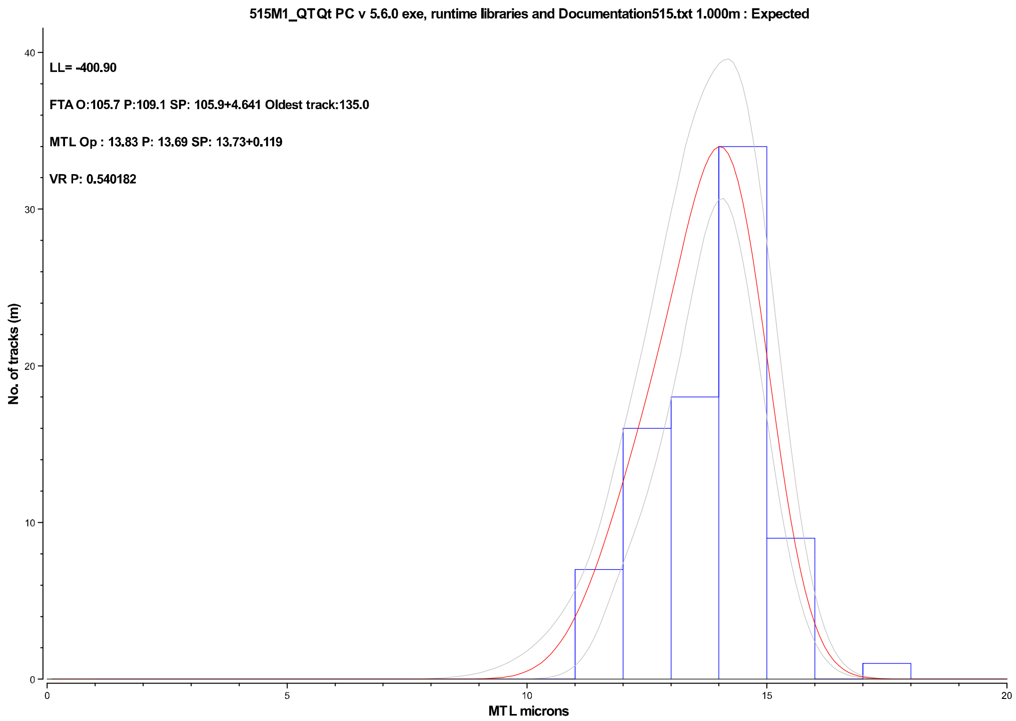
515M1_QTQt PC v 5.6.0 exe, runtime libraries and Documentation515.txt 1.000m : Expected
LL= -400.90
FTA O:105.7 P:109.1 SP: 105.9+4.641 Oldest track:135.0
MTL Op : 13.83 P: 13.69 SP: 13.73+0.119
VR P: 0.540182
No. of tracks (m)
MTL microns

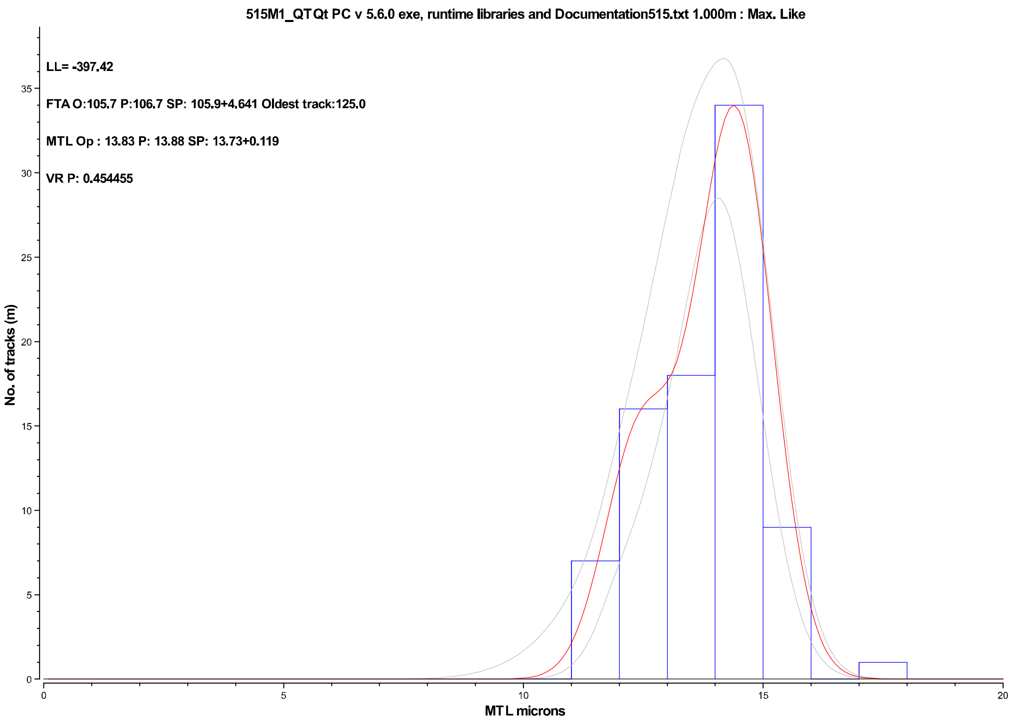
515M1_QTQt PC v 5.6.0 exe, runtime libraries and Documentation515.txt 1.000m : Max. Like
LL= -397.42
FTA O:105.7 P:106.7 SP: 105.9+4.641 Oldest track:125.0
MTL Op : 13.83 P: 13.88 SP: 13.73+0.119
VR P: 0.454455
No. of tracks (m)
MTL microns

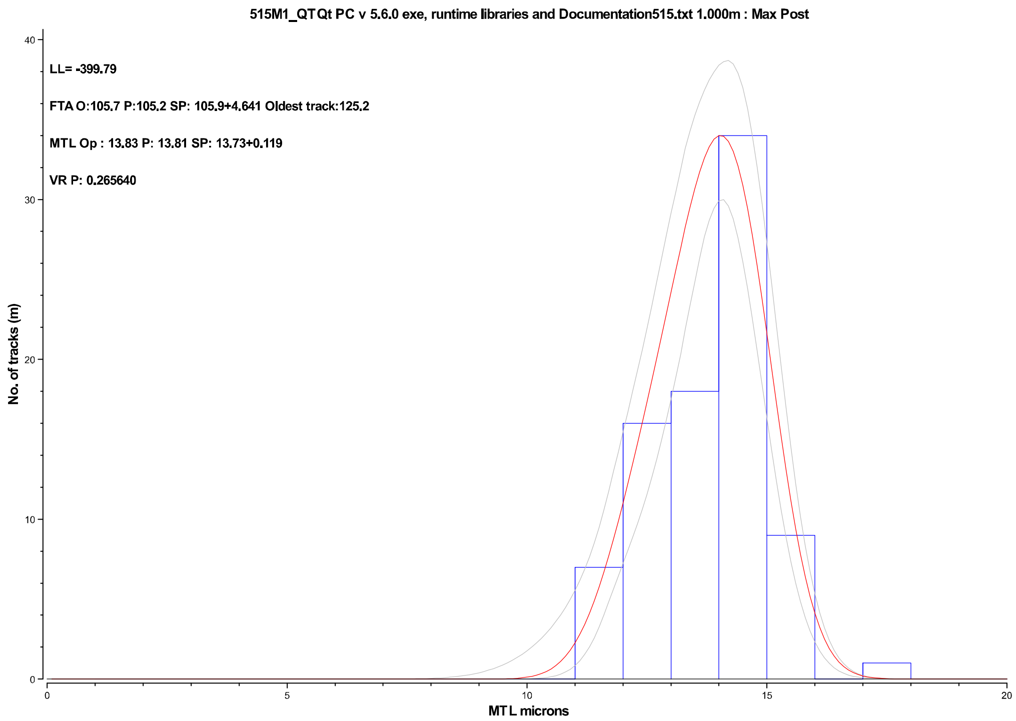
515M1_QTQt PC v 5.6.0 exe, runtime libraries and Documentation515.txt 1.000m : Max Post
LL= -399.79
FTA O:105.7 P:105.2 SP: 105.9+4.641 Oldest track:125.2
MTL Op : 13.83 P: 13.81 SP: 13.73+0.119
VR P: 0.265640
No. of tracks (m)
MTL microns
